# Catalytic Enantioselective Synthesis of α-Difunctionalized Cyclic Sulfones

**DOI:** 10.1021/acs.joc.2c01240

**Published:** 2022-07-08

**Authors:** Eleanor Bowen, Gillian Laidlaw, Bethany C. Atkinson, Timur A. McArdle-Ismaguilov, Vilius Franckevičius

**Affiliations:** Department of Chemistry, Lancaster University, Lancaster LA1 4YB, U.K.

## Abstract

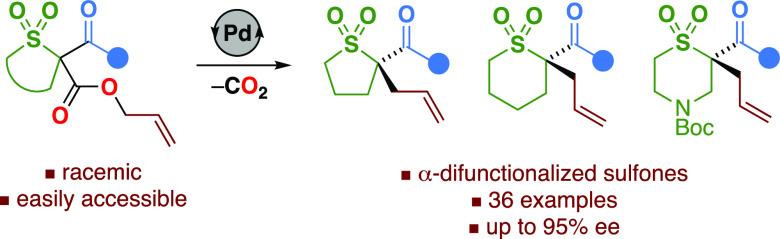

As saturated heterocyclic building blocks become increasingly popular in medicinal chemistry and drug discovery programs, expansion of the synthetic toolkit to novel stereofunctionalized heterocycles is a priority. Herein, we report the development of a palladium-catalyzed decarboxylative asymmetric allylic alkylation reaction to access a broad range of enantioenriched α-difunctionalized 5- and 6-membered sulfones from easily accessible racemic starting materials. The allylic alkylation step was found to occur with high levels of enantioselectivity as a result of a palladium-mediated dynamic kinetic resolution of *E*/*Z* enolate intermediates. This methodology paves the way to hitherto unexplored stereodefined cyclic sulfones for medicinal chemistry applications.

## Introduction

Heterocycles have been and remain to be fundamental building blocks of the majority of small molecule drugs.^1^ In order to enhance the developability of lead compounds and examine previously untapped areas of chemical and biological space, saturated heterocycles are becoming increasingly important in medicinal chemistry.^2^ In particular, new asymmetric synthetic methods are sought after to access novel stereofunctionalized heterocycles as high value motifs for drug discovery.^3^

**Figure 1:**
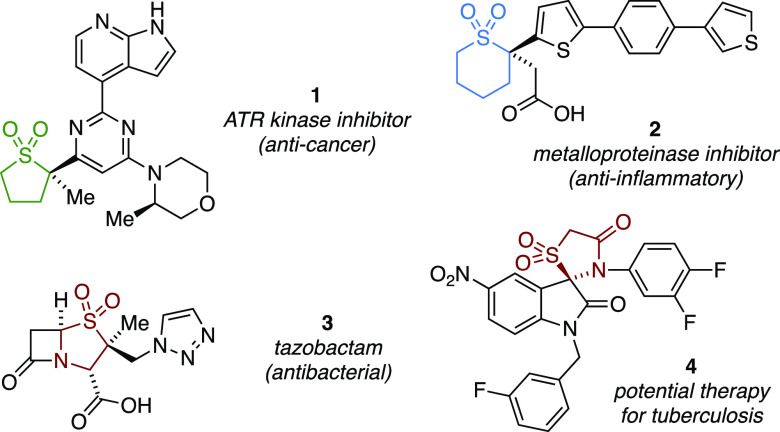
Enantioenriched α-disubstituted cyclic sulfones.

Saturated cyclic sulfones bearing a tetrasubstituted α-sulfonyl stereogenic center are a principal motif of a number of biologically active compounds (Figure 1). For example, **1** and **2** are a patented ATR kinase inhibitor for cancer chemotherapy^4^ and a matrix metalloproteinase inhibitor as an anti-inflammatory agent,^5^ respectively. Similarly, tazobactam (**3**) is a very common modified penicillin that is used in the clinic as a β-lactamase inhibitor to combat bacterial resistance,^6^ whereas Waldmann and co-workers have discovered that spirocyclic **4** is a selective and potent *Mycobacterium tuberculosis* protein tyrosine phosphatase B inhibitor, where the *R* enantiomer of **4** (IC_50_ 0.32 mM) was found to be 10 times more active than (*S*)-**4**.^7^ As such, the development of new enantioselective approaches to install tetrasubstituted α-sulfonyl stereogenic centers is a pertinent area of research.^8^ To date, only a handful of strategies have been reported for the construction of enantioenriched α-difunctionalized 5-membered sulfones, namely, diastereoselectively by using enantiopure starting materials,^9^ or a chiral auxiliary,^10^ enantioselectively by oxidation of 1,3-dithiolanes,^11^ and cyclization of linear precursors by means of enantioselective organocatalysis,^12^ metal catalysis,^13^ and photocatalysis.^14^ In addition, there is only one report of an enantioselective entry to α-difunctionalized 6-membered sulfones,^15^ utilizing stereoselective oxidation of 1,3-dithianes. To the best of our knowledge, there are no enantioselective methods that would enable the direct α-difunctionalization of cyclic sulfones and construct a tetrasubstituted α-sulfonyl stereogenic center.

**Scheme 1:**
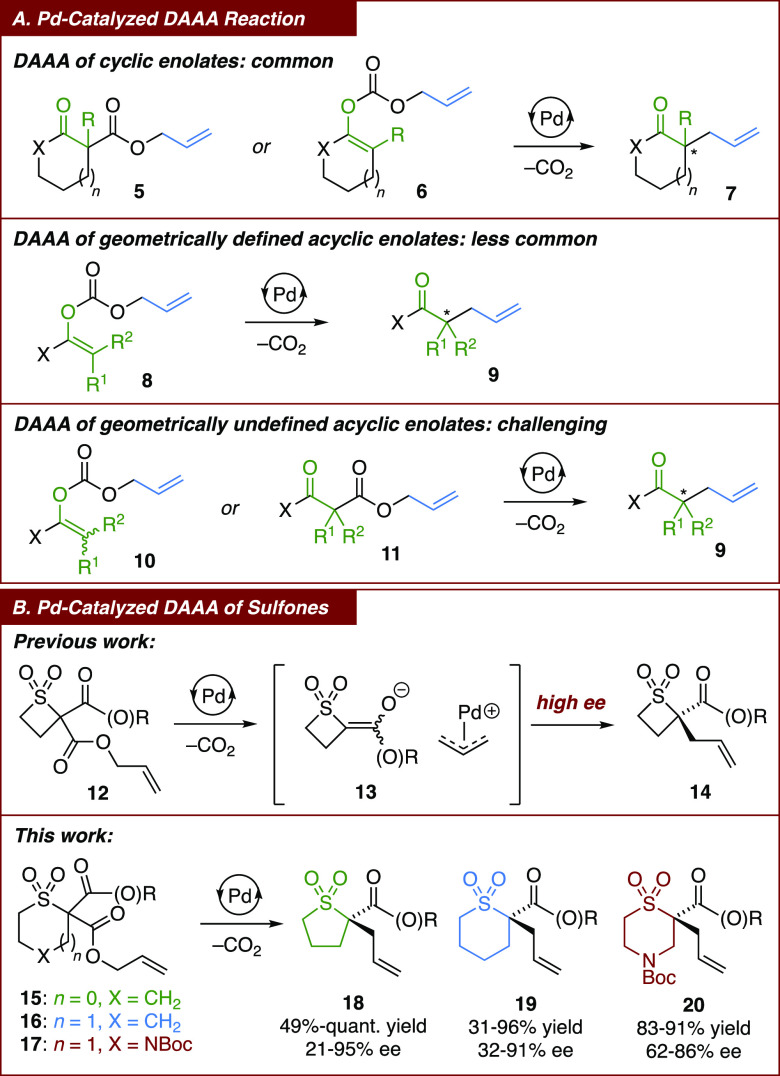
Pd-Catalyzed DAAA of Enolates

To install the α-sulfonyl stereocenter under mild, base-free conditions, we sought to explore the palladium-catalyzed decarboxylative asymmetric allylic alkylation (DAAA) reaction.^16^ Since the first report of the palladium-catalyzed DAAA reaction of ketone enolates with prochiral allylic electrophiles,^17^ this process has been most commonly used in the allylation of prochiral cyclic enolates, derived from allyl ester and allyl enol carbonate precursors **5** and **6**, respectively (A, Scheme 1).^18^ While the cyclic nature of the enolate intermediate typically affords high levels of stereocontrol in the construction of the quaternary stereocenter in **7**, the situation is more complex in the allylic alkylation of acyclic enolates: as the geometry of the enolate has an impact on the stereoselectivity of the reaction, the enolate precursor **8** must have a defined alkene geometry to ensure high levels of enantioselectivity in the formation of **9**.^19^ If a mixture of geometrical isomers of allyl enol carbonate **10** is used or a linear allyl ester substrate **11** affords a mixture of *E*/*Z* enolates *in situ* following decarboxylation,^20^ then only low levels of enantioselectivity would be expected to result in the formation of **9**. Due to the challenges associated with the preparation of geometrically pure allyl enol carbonate starting materials **8**, the palladium-catalyzed DAAA reaction of acyclic enolates is less common. Notwithstanding, Murakami and co-workers have been able to obtain **9** with high ee from linear precursors **11** due to coordinating effects in the transition state of alkylation,^21^ whereas Stoltz and co-workers have observed an unusual palladium-mediated dynamic kinetic resolution (DKR) of *E*/*Z* enolate intermediates,^22^ giving **9** with high ee from either allyl enol carbonate **10**, irrespective of its alkene geometry, or β-carbonyl ester **11**.

Alongside enolates, α-sulfonyl anions are also known to undergo palladium- and iridium-catalyzed asymmetric allylic alkylation,^23^ but these processes focus primarily on the installation of an allylic, rather than α-sulfonyl, stereogenic center. Although Tunge and co-workers successfully developed a stereoretentive palladium-catalyzed decarboxylative allylation of sulfones to give tetrasubstituted α-sulfonyl stereocenters, the use of enantiopure starting materials was required.^24^ To construct enantioenriched tetrasubstituted α-sulfonyl carbon centers from achiral or racemic starting materials, we developed the first palladium-catalyzed DAAA reaction that affords α-difunctionalized cyclic sulfones, namely, thietane 1,1-dioxides **14**, from racemic β-carbonyl sulfones **12**.^25^ Despite the implication of a mixture of *E*/*Z* enolates **13**, this reaction was found to proceed with high levels of stereoselectivity owing to the aforementioned palladium-mediated DKR of enolates. Herein, we describe the development of the palladium-catalyzed DAAA reaction of racemic 5- and 6-membered sulfones **15**–**17** in order to access enantioenriched α-difunctionalized sulfolanes **18**, thiane 1,1-dioxides **19**, and thiomorpholine 1,1-dioxides **20** without the need for pre-formed geometrically pure allyl enol carbonate starting materials.

## Results and Discussion

**Scheme 2:**
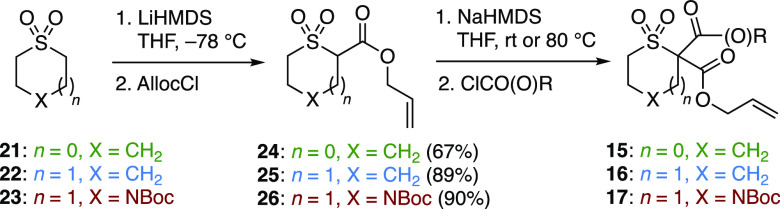
Substrate Synthesis

To investigate the palladium-catalyzed DAAA reaction in detail, three substrate classes were prepared in a divergent manner from the following cyclic sulfone scaffolds (Scheme 2): sulfolane (**21**), thiane 1,1-dioxide (**22**), and *N*-Boc thiomorpholine 1,1-dioxide (**23**). Sulfones **21**–**23** were appended with an allyl ester moiety in **24**–**26** in good yields. The reaction of the enolate of **24**–**26** with either a chloroformate or an acid chloride afforded a range of racemic ester- and ketone-substituted sulfone substrates **15**–**17**.

The optimization of the palladium-catalyzed DAAA reaction began with benzyl-ester-substituted sulfolane substrate **15a** (Table 1). When the reactions were run in THF as the solvent at room temperature in the presence of a set of ligands **L1**–**4**, PHOX ligand **L1** afforded **18a** in a racemic form (entry 1), whereas Trost ligands **L2** and **L3** gave poorly selective reactions (entries 2 and 3). The best result was obtained with (*S*,*S*)-ANDEN phenyl Trost ligand **L4** (entry 4), installing the tetrasubstituted α-stereogenic center in **18a** with 74% ee. Lowering the reaction temperature led to a small increase in selectivity (entry 5). A solvent screen indicated that DMF and acetonitrile were not selective (entries 6 and 7), whereas other solvents, such as toluene (entry 8), ethereal ones (entries 9–11), and chlorinated ones (entries 12 and 13), gave much higher selectivity. The best enantioselectivity of 86% ee was obtained with 1,4-dioxane as the solvent (entry 14), and the reaction was found to go to completion within 2 h (entry 15). Given the high freezing point of 1,4-dioxane, an attempt to lower the temperature of the reaction in a mixture of 1,4-dioxane with THF did not lead to an enhancement of ee.

**Table 1:**
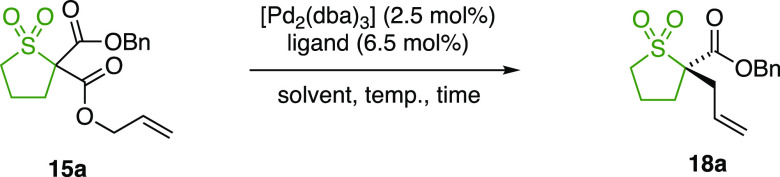
Reaction Optimization

entry^a^	solvent	ligand	temp.	time, h	yield, %^b^	ee, %^c^
1	THF	**L1**	rt	24	83	0
2	THF	**L2**	rt	24	78	11
3	THF	**L3**	rt	24	61	31
4	THF	**L4**	rt	24	77	74
5	THF	**L4**	–20 °C	48	70	77
6	DMF	**L4**	rt	24	84	–3
7	MeCN	**L4**	rt	24	84	13
8	toluene	**L4**	rt	24	68	61
9	MTBE	**L4**	rt	24	75	63
10	Et_2_O	**L4**	rt	24	79	65
11	DME	**L4**	rt	24	79	74
12	CH_2_Cl_2_	**L4**	rt	24	79	72
13	CHCl_3_	**L4**	rt	24	78	78
14	1,4-dioxane	**L4**	rt	24	88	86
15	1,4-dioxane	**L4**	rt	2	91	86
16	THF:1,4-dioxane 1:1	**L4**	–20 °C	48	75	81

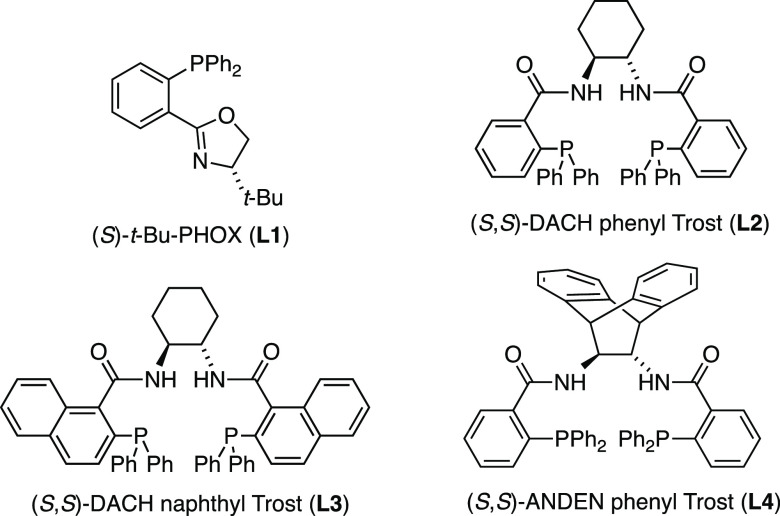

a Reaction performed with **15a** (0.15 mmol), Pd_2_(dba)_3_ (3.75 μmol), and ligand (9.75 μmol) in solvent (1.5 mL, 0.1 M).

b Isolated yield.

c Determined by chiral HPLC. THF = tetrahydrofuran; DMF = *N*,*N*-dimethylformamide; MTBE = methyl *tert*-butyl ether; DME = 1,2-dimethoxyethane; rt = room temperature.

**Scheme 3:**
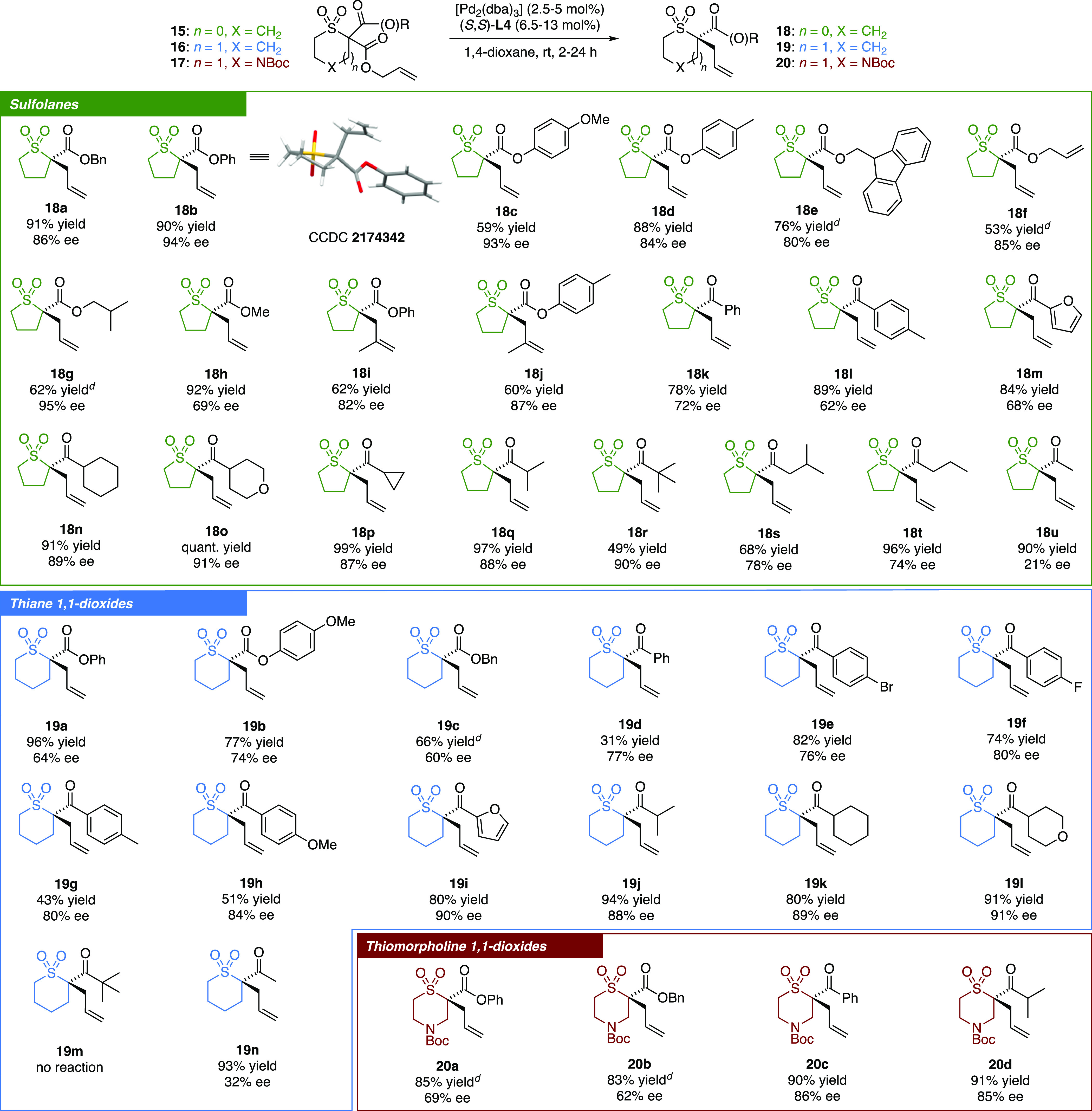
Substrate Scope Investigation^,^^,^ Reactions performed on a 0.09–0.29 mmol scale with [Pd_2_(dba)_3_] (2.5 mol % for esters and 5 mol % for ketones) and (*S*,*S*)-**L4** (6.5 mol % for esters and 13 mol % for ketones) in 1,4-dioxane (0.1 M). All yields are of the isolated product after purification by chromatography. All ee values were determined by chiral HPLC. Catalyst loading: [Pd_2_(dba)_3_] (5 mol %) and (*S*,*S*)-**L4** (13 mol %).

Using the optimal reaction conditions, a range of ester- and ketone-substituted cyclic sulfones **15**–**17** were tested to investigate the scope of this methodology (Scheme 3). Starting with sulfolanes **15**, phenyl- and *p*-methoxyphenyl esters **18b** and **18c** were isolated with high ee. In addition, an X-ray crystal structure of **18b** confirmed the absolute stereochemical configuration of the newly formed tetrasubstituted center,^26^ which is also in agreement with the stereochemical outcome of allylic alkylation of thietane 1,1-dioxides.^25^ By extension, the sense of stereoinduction was assumed to be the same for the other cyclic sulfone products. Alkyl esters **18c**–**g** were also obtained with high stereoselectivity, albeit the ee of the smaller methyl ester **18h** was lower (69% ee). High selectivity was also maintained in the formation of esters **18i** and **18j** that are functionalized with a substituted allyl group. Surprisingly, ketone substrates **15k**–**u** were found to be much less reactive than esters, necessitating a higher catalyst loading (5 mol % [Pd_2_(dba)_3_] and 13 mol % **L4**), where the higher stability of ketone enolates may potentially result in lower nucleophilicity. Although the ee values of aryl ketone products **18k**–**m** were lower, high enantioselectivity was observed in the formation of products bearing larger alkyl ketone substituents, including secondary alkyl ketones **18n**–**q** and *tert*-butyl **18r**. With decreasing steric hindrance, the selectivity was moderate for primary alkyl ketones **18s** and **18t**, and very low for small methyl ketone **18u**. When the same reaction conditions were applied to thiane 1,1-dioxide substrates **16**, ester-substituted products **19a**–**c** were formed with moderate selectivity. However, the allylic alkylation of thiane 1,1-dioxides **16** bearing a ketone side chain was more selective, giving phenyl ketone **19d**, *p*-substituted ketones **19e**–**h**, and heteroaryl ketone product **19i** in 76–90% ee. Secondary alkyl ketones **19j**–**l** were also formed with high enantioselectivity. *tert*-Butyl ketone substrate **16m** failed to give **19m** due to steric bulk, whereas the much smaller methyl ketone in **19n** gave a low ee. Finally, thiomorpholine 1,1-dioxide precursors **17** were found to be even less reactive than sulfolanes **15** and thiane 1,1-dioxides **16**, requiring a higher catalyst loading even for ester substrates. The selectivity trend was similar to that of thiane 1,1-dioxide products **19**: the ee values of esters **20a** and **20b** were moderate, whereas aryl and alkyl ketone products **20c** and **20d** were formed with much improved selectivity.

**Scheme 4:**
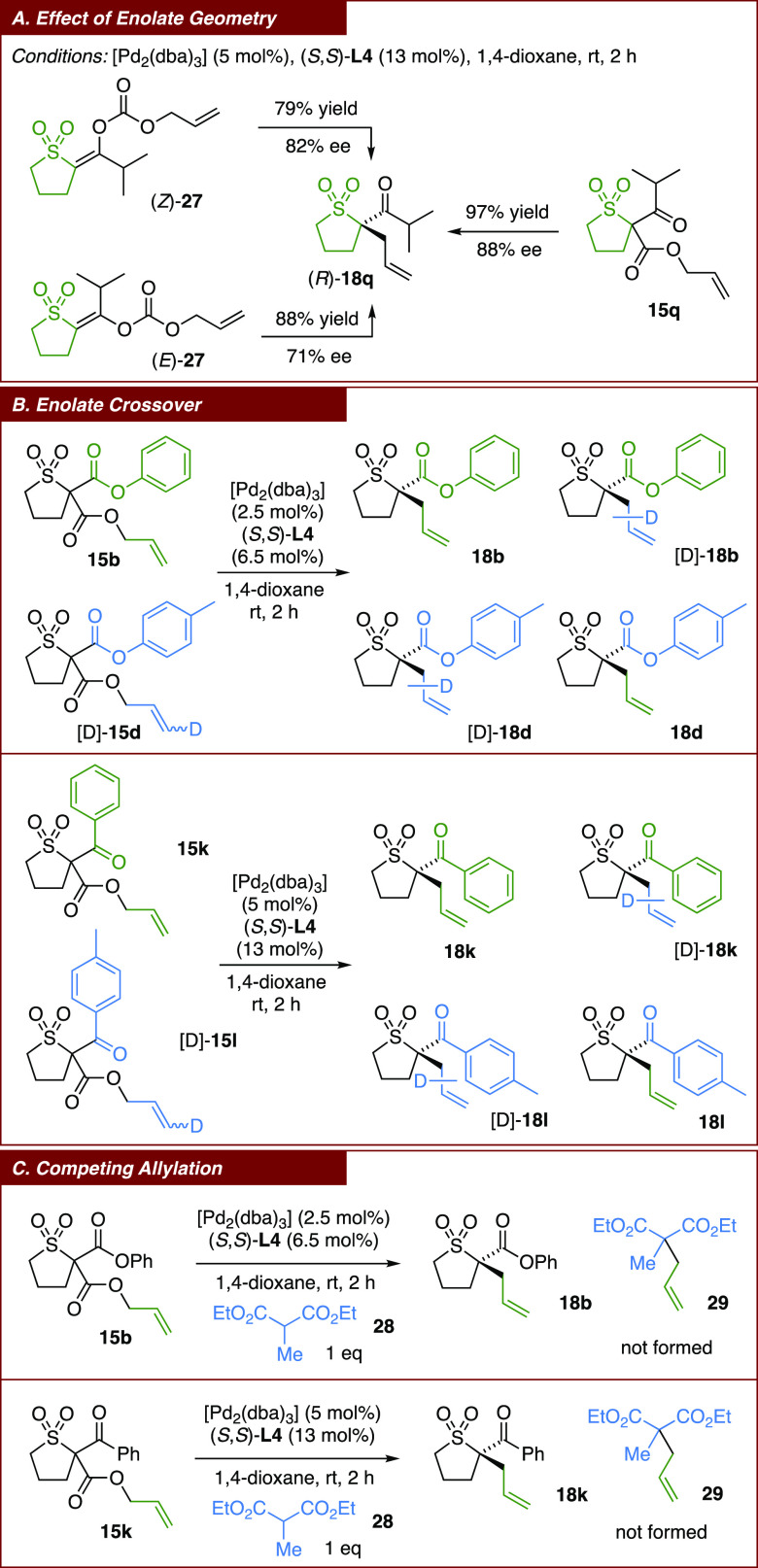
Mechanistic Study

Having observed enantioselective product formation despite the implication of exocyclic enolate intermediates in this DAAA reaction, the impact of the enolate geometry on both the magnitude and the sense of enantioinduction was studied (A, Scheme 4). Geometrically pure allyl enol carbonates (*Z*)-**27** and (*E*)-**27**, each of which should give rise to a geometrically pure enolate intermediate immediately after decarboxylation, were subjected to the catalytic reaction conditions. **18q** was isolated in 82% ee from (*Z*)-**27** and 71% ee from (*E*)-**27**, comprising the *R* stereochemical configuration of the major enantiomer in both cases. By comparison, β-ketoester **15q** also afforded (*R*)-**18q** as the major enantiomer in 88% ee. Given that the sense of stereoinduction is the same in all three cases, it is likely that the selectivity in the formation of (*R*)-**18q** arises from the selective alkylation of one of the two possible enolates in a dynamic kinetic resolution. For this to be the case, a fast interconversion of enolate intermediates needs to take place. As β-ketoester **15q** afforded (*R*)-**18q** with an ee (88%) that is closer in magnitude to the ee of (*R*)-**18q** derived from (*Z*)-**27** (82%) than the ee of (*R*)-**18q** derived from (*E*)-**27** (71%), it is likely that the rate of alkylation of the *Z*-enolate is faster than that of the *E*-enolate. As such, the enantioselectivity of allylation is presumably determined both by the rate of enolate isomerization and the steric effects of the enolate substituent in the transition state structure. We then tested how closely the enolate nucleophile and the π-allylpalladium(II) electrophile are associated during the course of the reaction (B, Scheme 4). Using a mixture of ester **15b** and deuterium-labeled [D]-**15d** in addition to the expected products **18b** and [D]-**18d**, the formation of crossover compounds [D]-**18b** and **18d** was also observed. The result of the reaction of ketone precursors **15k** and [D]-**15l** was analogous: a mixture of all four products **18k**, [D]-**18l**, [D]-**18k**, and **18l** was isolated. In light of full crossover, the nucleophile–electrophile ion pair can readily separate at some stage of the mechanism. Finally, to ascertain the implication of a free enolate intermediate, competitive allylation between β-estersulfolane **15b** and malonate **28** was probed (C, Scheme 4). Formation of an enolate of **15b** by means of decarboxylation in the presence of a malonate should result not only in the expected allylated product **18b** but also in the deprotonation and allylation of malonate **28** at least to some extent provided that enolate exchange is fast compared to allylation.^19a^^1^H NMR spectroscopy indicated that full conversion of **15b** to **18b** took place, whereas allylated **29** was not detected and unreacted malonate **28** was recovered. No allylation of malonate **28** was observed in the presence of β-ketosulfolane substrate **15k** either. Such a scenario would arise if a free enolate is not a long-lived intermediate in the reaction due to either a very fast allylic alkylation immediately after decarboxylation or a tight association of the enolate with the allylpalladium(II) electrophile. Given the implication of a palladium-mediated *E*/*Z* enolate interconversion prior to alkylation, the latter argument seems more likely. The tightly bound nature of the palladium enolate suggests that crossover must occur prior to decarboxylation.

**Scheme 5:**
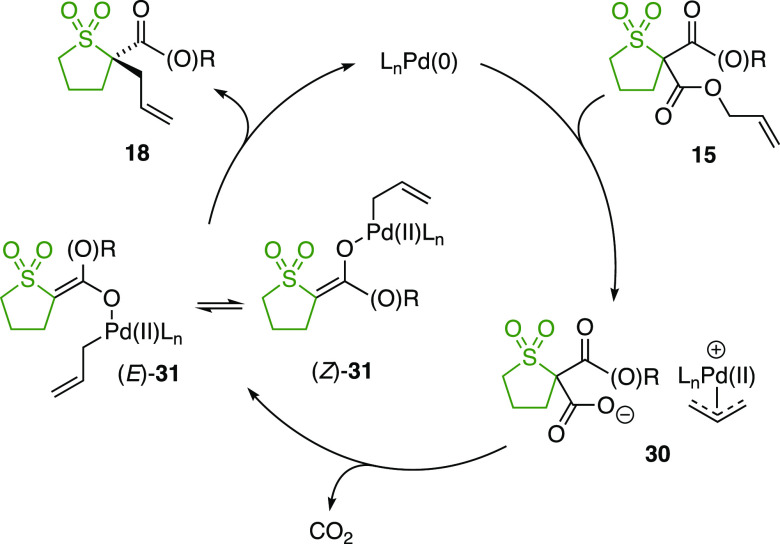
Proposed Catalytic Cycle

The proposed mechanism of the reaction begins with oxidative addition of the palladium(0) catalyst to allyl ester **15** (Scheme 5). The resulting intermediate **30** is likely to exist as a loosely bound ion pair between a carboxylate and a π-allylpalladium(II) complex that can readily undergo crossover. Subsequent decarboxylation gives rise to a mixture of *E*- and *Z*-enolates **31**, which are tightly associated with the σ-allylpalladium(II) complex. A fast isomerization of (*E*)-**31** and (*Z*)-**31** then takes place, presumably *via* a carbon-bound palladium enolate tautomer, and preferential allylic alkylation of (*Z*)-**31** over (*E*)-**31** gives rise to enantioenriched product **18**.

## Conclusions

In conclusion, we have developed a palladium-catalyzed decarboxylative asymmetric allylic alkylation reaction of 5- and 6-membered sulfones that paves the way for enantioenriched α-difunctionalized sulfolanes, thiane 1,1-dioxides, and thiomorpholine 1,1-dioxides. The success of this approach in achieving high levels of enantioselectivity relies on the dynamic kinetic resolution of *E*- and *Z*-enolate intermediates. This method, therefore, offers clear advantages in terms of operational simplicity in that readily accessible racemic allyl ester starting materials can be used without the requirement for the stereoselective synthesis of geometrically pure allyl enol carbonate substrates. What remains to be explored is whether the palladium-mediated dynamic kinetic resolution of acyclic enolates is more generally applicable in the stereoselective allylation of other heterocyclic and acyclic building blocks. This work is ongoing in our laboratory.

## Experimental Section

### General Information

Oven-dried glassware was used for all reactions under an argon atmosphere. Dry solvents were obtained from commercial sources or an Innovative Technologies PureSolv solvent drying system. All reagents and solvents were used as supplied. Ligands **L1**–**4** were obtained from commercial sources. Petrol refers to the fraction of petroleum that boils between 40 and 60 °C. Aqueous solutions were saturated unless stated otherwise. Silica gel (40–63 μm particle size) was used for flash column chromatography. Thin-layer chromatography (TLC) was carried out using silica gel 60 F254 aluminum-backed plates. Ultraviolet irradiation (254 nm) and staining with potassium permanganate or acidic ammonium molybdate(VI) solutions as appropriate were used to visualize TLC plates. ^1^H NMR spectra were obtained using either a Bruker AVANCE III 400 MHz spectrometer or a Bruker FOURIER 300 MHz spectrometer, in CDCl_3_ or DMSO-*d*_6_. ^13^C NMR spectra were recorded on the same spectrometers at 100 or 75 MHz, respectively. For ^1^H NMR spectra in CDCl_3_ or DMSO-*d*_6_, the residual protic solvent CHCl_3_ (δ_H_ = 7.26 ppm) or the central resonance of the residual protic solvent DMSO-*d*_5_ (δ_H_ = 2.50 ppm), respectively, was used as the internal reference. For ^13^C NMR spectra in CDCl_3_ or DMSO-*d*_6_, the central resonance of CDCl_3_ (δ_C_ = 77.0 ppm) or DMSO-*d*_6_ (δ_C_ = 39.5 ppm), respectively, was used as the internal reference. Where rotamers were present, NMR data were recorded in DMSO-*d*_6_ at 130 °C. NMR data are reported as follows: chemical shift, δ_H_ (in parts per million, ppm), (multiplicity, coupling constant, *J* in Hertz, and number of protons). Couplings are expressed as one, or a combination of the following: s, singlet; d, doublet; t, triplet; q, quartet; quint, quintet; sext, sextet; hept, heptet; and m, multiplet. When coincidental coupling constants were observed in the NMR spectra, the apparent multiplicity of the proton resonance in these cases was reported. 1D nuclear Overhauser effect spectroscopy was used to determine the alkene geometry in (*Z*)-**27** and (*E*)-**27**. High-resolution mass spectra (HRMS) were recorded using a Shimadzu LCMS-IT-TOF instrument using ESI or APCI conditions. Infrared spectra were recorded on an Agilent Technologies Cary 630 FTIR spectrometer. Melting points were measured on a Sanyo Gallenkamp capillary melting point apparatus. Enantiomeric excesses were determined by chiral HPLC on a Shimadzu NEXERA X2 UHPLC instrument equipped with a UV detector, using either a Chiralcel OD-H or Chiralpak AD-H column. Optical rotations were measured in CHCl_3_ using an AA-65 Automatic Polarimeter.

### Synthesis of Sulfones **21**–**23**

Sulfolane (**21**) was obtained from commercial sources. Thiane 1,1-dioxide (**22**)^27^ and *N*-Boc thiomorpholine 1,1-dioxide (**23**)^28^ were prepared according to literature procedures.

### Synthesis of Compounds **24**–**26**

#### Allyl-tetrahydrothiophene-2-carboxylate-1,1-dioxide (**24**)

A solution of LiHMDS (1 M in THF, 200 mL, 200 mmol) in THF (500 mL) was cooled to −78 °C. A solution of sulfolane (**21**, 12.06 g, 90 mmol) in THF (10 mL) was added dropwise. The mixture was stirred at −78 °C for 1 h. Allyl chloroformate (11.7 mL, 110 mmol) was added dropwise. The mixture was allowed to warm up to room temperature and was stirred for 15 h. The mixture was quenched with aq. HCl (1 N, 500 mL), and the mixture was extracted with EtOAc (3 × 500 mL). The combined organic phase was washed with water (3 × 1 L), brine (1 L), dried (MgSO_4_), and concentrated under reduced pressure. Purification by flash column chromatography [hexane:EtOAc 2:1] gave **24** (13.7 g, 67%) as a yellow oil. *R*_f_ = 0.21 [petrol:EtOAc 2:1]. ^1^H NMR (400 MHz, CDCl_3_): δ 5.92 (ddt, *J* = 17.2, 10.5, 5.9 Hz, 1H), 5.37 (dq, *J* = 17.2, 1.4 Hz, 1H), 5.27 (dq, *J* = 10.4, 1.2 Hz, 1H), 4.71 (dq, *J* = 6.0, 1.5 Hz, 2H), 3.95 (t, *J* = 7.6 Hz, 1H), 3.18–3.04 (m, 2H), 2.59–2.48 (m, 1H), 2.44–2.29 (m, 2H), 2.22–2.08 (m, 1H) ppm. ^13^C{^1^H} NMR (100 MHz, CDCl_3_): δ 165.2, 131.1, 119.2, 67.0, 64.6, 51.5, 25.9, 20.3 ppm. IR: ν_max_ (neat) 2969, 1737 cm^–1^. HRMS (ESI) *m/z*: calcd for C_8_H_11_O_4_S [M – H]^−^ 203.0384, found 203.0381.

#### Allyl-1,1-dioxo-thiane-2-carboxylate (**25**)

A solution of LiHMDS (1 M in THF, 180 mL, 180 mmol) in THF (450 mL) was cooled to −78 °C. A solution of thiane 1,1-dioxide (**22**, 9.52 mL, 100 mmol) in THF (10 mL) was added dropwise. The mixture was stirred at −78 °C for 1 h. Allyl chloroformate (11.7 mL, 110 mmol) was added dropwise. The mixture was allowed to warm up to room temperature and was stirred for 15 h. The mixture was quenched with aq. HCl (1 N, 500 mL), and the mixture was extracted with EtOAc (3 × 500 mL). The combined organic phase was washed with brine (1 L), dried (MgSO_4_), and concentrated under reduced pressure. Purification by flash column chromatography [hexane:EtOAc 3:1–2:1] gave **25** (17.4 g, 89%) as a yellow oil. *R*_f_ = 0.17 [petrol:EtOAc 4:1]. ^1^H NMR (300 MHz, CDCl_3_): δ 5.92 (ddt, *J* = 17.2, 10.4, 5.8 Hz, 1H), 5.38 (dq, *J* = 17.2, 1.4 Hz, 1H), 5.29 (dq, *J* = 10.4, 1.3 Hz, 1H), 4.71 (d, *J* = 5.0 Hz, 2H), 3.88 (ddd, *J* = 6.4, 4.7, 2.0 Hz, 1H), 3.43 (ddd, *J* = 13.6, 8.1, 5.2 Hz, 1H), 3.05–2.94 (m, 1H), 2.40–2.23 (m, 2H), 2.18–2.06 (m, 2H), 1.91 (dtt, *J* = 17.3, 8.9, 4.2 Hz, 1H), 1.67–1.53 (m, 1H) ppm. ^13^C{^1^H} NMR (75 MHz, CDCl_3_): δ 165.5, 130.9, 119.4, 66.7, 64.9, 50.9, 27.9, 24.0, 20.7 ppm. IR: ν_max_ (neat) 2939, 2870, 1731 cm^–1^. HRMS (ESI) *m/z*: calcd for C_9_H_15_O_4_S [M + H]^+^ 219.0686, found 219.0677.

#### 2-Allyl-4-*tert*-butyl-1,1-dioxo-1,4-thiazinane-2,4-dicarboxylate (**26**)

A solution of *N*-Boc thiomorpholine 1,1-dioxide (**23**, 11.75 g, 50 mmol) in THF (200 mL) was cooled to −78 °C. A solution of LiHMDS (1 M in THF, 100 mL, 100 mmol) in THF (50 mL) was added dropwise. The reaction mixture was stirred at −78 °C for 1 h. Allyl chloroformate (5.85 mL, 55 mmol) was added dropwise. The mixture was allowed to warm to room temperature and stirred for 4 h. The reaction was quenched with aq. HCl (1 N, 300 mL). The mixture was extracted with EtOAc (3 × 300 mL), washed with brine (500 mL), dried (MgSO_4_), and concentrated under reduced pressure. Purification by flash column chromatography [9:1–4:1 petrol:EtOAc] afforded **26** (14.32 g, 90%) as a colorless solid. *R*_f_ = 0.22 [2:1 petrol:EtOAc]. m.p.: 78–80 °C. ^1^H NMR (400 MHz, DMSO-*d*_6_, 130 °C): δ 5.94 (ddt, *J* = 17.3, 10.8, 5.5 Hz, 1H), 5.40 (dq, *J* = 17.3, 1.6 Hz, 1H), 5.27 (dq, *J* = 10.6, 1.4 Hz, 1H), 4.70 (dt, *J* = 5.4, 1.5 Hz, 2H), 4.21 (ddd, *J* = 5.6, 3.7, 1.8 Hz, 1H), 4.09 (ddd, *J* = 14.7, 6.2, 1.4 Hz, 1H), 4.01–3.91 (m, 2H), 3.75 (dddd, *J* = 14.6, 8.4, 3.3, 0.9 Hz, 1H), 3.37 (ddd, *J* = 14.0, 8.4, 3.6 Hz, 1H), 3.20 (dddd, *J* = 14.0, 6.9, 3.3, 1.8 Hz, 1H), 1.45 (s, 9H) ppm. ^13^C{^1^H} NMR (100 MHz, DMSO-*d*_6_, 130 °C): δ 163.1, 152.5, 130.9, 117.6, 79.9, 65.3, 63.5, 49.8, 44.4, 41.6, 27.3 ppm. IR: ν_max_ (neat) 2985, 2940, 1736, 1701 cm^–1^. HRMS (APCI) *m/z*: calcd for C_13_H_20_O_6_S [M – H]^−^ 318.1017, found 318.1009.

### Synthesis of Allylic Alkylation Precursors **15**–**17**

#### 2-Allyl-2-benzyldihydrothiophene-2,2(3*H*)-dicarboxylate-1,1-dioxide (**15a**)

**24** (1.00 g, 4.90 mmol) was dissolved in THF (50 mL). NaHMDS (1 M in THF, 5.39 mL, 5.39 mmol) was added dropwise. The solution was stirred at room temperature for 30 min. Benzyl chloroformate (0.77 mL, 5.39 mmol) was added dropwise, and the solution was stirred for 15 h. The reaction was quenched with aq. HCl (1 N, 50 mL). The mixture was extracted with EtOAc (3 × 50 mL), washed with brine (100 mL), dried (MgSO_4_), and concentrated under reduced pressure. Purification by flash column chromatography [hexane:EtOAc 19:1–4:1] gave **15a** (1.16 g, 70%) as a colorless oil. *R*_f_ = 0.28 [petrol:EtOAc 4:1]. ^1^H NMR (400 MHz, CDCl_3_): δ 7.39–7.32 (m, 5H), 5.80 (ddt, *J* = 17.2, 10.4, 5.7 Hz, 1H), 5.37–5.28 (m, 3H), 5.22 (dq, *J* = 10.4, 1.2 Hz, 1H), 4.70 (dt, *J* = 5.8, 1.4 Hz, 2H), 3.33 (t, *J* = 6.4 Hz, 2H), 2.77–2.71 (m, 2H), 2.30–2.20 (m, 2H) ppm. ^13^C{^1^H} NMR (100 MHz, CDCl_3_): δ 164.2, 164.0, 134.4, 130.5, 128.6, 128.3, 119.5, 75.0, 68.7, 67.6, 50.3, 30.0, 17.0 ppm. IR: ν_max_ (neat) 3034, 3017, 2961, 1754, 1724 cm^–1^. HRMS (ESI) *m/z*: calcd for C_16_H_18_NaO_6_S [M + Na]^+^ 361.0716, found 361.0701.

#### 2-Allyl-2-phenyldihydrothiophene-2,2(3*H*)-dicarboxylate 1,1-dioxide (**15b**)

**24** (50 mg, 0.25 mmol) was dissolved in THF (2 mL). NaHMDS (1 M in THF, 0.28 mL, 0.28 mmol) was added dropwise. The solution was stirred at room temperature for 30 min. Phenyl chloroformate (35 μL, 0.28 mmol) was added dropwise, and the mixture was stirred for 15 h. The reaction was quenched with aq. HCl (1 N, 10 mL). The mixture was extracted with EtOAc (3 × 10 mL), washed with brine (20 mL), dried (MgSO_4_), and concentrated under reduced pressure. Purification by flash column chromatography [hexane:EtOAc 9:1–4:1] gave **15b** (63 mg, 79%) as a colorless solid. *R*_f_ = 0.19 [petrol:EtOAc 4:1]. m.p.: 58–60 °C. ^1^H NMR (300 MHz, CDCl_3_): δ 7.44–7.36 (m, 2H), 7.31–7.24 (m, 1H), 7.18 (dd, *J* = 8.6, 1.3 Hz, 2H), 5.97 (ddt, *J* = 17.2, 10.4, 5.8 Hz, 1H), 5.45 (dq, *J* = 17.2, 1.5 Hz, 1H), 5.33 (dq, *J* = 10.5, 1.2 Hz, 1H), 4.84 (dq, *J* = 5.8, 1.3 Hz, 2H), 3.51–3.32 (m, 2H), 2.99–2.86 (m, 1H), 2.79 (quint, *J* = 7.5 Hz, 1H), 2.41–2.27 (m, 2H) ppm. ^13^C{^1^H} NMR (75 MHz, CDCl_3_): δ 164.2, 162.9, 150.3, 130.5, 129.6, 126.7, 121.2, 119.9, 75.0, 67.8, 50.6, 30.2, 17.3 ppm. IR: ν_max_ (neat) 3017, 2967, 1765, 1735 cm^–1^. HRMS (ESI) *m/z*: calcd for C_15_H_16_NaO_6_S [M + Na]^+^ 347.0560, found 347.0553.

#### 2-Allyl-2-(4-methoxyphenyl)dihydrothiophene-2,2(3*H*)-dicarboxylate-1,1-dioxide (**15c**)

**24** (306 mg, 1.5 mmol) was dissolved in THF (10 mL). NaHMDS (1 M in THF, 1.65 mL, 1.65 mmol) was added dropwise. The solution was stirred at room temperature for 30 min. 4-Methoxyphenyl chloroformate (0.245 mL, 1.65 mmol) was added dropwise, and the mixture was stirred for 15 h. The reaction was quenched with aq. HCl (1 N, 10 mL). The mixture was extracted with EtOAc (3 × 20 mL), washed with brine (50 mL), dried (MgSO_4_), and concentrated under reduced pressure. Purification by flash column chromatography [petrol:EtOAc 9:1–4:1] gave an inseparable mixture of **24**:**15c** in a 1:2.3 ratio (500 mg, corresponding to 400 mg of pure **15c**, 75%) as a clear oil. *R*_f_ = 0.11 [petrol:EtOAc 4:1]. ^1^H NMR (400 MHz, CDCl_3_, resonances due to **15c** quoted): δ 7.09 (d, *J* = 9.0 Hz, 2H), 6.89 (d, *J* = 9.2 Hz, 2H), 6.02–5.89 (m, 1H), 5.48–5.39 (m, 1H), 5.35–5.29 (m, 1H), 4.82 (dq, *J* = 5.9, 1.5 Hz, 2H), 3.82–3.76 (m, 3H), 3.47–3.32 (m, 2H), 2.90 (dt, *J* = 14.0, 7.2 Hz, 1H), 2.78 (dt, *J* = 14.5, 7.3 Hz, 1H), 2.32 (quint, *J* = 8.0 Hz, 2H) ppm. ^13^C{^1^H} NMR (100 MHz, CDCl_3_, resonances due to **15c** quoted): δ 164.2, 163.3, 157.8, 143.8, 130.5, 122.0, 119.8, 114.5, 75.0, 67.7, 55.6, 50.5, 30.2, 17.3 ppm. IR: ν_max_ (neat) 2974, 3014, 1735 cm^–1^. HRMS (ESI) *m/z*: calcd for C_16_H_18_NaO_7_S [M + Na]^+^ 377.0665, found 377.0650.

#### 2-Allyl-2-(*p*-tolyl)dihydrothiophene-2,2(3*H*)-dicarboxylate-1,1-dioxide (**15d**)

**24** (306 mg, 1.5 mmol) was dissolved in THF (10 mL). NaHMDS (1 M in THF, 1.65 mL, 1.65 mmol) was added dropwise. The solution was stirred at room temperature for 30 min. *p*-Tolyl chloroformate (0.150 mL, 1.65 mmol) was added dropwise, and the mixture was stirred for 15 h. The reaction was quenched with aq. HCl (1 N, 10 mL). The mixture was extracted with EtOAc (3 × 20 mL), washed with brine (50 mL), dried (MgSO_4_), and concentrated under reduced pressure. Purification by flash column chromatography [petrol:EtOAc 4:1] gave **15d** (153 mg, 30%) as a pale yellow solid. *R*_f_ = 0.17 [petrol:EtOAc 4:1]. m.p.: 58–59 °C. ^1^H NMR (400 MHz, CDCl_3_): δ 7.18 (d, *J* = 8.7 Hz, 2H), 7.04 (d, *J* = 8.6 Hz, 2H), 5.95 (ddt, *J* = 16.8, 11.1, 5.7 Hz, 1H), 5.44 (dd, *J* = 17.2, 1.7 Hz, 1H), 5.31 (dt, *J* = 10.5, 1.1 Hz, 1H), 4.82 (d, *J* = 5.9 Hz, 2H), 3.45–3.31 (m, 2H), 2.89 (dt, *J* = 14.2, 7.2 Hz, 1H), 2.77 (dtd, *J* = 14.7, 7.4, 1.2 Hz, 1H), 2.34 (s, 3H), 2.33–2.24 (m, 2H) ppm. ^13^C{^1^H} NMR (100 MHz, CDCl_3_): δ 164.1, 163.0, 148.0, 136.3, 130.4, 129.9, 120.7, 119.6, 74.9, 67.6, 50.5, 30.1, 20.8, 17.2 ppm. IR: ν_max_ (neat) 2976, 3010, 1735 cm^–1^. HRMS (ESI) *m/z*: calcd for C_16_H_18_NaO_6_S [M + Na]^+^ 361.0716, found 361.0713.

#### 2-((9*H*-Fluoren-9-yl)methyl)-2-allyldihydrothiophene-2,2(3*H*)-dicarboxylate-1,1-dioxide (**15e**)

**24** (306 mg, 1.5 mmol) was dissolved in THF (6 mL). LiHMDS (1 M in THF, 1.65 mL, 1.65 mmol) was added dropwise. The solution was stirred at room temperature for 30 min. A solution of Fmoc chloride (427 mg, 1.65 mmol) in THF (4 mL) was added dropwise, and the mixture was stirred for 15 h. The reaction was quenched with aq. HCl (1 N, 10 mL). The mixture was extracted with EtOAc (3 × 20 mL), washed with brine (50 mL), dried (MgSO_4_), and concentrated under reduced pressure. Purification by flash column chromatography [petrol:EtOAc 9:1–4:1] gave **15e** (377 mg, 59%) as a clear oil. N.B. The analogous reaction of **24** with NaHMDS as the base gave **15e** in only 9% yield. *R*_f_ = 0.36 [petrol:EtOAc 2:1]. ^1^H NMR (400 MHz, CDCl_3_): δ 7.76 (dt, *J* = 7.6, 1.0 Hz, 2H), 7.64 (d, *J* = 7.4 Hz, 2H), 7.41 (tdd, *J* = 7.6, 1.6, 1.2 Hz, 2H), 7.33 (qd, *J* = 7.3, 1.0 Hz, 2H), 5.87 (ddt, *J* = 17.2, 10.4, 5.8 Hz, 1H), 5.37 (dq, *J* = 17.2, 1.5 Hz, 1H), 5.24 (dq, *J* = 10.5, 1.2 Hz, 1H), 4.78 (dd, *J* = 10.8, 5.8 Hz, 1H), 4.75–4.71 (m, 2H), 4.53 (dd, *J* = 10.8, 6.7 Hz, 1H), 4.27 (t, *J* = 6.2 Hz, 1H), 3.21 (ddd, *J* = 13.2, 8.8, 5.9 Hz, 1H), 3.08 (ddd, *J* = 13.2, 9.0, 6.5 Hz, 1H), 2.68 (ddd, *J* = 14.1, 8.8, 6.5 Hz, 1H), 2.44 (ddd, *J* = 14.5, 8.7, 5.9 Hz, 1H), 2.15 (dtdd, *J* = 13.4, 8.9, 6.7, 6.0 Hz, 1H), 2.08–1.96 (m, 1H) ppm. ^13^C{^1^H} NMR (100 MHz, CDCl_3_): δ 164.5, 163.8, 143.4, 142.9, 141.3, 141.3, 130.6, 128.0, 128.0, 127.4, 127.3, 125.1, 125.0, 120.0, 119.9, 119.6, 75.0, 68.5, 67.6, 50.1, 46.6, 29.8, 16.8 ppm. IR: ν_max_ (neat) 2945, 1731 cm^–1^. HRMS (ESI) *m/z*: calcd for C_23_H_22_NaO_6_S [M + Na]^+^ 449.1029, found 449.1009.

#### Diallyl-1,1-dioxo-thiolane-2,2-dicarboxylate (**15f**)

**24** (408 mg, 2.0 mmol) was dissolved in THF (15 mL). NaHMDS (1 M in THF, 2.20 mL, 2.2 mmol) was added dropwise. The solution was stirred at room temperature for 30 min. Allyl chloroformate (0.234 mL, 2.2 mmol) was added dropwise, and the mixture was stirred for 15 h. The reaction was quenched with aq. HCl (1 N, 50 mL). The mixture was extracted with EtOAc (3 × 50 mL), washed with brine (150 mL), dried (MgSO_4_), and concentrated under reduced pressure. Purification by flash column chromatography [hexane:EtOAc 4:1] gave **15f** (212 mg, 37%) as a yellow oil. *R*_f_ = 0.55 [petrol:EtOAc 1:1]. ^1^H NMR (400 MHz, CDCl_3_): δ 5.85 (ddt, *J* = 17.2, 10.5, 5.7 Hz, 2H), 5.33 (dq, *J* = 17.2, 1.5 Hz, 2H), 5.22 (dq, *J* = 10.5, 1.2 Hz, 2H), 4.70 (dt, *J* = 5.7, 1.4 Hz, 4H), 3.32–3.25 (m, 2H), 2.70–2.63 (m, 2H), 2.25–2.15 (m, 2H) ppm. ^13^C{^1^H} NMR (100 MHz, CDCl_3_): δ 163.7, 130.4, 119.1, 74.8, 67.2, 50.1, 29.8, 16.8 ppm. IR: ν_max_ (neat) 2953, 1731 cm^–1^. HRMS (APCI) *m/z*: calcd for C_12_H_17_O_6_S [M + H]^+^ 289.0740, found 289.0726.

#### 2′-Allyl-2′-isobutyl-1,1-dioxo-thiolane-2,2-dicarboxylate (**15g**)

**24** (408 mg, 2.0 mmol) was dissolved in THF (15 mL). NaHMDS (1 M in THF, 2.20 mL, 2.2 mmol) was added dropwise. The solution was stirred at room temperature for 30 min. Isobutyl chloroformate (0.286 mL, 2.2 mmol) was added dropwise, and the mixture was stirred for 15 h. The reaction was quenched with aq. HCl (1 N, 50 mL). The mixture was extracted with EtOAc (3 × 50 mL), washed with brine (150 mL), dried (MgSO_4_), and concentrated under reduced pressure. Purification by flash column chromatography [hexane:EtOAc 4:1] gave **15g** (415 mg, 68%) as a yellow oil. *R*_f_ = 0.60 [petrol:EtOAc 1:1]. ^1^H NMR (400 MHz, CDCl_3_): δ 5.82 (ddt, *J* = 17.2, 10.5, 5.6 Hz, 1H), 5.30 (dq, *J* = 17.2, 1.5 Hz, 1H), 5.17 (dq, *J* = 10.5, 1.2 Hz, 1H), 4.65 (ddt, *J* = 5.4, 3.8, 1.4 Hz, 2H), 3.97 (dd, *J* = 10.5, 6.5 Hz, 1H), 3.91 (dd, *J* = 10.5, 6.5 Hz, 1H), 3.26–3.19 (m, 2H), 2.65–2.58 (m, 2H), 2.20–2.11 (m, 2H), 1.90 (hept, *J* = 6.8 Hz, 1H), 0.85 (d, *J* = 6.8 Hz, 6H) ppm. ^13^C{^1^H} NMR (100 MHz, CDCl_3_): δ 163.9, 163.7, 130.4, 118.9, 74.7, 72.6, 67.0, 49.9, 29.7, 27.2, 18.5, 18.5, 16.7 ppm. IR: ν_max_ (neat) 2961, 2877, 1731 cm^–1^. HRMS (APCI) *m/z*: calcd for C_13_H_20_O_6_S [M + H]^+^ 305.1053, found 305.1043.

#### 2-Allyl-2-methyldihydrothiophene-2,2(3*H*)-dicarboxylate 1,1-dioxide (**15h**)

**24** (200 mg, 0.98 mmol) was dissolved in THF (15 mL). NaHMDS (1 M in THF, 1.08 mL, 1.08 mmol) was added dropwise. The solution was stirred at room temperature for 30 min. Methyl chloroformate (0.083 mL, 1.08 mmol) was added dropwise, and the mixture was stirred for 15 h. The reaction was quenched with aq. HCl (1 N, 25 mL). The mixture was extracted with EtOAc (3 × 50 mL), washed with brine (150 mL), dried (MgSO_4_), and concentrated under reduced pressure. Purification by flash column chromatography [hexane:EtOAc 4:1] gave **15h** (141 mg, 55%) as a yellow oil. *R*_f_ = 0.18 [petrol:EtOAc 4:1]. ^1^H NMR (300 MHz, CDCl_3_): δ 5.92 (ddt, *J* = 17.1, 10.4, 5.6 Hz, 1H), 5.39 (dq, *J* = 17.2, 1.5 Hz, 1H), 5.29 (dq, *J* = 10.4, 1.3 Hz, 1H), 4.76 (dt, *J* = 5.6, 1.4 Hz, 2H), 3.88 (s, 3H), 3.41–3.25 (m, 2H), 2.73 (td, *J* = 7.2, 1.9 Hz, 2H), 2.26 (quint, *J* = 7.5 Hz, 2H) ppm. ^13^C{^1^H} NMR (75 MHz, CDCl_3_): δ 164.7, 164.1, 130.6, 119.4, 74.9, 67.5, 53.9, 50.2, 30.0, 17.0 ppm. IR: ν_max_ (neat) 2957, 1733 cm^–1^. HRMS (APCI) *m/z*: calcd for C_10_H_15_O_6_S [M + H]^+^ 263.0584, found 263.0577.
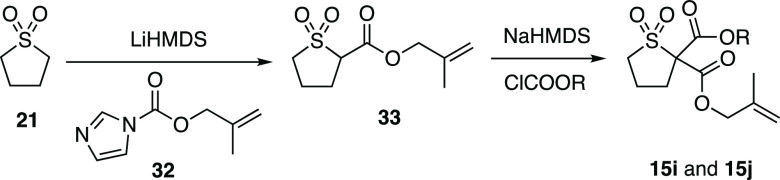


#### (2-Methylallyl)tetrahydrothiophene-2-carboxylate-1,1-dioxide (**33**)

**21** (334 mg, 2.78 mmol) was dissolved in THF (25 mL), and the solution was cooled to −78 °C. LiHMDS (1 M in THF, 5.57 mL, 5.57 mmol) was added dropwise. The mixture was stirred at −78 °C for 1 h. A solution of **32**(19c) (500 mg, 3.00 mmol) in THF (5 mL) was added dropwise. The mixture was allowed to warm up to room temperature and stirred for 15 h. The reaction was quenched with aq. HCl (1 N, 25 mL). The mixture was extracted with EtOAc (3 × 50 mL), washed with brine (150 mL), dried (MgSO_4_), and concentrated under reduced pressure. Purification by flash column chromatography [hexane:EtOAc 2:1] gave **33** (485 mg, 80%) as a colorless oil. *R*_f_ = 0.32 [petrol:EtOAc 2:1]. ^1^H NMR (300 MHz, CDCl_3_): δ 5.04 (hept, *J* = 1.2 Hz, 1H), 4.97 (tt, *J* = 1.6, 0.8 Hz, 1H), 4.67 (d, *J* = 12.8 Hz, 1H), 4.60 (d, *J* = 12.8 Hz, 1H), 3.94 (t, *J* = 7.6 Hz, 1H), 3.21–3.03 (m, 2H), 2.63–2.48 (m, 1H), 2.47–2.28 (m, 2H), 2.26–2.07 (m, 1H), 1.78 (s, 3H) ppm. ^13^C{^1^H} NMR (100 MHz, CDCl_3_): δ 165.3, 139.0, 114.3, 69.8, 64.7, 51.5, 26.0, 20.4, 19.4 ppm. IR: ν_max_ (neat) 3084, 2952, 1735 cm^–1^. HRMS (APCI) *m/z*: calcd for C_9_H_15_O_4_S [M + H]^+^ 219.0686, found 219.0676.

#### 2-(2-Methylallyl)-2-phenyldihydrothiophene-2,2(3*H*)-dicarboxylate-1,1-dioxide (**15i**)

**33** (100 mg, 0.46 mmol) was dissolved in THF (6 mL). NaHMDS (1 M in THF, 0.51 mL, 0.51 mmol) was added dropwise. The solution was stirred at room temperature for 30 min. Phenyl chloroformate (0.064 mL, 0.51 mmol) was added dropwise, and the mixture was stirred for 15 h. The reaction was quenched with aq. HCl (1 N, 5 mL). The mixture was extracted with EtOAc (3 × 10 mL), washed with brine (30 mL), dried (MgSO_4_), and concentrated under reduced pressure. Purification by flash column chromatography [hexane:EtOAc 4:1] gave **15i** (77 mg, 50%) as a colorless oil. *R*_f_ = 0.21 [petrol:EtOAc 4:1]. ^1^H NMR (400 MHz, CDCl_3_): δ 7.43–7.35 (m, 2H), 7.27 (t, *J* = 7.4 Hz, 1H), 7.17 (d, *J* = 7.4 Hz, 2H), 5.10 (quint, *J* = 1.2 Hz, 1H), 5.00 (t, *J* = 1.6 Hz, 1H), 4.77 (d, *J* = 12.7 Hz, 1H), 4.72 (d, *J* = 12.9 Hz, 1H), 3.46–3.32 (m, 2H), 2.91 (dt, *J* = 15.1, 7.8 Hz, 1H), 2.78 (dt, *J* = 14.5, 7.4 Hz, 1H), 2.36–2.26 (m, 2H), 1.80 (s, 3H) ppm. ^13^C{^1^H} NMR (100 MHz, CDCl_3_): δ 164.2, 162.9, 150.2, 138.4, 129.5, 126.6, 121.1, 114.6, 75.0, 70.5, 50.5, 30.2, 19.3, 17.2 ppm. IR: ν_max_ (neat) 3073, 2956, 1735 cm^–1^. HRMS (APCI) *m/z*: calcd for C_16_H_19_O_6_S [M + H]^+^ 339.0897, found 339.0895.

#### 2-(2-Methylallyl)-2-(*p*-tolyl)dihydrothiophene-2,2(3*H*)-dicarboxylate-1,1-dioxide (**15j**)

**33** (240 mg, 1.10 mmol) was dissolved in THF (10 mL). NaHMDS (1 M in THF, 1.35 mL, 1.35 mmol) was added dropwise. The solution was stirred at room temperature for 30 min. *p*-Tolyl chloroformate (0.200 mL, 1.35 mmol) was added dropwise, and the mixture was stirred for 15 h. The reaction was quenched with aq. HCl (1 N, 10 mL). The mixture was extracted with EtOAc (3 × 25 mL), washed with brine (50 mL), dried (MgSO_4_), and concentrated under reduced pressure. Purification by flash column chromatography [hexane:EtOAc 9:1–4:1] gave **15j** (213 mg, 55%) as a yellow solid. *R*_f_ = 0.30 [petrol:EtOAc 4:1]. m.p.: 70–71 °C. ^1^H NMR (400 MHz, CDCl_3_): δ 7.18 (d, *J* = 7.8 Hz, 2H), 7.05 (d, *J* = 8.7 Hz, 2H), 5.10 (hept, *J* = 1.2 Hz, 1H), 5.00 (tq, *J* = 2.4, 1.2 Hz, 1H), 4.77 (dt, *J* = 12.7, 0.8 Hz, 1H), 4.72 (dt, *J* = 12.8, 0.9 Hz, 1H), 3.47–3.32 (m, 2H), 2.96–2.86 (m, 1H), 2.79 (dt, *J* = 14.5, 7.4 Hz, 1H), 2.36–2.28 (m, 5H), 1.80 (s, 3H) ppm. ^13^C{^1^H} NMR (100 MHz, CDCl_3_): δ 164.2, 163.2, 148.1, 138.5, 136.4, 130.0, 120.8, 114.7, 75.0, 70.5, 50.5, 30.2, 20.9, 19.4, 17.2 ppm. IR: ν_max_ (neat) 3017, 2970, 2920, 1765, 1730 cm^–1^. HRMS (APCI) *m/z*: calcd for C_17_H_21_O_6_S [M + H]^+^ 353.1053, found 353.1052.

#### Allyl-2-benzoyltetrahydrothiophene-2-carboxylate-1,1-dioxide (**15k**)

**24** (1.00 g, 4.90 mmol) was dissolved in THF (50 mL). NaHMDS (1 M in THF, 5.39 mL, 5.39 mmol) was added dropwise. The solution was stirred at room temperature for 30 min. Benzoyl chloride (0.63 mL, 5.39 mmol) was added dropwise, and the mixture was heated at 80 °C for 15 h. The mixture was allowed to cool to room temperature and quenched with aq. HCl (1 N, 50 mL). The mixture was extracted with EtOAc (3 × 100 mL), washed with brine (200 mL), dried (MgSO_4_), and concentrated under reduced pressure. Purification by flash column chromatography [hexane:EtOAc 9:1–4:1] gave **15k** (1.13 g, 75%) as a colorless solid. *R*_f_ = 0.18 [petrol:EtOAc 4:1]. m.p.: 81–83 °C. ^1^H NMR (400 MHz, CDCl_3_): δ 7.97–7.91 (m, 2H), 7.58 (tt, *J* = 7.4, 1.2 Hz, 1H), 7.46 (tt, *J* = 7.1, 1.8 Hz, 2H), 5.63 (ddt, *J* = 17.2, 10.4, 5.9 Hz, 1H), 5.16 (dq, *J* = 12.9, 1.3 Hz, 1H), 5.13 (dq, *J* = 6.0, 1.3 Hz, 1H), 4.62 (dt, *J* = 6.0, 1.3 Hz, 2H), 3.49–3.33 (m, 2H), 3.14 (dt, *J* = 14.7, 7.5 Hz, 1H), 2.72 (ddd, *J* = 14.2, 7.5, 6.4 Hz, 1H), 2.40–2.19 (m, 2H) ppm. ^13^C{^1^H} NMR (75 MHz, CDCl_3_): δ 188.2, 166.0, 135.3, 133.8, 130.1, 129.0, 128.6, 120.0, 77.8, 67.3, 51.8, 31.9, 17.6 ppm. IR: ν_max_ (neat) 3066, 2954, 1735, 1685 cm^–1^. HRMS (ESI) *m/z*: calcd for C_15_H_16_NaO_5_S [M + Na]^+^ 331.0611, found 331.0597.

#### Allyl-2-(*p*-toluoyl)tetrahydrothiophene-2-carboxylate-1,1-dioxide (**15l**)

**24** (100 mg, 0.49 mmol) was dissolved in THF (6 mL). NaHMDS (1 M in THF, 0.54 mL, 0.54 mmol) was added dropwise. The solution was stirred at room temperature for 30 min. *p*-Toluoyl chloride (0.071 mL, 0.54 mmol) was added dropwise, and the mixture was heated at 80 °C for 15 h. The mixture was allowed to cool to room temperature and quenched with aq. HCl (1 N, 5 mL). The mixture was extracted with EtOAc (3 × 10 mL), washed with brine (30 mL), dried (MgSO_4_), and concentrated under reduced pressure. Purification by flash column chromatography [hexane:EtOAc 4:1] gave **15l** (79 mg, 50%) as a colorless solid. *R*_f_ = 0.14 [petrol:EtOAc 4:1]. m.p.: 64–66 °C. ^1^H NMR (400 MHz, CDCl_3_): δ 7.83 (d, *J* = 8.4 Hz, 2H), 7.24 (d, *J* = 8.1 Hz, 2H), 5.65 (ddt, *J* = 17.3, 10.4, 5.9 Hz, 1H), 5.20–5.09 (m, 2H), 4.61 (dt, *J* = 5.9, 1.3 Hz, 2H), 3.46–3.28 (m, 2H), 3.11 (dt, *J* = 14.7, 7.5 Hz, 1H), 2.67 (ddd, *J* = 14.2, 7.5, 6.4 Hz, 1H), 2.38 (s, 3H), 2.33–2.16 (m, 2H) ppm. ^13^C{^1^H} NMR (100 MHz, CDCl_3_): δ 187.4, 166.1, 144.8, 132.7, 130.1, 129.3, 129.1, 119.7, 77.6, 67.1, 51.7, 31.8, 21.6, 17.5 ppm. IR: ν_max_ (neat) 3076, 3022, 2993, 1739, 1679 cm^–1^. HRMS (ESI) *m/z*: calcd for C_16_H_19_O_5_S [M + H]^+^ 323.0948, found 323.0944.

#### Allyl-2-(furan-2-carbonyl)-1,1-dioxo-thiolane-2-carboxylate (**15m**)

**24** (408 mg, 2.0 mmol) was dissolved in THF (15 mL). NaHMDS (1 M in THF, 2.20 mL, 2.2 mmol) was added dropwise. The solution was stirred at room temperature for 30 min. 2-Furoyl chloride (0.217 mL, 2.2 mmol) was added dropwise, and the mixture was heated at 80 °C for 15 h. The mixture was allowed to cool to room temperature and quenched with aq. HCl (1 N, 25 mL). The mixture was extracted with EtOAc (3 × 50 mL), washed with brine (150 mL), dried (MgSO_4_), and concentrated under reduced pressure. Purification by flash column chromatography [hexane:EtOAc 6:1–4:1] gave an inseparable mixture of starting material **24** and **15m** in a 1:5.2 ratio (491 mg, corresponding to 434 mg of pure **15m**, 73%) as a yellow oil. *R*_f_ = 0.61 [petrol:EtOAc 1:1]. ^1^H NMR (400 MHz, CDCl_3_, resonances due to **15m** quoted): δ 7.55 (dd, *J* = 1.6, 0.6 Hz, 1H), 7.29 (dd, *J* = 3.7, 0.6 Hz, 1H), 6.49 (dd, *J* = 3.7, 1.7 Hz, 1H), 5.66 (ddt, *J* = 17.3, 10.5, 5.8 Hz, 1H), 5.16–5.07 (m, 2H), 4.58 (tt, *J* = 6.0, 1.3 Hz, 2H), 3.33–3.18 (m, 2H), 2.98 (dt, *J* = 14.2, 7.2 Hz, 1H), 2.52 (dt, *J* = 14.4, 7.3 Hz, 1H), 2.26–2.16 (m, 1H), 2.13–2.04 (m, 1H) ppm. ^13^C{^1^H} NMR (100 MHz, CDCl_3_, resonances due to **15m** quoted): δ 175.5, 165.0, 150.5, 147.2, 130.2, 119.7, 119.2, 112.6, 76.6, 66.8, 51.6, 30.3, 17.3 ppm. IR: ν_max_ (neat) 3136, 2953, 1735, 1671 cm^–1^. HRMS (APCI) *m/z*: calcd for C_13_H_15_O_6_S [M + H]^+^ 299.0584, found 299.0578.

#### Allyl-2-(cyclohexanecarbonyl)-1,1-dioxo-thiolane-2-carboxylate (**15n**)

**24** (408 mg, 2.0 mmol) was dissolved in THF (15 mL). NaHMDS (1 M in THF, 2.20 mL, 2.2 mmol) was added dropwise. The solution was stirred at room temperature for 30 min. Cyclohexanecarbonyl chloride (0.294 mL, 2.2 mmol) was added dropwise, and the mixture was heated at 80 °C for 15 h. The mixture was allowed to cool to room temperature and quenched with aq. HCl (1 N, 25 mL). The mixture was extracted with EtOAc (3 × 50 mL), washed with brine (150 mL), dried (MgSO_4_), and concentrated under reduced pressure. Purification by flash column chromatography [hexane:EtOAc 9:1–4:1] gave **15n** (444 mg, 71%) as a yellow solid. *R*_f_ = 0.71 [petrol:EtOAc 1:1]. m.p.: 54–56 °C. ^1^H NMR (400 MHz, CDCl_3_): δ 5.94 (ddt, *J* = 17.1, 10.4, 6.1 Hz, 1H), 5.42 (dq, *J* = 17.2, 1.4 Hz, 1H), 5.32 (dq, *J* = 10.4, 1.1 Hz, 1H), 4.76 (dt, *J* = 6.0, 1.0 Hz, 2H), 3.30–3.13 (m, 2H), 3.04–2.95 (m, 1H), 2.72 (dt, *J* = 14.7, 7.6 Hz, 1H), 2.57 (dt, *J* = 14.4, 7.4 Hz, 1H), 2.21–2.11 (m, 2H), 2.03–1.92 (m, 1H), 1.80–1.69 (m, 3H), 1.65 (ddd, *J* = 7.6, 3.8, 2.3 Hz, 1H), 1.45–1.33 (m, 1H), 1.31–1.16 (m, 4H) ppm. ^13^C{^1^H} NMR (100 MHz, CDCl_3_): δ 202.0, 164.5, 130.4, 120.5, 80.0, 67.6, 50.7, 49.6, 30.5, 28.9, 28.8, 25.5, 25.1, 16.9 ppm. IR: ν_max_ (neat) 2931, 2855, 1735, 1705 cm^–1^. HRMS (APCI) *m/z*: calcd for C_15_H_23_O_5_S [M + H]^+^ 315.1261, found 315.1255.

#### Allyl-1,1-dioxo-2-(tetrahydropyran-4-carbonyl)thiolane-2-carboxylate (**15o**)

**24** (408 mg, 2.0 mmol) was dissolved in THF (15 mL). NaHMDS (1 M in THF, 2.20 mL, 2.2 mmol) was added dropwise. The solution was stirred at room temperature for 30 min. Tetrahydro-2*H*-pyran-4-carbonyl chloride (327 mg, 2.2 mmol) was added, and the mixture was heated at 80 °C for 15 h. The mixture was allowed to cool to room temperature and quenched with aq. HCl (1 N, 25 mL). The mixture was extracted with EtOAc (3 × 50 mL), washed with brine (150 mL), dried (MgSO_4_), and concentrated under reduced pressure. Purification by flash column chromatography [hexane:EtOAc 6:1–4:1] gave an inseparable mixture of starting material **24** and **15o** in a 1:3.4 ratio (443 mg, corresponding to 372 mg of pure **15o**, 59%) as a colorless solid. *R*_f_ = 0.52 [petrol:EtOAc 1:1]. m.p.: 59–61 °C. ^1^H NMR (400 MHz, CDCl_3_, resonances due to **15o** quoted): δ 5.95 (ddt, *J* = 17.0, 10.4, 6.1 Hz, 1H), 5.43 (dq, *J* = 17.2, 1.4 Hz, 1H), 5.35 (dq, *J* = 10.4, 1.1 Hz, 1H), 4.77 (dt, *J* = 6.1, 1.2 Hz, 2H), 4.02–3.93 (m, 2H), 3.50–3.25 (m, 4H), 3.20 (ddd, *J* = 13.1, 8.4, 6.3 Hz, 1H), 2.84 (ddd, *J* = 14.8, 8.3, 6.9 Hz, 1H), 2.56 (ddd, *J* = 14.3, 8.0, 6.4 Hz, 1H), 2.30–2.12 (m, 2H), 1.93 (dtd, *J* = 13.4, 3.6, 1.6 Hz, 1H), 1.86–1.65 (m, 3H) ppm. ^13^C{^1^H} NMR (100 MHz, CDCl_3_, resonances due to **15o** quoted): δ 199.8, 164.3, 130.1, 120.2, 79.8, 67.3, 66.5, 66.4, 50.8, 46.6, 29.9, 28.6, 28.5, 16.9 ppm. IR: ν_max_ (neat) 2957, 2845, 1735, 1716 cm^–1^. HRMS (APCI) *m/z*: calcd for C_14_H_21_O_6_S [M + H]^+^ 317.1053, found 317.1038.

#### Allyl-2-(cyclopropanecarbonyl)-1,1-dioxo-thiolane-2-carboxylate (**15p**)

**24** (408 mg, 2.0 mmol) was dissolved in THF (15 mL). NaHMDS (1 M in THF, 2.20 mL, 2.2 mmol) was added dropwise. The solution was stirred at room temperature for 30 min. Cyclopropanecarbonyl chloride (0.200 mL, 2.2 mmol) was added dropwise, and the mixture was heated at 80 °C for 15 h. The mixture was allowed to cool to room temperature and quenched with aq. HCl (1 N, 25 mL). The mixture was extracted with EtOAc (3 × 50 mL), washed with brine (150 mL), dried (MgSO_4_), and concentrated under reduced pressure. Purification by flash column chromatography [hexane:EtOAc 4:1] gave **15p** (444 mg, 82%) as a colorless oil. *R*_f_ = 0.59 [petrol:EtOAc 1:1]. ^1^H NMR (400 MHz, CDCl_3_): δ 5.83 (ddt, *J* = 17.1, 10.5, 5.8 Hz, 1H), 5.29 (dq, *J* = 17.2, 1.4 Hz, 1H), 5.19 (dq, *J* = 10.5, 1.2 Hz, 1H), 4.66 (ddt, *J* = 5.6, 2.7, 1.3 Hz, 2H), 3.25 (ddd, *J* = 13.1, 8.9, 7.4 Hz, 1H), 3.12 (ddd, *J* = 14.0, 8.4, 5.6 Hz, 1H), 2.72 (ddd, *J* = 14.6, 8.2, 6.6 Hz, 1H), 2.42 (ddd, *J* = 14.5, 8.1, 6.6 Hz, 1H), 2.36–2.29 (m, 1H), 2.21–2.09 (m, 1H), 2.09–1.97 (m, 1H), 1.11–1.01 (m, 2H), 1.01–0.92 (m, 2H) ppm. ^13^C{^1^H} NMR (100 MHz, CDCl_3_): δ 197.5, 164.6, 130.3, 119.3, 79.5, 67.0, 50.8, 28.2, 20.9, 16.9, 13.5, 13.4 ppm. IR: ν_max_ (neat) 2955, 1735, 1701 cm^–1^. HRMS (APCI) *m/z*: calcd for C_12_H_17_O_5_S [M + H]^+^ 273.0791, found 273.0783.

#### Allyl-2-(2-methylpropanoyl)-1,1-dioxo-thiolane-2-carboxylate (**15q**)

**24** (250 mg, 1.12 mmol) was dissolved in THF (20 mL). NaHMDS (1 M in THF, 1.35 mL, 1.35 mmol) was added dropwise. The solution was stirred at room temperature for 30 min. Isobutyryl chloride (0.140 mL, 1.35 mmol) was added dropwise, and the mixture was heated at 80 °C for 15 h. The mixture was allowed to cool to room temperature and quenched with aq. HCl (1 N, 25 mL). The mixture was extracted with EtOAc (3 × 50 mL), washed with brine (150 mL), dried (MgSO_4_), and concentrated under reduced pressure. Purification by flash column chromatography [hexane:EtOAc 4:1] gave **15q** (184 mg, 60%) as a yellow oil. *R*_f_ = 0.33 [petrol:EtOAc 4:1]. ^1^H NMR (400 MHz, CDCl_3_) δ 5.96 (ddt, *J* = 17.2, 10.4, 6.0 Hz, 1H), 5.43 (dq, *J* = 17.1, 1.4 Hz, 1H), 5.34 (dq, *J* = 10.4, 1.1 Hz, 1H), 4.78 (dt, *J* = 6.0, 1.2 Hz, 2H), 3.37–3.17 (m, 3H), 2.81–2.71 (m, 1H), 2.61 (dt, *J* = 14.4, 7.4 Hz, 1H), 2.24–2.15 (m, 2H), 1.19 (d, *J* = 6.5 Hz, 3H), 1.12 (d, *J* = 6.7 Hz, 3H) ppm. ^13^C{^1^H} NMR (100 MHz, CDCl_3_): δ 203.5, 164.6, 130.4, 120.5, 80.1, 67.7, 50.7, 39.6, 28.9, 20.4, 19.4, 17.0 ppm. IR: ν_max_ (neat) 2978, 2877, 1735, 1718 cm^–1^. HRMS (APCI) *m/z*: calcd for C_12_H_19_O_5_S [M + H]^+^ 275.0948, found 275.0943.

#### Allyl-2-(2,2-dimethylpropanoyl)-1,1-dioxo-thiolane-2-carboxylate (**15r**)

**24** (408 mg, 2.0 mmol) was dissolved in THF (15 mL). NaHMDS (1 M in THF, 2.20 mL, 2.2 mmol) was added dropwise. The solution was stirred at room temperature for 30 min. Pivaloyl chloride (0.271 mL, 2.2 mmol) was added dropwise, and the mixture was heated at 80 °C for 15 h. The mixture was allowed to cool to room temperature and quenched with aq. HCl (1 N, 25 mL). The mixture was extracted with EtOAc (3 × 50 mL), washed with brine (150 mL), dried (MgSO_4_), and concentrated under reduced pressure. Purification by flash column chromatography [hexane:EtOAc 9:1–4:1] gave **15r** (118 mg, 20%) as a yellow oil. *R*_f_ = 0.63 [petrol:EtOAc 1:1]. ^1^H NMR (400 MHz, CDCl_3_) δ 5.89 (ddt, *J* = 16.5, 10.4, 6.1 Hz, 1H), 5.38 (dq, *J* = 17.2, 1.3 Hz, 1H), 5.28 (dd, *J* = 10.4, 1.0 Hz, 1H), 4.75–4.64 (m, 2H), 3.31–3.17 (m, 2H), 2.81 (dt, *J* = 14.8, 7.6 Hz, 1H), 2.50 (ddd, *J* = 14.2, 7.6, 6.4 Hz, 1H), 2.22–2.03 (m, 2H), 1.22 (s, 9H) ppm. ^13^C{^1^H} NMR (100 MHz, CDCl_3_): δ 203.3, 165.3, 130.1, 120.5, 79.0, 67.3, 50.9, 46.1, 31.4, 27.8, 17.0 ppm. IR: ν_max_ (neat) 2963, 1703 cm^–1^. HRMS (APCI) *m/z*: calcd for C_13_H_21_O_5_S [M + H]^+^ 289.1104, found 289.1099.

#### Allyl-2-(3-methylbutanoyl)-1,1-dioxo-thiolane-2-carboxylate (**15s**)

**24** (408 mg, 2.0 mmol) was dissolved in THF (15 mL). NaHMDS (1 M in THF, 2.20 mL, 2.2 mmol) was added dropwise. The solution was stirred at room temperature for 30 min. Isovaleryl chloride (0.268 mL, 2.2 mmol) was added dropwise, and the mixture was heated at 80 °C for 15 h. The mixture was allowed to cool to room temperature and quenched with aq. HCl (1 N, 25 mL). The mixture was extracted with EtOAc (3 × 50 mL), washed with brine (150 mL), dried (MgSO_4_), and concentrated under reduced pressure. Purification by flash column chromatography [hexane:EtOAc 6:1] gave **15s** (384 mg, 67%) as a yellow oil. *R*_f_ = 0.70 [petrol:EtOAc 1:1]. ^1^H NMR (400 MHz, CDCl_3_) δ 5.84 (ddt, *J* = 17.1, 10.4, 5.9 Hz, 1H), 5.32 (dq, *J* = 17.2, 1.4 Hz, 1H), 5.23 (dq, *J* = 10.4, 1.1 Hz, 1H), 4.67 (dt, *J* = 6.0, 1.3 Hz, 2H), 3.24 (ddd, *J* = 13.2, 8.9, 6.8 Hz, 1H), 3.13 (ddd, *J* = 13.2, 8.6, 6.3 Hz, 1H), 2.74–2.55 (m, 3H), 2.45 (ddd, *J* = 14.5, 8.3, 6.2 Hz, 1H), 2.19–2.02 (m, 3H), 0.85 (d, *J* = 6.7 Hz, 3H), 0.82 (d, *J* = 6.7 Hz, 3H) ppm. ^13^C{^1^H} NMR (100 MHz, CDCl_3_): δ 197.0, 164.6, 130.2, 119.9, 79.4, 67.2, 50.5, 49.7, 28.4, 23.7, 22.0, 22.0, 16.7 ppm. IR: ν_max_ (neat) 2959, 2873, 1735, 1718 cm^–1^. HRMS (APCI) *m/z*: calcd for C_13_H_20_NaO_5_S [M + Na]^+^ 311.0924, found 311.0916.

#### Allyl-2-butanoyl-1,1-dioxo-thiolane-2-carboxylate (**15t**)

**24** (408 mg, 2.0 mmol) was dissolved in THF (15 mL). NaHMDS (1 M in THF, 2.20 mL, 2.2 mmol) was added dropwise. The solution was stirred at room temperature for 30 min. Butyryl chloride (0.228 mL, 2.2 mmol) was added dropwise, and the mixture was heated at 80 °C for 15 h. The mixture was allowed to cool to room temperature and quenched with aq. HCl (1 N, 25 mL). The mixture was extracted with EtOAc (3 × 50 mL), washed with brine (150 mL), dried (MgSO_4_), and concentrated under reduced pressure. Purification by flash column chromatography [hexane:EtOAc 6:1] gave **15t** (265 mg, 48%) as a colorless oil. *R*_f_ = 0.68 [petrol:EtOAc 1:1]. ^1^H NMR (400 MHz, CDCl_3_) δ 5.86 (ddt, *J* = 17.1, 10.4, 5.9 Hz, 1H), 5.33 (dq, *J* = 17.2, 1.4 Hz, 1H), 5.24 (dq, *J* = 10.4, 1.1 Hz, 1H), 4.68 (dq, *J* = 6.0, 1.0 Hz, 2H), 3.25 (ddd, *J* = 13.1, 8.8, 7.1 Hz, 1H), 3.14 (ddd, *J* = 13.2, 8.5, 6.1 Hz, 1H), 2.82–2.64 (m, 3H), 2.46 (ddd, *J* = 14.4, 8.2, 6.3 Hz, 1H), 2.22–2.05 (m, 2H), 1.58 (sext d, *J* = 7.6, 1.1 Hz, 2H), 0.85 (t, *J* = 7.4 Hz, 3H) ppm. ^13^C{^1^H} NMR (100 MHz, CDCl_3_): δ 197.7, 164.8, 130.2, 119.9, 79.4, 67.2, 50.7, 43.2, 28.5, 17.0, 16.9, 13.1 ppm. IR: ν_max_ (neat) 2965, 2877, 1718 cm^–1^. HRMS (APCI) *m/z*: calcd for C_12_H_19_O_5_S [M + H]^+^ 275.0948, found 275.0939.

#### Allyl-2-acetyltetrahydrothiophene-2-carboxylate-1,1-dioxide (**15u**)

**24** (200 mg, 0.98 mmol) was dissolved in THF (15 mL). NaHMDS (1 M in THF, 1.08 mL, 1.08 mmol) was added dropwise. The solution was stirred at room temperature for 30 min. Acetyl chloride (0.077 mL, 1.08 mmol) was added dropwise, and the mixture was heated at 80 °C for 15 h. The mixture was allowed to cool to room temperature and quenched with aq. HCl (1 N, 10 mL). The mixture was extracted with EtOAc (3 × 25 mL), washed with brine (50 mL), dried (MgSO_4_), and concentrated under reduced pressure. Purification by flash column chromatography [hexane:EtOAc 9:1–4:1] gave **15u** (121 mg, 50%) as a yellow oil. *R*_f_ = 0.26 [petrol:EtOAc 4:1]. ^1^H NMR (300 MHz, CDCl_3_) δ 5.91 (ddt, *J* = 17.2, 10.4, 5.9 Hz, 1H), 5.38 (dq, *J* = 17.2, 1.4 Hz, 1H), 5.31 (dq, *J* = 10.4, 1.2 Hz, 1H), 4.74 (dq, *J* = 6.0, 1.2 Hz, 2H), 3.39–3.13 (m, 2H), 2.81 (ddd, *J* = 14.4, 8.3, 6.6 Hz, 1H), 2.58–2.45 (m, 4H), 2.33–2.06 (m, 2H) ppm. ^13^C{^1^H} NMR (75 MHz, CDCl_3_): δ 195.3, 164.8, 130.3, 120.1, 79.8, 67.6, 51.1, 29.3, 28.6, 17.3 ppm. IR: ν_max_ (neat) 2956, 1718 cm^–1^. HRMS (APCI) *m/z*: calcd for C_10_H_15_O_5_S [M + H]^+^ 247.0635, found 247.0625.

#### 2-Allyl-2-phenyl-1,1-dioxo-thiane-2,2-dicarboxylate (**16a**)

**25** (300 mg, 1.37 mmol) was dissolved in THF (20 mL). NaHMDS (1 M in THF, 1.51 mL, 1.51 mmol) was added dropwise. The solution was stirred at room temperature for 30 min. Phenyl chloroformate (0.190 mL, 1.51 mmol) was added dropwise, and the mixture was stirred for 15 h. The reaction was quenched with aq. HCl (1 N, 25 mL). The mixture was extracted with EtOAc (3 × 50 mL), washed with brine (150 mL), dried (MgSO_4_), and concentrated under reduced pressure. Purification by flash column chromatography [hexane:EtOAc 4:1] gave **16a** (195 mg, 43%) as a yellow oil. *R*_f_ = 0.22 [petrol:EtOAc 4:1]. ^1^H NMR (400 MHz, CDCl_3_) δ 7.41 (t, *J* = 7.9 Hz, 2H), 7.28 (t, *J* = 7.4 Hz, 1H), 7.14 (d, *J* = 7.8 Hz, 2H), 5.97 (ddt, *J* = 16.4, 10.5, 5.7 Hz, 1H), 5.45 (dq, *J* = 17.2, 1.6 Hz, 1H), 5.33 (dd, *J* = 10.5, 1.3 Hz, 1H), 4.85 (dd, *J* = 5.7, 1.6 Hz, 2H), 3.57 (dt, *J* = 13.7, 6.3 Hz, 1H), 3.50–3.39 (m, 1H), 2.67 (q, *J* = 6.2, 5.7 Hz, 2H), 2.13 (quint, *J* = 6.1 Hz, 2H), 1.78 (quint, *J* = 5.8 Hz, 2H) ppm. ^13^C{^1^H} NMR (100 MHz, CDCl_3_): δ 163.6, 162.7, 150.1, 130.4, 129.6, 126.7, 121.1, 120.0, 76.5, 67.6, 52.0, 32.5, 24.0, 19.9 ppm. IR: ν_max_ (neat) 3017, 2985, 2946, 2872, 1767, 1737 cm^–1^. HRMS (APCI) *m/z*: calcd for C_16_H_19_O_6_S [M + H]^+^ 339.0897, found 339.0884.

#### 2-Allyl-2′-(4-methoxyphenyl)-1,1-dioxo-thiane-2,2-dicarboxylate (**16b**)

**25** (436 mg, 2.0 mmol) was dissolved in THF (15 mL). NaHMDS (1 M in THF, 2.20 mL, 2.2 mmol) was added dropwise. The solution was stirred at room temperature for 30 min. 4-Methoxyphenyl chloroformate (0.327 mL, 2.2 mmol) was added dropwise, and the mixture was stirred for 15 h. The reaction was quenched with aq. HCl (1 N, 25 mL). The mixture was extracted with EtOAc (3 × 50 mL), washed with brine (150 mL), dried (MgSO_4_), and concentrated under reduced pressure. Purification by flash column chromatography [hexane:EtOAc 3:1] gave an inseparable mixture of starting material **25** and **16b** in a 1:3.9 ratio (569 mg, corresponding to 494 mg of pure **16b**, 67%) as a colorless solid. *R*_f_ = 0.55 [petrol:EtOAc 1:1]. m.p.: 67–69 °C. ^1^H NMR (400 MHz, CDCl_3_) δ 6.94 (d, *J* = 9.1 Hz, 2H), 6.79 (d, *J* = 9.1 Hz, 2H), 5.85 (ddt, *J* = 17.1, 10.6, 5.6 Hz, 1H), 5.33 (dq, *J* = 17.3, 1.4 Hz, 1H), 5.20 (dq, *J* = 10.5, 1.3 Hz, 1H), 4.72 (ddt, *J* = 6.0, 3.2, 1.3 Hz, 2H), 3.65 (s, 3H), 3.42 (dt, *J* = 13.3, 6.3 Hz, 1H), 3.34–3.24 (m, 1H), 2.61–2.44 (m, 2H), 2.03–1.89 (m, 2H), 1.68–1.57 (m, 2H) ppm. ^13^C{^1^H} NMR (100 MHz, CDCl_3_): δ 163.1, 162.6, 157.3, 143.0, 130.2, 121.4, 119.1, 114.0, 76.0, 67.0, 55.1, 51.5, 32.0, 23.5, 19.3 ppm. IR: ν_max_ (neat) 2944, 2838, 1735, 1321, 1129 cm^–1^. HRMS (ESI) *m/z*: calcd for C_17_H_20_NaO_7_S [M + Na]^+^ 391.0822, found 391.0832.

#### 2-Allyl-2-benzyl-1,1-dioxo-thiane-2,2-dicarboxylate (**16c**)

**25** (436 mg, 2.0 mmol) was dissolved in THF (15 mL). NaHMDS (1 M in THF, 2.20 mL, 2.2 mmol) was added dropwise. The solution was stirred at room temperature for 30 min. Benzyl chloroformate (0.314 mL, 2.2 mmol) was added dropwise, and the mixture was stirred for 15 h. The reaction was quenched with aq. HCl (1 N, 25 mL). The mixture was extracted with EtOAc (3 × 50 mL), washed with brine (150 mL), dried (MgSO_4_), and concentrated under reduced pressure. Purification by flash column chromatography [hexane:EtOAc 4:1] gave **16c** (417 mg, 59%) as a colorless oil. *R*_f_ = 0.45 [petrol:EtOAc 1:1]. ^1^H NMR (400 MHz, CDCl_3_) δ 7.38–7.31 (m, 5H), 5.75 (ddt, *J* = 17.2, 10.5, 5.7 Hz, 1H), 5.35–5.26 (m, 3H), 5.20 (dq, *J* = 10.4, 1.2 Hz, 1H), 4.67 (dq, *J* = 5.7, 1.3 Hz, 2H), 3.43 (t, *J* = 6.2 Hz, 2H), 2.58–2.50 (m, 2H), 2.07 (quint, *J* = 6.1 Hz, 2H), 1.70–1.61 (m, 2H) ppm. ^13^C{^1^H} NMR (100 MHz, CDCl_3_): δ 163.8, 163.5, 134.4, 130.4, 128.6, 128.6, 128.2, 119.5, 76.4, 68.5, 67.4, 51.8, 32.4, 23.9, 19.8 ppm. IR: ν_max_ (neat) 2935, 1731 cm^–1^. HRMS (ESI) *m/z*: calcd for C_17_H_20_NaO_6_S [M + Na]^+^ 375.0873, found 375.0868.

#### Allyl-2-benzoyl-1,1-dioxo-thiane-2-carboxylate (**16d**)

**25** (436 mg, 2.0 mmol) was dissolved in THF (15 mL). NaHMDS (1 M in THF, 2.20 mL, 2.2 mmol) was added dropwise. The solution was stirred at room temperature for 30 min. Benzoyl chloride (0.256 mL, 2.2 mmol) was added dropwise, and the mixture was heated at 80 °C for 15 h. The mixture was allowed to cool to room temperature and quenched with aq. HCl (1 N, 25 mL). The mixture was extracted with EtOAc (3 × 50 mL), washed with brine (150 mL), dried (MgSO_4_), and concentrated under reduced pressure. Purification by flash column chromatography [hexane:EtOAc 4:1] gave **16d** (521 mg, 81%) as a colorless oil. *R*_f_ = 0.65 [petrol:EtOAc 1:1]. ^1^H NMR (400 MHz, CDCl_3_) δ 7.89 (dd, *J* = 8.5, 1.2 Hz, 2H), 7.50 (tt, *J* = 7.4, 1.2 Hz, 1H), 7.36 (t, *J* = 7.4 Hz, 2H), 5.53 (ddt, *J* = 17.1, 10.4, 5.9 Hz, 1H), 5.10 (dq, *J* = 17.2, 1.4 Hz, 1H), 5.05 (dq, *J* = 10.4, 1.1 Hz, 1H), 4.55 (dq, *J* = 5.9, 1.1 Hz, 2H), 3.53 (dt, *J* = 13.5, 6.6 Hz, 1H), 3.48–3.37 (m, 1H), 2.80 (ddd, *J* = 15.1, 9.7, 3.2 Hz, 1H), 2.58 (ddd, *J* = 15.1, 7.9, 2.9 Hz, 1H), 2.03 (quint, *J* = 6.2 Hz, 2H), 1.67–1.49 (m, 2H) ppm. ^13^C{^1^H} NMR (100 MHz, CDCl_3_): δ 187.9, 165.5, 134.9, 133.5, 129.7, 129.0, 128.2, 119.6, 80.0, 67.0, 52.4, 32.9, 23.6, 19.3 ppm. IR: ν_max_ (neat) 2937, 2868, 1735, 1679 cm^–1^. HRMS (APCI) *m/z*: calcd for C_16_H_18_NaO_5_S [M + Na]^+^ 345.0767, found 345.0756.

#### Allyl-2-(4-bromobenzoyl)-1,1-dioxo-thiane-2-carboxylate (**16e**)

**25** (436 mg, 2.0 mmol) was dissolved in THF (15 mL). NaHMDS (1 M in THF, 2.20 mL, 2.2 mmol) was added dropwise. The solution was stirred at room temperature for 30 min. 4-Bromobenzoyl chloride (483 mg, 2.2 mmol) was added, and the mixture was heated at 80 °C for 15 h. The mixture was allowed to cool to room temperature and quenched with aq. HCl (1 N, 25 mL). The mixture was extracted with EtOAc (3 × 50 mL), washed with brine (150 mL), dried (MgSO_4_), and concentrated under reduced pressure. Purification by flash column chromatography [hexane:EtOAc 4:1] gave **16e** (615 mg, 77%) as a colorless solid. *R*_f_ = 0.65 [petrol:EtOAc 1:1]. m.p.: 83–85 °C. ^1^H NMR (400 MHz, CDCl_3_) δ 7.79 (d, *J* = 8.8 Hz, 2H), 7.52 (d, *J* = 8.8 Hz, 2H), 5.57 (ddt, *J* = 17.1, 10.4, 6.0 Hz, 1H), 5.17–5.06 (m, 2H), 4.56 (ddt, *J* = 6.0, 2.6, 1.2 Hz, 2H), 3.60 (dt, *J* = 14.4, 7.1 Hz, 1H), 3.35 (dt, *J* = 13.9, 5.0 Hz, 1H), 2.79 (ddd, *J* = 14.5, 11.0, 3.0 Hz, 1H), 2.50 (ddd, *J* = 15.0, 6.9, 2.5 Hz, 1H), 2.08–1.98 (m, 2H), 1.72–1.62 (m, 1H), 1.56–1.43 (m, 1H) ppm. ^13^C{^1^H} NMR (100 MHz, CDCl_3_): δ 186.7, 165.7, 133.9, 131.6, 130.9, 129.7, 129.0, 120.2, 79.9, 67.2, 52.5, 33.0, 23.7, 19.5 ppm. IR: ν_max_ (neat) 3091, 2937, 2808, 1735 cm^–1^. HRMS (APCI) *m/z*: calcd for C_16_H_18_^79^BrO_5_S [M + H]^+^ 401.0053, found 401.0054.

#### Allyl-2-(4-fluorobenzoyl)-1,1-dioxo-thiane-2-carboxylate (**16f**)

**25** (436 mg, 2.0 mmol) was dissolved in THF (15 mL). NaHMDS (1 M in THF, 2.20 mL, 2.2 mmol) was added dropwise. The solution was stirred at room temperature for 30 min. 4-Fluorobenzoyl chloride (0.260 mL, 2.2 mmol) was added dropwise, and the mixture was heated at 80 °C for 15 h. The mixture was allowed to cool to room temperature and quenched with aq. HCl (1 N, 25 mL). The mixture was extracted with EtOAc (3 × 50 mL), washed with brine (150 mL), dried (MgSO_4_), and concentrated under reduced pressure. Purification by flash column chromatography [hexane:EtOAc 4:1] gave **16f** (564 mg, 83%) as a colorless solid. *R*_f_ = 0.61 [petrol:EtOAc 1:1]. m.p.: 88–90 °C. ^1^H NMR (400 MHz, CDCl_3_) δ 7.96 (dd, *J* = 9.0, 5.3 Hz, 2H), 7.04 (t, *J* = 8.7 Hz, 2H), 5.56 (ddt, *J* = 17.4, 10.4, 6.0 Hz, 1H), 5.16–5.06 (m, 2H), 4.56 (dq, *J* = 6.4, 1.5 Hz, 2H), 3.60 (dt, *J* = 14.3, 7.5 Hz, 1H), 3.35 (dt, *J* = 14.2, 5.4 Hz, 1H), 2.80 (ddd, *J* = 14.2, 11.0, 3.1 Hz, 1H), 2.51 (ddd, *J* = 15.1, 7.2, 3.0 Hz, 1H), 2.08–1.99 (m, 2H), 1.73–1.61 (m, 1H), 1.56–1.41 (m, 1H) ppm. ^13^C{^1^H} NMR (100 MHz, CDCl_3_): δ 185.9, 165.7, 165.7 (d, *J* = 256.9 Hz), 132.3 (d, *J* = 9.6 Hz), 131.4 (d, *J* = 3.0 Hz), 129.7, 120.0, 115.4 (d, *J* = 22.0 Hz), 79.8, 67.1, 52.4, 33.0, 23.6, 19.4 ppm. ^19^F{^1^H} NMR (376 MHz, CDCl_3_): δ −104.7 ppm. IR: ν_max_ (neat) 2939, 2868, 1735, 1632 cm^–1^. HRMS (APCI) *m/z*: calcd for C_16_H_18_FO_5_S [M + H]^+^ 341.0853, found 341.0836.

#### Allyl-2-(4-methylbenzoyl)-1,1-dioxo-thiane-2-carboxylate (**16g**)

**25** (436 mg, 2.0 mmol) was dissolved in THF (15 mL). NaHMDS (1 M in THF, 2.20 mL, 2.2 mmol) was added dropwise. The solution was stirred at room temperature for 30 min. *p*-Toluoyl chloride (0.291 mL, 2.2 mmol) was added dropwise, and the mixture was heated at 80 °C for 15 h. The mixture was allowed to cool to room temperature and quenched with aq. HCl (1 N, 25 mL). The mixture was extracted with EtOAc (3 × 50 mL), washed with brine (150 mL), dried (MgSO_4_), and concentrated under reduced pressure. Purification by flash column chromatography [hexane:EtOAc 4:1] gave **16g** (580 mg, 86%) as a colorless solid. *R*_f_ = 0.67 [petrol:EtOAc 1:1]. m.p.: 112–114 °C. ^1^H NMR (400 MHz, CDCl_3_) δ 7.78 (d, *J* = 8.4 Hz, 2H), 7.15 (d, *J* = 8.1 Hz, 2H), 5.56 (ddt, *J* = 17.1, 10.4, 5.9 Hz, 1H), 5.12 (dq, *J* = 17.2, 1.4 Hz, 1H), 5.05 (dq, *J* = 10.4, 1.1 Hz, 1H), 4.56 (dq, *J* = 5.8, 1.0 Hz, 2H), 3.55–3.36 (m, 2H), 2.77 (ddd, *J* = 15.0, 9.7, 3.2 Hz, 1H), 2.56 (ddd, *J* = 15.0, 7.8, 2.8 Hz, 1H), 2.30 (s, 3H), 2.01 (quint, *J* = 5.9 Hz, 2H), 1.66–1.44 (m, 2H) ppm. ^13^C{^1^H} NMR (100 MHz, CDCl_3_): δ 187.2, 165.5, 144.6, 132.2, 129.8, 129.2, 128.9, 119.5, 79.9, 66.9, 52.4, 32.9, 23.6, 21.3, 19.3 ppm. IR: ν_max_ (neat) 2939, 2868, 1735, 1677 cm^–1^. HRMS (APCI) *m/z*: calcd for C_17_H_21_O_5_S [M + H]^+^ 337.1104, found 337.1091.

#### Allyl-2-(4-methoxybenzoyl)-1,1-dioxo-thiane-2-carboxylate (**16h**)

**25** (436 mg, 2.0 mmol) was dissolved in THF (15 mL). NaHMDS (1 M in THF, 2.20 mL, 2.2 mmol) was added dropwise. The solution was stirred at room temperature for 30 min. 4-Methoxybenzoyl chloride (0.298 mL, 2.2 mmol) was added dropwise, and the mixture was heated at 80 °C for 15 h. The mixture was allowed to cool to room temperature and quenched with aq. HCl (1 N, 25 mL). The mixture was extracted with EtOAc (3 × 50 mL), washed with brine (150 mL), dried (MgSO_4_), and concentrated under reduced pressure. Purification by flash column chromatography [hexane:EtOAc 3:1] gave an inseparable mixture of starting material **25** and **16h** in a 1:8.1 ratio (673 mg, corresponding to 625 mg of pure **16h**, 89%) as a colorless oil. *R*_f_ = 0.59 [petrol:EtOAc 1:1]. ^1^H NMR (400 MHz, CDCl_3_, resonances due to **16h** quoted) δ 7.87 (d, *J* = 9.0 Hz, 2H), 6.82 (d, *J* = 9.1 Hz, 2H), 5.58 (ddt, *J* = 17.1, 10.4, 5.9 Hz, 1H), 5.12 (dq, *J* = 17.2, 1.4 Hz, 1H), 5.06 (dq, *J* = 10.4, 1.1 Hz, 1H), 4.56 (dq, *J* = 5.8, 1.5 Hz, 2H), 3.75 (s, 3H), 3.51 (dt, *J* = 13.5, 6.6 Hz, 1H), 3.43–3.32 (m, 1H), 2.76 (ddd, *J* = 14.8, 10.1, 3.0 Hz, 1H), 2.52 (ddd, *J* = 14.9, 7.9, 2.7 Hz, 1H), 2.04–1.96 (m, 2H), 1.67–1.55 (m, *J* = 5.4, 4.7 Hz, 1H), 1.55–1.42 (m, 1H) ppm. ^13^C{^1^H} NMR (100 MHz, CDCl_3_, resonances due to **16h** quoted): δ 185.7, 165.8, 163.8, 131.8, 129.9, 127.5, 119.5, 113.4, 79.8, 66.9, 55.2, 52.4, 32.9, 23.6, 19.3 ppm. IR: ν_max_ (neat) 2939, 1735, 1671 cm^–1^. HRMS (ESI) *m/z*: calcd for C_17_H_20_NaO_6_S [M + Na]^+^ 375.0873, found 375.0870.

#### Allyl-2-(furan-2-carbonyl)-1,1-dioxo-thiane-2-carboxylate (**16i**)

**25** (436 mg, 2.0 mmol) was dissolved in THF (15 mL). NaHMDS (1 M in THF, 2.20 mL, 2.2 mmol) was added dropwise. The solution was stirred at room temperature for 30 min. 2-Furoyl chloride (0.217 mL, 2.2 mmol) was added dropwise, and the mixture was heated at 80 °C for 15 h. The mixture was allowed to cool to room temperature and quenched with aq. HCl (1 N, 25 mL). The mixture was extracted with EtOAc (3 × 50 mL), washed with brine (150 mL), dried (MgSO_4_), and concentrated under reduced pressure. Purification by flash column chromatography [hexane:EtOAc 3:1] gave an inseparable mixture of starting material **25** and **16i** in a 1:6.8 ratio (497 mg, corresponding to 420 mg of pure **16i**, 72%) as a yellow oil. *R*_f_ = 0.40 [petrol:EtOAc 1:1]. ^1^H NMR (400 MHz, CDCl_3_, resonances due to **16i** quoted) δ 7.49 (dd, *J* = 1.6, 0.5 Hz, 1H), 7.23 (dd, *J* = 3.7, 0.8 Hz, 1H), 6.45 (dd, *J* = 3.7, 1.7 Hz, 1H), 5.58 (ddt, *J* = 17.2, 10.4, 5.8 Hz, 1H), 5.09 (dq, *J* = 17.2, 1.5 Hz, 1H), 5.01 (dq, *J* = 10.5, 1.2 Hz, 1H), 4.55 (dq, *J* = 5.9, 1.5 Hz, 2H), 3.46–3.26 (m, 2H), 2.59 (ddd, *J* = 15.2, 7.4, 4.1 Hz, 1H), 2.49 (ddd, *J* = 15.1, 8.7, 4.0 Hz, 1H), 1.93 (quint, *J* = 6.1 Hz, 2H), 1.55–1.38 (m, 2H) ppm. ^13^C{^1^H} NMR (100 MHz, CDCl_3_, resonances due to **16i** quoted): δ 176.3, 163.7, 149.9, 147.3, 130.1, 120.5, 119.0, 112.6, 79.0, 66.7, 52.2, 31.4, 23.4, 19.0 ppm. IR: ν_max_ (neat) 3144, 2950, 1748, 1649, 1317, 1125 cm^–1^. HRMS (APCI) *m/z*: calcd for C_14_H_17_O_6_S [M + H]^+^ 313.0740, found 313.0731.

#### Allyl-2-(2-methylpropanoyl)-1,1-dioxo-thiane-2-carboxylate (**16j**)

**25** (300 mg, 1.37 mmol) was dissolved in THF (20 mL). NaHMDS (1 M in THF, 1.51 mL, 1.51 mmol) was added dropwise. The solution was stirred at room temperature for 30 min. Isobutyryl chloride (0.150 mL, 1.51 mmol) was added dropwise, and the mixture was heated at 80 °C for 15 h. The mixture was allowed to cool to room temperature and quenched with aq. HCl (1 N, 25 mL). The mixture was extracted with EtOAc (3 × 50 mL), washed with brine (150 mL), dried (MgSO_4_), and concentrated under reduced pressure. Purification by flash column chromatography [hexane:EtOAc 9:1–4:1] gave **16j** (191 mg, 48%) as a yellow oil. *R*_f_ = 0.32 [petrol:EtOAc 4:1]. ^1^H NMR (400 MHz, CDCl_3_) δ 5.89 (ddt, *J* = 17.2, 10.3, 6.1 Hz, 1H), 5.38 (dq, *J* = 17.2, 1.4 Hz, 1H), 5.30 (dq, *J* = 10.4, 1.1 Hz, 1H), 4.81–4.65 (m, 2H), 3.60 (ddd, *J* = 15.0, 9.5, 6.3 Hz, 1H), 3.20 (dt, *J* = 14.0, 5.0 Hz, 1H), 3.05 (hept, *J* = 6.6 Hz, 1H), 2.48 (ddd, *J* = 15.0, 11.5, 3.4 Hz, 1H), 2.41–2.32 (m, 1H), 2.08–1.99 (m, 2H), 1.76–1.65 (m, 1H), 1.58–1.46 (m, 1H), 1.16 (d, *J* = 6.6 Hz, 3H), 1.11 (d, *J* = 6.7 Hz, 3H) ppm. ^13^C{^1^H} NMR (100 MHz, CDCl_3_): δ 202.4, 165.1, 130.2, 120.7, 81.4, 67.4, 52.4, 40.6, 31.3, 24.0, 20.7, 19.9, 19.9 ppm. IR: ν_max_ (neat) 2976, 2939, 2876, 1744, 1716 cm^–1^. HRMS (APCI) *m/z*: calcd for C_13_H_21_O_5_S [M + H]^+^ 289.1104, found 289.1097.

#### Allyl-2-(cyclohexanecarbonyl)-1,1-dioxo-thiane-2-carboxylate (**16k**)

**25** (436 mg, 2.0 mmol) was dissolved in THF (15 mL). NaHMDS (1 M in THF, 2.20 mL, 2.2 mmol) was added dropwise. The solution was stirred at room temperature for 30 min. Cyclohexanecarbonyl chloride (0.294 mL, 2.2 mmol) was added dropwise, and the mixture was heated at 80 °C for 15 h. The mixture was allowed to cool to room temperature and quenched with aq. HCl (1 N, 25 mL). The mixture was extracted with EtOAc (3 × 50 mL), washed with brine (150 mL), dried (MgSO_4_), and concentrated under reduced pressure. Purification by flash column chromatography [hexane:EtOAc 4:1] gave **16k** (403 mg, 61%) as a colorless solid. *R*_f_ = 0.71 [petrol:EtOAc 1:1]. m.p.: 78–79 °C. ^1^H NMR (400 MHz, CDCl_3_) δ 5.81 (ddt, *J* = 16.5, 10.4, 6.0 Hz, 1H), 5.29 (dq, *J* = 17.2, 1.2 Hz, 1H), 5.21 (dq, *J* = 10.4, 1.0 Hz, 1H), 4.64 (dtd, *J* = 5.7, 2.4, 1.1 Hz, 2H), 3.48 (ddd, *J* = 14.4, 9.9, 5.0 Hz, 1H), 3.10 (dt, *J* = 14.0, 4.7 Hz, 1H), 2.65 (tt, *J* = 11.4, 3.1 Hz, 1H), 2.36 (ddd, *J* = 14.8, 11.3, 3.3 Hz, 1H), 2.25 (ddt, *J* = 15.2, 5.3, 2.6 Hz, 1H), 2.00–1.88 (m, 3H), 1.68–1.50 (m, 5H), 1.48–1.27 (m, 2H), 1.25–1.06 (m, 4H) ppm. ^13^C{^1^H} NMR (100 MHz, CDCl_3_): δ 200.5, 164.7, 130.0, 120.2, 81.0, 67.0, 52.0, 50.4, 30.9, 30.2, 29.2, 25.2, 25.0, 24.9, 23.6, 19.5 ppm. IR: ν_max_ (neat) 2926, 2853, 1746, 1716 cm^–1^. HRMS (APCI) *m/z*: calcd for C_16_H_25_O_5_S [M + H]^+^ 329.1417, found 329.1427.

#### Allyl-2-(tetrahydropyran-4-carbonyl)-1,1-dioxo-thiane-2-carboxylate (**16l**)

**25** (436 mg, 2.0 mmol) was dissolved in THF (15 mL). NaHMDS (1 M in THF, 2.20 mL, 2.2 mmol) was added dropwise. The solution was stirred at room temperature for 30 min. Tetrahydro-2*H*-pyran-4-carbonyl chloride (327 mg, 2.2 mmol) was added, and the mixture was heated at 80 °C for 15 h. The mixture was allowed to cool to room temperature and quenched with aq. HCl (1 N, 25 mL). The mixture was extracted with EtOAc (3 × 50 mL), washed with brine (150 mL), dried (MgSO_4_), and concentrated under reduced pressure. Purification by flash column chromatography [hexane:EtOAc 3:1] gave an inseparable mixture of starting material **25** and **16l** in a 1:4.1 ratio (504 mg, corresponding to 434 mg of pure **16l**, 66%) as a colorless oil. *R*_f_ = 0.62 [petrol:EtOAc 1:1]. ^1^H NMR (400 MHz, CDCl_3_, resonances due to **16l** quoted) δ 5.80–5.66 (m, 1H), 5.21 (dq, *J* = 17.2, 1.4 Hz, 1H), 5.14 (dd, *J* = 10.4, 1.1 Hz, 1H), 4.61–4.53 (m, 2H), 3.73 (ddd, *J* = 12.0, 4.4, 2.0 Hz, 2H), 3.49 (ddd, *J* = 14.3, 12.0, 4.2 Hz, 1H), 3.16 (tt, *J* = 11.8, 2.2 Hz, 2H), 2.99 (dt, *J* = 14.2, 4.3 Hz, 1H), 2.91 (tt, *J* = 11.5, 3.6 Hz, 1H), 2.28 (ddd, *J* = 15.4, 12.4, 3.3 Hz, 1H), 2.18–2.08 (m, 1H), 1.96–1.79 (m, 2H), 1.79–1.71 (m, 1H), 1.60 (td, *J* = 12.3, 4.1 Hz, 2H), 1.51–1.31 (m, 3H) ppm. ^13^C{^1^H} NMR (100 MHz, CDCl_3_, resonances due to **16l** quoted): δ 198.6, 164.4, 129.9, 120.2, 80.5, 66.8, 66.2, 66.0, 51.8, 47.3, 30.7, 29.5, 28.8, 23.3, 19.2 ppm. IR: ν_max_ (neat) 2957, 2853, 1746, 1716 cm^–1^. HRMS (APCI) *m/z*: calcd for C_15_H_23_O_6_S [M + H]^+^ 331.1210, found 331.1207.

#### Allyl-2-(2,2-dimethylpropanoyl)-1,1-dioxo-thiane-2-carboxylate (**16m**)

**25** (436 mg, 2.0 mmol) was dissolved in THF (15 mL). NaHMDS (1 M in THF, 2.20 mL, 2.2 mmol) was added dropwise. The solution was stirred at room temperature for 30 min. Pivaloyl chloride (0.271 mL, 2.2 mmol) was added dropwise, and the mixture was heated at 80 °C for 15 h. The mixture was allowed to cool to room temperature and quenched with aq. HCl (1 N, 25 mL). The mixture was extracted with EtOAc (3 × 50 mL), washed with brine (150 mL), dried (MgSO_4_), and concentrated under reduced pressure. Purification by flash column chromatography [hexane:EtOAc 4:1] gave **16m** (138 mg, 23%) as a colorless oil. *R*_f_ = 0.69 [petrol:EtOAc 1:1]. ^1^H NMR (400 MHz, CDCl_3_) δ 5.88 (ddt, *J* = 17.3, 10.4, 5.9 Hz, 1H), 5.37 (dq, *J* = 17.2, 1.4 Hz, 1H), 5.27 (dq, *J* = 10.4, 1.1 Hz, 1H), 4.70 (ddt, *J* = 5.8, 2.3, 1.3 Hz, 2H), 3.47–3.32 (m, 2H), 2.56 (ddd, *J* = 15.3, 8.9, 3.8 Hz, 1H), 2.43 (ddd, *J* = 15.3, 7.6, 3.8 Hz, 1H), 1.99 (quint, *J* = 6.6, 6.2 Hz, 2H), 1.62–1.42 (m, 2H), 1.21 (s, 9H) ppm. ^13^C{^1^H} NMR (100 MHz, CDCl_3_): δ 203.2, 164.6, 130.1, 120.2, 81.3, 67.1, 52.7, 46.9, 32.4, 27.9, 23.8, 19.6 ppm. IR: ν_max_ (neat) 2939, 1742, 1697 cm^–1^. HRMS (APCI) *m/z*: calcd for C_14_H_23_O_5_S [M + H]^+^ 303.1261, found 303.1267.

#### Allyl-2-acetyl-1,1-dioxo-thiane-2-carboxylate (**16n**)

**25** (300 mg, 1.37 mmol) was dissolved in THF (20 mL). NaHMDS (1 M in THF, 1.51 mL, 1.51 mmol) was added dropwise. The solution was stirred at room temperature for 30 min. Acetyl chloride (0.110 mL, 1.51 mmol) was added dropwise, and the mixture was heated at 80 °C for 15 h. The mixture was allowed to cool to room temperature and quenched with aq. HCl (1 N, 25 mL). The mixture was extracted with EtOAc (3 × 50 mL), washed with brine (150 mL), dried (MgSO_4_), and concentrated under reduced pressure. Purification by flash column chromatography [hexane:EtOAc 9:1–4:1] gave **16n** (132 mg, 37%) as a yellow oil. *R*_f_ = 0.33 [petrol:EtOAc 4:1]. ^1^H NMR (300 MHz, CDCl_3_) δ 5.90 (ddt, *J* = 17.2, 10.4, 5.9 Hz, 1H), 5.38 (dq, *J* = 17.1, 1.4 Hz, 1H), 5.32 (dq, *J* = 10.4, 1.1 Hz, 1H), 4.74 (dtd, *J* = 6.0, 1.5, 1.0 Hz, 2H), 3.74–3.57 (m, 1H), 3.17 (dt, *J* = 13.7, 4.3 Hz, 1H), 2.53–2.31 (m, 5H), 2.14–1.98 (m, 2H), 1.83–1.70 (m, 1H), 1.70–1.51 (m, 1H) ppm. ^13^C{^1^H} NMR (75 MHz, CDCl_3_): δ 194.1, 165.0, 130.2, 120.5, 80.7, 67.3, 52.1, 31.1, 29.6, 23.9, 19.7 ppm. IR: ν_max_ (neat) 2939, 2870, 1718 cm^–1^. HRMS (APCI) *m/z*: calcd for C_11_H_17_O_5_S [M + H]^+^ 261.0791, found 261.0786.

#### 2-Allyl-2′-phenyl-4-*tert*-butyl-1,1-dioxo-1,4-thiazinane-2,2,4-tricarboxylate (**17a**)

**26** (638 mg, 2.0 mmol) was dissolved in THF (15 mL). NaHMDS (1 M in THF, 2.20 mL, 2.2 mmol) was added dropwise. The solution was stirred at room temperature for 30 min. Phenyl chloroformate (0.276 mL, 2.2 mmol) was added dropwise, and the mixture was stirred for 15 h. The reaction was quenched with aq. HCl (1 N, 25 mL). The mixture was extracted with EtOAc (3 × 50 mL), washed with brine (150 mL), dried (MgSO_4_), and concentrated under reduced pressure. Purification by flash column chromatography [hexane:EtOAc 6:1] gave **17a** (741 mg, 84%) as a colorless solid. *R*_f_ = 0.53 [petrol:EtOAc 2:1]. m.p.: 103–105 °C. ^1^H NMR (400 MHz, DMSO-*d*_6_, 130 °C) δ 7.47 (t, *J* = 8.2 Hz, 2H), 7.34 (tt, *J* = 7.4, 1.2 Hz, 1H), 7.17 (dd, *J* = 8.6, 1.1 Hz, 2H), 5.99 (ddt, *J* = 17.2, 10.9, 5.5 Hz, 1H), 5.45 (dq, *J* = 17.3, 1.5 Hz, 1H), 5.32 (dq, *J* = 10.6, 1.3 Hz, 1H), 4.84 (ddt, *J* = 7.0, 5.6, 1.4 Hz, 2H), 4.38 (s, 2H), 3.99 (ddd, *J* = 14.8, 6.8, 4.3 Hz, 1H), 3.90 (ddd, *J* = 14.7, 7.2, 4.2 Hz, 1H), 3.66–3.52 (m, 2H), 1.43 (s, 9H) ppm. ^13^C{^1^H} NMR (100 MHz, DMSO-*d*_6_, 130 °C): δ 161.3, 160.4, 152.5, 149.3, 130.3, 129.0, 126.0, 120.0, 118.3, 80.5, 74.8, 66.7, 50.6, 48.1, 41.8, 27.2 ppm. IR: ν_max_ (neat) 2978, 1742, 1701 cm^–1^. HRMS (ESI) *m/z*: calcd for C_20_H_25_NNaO_8_S [M + Na]^+^ 462.1193, found 462.1199.

#### 2-Allyl-2′-benzyl-4-*tert*-butyl-1,1-dioxo-1,4-thiazinane-2,2,4-tricarboxylate (**17b**)

**26** (638 mg, 2.0 mmol) was dissolved in THF (15 mL). NaHMDS (1 M in THF, 2.20 mL, 2.2 mmol) was added dropwise. The solution was stirred at room temperature for 30 min. Benzyl chloroformate (0.314 mL, 2.2 mmol) was added dropwise, and the mixture was stirred for 15 h. The reaction was quenched with aq. HCl (1 N, 25 mL). The mixture was extracted with EtOAc (3 × 50 mL), washed with brine (150 mL), dried (MgSO_4_), and concentrated under reduced pressure. Purification by flash column chromatography [hexane:EtOAc 6:1] gave **17b** (755 mg, 83%) as a colorless oil. *R*_f_ = 0.53 [petrol:EtOAc 2:1]. ^1^H NMR (400 MHz, DMSO-*d*_6_, 130 °C) δ 7.40–7.35 (m, 5H), 5.83 (ddt, *J* = 17.2, 10.8, 5.5 Hz, 1H), 5.33 (dq, *J* = 17.2, 1.6 Hz, 1H), 5.32 (d, *J* = 12.5 Hz, 1H), 5.26 (d, *J* = 12.6 Hz, 1H), 5.22 (dq, *J* = 10.6, 1.3 Hz, 1H), 4.69 (ddt, *J* = 8.6, 5.5, 1.5 Hz, 2H), 4.31–4.22 (m, 2H), 3.89 (q, *J* = 5.8 Hz, 2H), 3.52 (ddd, *J* = 6.2, 4.9, 0.7 Hz, 2H), 1.43 (s, 9H) ppm. ^13^C{^1^H} NMR (100 MHz, DMSO-*d*_6_, 130 °C): δ 161.5, 161.4, 152.4, 133.9, 130.2, 127.6, 127.6, 127.1, 118.0, 80.3, 74.6, 67.6, 66.3, 50.4, 48.2, 41.7, 27.2 ppm. IR: ν_max_ (neat) 2978, 1735, 1701 cm^–1^. HRMS (ESI) *m/z*: calcd for C_21_H_27_NNaO_8_S [M + Na]^+^ 476.1350, found 476.1364.

#### 2-Allyl-4-tert-butyl-2-benzoyl-1,1-dioxo-1,4-thiazinane-2,4-dicarboxylate (**17c**)

**26** (638 mg, 2.0 mmol) was dissolved in THF (15 mL). NaHMDS (1 M in THF, 2.20 mL, 2.2 mmol) was added dropwise. The solution was stirred at room temperature for 30 min. Benzoyl chloride (0.256 mL, 2.2 mmol) was added dropwise, and the mixture was heated at 80 °C for 15 h. The mixture was allowed to cool to room temperature and quenched with aq. HCl (1 N, 25 mL). The mixture was extracted with EtOAc (3 × 50 mL), washed with brine (150 mL), dried (MgSO_4_), and concentrated under reduced pressure. Purification by flash column chromatography [hexane:EtOAc 6:1] gave **17c** (535 mg, 63%) as a colorless solid. *R*_f_ = 0.45 [petrol:EtOAc 2:1]. m.p.: 149–150 °C. ^1^H NMR (400 MHz, DMSO-*d*_6_, 130 °C) δ 7.85 (dd, *J* = 8.5, 1.2 Hz, 2H), 7.67 (tt, *J* = 7.6, 1.6 Hz, 1H), 7.53 (t, *J* = 7.2 Hz, 2H), 5.69 (ddt, *J* = 17.2, 10.5, 5.7 Hz, 1H), 5.20 (dq, *J* = 17.3, 1.5 Hz, 1H), 5.15 (dq, *J* = 10.5, 1.3 Hz, 1H), 4.67 (ddt, *J* = 13.2, 5.7, 1.4 Hz, 1H), 4.58 (ddt, *J* = 13.2, 5.7, 1.4 Hz, 1H), 4.48 (d, *J* = 4.1 Hz, 2H), 3.92 (ddt, *J* = 15.5, 10.3, 5.8 Hz, 2H), 3.62 (ddd, *J* = 6.6, 4.9, 3.8 Hz, 2H), 1.29 (s, 9H) ppm. ^13^C{^1^H} NMR (100 MHz, DMSO-*d*_6_, 130 °C): δ 187.4, 163.1, 152.3, 134.9, 133.1, 129.8, 127.9, 127.9, 118.5, 80.3, 78.8, 66.4, 51.2, 49.3, 41.6, 26.9 ppm. IR: ν_max_ (neat) 2976, 2933, 1735, 1697, 1671 cm^–1^. HRMS (ESI) *m/z*: calcd for C_20_H_25_NNaO_7_S [M + Na]^+^ 446.1244, found 446.1239.

#### 2-Allyl-4-*tert*-butyl-2-(2-methylpropanoyl)-1,1-dioxo-1,4-thiazinane-2,4-dicarboxylate (**17d**)

**26** (638 mg, 2.0 mmol) was dissolved in THF (15 mL). NaHMDS (1 M in THF, 2.20 mL, 2.2 mmol) was added dropwise. The solution was stirred at room temperature for 30 min. Isobutyryl chloride (0.234 mL, 2.2 mmol) was added dropwise, and the mixture was heated at 80 °C for 15 h. The mixture was allowed to cool to room temperature and quenched with aq. HCl (1 N, 25 mL). The mixture was extracted with EtOAc (3 × 50 mL), washed with brine (150 mL), dried (MgSO_4_), and concentrated under reduced pressure. Purification by flash column chromatography [hexane:EtOAc 9:1] gave **17d** (496 mg, 64%) as a colorless solid. *R*_f_ = 0.61 [petrol:EtOAc 2:1]. m.p.: 86–88 °C. ^1^H NMR (400 MHz, DMSO-*d*_6_, 130 °C) δ 5.96 (ddt, *J* = 17.2, 10.5, 5.8 Hz, 1H), 5.43 (dq, *J* = 17.2, 1.5 Hz, 1H), 5.32 (dq, *J* = 10.5, 1.2 Hz, 1H), 4.75 (tt, *J* = 5.7, 1.4 Hz, 2H), 4.41 (dd, *J* = 15.0, 2.0 Hz, 1H), 4.13 (dtd, *J* = 11.5, 4.6, 1.8 Hz, 1H), 3.95 (d, *J* = 15.0 Hz, 1H), 3.63 (d, *J* = 10.8 Hz, 1H), 3.57 (d, *J* = 11.0 Hz, 1H), 3.42 (dd, *J* = 10.6, 5.2 Hz, 1H), 3.07 (hept, *J* = 6.6 Hz, 1H), 1.43 (s, 9H), 1.15 (d, *J* = 6.7 Hz, 3H), 1.12 (d, *J* = 6.6 Hz, 3H) ppm. ^13^C{^1^H} NMR (100 MHz, DMSO-*d*_6_, 130 °C): δ 200.7, 162.8, 152.4, 130.1, 118.9, 80.2, 79.1, 66.6, 50.8, 47.8, 41.7, 39.5, 27.2, 19.1, 18.4 ppm. IR: ν_max_ (neat) 2978, 2946, 1716, 1694 cm^–1^. HRMS (ESI) *m/z*: calcd for C_17_H_27_NNaO_7_S [M + Na]^+^ 412.1400, found 412.1416.

### Synthesis of Allylated Products **18**–**20**

#### (2*R*)-Benzyl-2-allyltetrahydrothiophene-2-carboxylate-1,1-dioxide (**18a**)

A vial was charged with **15a** (51 mg, 0.15 mmol), [Pd_2_dba_3_] (3.5 mg, 3.75 μmol), **L4** (8.0 mg, 9.75 μmol), and 1,4-dioxane (1.5 mL). The mixture was stirred at room temperature for 2 h and then concentrated under reduced pressure. Purification by flash column chromatography [hexane:EtOAc 4:1] gave **18a** (40 mg, 91%) as a yellow oil. *R*_f_ = 0.20 [petrol:EtOAc 4:1]. ^1^H NMR (400 MHz, CDCl_3_) δ 7.44–7.29 (m, 5H), 5.55 (dddd, *J* = 16.7, 10.1, 7.9, 6.4 Hz, 1H), 5.29 (d, *J* = 12.3 Hz, 1H), 5.19 (d, *J* = 12.2 Hz, 1H), 5.16–5.08 (m, 2H), 3.25–3.16 (m, 1H), 3.15–3.03 (m, 2H), 2.81–2.72 (m, 1H), 2.42 (ddt, *J* = 14.1, 7.9, 1.1 Hz, 1H), 2.31–2.18 (m, 1H), 2.14–2.01 (m, 2H) ppm. ^13^C{^1^H} NMR (100 MHz, CDCl_3_): δ 167.4, 134.9, 130.9, 128.5, 128.4, 128.4, 120.5, 70.2, 68.3, 51.3, 37.1, 30.8, 18.3 ppm. IR: ν_max_ (neat) 3066, 3034, 2954, 1733 cm^–1^. HRMS (ESI) *m/z*: calcd for C_15_H_19_O_4_S [M + H]^+^ 295.0999, found 295.0986. HPLC: 86% ee (Chiralpak AD-H, hexane:*i*-PrOH = 95:5, flow rate = 1 mL/min, 30.0 °C, λ = 220 nm) *t*_R_ = 20.8 min (major), *t*_R_ = 22.9 min (minor). [α]: −55.1 (*c* = 0.12, CHCl_3_).

#### (2*R*)-Phenyl-2-allyltetrahydrothiophene-2-carboxylate-1,1-dioxide (**18b**)

A vial was charged with **15b** (48.5 mg, 0.15 mmol), [Pd_2_dba_3_] (3.5 mg, 3.75 μmol), **L4** (8.0 mg, 9.75 μmol), and 1,4-dioxane (1.5 mL). The mixture was stirred at room temperature for 2 h and then concentrated under reduced pressure. Purification by flash column chromatography [hexane:EtOAc 4:1] gave **18b** (39 mg, 90%) as a yellow oil. *R*_f_ = 0.20 [petrol:EtOAc 4:1]. ^1^H NMR (300 MHz, CDCl_3_) δ 7.43–7.35 (m, 2H), 7.25 (tt, *J* = 7.4, 1.3 Hz, 1H), 7.15 (d, *J* = 7.4 Hz, 2H), 5.79 (ddt, *J* = 17.1, 10.0, 7.1 Hz, 1H), 5.37–5.23 (m, 2H), 3.35–3.22 (m, 2H), 3.21–3.09 (m, 1H), 2.94–2.82 (m, 1H), 2.54 (ddt, *J* = 14.2, 7.5, 1.1 Hz, 1H), 2.38–2.24 (m, 1H), 2.20–2.08 (m, 2H) ppm. ^13^C{^1^H} NMR (75 MHz, CDCl_3_): δ 166.4, 150.6, 130.9, 129.5, 126.4, 121.4, 120.9, 70.3, 51.8, 37.4, 31.2, 18.6 ppm. IR: ν_max_ (neat) 3079, 2952, 1750 cm^–1^. HRMS (APCI) *m/z*: calcd for C_14_H_17_O_4_S [M + H]^+^ 281.0842, found 281.0832. HPLC: 94% ee (Chiralcel OD-H, hexane:*i*-PrOH = 90:10, flow rate = 1 mL/min, 30.0 °C, λ = 220 nm) *t*_R_ = 14.4 min (major), *t*_R_ = 17.4 min (minor). [α]: −68.5 (*c* = 0.07, CHCl_3_).

#### (2*R*)-4-Methoxyphenyl-2-allyltetrahydrothiophene-2-carboxylate-1,1-dioxide (**18c**)

A vial was charged with a 1:2.3 mixture of **24**:**15c** (67 mg, corresponding to 53.5 mg of pure **15c**, 0.15 mmol), [Pd_2_dba_3_] (3.5 mg, 3.75 μmol), **L4** (8.0 mg, 9.75 μmol), and 1,4-dioxane (1.5 mL). The mixture was stirred at room temperature for 2 h and then concentrated under reduced pressure. Purification by flash column chromatography [petrol:EtOAc 2:1] gave **18c** (27.5 mg, 59%) as a colorless solid. *R*_f_ = 0.28 [petrol:EtOAc 2:1]. m.p.: 88–89 °C. ^1^H NMR (400 MHz, CDCl_3_) δ 7.06 (d, *J* = 9.1 Hz, 2H), 6.88 (d, *J* = 9.1 Hz, 2H), 5.77 (ddt, *J* = 17.1, 9.9, 7.2 Hz, 1H), 5.32 (d, *J* = 17.0 Hz, 1H), 5.27 (d, *J* = 10.3 Hz, 1H), 3.79 (s, 3H), 3.27 (dt, *J* = 13.4, 6.7 Hz, 2H), 3.14 (dt, *J* = 12.7, 7.5 Hz, 1H), 2.92–2.83 (m, 1H), 2.53 (dd, *J* = 14.3, 7.4 Hz, 1H), 2.36–2.25 (m, 1H), 2.13 (ddd, *J* = 13.8, 11.2, 6.3 Hz, 2H) ppm. ^13^C{^1^H} NMR (100 MHz, CDCl_3_): δ 166.7, 157.6, 144.1, 131.0, 122.1, 120.8, 114.5, 70.2, 55.6, 51.7, 37.3, 31.2, 18.5 ppm. IR: ν_max_ (neat) 2945, 1752 cm^–1^. HRMS (APCI) *m/z*: calcd for C_15_H_18_NaO_5_S [M + Na]^+^ 333.0767, found 333.0755. HPLC: 93% ee (Chiralpak AD-H, hexane:*i*-PrOH = 90:10, flow rate = 1 mL/min, 30.0 °C, λ = 220 nm) *t*_R_ = 22.1 min (major), *t*_R_ = 26.2 min (minor). [α]: −46.7 (*c* = 0.23, CHCl_3_).

#### (2*R*)-*p*-Tolyl-2-allyltetrahydrothiophene-2-carboxylate-1,1-dioxide (**18d**)

A vial was charged with **15d** (51 mg, 0.15 mmol), [Pd_2_dba_3_] (3.5 mg, 3.75 μmol), **L4** (8.0 mg, 9.75 μmol), and 1,4-dioxane (1.5 mL). The mixture was stirred at room temperature for 2 h and then concentrated under reduced pressure. Purification by flash column chromatography [petrol:EtOAc 4:1] gave **18d** (38.5 mg, 88%) as colorless crystals. *R*_f_ = 0.37 [petrol:EtOAc 2:1]. m.p.: 106–108 °C. ^1^H NMR (400 MHz, CDCl_3_) δ 7.17 (d, *J* = 8.7 Hz, 2H), 7.02 (d, *J* = 8.5 Hz, 2H), 5.77 (ddt, *J* = 17.1, 10.0, 7.2 Hz, 1H), 5.33 (dd, *J* = 17.0, 1.5 Hz, 1H), 5.27 (dd, *J* = 10.2, 1.6 Hz, 1H), 3.33–3.23 (m, 2H), 3.18–3.10 (m, 1H), 2.92–2.82 (m, 1H), 2.53 (dd, *J* = 14.1, 7.5 Hz, 1H), 2.34 (s, 3H), 2.34–2.25 (m, 1H), 2.20–2.08 (m, 2H) ppm. ^13^C{^1^H} NMR (100 MHz, CDCl_3_): δ 166.5, 148.4, 136.0, 131.0, 130.0, 121.0, 120.8, 70.2, 51.7, 37.3, 31.2, 20.9, 18.5 ppm. IR: ν_max_ (neat) 2945, 1746 cm^–1^. HRMS (APCI) *m/z*: calcd for C_15_H_19_O_4_S [M + H]^+^ 295.0999, found 295.0989. HPLC: 84% ee (Chiralpak AD-H, hexane:*i*-PrOH = 90:10, flow rate = 1 mL/min, 30.0 °C, λ = 220 nm) *t*_R_ = 13.4 min (major), *t*_R_ = 15.5 min (minor). [α]: −56.2 (*c* = 0.27, CHCl_3_).

#### (2*R*)-9*H*-Fluoren-9-yl-2-allyltetrahydrothiophene-2-carboxylate-1,1-dioxide (**18e**)

A vial was charged with **15e** (64 mg, 0.15 mmol), [Pd_2_dba_3_] (6.9 mg, 7.5 μmol), **L4** (15.9 mg, 19.5 μmol), and 1,4-dioxane (1.5 mL). The mixture was stirred at room temperature for 3 h and then concentrated under reduced pressure. Purification by flash column chromatography [petrol:EtOAc 4:1] gave **18e** (42 mg, 76%) as a clear oil. *R*_f_ = 0.30 [petrol:EtOAc 2:1]. ^1^H NMR (400 MHz, CDCl_3_) δ 7.76 (d, *J* = 7.5 Hz, 2H), 7.68 (d, *J* = 7.5 Hz, 1H), 7.63 (d, *J* = 7.4 Hz, 1H), 7.41 (t, *J* = 7.5 Hz, 2H), 7.34 (t, *J* = 7.3 Hz, 2H), 5.44 (dddd, *J* = 16.1, 9.8, 8.1, 6.0 Hz, 1H), 5.16–5.04 (m, 2H), 4.79 (dd, *J* = 10.8, 5.5 Hz, 1H), 4.45 (dd, *J* = 10.7, 6.6 Hz, 1H), 4.28 (t, *J* = 6.1 Hz, 1H), 3.13–2.96 (m, 3H), 2.59–2.49 (m, 1H), 2.40 (dd, *J* = 14.0, 8.2 Hz, 1H), 2.11–1.92 (m, 3H) ppm. ^13^C{^1^H} NMR (100 MHz, CDCl_3_): δ 167.2, 143.7, 143.2, 141.4, 141.2, 131.0, 127.9, 127.8, 127.2, 125.1, 124.9, 120.3, 119.9, 119.9, 70.1, 67.9, 51.0, 46.8, 36.8, 30.7, 18.1 ppm. IR: ν_max_ (neat) 2945, 1733 cm^–1^. HRMS (ESI) *m/z*: calcd for C_22_H_22_NaO_4_S [M + Na]^+^ 405.1131, found 405.1128. HPLC: 80% ee (Chiralpak AD-H, hexane:*i*-PrOH = 90:10, flow rate = 1 mL/min, 30.0 °C, λ = 245 nm) *t*_R_ = 20.1 min (major), *t*_R_ = 28.6 min (minor). [α]: +4.7 (*c* = 0.69, CHCl_3_).

#### (2*R*)-Allyl-2-allyl-1,1-dioxo-thiolane-2-carboxylate (**18f**)

A vial was charged with **15f** (43 mg, 0.15 mmol), [Pd_2_dba_3_] (6.9 mg, 7.5 μmol), **L4** (15.9 mg, 19.5 μmol), and 1,4-dioxane (1.5 mL). The mixture was stirred at room temperature for 15 h and then concentrated under reduced pressure. Purification by flash column chromatography [hexane:EtOAc 5:1] gave **18f** (19.5 mg, 53%) as a colorless oil. *R*_f_ = 0.36 [petrol:EtOAc 2:1]. ^1^H NMR (400 MHz, CDCl_3_) δ 5.94 (ddt, *J* = 17.2, 10.4, 5.9 Hz, 1H), 5.68–5.55 (m, 1H), 5.39 (dq, *J* = 17.2, 1.5 Hz, 1H), 5.28 (dq, *J* = 10.4, 1.2 Hz, 1H), 5.25–5.14 (m, 2H), 4.72 (tt, *J* = 6.0, 1.4 Hz, 2H), 3.27–3.16 (m, 1H), 3.18–3.03 (m, 2H), 2.82–2.71 (m, 1H), 2.43 (ddt, *J* = 14.1, 7.9, 0.9 Hz, 1H), 2.34–2.18 (m, 1H), 2.16–2.00 (m, 2H) ppm. ^13^C{^1^H} NMR (100 MHz, CDCl_3_): δ 167.0, 131.2, 131.1, 120.6, 119.3, 70.2, 67.1, 51.3, 37.1, 30.8, 18.3 ppm. IR: ν_max_ (neat) 3082, 2952, 1733 cm^–1^. HRMS (APCI) *m/z*: calcd for C_11_H_17_O_4_S [M + H]^+^ 245.0842, found 245.0846. HPLC: 85% ee (Chiralpak AD-H, hexane:*i*-PrOH = 95:5, flow rate = 1 mL/min, 30.0 °C, λ = 235 nm) *t*_R_ = 11.0 min (minor), *t*_R_ = 11.6 min (major). [α]: −42.3 (*c* = 0.13, CHCl_3_).

#### (2*R*)-Isobutyl-2-allyl-1,1-dioxo-thiolane-2-carboxylate (**18g**)

A vial was charged with **15g** (47 mg, 0.15 mmol), [Pd_2_dba_3_] (6.9 mg, 7.5 μmol), **L4** (15.9 mg, 19.5 μmol), and 1,4-dioxane (1.5 mL). The mixture was stirred at room temperature for 15 h and then concentrated under reduced pressure. Purification by flash column chromatography [hexane:EtOAc 4:1] gave **18g** (24 mg, 62%) as a colorless oil. *R*_f_ = 0.66 [petrol:EtOAc 1:1]. ^1^H NMR (400 MHz, CDCl_3_) δ 5.62 (dddd, *J* = 16.6, 10.1, 8.0, 6.3 Hz, 1H), 5.24–5.15 (m, 2H), 4.04 (dd, *J* = 10.5, 6.4 Hz, 1H), 3.96 (dd, *J* = 10.5, 6.7 Hz, 1H), 3.25–3.16 (m, 1H), 3.16–3.04 (m, 2H), 2.80–2.72 (m, 1H), 2.43 (dd, *J* = 14.1, 8.0 Hz, 1H), 2.31–2.19 (m, 1H), 2.13–1.96 (m, 3H), 0.98 (d, *J* = 1.3 Hz, 3H), 0.96 (d, *J* = 1.3 Hz, 3H) ppm. ^13^C{^1^H} NMR (100 MHz, CDCl_3_): δ 167.3, 131.2, 120.4, 72.7, 70.3, 51.3, 37.1, 30.8, 27.6, 19.1, 19.0, 18.3 ppm. IR: ν_max_ (neat) 2961, 2875, 1733 cm^–1^. HRMS (APCI) *m/z*: calcd for C_12_H_21_O_4_S [M + H]^+^ 261.1155, found 261.1154. HPLC: 95% ee (Chiralpak AD-H, hexane:*i*-PrOH = 95:5, flow rate = 1 mL/min, 30.0 °C, λ = 210 nm) *t*_R_ = 8.7 min (major), *t*_R_ = 18.7 min (minor). [α]: −16.8 (*c* = 0.21, CHCl_3_).

#### (2*R*)-Methyl-2-allyl-tetrahydrothiophene-2-carboxylate-1,1-dioxide (**18h**)

A vial was charged with **15h** (77 mg, 0.29 mmol), [Pd_2_dba_3_] (6.7 mg, 7.3 μmol), **L4** (15.3 mg, 18.9 μmol), and 1,4-dioxane (2.9 mL). The mixture was stirred at room temperature for 2 h and then concentrated under reduced pressure. Purification by flash column chromatography [hexane:EtOAc 4:1] gave **18h** (58 mg, 92%) as a colorless oil. *R*_f_ = 0.32 [petrol:EtOAc 4:1]. ^1^H NMR (400 MHz, CDCl_3_) δ 5.61 (dddd, *J* = 17.0, 10.1, 7.9, 6.4 Hz, 1H), 5.24–5.15 (m, 2H), 3.84 (s, 3H), 3.25–3.17 (m, 1H), 3.14–3.04 (m, 2H), 2.81–2.72 (m, 1H), 2.42 (ddt, *J* = 14.2, 7.9, 1.1 Hz, 1H), 2.32–2.20 (m, 1H), 2.15–2.01 (m, 2H) ppm. ^13^C{^1^H} NMR (100 MHz, CDCl_3_): δ 167.8, 131.1, 120.5, 70.3, 53.4, 51.3, 37.1, 30.8, 18.4 ppm. IR: ν_max_ (neat) 3081, 3006, 2954, 1735 cm^–1^. HRMS (APCI) *m/z*: calcd for C_9_H_15_O_4_S [M + H]^+^ 219.0686, found 219.0680. HPLC: 69% ee (Chiralcel OD-H, hexane:*i*-PrOH = 95:5, flow rate = 1 mL/min, 30.0 °C, λ = 220 nm) *t*_R_ = 17.3 min (minor), *t*_R_ = 19.0 min (major). [α]: −56.7 (*c* = 0.30, CHCl_3_).

#### (2*R*)-Phenyl-2-(2-methylallyl)tetrahydrothiophene-2-carboxylate-1,1-dioxide (**18i**)

A vial was charged with **15i** (30 mg, 0.089 mmol), [Pd_2_dba_3_] (2.1 mg, 2.5 μmol), **L4** (4.7 mg, 5.8 μmol), and 1,4-dioxane (0.9 mL). The mixture was stirred at room temperature for 2 h and then concentrated under reduced pressure. Purification by flash column chromatography [hexane:EtOAc 19:1–4:1] gave **18i** (16 mg, 62%) as a colorless oil. *R*_f_ = 0.21 [petrol:EtOAc 4:1]. ^1^H NMR (300 MHz, CDCl_3_) δ 7.39 (t, *J* = 7.7 Hz, 2H), 7.25 (tt, *J* = 7.4, 1.2 Hz, 1H), 7.16 (d, *J* = 7.4 Hz, 2H), 4.99 (t, *J* = 1.7 Hz, 1H), 4.86 (t, *J* = 1.2 Hz, 1H), 3.34 (d, *J* = 15.4 Hz, 1H), 3.30–3.21 (m, 1H), 3.20–3.07 (m, 1H), 3.01–2.88 (m, 1H), 2.56 (d, *J* = 15.4 Hz, 1H), 2.42–2.02 (m, 3H), 1.83 (s, 3H) ppm. ^13^C{^1^H} NMR (75 MHz, CDCl_3_): δ 167.0, 150.7, 139.6, 129.5, 126.4, 121.3, 115.3, 70.0, 51.4, 40.4, 31.1, 23.3, 18.6 ppm. IR: ν_max_ (neat) 3075, 2948, 1752 cm^–1^. HRMS (APCI) *m/z*: calcd for C_15_H_19_O_4_S [M + H]^+^ 295.0999, found 295.0999. HPLC: 82% ee (Chiralcel OD-H, hexane:*i*-PrOH = 95:5, flow rate = 1 mL/min, 30.0 °C, λ = 220 nm) *t*_R_ = 18.9 min (major), *t*_R_ = 20.9 min (minor). [α]: −217 (*c* = 0.05, CHCl_3_).

#### (2*R*)-(*p*-Tolyl)-2-(2-methylallyl)tetrahydrothiophene-2-carboxylate-1,1-dioxide (**18j**)

A vial was charged with **15j** (30 mg, 0.085 mmol), [Pd_2_dba_3_] (2.1 mg, 2.1 μmol), **L4** (4.5 mg, 5.5 μmol), and 1,4-dioxane (0.9 mL). The mixture was stirred at room temperature for 2 h and then concentrated under reduced pressure. Purification by flash column chromatography [hexane:EtOAc 19:1–4:1] gave **18j** (16 mg, 60%) as a colorless oil. *R*_f_ = 0.25 [petrol:EtOAc 4:1]. ^1^H NMR (300 MHz, CDCl_3_) δ 7.17 (d, *J* = 8.8 Hz, 2H), 7.03 (d, *J* = 8.6 Hz, 2H), 4.98 (dq, *J* = 2.6, 1.4 Hz, 1H), 4.85 (quint, *J* = 1.2 Hz, 1H), 3.32 (d, *J* = 14.5 Hz, 1H), 3.29–3.20 (m, 1H), 3.19–3.07 (m, 1H), 2.99–2.86 (m, 1H), 2.55 (dd, *J* = 15.3, 1.0 Hz, 1H), 2.34 (s, 3H), 2.33–2.24 (m, 1H), 2.24–2.05 (m, 2H), 1.81 (dt, *J* = 1.5, 0.7 Hz, 3H) ppm. ^13^C{^1^H} NMR (75 MHz, CDCl_3_): δ 167.1, 148.5, 139.7, 136.0, 130.0, 121.0, 115.3, 70.0, 51.4, 40.3, 31.0, 23.3, 20.9, 18.6 ppm. IR: ν_max_ (neat) 3075, 3032, 2948, 1750 cm^–1^. HRMS (APCI) *m/z*: calcd for C_16_H_21_O_4_S [M + H]^+^ 309.1155, found 309.1145. HPLC: 87% ee (Chiralcel OD-H, hexane:*i*-PrOH = 95:5, flow rate = 1 mL/min, 30.0 °C, λ = 220 nm) *t*_R_ = 18.6 min (major), *t*_R_ = 21.6 min (minor). [α]: −100 (*c* = 0.10, CHCl_3_).

#### (2*R*)-(2-Allyl-1,1-dioxidotetrahydrothiophen-2-yl)(phenyl)methanone (**18k**)

A vial was charged with **15k** (46 mg, 0.15 mmol), [Pd_2_dba_3_] (6.9 mg, 7.5 μmol), **L4** (15.9 mg, 19.5 μmol), and 1,4-dioxane (1.5 mL). The mixture was stirred at room temperature for 2 h and then concentrated under reduced pressure. Purification by flash column chromatography [hexane:EtOAc 4:1] gave **18k** (31 mg, 78%) as a colorless oil. *R*_f_ = 0.27 [petrol:EtOAc 4:1]. ^1^H NMR (300 MHz, CDCl_3_) δ 8.00 (d, *J* = 7.0 Hz, 2H), 7.56 (tt, *J* = 7.3, 1.4 Hz, 1H), 7.47 (t, *J* = 7.3 Hz, 2H), 5.32 (dddd, *J* = 16.7, 10.1, 8.0, 6.5 Hz, 1H), 5.00 (ddt, *J* = 10.1, 1.8, 1.0 Hz, 1H), 4.88 (dq, *J* = 16.9, 1.5 Hz, 1H), 3.37–3.03 (m, 4H), 2.64 (ddt, *J* = 14.6, 7.9, 1.1 Hz, 1H), 2.35–2.01 (m, 3H) ppm. ^13^C{^1^H} NMR (75 MHz, CDCl_3_): δ 193.9, 136.0, 132.9, 130.1, 129.4, 128.4, 120.7, 73.9, 53.7, 39.4, 32.5, 18.7 ppm. IR: ν_max_ (neat) 3066, 2950, 1675 cm^–1^. HRMS (APCI) *m/z*: calcd for C_14_H_17_O_3_S [M + H]^+^ 265.0893, found 265.0885. HPLC: 72% ee (Chiralcel OD-H, hexane:*i*-PrOH = 90:10, flow rate = 1 mL/min, 30.0 °C, λ = 254 nm) *t*_R_ = 13.4 min (minor), *t*_R_ = 18.0 min (major). [α]: −44.9 (*c* = 0.35, CHCl_3_).

#### (2*R*)-(2-Allyl-1,1-dioxidotetrahydrothiophen-2-yl)(*p*-tolyl)methanone (**18l**)

A vial was charged with **15l** (30 mg, 0.093 mmol), [Pd_2_dba_3_] (4.6 mg, 5.0 μmol), **L4** (9.8 mg, 12.0 μmol), and 1,4-dioxane (0.9 mL). The mixture was stirred at room temperature for 2 h and then concentrated under reduced pressure. Purification by flash column chromatography [hexane:EtOAc 4:1] gave **18l** (23 mg, 89%) as a colorless solid. *R*_f_ = 0.22 [petrol:EtOAc 4:1]. m.p.: 81–82 °C. ^1^H NMR (300 MHz, CDCl_3_) δ 7.93 (d, *J* = 8.5 Hz, 2H), 7.26 (d, *J* = 7.9 Hz, 2H), 5.33 (dddd, *J* = 16.6, 10.1, 8.1, 6.4 Hz, 1H), 5.01 (ddt, *J* = 10.1, 1.8, 1.0 Hz, 1H), 4.90 (dq, *J* = 16.8, 1.4 Hz, 1H), 3.38–3.02 (m, 4H), 2.63 (ddt, *J* = 14.6, 8.1, 1.1 Hz, 1H), 2.40 (s, 3H), 2.33–2.00 (m, 3H) ppm. ^13^C{^1^H} NMR (75 MHz, CDCl_3_): δ 193.1, 143.9, 133.3, 130.2, 129.6, 129.1, 120.6, 73.9, 53.8, 39.5, 32.5, 21.6, 18.7 ppm. IR: ν_max_ (neat) 3066, 2954, 2926, 2854, 1675 cm^–1^. HRMS (APCI) *m/z*: calcd for C_15_H_19_O_3_S [M + H]^+^ 279.1049, found 279.1041. HPLC: 62% ee (Chiralcel OD-H, hexane:*i*-PrOH = 95:5, flow rate = 1 mL/min, 30.0 °C, λ = 220 nm) *t*_R_ = 21.2 min (minor), *t*_R_ = 33.4 min (major). [α]: −68.8 (*c* = 0.11, CHCl_3_).

#### (2*R*)-(2-Allyl-1,1-dioxo-thiolan-2-yl)-(2-furyl)methanone (**18m**)

A vial was charged with a 1:5.2 mixture of **24**:**15m** (51 mg, corresponding to 44.5 mg of pure **15m**, 0.15 mmol), [Pd_2_dba_3_] (6.9 mg, 7.5 μmol), **L4** (15.9 mg, 19.5 μmol), and 1,4-dioxane (1.5 mL). The mixture was stirred at room temperature for 2 h and then concentrated under reduced pressure. Purification by flash column chromatography [hexane:EtOAc 5:1] gave **18m** (32 mg, 84%) as a yellow oil. *R*_f_ = 0.33 [petrol:EtOAc 2:1]. ^1^H NMR (400 MHz, CDCl_3_) δ 7.67 (dd, *J* = 1.7, 0.7 Hz, 1H), 7.42 (dd, *J* = 3.7, 0.8 Hz, 1H), 6.57 (dd, *J* = 3.7, 1.7 Hz, 1H), 5.46 (dddd, *J* = 17.0, 10.1, 7.7, 6.9 Hz, 1H), 5.07 (dq, *J* = 10.1, 1.2 Hz, 1H), 5.02 (dq, *J* = 16.7, 1.4 Hz, 1H), 3.56 (dd, *J* = 14.5, 6.9 Hz, 1H), 3.26–3.11 (m, 2H), 3.14–3.02 (m, 1H), 2.63 (ddt, *J* = 14.5, 7.7, 1.2 Hz, 1H), 2.24–2.06 (m, 2H), 2.09–1.97 (m, 1H) ppm. ^13^C{^1^H} NMR (100 MHz, CDCl_3_): δ 180.9, 151.9, 146.9, 130.5, 120.5, 120.0, 112.5, 73.1, 53.6, 38.6, 31.1, 18.4 ppm. IR: ν_max_ (neat) 2953, 1662 cm^–1^. HRMS (APCI) *m/z*: calcd for C_12_H_15_O_4_S [M + H]^+^ 255.0686, found 255.0680. HPLC: 68% ee (Chiralpak AD-H, hexane:*i*-PrOH = 95:5, flow rate = 1 mL/min, 30.0 °C, λ = 220 nm) *t*_R_ = 22.8 min (minor), *t*_R_ = 26.8 min (major). [α]: −59.4 (*c* = 0.24, CHCl_3_).

#### (2*R*)-(2-Allyl-1,1-dioxo-thiolan-2-yl)cyclohexylmethanone (**18n**)

A vial was charged with **15n** (47 mg, 0.15 mmol), [Pd_2_dba_3_] (6.9 mg, 7.5 μmol), **L4** (15.9 mg, 19.5 μmol), and 1,4-dioxane (1.5 mL). The mixture was stirred at room temperature for 2 h and then concentrated under reduced pressure. Purification by flash column chromatography [hexane:EtOAc 5:1] gave **18n** (37 mg, 91%) as a colorless solid. *R*_f_ = 0.61 [petrol:EtOAc 2:1]. m.p.: 60–62 °C. ^1^H NMR (400 MHz, CDCl_3_) δ 5.48 (dddd, *J* = 17.3, 9.8, 8.5, 5.3 Hz, 1H), 5.22–5.15 (m, 2H), 3.17–3.03 (m, 3H), 2.89 (tt, *J* = 11.3, 3.1 Hz, 1H), 2.78–2.68 (m, 1H), 2.62 (ddt, *J* = 15.1, 8.5, 1.0 Hz, 1H), 2.17–2.07 (m, 1H), 2.07–1.94 (m, 3H), 1.83–1.72 (m, 3H), 1.72–1.64 (m, 1H), 1.45–1.22 (m, 5H) ppm. ^13^C{^1^H} NMR (100 MHz, CDCl_3_): δ 206.6, 130.8, 120.6, 74.9, 51.6, 48.5, 36.2, 30.4, 29.1, 28.6, 25.7, 25.6, 25.4, 17.5 ppm. IR: ν_max_ (neat) 2929, 2853, 1701 cm^–1^. HRMS (APCI) *m/z*: calcd for C_14_H_23_O_3_S [M + H]^+^ 271.1362, found 271.1373. HPLC: 89% ee (Chiralcel OD-H, hexane:*i*-PrOH = 95:5, flow rate = 1 mL/min, 30.0 °C, λ = 225 nm) *t*_R_ = 9.0 min (minor), *t*_R_ = 9.9 min (major). [α]: −90.2 (*c* = 0.28, CHCl_3_).

#### (2*R*)-(2-Allyl-1,1-dioxo-thiolan-2-yl)-tetrahydropyran-4-yl-methanone (**18o**)

A vial was charged with a 1:3.4 mixture of **24**:**15o** (56.5 mg, corresponding to 47.5 mg of pure **15o**, 0.15 mmol), [Pd_2_dba_3_] (6.9 mg, 7.5 μmol), **L4** (15.9 mg, 19.5 μmol), and 1,4-dioxane (1.5 mL). The mixture was stirred at room temperature for 18 h and then concentrated under reduced pressure. Purification by flash column chromatography [hexane:EtOAc 3:1] gave **18o** (41 mg, quant.) as a colorless solid. *R*_f_ = 0.41 [petrol:EtOAc 1:1]. m.p.: 54–57 °C. ^1^H NMR (400 MHz, CDCl_3_) δ 5.52–5.39 (m, 1H), 5.22 (dt, *J* = 1.6, 1.0 Hz, 1H), 5.18 (dq, *J* = 6.6, 1.4 Hz, 1H), 4.03–3.94 (m, 2H), 3.49–3.39 (m, 2H), 3.20–3.03 (m, 4H), 2.76 (dt, *J* = 13.8, 6.9 Hz, 1H), 2.66 (ddt, *J* = 15.3, 8.2, 1.0 Hz, 1H), 2.16–1.91 (m, 3H), 1.89–1.69 (m, 3H), 1.64 (ddd, *J* = 13.3, 3.8, 2.0 Hz, 1H) ppm. ^13^C{^1^H} NMR (100 MHz, CDCl_3_): δ 204.9, 130.4, 120.9, 75.0, 67.1, 66.8, 51.9, 45.6, 36.3, 30.2, 28.7, 28.7, 17.6 ppm. IR: ν_max_ (neat) 2957, 2931, 1709 cm^–1^. HRMS (APCI) *m/z*: calcd for C_13_H_21_O_4_S [M + H]^+^ 273.1155, found 273.1144. HPLC: 91% ee (Chiralpak AD-H, hexane:*i*-PrOH = 95:5, flow rate = 1 mL/min, 30.0 °C, λ = 200 nm) *t*_R_ = 19.7 min (minor), *t*_R_ = 20.8 min (major). [α]: −77.0 (*c* = 0.34, CHCl_3_).

#### (2*R*)-(2-Allyl-1,1-dioxo-thiolan-2-yl)cyclopropylmethanone (**18p**)

A vial was charged with **15p** (41 mg, 0.15 mmol), [Pd_2_dba_3_] (6.9 mg, 7.5 μmol), **L4** (15.9 mg, 19.5 μmol), and 1,4-dioxane (1.5 mL). The mixture was stirred at room temperature for 2 h and then concentrated under reduced pressure. Purification by flash column chromatography [hexane:EtOAc 5:1] gave **18p** (34 mg, 99%) as a colorless oil. *R*_f_ = 0.66 [petrol:EtOAc 2:1]. ^1^H NMR (400 MHz, CDCl_3_) δ 5.53 (dddd, *J* = 16.7, 10.0, 7.8, 6.5 Hz, 1H), 5.25–5.15 (m, 2H), 3.24 (ddd, *J* = 14.7, 6.6, 1.5 Hz, 1H), 3.19–3.06 (m, 2H), 2.79 (dt, *J* = 13.9, 7.0 Hz, 1H), 2.62 (ddt, *J* = 14.7, 7.9, 1.0 Hz, 1H), 2.32 (tt, *J* = 7.7, 4.5 Hz, 1H), 2.19–1.98 (m, 2H), 1.92 (dt, *J* = 13.9, 7.0 Hz, 1H), 1.31–1.23 (m, 1H), 1.09–0.97 (m, 3H) ppm. ^13^C{^1^H} NMR (100 MHz, CDCl_3_): δ 202.7, 130.5, 120.6, 74.5, 51.6, 37.3, 28.9, 19.8, 17.7, 13.9, 12.3 ppm. IR: ν_max_ (neat) 2952, 1697 cm^–1^. HRMS (APCI) *m/z*: calcd for C_11_H_17_O_3_S [M + H]^+^ 229.0893, found 229.0890. HPLC: 87% ee (Chiralpak AD-H, hexane:*i*-PrOH = 95:5, flow rate = 1 mL/min, 30.0 °C, λ = 215 nm) *t*_R_ = 12.0 min (minor), *t*_R_ = 14.0 min (major). [α]: −51.0 (*c* = 0.27, CHCl_3_).

#### (2*R*)-2-(2-Methylpropanoyl)-2-(prop-2-en-1-yl)thiolane-1,1-dione (**18q**)

A vial was charged with **15q** (50 mg, 0.18 mmol), [Pd_2_dba_3_] (8.2 mg, 9.1 μmol), **L4** (19.0 mg, 23.0 μmol), and 1,4-dioxane (1.8 mL). The mixture was stirred at room temperature for 2 h and then concentrated under reduced pressure. Purification by flash column chromatography [hexane:EtOAc 4:1] gave **18q** (40 mg, 97%) as a yellow oil. *R*_f_ = 0.29 [petrol:EtOAc 4:1]. ^1^H NMR (400 MHz, CDCl_3_) δ 5.46 (dddd, *J* = 17.2, 9.9, 8.4, 5.6 Hz, 1H), 5.21–5.17 (m, 1H), 5.17–5.12 (m, 1H), 3.19–3.02 (m, 4H), 2.75–2.67 (m, 1H), 2.59 (ddt, *J* = 15.0, 8.4, 1.1 Hz, 1H), 2.16–1.94 (m, 3H), 1.13 (d, *J* = 6.6 Hz, 3H), 1.10 (d, *J* = 6.6 Hz, 3H) ppm. ^13^C{^1^H} NMR (100 MHz, CDCl_3_): δ 207.8, 130.6, 120.6, 74.8, 51.5, 38.0, 36.2, 28.5, 20.3, 19.6, 17.5 ppm. IR: ν_max_ (neat) 3083, 2976, 2939, 2876, 1709 cm^–1^. HRMS (APCI) *m/z*: calcd for C_11_H_19_O_3_S [M + H]^+^ 231.1049, found 231.1042. HPLC: 88% ee (Chiralcel OD-H, hexane:*i*-PrOH = 95:5, flow rate = 1 mL/min, 30.0 °C, λ = 210 nm) *t*_R_ = 10.5 min (minor), *t*_R_ = 12.6 min (major). [α]: −173 (*c* = 0.18, CHCl_3_).

#### (2*R*)-1-(2-Allyl-1,1-dioxo-thiolan-2-yl)-2,2-dimethylpropan-1-one (**18r**)

A vial was charged with **15r** (43 mg, 0.15 mmol), [Pd_2_dba_3_] (6.9 mg, 7.5 μmol), **L4** (15.9 mg, 19.5 μmol), and 1,4-dioxane (1.5 mL). The mixture was stirred at room temperature for 18 h and then concentrated under reduced pressure. Purification by flash column chromatography [hexane:EtOAc 5:1] gave **18r** (18 mg, 49%) as a colorless oil. *R*_f_ = 0.52 [petrol:EtOAc 2:1]. ^1^H NMR (400 MHz, CDCl_3_) δ 5.51 (dddd, *J* = 17.3, 9.8, 8.0, 6.1 Hz, 1H), 5.19 (ddq, *J* = 14.7, 3.1, 1.6 Hz, 2H), 3.28 (ddq, *J* = 15.1, 6.1, 1.3 Hz, 1H), 3.19–3.05 (m, 2H), 2.96–2.86 (m, 1H), 2.60 (ddt, *J* = 15.1, 8.0, 1.1 Hz, 1H), 2.16–1.98 (m, 3H), 1.31 (s, 9H) ppm. ^13^C{^1^H} NMR (100 MHz, CDCl_3_): δ 207.8, 131.2, 120.6, 76.0, 52.7, 46.3, 37.8, 31.7, 28.3, 18.0 ppm. IR: ν_max_ (neat) 2972, 1686 cm^–1^. HRMS (APCI) *m/z*: calcd for C_12_H_21_O_3_S [M + H]^+^ 245.1206, found 245.1207. HPLC: 90% ee (Chiralcel OD-H, hexane:*i*-PrOH = 95:5, flow rate = 1 mL/min, 30.0 °C, λ = 220 nm) *t*_R_ = 11.4 min (minor), *t*_R_ = 12.0 min (major). [α]: −35.7 (*c* = 0.14, CHCl_3_).

#### (2*R*)-1-(2-Allyl-1,1-dioxo-thiolan-2-yl)-3-methyl-butan-1-one (**18s**)

A vial was charged with **15s** (43 mg, 0.15 mmol), [Pd_2_dba_3_] (6.9 mg, 7.5 μmol), **L4** (15.9 mg, 19.5 μmol), and 1,4-dioxane (1.5 mL). The mixture was stirred at room temperature for 2 h and then concentrated under reduced pressure. Purification by flash column chromatography [hexane:EtOAc 5:1] gave **18s** (25 mg, 68%) as a colorless oil. *R*_f_ = 0.47 [petrol:EtOAc 2:1]. ^1^H NMR (400 MHz, CDCl_3_) δ 5.57–5.44 (m, 1H), 5.23–5.16 (m, 2H), 3.17–3.01 (m, 3H), 2.79 (dt, *J* = 13.7, 7.0 Hz, 1H), 2.73 (dd, *J* = 18.4, 7.0 Hz, 1H), 2.57 (ddt, *J* = 15.1, 7.3, 1.2 Hz, 1H), 2.48 (dd, *J* = 18.4, 6.2 Hz, 1H), 2.26–2.12 (m, 2H), 2.04 (dtd, *J* = 13.5, 7.0, 1.1 Hz, 1H), 1.93 (dt, *J* = 13.8, 6.8 Hz, 1H), 0.94 (d, *J* = 2.2 Hz, 3H), 0.93 (d, *J* = 2.2 Hz, 3H) ppm. ^13^C{^1^H} NMR (100 MHz, CDCl_3_): δ 202.1, 130.4, 120.6, 73.8, 51.4, 48.9, 36.7, 29.2, 23.7, 22.7, 22.2, 17.7 ppm. IR: ν_max_ (neat) 2957, 2871, 1712 cm^–1^. HRMS (APCI) *m/z*: calcd for C_12_H_21_O_3_S [M + H]^+^ 245.1206, found 245.1207. HPLC: 78% ee (Chiralcel OD-H, hexane:*i*-PrOH = 95:5, flow rate = 1 mL/min, 30.0 °C, λ = 280 nm) *t*_R_ = 8.3 min (minor), *t*_R_ = 9.3 min (major). [α]: −61.9 (*c* = 0.21, CHCl_3_).

#### (2*R*)-1-(2-Allyl-1,1-dioxo-thiolan-2-yl)butan-1-one (**18t**)

A vial was charged with **15t** (41 mg, 0.15 mmol), [Pd_2_dba_3_] (6.9 mg, 7.5 μmol), **L4** (15.9 mg, 19.5 μmol), and 1,4-dioxane (1.5 mL). The mixture was stirred at room temperature for 2 h and then concentrated under reduced pressure. Purification by flash column chromatography [hexane:EtOAc 5:1] gave **18t** (33 mg, 96%) as a colorless oil. *R*_f_ = 0.55 [petrol:EtOAc 2:1]. ^1^H NMR (400 MHz, CDCl_3_) δ 5.50 (ddt, *J* = 17.0, 10.1, 7.0 Hz, 1H), 5.21 (dq, *J* = 11.1, 1.4 Hz, 1H), 5.17 (dq, *J* = 4.0, 1.4 Hz, 1H), 3.17–3.02 (m, 3H), 2.86–2.76 (m, 2H), 2.62–2.47 (m, 2H), 2.16 (ddq, *J* = 14.0, 8.1, 7.0 Hz, 1H), 2.05 (ddq, *J* = 13.6, 8.1, 6.9 Hz, 1H), 1.92 (dt, *J* = 13.9, 7.0 Hz, 1H), 1.64 (sext, *J* = 7.6 Hz, 2H), 0.93 (t, *J* = 7.4 Hz, 3H) ppm. ^13^C{^1^H} NMR (100 MHz, CDCl_3_): δ 202.7, 130.5, 120.6, 73.9, 51.5, 42.3, 36.8, 29.3, 17.8, 16.9, 13.5 ppm. IR: ν_max_ (neat) 2963, 2875, 1712 cm^–1^. HRMS (APCI) *m/z*: calcd for C_11_H_19_O_3_S [M + H]^+^ 231.1049, found 231.1040. HPLC: 74% ee (Chiralcel OD-H, hexane:*i*-PrOH = 95:5, flow rate = 1 mL/min, 30.0 °C, λ = 280 nm) *t*_R_ = 10.1 min (minor), *t*_R_ = 11.5 min (major). [α]: −59.0 (*c* = 0.25, CHCl_3_).

#### (2*R*)-(2-Allyl-1,1-dioxo-thiolan-2-yl)(methyl)methanone (**18u**)

A vial was charged with **15u** (55 mg, 0.22 mmol), [Pd_2_dba_3_] (10.0 mg, 11 μmol), **L4** (23.2 mg, 29 μmol), and 1,4-dioxane (2.2 mL). The mixture was stirred at room temperature for 2 h and then concentrated under reduced pressure. Purification by flash column chromatography [hexane:EtOAc 4:1] gave **18u** (40 mg, 90%) as a yellow oil. *R*_f_ = 0.21 [petrol:EtOAc 4:1]. ^1^H NMR (400 MHz, CDCl_3_) δ 5.57–5.43 (m, 1H), 5.24–5.14 (m, 2H), 3.17–2.99 (m, 3H), 2.79 (dtd, *J* = 13.8, 6.9, 1.6 Hz, 1H), 2.57 (ddq, *J* = 15.0, 7.1, 1.5 Hz, 1H), 2.36 (s, 3H), 2.21–1.98 (m, 2H), 1.90 (dt, *J* = 14.0, 7.1 Hz, 1H) ppm. ^13^C{^1^H} NMR (100 MHz, CDCl_3_): δ 200.5, 130.3, 120.6, 74.1, 51.5, 36.9, 29.2, 27.9, 17.8 ppm. IR: ν_max_ (neat) 3082, 2954, 1713 cm^–1^. HRMS (APCI) *m/z*: calcd for C_9_H_15_O_3_S [M + H]^+^ 203.0736, found 203.0737. HPLC: 21% ee (Chiralcel OD-H, hexane:*i*-PrOH = 95:5, flow rate = 1 mL/min, 30.0 °C, λ = 210 nm) *t*_R_ = 15.5 min (minor), *t*_R_ = 16.9 min (major). [α]: −23.2 (*c* = 0.35, CHCl_3_).

#### (2*R*)-Phenyl-2-allyl-1,1-dioxo-thiane-2-carboxylate (**19a**)

A vial was charged with **16a** (40 mg, 0.12 mmol), [Pd_2_dba_3_] (2.7 mg, 3.0 μmol), **L4** (6.3 mg, 7.8 μmol), and 1,4-dioxane (1.2 mL). The mixture was stirred at room temperature for 2 h and then concentrated under reduced pressure. Purification by flash column chromatography [hexane:EtOAc 4:1] gave **19a** (34 mg, 96%) as a yellow oil. *R*_f_ = 0.21 [petrol:EtOAc 4:1]. ^1^H NMR (300 MHz, CDCl_3_) δ 7.40 (t, *J* = 8.0 Hz, 2H), 7.27 (tt, *J* = 7.6, 1.2 Hz, 1H), 7.11 (d, *J* = 7.4 Hz, 2H), 5.95 (dddd, *J* = 16.9, 10.0, 8.4, 6.2 Hz, 1H), 5.37–5.22 (m, 2H), 3.51–3.39 (m, 1H), 3.31 (ddt, *J* = 14.3, 6.3, 1.4 Hz, 1H), 3.12 (dt, *J* = 14.0, 5.2 Hz, 1H), 2.75 (dd, *J* = 14.3, 8.4 Hz, 1H), 2.45 (ddd, *J* = 14.8, 6.4, 3.5 Hz, 1H), 2.22–2.05 (m, 3H), 1.97–1.68 (m, 2H) ppm. ^13^C{^1^H} NMR (75 MHz, CDCl_3_): δ 166.9, 150.2, 130.8, 129.6, 126.5, 121.3, 120.8, 71.0, 50.4, 35.6, 33.1, 24.0, 20.3 ppm. IR: ν_max_ (neat) 3079, 2935, 2856, 1750 cm^–1^. HRMS (APCI) *m/z*: calcd for C_15_H_19_O_4_S [M + H]^+^ 295.0999, found 295.0985. HPLC: 64% ee (Chiralpak AD-H, hexane:*i*-PrOH = 90:10, flow rate = 1 mL/min, 30.0 °C, λ = 220 nm) *t*_R_ = 11.8 min (major), *t*_R_ = 14.5 min (minor). [α]: −55.0 (*c* = 0.25, CHCl_3_).

#### (2*R*)-(4-Methoxyphenyl)-2-allyl-1,1-dioxo-thiane-2-carboxylate (**19b**)

A vial was charged with a 1:3.9 mixture of **25**:**16b** (63.5 mg, corresponding to 55 mg of pure **16b**, 0.15 mmol), [Pd_2_dba_3_] (3.5 mg, 3.75 μmol), **L4** (8.0 mg, 9.75 μmol), and 1,4-dioxane (1.5 mL). The mixture was stirred at room temperature for 15 h and then concentrated under reduced pressure. Purification by flash column chromatography [hexane:EtOAc 3:1] gave **19b** (37 mg, 77%) as a colorless oil. *R*_f_ = 0.25 [petrol:EtOAc 2:1]. ^1^H NMR (400 MHz, CDCl_3_) δ 7.02 (d, *J* = 9.2 Hz, 2H), 6.89 (d, *J* = 9.1 Hz, 2H), 5.98–5.86 (m, 1H), 5.33–5.21 (m, 2H), 3.79 (s, 3H), 3.47–3.37 (m, 1H), 3.28 (ddt, *J* = 14.3, 6.2, 1.3 Hz, 1H), 3.14–3.06 (m, 1H), 2.73 (dd, *J* = 14.3, 8.4 Hz, 1H), 2.47–2.37 (m, 1H), 2.19–2.05 (m, 3H), 1.93–1.78 (m, 1H), 1.78–1.68 (m, 1H) ppm. ^13^C{^1^H} NMR (100 MHz, CDCl_3_): δ 167.1, 157.7, 143.6, 130.8, 122.0, 120.6, 114.5, 70.9, 55.6, 50.3, 35.5, 32.9, 23.9, 20.2 ppm. IR: ν_max_ (neat) 2935, 1750, 1504 cm^–1^. HRMS (ESI) *m/z*: calcd for C_15_H_20_NaO_5_S [M + Na]^+^ 347.0924, found 347.0922. HPLC: 74% ee (Chiralpak AD-H, hexane:*i*-PrOH = 95:5, flow rate = 1 mL/min, 30.0 °C, λ = 220 nm) *t*_R_ = 34.0 min (major), *t*_R_ = 57.3 min (minor). [α]: −41.6 (*c* = 0.30, CHCl_3_).

#### (2*R*)-Benzyl-2-allyl-1,1-dioxo-thiane-2-carboxylate (**19c**)

A vial was charged with **16c** (53 mg, 0.15 mmol), [Pd_2_dba_3_] (6.9 mg, 7.5 μmol), **L4** (15.9 mg, 19.5 μmol), and 1,4-dioxane (1.5 mL). The mixture was stirred at room temperature for 24 h and then concentrated under reduced pressure. Purification by flash column chromatography [hexane:EtOAc 4:1] gave **19c** (30.5 mg, 66%) as a colorless oil. *R*_f_ = 0.41 [petrol:EtOAc 2:1]. ^1^H NMR (400 MHz, CDCl_3_) δ 7.44–7.28 (m, 5H), 5.72 (dddd, *J* = 16.5, 10.1, 8.4, 6.2 Hz, 1H), 5.28 (d, *J* = 12.2 Hz, 1H), 5.23 (d, *J* = 12.2 Hz, 1H), 5.12 (dq, *J* = 9.4, 1.4 Hz, 1H), 5.10–5.05 (m, 1H), 3.36 (ddd, *J* = 14.4, 7.8, 5.8 Hz, 1H), 3.12 (ddt, *J* = 14.2, 6.3, 1.4 Hz, 1H), 3.04 (dt, *J* = 14.2, 5.4 Hz, 1H), 2.62 (dd, *J* = 14.2, 8.4 Hz, 1H), 2.32–2.23 (m, 1H), 2.09–2.00 (m, 3H), 1.74–1.57 (m, 2H) ppm. ^13^C{^1^H} NMR (100 MHz, CDCl_3_): δ 167.7, 134.8, 130.8, 128.6, 128.5, 128.4, 120.2, 70.9, 67.9, 50.3, 35.5, 32.9, 23.9, 20.2 ppm. IR: ν_max_ (neat) 2924, 2853, 1731 cm^–1^. HRMS (ESI) *m/z*: calcd for C_16_H_21_O_4_S [M + H]^+^ 309.1155, found 309.1141. HPLC: 60% ee (Chiralpak AD-H, hexane:*i*-PrOH = 90:10, flow rate = 1 mL/min, 30.0 °C, λ = 220 nm) *t*_R_ = 13.8 min (major), *t*_R_ = 21.6 min (minor). [α]: −17.7 (*c* = 0.23, CHCl_3_).

#### (2*R*)-(2-Allyl-1,1-dioxo-thian-2-yl)phenylmethanone (**19d**)

A vial was charged with **16d** (48 mg, 0.15 mmol), [Pd_2_dba_3_] (6.9 mg, 7.5 μmol), **L4** (15.9 mg, 19.5 μmol), and 1,4-dioxane (1.5 mL). The mixture was stirred at room temperature for 24 h and then concentrated under reduced pressure. Purification by flash column chromatography [hexane:EtOAc 7:1] gave **19d** (13 mg, 31%) as a colorless oil. *R*_f_ = 0.38 [petrol:EtOAc 2:1]. ^1^H NMR (400 MHz, CDCl_3_) δ 7.86 (dd, *J* = 8.5, 1.3 Hz, 2H), 7.53 (tt, *J* = 7.6, 1.2 Hz, 1H), 7.43 (t, *J* = 7.5 Hz, 2H), 5.54 (dddd, *J* = 17.0, 10.2, 8.4, 6.2 Hz, 1H), 5.02 (dq, *J* = 10.3, 1.3 Hz, 1H), 4.94 (dq, *J* = 16.9, 1.5 Hz, 1H), 3.48 (ddt, *J* = 15.1, 6.2, 1.5 Hz, 1H), 3.47–3.35 (m, 1H), 3.16 (dt, *J* = 14.3, 6.2 Hz, 1H), 2.84 (ddt, *J* = 15.1, 8.4, 1.1 Hz, 1H), 2.59 (ddd, *J* = 15.0, 8.2, 3.4 Hz, 1H), 2.18 (ddd, *J* = 14.7, 9.1, 3.2 Hz, 2H), 2.11 (quint, *J* = 6.0 Hz, 2H), 1.85–1.71 (m, 1H), 1.72–1.62 (m, 1H) ppm. ^13^C{^1^H} NMR (100 MHz, CDCl_3_): δ 198.1, 138.2, 132.1, 130.3, 128.3, 128.3, 120.5, 76.0, 51.0, 36.0, 33.1, 24.0, 20.1 ppm. IR: ν_max_ (neat) 3069, 2935, 1671 cm^–1^. HRMS (ESI) *m/z*: calcd for C_15_H_19_O_3_S [M + H]^+^ 279.1049, found 279.1049. HPLC: 77% ee (Chiralcel OD-H, hexane:*i*-PrOH = 95:5, flow rate = 1 mL/min, 30.0 °C, λ = 240 nm) *t*_R_ = 25.9 min (major), *t*_R_ = 28.1 min (minor). [α]: −13.7 (*c* = 0.11, CHCl_3_).

#### (2*R*)-(2-Allyl-1,1-dioxo-thian-2-yl)-(4-bromophenyl)methanone (**19e**)

A vial was charged with **16e** (60 mg, 0.15 mmol), [Pd_2_dba_3_] (6.9 mg, 7.5 μmol), **L4** (15.9 mg, 19.5 μmol), and 1,4-dioxane (1.5 mL). The mixture was stirred at room temperature for 15 h and then concentrated under reduced pressure. Purification by flash column chromatography [hexane:EtOAc 6:1] gave **19e** (44 mg, 82%) as a colorless oil. *R*_f_ = 0.38 [petrol:EtOAc 2:1]. ^1^H NMR (400 MHz, CDCl_3_) δ 7.79 (d, *J* = 8.8 Hz, 2H), 7.58 (d, *J* = 8.8 Hz, 2H), 5.47 (dddd, *J* = 17.0, 10.2, 8.3, 6.2 Hz, 1H), 5.05 (dq, *J* = 10.2, 1.3 Hz, 1H), 4.97 (dq, *J* = 16.9, 1.4 Hz, 1H), 3.45 (ddt, *J* = 15.0, 6.2, 1.6 Hz, 1H), 3.32 (dt, *J* = 14.3, 6.4 Hz, 1H), 3.17 (ddd, *J* = 14.4, 7.1, 6.0 Hz, 1H), 2.81 (ddt, *J* = 15.0, 8.3, 1.1 Hz, 1H), 2.56 (ddd, *J* = 15.0, 8.9, 3.4 Hz, 1H), 2.18–2.08 (m, 3H), 1.86–1.76 (m, 1H), 1.70–1.60 (m, 1H) ppm. ^13^C{^1^H} NMR (100 MHz, CDCl_3_): δ 196.8, 136.8, 131.6, 130.2, 129.9, 127.3, 120.9, 75.8, 50.9, 35.8, 32.6, 23.9, 20.0 ppm. IR: ν_max_ (neat) 2935, 2864, 1675 cm^–1^. HRMS (APCI) *m/z*: calcd for C_15_H_16_^79^BrO_3_S [M – H]^−^ 355.0009, found 355.0014. HPLC: 76% ee (Chiralpak AD-H, hexane:*i*-PrOH = 95:5, flow rate = 1 mL/min, 30.0 °C, λ = 254 nm) *t*_R_ = 27.5 min (major), *t*_R_ = 37.6 min (minor). [α]: −10.8 (*c* = 0.37, CHCl_3_).

#### (2*R*)-(2-Allyl-1,1-dioxo-thian-2-yl)-(4-fluorophenyl)methanone (**19f**)

A vial was charged with **16f** (51 mg, 0.15 mmol), [Pd_2_dba_3_] (6.9 mg, 7.5 μmol), **L4** (15.9 mg, 19.5 μmol), and 1,4-dioxane (1.5 mL). The mixture was stirred at room temperature for 15 h and then concentrated under reduced pressure. Purification by flash column chromatography [hexane:EtOAc 6:1] gave **19f** (33 mg, 74%) as a colorless oil. *R*_f_ = 0.35 [petrol:EtOAc 2:1]. ^1^H NMR (400 MHz, CDCl_3_) δ 7.98 (dd, *J* = 9.0, 5.3 Hz, 2H), 7.11 (dd, *J* = 9.0, 8.3 Hz, 2H), 5.48 (dddd, *J* = 16.9, 10.1, 8.3, 6.1 Hz, 1H), 5.04 (dq, *J* = 10.2, 1.3 Hz, 1H), 4.95 (dq, *J* = 16.9, 1.5 Hz, 1H), 3.47 (ddt, *J* = 15.1, 6.3, 1.7 Hz, 1H), 3.34 (dt, *J* = 14.2, 6.4 Hz, 1H), 3.17 (dt, *J* = 14.2, 6.6 Hz, 1H), 2.83 (ddt, *J* = 15.0, 8.3, 1.1 Hz, 1H), 2.59 (ddd, *J* = 15.0, 8.8, 3.4 Hz, 1H), 2.21–2.08 (m, 3H), 1.87–1.76 (m, 1H), 1.71–1.60 (m, 1H) ppm. ^13^C{^1^H} NMR (100 MHz, CDCl_3_): δ 195.9, 165.0 (d, *J* = 254.9 Hz), 134.1 (d, *J* = 3.3 Hz), 131.5 (d, *J* = 36.0 Hz), 130.0, 120.8, 115.5 (d, *J* = 21.8 Hz), 75.9, 50.9, 35.9, 32.8, 23.9, 20.0 ppm. ^19^F{^1^H} NMR (376 MHz, CDCl_3_): δ −105.4 ppm. IR: ν_max_ (neat) 3078, 2935, 1673 cm^–1^. HRMS (APCI) *m/z*: calcd for C_15_H_18_FO_3_S [M + H]^+^ 297.0955, found 297.0947. HPLC: 80% ee (Chiralcel OD-H, hexane:*i*-PrOH = 95:5, flow rate = 1 mL/min, 30.0 °C, λ = 254 nm) *t*_R_ = 19.0 min (major), *t*_R_ = 22.3 min (minor). [α]: −11.0 (*c* = 0.25, CHCl_3_).

#### (2*R*)-(2-Allyl-1,1-dioxo-thian-2-yl)-(*p*-tolyl)methanone (**19g**)

A vial was charged with **16g** (50.5 mg, 0.15 mmol), [Pd_2_dba_3_] (6.9 mg, 7.5 μmol), **L4** (15.9 mg, 19.5 μmol), and 1,4-dioxane (1.5 mL). The mixture was stirred at room temperature for 15 h and then concentrated under reduced pressure. Purification by flash column chromatography [hexane:EtOAc 6:1] gave **19g** (19 mg, 43%) as a colorless solid. *R*_f_ = 0.51 [petrol:EtOAc 2:1]. m.p.: 70–71 °C. ^1^H NMR (400 MHz, CDCl_3_) δ 7.81 (d, *J* = 8.3 Hz, 2H), 7.23 (d, *J* = 7.9 Hz, 2H), 5.55 (dddd, *J* = 16.9, 10.2, 8.4, 6.1 Hz, 1H), 5.01 (dt, *J* = 10.4, 1.2 Hz, 1H), 4.95 (dq, *J* = 17.0, 1.5 Hz, 1H), 3.52–3.38 (m, 2H), 3.15 (ddd, *J* = 14.1, 6.7, 5.3 Hz, 1H), 2.85 (ddt, *J* = 15.3, 8.4, 1.1 Hz, 1H), 2.60 (ddd, *J* = 15.0, 7.8, 3.5 Hz, 1H), 2.40 (s, 3H), 2.18 (ddd, *J* = 15.0, 9.5, 3.4 Hz, 1H), 2.13–2.06 (m, 2H), 1.80–1.62 (m, 2H) ppm. ^13^C{^1^H} NMR (100 MHz, CDCl_3_): δ 197.1, 143.2, 135.1, 130.5, 129.0, 128.8, 120.3, 76.2, 51.1, 36.0, 33.3, 24.0, 21.5, 20.2 ppm. IR: ν_max_ (neat) 2926, 1656 cm^–1^. HRMS (APCI) *m/z*: calcd for C_16_H_21_O_3_S [M + H]^+^ 293.1206, found 293.1192. HPLC: 80% ee (Chiralpak AD-H, hexane:*i*-PrOH = 95:5, flow rate = 1 mL/min, 30.0 °C, λ = 254 nm) *t*_R_ = 25.6 min (major), *t*_R_ = 31.3 min (minor). [α]: −20.6 (*c* = 0.17, CHCl_3_).

#### (2*R*)-(2-Allyl-1,1-dioxo-thian-2-yl)-(4-methoxyphenyl)methanone (**19h**)

A vial was charged with a 1:8.1 mixture of **25**:**16h** (57 mg, corresponding to 53 mg of pure **16h**, 0.15 mmol), [Pd_2_dba_3_] (6.9 mg, 7.5 μmol), **L4** (15.9 mg, 19.5 μmol), and 1,4-dioxane (1.5 mL). The mixture was stirred at room temperature for 15 h and then concentrated under reduced pressure. Purification by flash column chromatography [hexane:EtOAc 2:1] gave **19h** (23.5 mg, 51%) as a colorless oil. *R*_f_ = 0.55 [petrol:EtOAc 1:1]. ^1^H NMR (400 MHz, CDCl_3_) δ 8.00 (d, *J* = 9.1 Hz, 2H), 6.92 (d, *J* = 9.1 Hz, 2H), 5.55 (dddd, *J* = 17.0, 10.2, 8.4, 6.1 Hz, 1H), 5.00 (dq, *J* = 10.3, 1.3 Hz, 1H), 4.95 (dq, *J* = 16.9, 1.6 Hz, 1H), 3.87 (s, 3H), 3.50 (ddt, *J* = 15.3, 6.1, 1.5 Hz, 1H), 3.42 (ddd, *J* = 13.7, 7.9, 5.5 Hz, 1H), 3.14 (ddd, *J* = 14.2, 6.6, 4.9 Hz, 1H), 2.86 (ddt, *J* = 15.3, 8.5, 1.1 Hz, 1H), 2.62 (ddd, *J* = 15.0, 7.8, 3.4 Hz, 1H), 2.19 (ddd, *J* = 15.0, 9.5, 3.4 Hz, 1H), 2.14–2.04 (m, 2H), 1.81–1.60 (m, 2H) ppm. ^13^C{^1^H} NMR (100 MHz, CDCl_3_): δ 195.0, 163.1, 131.6, 130.6, 130.0, 120.2, 113.5, 76.3, 55.5, 51.1, 36.1, 33.4, 24.0, 20.2 ppm. IR: ν_max_ (neat) 2935, 1697 cm^–1^. HRMS (APCI) *m/z*: calcd for C_16_H_21_O_4_S [M + H]^+^ 309.1155, found 309.1145. HPLC: 84% ee (Chiralpak AD-H, hexane:*i*-PrOH = 95:5, flow rate = 1 mL/min, 30.0 °C, λ = 280 nm) *t*_R_ = 44.9 min (major), *t*_R_ = 54.2 min (minor). [α]: +20.0 (*c* = 0.20, CHCl_3_).

#### (2*R*)-(2-Allyl-1,1-dioxo-thian-2-yl)-(2-furyl)methanone (**19i**)

A vial was charged with a 1:6.8 mixture of **25**:**16i** (52 mg, corresponding to 47 mg of pure **16i**, 0.15 mmol), [Pd_2_dba_3_] (6.9 mg, 7.5 μmol), **L4** (15.9 mg, 19.5 μmol), and 1,4-dioxane (1.5 mL). The mixture was stirred at room temperature for 15 h and then concentrated under reduced pressure. Purification by flash column chromatography [hexane:EtOAc 3:1] gave **19i** (32 mg, 80%) as a colorless oil. *R*_f_ = 0.41 [petrol:EtOAc 2:1]. ^1^H NMR (400 MHz, CDCl_3_) δ 7.60 (dd, *J* = 1.7, 0.8 Hz, 1H), 7.43 (dd, *J* = 3.6, 0.8 Hz, 1H), 6.56 (dd, *J* = 3.7, 1.7 Hz, 1H), 5.77 (dddd, *J* = 16.9, 10.2, 9.1, 5.7 Hz, 1H), 4.97–4.86 (m, 2H), 3.56–3.40 (m, 2H), 3.10 (dt, *J* = 14.1, 4.7 Hz, 1H), 3.01 (dd, *J* = 15.3, 9.1 Hz, 1H), 2.66 (dddd, *J* = 15.0, 6.0, 3.3, 1.4 Hz, 1H), 2.19 (ddd, *J* = 15.0, 11.8, 3.3 Hz, 1H), 2.10–1.98 (m, 2H), 1.72–1.62 (m, 1H), 1.60–1.48 (m, 1H) ppm. ^13^C{^1^H} NMR (100 MHz, CDCl_3_): δ 184.0, 151.7, 146.2, 131.5, 120.8, 119.2, 112.6, 75.4, 51.2, 35.5, 33.3, 24.0, 20.5 ppm. IR: ν_max_ (neat) 2953, 1670 cm^–1^. HRMS (APCI) *m/z*: calcd for C_13_H_17_O_4_S [M + H]^+^ 269.0842, found 269.0829. HPLC: 90% ee (Chiralcel OD-H, hexane:*i*-PrOH = 95:5, flow rate = 1 mL/min, 30.0 °C, λ = 280 nm) *t*_R_ = 30.4 min (major), *t*_R_ = 37.0 min (minor). [α]: −4.1 (*c* = 0.25, CHCl_3_).

#### (2*R*)-1-(2-allyl-1,1-dioxo-thian-2-yl)-2-methylpropan-1-one (**19j**)

A vial was charged with **16j** (40 mg, 0.14 mmol), [Pd_2_dba_3_] (6.4 mg, 7.0 μmol), **L4** (14.8 mg, 18.2 μmol), and 1,4-dioxane (1.4 mL). The mixture was stirred at room temperature for 2 h and then concentrated under reduced pressure. Purification by flash column chromatography [hexane:EtOAc 4:1] gave **19j** (32 mg, 94%) as a yellow oil. *R*_f_ = 0.22 [petrol:EtOAc 4:1]. ^1^H NMR (300 MHz, CDCl_3_) δ 5.50 (dddd, *J* = 17.0, 9.9, 8.5, 5.6 Hz, 1H), 5.26–5.13 (m, 2H), 3.48 (hept, *J* = 6.7 Hz, 1H), 3.29–3.00 (m, 3H), 2.74 (ddt, *J* = 15.1, 8.5, 1.1 Hz, 1H), 2.28 (ddd, *J* = 14.2, 10.0, 3.5 Hz, 1H), 2.13–2.00 (m, 3H), 1.79–1.66 (m, 1H), 1.64–1.49 (m, 1H), 1.15 (d, *J* = 6.6 Hz, 3H), 1.12 (d, *J* = 6.7 Hz, 3H) ppm. ^13^C{^1^H} NMR (75 MHz, CDCl_3_): δ 209.3, 130.2, 120.4, 75.6, 50.8, 37.3, 34.5, 29.7, 24.0, 20.4, 20.2, 19.7 ppm. IR: ν_max_ (neat) 2973, 2937, 2874, 1709 cm^–1^. HRMS (APCI) *m/z*: calcd for C_12_H_21_O_3_S [M + H]^+^ 245.1206, found 245.1208. HPLC: 88% ee (Chiralpak AD-H, hexane:*i*-PrOH = 90:10, flow rate = 1 mL/min, 30.0 °C, λ = 210 nm) *t*_R_ = 13.3 min (major), *t*_R_ = 14.4 min (minor). [α]: −168 (*c* = 0.20, CHCl_3_).

#### (2*R*)-(2-Allyl-1,1-dioxo-thian-2-yl)cyclohexylmethanone (**19k**)

A vial was charged with **16k** (49 mg, 0.15 mmol), [Pd_2_dba_3_] (6.9 mg, 7.5 μmol), **L4** (15.9 mg, 19.5 μmol), and 1,4-dioxane (1.5 mL). The mixture was stirred at room temperature for 15 h and then concentrated under reduced pressure. Purification by flash column chromatography [hexane:EtOAc 7:1] gave **19k** (34 mg, 80%) as a colorless oil. *R*_f_ = 0.72 [petrol:EtOAc 4:1]. ^1^H NMR (400 MHz, CDCl_3_) δ 5.47 (dddd, *J* = 16.9, 10.0, 8.6, 5.5 Hz, 1H), 5.22–5.12 (m, 2H), 3.26–3.09 (m, 3H), 3.03 (dt, *J* = 14.3, 5.7 Hz, 1H), 2.72 (dd, *J* = 15.0, 8.6 Hz, 1H), 2.25 (ddd, *J* = 15.2, 9.9, 3.5 Hz, 1H), 2.09–1.98 (m, 3H), 1.98–1.88 (m, 1H), 1.79–1.63 (m, 5H), 1.60–1.49 (m, 1H), 1.44 (qd, *J* = 12.9, 3.4 Hz, 1H), 1.38–1.18 (m, 4H) ppm. ^13^C{^1^H} NMR (100 MHz, CDCl_3_): δ 207.9, 130.4, 120.3, 75.4, 50.9, 47.9, 34.6, 30.1, 29.8, 29.7, 25.6, 25.5, 25.4, 24.0, 19.7 ppm. IR: ν_max_ (neat) 2927, 2855, 1701 cm^–1^. HRMS (APCI) *m/z*: calcd for C_15_H_25_O_3_S [M + H]^+^ 285.1519, found 285.1516. HPLC: 89% ee (Chiralcel OD-H, hexane:*i*-PrOH = 95:5, flow rate = 1 mL/min, 30.0 °C, λ = 210 nm) *t*_R_ = 9.2 min (major), *t*_R_ = 10.7 min (minor). [α]: −62.5 (*c* = 0.29, CHCl_3_).

#### (2*R*)-(2-Allyl-1,1-dioxo-thian-2-yl)-tetrahydropyran-4-yl-methanone (**19l**)

A vial was charged with a 1:4.1 mixture of **25**:**16l** (58 mg, corresponding to 49.5 mg of pure **16l**, 0.15 mmol), [Pd_2_dba_3_] (6.9 mg, 7.5 μmol), **L4** (15.9 mg, 19.5 μmol), and 1,4-dioxane (1.5 mL). The mixture was stirred at room temperature for 15 h and then concentrated under reduced pressure. Purification by flash column chromatography [hexane:EtOAc 2:1] gave **19l** (39 mg, 91%) as a colorless solid. *R*_f_ = 0.18 [petrol:EtOAc 2:1]. m.p.: 69–70 °C. ^1^H NMR (400 MHz, CDCl_3_) δ 5.41 (dddd, *J* = 16.9, 9.9, 8.3, 5.7 Hz, 1H), 5.24–5.13 (m, 2H), 3.97 (dt, *J* = 4.2, 2.0 Hz, 1H), 3.95 (dt, *J* = 4.3, 2.2 Hz, 1H), 3.49 (tt, *J* = 11.7, 3.8 Hz, 1H), 3.42 (tt, *J* = 11.9, 2.5 Hz, 2H), 3.27–3.09 (m, 2H), 2.99 (dt, *J* = 14.3, 4.9 Hz, 1H), 2.75 (dd, *J* = 15.1, 8.3 Hz, 1H), 2.25 (ddd, *J* = 14.5, 10.8, 3.3 Hz, 1H), 2.10–1.99 (m, 3H), 1.86 (dd, *J* = 13.3, 4.4 Hz, 1H), 1.80 (dd, *J* = 12.5, 4.1 Hz, 1H), 1.77–1.64 (m, 2H), 1.60–1.48 (m, 2H) ppm. ^13^C{^1^H} NMR (100 MHz, CDCl_3_): δ 206.0, 129.9, 120.7, 75.5, 67.1, 66.8, 50.8, 45.0, 34.4, 29.7, 29.4, 29.3, 24.0, 19.6 ppm. IR: ν_max_ (neat) 2952, 2847, 1705 cm^–1^. HRMS (APCI) *m/z*: calcd for C_14_H_23_O_4_S [M + H]^+^ 287.1312, found 287.1306. HPLC: 91% ee (Chiralpak AD-H, hexane:*i*-PrOH = 95:5, flow rate = 1 mL/min, 30.0 °C, λ = 210 nm) *t*_R_ = 21.7 min (minor), *t*_R_ = 24.3 min (major). [α]: −73.0 (*c* = 0.34, CHCl_3_).

#### (2*R*)-1-(2-Allyl-1,1-dioxo-thian-2-yl)ethanone (**19n**)

A vial was charged with **16n** (40 mg, 0.15 mmol), [Pd_2_dba_3_] (6.9 mg, 7.5 μmol), **L4** (15.9 mg, 19.5 μmol), and 1,4-dioxane (1.5 mL). The mixture was stirred at room temperature for 2 h and then concentrated under reduced pressure. Purification by flash column chromatography [hexane:EtOAc 4:1] gave **19n** (30 mg, 93%) as a yellow oil. *R*_f_ = 0.20 [petrol:EtOAc 4:1]. ^1^H NMR (300 MHz, CDCl_3_) δ 5.54 (dddd, *J* = 16.5, 9.9, 7.9, 6.5 Hz, 1H), 5.28–5.14 (m, 2H), 3.23–2.99 (m, 3H), 2.69 (ddt, *J* = 14.7, 7.9, 1.1 Hz, 1H), 2.43 (s, 3H), 2.30 (ddd, *J* = 14.9, 9.4, 3.6 Hz, 1H), 2.11–1.98 (m, 3H), 1.85–1.71 (m, 1H), 1.67–1.52 (m, 1H) ppm. ^13^C{^1^H} NMR (75 MHz, CDCl_3_): δ 201.6, 129.7, 120.7, 75.1, 50.5, 34.9, 30.0, 28.8, 24.0, 19.9 ppm. IR: ν_max_ (neat) 2941, 2868, 1709 cm^–1^. HRMS (APCI) *m/z*: calcd for C_10_H_17_O_3_S [M + H]^+^ 217.0893, found 217.0884. HPLC: 32% ee (Chiralcel OD-H, hexane:*i*-PrOH = 95:5, flow rate = 1 mL/min, 30.0 °C, λ = 210 nm) *t*_R_ = 16.3 min (major), *t*_R_ = 17.9 min (minor). [α]: −55.1 (*c* = 0.34, CHCl_3_).

#### (2*S*)-4-*tert*-Butyl-2-phenyl-2-allyl-1,1-dioxo-1,4-thiazinane-2,4-dicarboxylate (**20a**)

A vial was charged with **17a** (66 mg, 0.15 mmol), [Pd_2_dba_3_] (6.9 mg, 7.5 μmol), **L4** (15.9 mg, 19.5 μmol), and 1,4-dioxane (1.5 mL). The mixture was stirred at room temperature for 15 h and then concentrated under reduced pressure. Purification by flash column chromatography [hexane:EtOAc 4:1] gave **20a** (50.5 mg, 85%) as a colorless oil. *R*_f_ = 0.43 [petrol:EtOAc 2:1]. ^1^H NMR (400 MHz, DMSO-*d*_6_, 130 °C) δ 7.45 (t, *J* = 7.8 Hz, 2H), 7.30 (t, *J* = 7.4 Hz, 1H), 7.15 (d, *J* = 8.1 Hz, 2H), 5.97 (ddt, *J* = 17.2, 10.1, 7.0 Hz, 1H), 5.35 (dq, *J* = 17.0, 1.5 Hz, 1H), 5.28 (dq, *J* = 10.1, 1.3 Hz, 1H), 4.20 (d, *J* = 14.9 Hz, 1H), 3.96 (d, *J* = 14.9 Hz, 1H), 3.90 (t, *J* = 5.6 Hz, 2H), 3.44 (qt, *J* = 14.3, 5.6 Hz, 2H), 3.06 (dd, *J* = 14.5, 7.1 Hz, 1H), 2.78 (tt, *J* = 7.5, 1.3 Hz, 1H), 1.44 (s, 9H) ppm. ^13^C{^1^H} NMR (100 MHz, DMSO-*d*_6_, 130 °C): δ 163.8, 152.8, 149.5, 129.8, 128.8, 125.7, 120.3, 119.7, 80.2, 70.5, 49.2, 47.5, 41.7, 32.3, 27.3 ppm. IR: ν_max_ (neat) 2978, 2931, 1751, 1695 cm^–1^. HRMS (APCI) *m/z*: calcd for C_19_H_25_NNaO_6_S [M + Na]^+^ 418.1295, found 418.1288. HPLC: 69% ee (Chiralpak AD-H, hexane:*i*-PrOH = 95:5, flow rate = 1 mL/min, 30.0 °C, λ = 204 nm) *t*_R_ = 17.1 min (major), *t*_R_ = 18.3 min (minor). [α]: −15.9 (*c* = 0.38, CHCl_3_).

#### (2*S*)-2-Benzyl-4-*tert*-butyl-2-allyl-1,1-dioxo-1,4-thiazinane-2,4-dicarboxylate (**20b**)

A vial was charged with **17b** (68 mg, 0.15 mmol), [Pd_2_dba_3_] (6.9 mg, 7.5 μmol), **L4** (15.9 mg, 19.5 μmol), and 1,4-dioxane (1.5 mL). The mixture was stirred at room temperature for 15 h and then concentrated under reduced pressure. Purification by flash column chromatography [hexane:EtOAc 4:1] gave **20b** (51 mg, 83%) as a colorless oil. *R*_f_ = 0.62 [petrol:EtOAc 2:1]. ^1^H NMR (400 MHz, DMSO-*d*_6_, 130 °C) δ 7.42–7.34 (m, 5H), 5.77 (ddt, *J* = 17.2, 10.2, 7.1 Hz, 1H), 5.25–5.09 (m, 4H), 4.02 (d, *J* = 14.5 Hz, 1H), 3.90 (d, *J* = 14.5 Hz, 1H), 3.87–3.73 (m, 2H), 3.41–3.26 (m, 2H), 2.91 (dd, *J* = 14.6, 7.0 Hz, 1H), 2.65 (ddt, *J* = 14.5, 7.1, 1.3 Hz, 1H), 1.44 (s, 9H) ppm. ^13^C{^1^H} NMR (100 MHz, DMSO-*d*_6_, 130 °C): δ 164.8, 152.7, 134.4, 129.9, 127.7, 127.5, 127.2, 119.4, 80.1, 70.3, 66.9, 49.0, 47.5, 41.6, 32.3, 27.3 ppm. IR: ν_max_ (neat) 2976, 2931, 1735, 1697 cm^–1^. HRMS (APCI) *m/z*: calcd for C_20_H_27_NNaO_6_S [M + Na]^+^ 432.1451, found 432.1445. HPLC: 62% ee (Chiralpak AD-H, hexane:*i*-PrOH = 95:5, flow rate = 1 mL/min, 30.0 °C, λ = 204 nm) *t*_R_ = 18.8 min (major), *t*_R_ = 32.9 min (minor). [α]: −6.0 (*c* = 0.34, CHCl_3_).

#### (2*S*)-*tert*-Butyl-2-allyl-2-benzoyl-1,1-dioxo-1,4-thiazinane-4-carboxylate (**20c**)

A vial was charged with **17c** (63.5 mg, 0.15 mmol), [Pd_2_dba_3_] (6.9 mg, 7.5 μmol), **L4** (15.9 mg, 19.5 μmol), and 1,4-dioxane (1.5 mL). The mixture was stirred at room temperature for 15 h and then concentrated under reduced pressure. Purification by flash column chromatography [hexane:EtOAc 4:1] gave **20c** (50 mg, 90%) as a colorless oil. *R*_f_ = 0.46 [petrol:EtOAc 2:1]. ^1^H NMR (400 MHz, DMSO-*d*_6_, 130 °C) δ 7.85 (dd, *J* = 8.4, 1.2 Hz, 2H), 7.59 (tt, *J* = 7.4, 1.4 Hz, 1H), 7.50 (t, *J* = 7.5 Hz, 2H), 5.61 (ddt, *J* = 17.3, 10.3, 7.0 Hz, 1H), 4.99 (dq, *J* = 10.3, 1.3 Hz, 1H), 4.93 (dq, *J* = 17.1, 1.6 Hz, 1H), 4.19 (d, *J* = 15.1 Hz, 1H), 4.08 (dd, *J* = 15.1, 1.4 Hz, 1H), 3.97 (dddd, *J* = 14.4, 6.7, 4.2, 1.5 Hz, 1H), 3.76 (ddd, *J* = 14.1, 8.2, 3.7 Hz, 1H), 3.51–3.36 (m, 2H), 3.22 (ddq, *J* = 15.4, 7.2, 1.2 Hz, 1H), 2.89 (ddt, *J* = 15.3, 6.8, 1.4 Hz, 1H), 1.38 (s, 9H) ppm. ^13^C{^1^H} NMR (100 MHz, DMSO-*d*_6_, 130 °C): δ 195.0, 152.8, 137.5, 131.6, 129.6, 127.5, 127.5, 119.4, 80.1, 75.5, 49.8, 48.1, 41.6, 32.8, 27.2 ppm. IR: ν_max_ (neat) 2980, 2931, 1695, 1673 cm^–1^. HRMS (ESI) *m/z*: calcd for C_19_H_25_NNaO_5_S [M + Na]^+^ 402.1346, found 402.1349. HPLC: 86% ee (Chiralcel OD-H, hexane:*i*-PrOH = 95:5, flow rate = 1 mL/min, 30.0 °C, λ = 210 nm) *t*_R_ = 22.9 min (major), *t*_R_ = 30.2 min (minor). [α]: −32.8 (*c* = 0.43, CHCl_3_).

#### (2*S*)-*tert*-Butyl-2-allyl-2-(2-methylpropanoyl)-1,1-dioxo-1,4-thiazinane-4-carboxylate (**20d**)

A vial was charged with **17d** (58.5 mg, 0.15 mmol), [Pd_2_dba_3_] (6.9 mg, 7.5 μmol), **L4** (15.9 mg, 19.5 μmol), and 1,4-dioxane (1.5 mL). The mixture was stirred at room temperature for 15 h and then concentrated under reduced pressure. Purification by flash column chromatography [hexane:EtOAc 6:1] gave **20d** (47 mg, 91%) as a colorless oil. *R*_f_ = 0.56 [petrol:EtOAc 2:1]. ^1^H NMR (400 MHz, DMSO-*d*_6_, 130 °C) δ 5.69 (dddd, *J* = 16.8, 10.2, 7.5, 6.4 Hz, 1H), 5.25 (dq, *J* = 17.0, 1.6 Hz, 1H), 5.19 (dq, *J* = 10.2, 1.4 Hz, 1H), 4.23 (dd, *J* = 14.9, 2.2 Hz, 1H), 4.21–4.14 (m, 1H), 3.70 (dd, *J* = 14.9, 1.3 Hz, 1H), 3.50–3.37 (m, 2H), 3.30 (hept, *J* = 6.7 Hz, 1H), 3.23–3.16 (m, 1H), 3.05 (ddq, *J* = 15.1, 7.4, 1.3 Hz, 1H), 2.77 (ddt, *J* = 15.1, 6.4, 1.6 Hz, 1H), 1.45 (s, 9H), 1.07 (d, *J* = 6.6 Hz, 3H), 1.07 (d, *J* = 1.5 Hz, 3H) ppm. ^13^C{^1^H} NMR (100 MHz, DMSO-*d*_6_, 130 °C): δ 206.6, 152.9, 129.9, 119.5, 80.0, 75.1, 49.0, 46.1, 41.6, 36.8, 32.4, 27.3, 18.7, 18.6 ppm. IR: ν_max_ (neat) 2978, 2931, 1695 cm^–1^. HRMS (ESI) *m/z*: calcd for C_16_H_27_NNaO_5_S [M + Na]^+^ 368.1502, found 368.1491. HPLC: 85% ee (Chiralpak AD-H, hexane:*i*-PrOH = 95:5, flow rate = 1 mL/min, 30.0 °C, λ = 210 nm) *t*_R_ = 8.0 min (major), *t*_R_ = 10.1 min (minor). [α]: −18.3 (*c* = 0.38, CHCl_3_).

### Synthesis of Allyl Enol Carbonates **27**

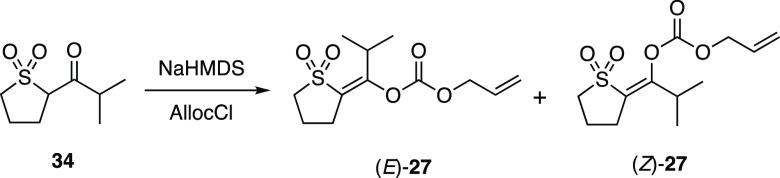



#### 1-((2*Z*)-1,1-Dioxo-thiolan-2-ylidene)-2-methylpropylprop-2-en-1-yl Carbonate ((*E*)-**27**) and 1-((2*E*)-1,1-Dioxo-thiolan-2-ylidene)-2-methylpropylprop-2-en-1-yl Carbonate ((*Z*)-**27**)

A solution of **34**(29) (234 mg, 1.23 mmol) in THF (20 mL) was cooled to −78 °C. NaHMDS (1 M in THF, 1.48 mL, 1.48 mmol) was added dropwise, and the mixture was stirred at −78 °C for 30 min. Allyl chloroformate (0.157 mL, 1.48 mmol) was added dropwise. The mixture was allowed to warm to room temperature and stirred for 15 h. The reaction was quenched with aq. HCl (1 N, 10 mL) and diluted with water (10 mL). The mixture was extracted with EtOAc (3 × 50 mL), washed with brine (100 mL), dried (MgSO_4_), and concentrated under reduced pressure. Purification by flash column chromatography [6:1–5:1–2:1 heptane:EtOAc] gave (*E*)-**27** (82 mg, 24%) as a colorless oil and (*Z*)-**27** (59 mg, 18%) as a colorless oil.

Isomer (*E*)-**27**. *R*_f_ = 0.33 [petrol:EtOAc 2:1]. ^1^H NMR (400 MHz, CDCl_3_) δ 5.95 (ddt, *J* = 17.1, 10.4, 5.8 Hz, 1H), 5.41 (dq, *J* = 17.2, 1.4 Hz, 1H), 5.33 (dq, *J* = 10.4, 1.2 Hz, 1H), 4.69 (dt, *J* = 5.8, 1.3 Hz, 2H), 3.42 (hept, *J* = 6.8 Hz, 1H), 3.12 (t, *J* = 7.1 Hz, 2H), 2.63 (t, *J* = 7.0 Hz, 2H), 2.15 (quint, *J* = 7.0 Hz, 2H), 1.13 (d, *J* = 6.8 Hz, 6H) ppm. ^13^C{^1^H} NMR (100 MHz, CDCl_3_): δ 157.3, 150.8, 130.7, 128.5, 119.8, 69.5, 52.3, 30.6, 25.6, 18.9, 18.8 ppm. IR: ν_max_ (neat) 2974, 2939, 1761, 1675 cm^–1^. HRMS (APCI) *m/z*: calcd for C_12_H_18_NO_5_S [M + NH_4_]^+^ 292.1213, found 292.1205.

Isomer (*Z*)-**27**. *R*_f_ = 0.11 [petrol:EtOAc 2:1]. ^1^H NMR (400 MHz, CDCl_3_) δ 5.97 (ddt, *J* = 17.2, 10.5, 5.8 Hz, 1H), 5.41 (dq, *J* = 17.2, 1.5 Hz, 1H), 5.29 (dq, *J* = 10.4, 1.2 Hz, 1H), 4.72 (dt, *J* = 5.8, 1.4 Hz, 2H), 3.06 (t, *J* = 7.1 Hz, 2H), 2.79 (t, *J* = 7.0 Hz, 2H), 2.67 (hept, *J* = 7.0 Hz, 1H), 2.21 (quint, *J* = 7.1 Hz, 2H), 1.16 (d, *J* = 7.0 Hz, 6H) ppm. ^13^C{^1^H} NMR (100 MHz, CDCl_3_): δ 155.8, 152.3, 131.1, 126.9, 119.2, 69.6, 51.3, 32.5, 24.8, 19.0, 18.8 ppm. IR: ν_max_ (neat) 2924, 2853, 1766, 1673 cm^–1^. HRMS (ESI) *m/z*: calcd for C_12_H_18_NaO_5_S [M + Na]^+^ 297.0767, found 297.0764.

### Synthesis of Deuterium-Labeled Substrates

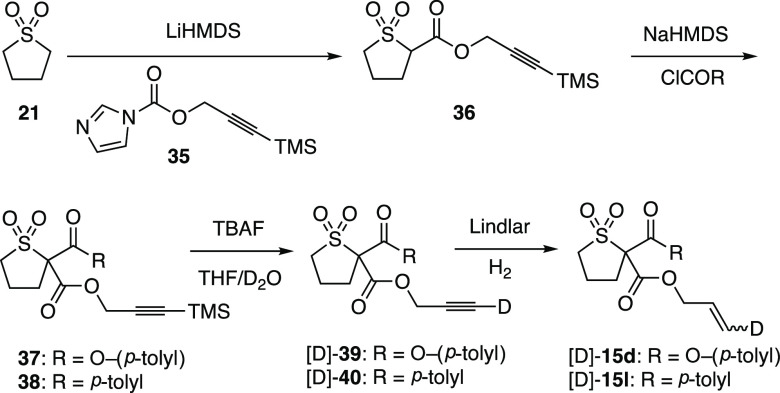



#### 3-Trimethylsilylprop-2-ynylimidazole-1-carboxylate (**35**)

A solution of 1,1′-carbonyldiimidazole (8.51 g, 52.5 mmol) in THF (200 mL) was cooled to 0 °C. (3-Trimethylsilyl)propargyl alcohol (5.18 mL, 35 mmol) was added dropwise. The reaction mixture was stirred at 0 °C for 2 h. The mixture was allowed to warm to room temperature and concentrated under reduced pressure. Purification by flash column chromatography [petrol:EtOAc 9:1–4:1] gave **35** (3.35 g, 43%) as a colorless oil. *R*_f_ = 0.32 [petrol:EtOAc 2:1]. ^1^H NMR (400 MHz, CDCl_3_) δ 8.18 (t, *J* = 1.1 Hz, 1H), 7.46 (t, *J* = 1.5 Hz, 1H), 7.08 (dd, *J* = 1.7, 0.8 Hz, 1H), 4.99 (s, 2H), 0.20 (s, 9H) ppm. ^13^C{^1^H} NMR (100 MHz, CDCl_3_): δ 148.0, 137.2, 130.7, 117.2, 96.7, 94.5, 56.1, −0.5 ppm. IR: ν_max_ (neat) 2961, 2902, 2186, 1763 cm^–1^. HRMS (APCI) *m/z*: calcd for C_10_H_15_N_2_O_2_Si [M + H]^+^ 223.0897, found 223.0890.

#### 3-Trimethylsilylprop-2-ynyl-1,1-dioxothiolane-2-carboxylate (**36**)

A solution of sulfolane (**21**, 418 mg, 3.48 mmol) in THF (25 mL) was cooled to −78 °C. LiHMDS (1 M in THF, 7.31 mL, 7.31 mmol) was added dropwise. The solution was stirred at −78 °C for 1 h. The mixture was allowed to warm to room temperature, and a solution of **35** (850 mg, 3.83 mmol) in THF (10 mL) was added dropwise. The mixture was stirred at room temperature for 1 h. The reaction was quenched with aq. HCl (1 N, 50 mL). The mixture was extracted with EtOAc (3 × 50 mL), washed with brine (100 mL), dried (MgSO_4_), and concentrated under reduced pressure. Purification by flash column chromatography [hexane:EtOAc 9:1–4:1] gave **36** (538 mg, 56%) as a colorless solid. *R*_f_ = 0.29 [petrol:EtOAc 2:1]. m.p.: 105–106 °C. ^1^H NMR (400 MHz, CDCl_3_): δ 4.92 (d, *J* = 15.8 Hz, 1H), 4.70 (d, *J* = 15.8 Hz, 1H), 3.96 (t, *J* = 7.6 Hz, 1H), 3.19–3.05 (m, 2H), 2.62–2.50 (m, 1H), 2.46–2.31 (m, 2H), 2.23–2.10 (m, 1H), 0.17 (s, 9H) ppm. ^13^C{^1^H} NMR (100 MHz, CDCl_3_): δ 164.9, 97.7, 93.2, 64.4, 54.5, 51.5, 26.0, 20.4, −0.4 ppm. IR: ν_max_ (neat) 2965, 2902, 2190, 1739 cm^–1^. HRMS (APCI) *m/z*: calcd for C_11_H_19_O_4_SSi [M + H]^+^ 275.0768, found 275.0767.

#### 2-(3-Trimethylsilylprop-2-ynyl)-2-tolyl dihydrothiophene-2,2(3*H*)-dicarboxylate 1,1-dioxide (**37**)

**36** (588 mg, 2.15 mmol) was dissolved in THF (25 mL). NaHMDS (1 M in THF, 2.37 mL, 2.37 mmol) was added dropwise. The solution was stirred at room temperature for 30 min. *p*-Tolyl chloroformate (0.34 mL, 2.37 mmol) was added dropwise, and the mixture was stirred for 5 h. The reaction was quenched with aq. HCl (1 N, 25 mL). The mixture was extracted with EtOAc (3 × 50 mL), washed with brine (100 mL), dried (MgSO_4_), and concentrated under reduced pressure. Purification by flash column chromatography [hexane:EtOAc 9:1–6:1] gave **37** (538 mg, 61%) as a colorless oil. *R*_f_ = 0.61 [petrol:EtOAc 2:1]. ^1^H NMR (400 MHz, CDCl_3_): δ 7.18 (d, *J* = 8.4 Hz, 2H), 7.08 (d, *J* = 8.5 Hz, 2H), 4.94 (d, *J* = 15.7 Hz, 1H), 4.89 (d, *J* = 15.7 Hz, 1H), 3.49–3.31 (m, 2H), 2.94 (dt, *J* = 14.9, 7.6 Hz, 1H), 2.75 (dt, *J* = 14.5, 7.4 Hz, 1H), 2.38–2.27 (m, 5H), 0.16 (s, 9H) ppm. ^13^C{^1^H} NMR (100 MHz, CDCl_3_): δ 163.9, 162.8, 148.1, 136.4, 130.0, 120.9, 97.1, 93.9, 74.8, 55.3, 50.6, 30.2, 20.9, 17.2, −0.5 ppm. IR: ν_max_ (neat) 3034, 2960, 2193, 1746 cm^–1^. HRMS (APCI) *m/z*: calcd for C_19_H_25_O_6_SSi [M + H]^+^ 409.1136, found 409.1126.

#### 3-Trimethylsilylprop-2-ynyl-2-(4-methylbenzoyl)-1,1-dioxo-thiolane-2-carboxylate (**38**)

**36** (533 mg, 1.95 mmol) was dissolved in THF (20 mL). NaHMDS (1 M in THF, 2.14 mL, 2.14 mmol) was added dropwise. The solution was stirred at room temperature for 30 min. *p*-Toluoyl chloride (0.283 mL, 2.14 mmol) was added dropwise, and the mixture was heated at 80 °C for 15 h. The reaction was quenched with aq. HCl (1 N, 25 mL). The mixture was extracted with EtOAc (3 × 50 mL), washed with brine (100 mL), dried (MgSO_4_), and concentrated under reduced pressure. Purification by flash column chromatography [hexane:EtOAc 9:1–4:1] gave **38** (577 mg, 75%) as a colorless oil. *R*_f_ = 0.52 [petrol:EtOAc 2:1]. ^1^H NMR (400 MHz, CDCl_3_): δ 7.88 (d, *J* = 8.4 Hz, 2H), 7.26 (d, *J* = 7.9 Hz, 2H), 4.78 (d, *J* = 15.6 Hz, 1H), 4.71 (d, *J* = 15.6 Hz, 1H), 3.46 (ddd, *J* = 13.0, 9.0, 6.7 Hz, 1H), 3.35 (ddd, *J* = 13.0, 8.6, 6.1 Hz, 1H), 3.20 (dt, *J* = 14.4, 7.7 Hz, 1H), 2.65 (ddd, *J* = 14.1, 7.4, 6.5 Hz, 1H), 2.40 (s, 3H), 2.38–2.19 (m, 2H), 0.12 (s, 9H) ppm. ^13^C{^1^H} NMR (100 MHz, CDCl_3_): δ 186.6, 165.8, 145.0, 132.5, 129.4, 129.4, 96.9, 93.4, 77.4, 54.6, 51.9, 32.0, 21.7, 17.5, −0.5 ppm. IR: ν_max_ (neat) 2957, 2917, 2849, 2186, 1740, 1683 cm^–1^. HRMS (APCI) *m/z*: calcd for C_19_H_25_O_5_SSi [M + H]^+^ 393.1186, found 393.1170.

#### 2-(3-^2^H-Prop-2-ynyl)-2-tolyldihydrothiophene-2,2(3*H*)-dicarboxylate 1,1-dioxide ([D]-**39**)

**37** (414 mg, 1.01 mmol) was dissolved in THF (15 mL). Deuterium oxide (5 mL) was added followed by tetrabutylammonium fluoride (1 M in THF, 1.12 mL, 1.12 mmol), and the reaction mixture was stirred at room temperature for 2 h. The solution was diluted with water (20 mL). The mixture was extracted with EtOAc (3 × 50 mL), washed with brine (100 mL), dried (MgSO_4_), and concentrated under reduced pressure. Purification by flash column chromatography [hexane:EtOAc 6:1–4:1] gave [D]-**39** (304 mg, 89%, 95% D) as a pale yellow solid. *R*_f_ = 0.32 [petrol:EtOAc 2:1]. m.p.: 97–98 °C. ^1^H NMR (400 MHz, CDCl_3_): δ 7.19 (d, *J* = 8.4 Hz, 2H), 7.08 (d, *J* = 8.5 Hz, 2H), 4.95 (d, *J* = 15.5 Hz, 1H), 4.89 (d, *J* = 15.4 Hz, 1H), 3.49–3.33 (m, 2H), 2.93 (dt, *J* = 14.8, 7.4 Hz, 1H), 2.79 (dt, *J* = 14.5, 7.3 Hz, 1H), 2.55 (t, *J* = 2.5 Hz, 0.05H), 2.38–2.29 (m, 5H) ppm. ^13^C{^1^H} NMR (100 MHz, CDCl_3_): δ 163.8, 162.8, 148.1, 136.4, 130.0, 120.8, 74.9, 54.5, 50.7, 30.2, 20.9, 17.3 ppm; two alkyne carbon signals were not observed. IR: ν_max_ (neat) 3017, 2965, 2918, 2850, 2579, 1984, 1767, 1743 cm^–1^. HRMS (APCI) *m/z*: calcd for C_16_H_16_DO_6_S [M + H]^+^ 338.0803, found 338.0794.

#### (3-^2^H-Prop-2-ynyl)-2-(4-methylbenzoyl)-1,1-dioxo-thiolane-2-carboxylate ([D]-**40**)

**38** (517 mg, 1.32 mmol) was dissolved in THF (15 mL). Deuterium oxide (5 mL) was added followed by tetrabutylammonium fluoride (1 M in THF, 1.98 mL, 1.98 mmol), and the reaction mixture was stirred at room temperature for 6 h. The solution was diluted with water (20 mL). The mixture was extracted with EtOAc (3 × 50 mL), washed with brine (100 mL), dried (MgSO_4_), and concentrated under reduced pressure. Purification by flash column chromatography [hexane:EtOAc 6:1] gave [D]-**40** (343 mg, 81%, 98% D) as a colorless solid. *R*_f_ = 0.24 [petrol:EtOAc 2:1]. m.p.: 143–144 °C. ^1^H NMR (400 MHz, CDCl_3_): δ 7.86 (d, *J* = 8.3 Hz, 2H), 7.26 (d, *J* = 7.9 Hz, 2H), 4.78 (d, *J* = 15.4 Hz, 1H), 4.68 (d, *J* = 15.4 Hz, 1H), 3.50–3.32 (m, 2H), 3.18 (dt, *J* = 14.8, 7.6 Hz, 1H), 2.69 (ddd, *J* = 14.2, 7.4, 6.4 Hz, 1H), 2.53 (t, *J* = 2.4 Hz, 0.01H), 2.40 (s, 3H), 2.37–2.20 (m, 2H) ppm. ^13^C{^1^H} NMR (100 MHz, CDCl_3_): δ 186.9, 165.8, 145.1, 132.6, 129.4, 129.3, 77.4, 53.9, 51.8, 31.9, 21.7, 17.6 ppm; two alkyne carbon signals were not observed. IR: ν_max_ (neat) 3013, 2958, 2920, 2578, 1981, 1744, 1677 cm^–1^. HRMS (APCI) *m/z*: calcd for C_16_H_16_DO_5_S [M + H]^+^ 322.0854, found 322.0838.

#### 2-(3-^2^H-Allyl)-2-tolyl-dihydrothiophene-2,2(3*H*)-dicarboxylate-1,1-dioxide ([D]-**15d**)

A suspension of [D]-**39** (194 mg, 0.58 mmol), Pd/CaCO_3_ (19.4 mg), and quinoline (0.136 mL, 1.15 mmol) in EtOAc (11.5 mL) was degassed with argon. The mixture was cooled to 0 °C and then stirred at 0 °C under a hydrogen atmosphere for 20 min. The suspension was filtered through a pad of Celite, washed with aq. HCl (1 N, 15 mL) and brine (15 mL), dried (MgSO_4_), and concentrated under reduced pressure. Purification by flash column chromatography [hexane:EtOAc 9:1] gave [D]-**15d** (160 mg, 82%, 93% D) as a colorless solid. *R*_f_ = 0.32 [petrol:EtOAc 2:1]. m.p.: 60–61 °C. ^1^H NMR (400 MHz, CDCl_3_): δ 7.18 (d, *J* = 8.1 Hz, 2H), 7.05 (d, *J* = 8.5 Hz, 2H), 6.02–5.90 (m, 1H), 5.44 (ddt, *J* = 17.2, 4.9, 1.4 Hz, 0.25H), 5.34–5.28 (m, 0.82H), 4.83 (dt, *J* = 5.9, 1.3 Hz, 2H), 3.47–3.33 (m, 2H), 2.91 (dt, *J* = 14.9, 7.6 Hz, 1H), 2.79 (dt, *J* = 14.5, 7.4 Hz, 1H), 2.36–2.28 (m, 5H) ppm. ^13^C{^1^H} NMR (100 MHz, CDCl_3_): δ 164.2, 163.1, 148.1, 136.4, 130.4, 130.0, 120.8, 119.5 (t, *J* = 24.1 Hz), 75.0, 67.7, 50.5, 30.2, 20.8, 17.3 ppm. IR: ν_max_ (neat) 3034, 2954, 1735 cm^–1^. HRMS (APCI) *m/z*: calcd for C_16_H_18_DO_6_S [M + H]^+^ 340.0960, found 340.0947.

#### (3-^2^H-Allyl)-2-(4-methylbenzoyl)-1,1-dioxo-thiolane-2-carboxylate ([D]-**15l**)

A suspension of [D]-**40** (212 mg, 0.66 mmol), Pd/CaCO_3_ (21.2 mg), and quinoline (0.156 mL, 1.32 mmol) in EtOAc (13 mL) was degassed with argon. The mixture was stirred at room temperature under a hydrogen atmosphere for 15 min. The suspension was filtered through a pad of Celite, washed with aq. HCl (1 N, 15 mL) and brine (15 mL), dried (MgSO_4_), and concentrated under reduced pressure. Purification by flash column chromatography [hexane:EtOAc 9:1–6:1] gave [D]-**15l** (152 mg, 71%, 93% D) as a colorless solid. *R*_f_ = 0.34 [petrol:EtOAc 2:1]. m.p.: 68–69 °C. ^1^H NMR (300 MHz, CDCl_3_): δ 7.85 (d, *J* = 8.5 Hz, 2H), 7.25 (d, *J* = 8.5 Hz, 2H), 5.75–5.59 (m, 1H), 5.23–5.09 (m, 1.07H), 4.63 (dd, *J* = 5.9, 1.2 Hz, 2H), 3.40 (qdd, *J* = 13.0, 8.7, 6.6 Hz, 2H), 3.13 (dt, *J* = 14.7, 7.5 Hz, 1H), 2.70 (dt, *J* = 14.2, 7.0 Hz, 1H), 2.40 (s, 3H), 2.38–2.16 (m, 2H) ppm. ^13^C{^1^H} NMR (75 MHz, CDCl_3_): δ 187.5, 166.2, 144.9, 132.8, 130.1, 129.4, 129.2, 119.6 (t, *J* = 24.6 Hz), 77.7, 67.2, 51.7, 31.9, 21.7, 17.6 ppm. IR: ν_max_ (neat) 3050, 3017, 2999, 2953, 1733, 1675 cm^–1^. HRMS (APCI) *m/z*: calcd for C_16_H_18_DO_5_S [M + H]^+^ 324.1010, found 324.0997.

## Supporting Information Available

Copies of NMR spectra of all new compounds, HPLC traces for ee determination, X-ray data for **18b**, and details of the mechanistic study (PDF)
