# Supplementary material for: Catalytic Enantioselective Synthesis of α-Difunctionalized Cyclic Sulfones
Source: J Org Chem. 2022 Jul 8;87(15):10256–76. doi: 10.1021/acs.joc.2c01240 (PMC9490805; doi:10.1021/acs.joc.2c01240)
Supplement: Supplementary file 1 — jo2c01240_si_001.pdf [file jo2c01240_si_001.pdf]

Catalytic Enantioselective Synthesis of α-Difunctionalized Cyclic Sulfones

*Eleanor Bowen, Gillian Laidlaw, Bethany C. Atkinson, Timur A. McArdle-Ismaguilov and Vilius Franckevičius**

Department of Chemistry, Lancaster University, Lancaster LA1 4YB, U.K.

E-mail*: v.franckevicius@lancaster.ac.uk

SUPPORTING INFORMATION

1. NMR Spectra S1

2. HPLC Data S96

3. X-Ray Data for **18b** S172

4. Mechanistic Study

4.1. Effect of Enolate Geometry S174

4.2. Enolate Crossover S177

4.3. Competing Allylation S187

1. NMR Spectra.

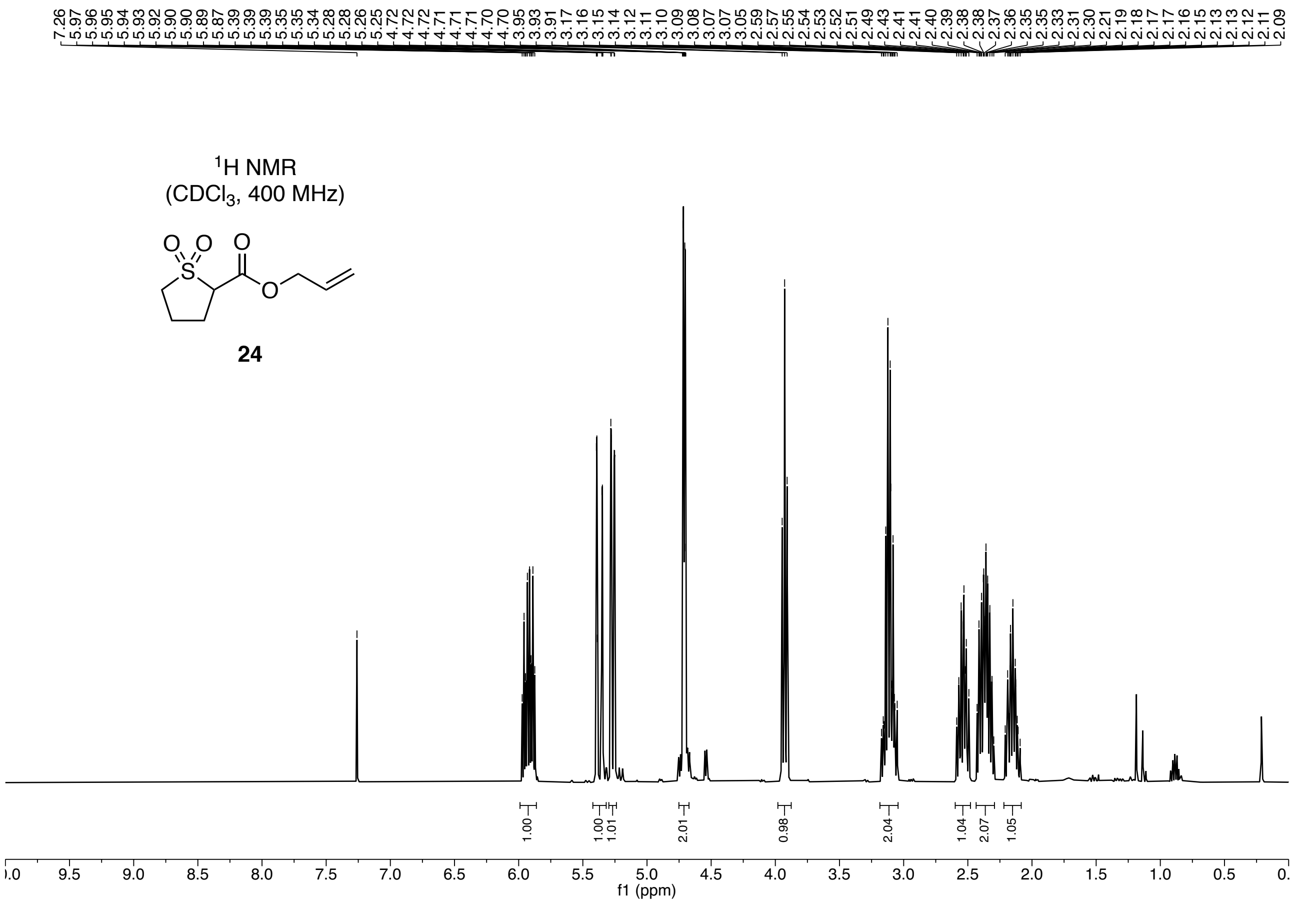

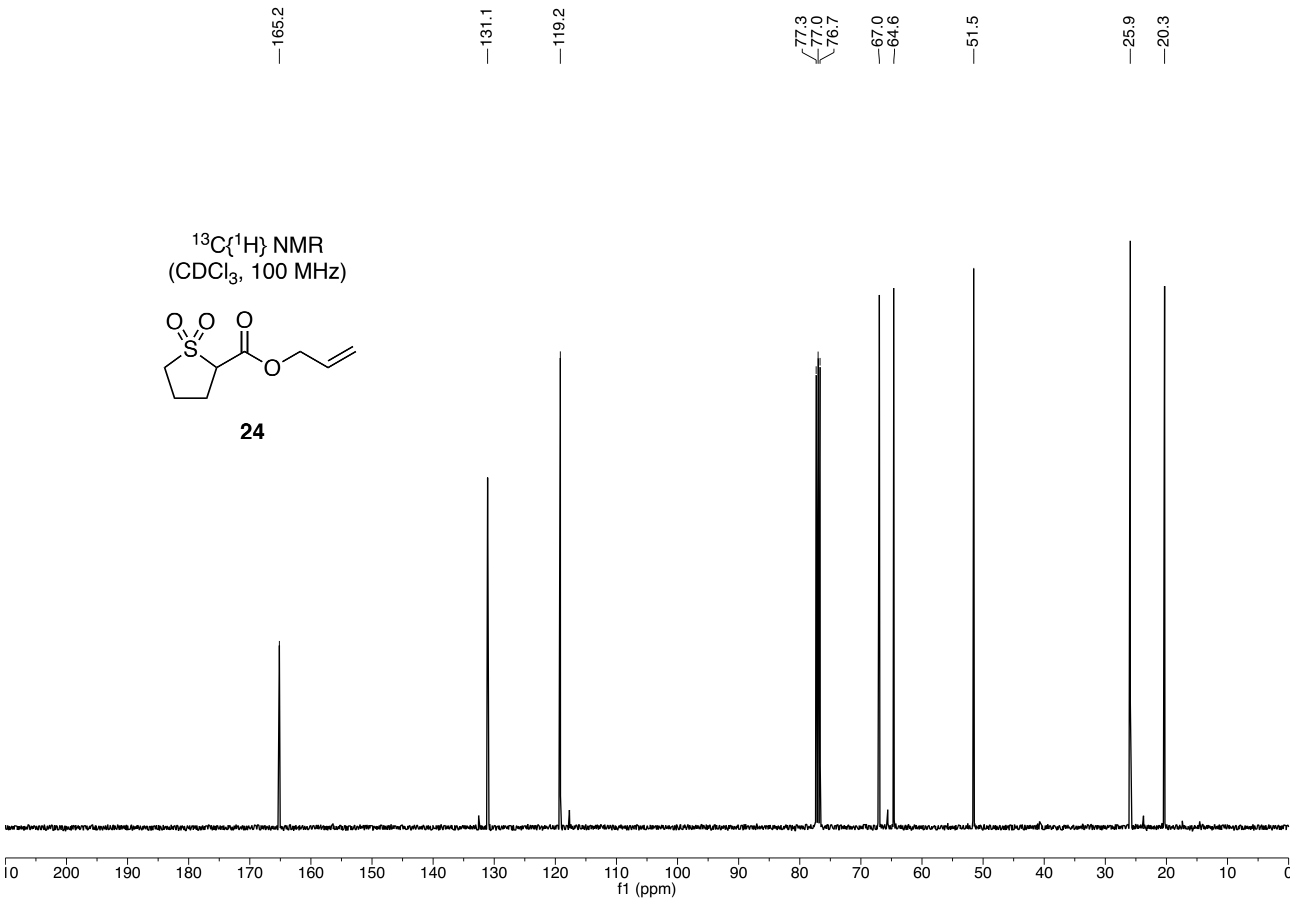

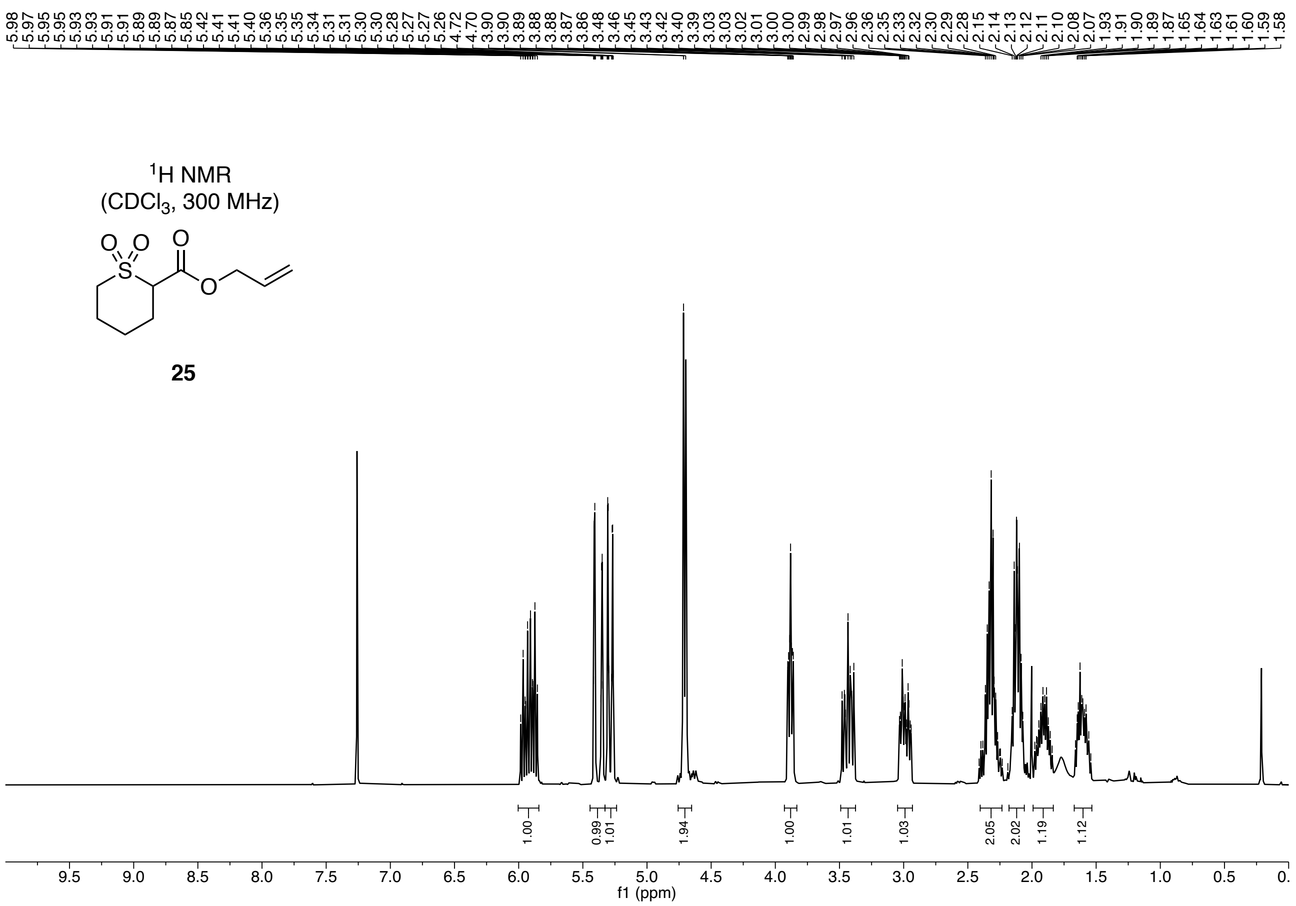
5.98 5.97 5.95 5.93 5.93 5.91 5.91 5.89 5.89 5.87 5.85 5.42 5.41 5.40 5.36 5.35 5.34 5.31 5.31 5.30 5.30 5.28 5.27 5.26 4.72 4.70 3.90 3.90 3.89 3.88 3.88 3.87 3.86 3.48 3.46 3.45 3.43 3.42 3.40 3.39 3.03 3.03 3.02 3.01 3.00 3.00 2.99 2.98 2.97 2.96 2.36 2.35 2.33 2.32 2.30 2.29 2.28 2.15 2.14 2.13 2.12 2.11 2.10 2.08 2.07 1.93 1.91 1.90 1.89 1.87 1.65 1.64 1.63 1.61 1.60 1.59 1.58
1H NMR
(CDCl3, 300 MHz)
25
1.00 0.99 1.01 1.94 1.00 1.01 1.03 2.05 2.02 1.19 1.12
9.5 9.0 8.5 8.0 7.5 7.0 6.5 6.0 5.5 5.0 4.5 4.0 3.5 3.0 2.5 2.0 1.5 1.0 0.5
f1 (ppm)

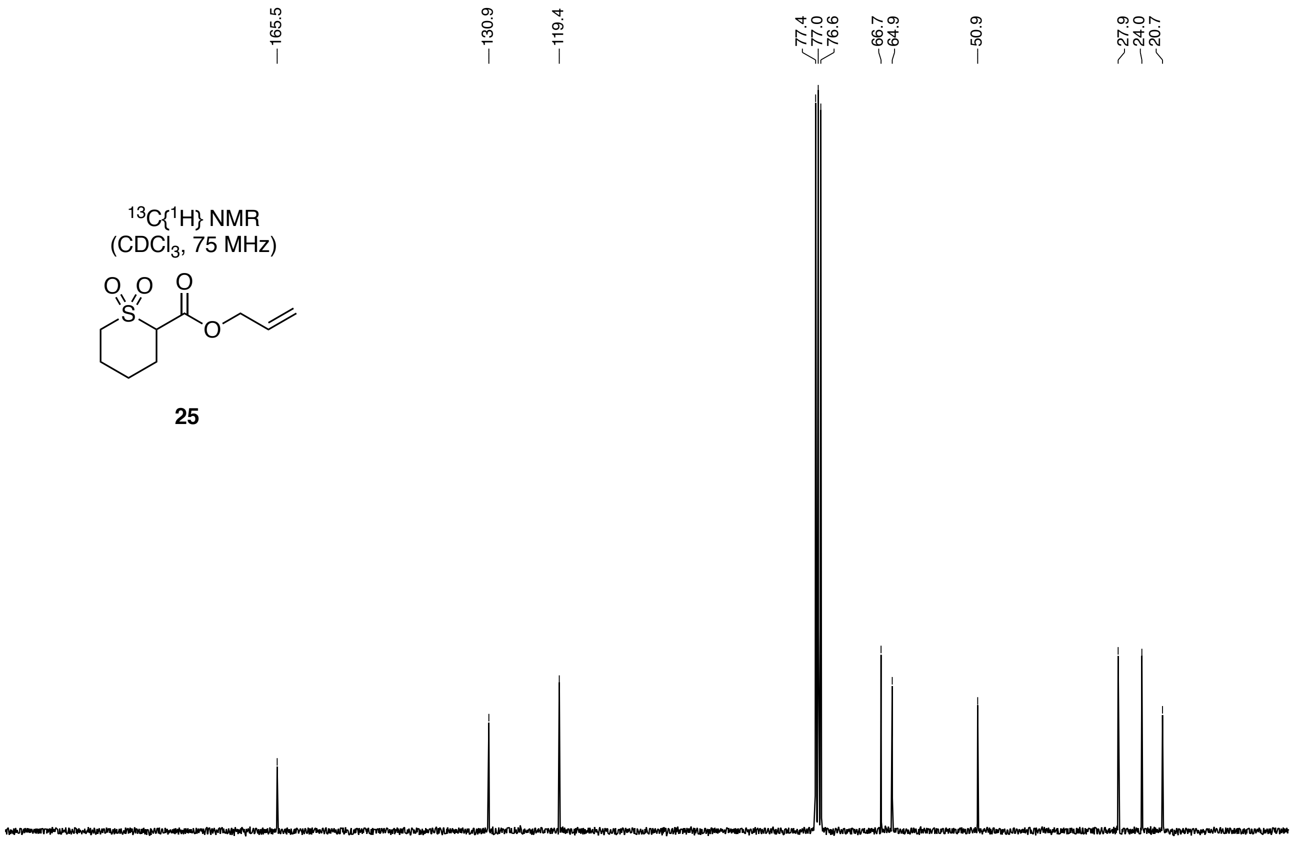
165.5 130.9 119.4 77.4 77.0 76.6 66.7 64.9 50.9 27.9 24.0 20.7
13C{1H} NMR
(CDCl3, 75 MHz)
25
200 190 180 170 160 150 140 130 120 110 100 90 80 70 60 50 40 30 20 10
f1 (ppm)

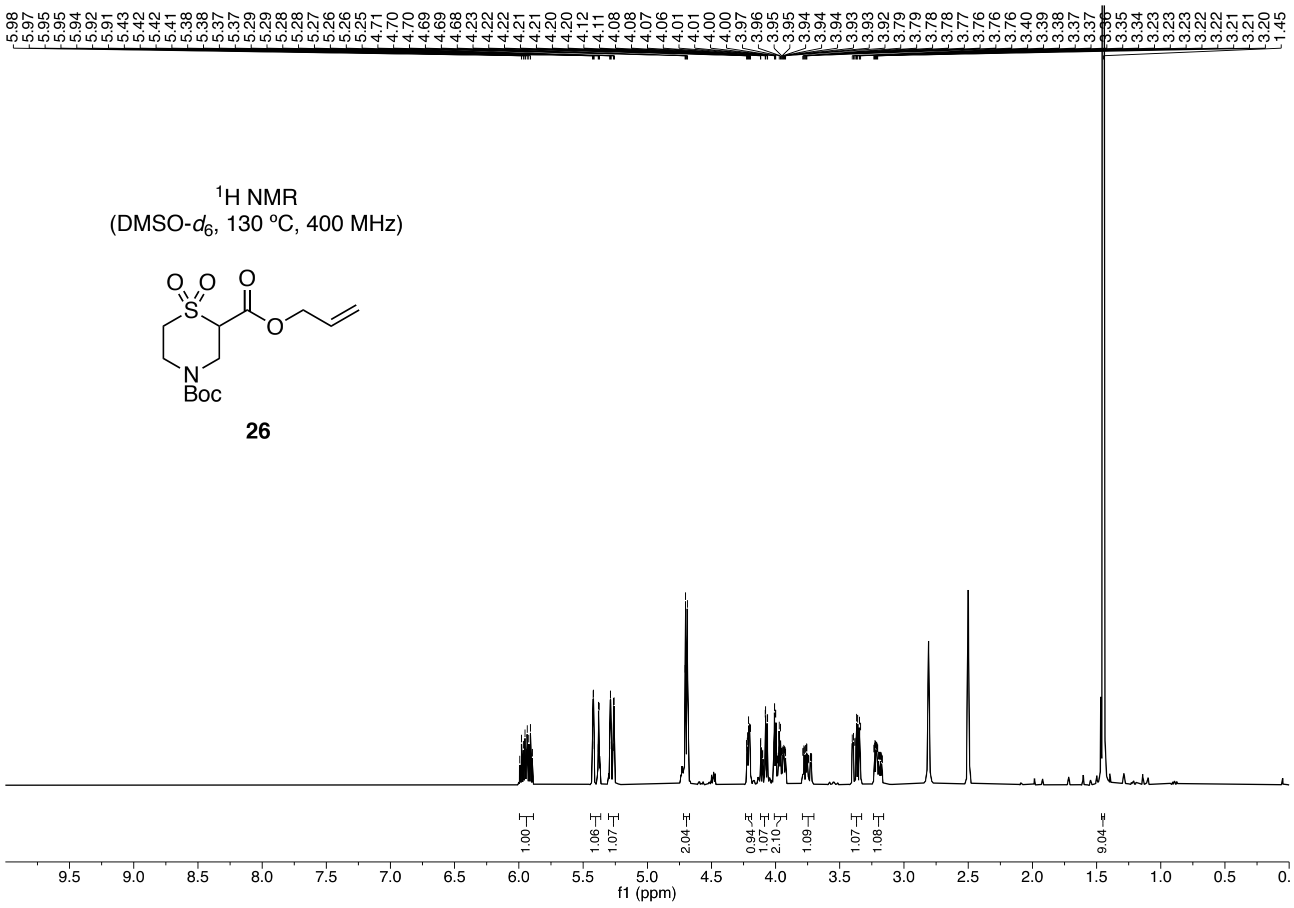
1H NMR
(DMSO-d6, 130 °C, 400 MHz)
Boc
26
f1 (ppm)

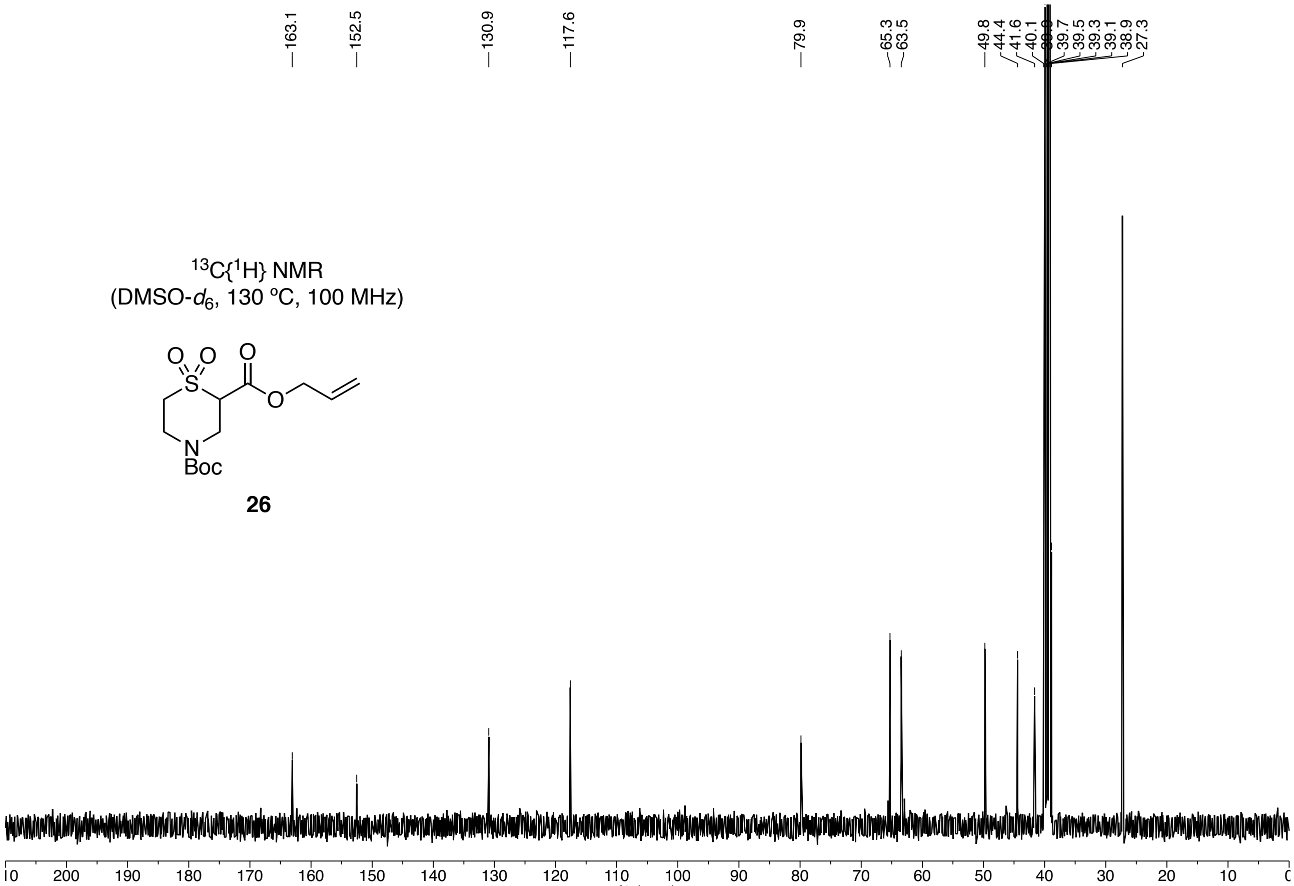
163.1
152.5
130.9
117.6
79.9
65.3
63.5
49.8
44.4
41.6
40.1
39.7
39.5
39.3
39.1
38.9
27.3
13C{1H} NMR
(DMSO-d6, 130 °C, 100 MHz)
Boc
26
f1 (ppm)

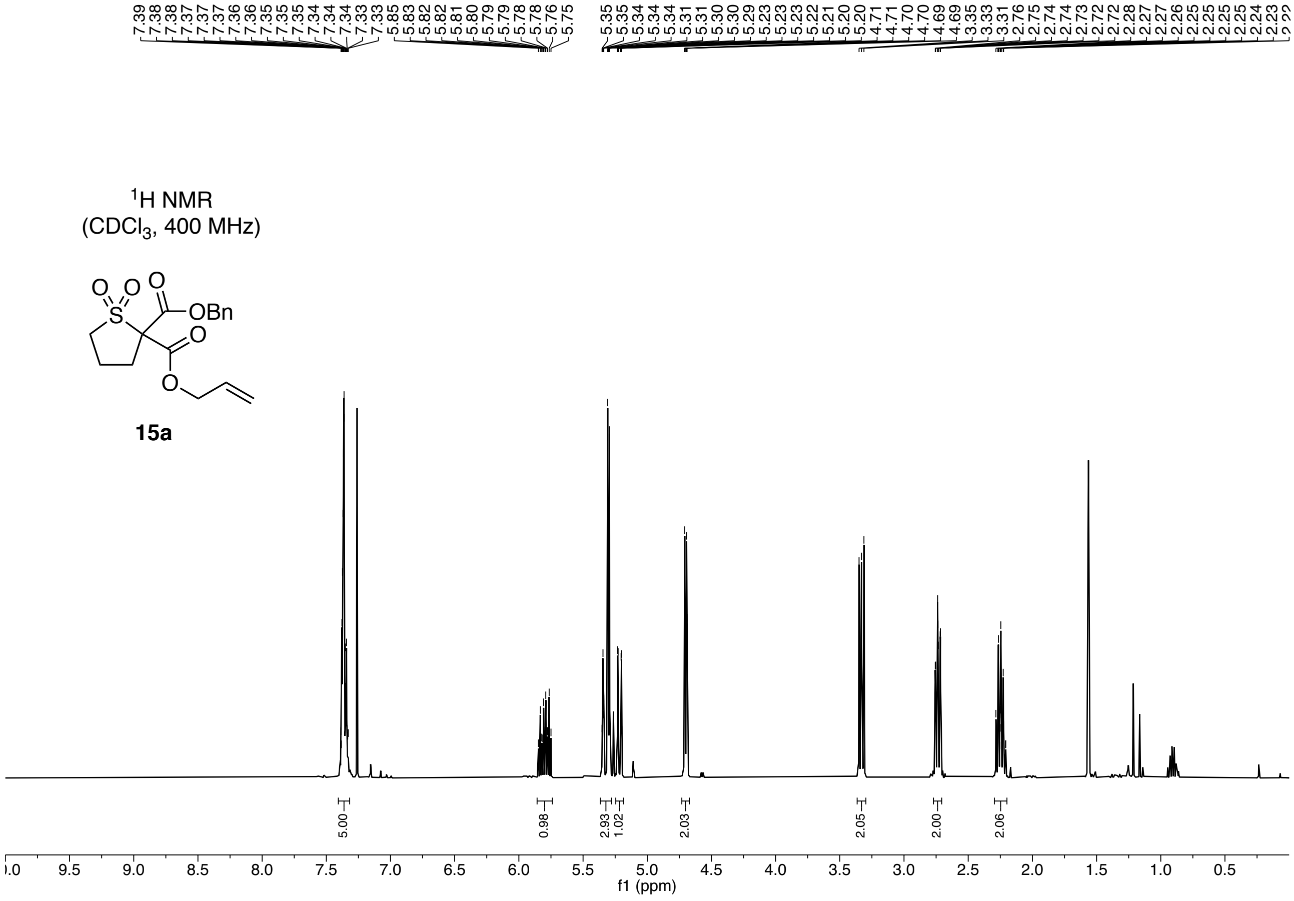

1H NMR
(CDCl3, 400 MHz)
OBn
15a
5.00
0.98
2.93
1.02
2.03
2.05
2.00
2.06
f1 (ppm)

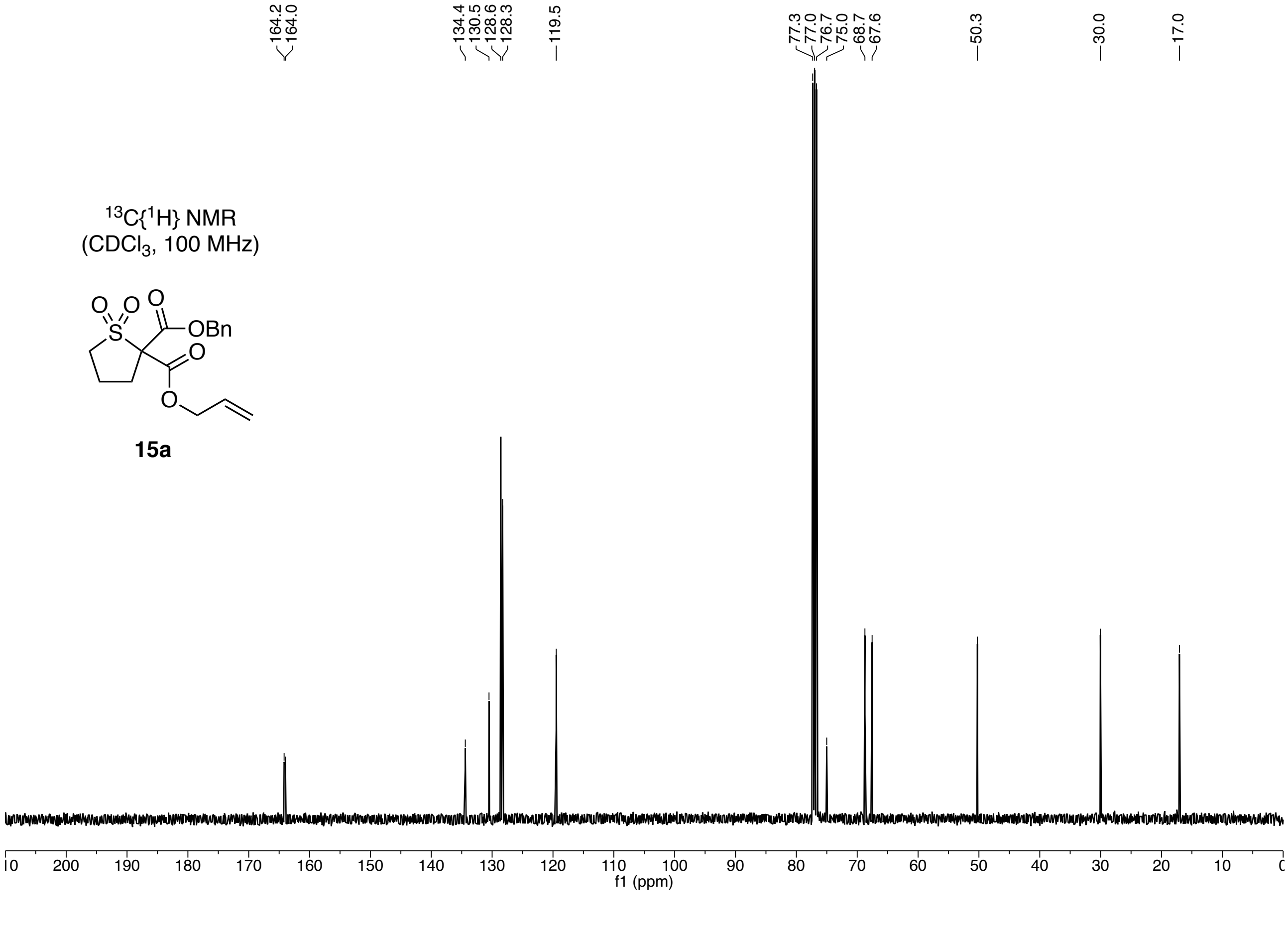

164.2
164.0
134.4
130.5
128.6
128.3
119.5
77.3
77.0
76.7
75.0
68.7
67.6
50.3
30.0
17.0
13C{1H} NMR
(CDCl3, 100 MHz)
OBn
15a
f1 (ppm)

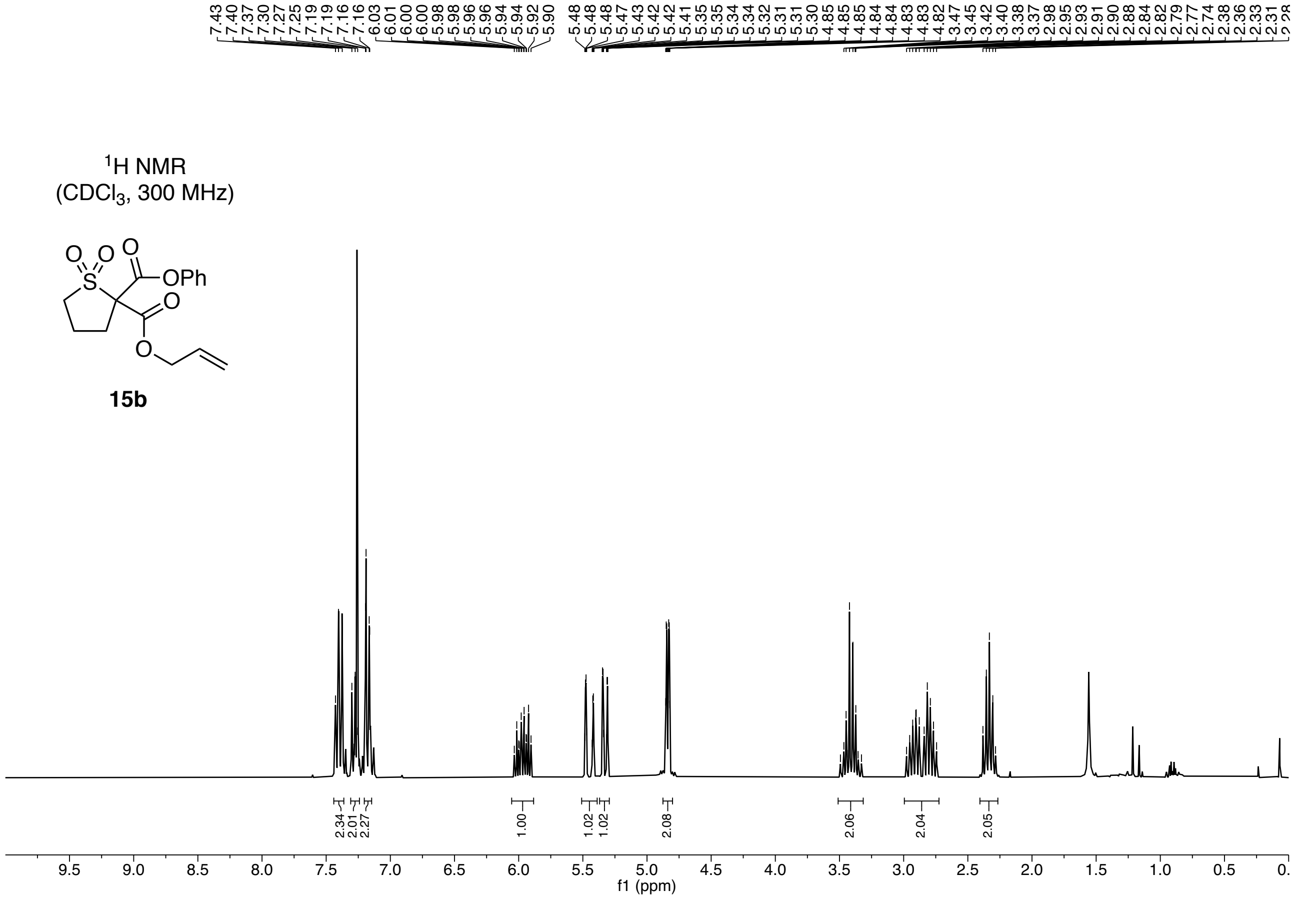
7.43 7.40 7.37 7.30 7.27 7.25 7.19 7.16 7.16 6.03 6.01 6.00 5.98 5.96 5.96 5.94 5.94 5.92 5.90
5.48 5.48 5.48 5.47 5.43 5.42 5.42 5.41 5.35 5.35 5.34 5.32 5.31 5.31 5.30 4.85 4.85 4.84 4.84 4.83 4.83 4.82 3.47 3.45 3.45 3.42 3.40 3.38 3.37 2.98 2.98 2.95 2.93 2.91 2.90 2.88 2.84 2.82 2.82 2.79 2.77 2.74 2.38 2.36 2.33 2.31
^{1}H NMR
($CDCl_3$, 300 MHz)
OPh
15b
2.34 2.01 2.27 1.00 1.02 1.02 2.08 2.06 2.04 2.05
f1 (ppm)

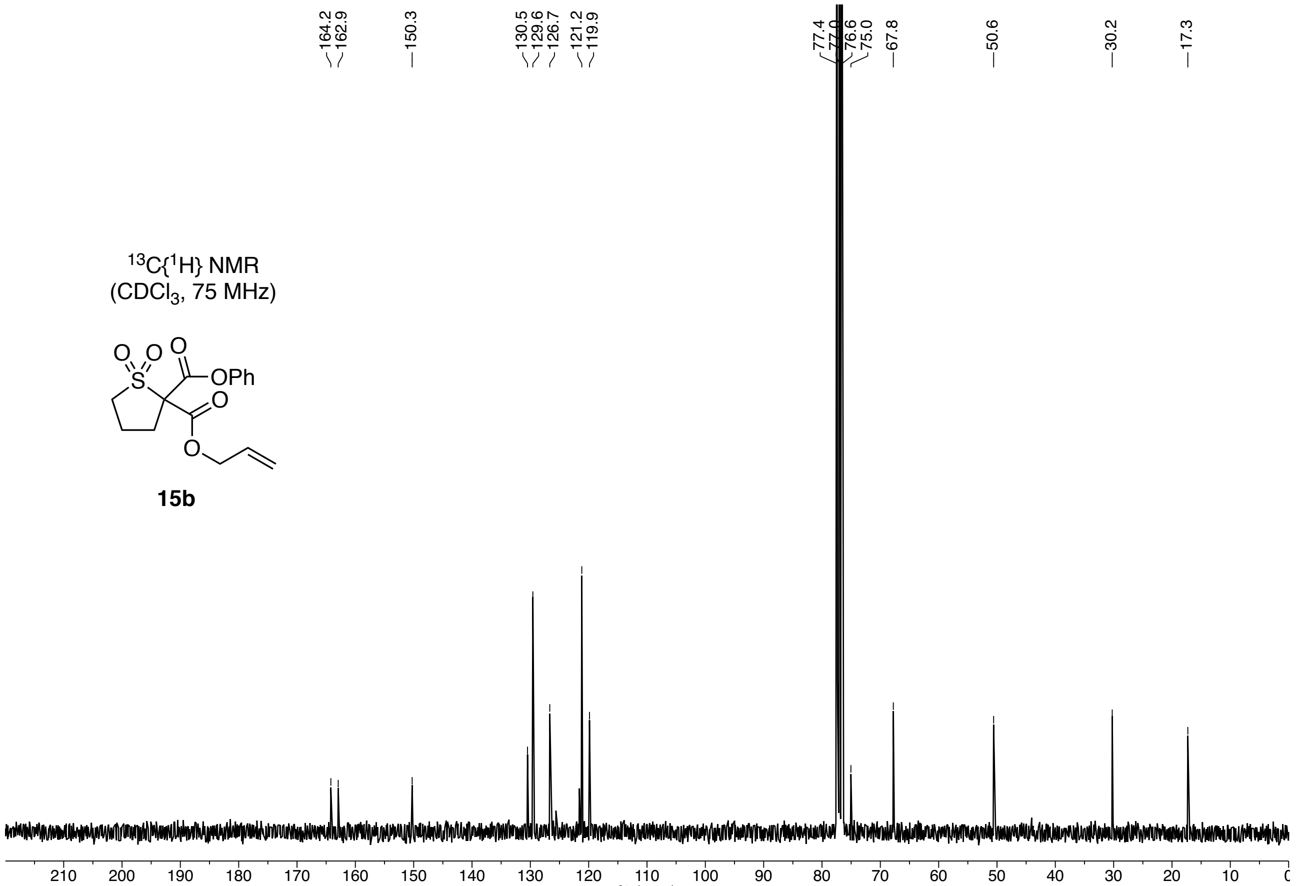
164.2 162.9 150.3 130.5 129.6 126.7 121.2 119.9 77.4 77.0 76.6 75.0 67.8 50.6 30.2 17.3
$^{13}C\{^1H\}$ NMR
($CDCl_3$, 75 MHz)
OPh
15b
f1 (ppm)

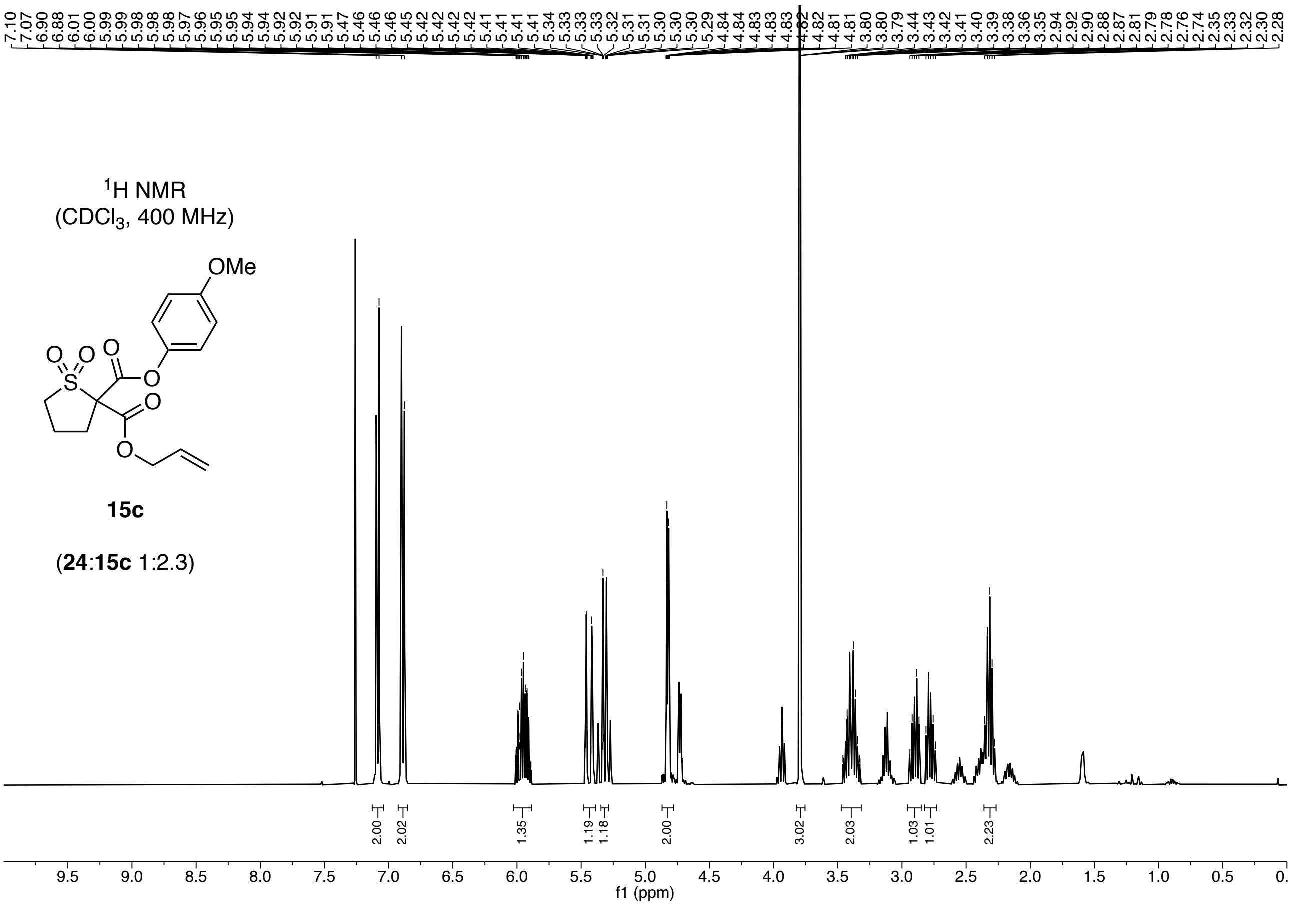
7.10 7.07 6.90 6.88 6.01 6.00 5.99 5.99 5.98 5.98 5.98 5.97 5.96 5.95 5.95 5.94 5.94 5.92 5.92 5.91 5.91 5.47 5.46 5.46 5.45 5.42 5.42 5.42 5.42 5.41 5.41 5.41 5.41 5.34 5.33 5.33 5.32 5.31 5.31 5.30 5.30 5.30 5.29 4.84 4.84 4.83 4.83 4.83 4.82 4.82 4.81 3.80 3.80 3.79 3.44 3.43 3.42 3.41 3.40 3.39 3.38 3.36 3.35 2.94 2.92 2.92 2.90 2.88 2.87 2.81 2.79 2.78 2.76 2.74 2.35 2.33 2.32 2.30 2.28
1H NMR
(CDCl3, 400 MHz)
OMe
O O
S
O
O
O
O
15c
(24:15c 1:2.3)
2.00 2.02 1.35 1.19 1.18 2.00 3.02 2.03 1.03 1.01 2.23
9.5 9.0 8.5 8.0 7.5 7.0 6.5 6.0 5.5 5.0 4.5 4.0 3.5 3.0 2.5 2.0 1.5 1.0 0.5 0.
f1 (ppm)

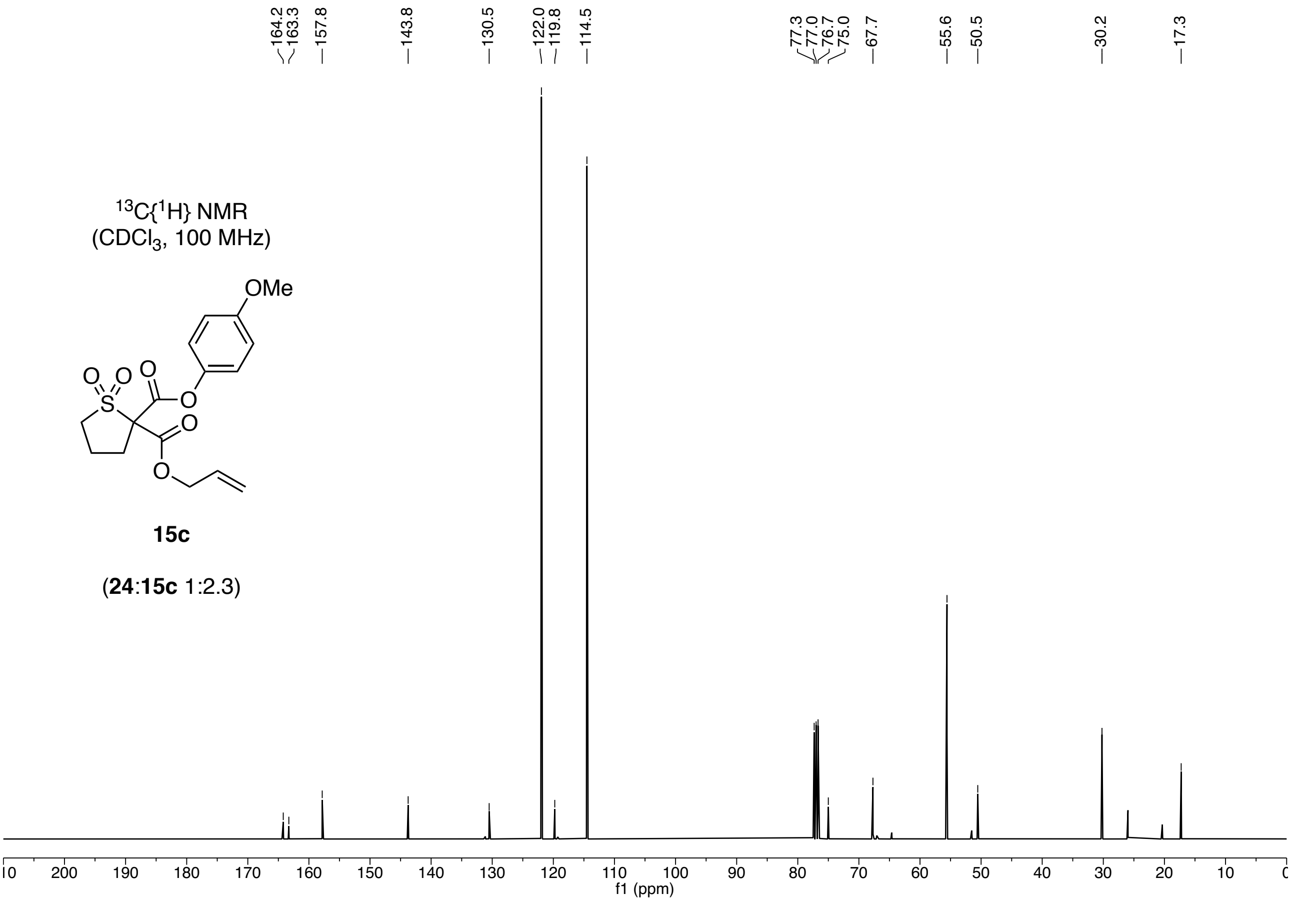
164.2 163.3 157.8 143.8 130.5 122.0 119.8 114.5 77.3 77.0 76.7 75.0 67.7 55.6 50.5 30.2 17.3
13C{1H} NMR
(CDCl3, 100 MHz)
OMe
O O
S
O
O
O
O
15c
(24:15c 1:2.3)
200 190 180 170 160 150 140 130 120 110 100 90 80 70 60 50 40 30 20 10
f1 (ppm)

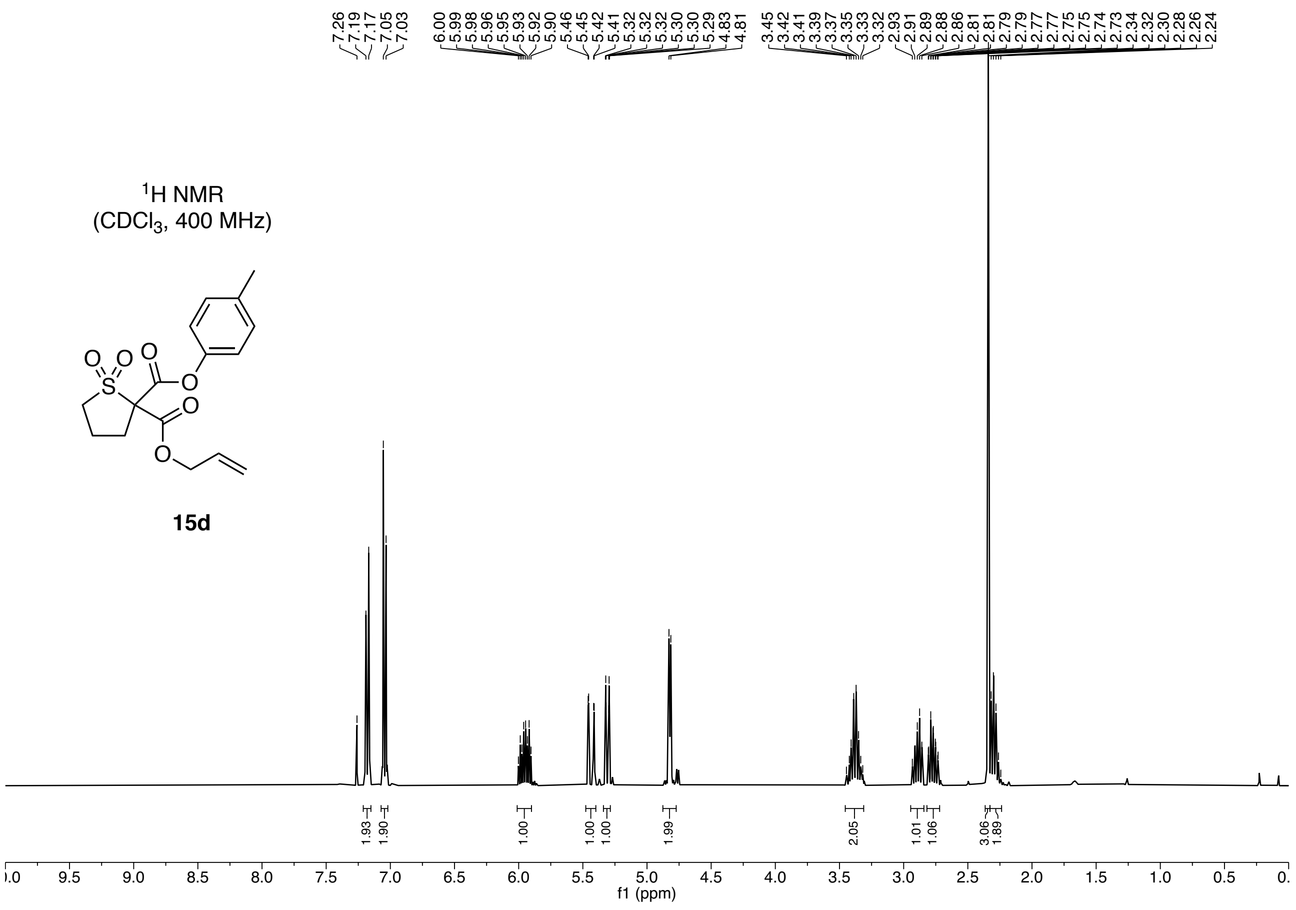

7.26 7.19 7.17 7.05 7.03 6.00 5.99 5.98 5.96 5.95 5.93 5.92 5.90 5.46 5.45 5.42 5.41 5.32 5.32 5.32 5.30 5.30 5.29 4.83 4.81 3.45 3.42 3.41 3.39 3.37 3.35 3.33 3.32 2.93 2.91 2.89 2.88 2.86 2.81 2.81 2.79 2.79 2.77 2.77 2.75 2.74 2.73 2.34 2.32 2.30 2.28 2.26 2.24
^{1}H NMR
(CDCl$_3$, 400 MHz)
15d
1.93 1.90 1.00 1.00 1.00 1.99 2.05 1.01 1.06 3.06 1.89
f1 (ppm)

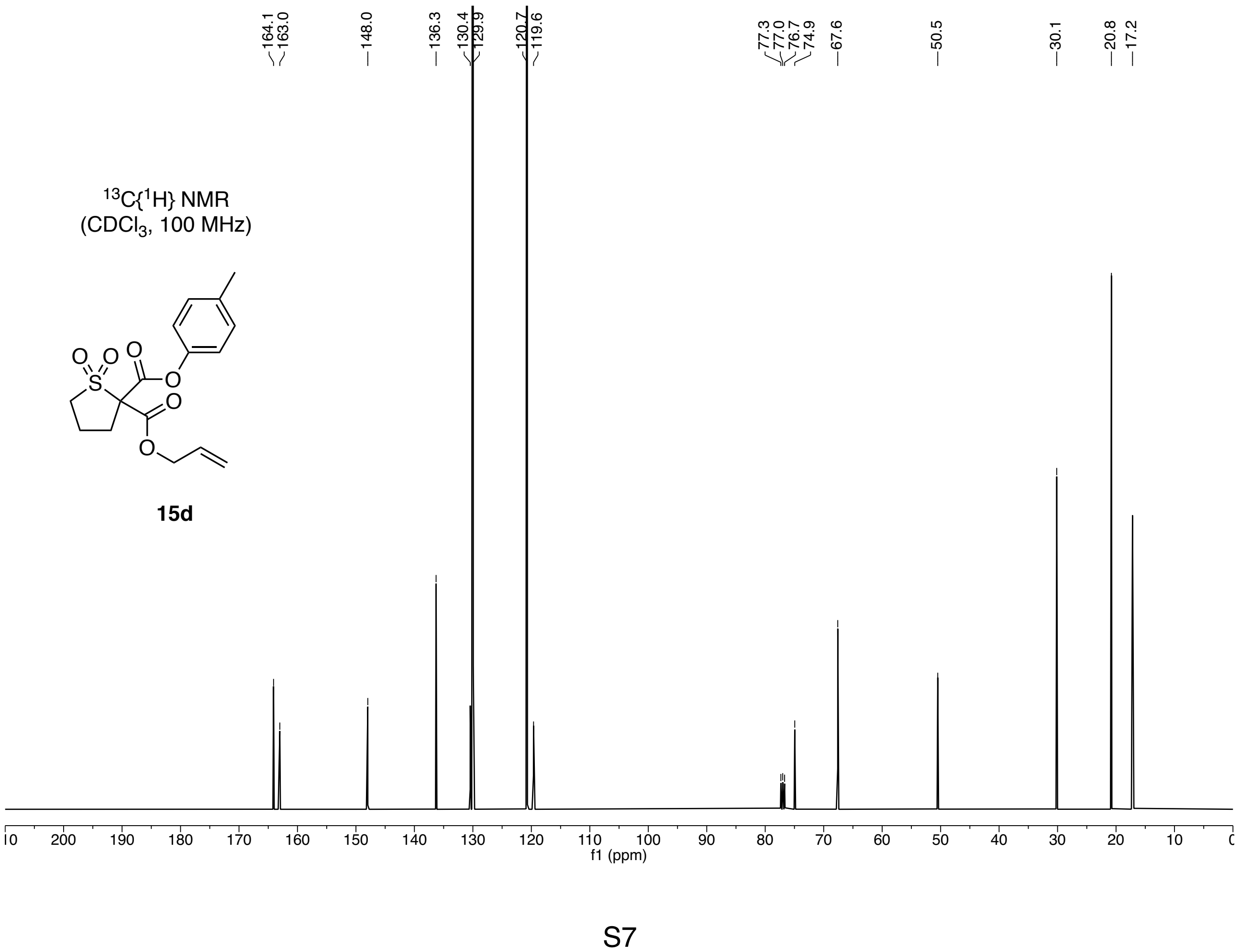

164.1 163.0 148.0 136.3 130.4 129.9 120.7 119.6 77.3 77.0 76.7 74.9 67.6 50.5 30.1 20.8 17.2
^{13}C{^{1}H} NMR
(CDCl$_3$, 100 MHz)
15d
f1 (ppm)

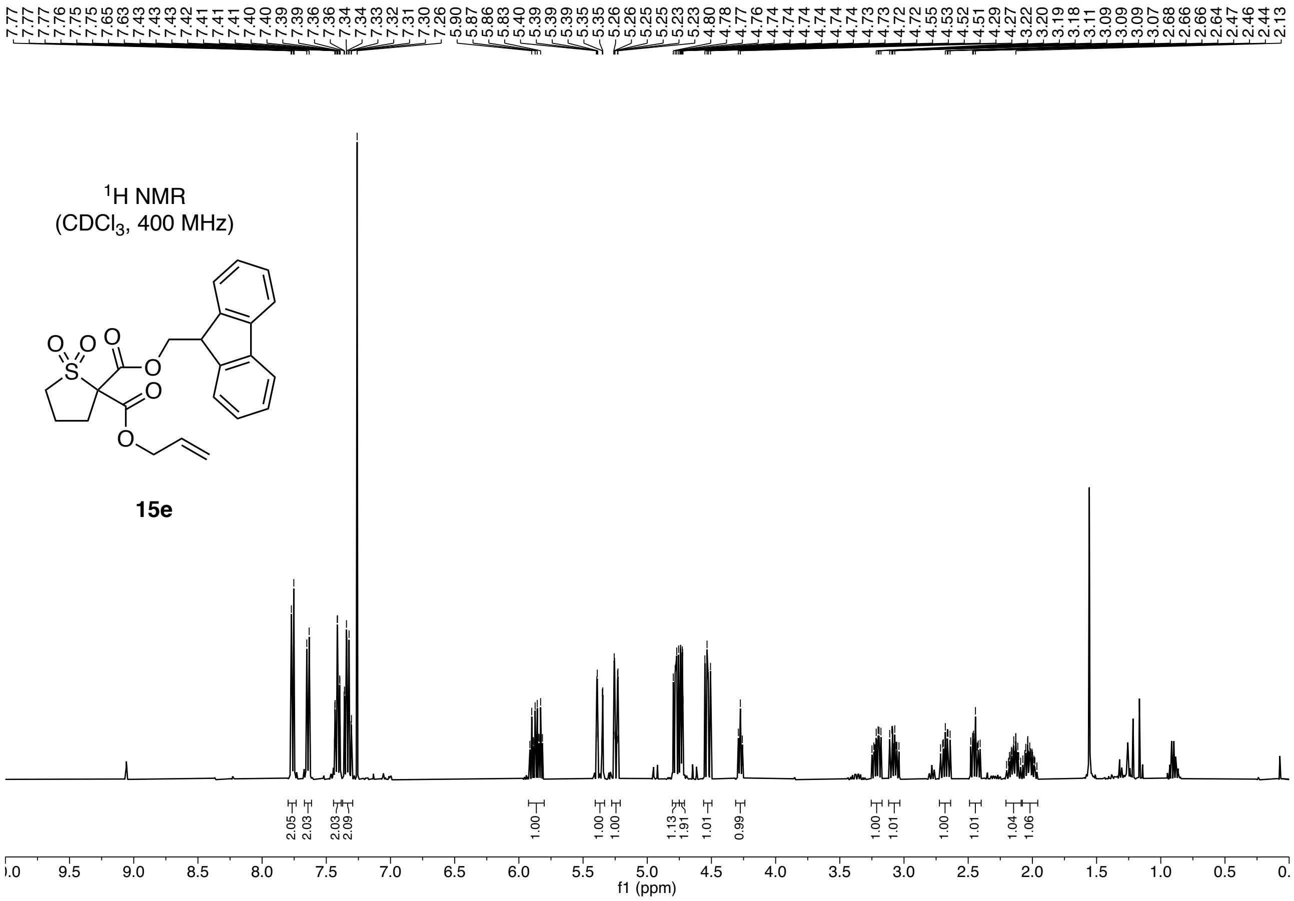
1H NMR
(CDCl3, 400 MHz)
15e
f1 (ppm)

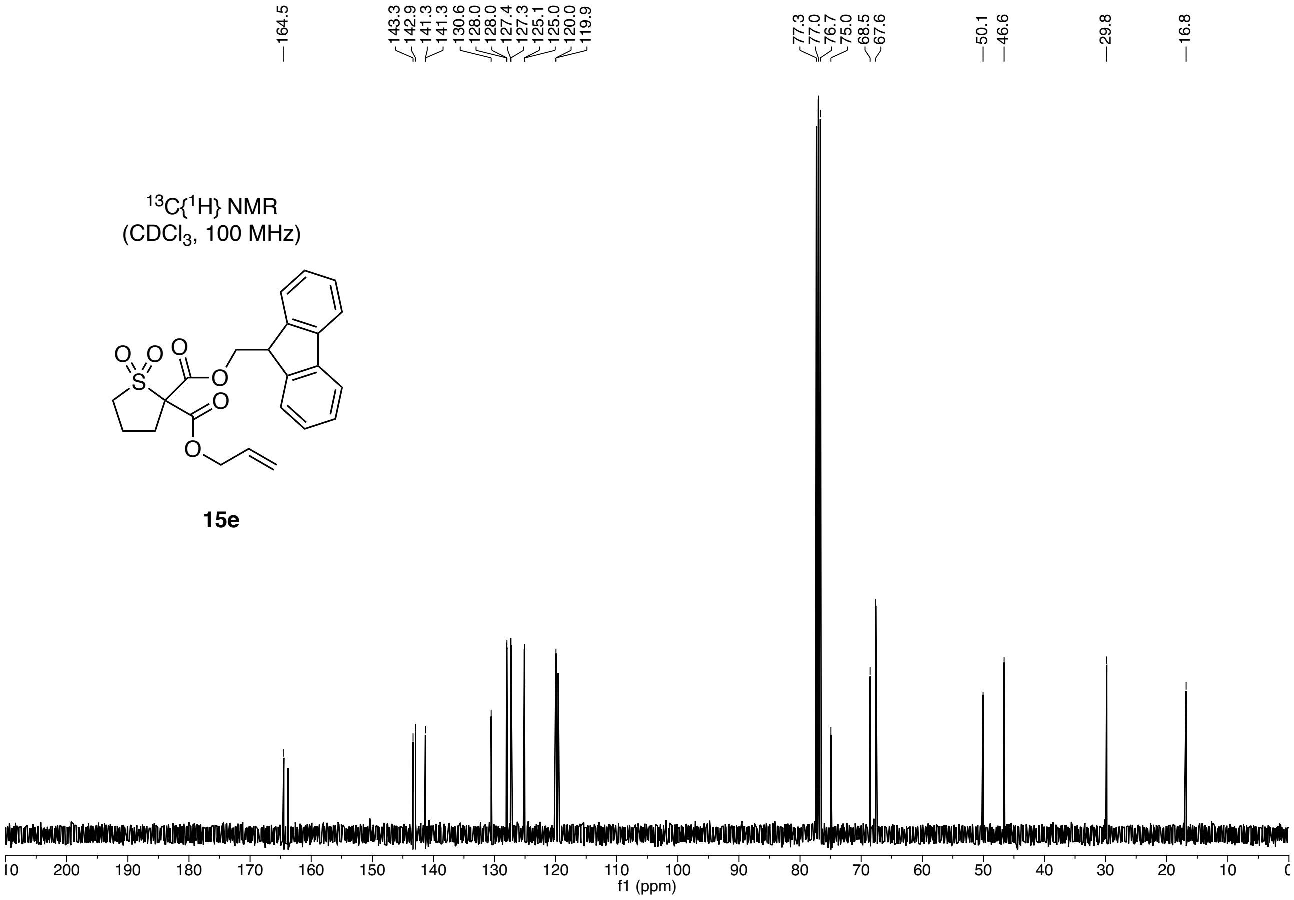
13C{1H} NMR
(CDCl3, 100 MHz)
15e
f1 (ppm)

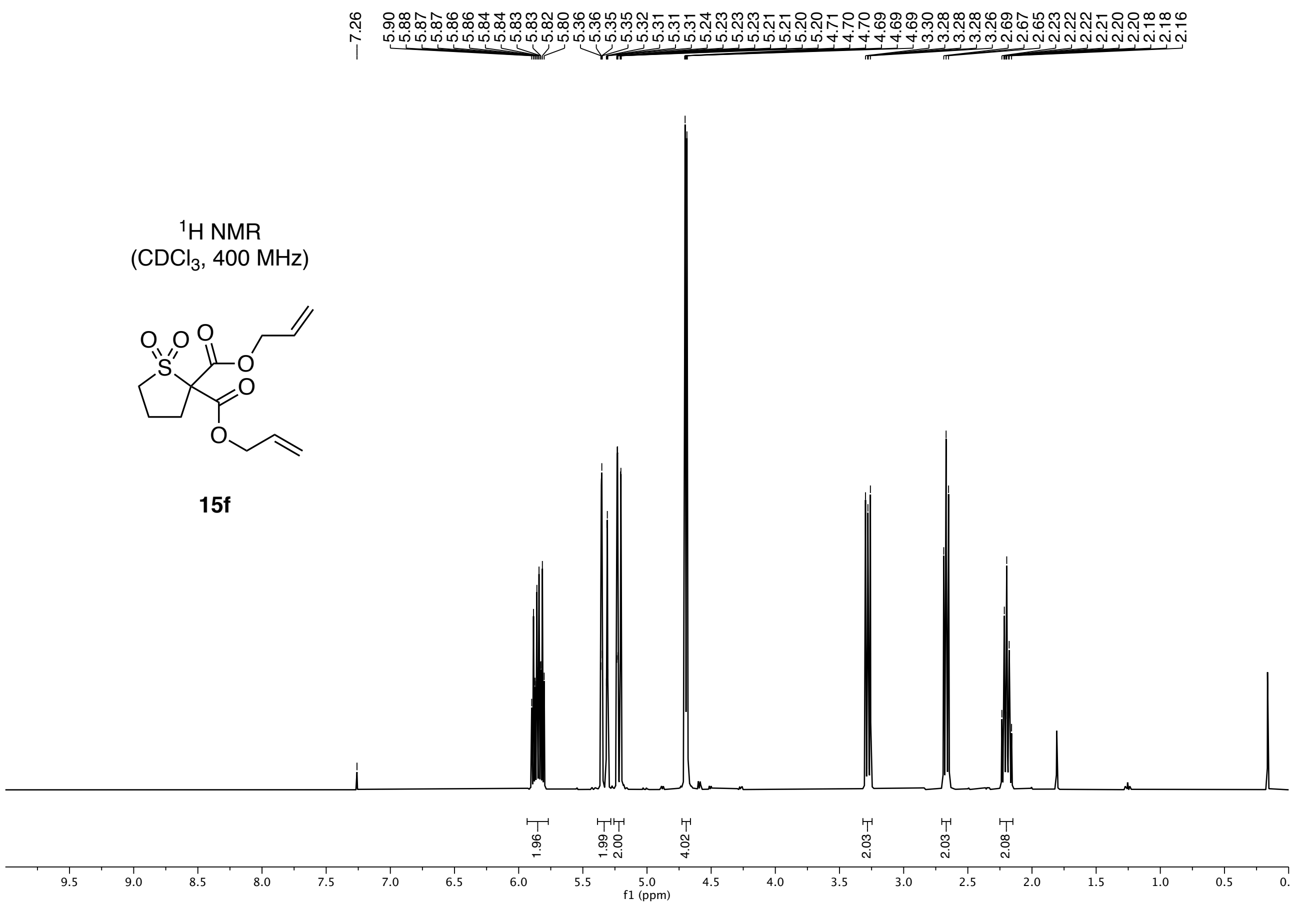

7.26
5.90
5.88
5.87
5.87
5.86
5.86
5.84
5.84
5.83
5.83
5.82
5.80
5.36
5.36
5.35
5.35
5.32
5.31
5.31
5.31
5.24
5.23
5.23
5.23
5.21
5.21
5.20
5.20
4.71
4.70
4.70
4.69
4.69
4.69
3.30
3.28
3.28
3.26
2.69
2.67
2.65
2.23
2.22
2.22
2.21
2.20
2.20
2.18
2.18
2.16
^{1}H NMR
($CDCl_3$, 400 MHz)
O O O
S
O
O
O
15f
1.96
1.99
2.00
4.02
2.03
2.03
2.08
9.5 9.0 8.5 8.0 7.5 7.0 6.5 6.0 5.5 5.0 4.5 4.0 3.5 3.0 2.5 2.0 1.5 1.0 0.5
f1 (ppm)

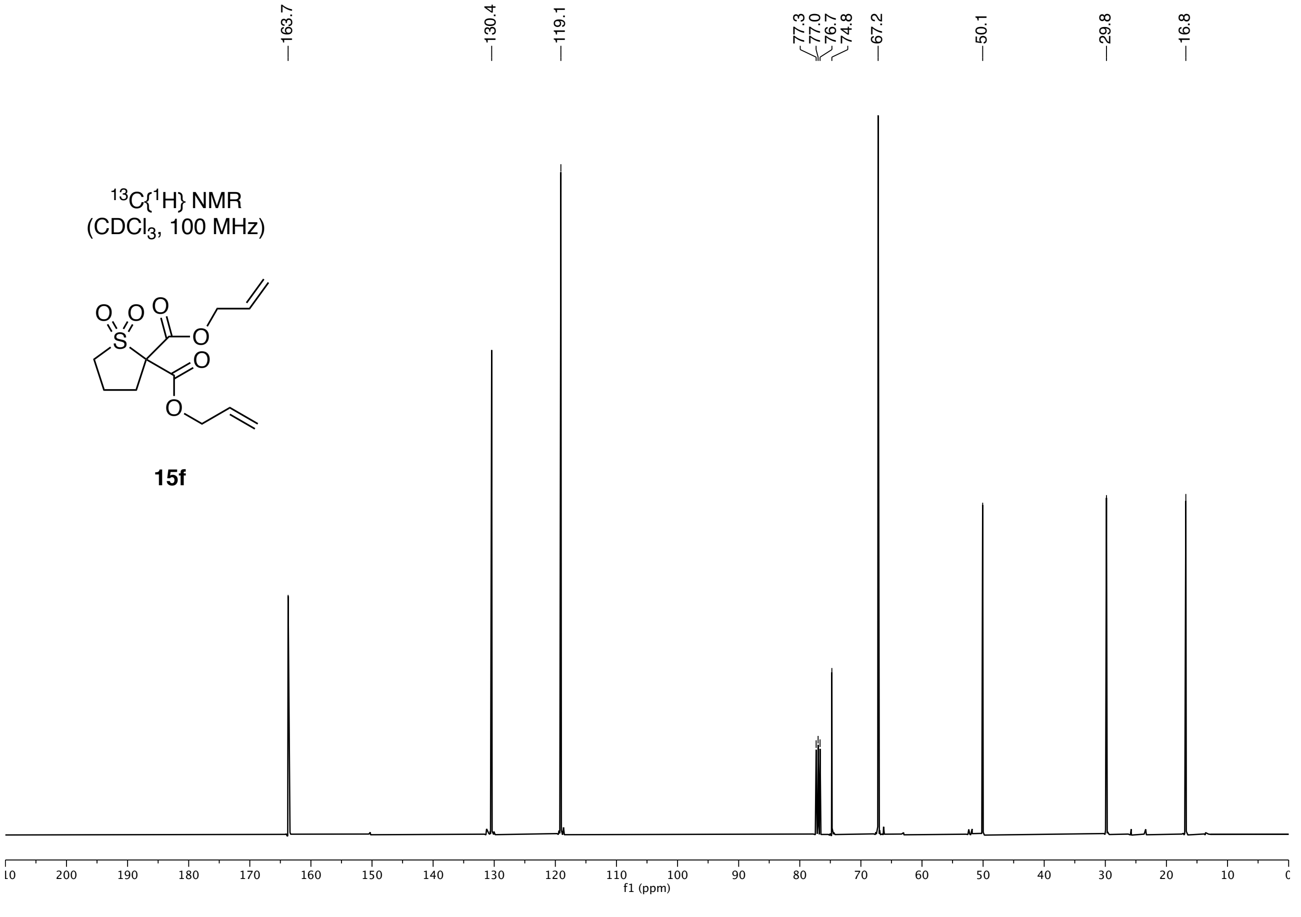

163.7
130.4
119.1
77.3
77.0
76.7
74.8
67.2
50.1
29.8
16.8
$^{13}C\{^1H\}$ NMR
($CDCl_3$, 100 MHz)
O O O
S
O
O
O
15f
200 190 180 170 160 150 140 130 120 110 100 90 80 70 60 50 40 30 20 10
f1 (ppm)

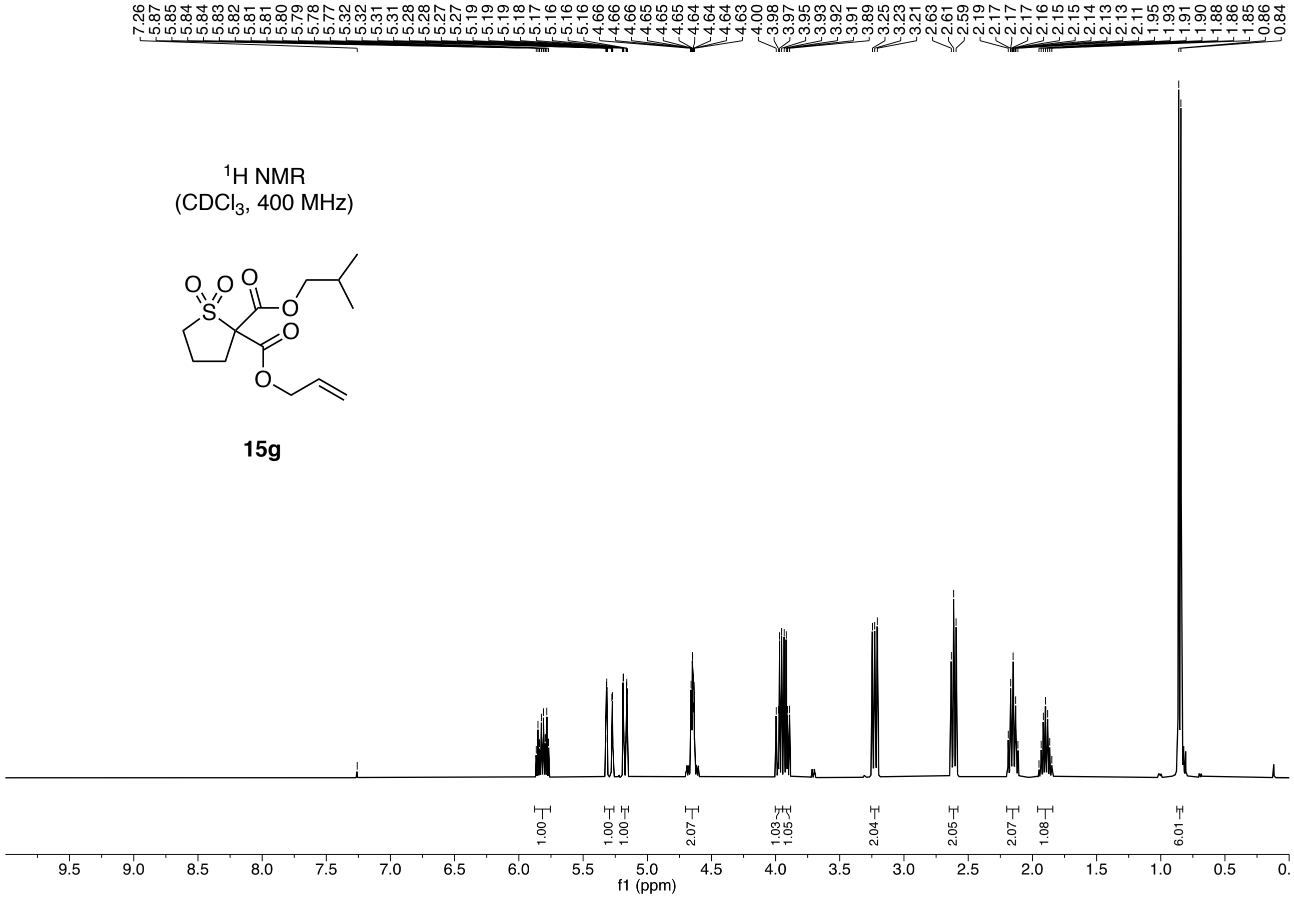
7.26 5.87 5.85 5.84 5.83 5.82 5.81 5.80 5.79 5.78 5.77 5.32 5.32 5.31 5.31 5.28 5.28 5.27 5.27 5.19 5.19 5.19 5.18 5.17 5.16 5.16 5.16 4.66 4.66 4.65 4.65 4.64 4.64 4.64 4.63 4.00 3.98 3.97 3.95 3.93 3.92 3.91 3.89 3.25 3.23 3.21 2.63 2.61 2.59 2.19 2.17 2.17 2.17 2.16 2.15 2.14 2.13 2.13 2.11 1.95 1.93 1.91 1.91 1.90 1.88 1.86 1.85 0.86 0.84
¹H NMR
(CDCl3, 400 MHz)
15g
1.00 1.00 1.00 2.07 1.03 1.05 2.04 2.05 2.07 1.08 6.01
f1 (ppm)

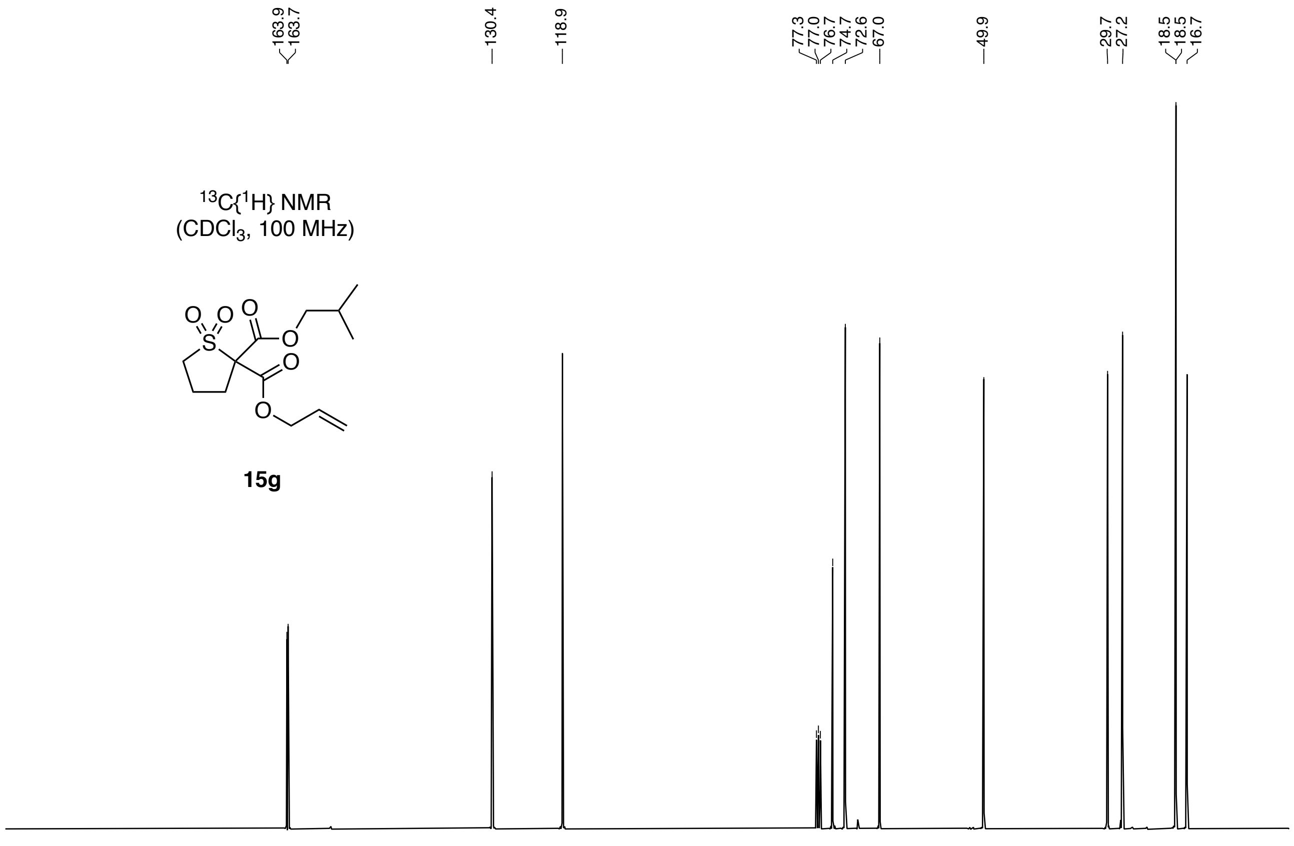
163.9 163.7 130.4 118.9 77.3 77.0 76.7 74.7 72.6 67.0 49.9 29.7 27.2 18.5 18.5 16.7
¹³C{¹H} NMR
(CDCl3, 100 MHz)
15g
f1 (ppm)

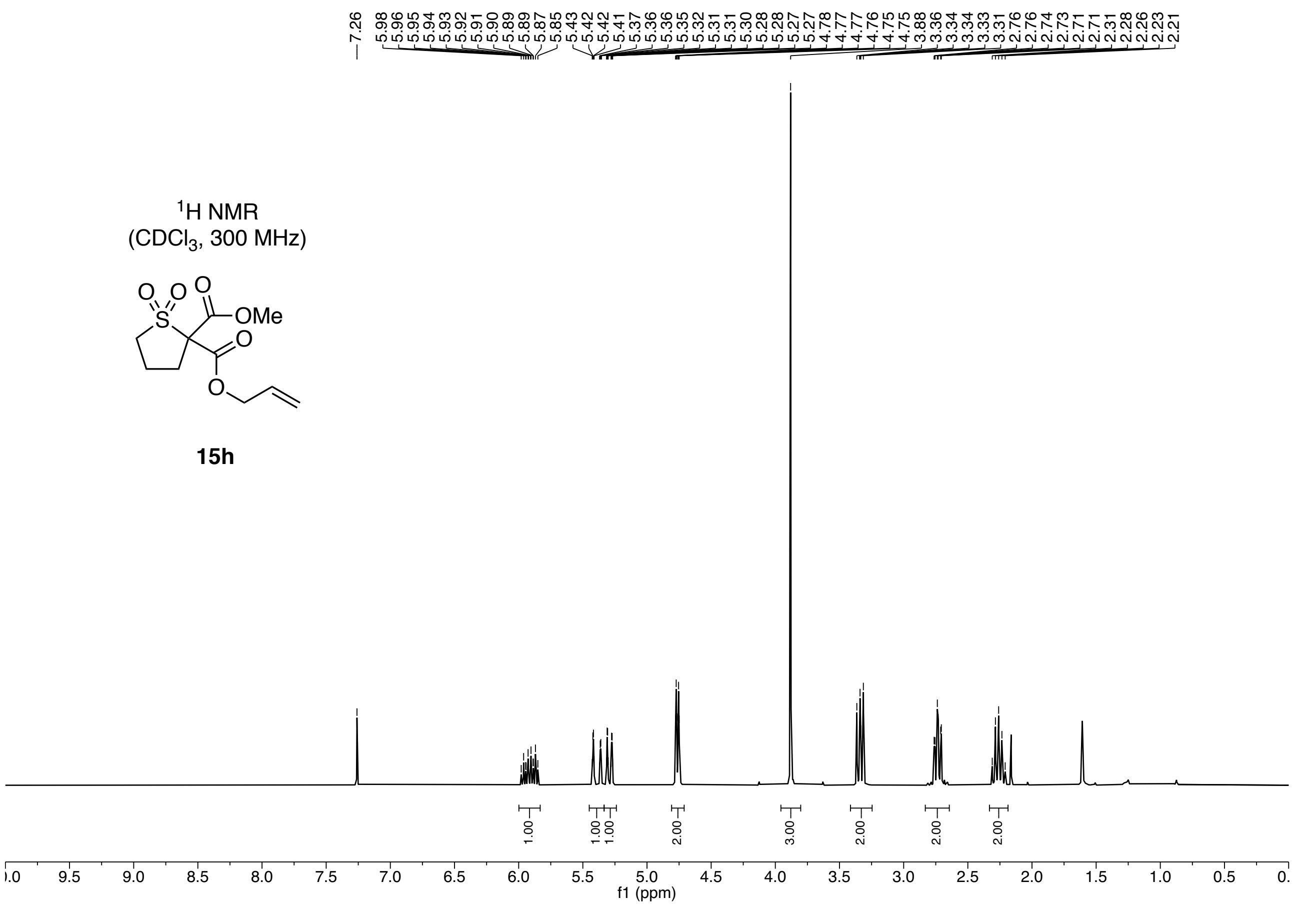

7.26
5.98 5.96 5.95 5.94 5.93 5.92 5.91 5.90 5.89 5.87 5.85 5.43 5.42 5.42 5.41 5.37 5.36 5.36 5.35 5.32 5.31 5.31 5.30 5.28 5.28 5.27 5.27 4.78 4.77 4.77 4.76 4.75 4.75 3.88 3.36 3.34 3.33 3.31 2.76 2.76 2.74 2.73 2.71 2.71 2.31 2.28 2.26 2.23 2.21
^{1}H NMR
(CDCl$_3$, 300 MHz)
O O O
S
OMe
O
O
15h
1.00
1.00
1.00
2.00
3.00
2.00
2.00
2.00
f1 (ppm)

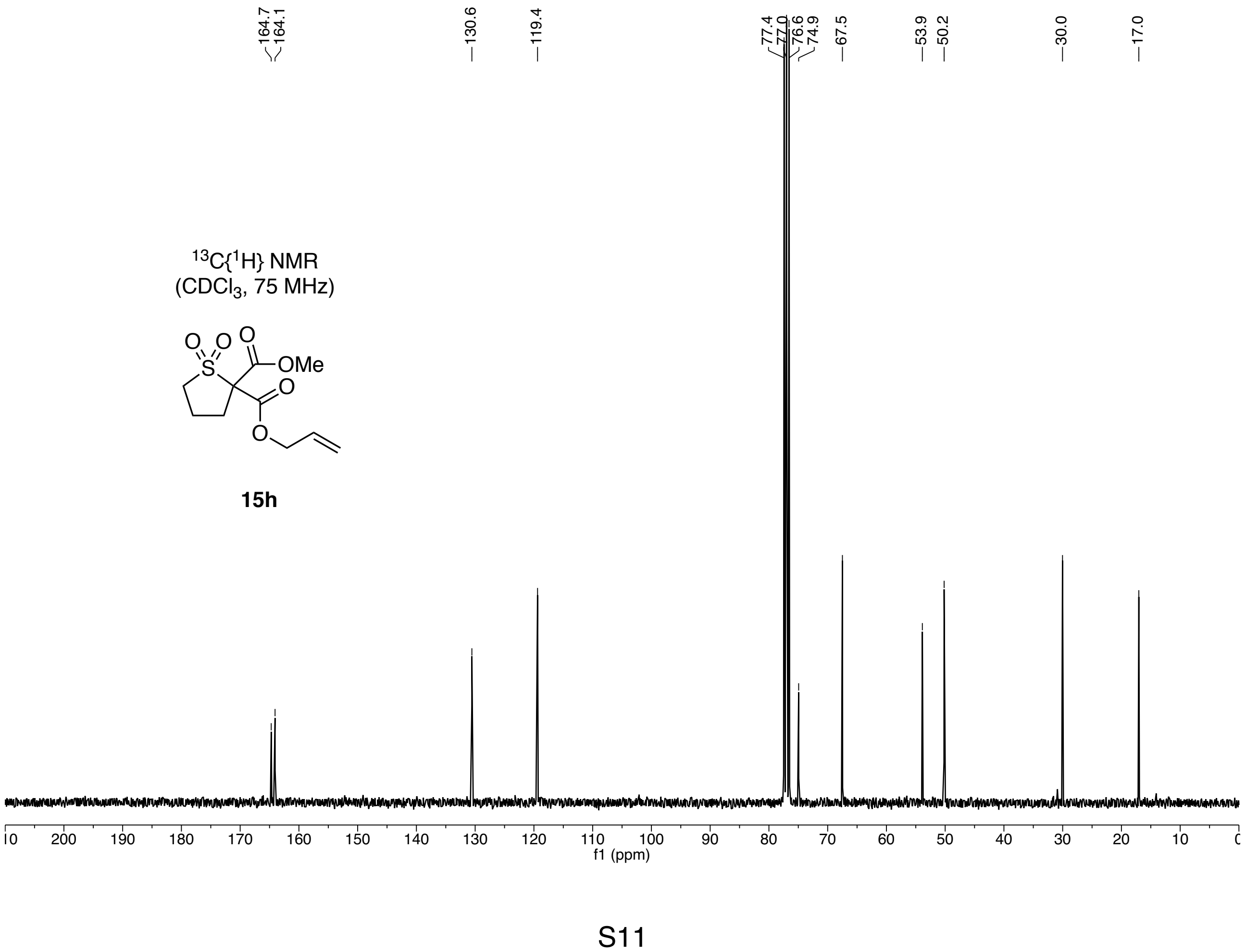

164.7
164.1
130.6
119.4
77.4
77.0
76.6
74.9
67.5
53.9
50.2
30.0
17.0
^{13}C{^{1}H} NMR
(CDCl$_3$, 75 MHz)
O O O
S
OMe
O
O
15h
f1 (ppm)

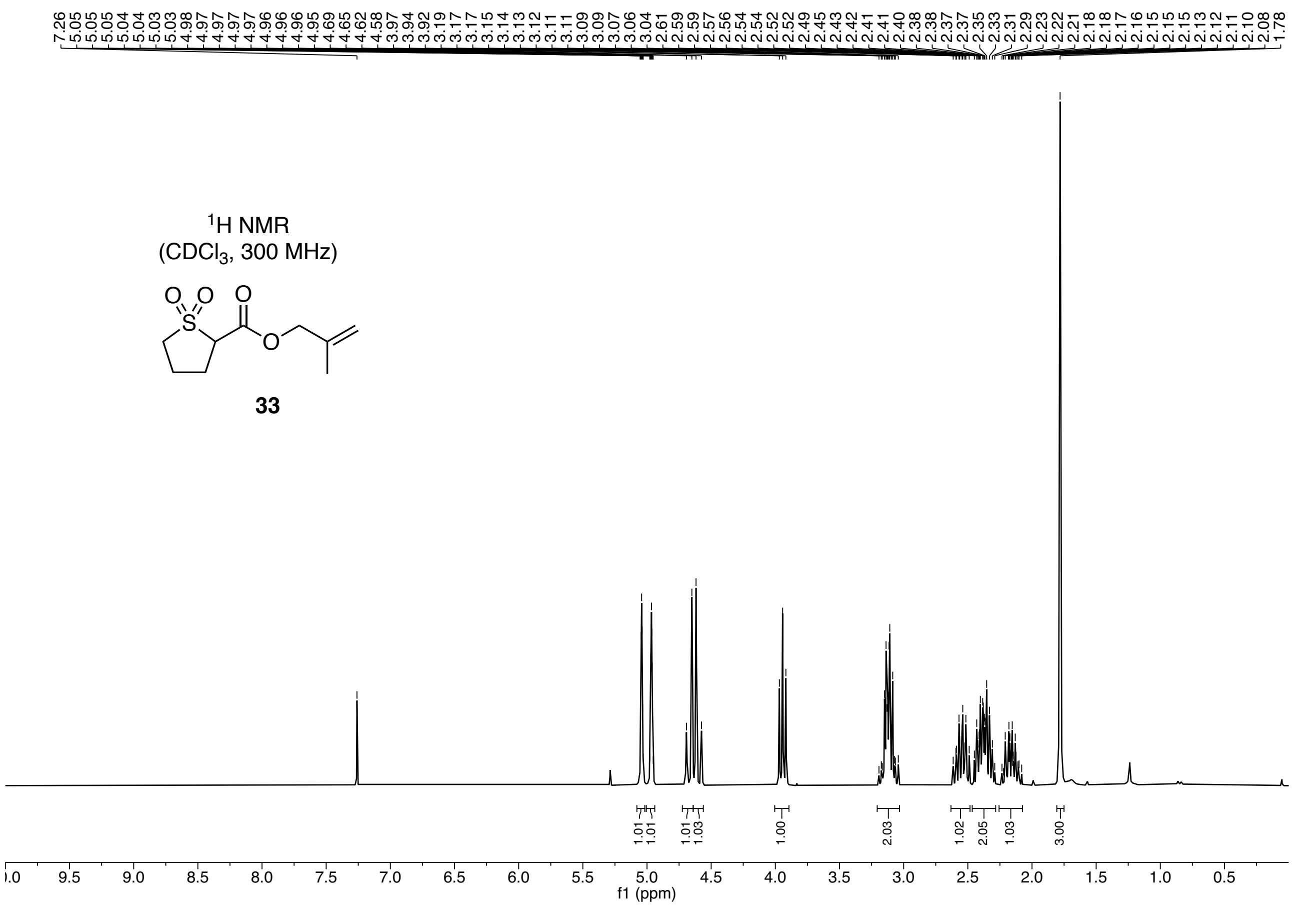

7.26 5.05 5.05 5.04 5.04 5.03 5.03 4.98 4.97 4.97 4.97 4.96 4.96 4.95 4.69 4.65 4.62 4.58 3.97 3.94 3.92 3.19 3.17 3.15 3.14 3.13 3.12 3.11 3.11 3.09 3.09 3.07 3.06 3.04 2.61 2.59 2.59 2.57 2.56 2.54 2.54 2.52 2.52 2.49 2.45 2.43 2.42 2.41 2.41 2.40 2.38 2.38 2.37 2.35 2.33 2.31 2.29 2.23 2.22 2.21 2.18 2.17 2.16 2.15 2.15 2.13 2.12 2.11 2.10 2.08 1.78
^{1}H NMR
(CDCl$_3$, 300 MHz)
33
1.01 1.01 1.01 1.03 1.00 2.03 1.02 2.05 1.03 3.00
f1 (ppm)

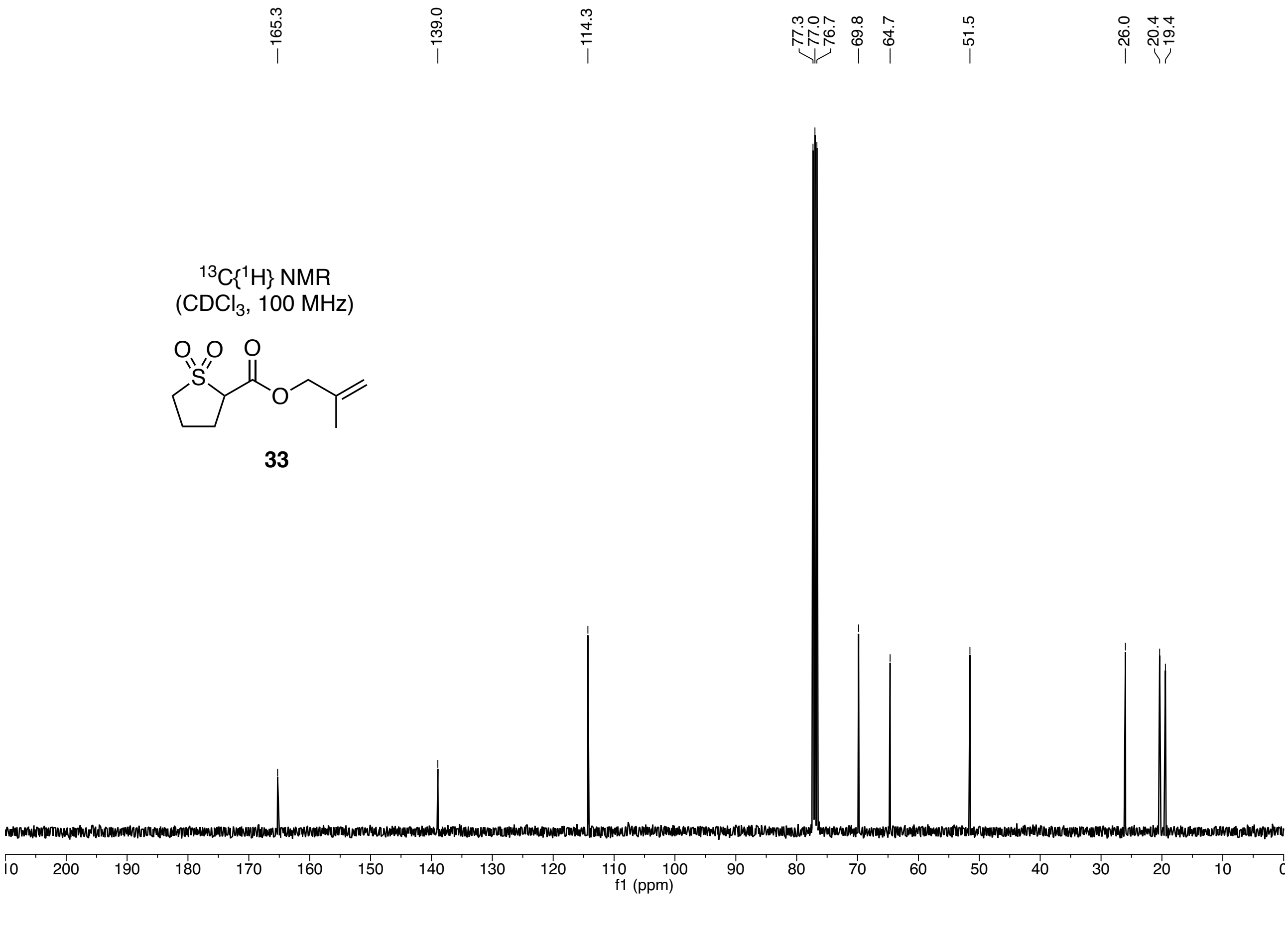

165.3 139.0 114.3 77.3 77.0 76.7 69.8 64.7 51.5 26.0 20.4 19.4
^{13}C{^{1}H} NMR
(CDCl$_3$, 100 MHz)
33
f1 (ppm)

^{1}H NMR
($CDCl_3$, 400 MHz)

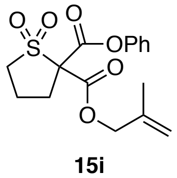

15i

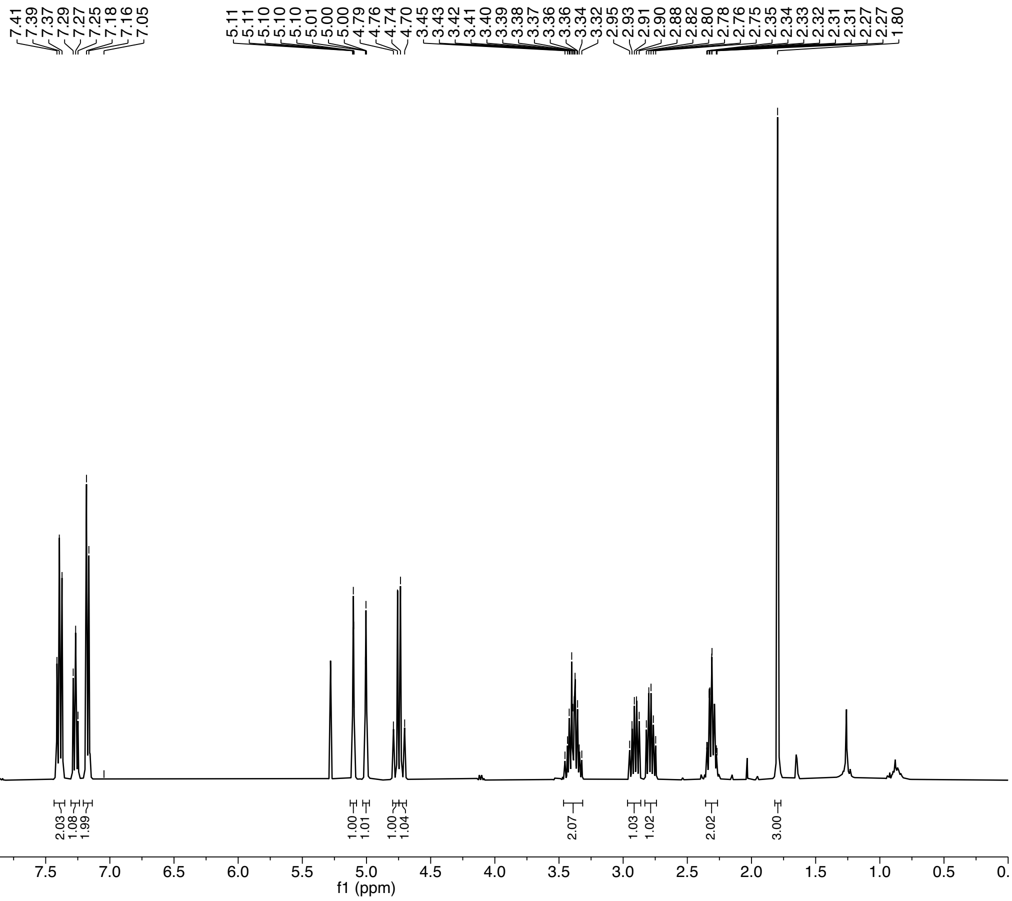

^{13}C{^{1}H} NMR
($CDCl_3$, 100 MHz)

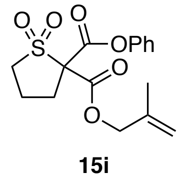

15i

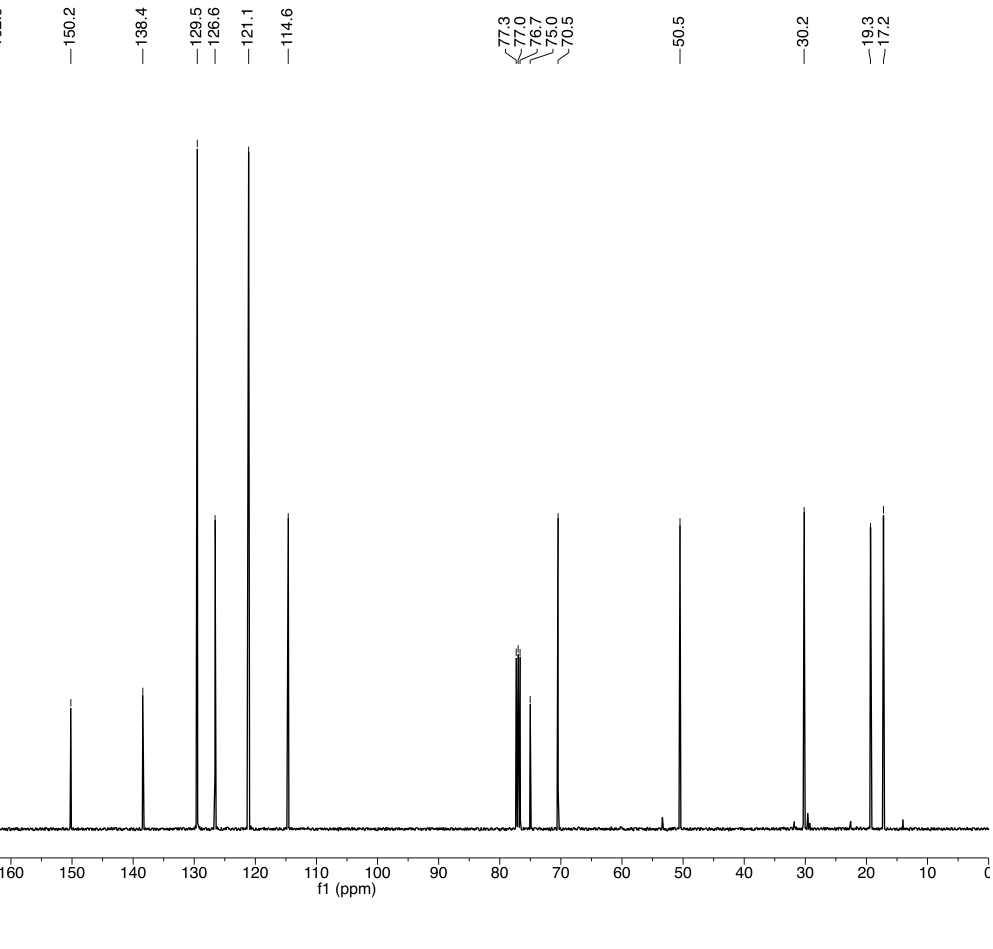

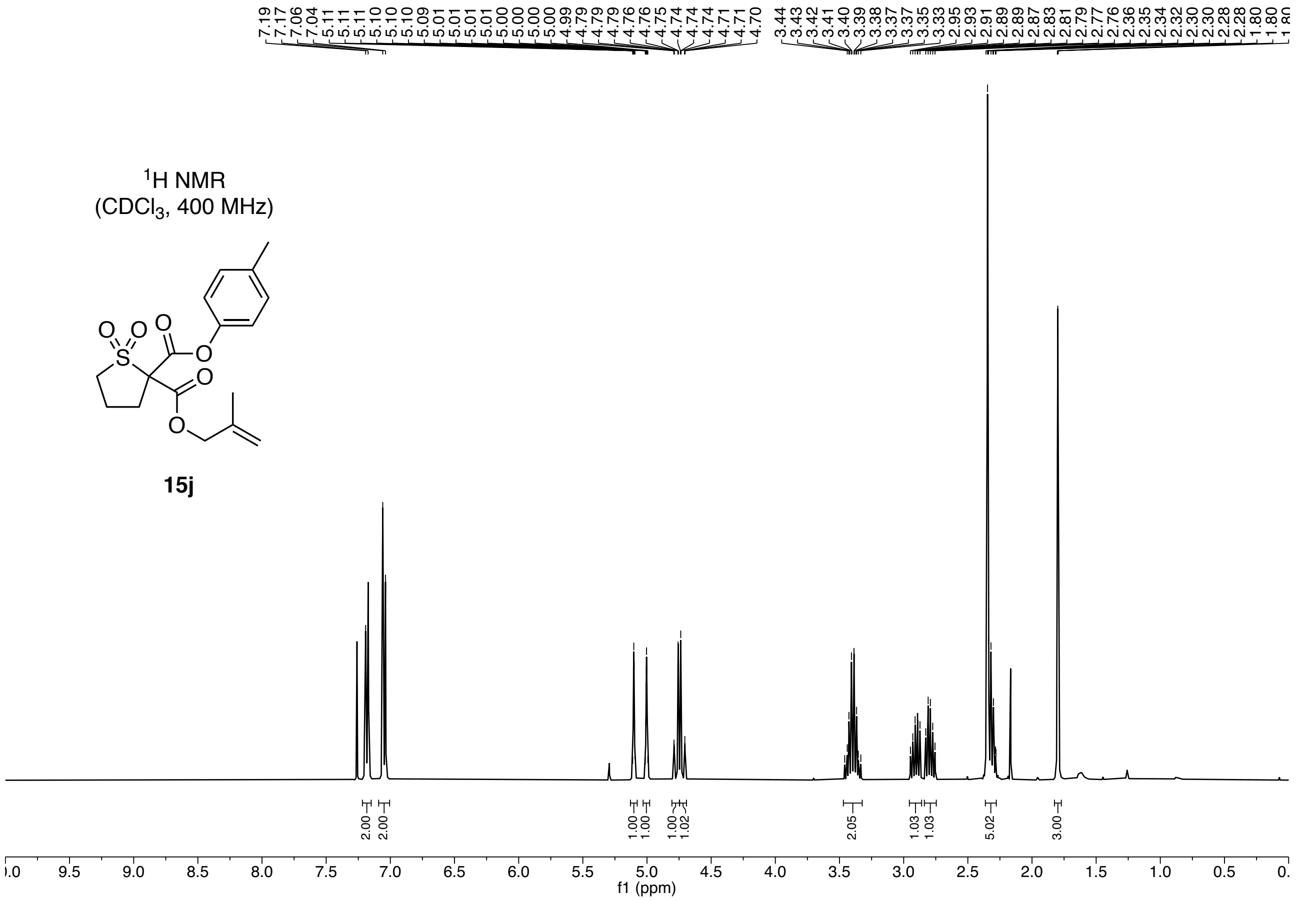
1H NMR
(CDCl3, 400 MHz)
15j
f1 (ppm)

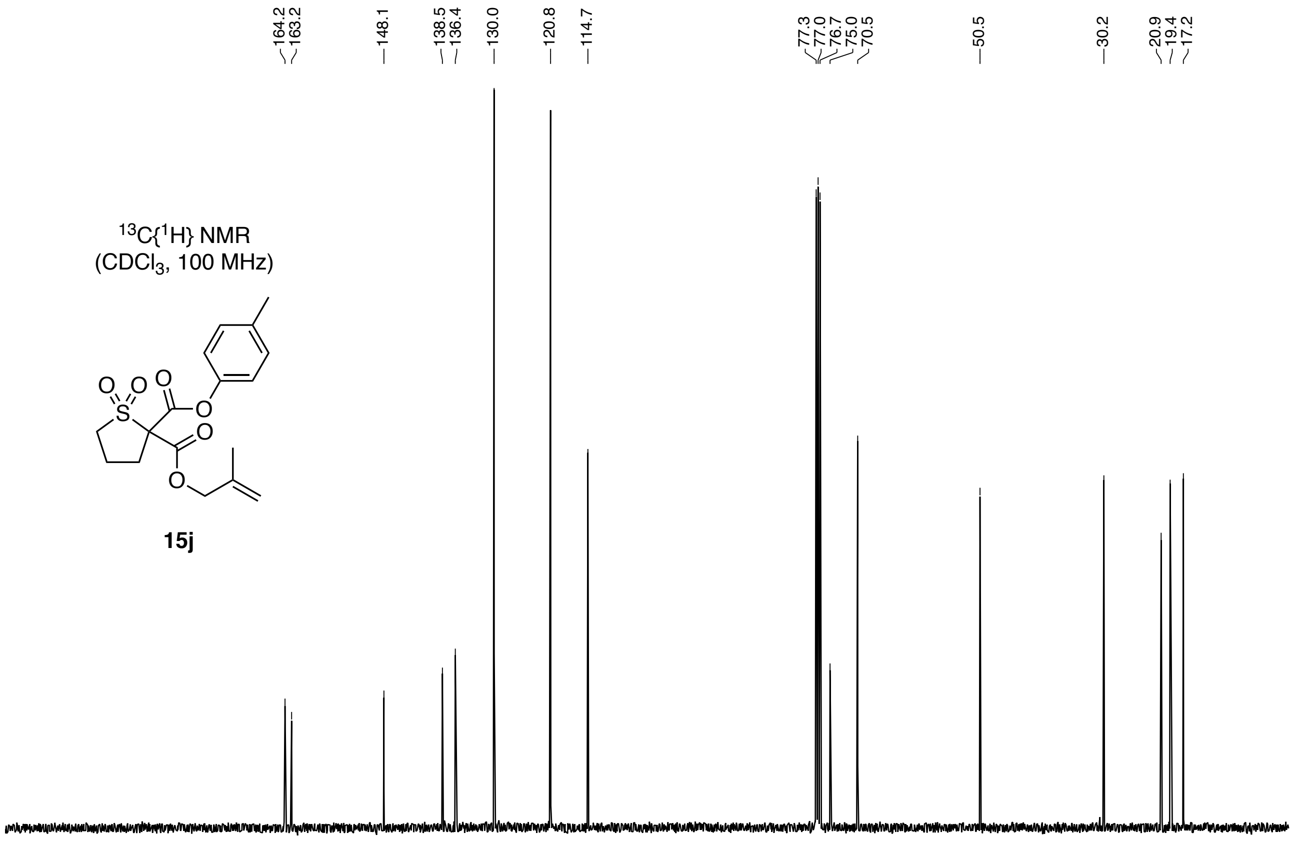
13C{1H} NMR
(CDCl3, 100 MHz)
164.2
163.2
148.1
138.5
136.4
130.0
120.8
114.7
77.3
77.0
76.7
75.0
70.5
50.5
30.2
20.9
19.4
17.2
15j
f1 (ppm)

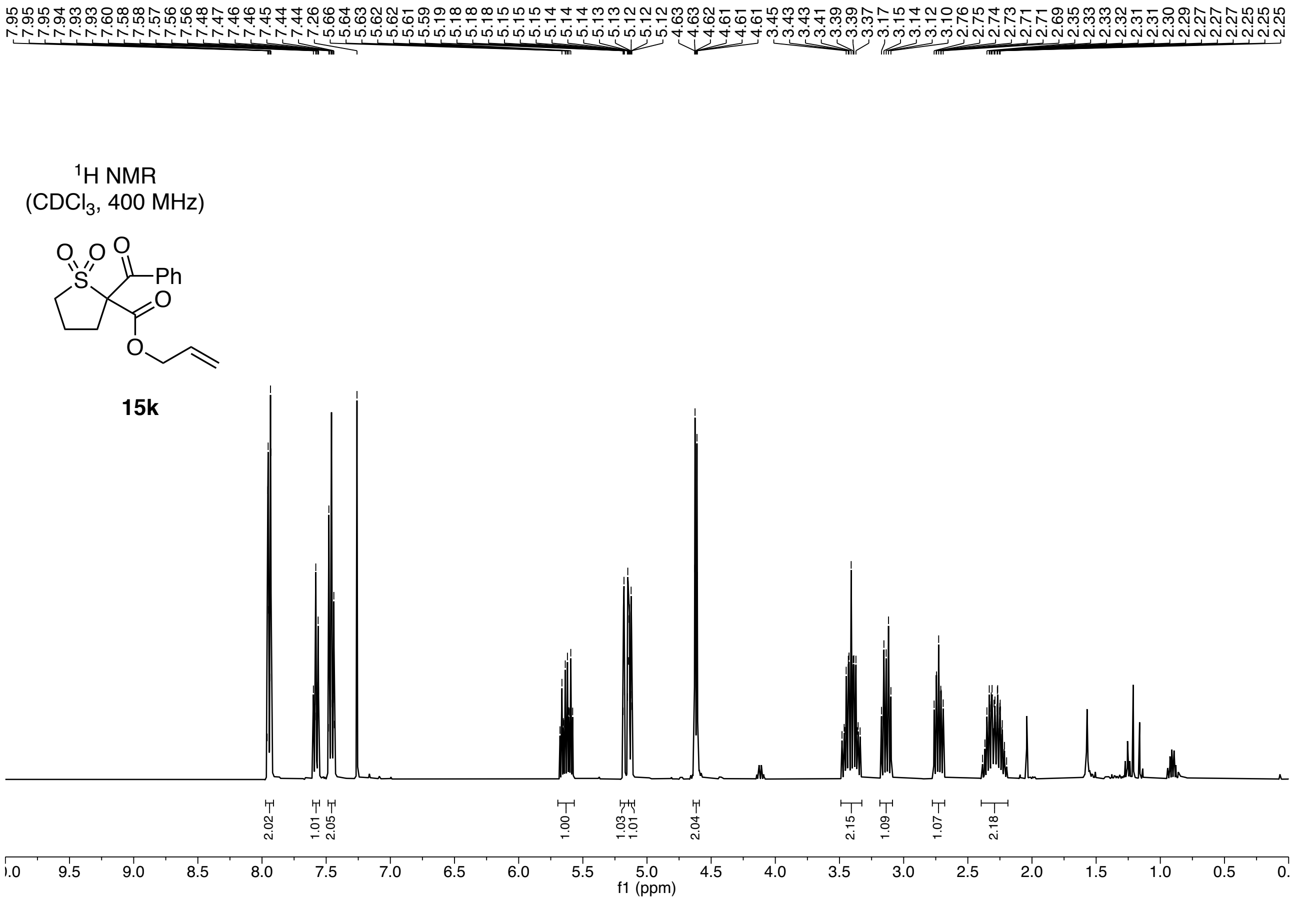
7.95 7.95 7.94 7.93 7.93 7.60 7.58 7.58 7.57 7.56 7.56 7.48 7.47 7.46 7.46 7.45 7.44 7.44 7.26 5.66 5.64 5.63 5.62 5.61 5.59 5.19 5.18 5.18 5.18 5.15 5.15 5.15 5.14 5.14 5.14 5.13 5.13 5.12 5.12 5.12 4.63 4.63 4.62 4.61 4.61 4.61 3.45 3.43 3.43 3.41 3.39 3.39 3.37 3.17 3.15 3.14 3.12 3.10 2.76 2.75 2.74 2.73 2.71 2.71 2.69 2.35 2.33 2.33 2.32 2.31 2.30 2.29 2.27 2.27 2.27 2.25 2.25 2.25
1H NMR
(CDCl3, 400 MHz)
O O O
S
Ph
O
O
15k
2.02 1.01 2.05 1.00 1.03 1.01 2.04 2.15 1.09 1.07 2.18
9.5 9.0 8.5 8.0 7.5 7.0 6.5 6.0 5.5 5.0 4.5 4.0 3.5 3.0 2.5 2.0 1.5 1.0 0.5
f1 (ppm)

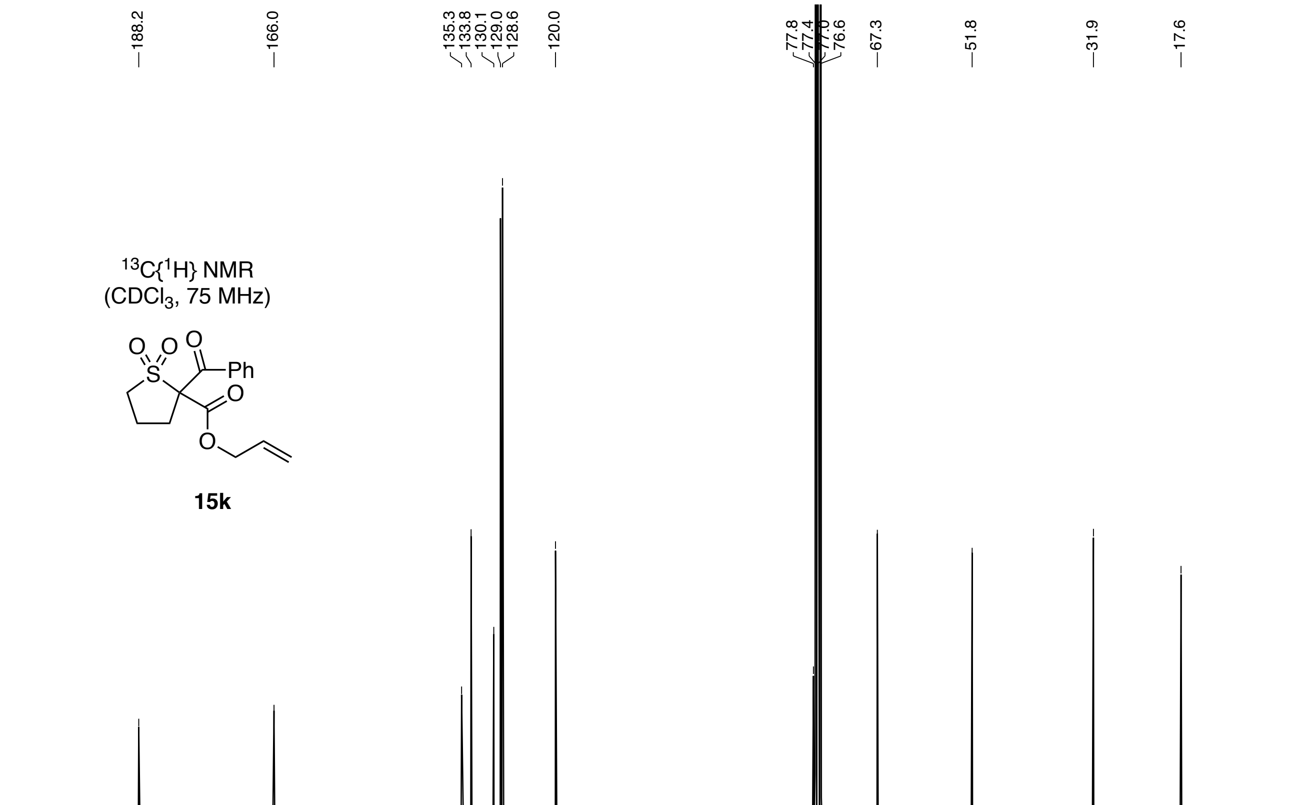
188.2 166.0 135.3 133.8 130.1 129.0 128.6 120.0 77.8 77.4 77.0 76.6 67.3 51.8 31.9 17.6
13C{1H} NMR
(CDCl3, 75 MHz)
O O O
S
Ph
O
O
15k
200 190 180 170 160 150 140 130 120 110 100 90 80 70 60 50 40 30 20 10
f1 (ppm)

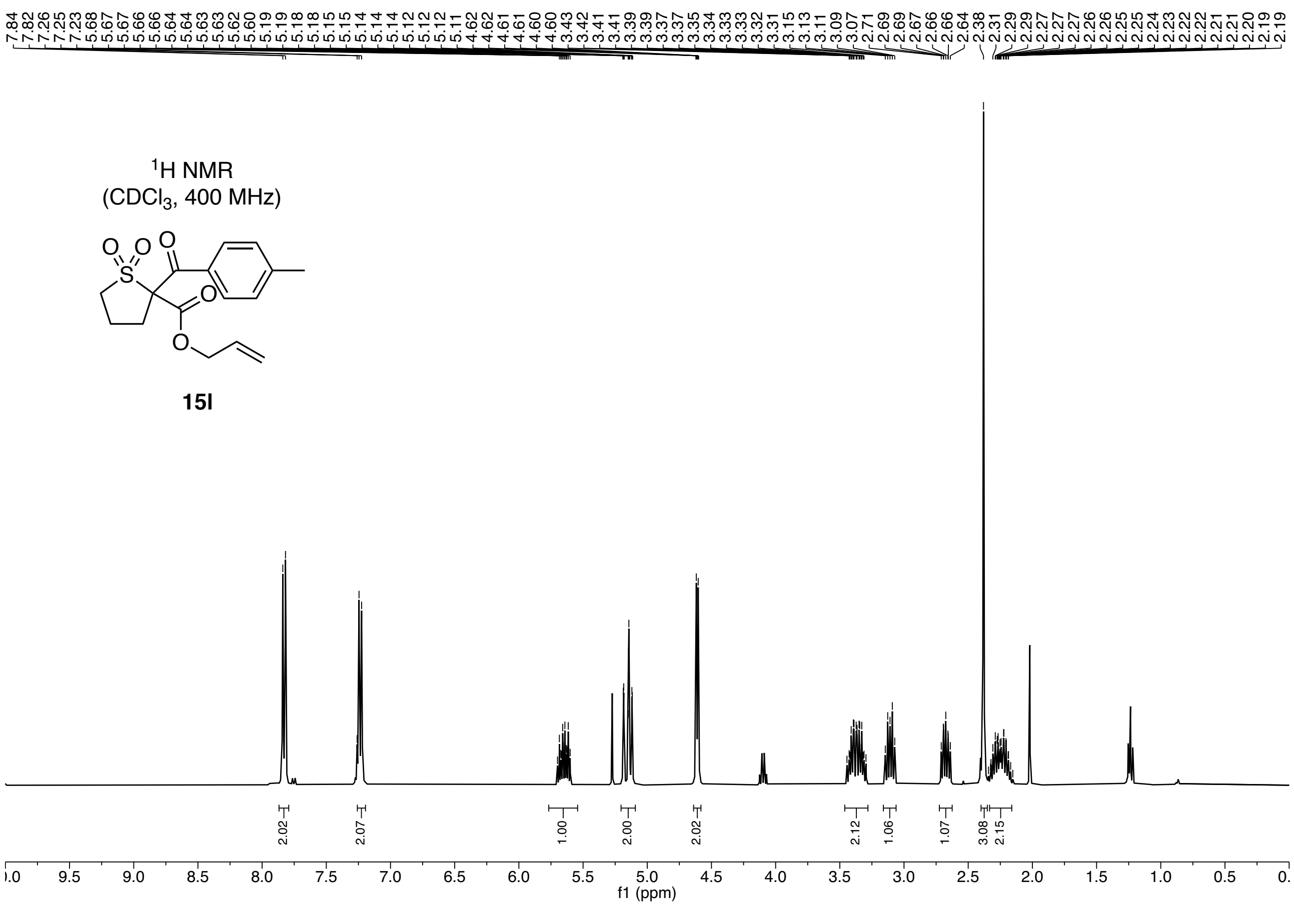

7.84 7.82 7.26 7.25 7.23 5.68 5.67 5.66 5.66 5.64 5.63 5.63 5.62 5.60 5.19 5.19 5.18 5.15 5.15 5.14 5.14 5.14 5.12 5.12 5.11 4.62 4.62 4.61 4.61 4.60 3.43 3.42 3.41 3.41 3.39 3.39 3.37 3.37 3.35 3.34 3.33 3.33 3.32 3.31 3.15 3.13 3.11 3.09 3.07 2.71 2.69 2.69 2.67 2.66 2.66 2.64 2.38 2.31 2.29 2.29 2.27 2.27 2.27 2.26 2.26 2.25 2.25 2.24 2.23 2.22 2.22 2.21 2.21 2.20 2.19 2.19
1H NMR
(CDCl3, 400 MHz)
15l
2.02 2.07 1.00 2.00 2.02 2.12 1.06 1.07 3.08 2.15
f1 (ppm)

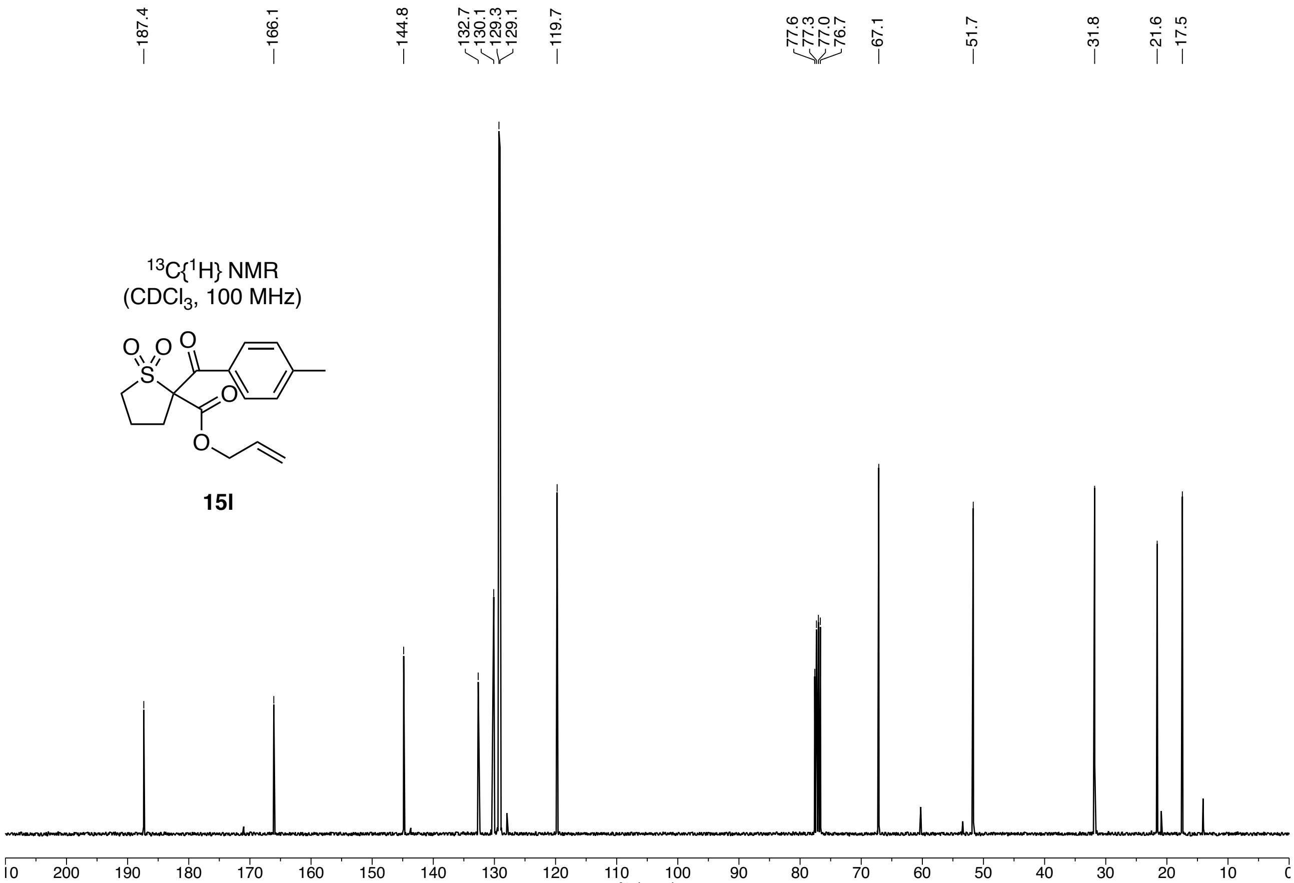

187.4 166.1 144.8 132.7 130.1 129.3 129.1 119.7 77.6 77.3 77.0 76.7 67.1 51.7 31.8 21.6 17.5
13C{1H} NMR
(CDCl3, 100 MHz)
15l
f1 (ppm)

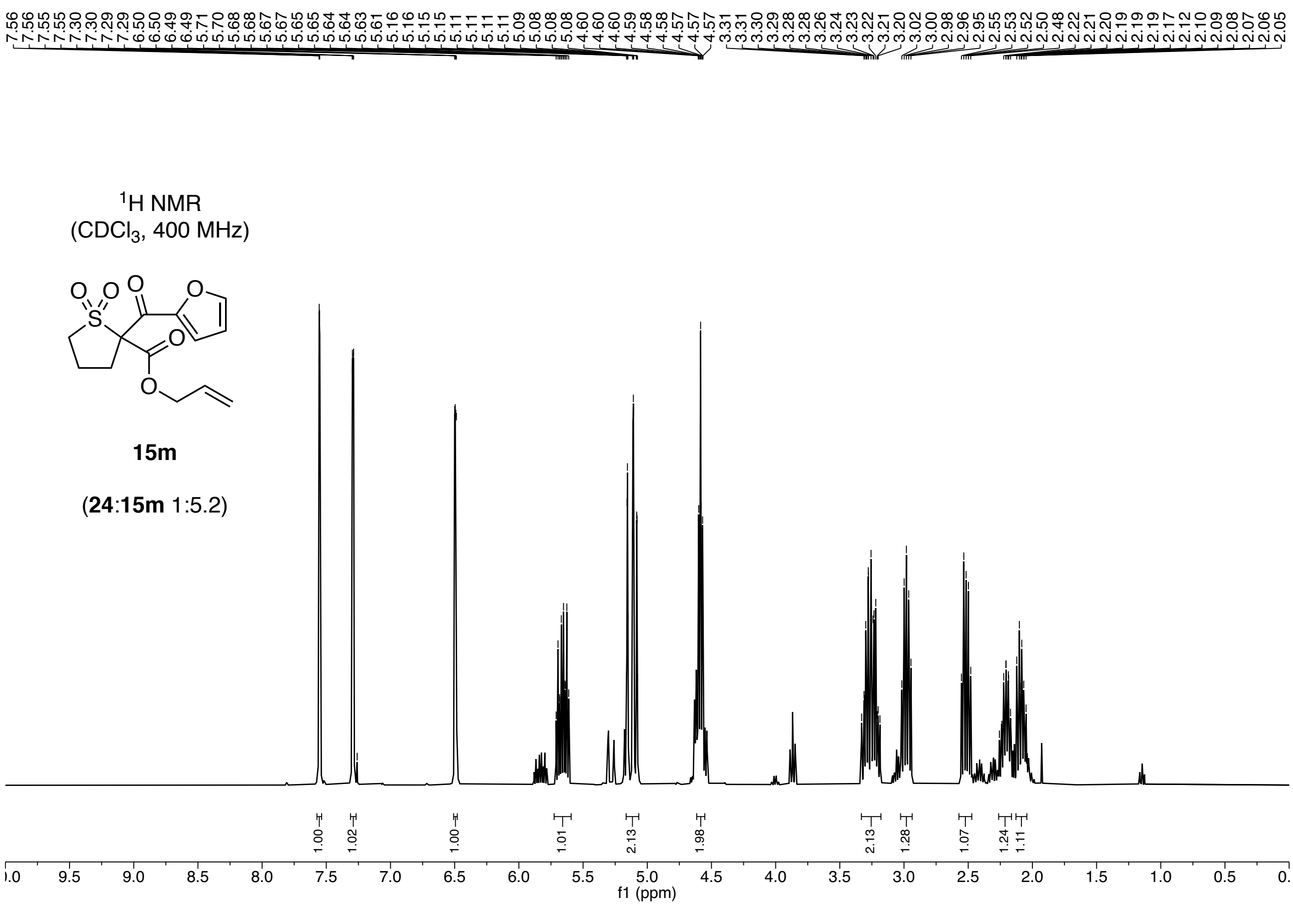
7.56 7.56 7.55 7.55 7.30 7.29 7.29 6.50 6.50 6.49 6.49 5.71 5.70 5.68 5.68 5.67 5.67 5.65 5.64 5.64 5.63 5.61 5.16 5.16 5.15 5.15 5.11 5.11 5.11 5.11 5.09 5.08 5.08 5.08 4.60 4.60 4.59 4.58 4.58 4.57 4.57 4.57 3.31 3.30 3.29 3.28 3.26 3.24 3.23 3.22 3.21 3.20 3.02 3.00 2.98 2.96 2.95 2.55 2.53 2.52 2.50 2.48 2.22 2.21 2.20 2.19 2.19 2.17 2.12 2.10 2.09 2.08 2.06 2.05
^{1}H NMR
($CDCl_3$, 400 MHz)
15m
(24:15m 1:5.2)
1.00 1.02 1.00 1.01 2.13 1.98 2.13 1.28 1.07 1.24 1.11
f1 (ppm)

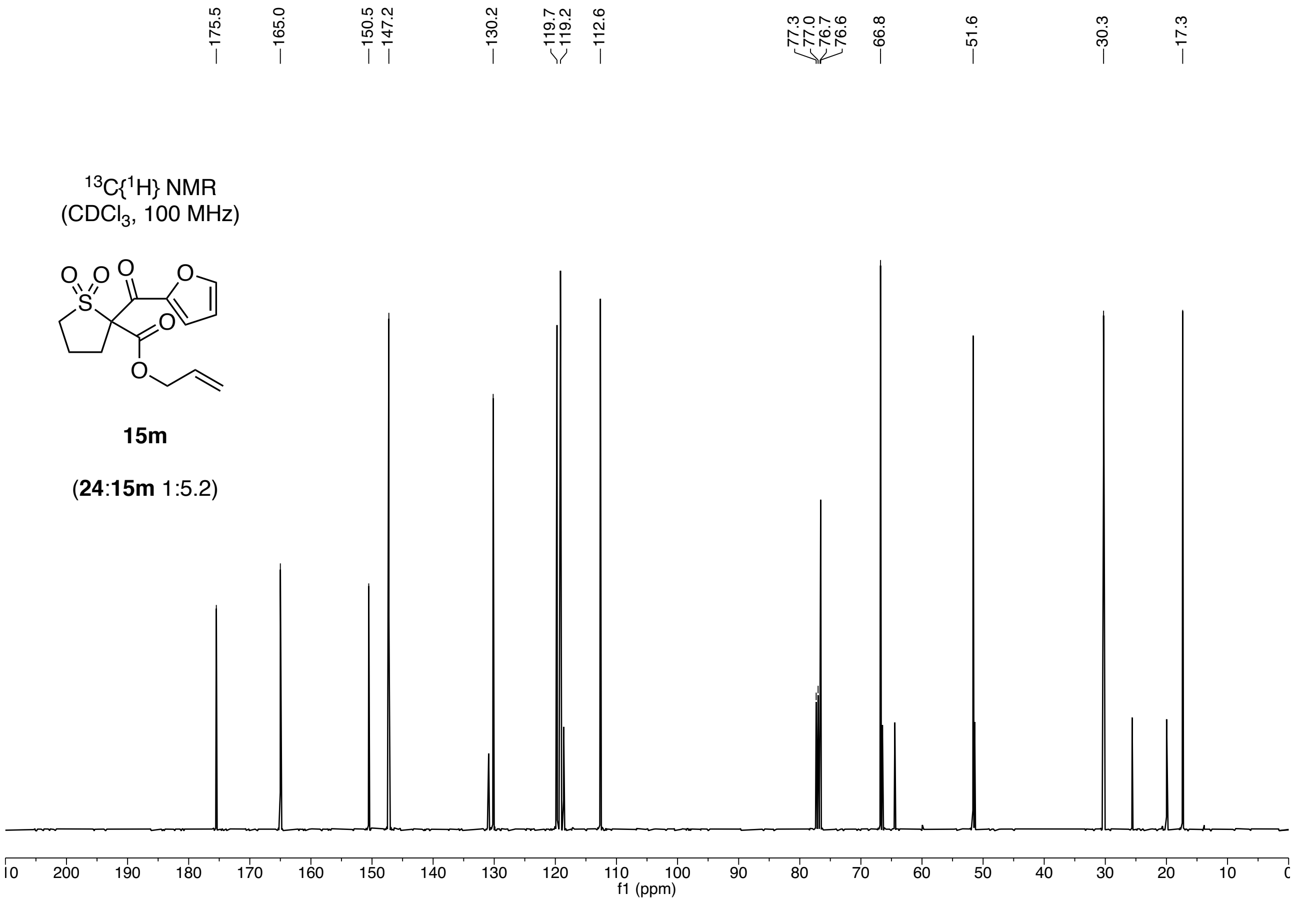
175.5 165.0 150.5 147.2 130.2 119.7 119.2 112.6 77.3 77.0 76.7 76.6 66.8 51.6 30.3 17.3
$^{13}C\{^1H\}$ NMR
($CDCl_3$, 100 MHz)
15m
(24:15m 1:5.2)
f1 (ppm)

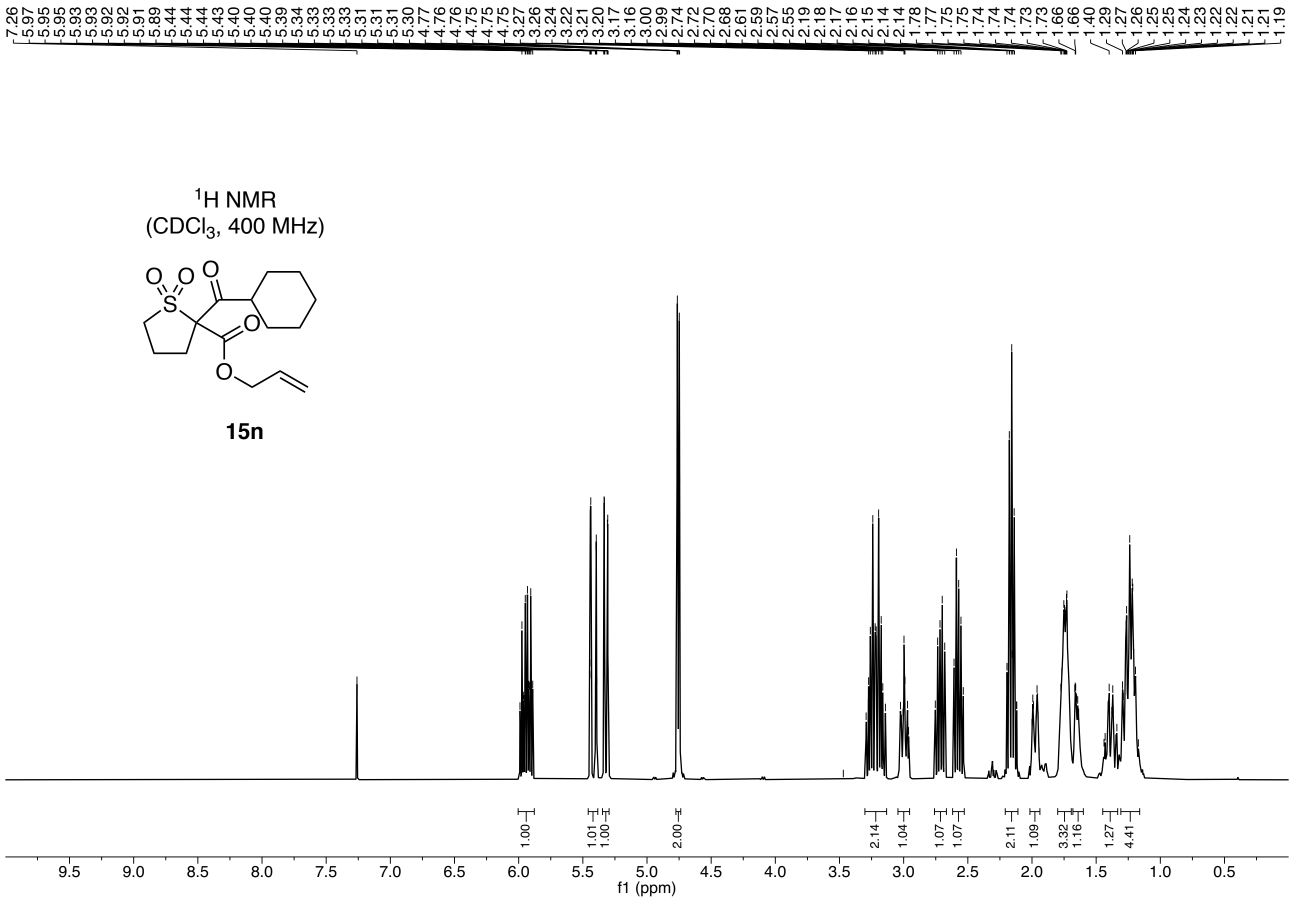
7.26 5.97 5.95 5.95 5.93 5.93 5.92 5.92 5.91 5.89 5.44 5.44 5.44 5.43 5.40 5.40 5.39 5.34 5.33 5.33 5.33 5.31 5.31 5.31 5.30 4.77 4.76 4.76 4.75 4.75 4.75 3.27 3.26 3.24 3.22 3.21 3.20 3.17 3.16 3.00 2.99 2.74 2.72 2.72 2.70 2.68 2.61 2.59 2.57 2.55 2.19 2.18 2.17 2.16 2.15 2.14 2.14 1.78 1.77 1.75 1.75 1.74 1.74 1.74 1.73 1.73 1.66 1.66 1.40 1.29 1.27 1.26 1.25 1.25 1.24 1.23 1.22 1.22 1.21 1.21 1.19
¹H NMR
(CDCl₃, 400 MHz)
15n
1.00 1.01 1.00 2.00 2.14 1.04 1.07 1.07 2.11 1.09 3.32 1.16 1.27 4.41
9.5 9.0 8.5 8.0 7.5 7.0 6.5 6.0 5.5 5.0 4.5 4.0 3.5 3.0 2.5 2.0 1.5 1.0 0.5
f1 (ppm)

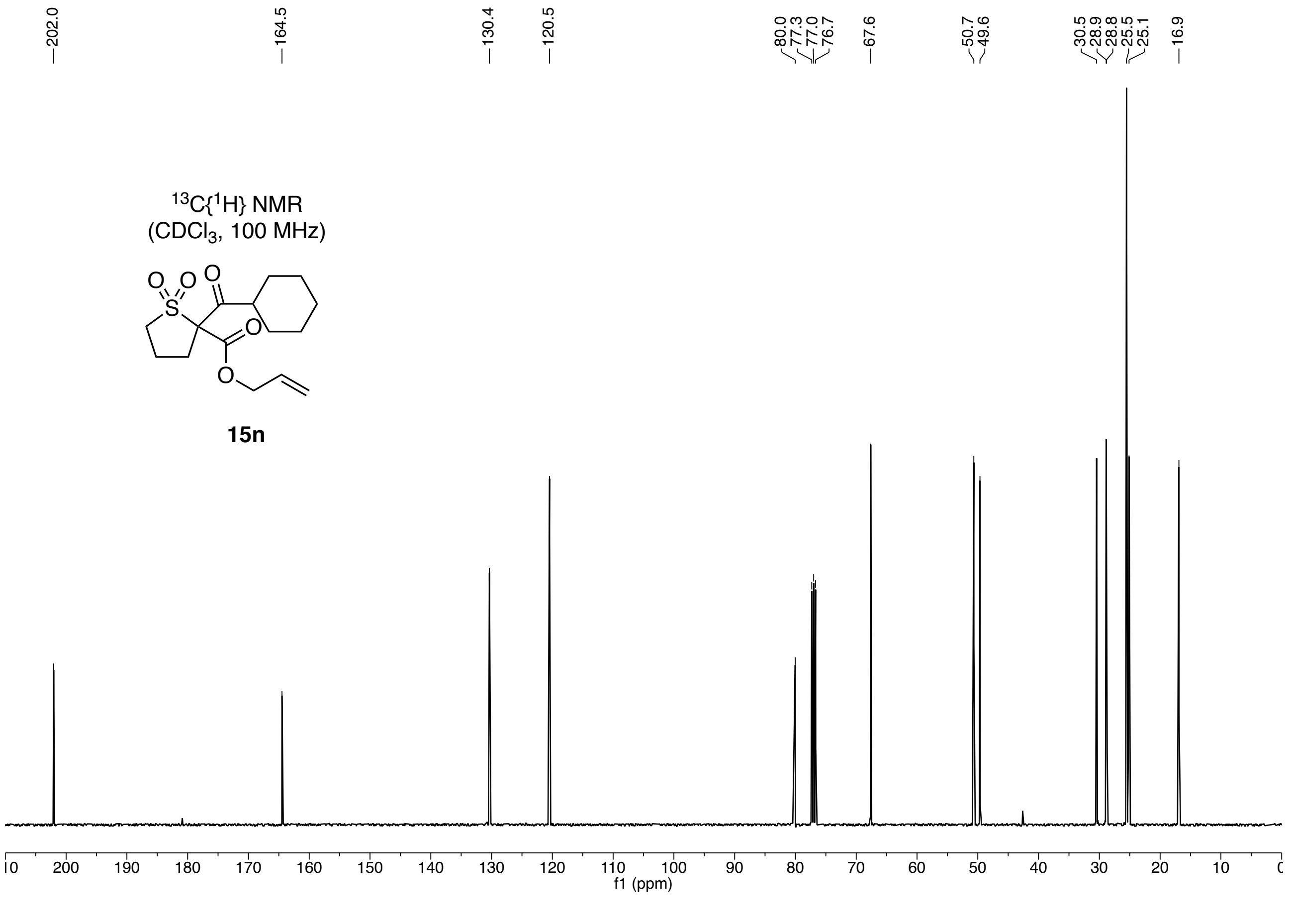
202.0 164.5 130.4 120.5 80.0 77.3 77.0 76.7 67.6 50.7 49.6 30.5 28.9 28.8 25.5 25.1 16.9
¹³C{¹H} NMR
(CDCl₃, 100 MHz)
15n
200 190 180 170 160 150 140 130 120 110 100 90 80 70 60 50 40 30 20 10
f1 (ppm)

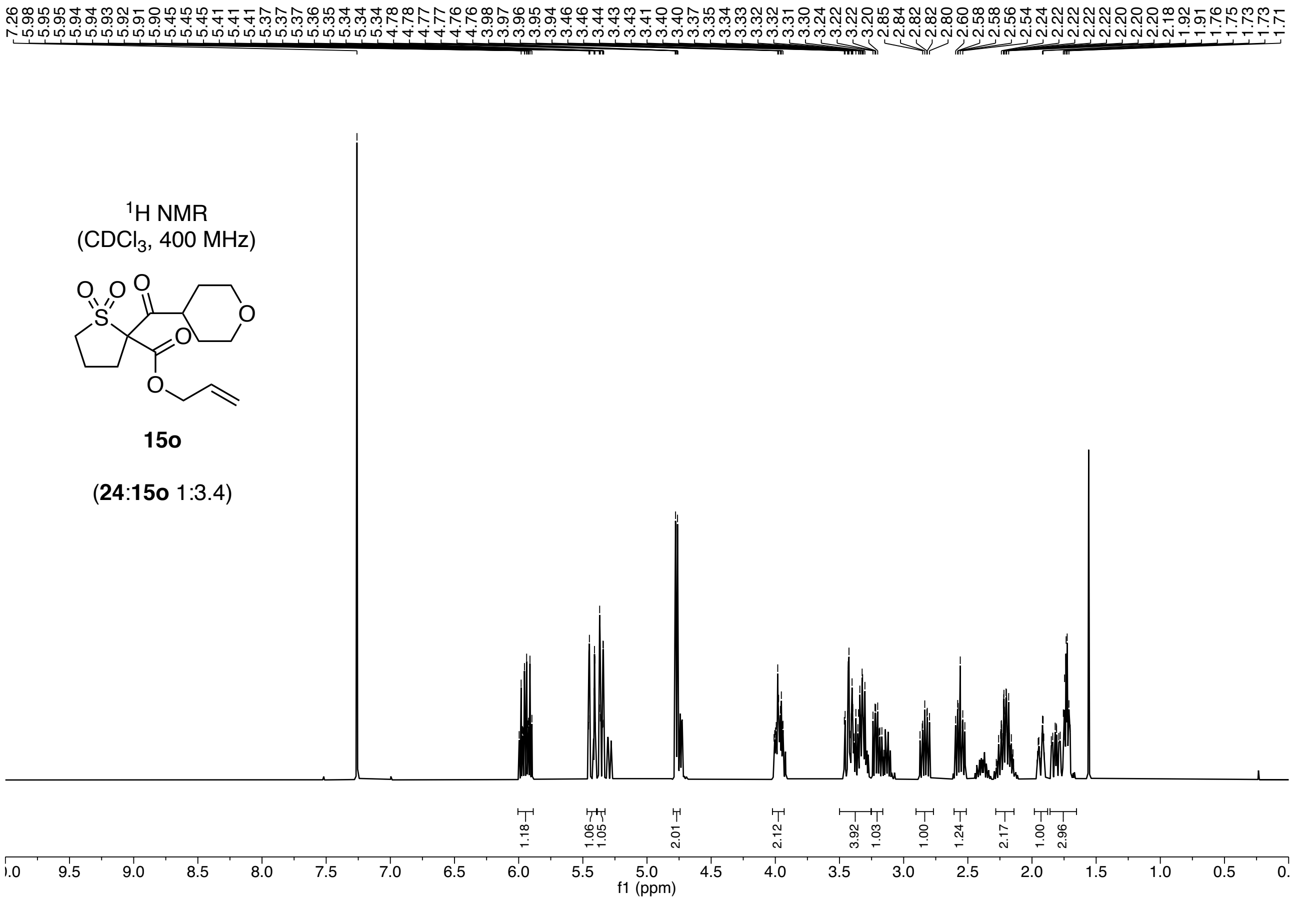

7.26 5.98 5.95 5.94 5.94 5.93 5.92 5.91 5.90 5.45 5.45 5.41 5.41 5.37 5.37 5.36 5.35 5.34 5.34 5.34 4.78 4.77 4.77 4.76 3.98 3.97 3.96 3.95 3.94 3.46 3.46 3.44 3.43 3.43 3.41 3.40 3.37 3.35 3.34 3.33 3.32 3.32 3.31 3.30 3.24 3.22 3.22 3.20 2.85 2.84 2.82 2.82 2.80 2.60 2.58 2.58 2.56 2.54 2.24 2.22 2.22 2.22 2.22 2.20 2.20 2.20 2.18 1.92 1.91 1.76 1.75 1.73 1.71
¹H NMR
(CDCl3, 400 MHz)
O O O
S
O
O
O
15o
(24:15o 1:3.4)
1.18 1.06 1.05 2.01 2.12 3.92 1.03 1.00 1.24 2.17 1.00 2.96
9.5 9.0 8.5 8.0 7.5 7.0 6.5 6.0 5.5 5.0 4.5 4.0 3.5 3.0 2.5 2.0 1.5 1.0 0.5
f1 (ppm)

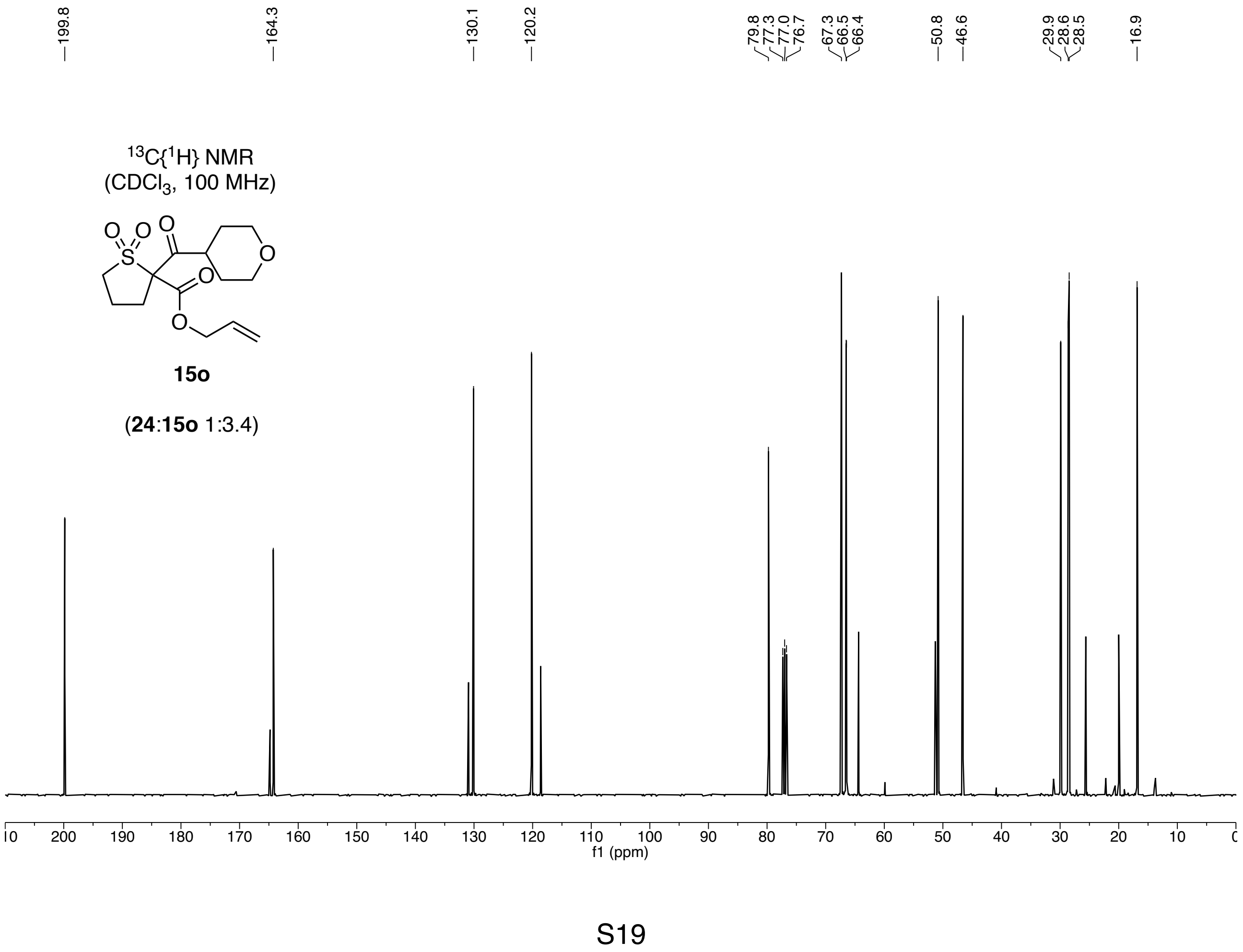

199.8 164.3 130.1 120.2 79.8 77.3 77.0 76.7 67.3 66.5 66.4 50.8 46.6 29.9 28.6 28.5 16.9
¹³C{¹H} NMR
(CDCl3, 100 MHz)
O O O
S
O
O
O
15o
(24:15o 1:3.4)
200 190 180 170 160 150 140 130 120 110 100 90 80 70 60 50 40 30 20 10
f1 (ppm)

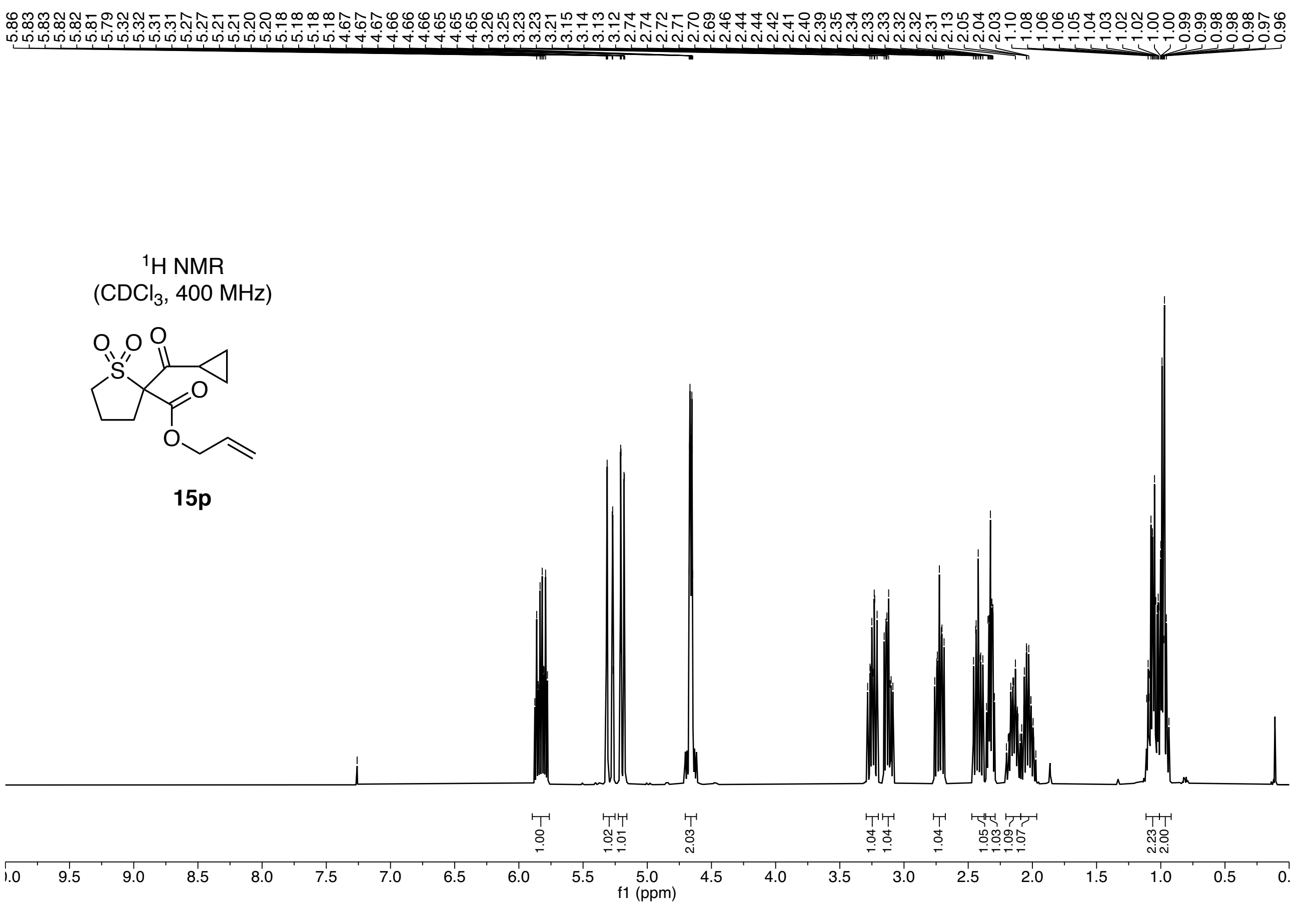
5.86 5.83 5.83 5.82 5.82 5.81 5.79 5.32 5.32 5.31 5.31 5.27 5.27 5.21 5.21 5.20 5.20 5.18 5.18 5.18 5.18 4.67 4.67 4.66 4.66 4.66 4.65 4.65 4.65 3.26 3.25 3.23 3.23 3.21 3.15 3.14 3.13 3.12 2.74 2.74 2.72 2.72 2.71 2.70 2.69 2.46 2.44 2.44 2.42 2.41 2.40 2.39 2.35 2.34 2.33 2.33 2.32 2.32 2.31 2.13 2.05 2.04 2.03 1.10 1.08 1.06 1.06 1.05 1.04 1.03 1.02 1.00 1.00 0.99 0.99 0.98 0.98 0.97 0.96
^{1}H NMR
(CDCl$_3$, 400 MHz)
15p
1.00
1.02
1.01
2.03
1.04
1.04
1.04
1.05
1.03
1.09
1.07
2.23
2.00
f1 (ppm)

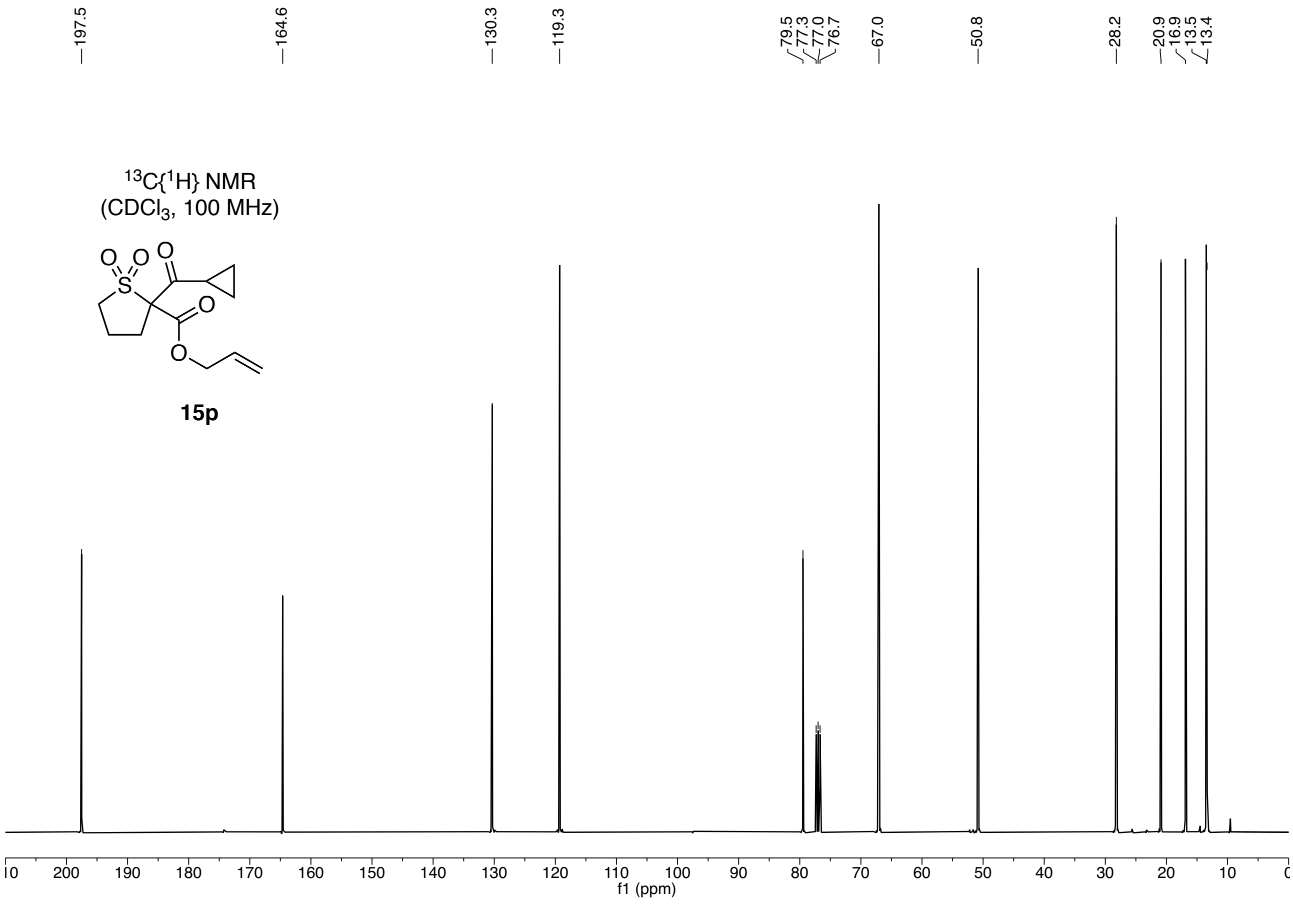
197.5
164.6
130.3
119.3
79.5
77.3
77.0
76.7
67.0
50.8
28.2
20.9
16.9
13.5
13.4
^{13}C{^{1}H} NMR
(CDCl$_3$, 100 MHz)
15p
f1 (ppm)

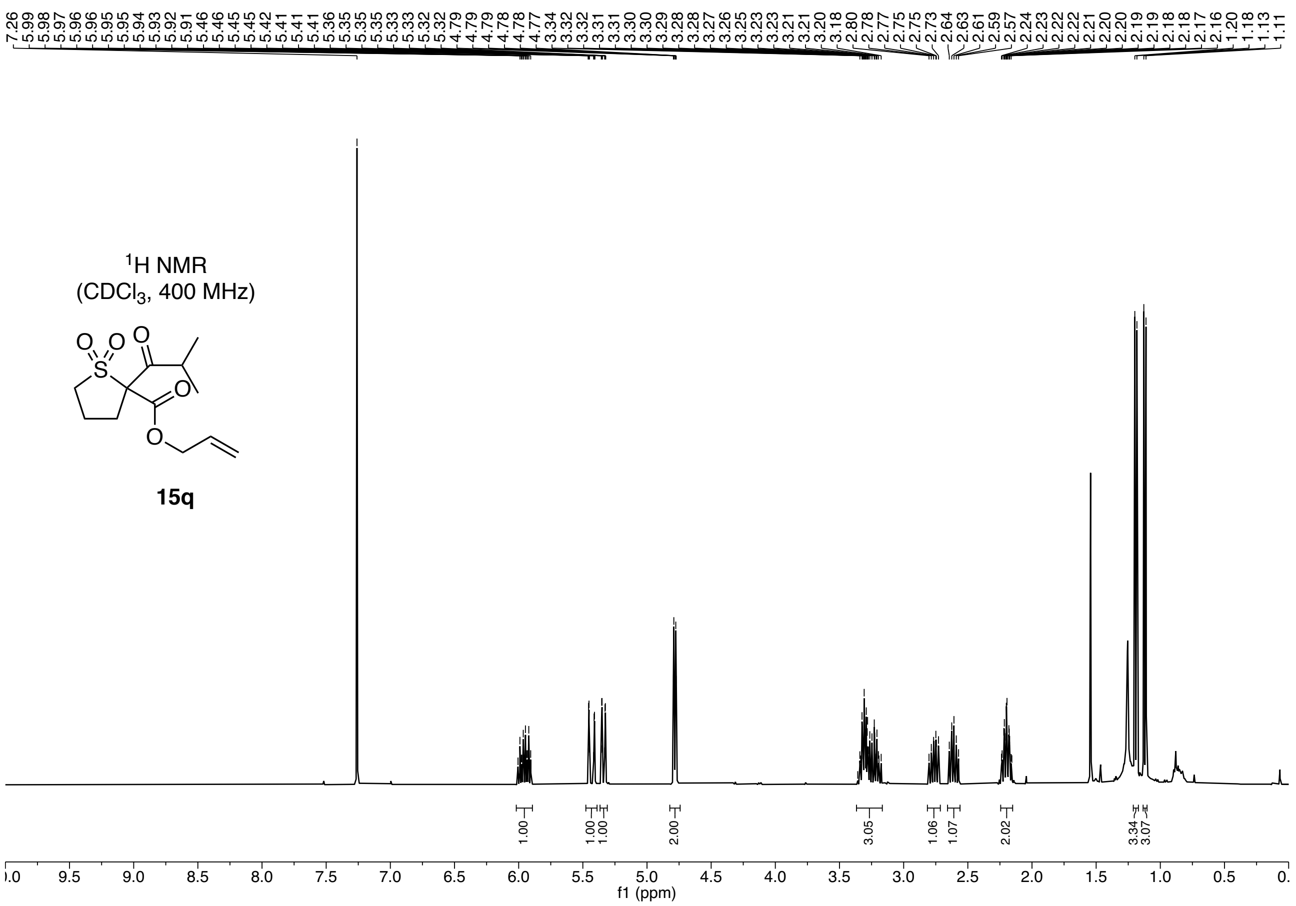

1H NMR
(CDCl3, 400 MHz)
15q
f1 (ppm)

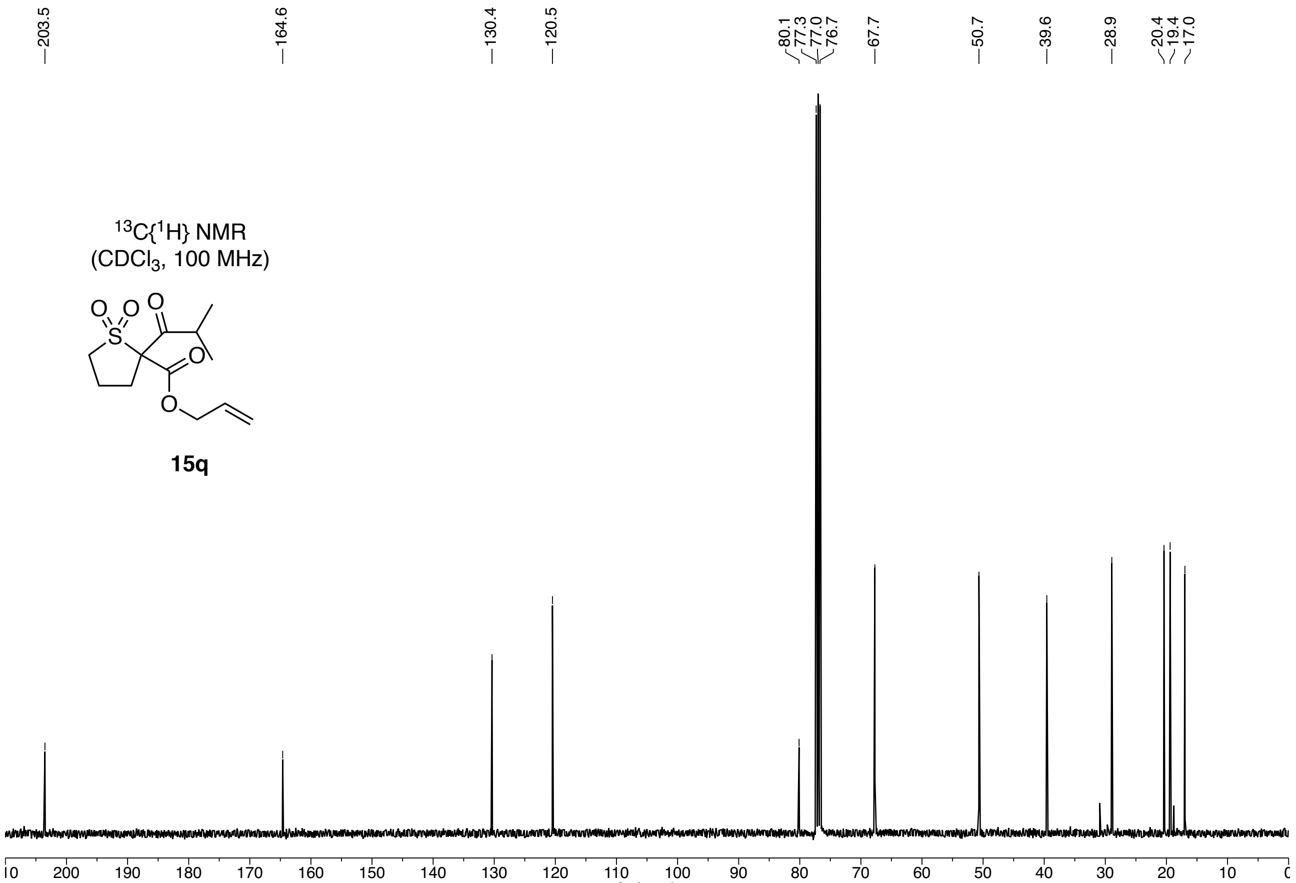

13C{1H} NMR
(CDCl3, 100 MHz)
15q
f1 (ppm)

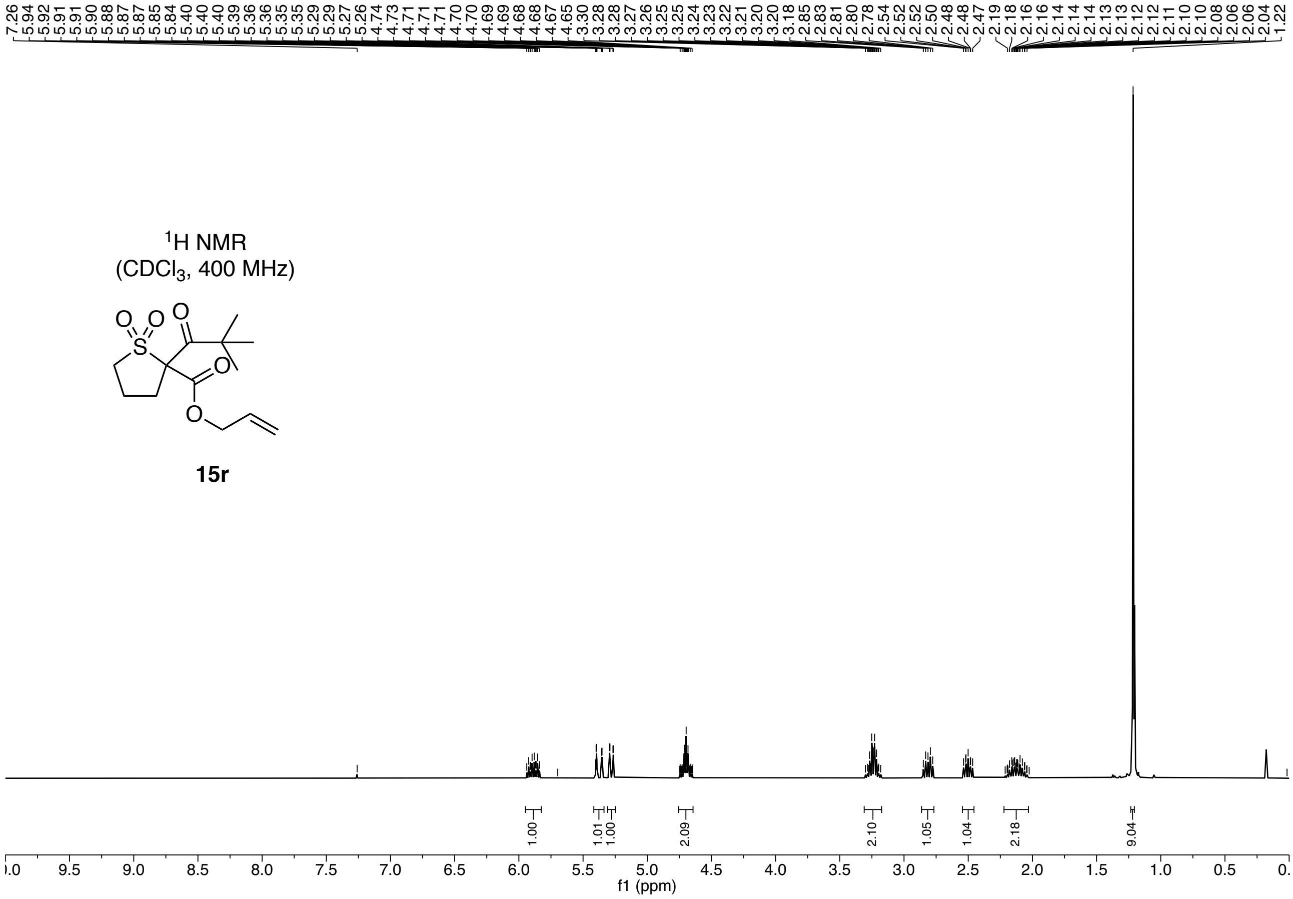
7.26 5.94 5.92 5.91 5.91 5.90 5.88 5.87 5.85 5.84 5.40 5.40 5.39 5.36 5.36 5.35 5.29 5.29 5.27 5.26 4.74 4.73 4.71 4.71 4.70 4.69 4.69 4.68 4.67 4.65 3.30 3.28 3.28 3.27 3.26 3.25 3.24 3.23 3.22 3.21 3.20 3.20 3.18 2.85 2.83 2.81 2.80 2.78 2.54 2.52 2.52 2.50 2.48 2.48 2.47 2.19 2.18 2.16 2.14 2.14 2.13 2.13 2.12 2.12 2.11 2.10 2.08 2.06 2.04 1.22
^{1}H NMR
(CDCl$_3$, 400 MHz)
15r
1.00 1.01 1.00 2.09 2.10 1.05 1.04 2.18 9.04
f1 (ppm)

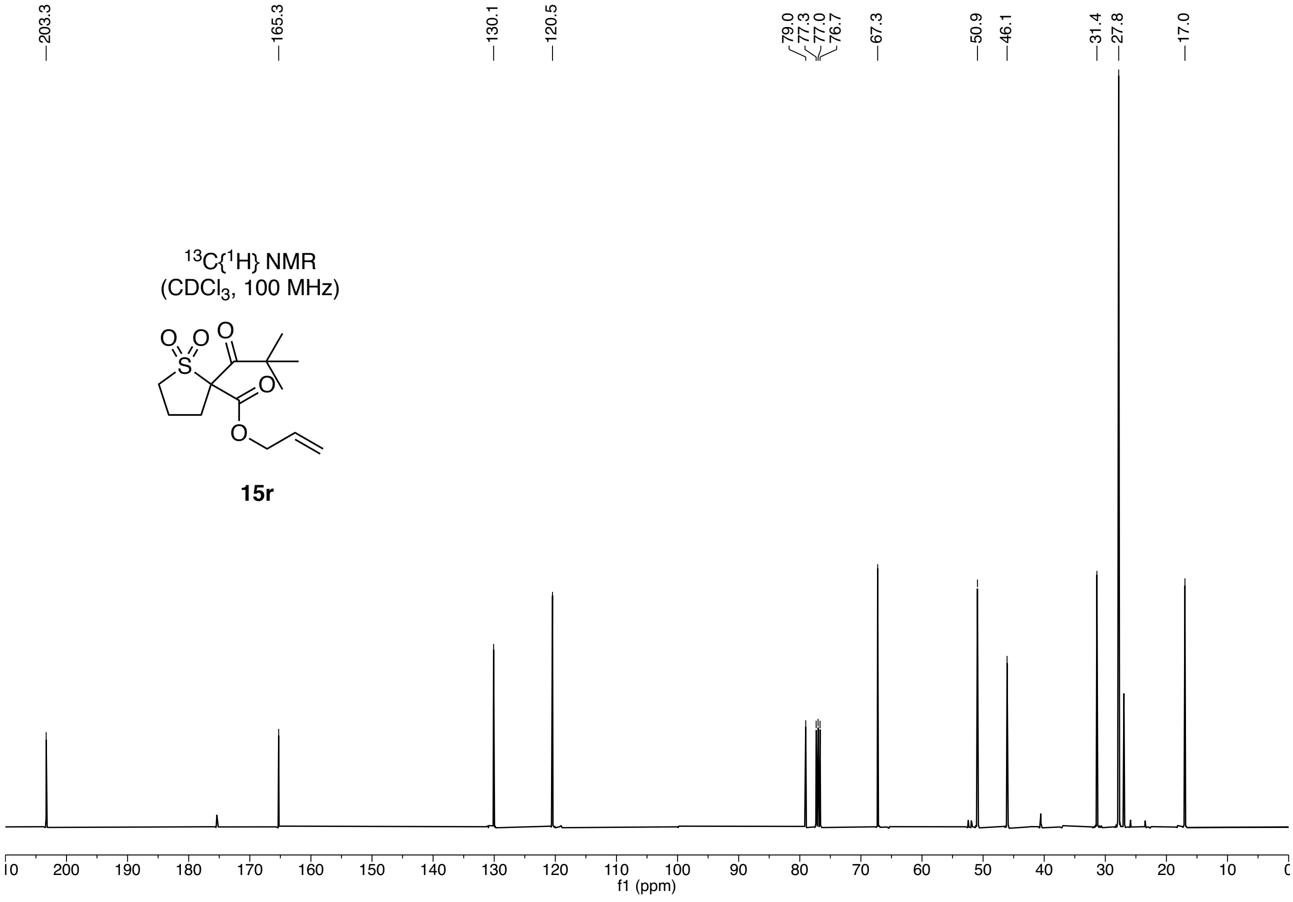
203.3 165.3 130.1 120.5 79.0 77.3 77.0 76.7 67.3 50.9 46.1 31.4 27.8 17.0
^{13}C{^{1}H} NMR
(CDCl$_3$, 100 MHz)
15r
f1 (ppm)

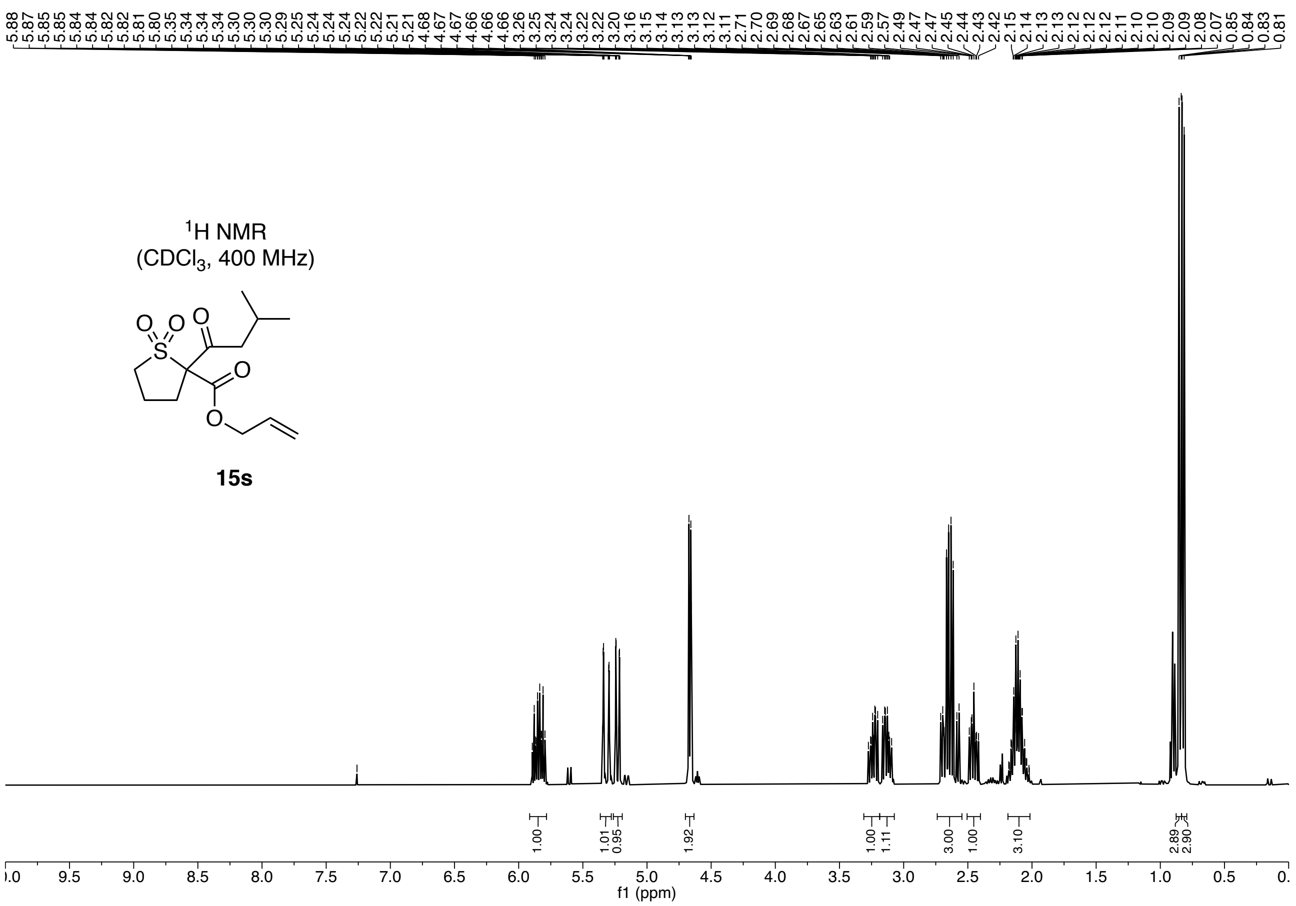
5.88 5.87 5.85 5.85 5.84 5.84 5.82 5.82 5.81 5.80 5.35 5.34 5.34 5.30 5.30 5.29 5.25 5.24 5.24 5.22 5.22 5.21 5.21 4.68 4.67 4.67 4.66 4.66 4.66 3.26 3.25 3.24 3.24 3.22 3.22 3.20 3.16 3.15 3.14 3.13 3.13 3.12 3.11 2.71 2.70 2.69 2.68 2.67 2.65 2.63 2.61 2.59 2.57 2.49 2.47 2.47 2.45 2.44 2.43 2.42 2.15 2.14 2.13 2.13 2.12 2.12 2.12 2.11 2.10 2.10 2.09 2.09 2.08 2.07 0.85 0.84 0.83 0.81
^{1}H NMR
(CDCl$_3$, 400 MHz)
O O O S O O
15s
1.00 1.01 0.95 1.92 1.00 1.11 3.00 1.00 3.10 2.89 2.90
9.5 9.0 8.5 8.0 7.5 7.0 6.5 6.0 5.5 5.0 4.5 4.0 3.5 3.0 2.5 2.0 1.5 1.0 0.5
f1 (ppm)

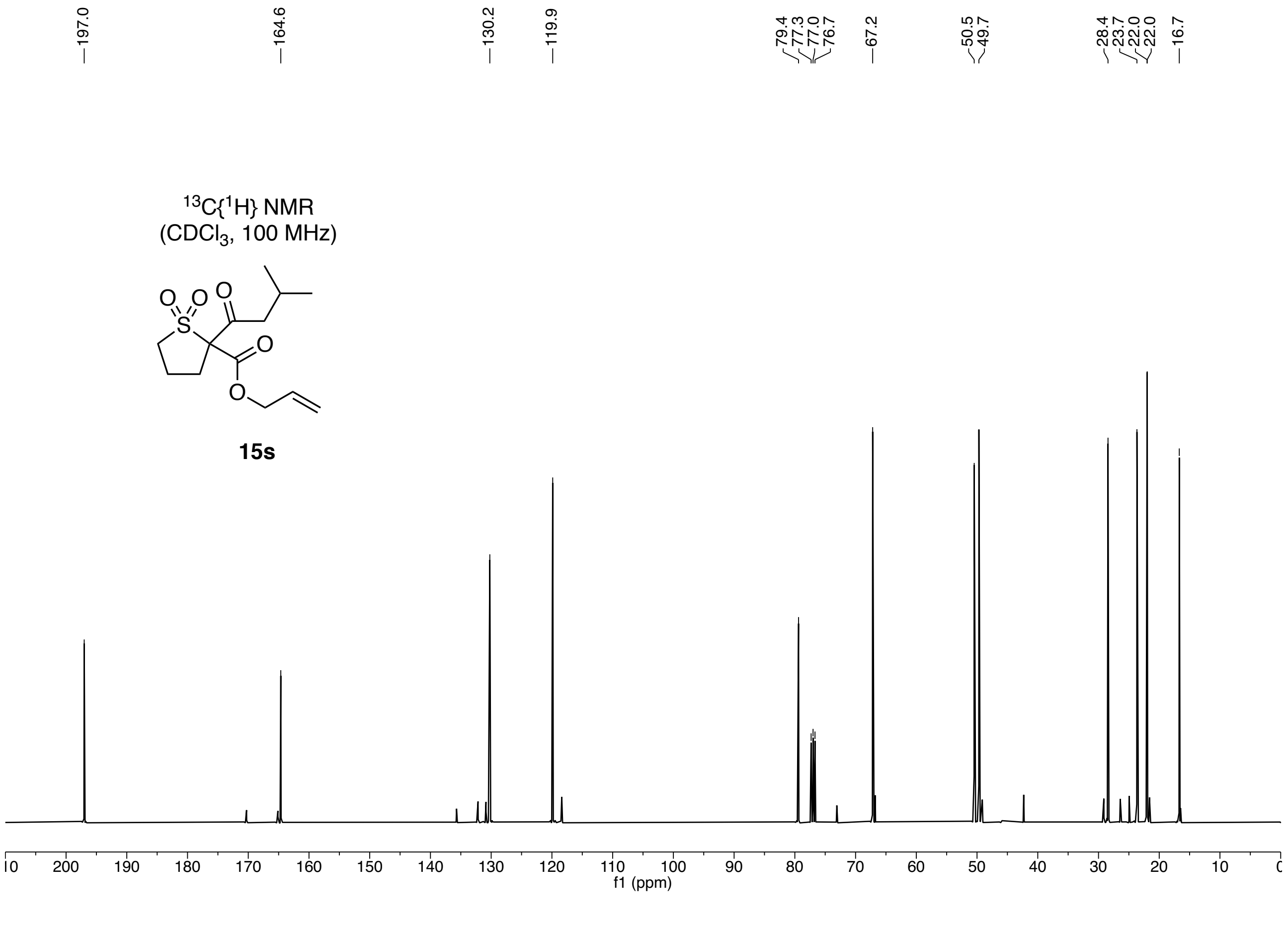
197.0
164.6
130.2
119.9
79.4 77.3 77.0 76.7
67.2
50.5 49.7
28.4 23.7 22.0 22.0
16.7
^{13}C{^{1}H} NMR
(CDCl$_3$, 100 MHz)
O O O S O O
15s
200 190 180 170 160 150 140 130 120 110 100 90 80 70 60 50 40 30 20 10
f1 (ppm)

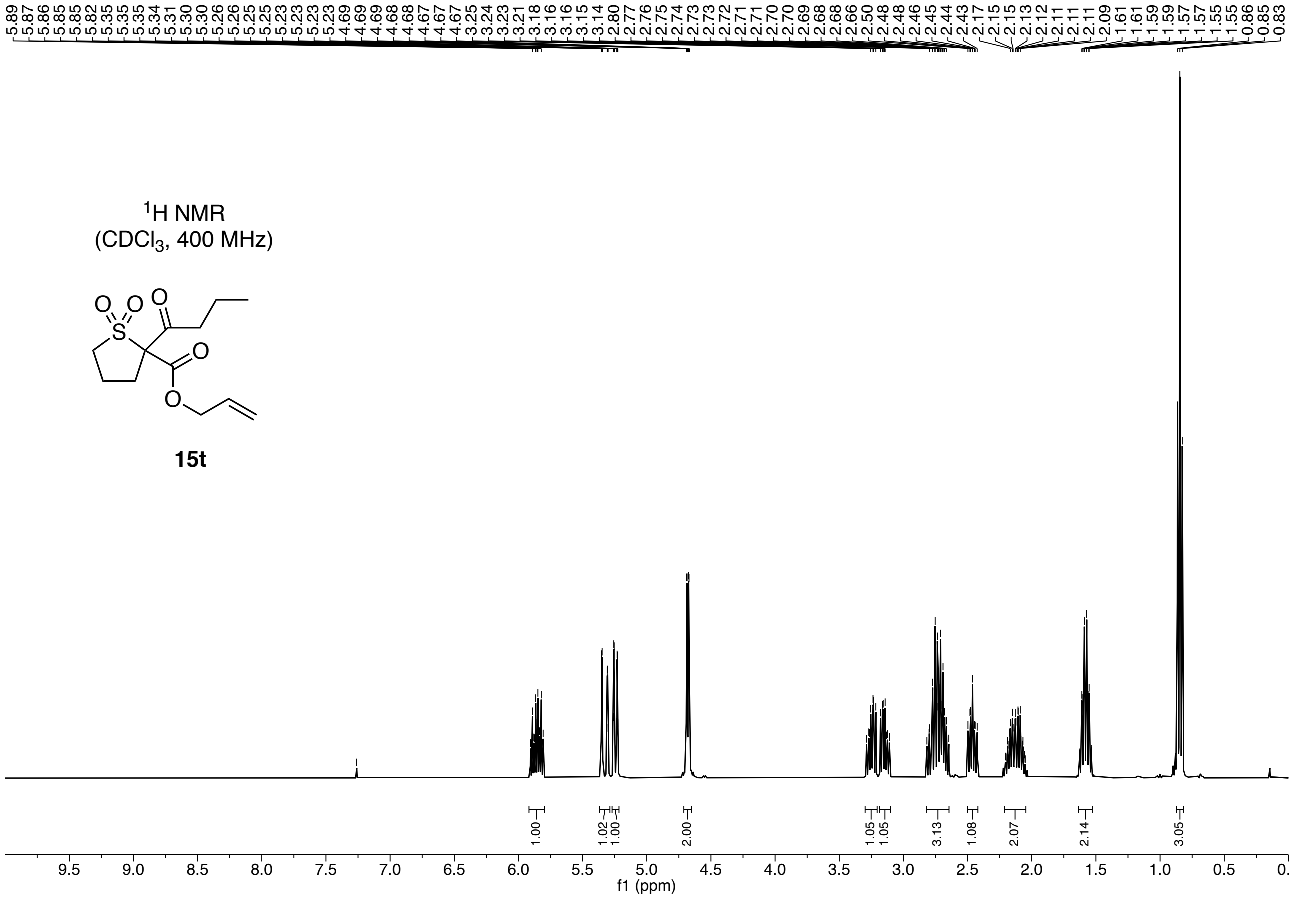
5.89 5.87 5.86 5.85 5.85 5.82 5.35 5.35 5.35 5.34 5.31 5.30 5.30 5.26 5.25 5.25 5.25 5.23 5.23 5.23 5.23 5.23 4.69 4.69 4.68 4.68 4.67 4.67 4.67 3.25 3.24 3.23 3.23 3.21 3.18 3.16 3.16 3.15 3.14 2.80 2.77 2.76 2.75 2.74 2.73 2.73 2.72 2.71 2.71 2.70 2.70 2.69 2.69 2.68 2.66 2.50 2.48 2.48 2.46 2.45 2.44 2.43 2.17 2.15 2.15 2.13 2.12 2.11 2.11 2.11 2.09 1.61 1.59 1.59 1.57 1.57 1.55 0.86 0.85 0.83
^{1}H NMR
($CDCl_3$, 400 MHz)
15t
1.00 1.02 1.00 2.00 1.05 1.05 3.13 1.08 2.07 2.14 3.05
9.5 9.0 8.5 8.0 7.5 7.0 6.5 6.0 5.5 5.0 4.5 4.0 3.5 3.0 2.5 2.0 1.5 1.0 0.5
f1 (ppm)

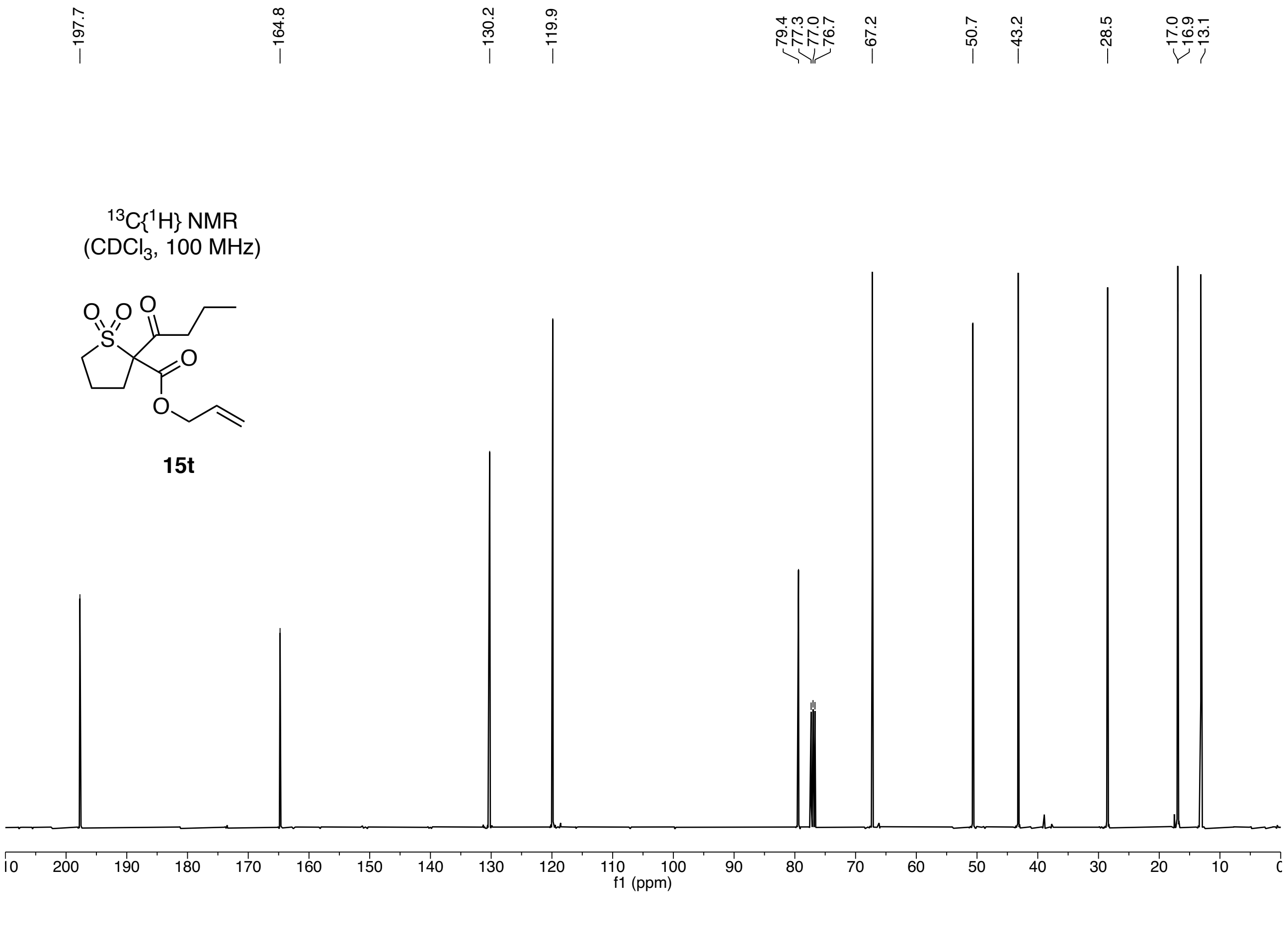
197.7
164.8
130.2
119.9
79.4 77.3 77.0 76.7
67.2
50.7
43.2
28.5
17.0 16.9 13.1
^{13}C{^{1}H} NMR
($CDCl_3$, 100 MHz)
15t
200 190 180 170 160 150 140 130 120 110 100 90 80 70 60 50 40 30 20 10
f1 (ppm)

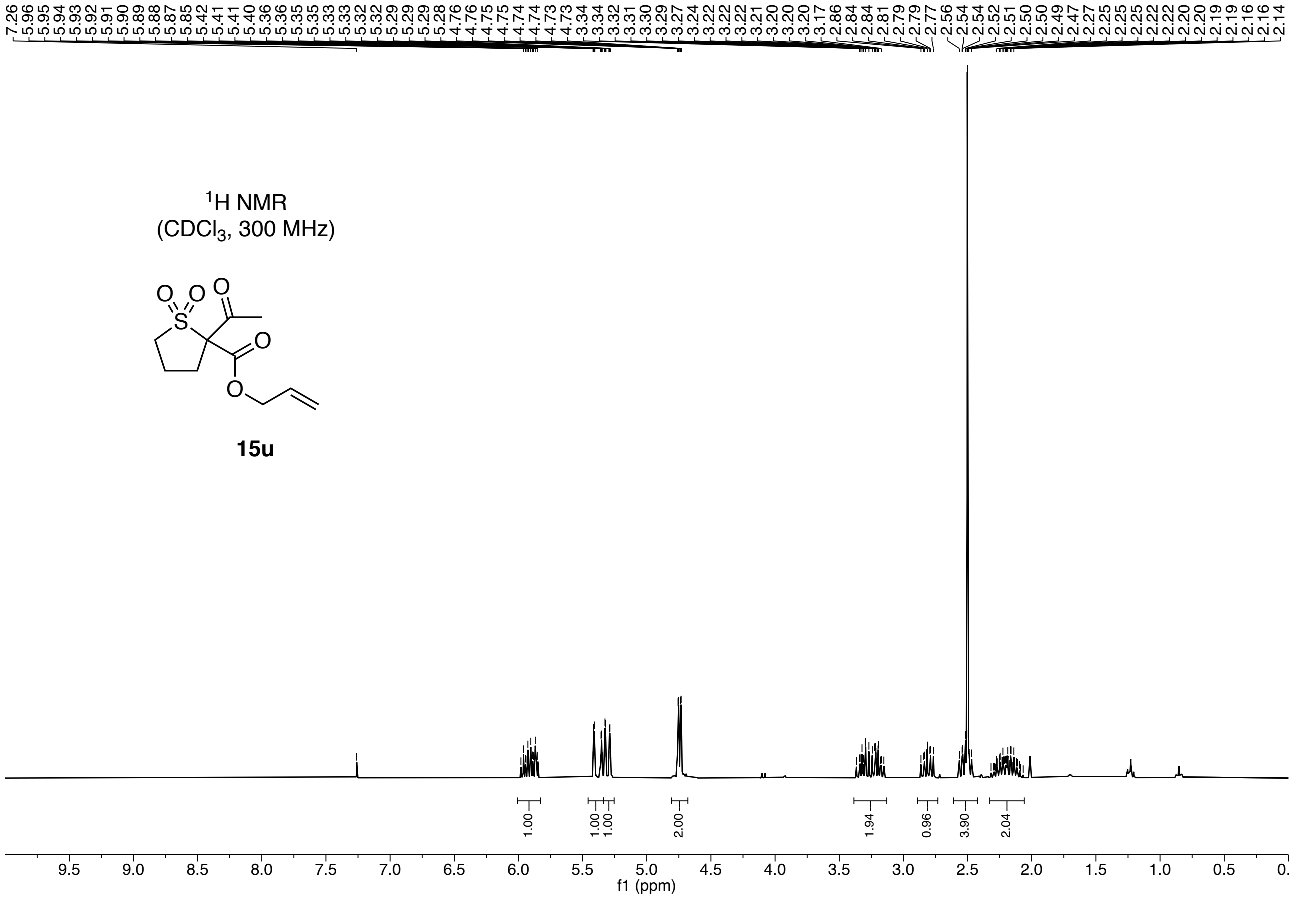

1H NMR
(CDCl3, 300 MHz)
15u
f1 (ppm)

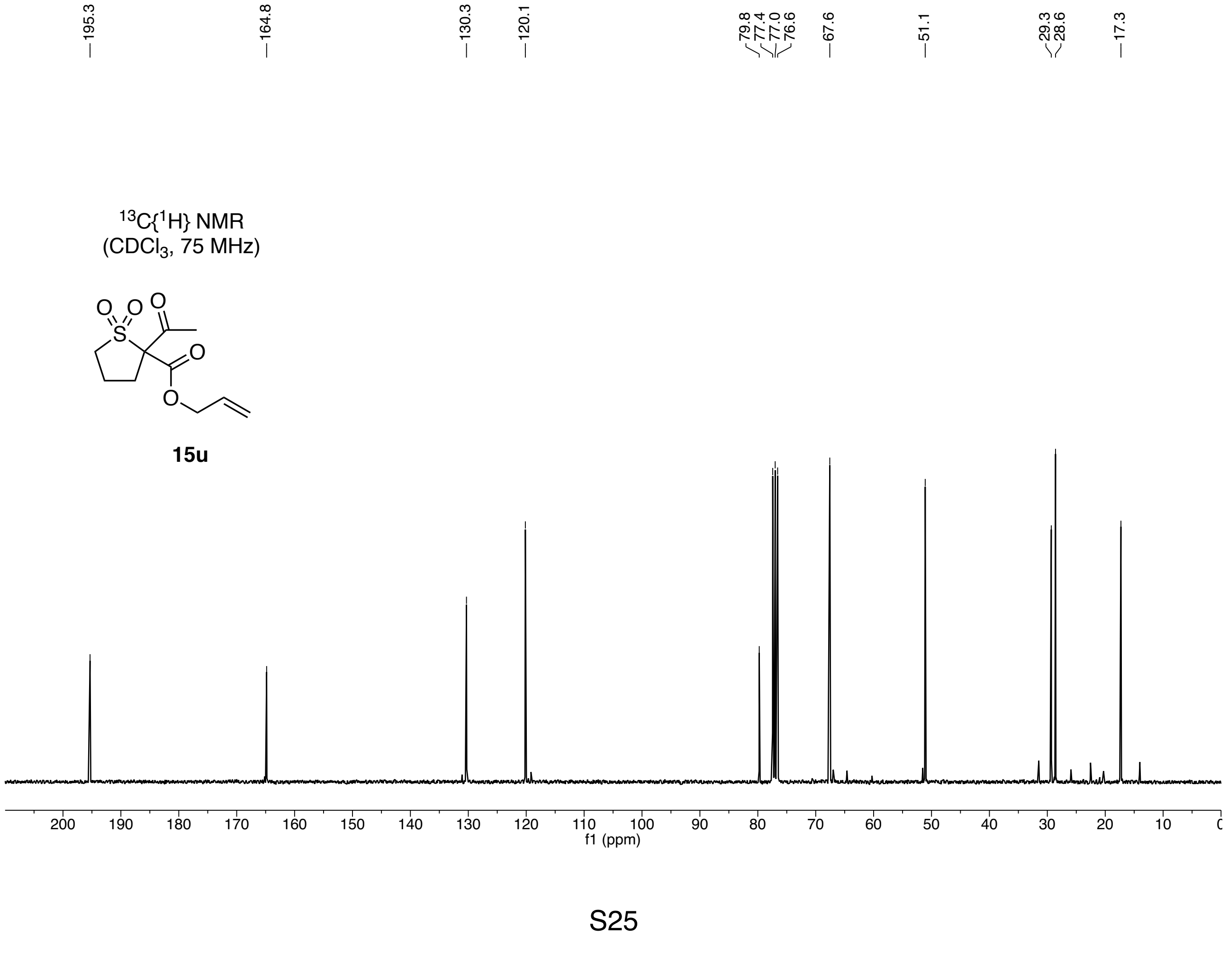

195.3
164.8
130.3
120.1
79.8
77.4
77.0
76.6
67.6
51.1
29.3
28.6
17.3
13C{1H} NMR
(CDCl3, 75 MHz)
15u
f1 (ppm)

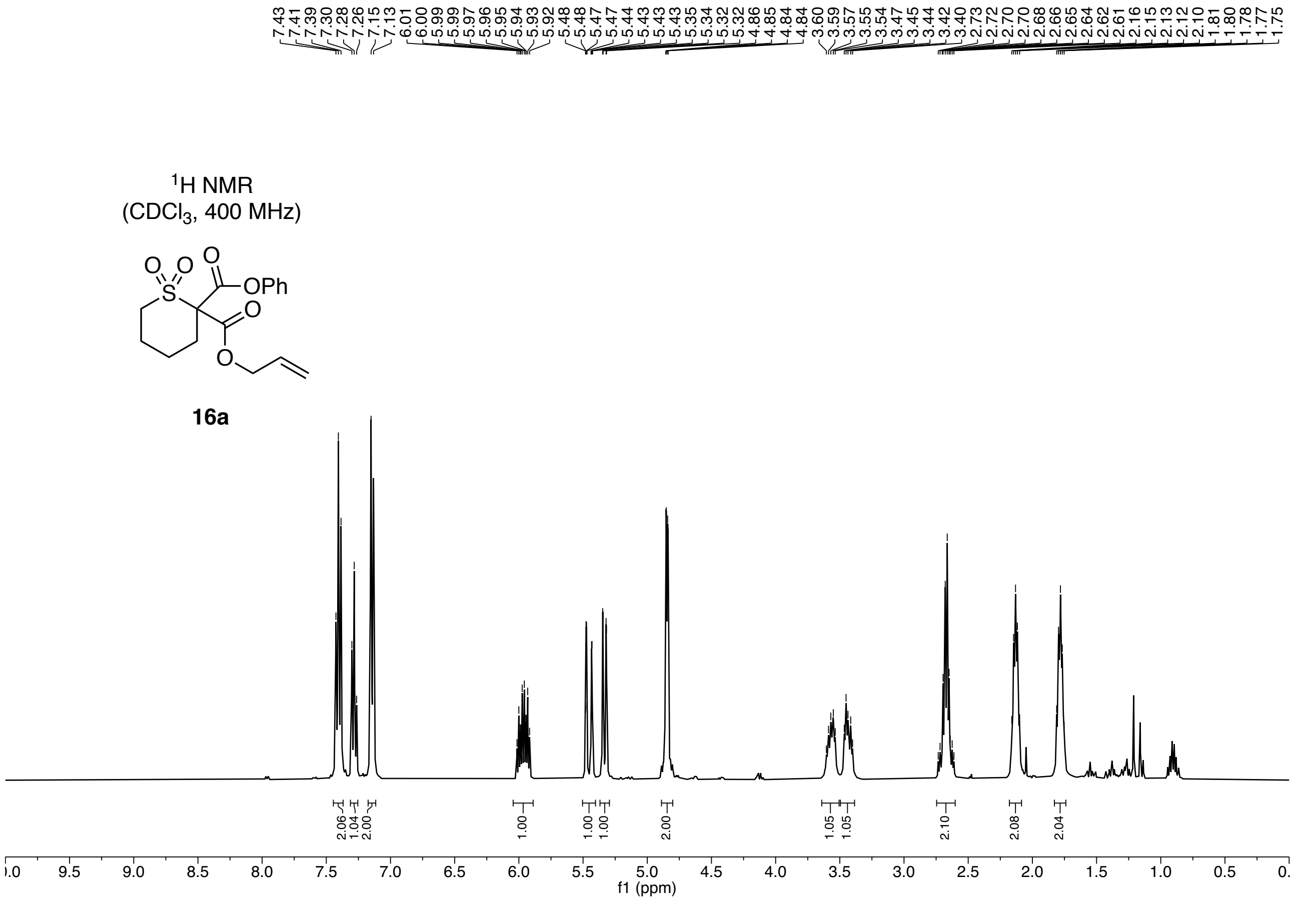
7.43 7.41 7.39 7.30 7.28 7.26 7.15 7.13 6.01 6.00 5.99 5.97 5.96 5.95 5.94 5.93 5.92 5.48 5.48 5.47 5.44 5.43 5.43 5.35 5.34 5.32 5.32 4.86 4.85 4.84 4.84 3.60 3.59 3.57 3.55 3.54 3.47 3.45 3.44 3.42 3.40 2.73 2.72 2.70 2.70 2.68 2.66 2.65 2.64 2.62 2.61 2.16 2.15 2.13 2.12 2.10 1.81 1.80 1.78 1.77 1.75
¹H NMR
(CDCl3, 400 MHz)
OPh
16a
2.06 1.04 2.00 1.00 1.00 1.00 2.00 1.05 1.05 2.10 2.08 2.04
f1 (ppm)

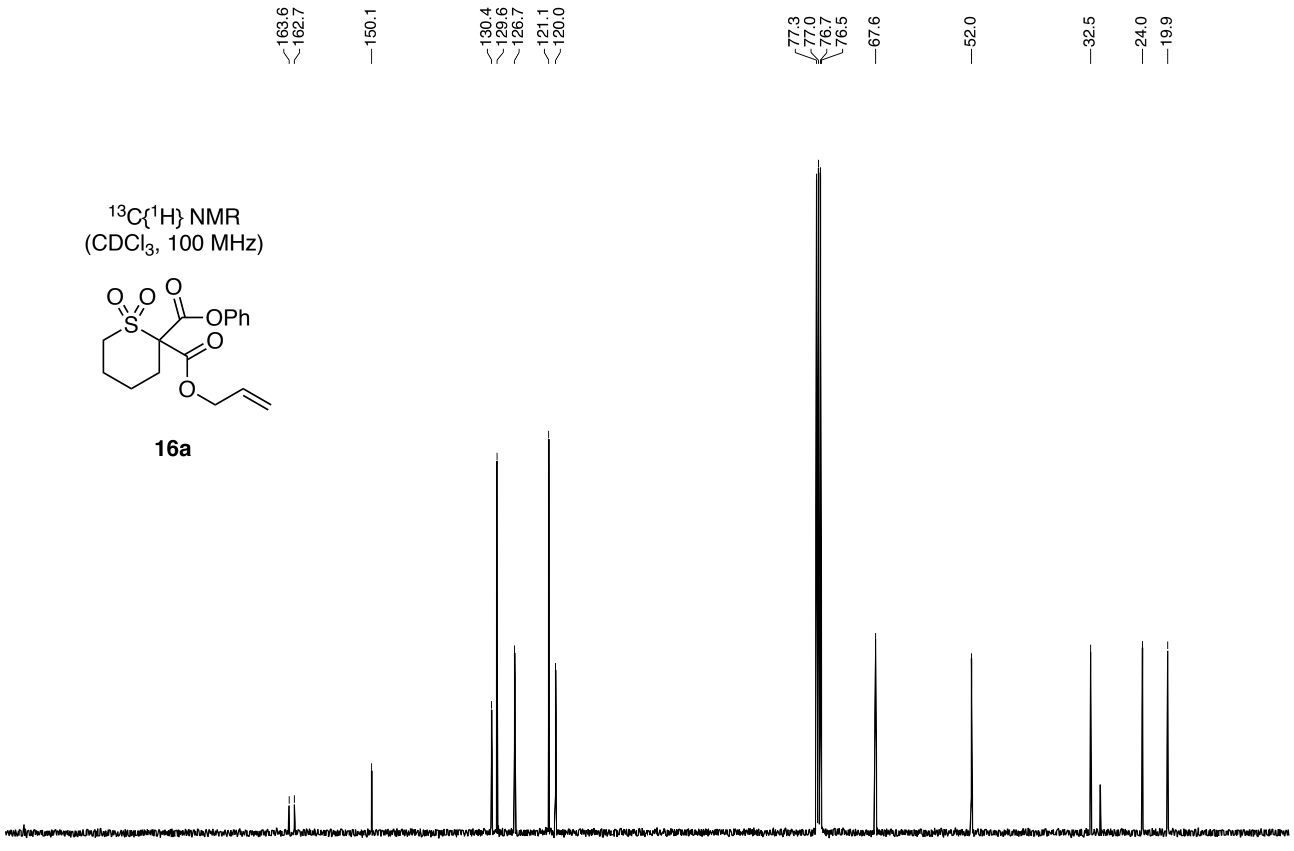
163.6 162.7 150.1 130.4 129.6 126.7 121.1 120.0 77.3 77.0 76.7 76.5 67.6 52.0 32.5 24.0 19.9
¹³C{¹H} NMR
(CDCl3, 100 MHz)
OPh
16a
f1 (ppm)

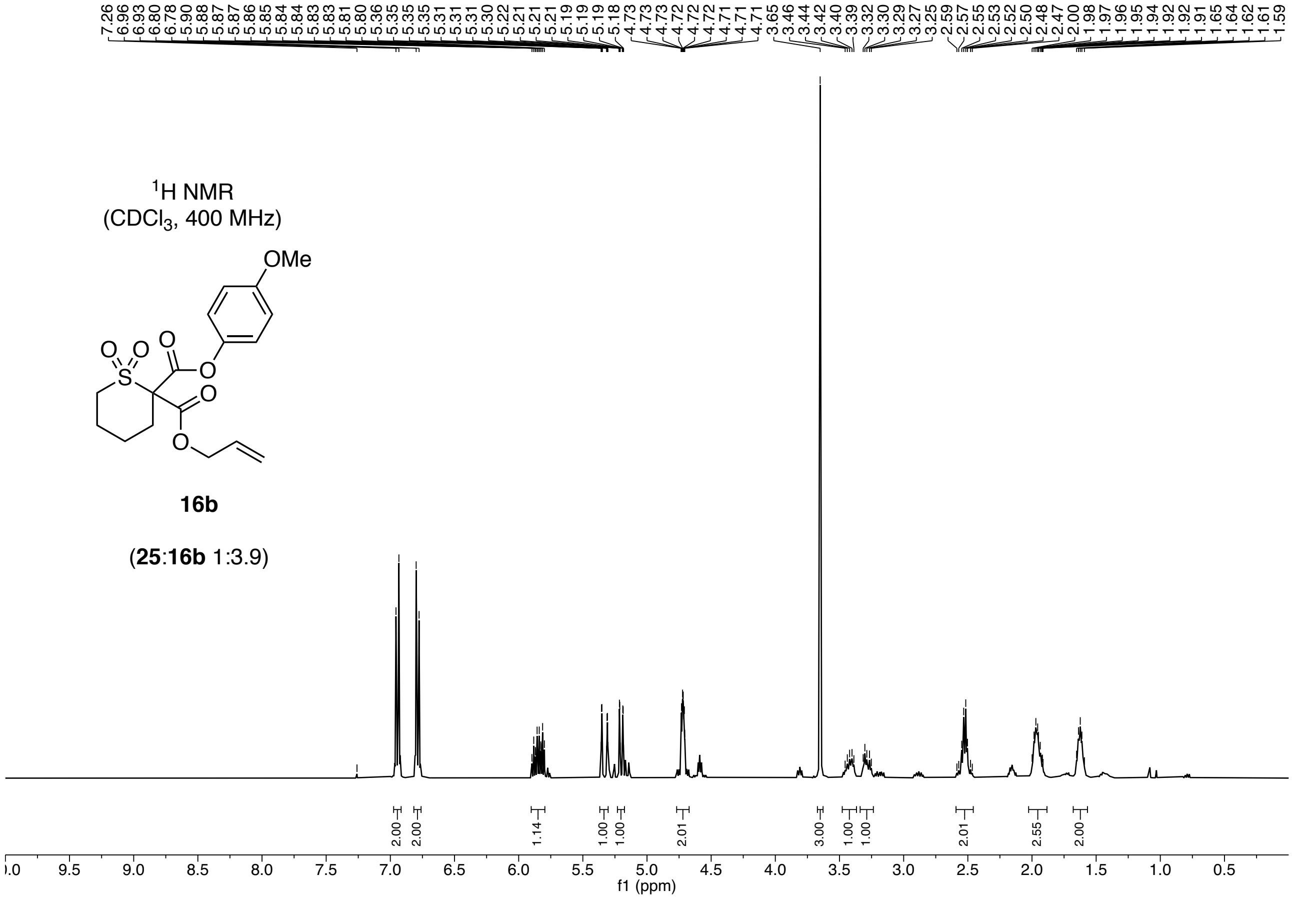
7.26 6.96 6.93 6.80 6.78 5.90 5.88 5.87 5.86 5.85 5.84 5.83 5.83 5.81 5.80 5.36 5.35 5.35 5.31 5.31 5.31 5.30 5.22 5.21 5.21 5.19 5.19 5.18 4.73 4.73 4.72 4.72 4.72 4.71 4.71 3.65 3.46 3.44 3.42 3.40 3.39 3.32 3.30 3.29 3.27 3.25 2.59 2.57 2.55 2.53 2.52 2.50 2.48 2.47 2.00 1.98 1.97 1.96 1.95 1.94 1.92 1.92 1.91 1.65 1.64 1.62 1.61 1.59
^{1}H NMR
(CDCl$_3$, 400 MHz)
OMe
O
S
16b
(**25**:**16b** 1:3.9)
2.00 2.00 1.14 1.00 1.00 2.01 3.00 1.00 1.00 2.01 2.55 2.00
f1 (ppm)

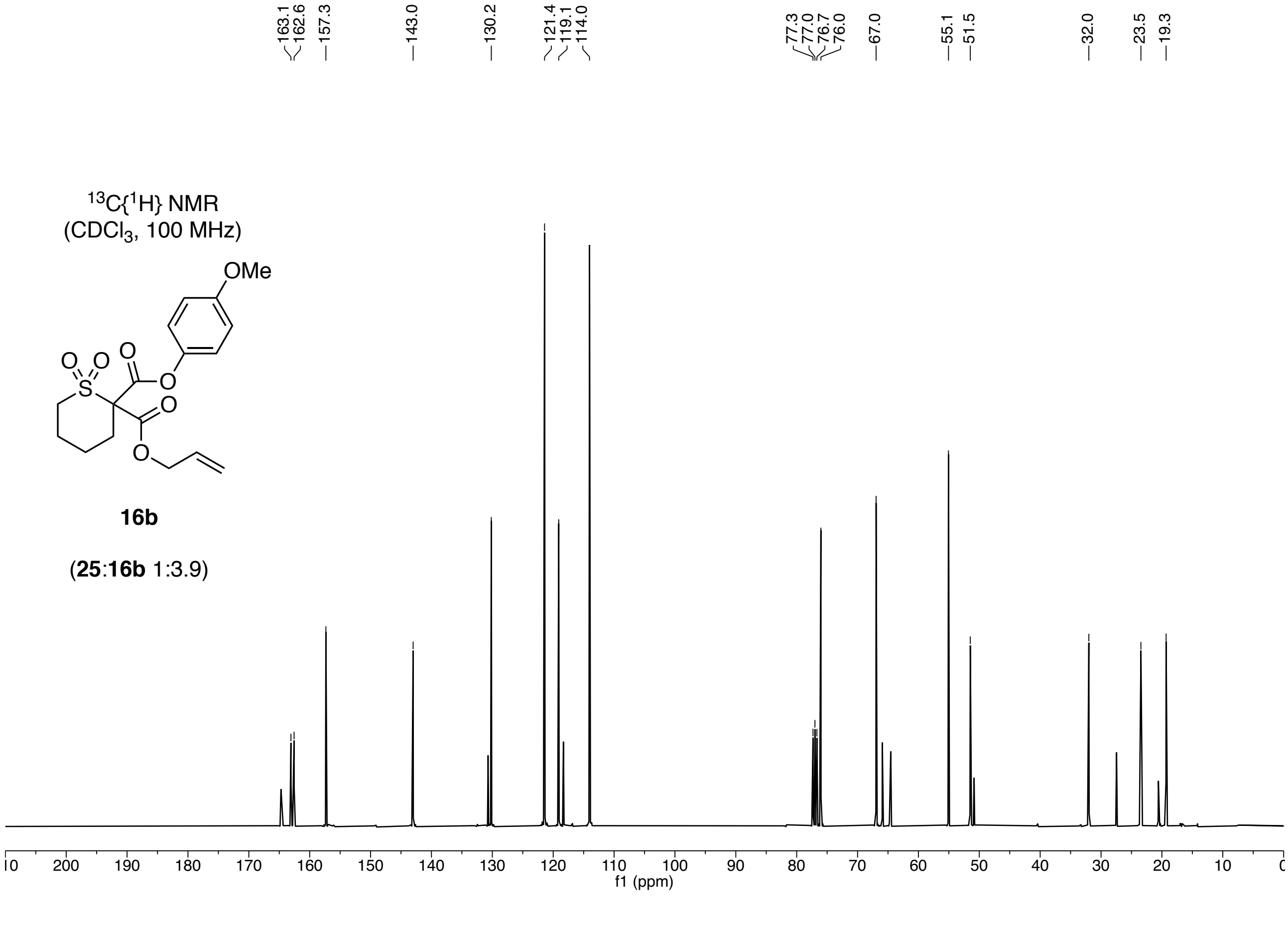
163.1 162.6 157.3 143.0 130.2 121.4 119.1 114.0 77.3 77.0 76.7 76.0 67.0 55.1 51.5 32.0 23.5 19.3
^{13}C{^{1}H} NMR
(CDCl$_3$, 100 MHz)
OMe
O
S
16b
(**25**:**16b** 1:3.9)
f1 (ppm)

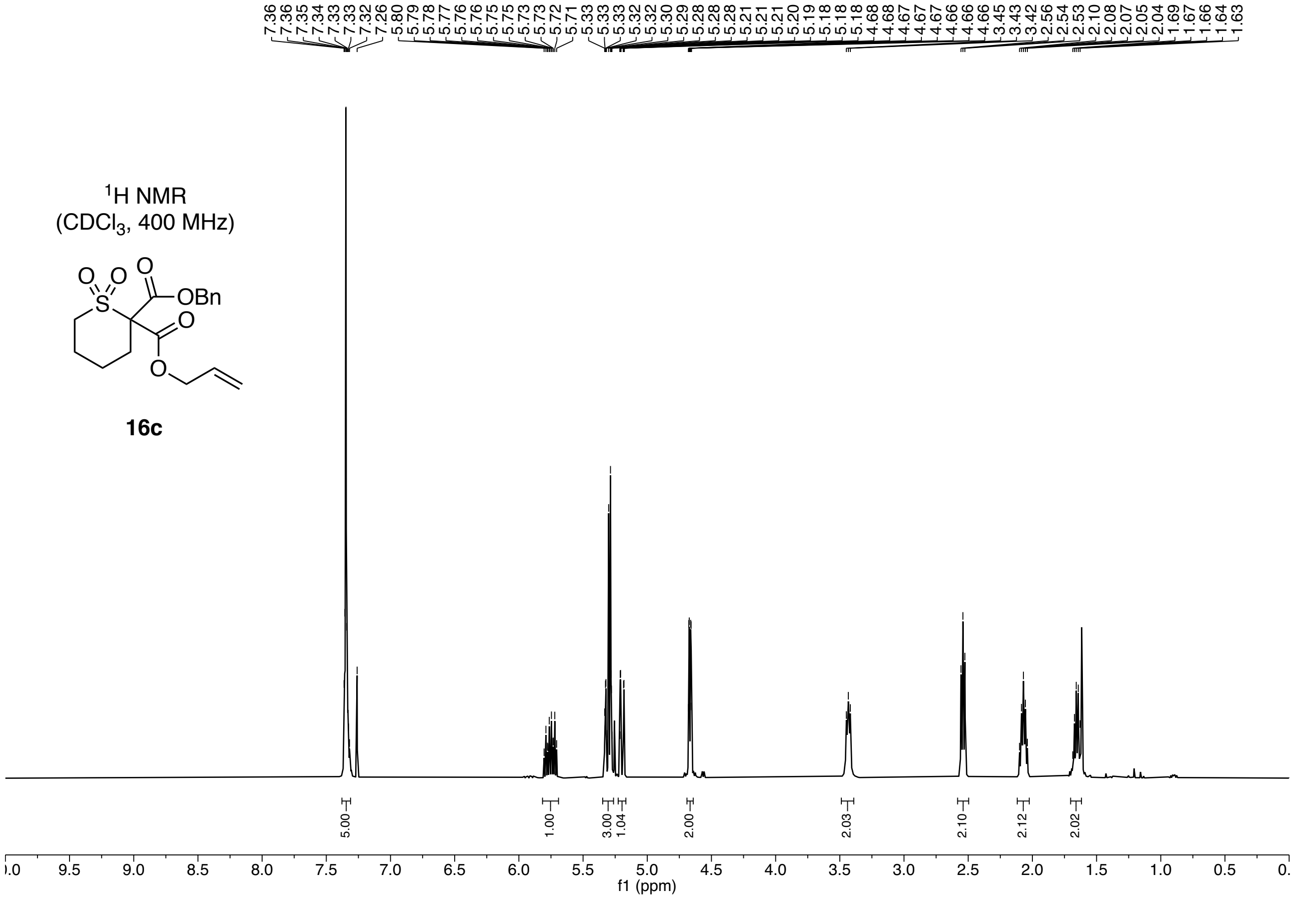
^{1}H NMR
(CDCl$_3$, 400 MHz)
OBn
16c
f1 (ppm)

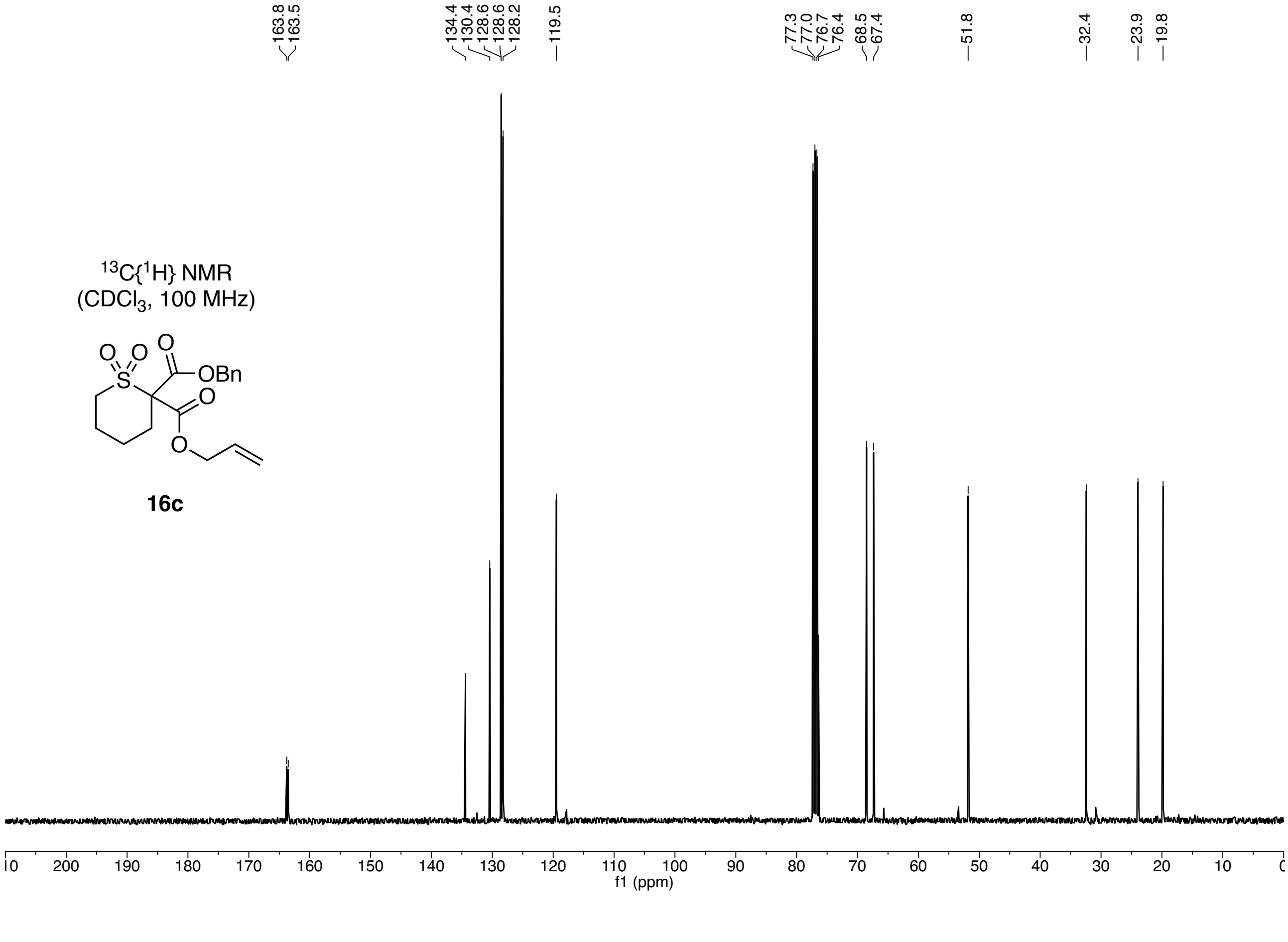
^{13}C{^{1}H} NMR
(CDCl$_3$, 100 MHz)
OBn
16c
f1 (ppm)

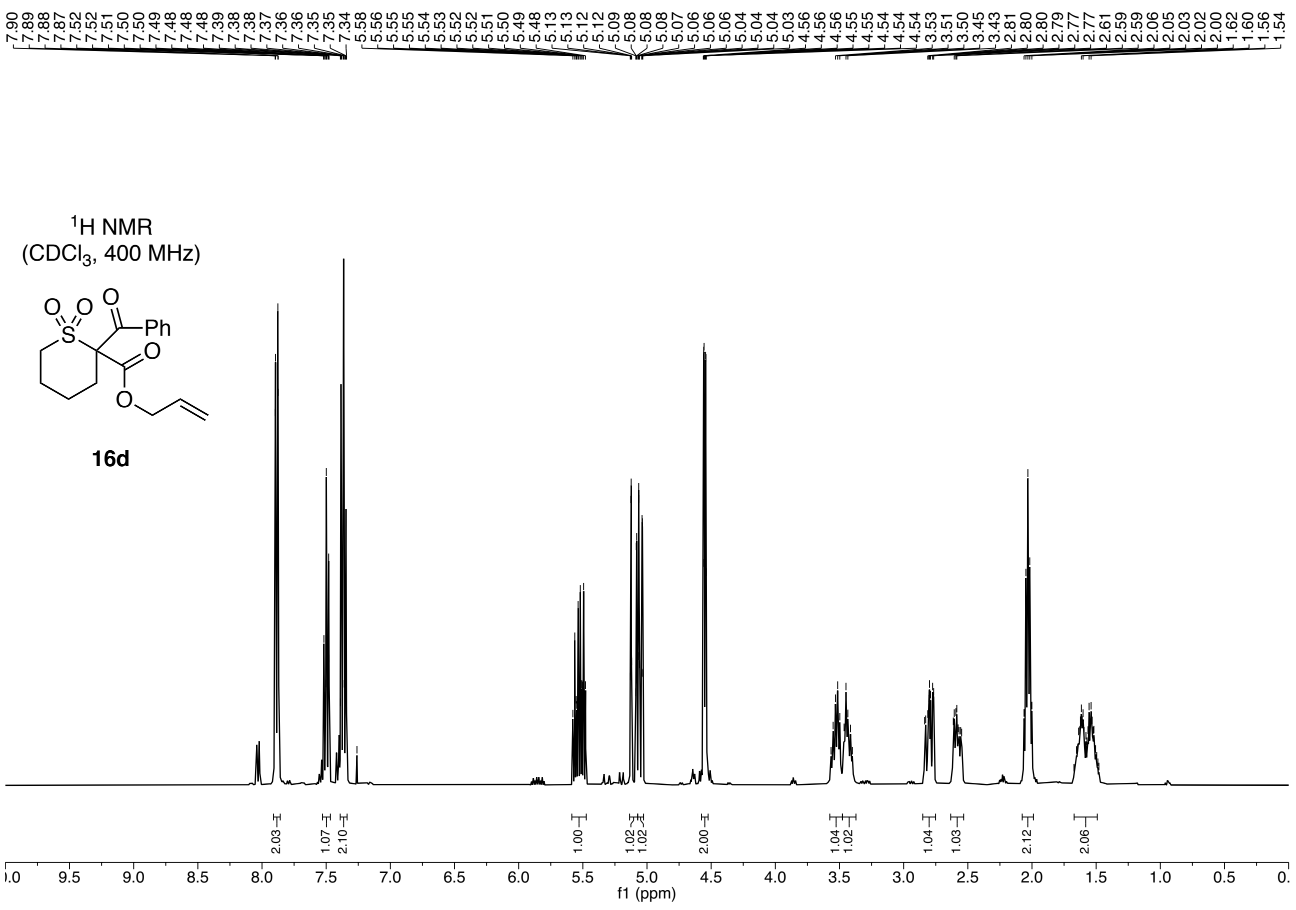

¹H NMR
(CDCl₃, 400 MHz)
16d
f1 (ppm)

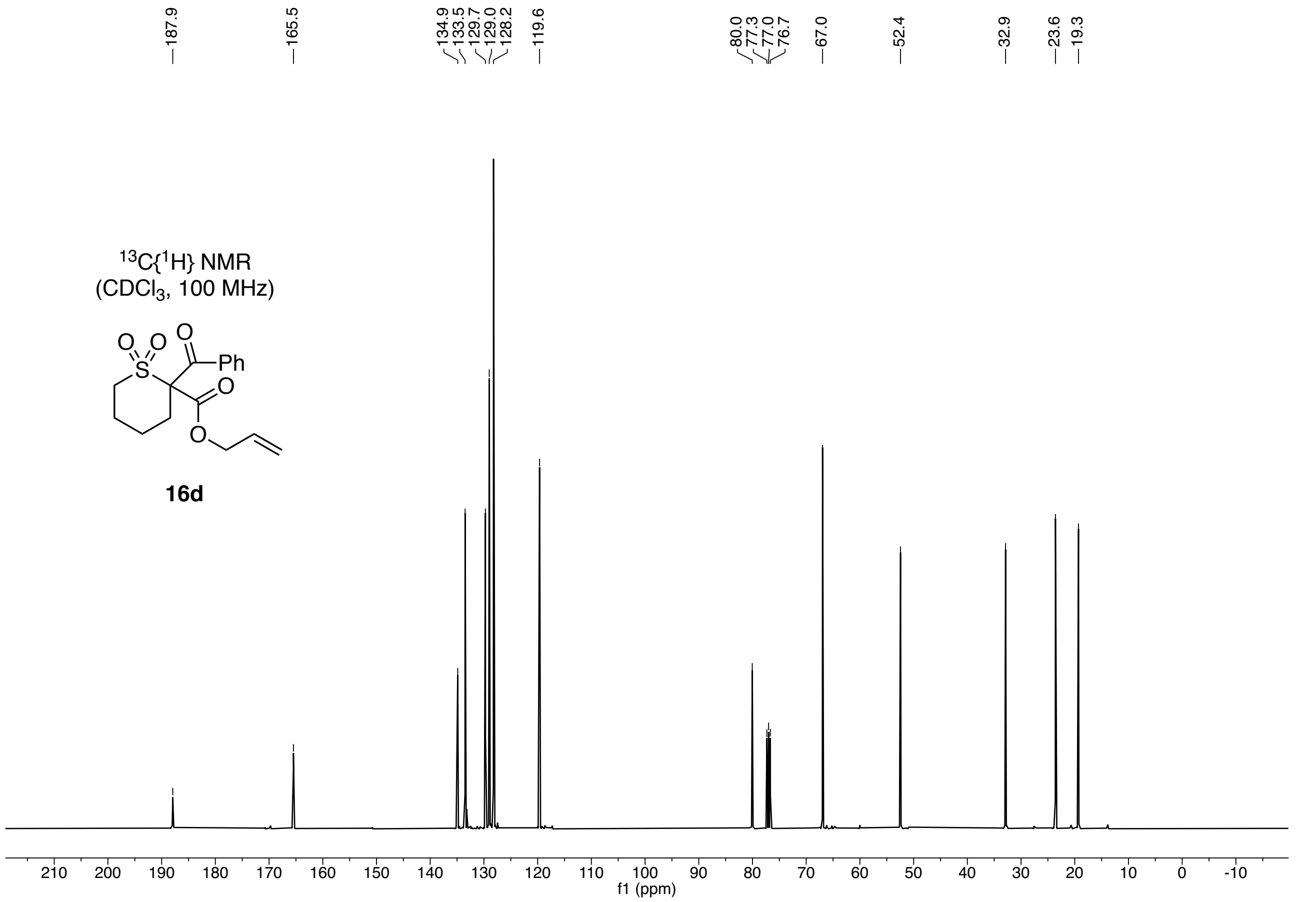

187.9
165.5
134.9
133.5
129.7
129.0
128.2
119.6
80.0
77.3
77.0
76.7
67.0
52.4
32.9
23.6
19.3
¹³C{¹H} NMR
(CDCl₃, 100 MHz)
16d
f1 (ppm)

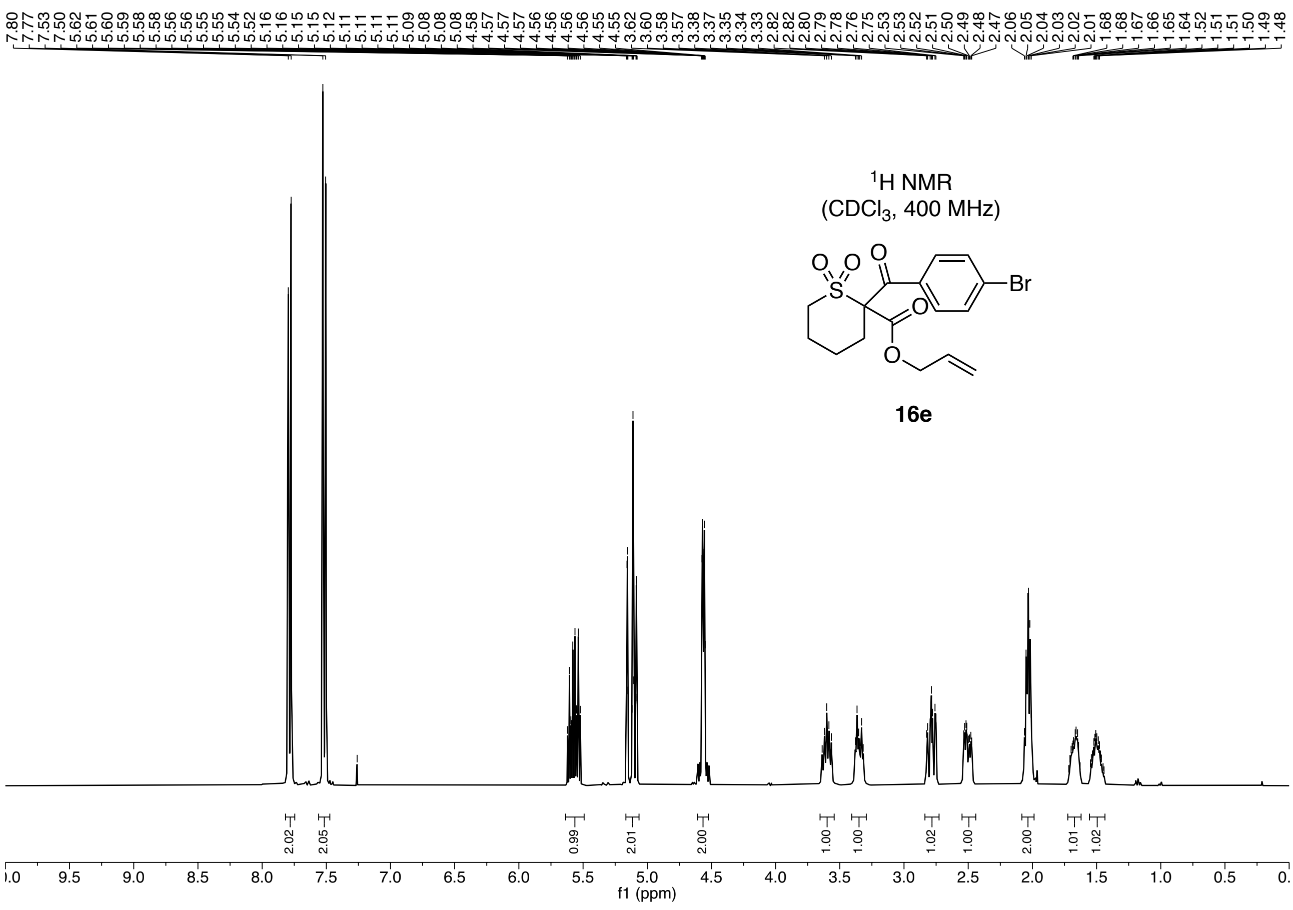

^{1}H NMR
(CDCl$_3$, 400 MHz)
16e
f1 (ppm)

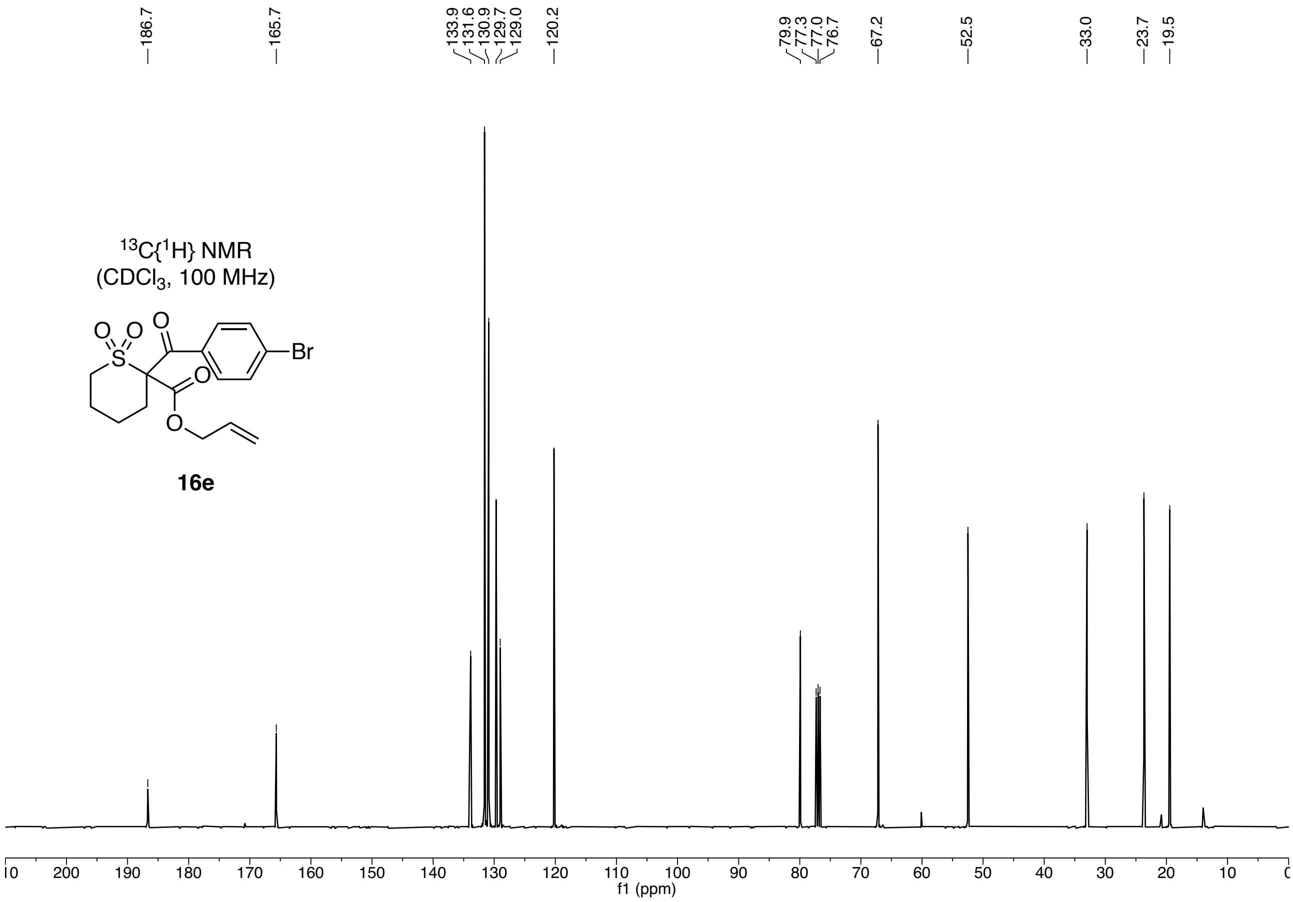

186.7
165.7
133.9
131.6
130.9
129.7
129.0
120.2
79.9
77.3
77.0
76.7
67.2
52.5
33.0
23.7
19.5
^{13}C{^{1}H} NMR
(CDCl$_3$, 100 MHz)
16e
f1 (ppm)

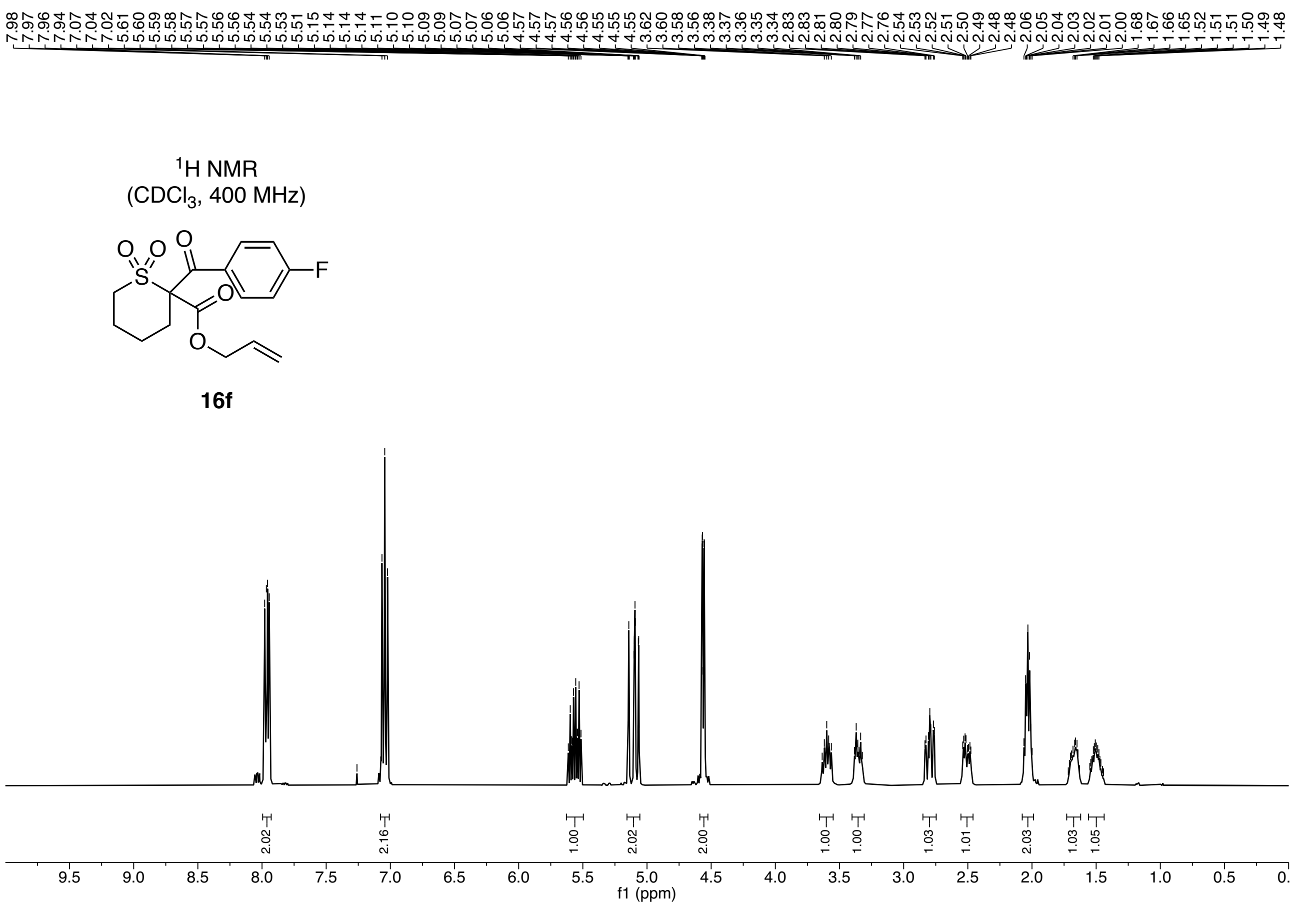
^{1}H NMR
(CDCl$_3$, 400 MHz)
16f
f1 (ppm)

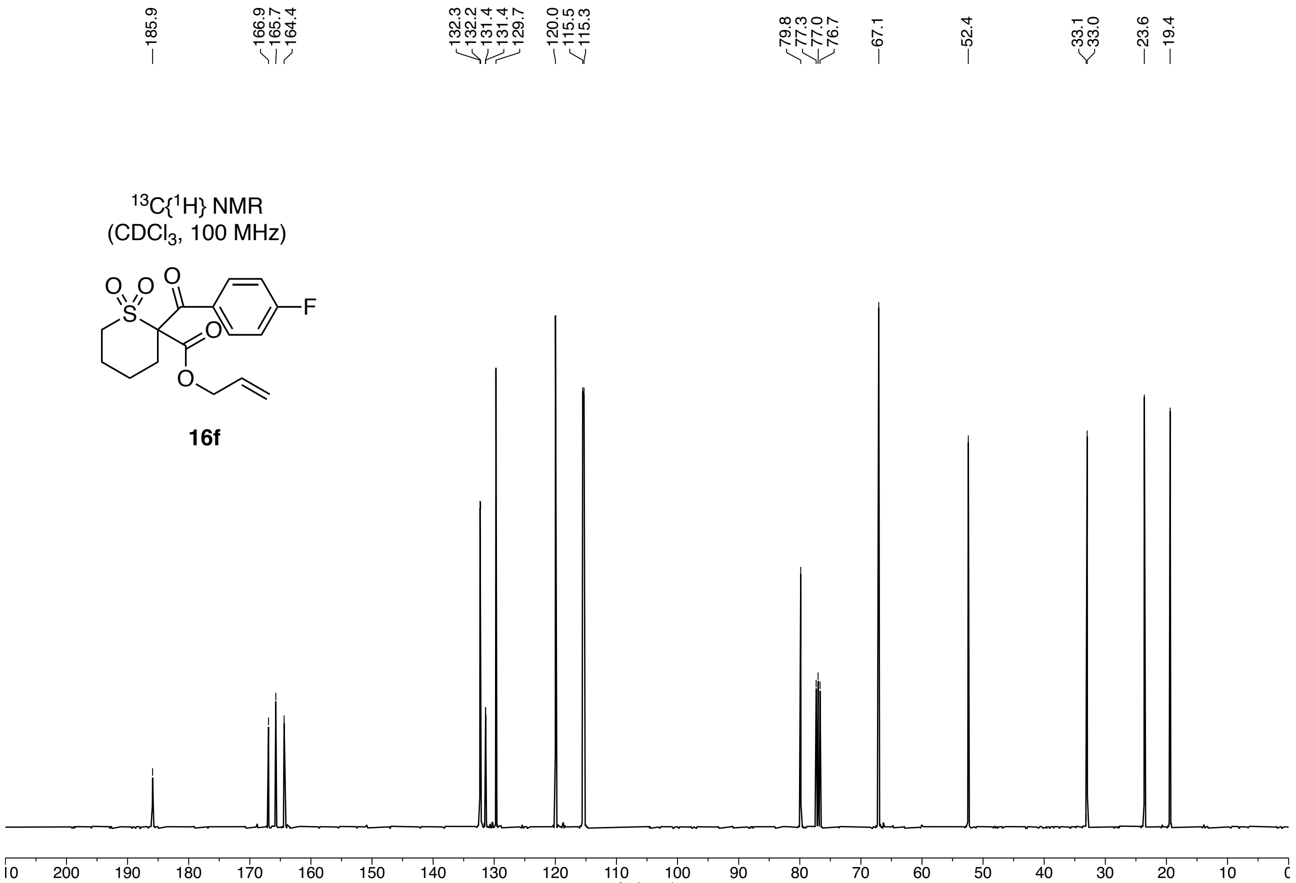
^{13}C{^{1}H} NMR
(CDCl$_3$, 100 MHz)
16f
f1 (ppm)

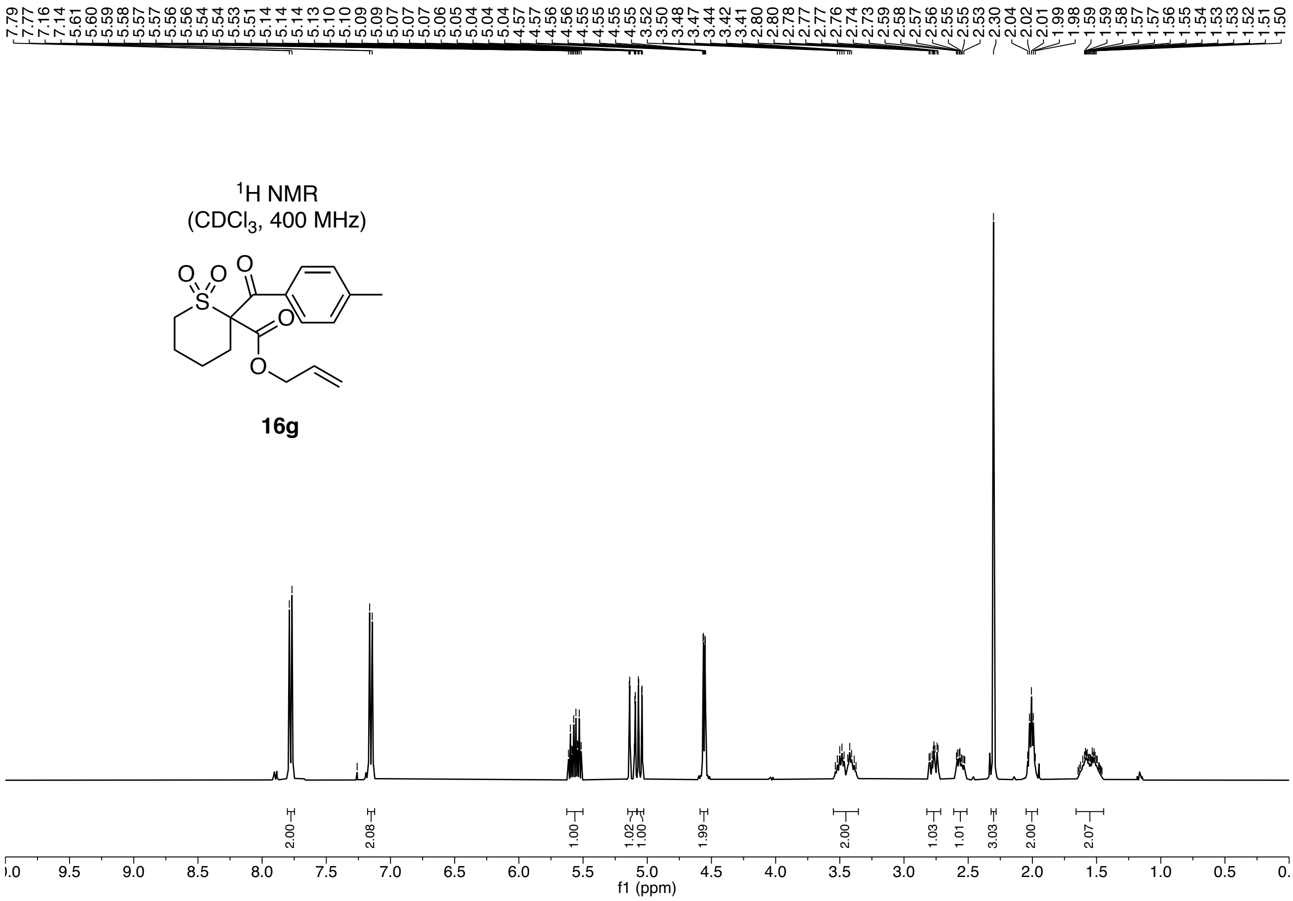
7.79 7.77 7.16 7.14 5.61 5.60 5.59 5.58 5.57 5.57 5.56 5.54 5.54 5.53 5.51 5.14 5.14 5.13 5.10 5.10 5.09 5.07 5.07 5.06 5.05 5.04 5.04 5.04 4.57 4.57 4.56 4.56 4.55 4.55 4.55 3.52 3.50 3.48 3.47 3.44 3.42 3.41 2.80 2.80 2.78 2.77 2.77 2.76 2.74 2.73 2.59 2.58 2.57 2.56 2.55 2.55 2.53 2.30 2.04 2.02 2.01 1.99 1.98 1.59 1.59 1.58 1.57 1.57 1.56 1.55 1.54 1.53 1.53 1.52 1.51 1.50
1H NMR
(CDCl3, 400 MHz)
16g
2.00 2.08 1.00 1.02 1.00 1.99 2.00 1.03 1.01 3.03 2.00 2.07
f1 (ppm)

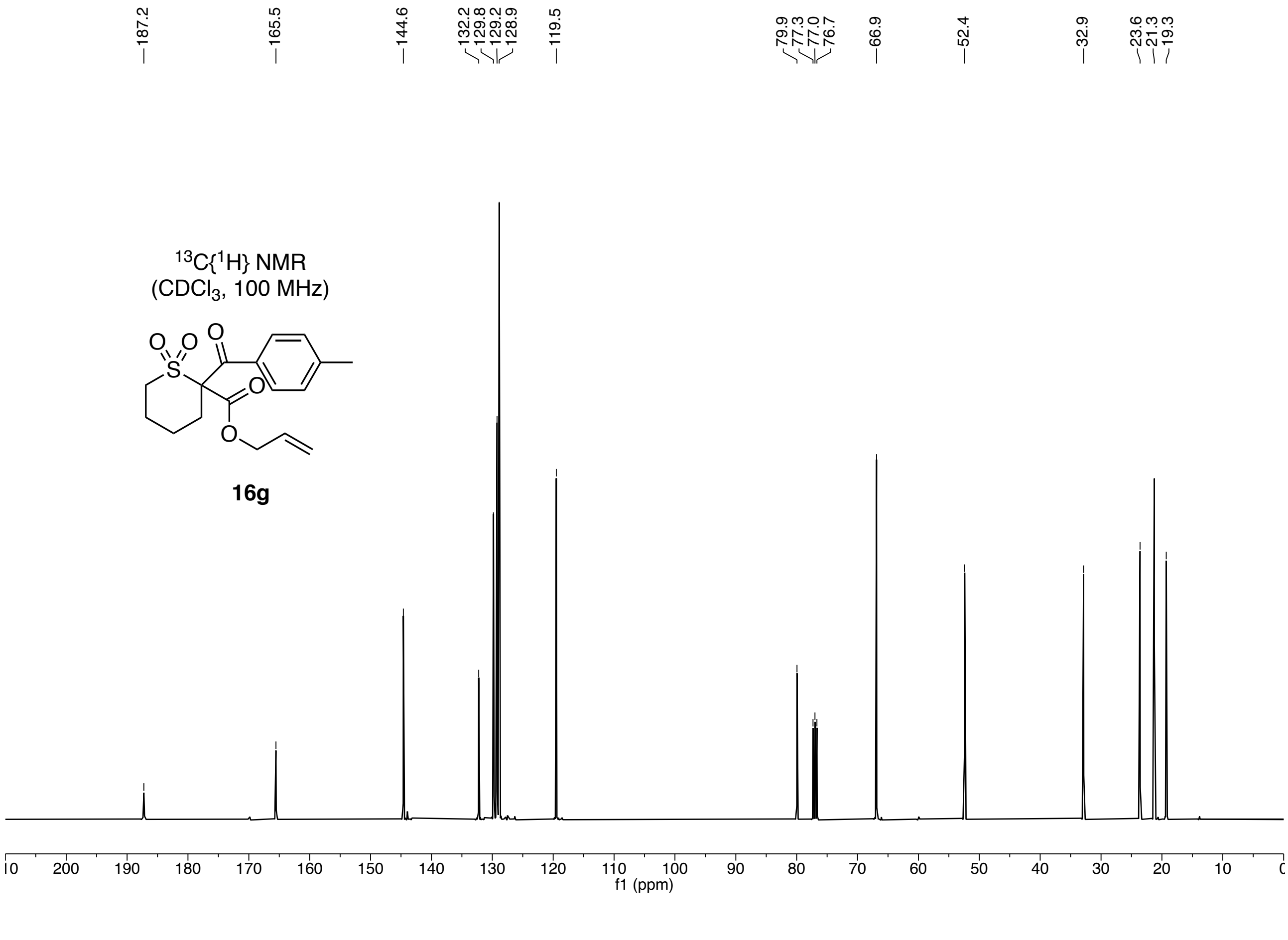
187.2 165.5 144.6 132.2 129.8 129.2 128.9 119.5 79.9 77.3 77.0 76.7 66.9 52.4 32.9 23.6 21.3 19.3
13C{1H} NMR
(CDCl3, 100 MHz)
16g
f1 (ppm)

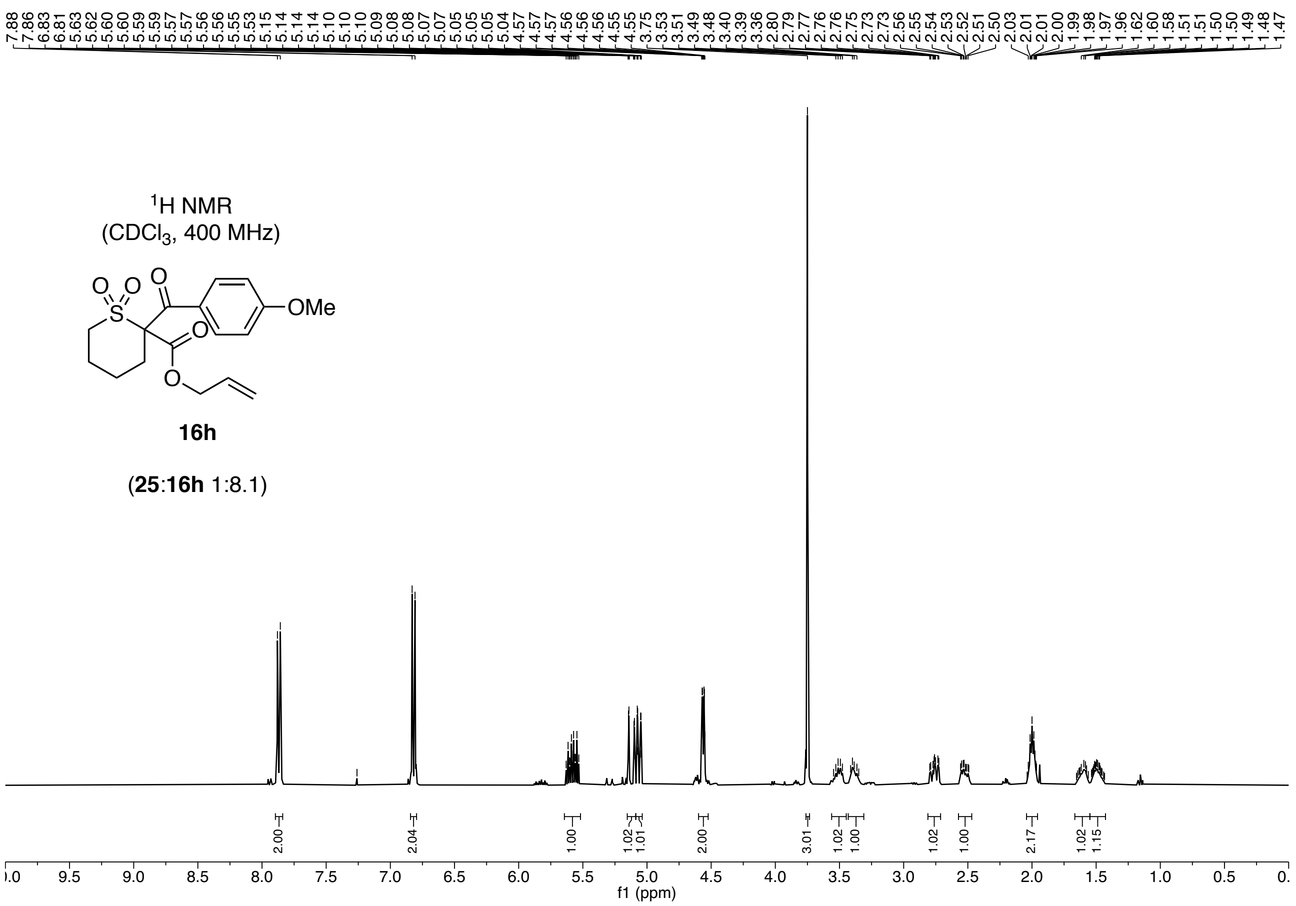
1H NMR
(CDCl3, 400 MHz)
OMe
16h
(25:16h 1:8.1)
f1 (ppm)

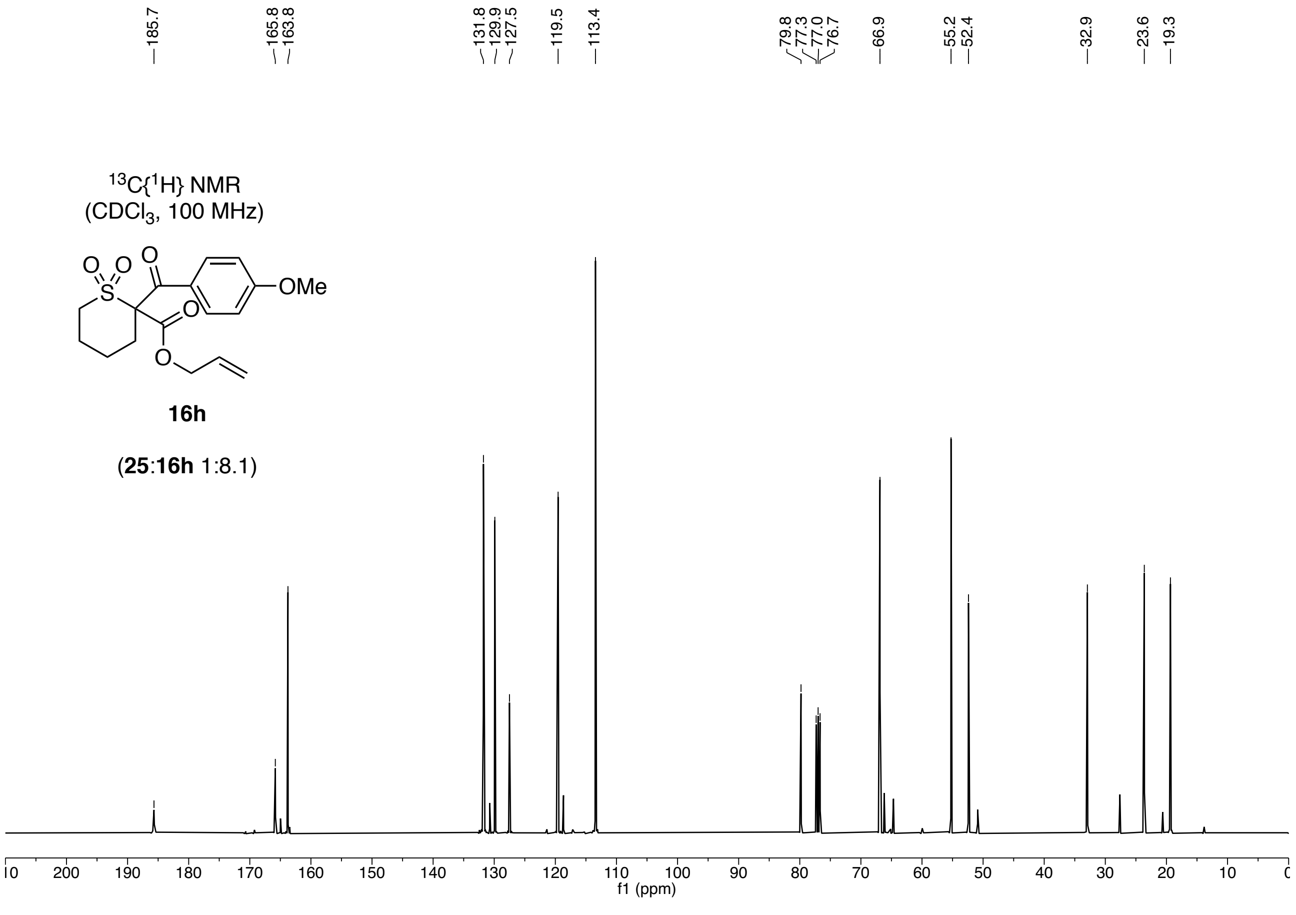
13C{1H} NMR
(CDCl3, 100 MHz)
OMe
16h
(25:16h 1:8.1)
185.7
165.8
163.8
131.8
129.9
127.5
119.5
113.4
79.8
77.3
77.0
76.7
66.9
55.2
52.4
32.9
23.6
19.3
f1 (ppm)

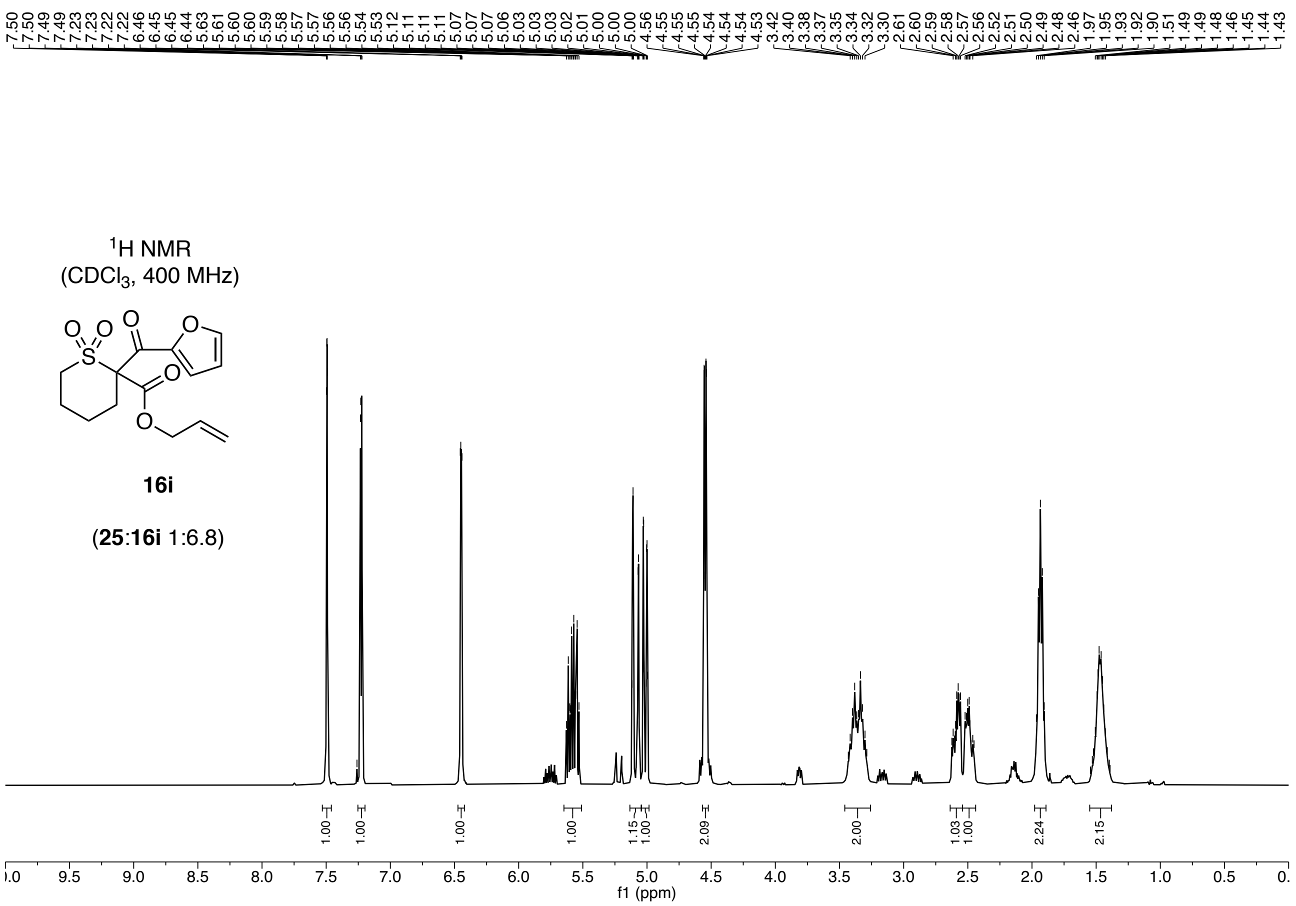
^{1}H NMR
(CDCl$_3$, 400 MHz)
16i
(25:16i 1:6.8)
f1 (ppm)

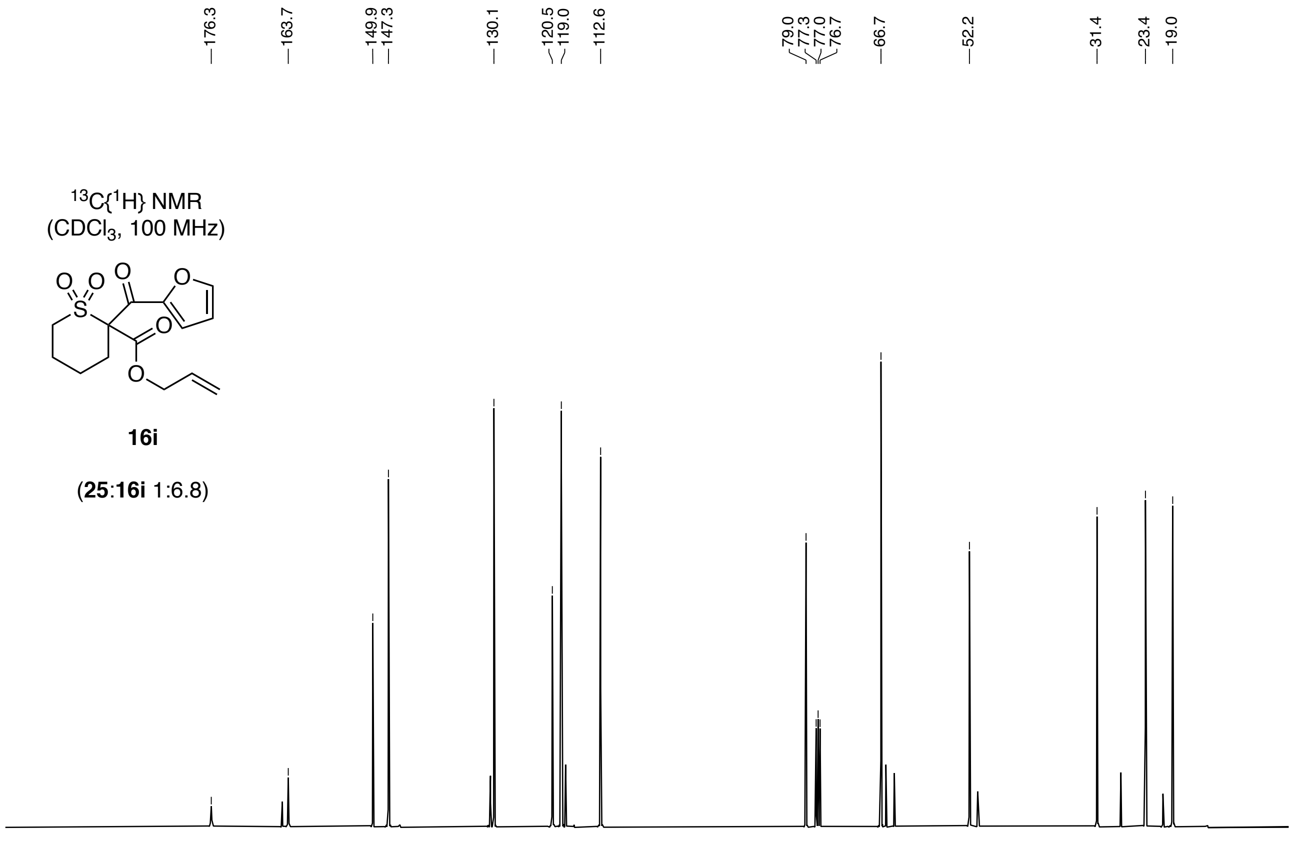
^{13}C{^{1}H} NMR
(CDCl$_3$, 100 MHz)
16i
(25:16i 1:6.8)
176.3
163.7
149.9
147.3
130.1
120.5
119.0
112.6
79.0
77.3
77.0
76.7
66.7
52.2
31.4
23.4
19.0
f1 (ppm)

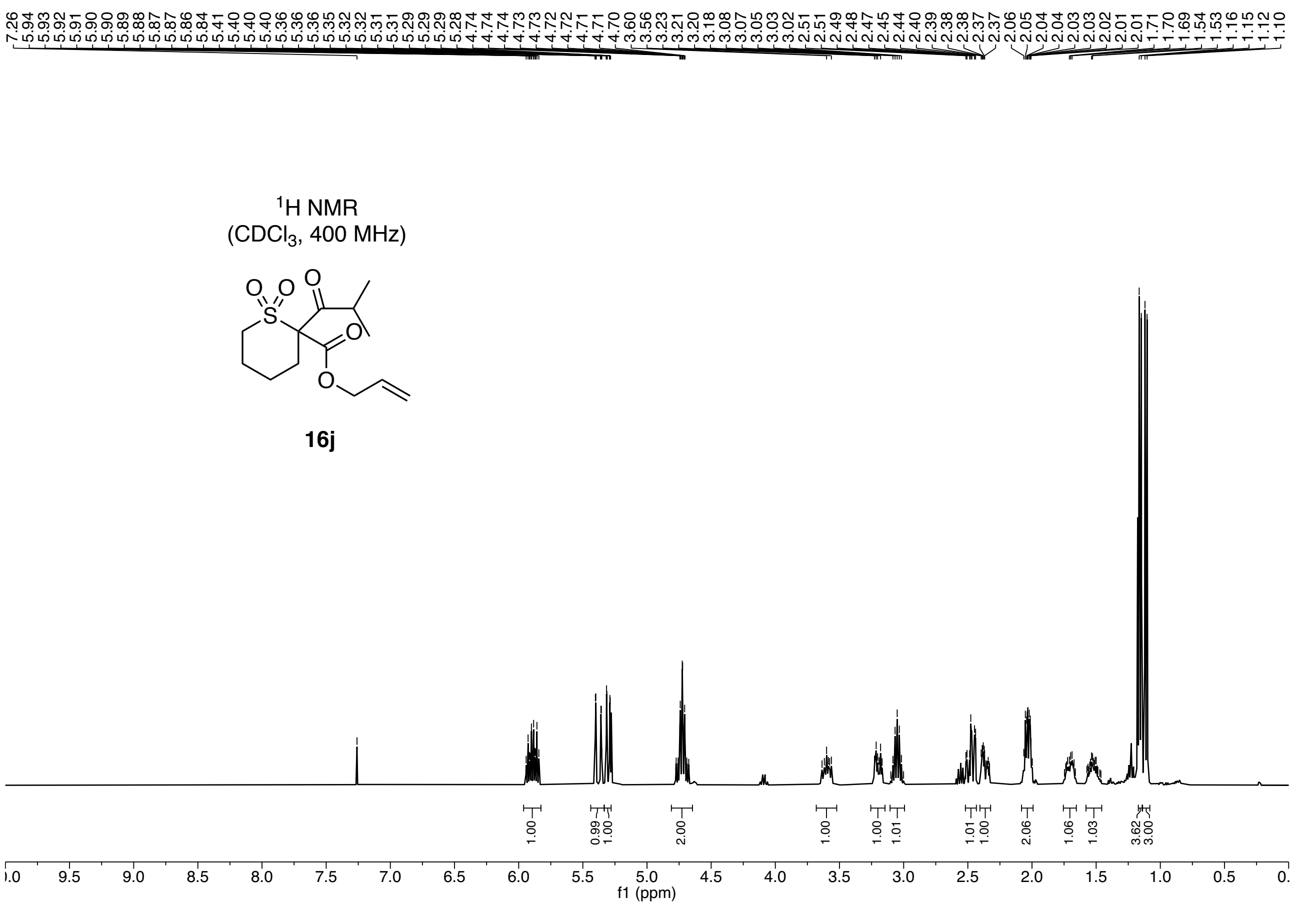
7.26 5.94 5.93 5.92 5.91 5.90 5.89 5.88 5.87 5.86 5.84 5.41 5.40 5.40 5.36 5.36 5.35 5.32 5.32 5.31 5.29 5.29 5.28 4.74 4.74 4.73 4.73 4.72 4.72 4.71 4.70 3.60 3.56 3.23 3.21 3.20 3.18 3.08 3.07 3.05 3.03 3.02 2.51 2.51 2.49 2.48 2.47 2.45 2.44 2.40 2.39 2.38 2.37 2.37 2.06 2.05 2.04 2.04 2.03 2.03 2.02 2.02 2.01 2.01 1.71 1.70 1.69 1.54 1.53 1.16 1.15 1.12 1.10
^{1}H NMR
($CDCl_3$, 400 MHz)
16j
1.00 0.99 1.00 2.00 1.00 1.00 1.01 1.01 1.00 2.06 1.06 1.03 3.62 3.00
f1 (ppm)

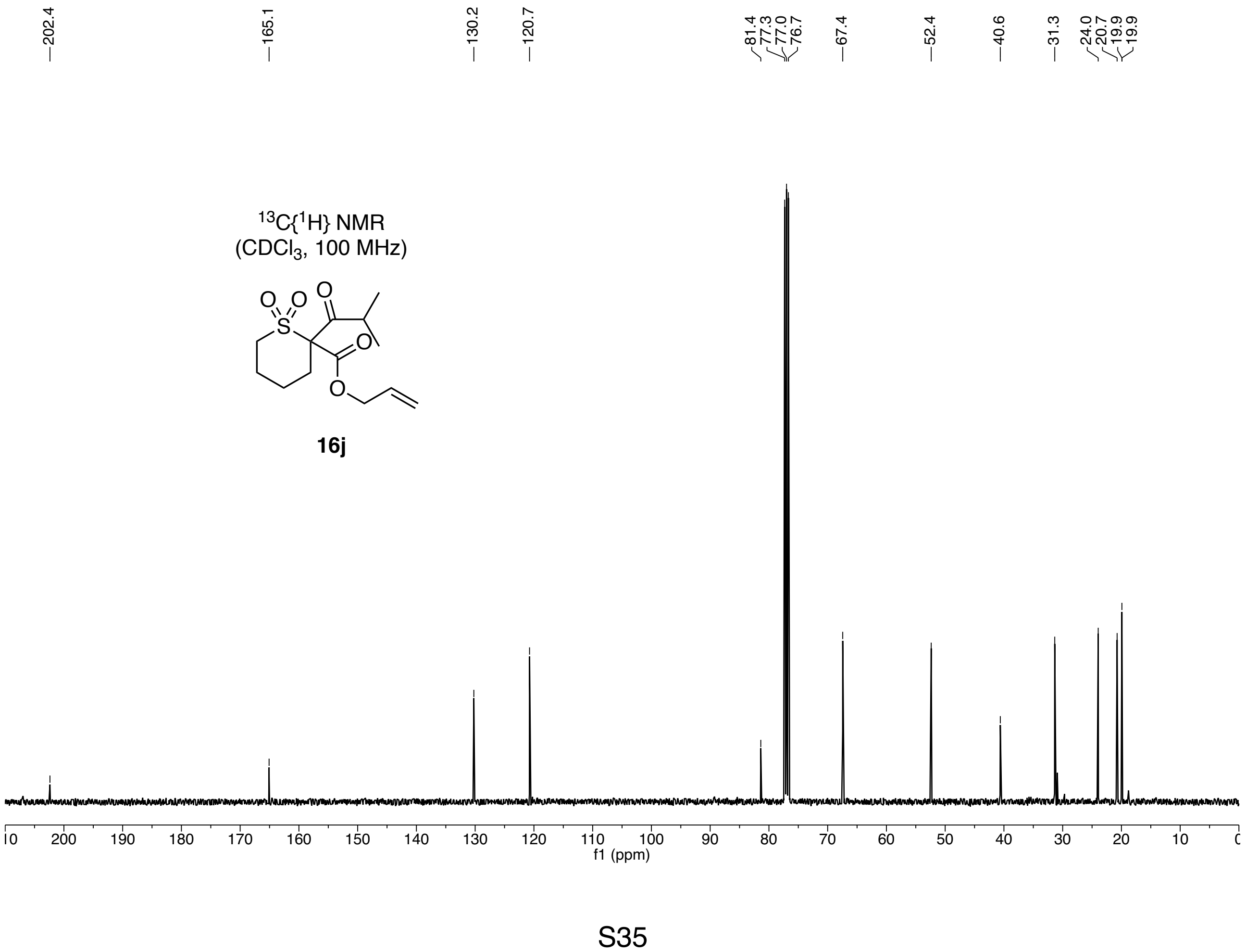
202.4 165.1 130.2 120.7 81.4 77.3 77.0 76.7 67.4 52.4 40.6 31.3 24.0 20.7 19.9 19.9
$^{13}C\{^1H\}$ NMR
($CDCl_3$, 100 MHz)
16j
f1 (ppm)

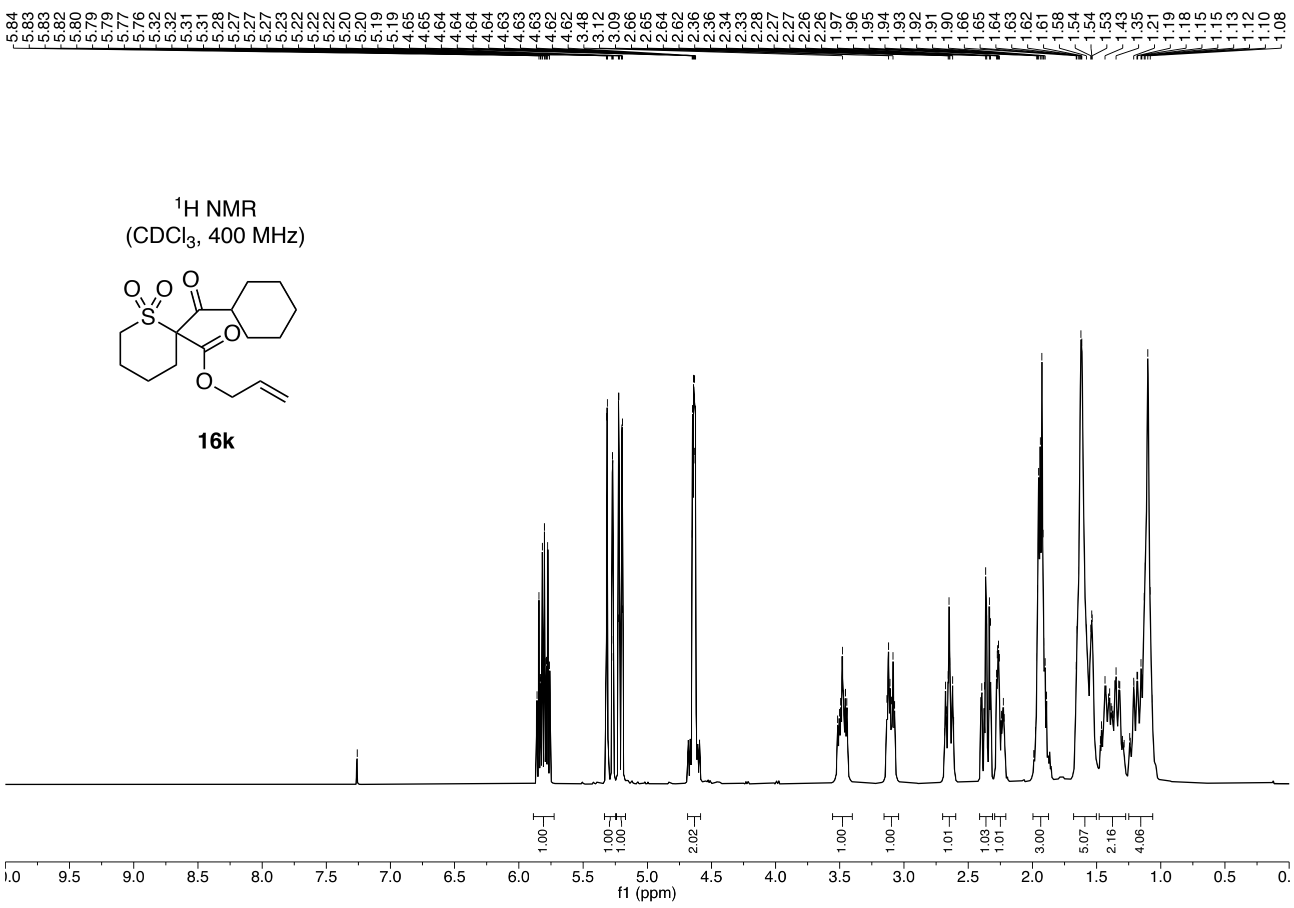
5.84 5.83 5.82 5.80 5.79 5.79 5.77 5.76 5.32 5.32 5.31 5.28 5.27 5.27 5.23 5.22 5.22 5.22 5.20 5.20 5.19 5.19 4.65 4.64 4.64 4.64 4.63 4.63 4.63 4.62 4.62 3.48 3.12 3.09 2.66 2.65 2.64 2.62 2.36 2.36 2.34 2.33 2.28 2.27 2.27 2.26 2.26 1.97 1.96 1.95 1.94 1.93 1.92 1.91 1.90 1.66 1.65 1.64 1.63 1.62 1.61 1.58 1.54 1.54 1.53 1.43 1.35 1.21 1.19 1.18 1.15 1.13 1.12 1.10 1.08
1H NMR
(CDCl3, 400 MHz)
16k
1.00 1.00 1.00 2.02 1.00 1.00 1.01 1.03 1.01 3.00 5.07 2.16 4.06
f1 (ppm)

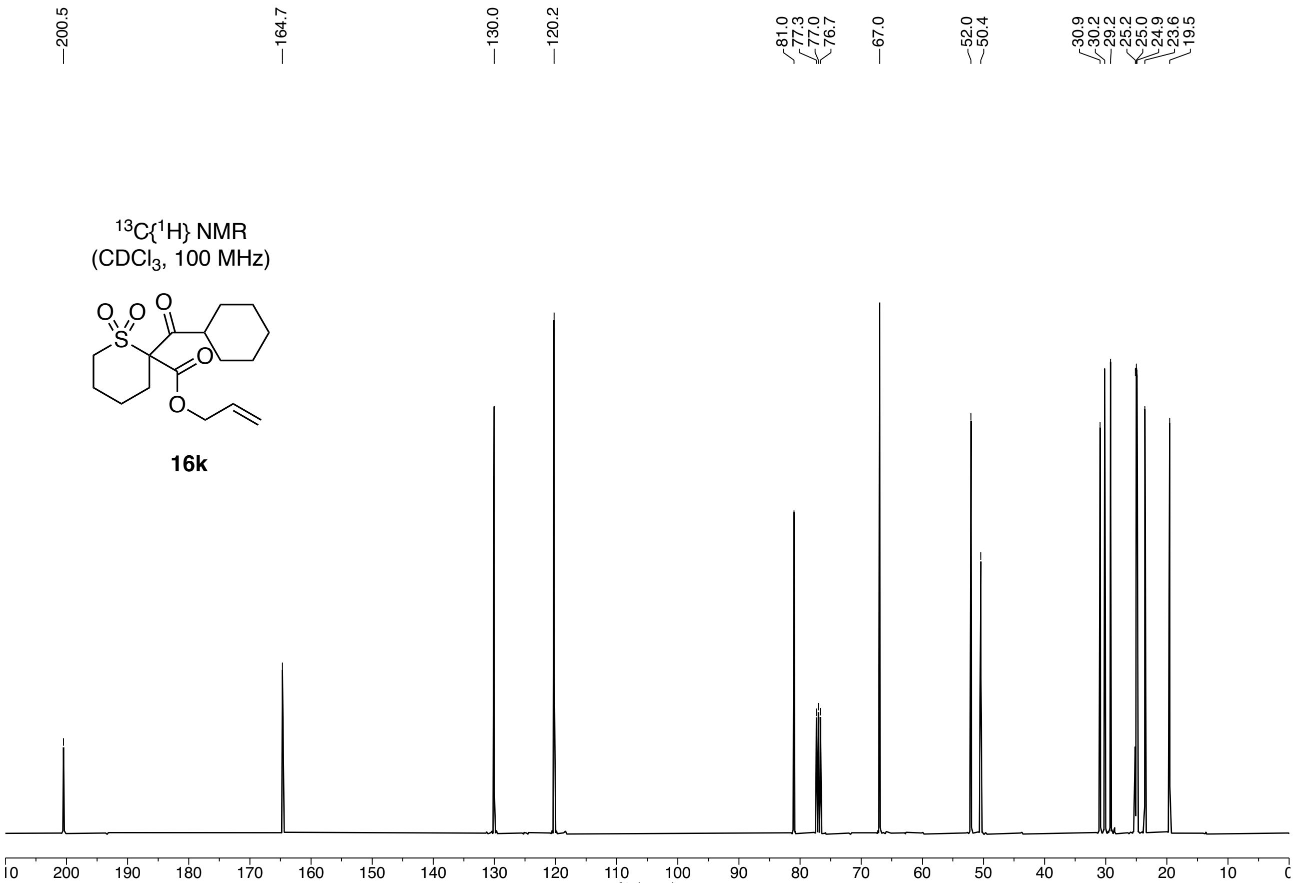
200.5 164.7 130.0 120.2 81.0 77.3 77.0 76.7 67.0 52.0 50.4 30.9 30.2 29.2 25.2 25.0 24.9 23.6 19.5
13C{1H} NMR
(CDCl3, 100 MHz)
16k
f1 (ppm)

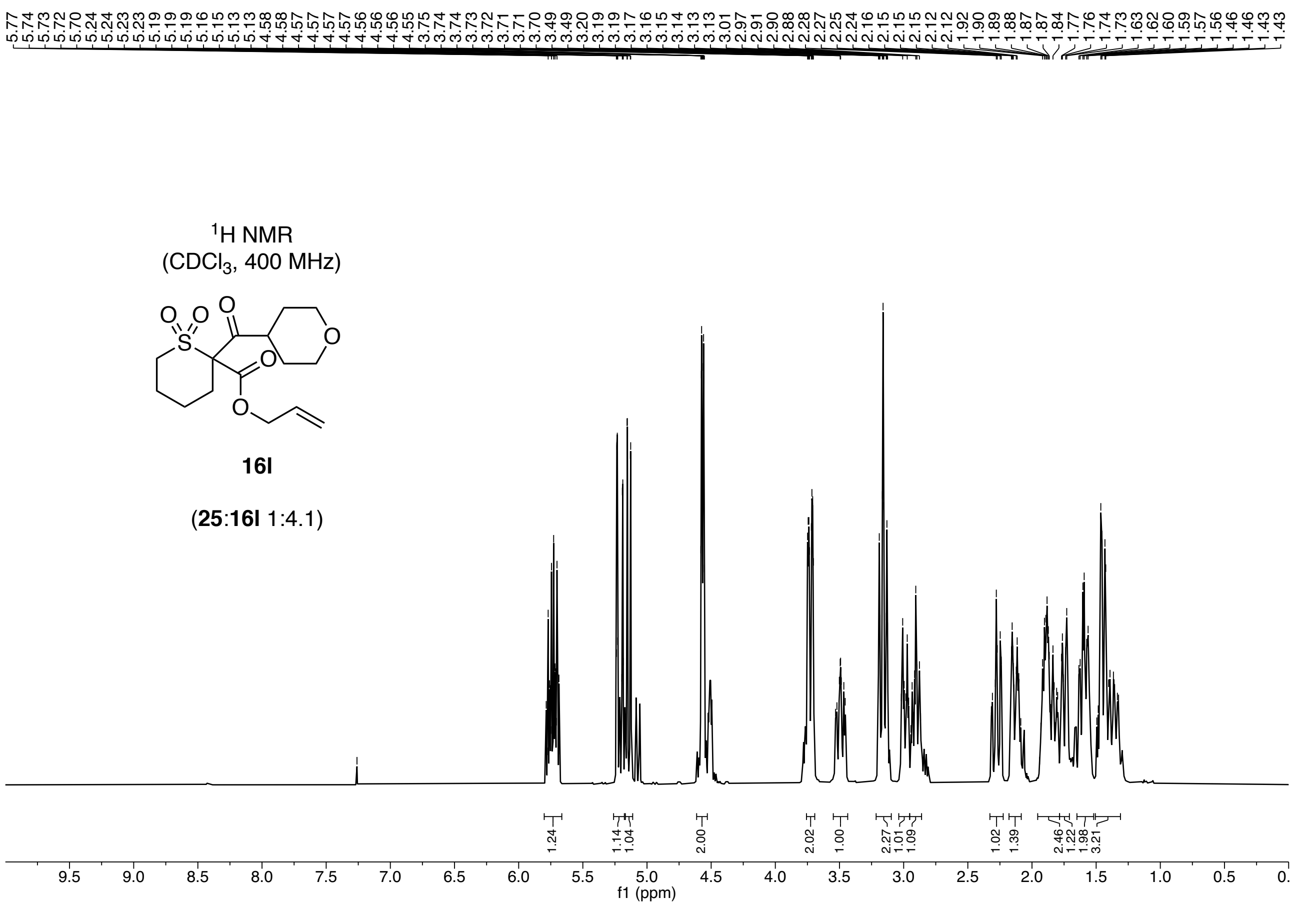
1H NMR
(CDCl3, 400 MHz)
16l
(25:16l 1:4.1)
f1 (ppm)

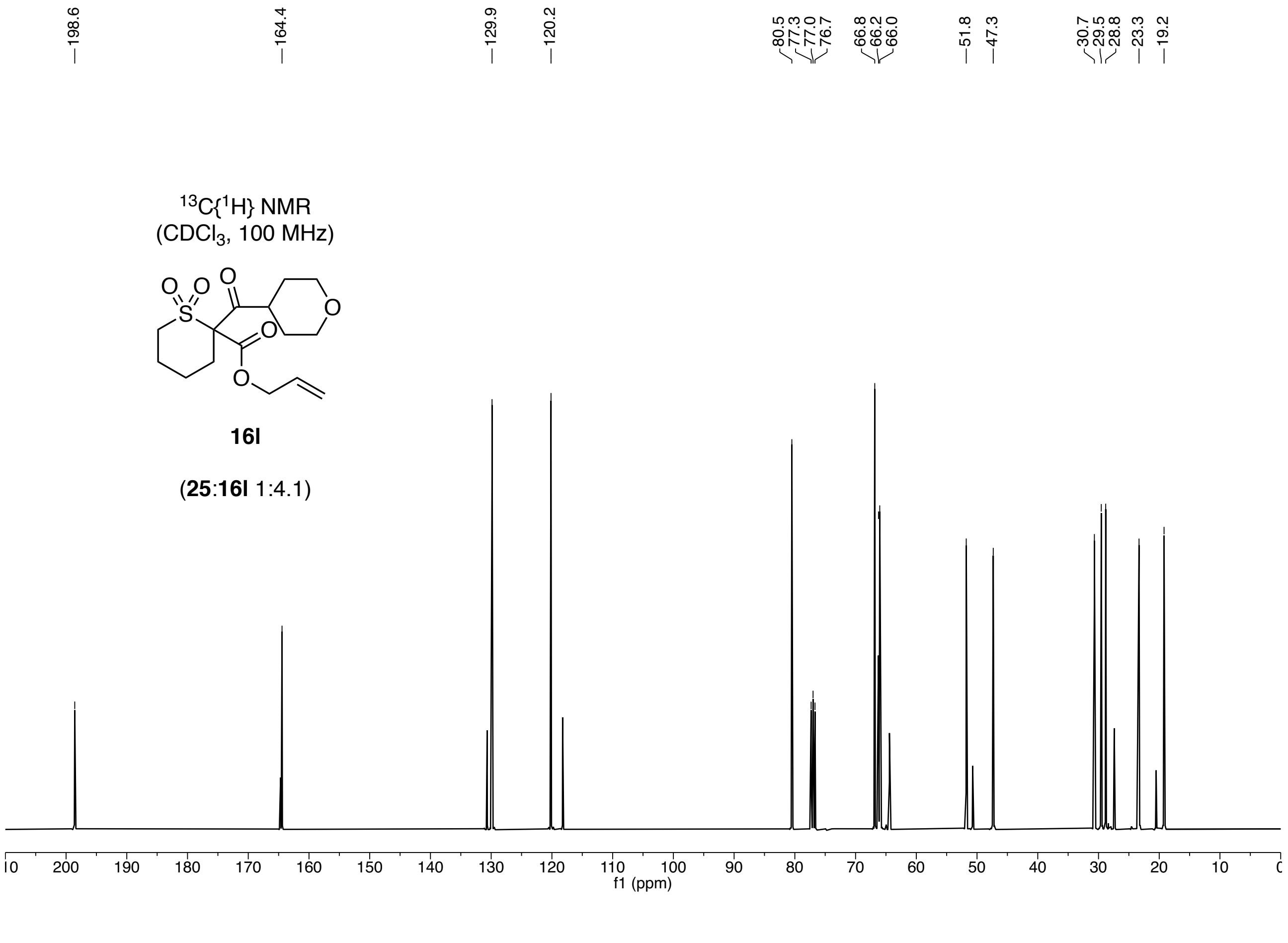
198.6
164.4
129.9
120.2
80.5
77.3
77.0
76.7
66.8
66.2
66.0
51.8
47.3
30.7
29.5
28.8
23.3
19.2
13C{1H} NMR
(CDCl3, 100 MHz)
16l
(25:16l 1:4.1)
f1 (ppm)

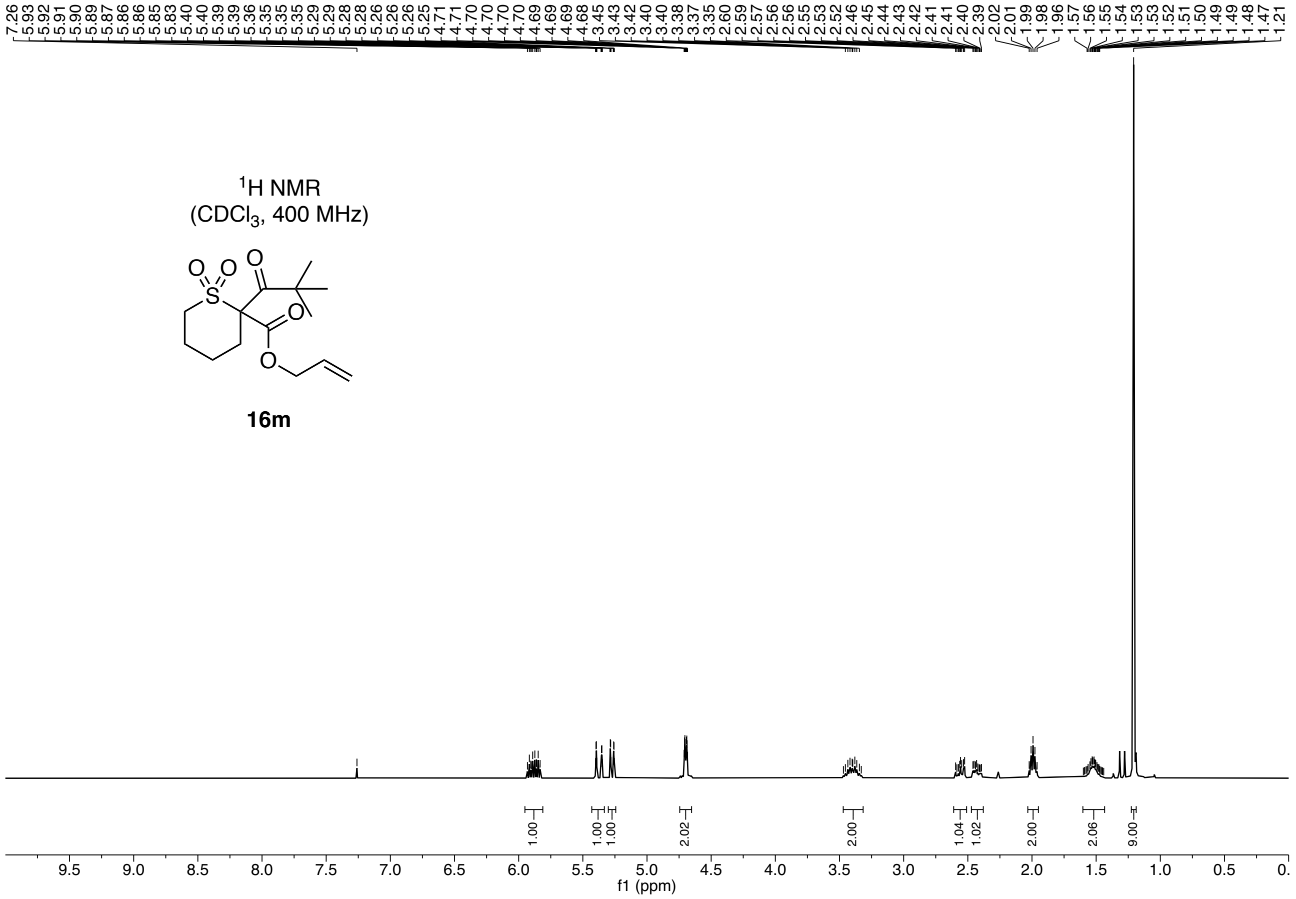
7.26 5.93 5.92 5.91 5.90 5.89 5.87 5.86 5.85 5.83 5.40 5.40 5.39 5.36 5.35 5.35 5.29 5.28 5.28 5.26 5.26 5.25 4.71 4.71 4.70 4.70 4.70 4.69 4.69 4.68 3.45 3.43 3.42 3.40 3.38 3.37 3.35 2.60 2.59 2.57 2.56 2.56 2.55 2.53 2.52 2.46 2.45 2.44 2.43 2.42 2.41 2.41 2.40 2.39 2.02 2.01 1.99 1.98 1.96 1.57 1.56 1.55 1.54 1.53 1.53 1.52 1.51 1.50 1.49 1.49 1.48 1.47 1.21
1H NMR
(CDCl3, 400 MHz)
16m
1.00 1.00 1.00 2.02 2.00 1.04 1.02 2.00 2.06 9.00
f1 (ppm)

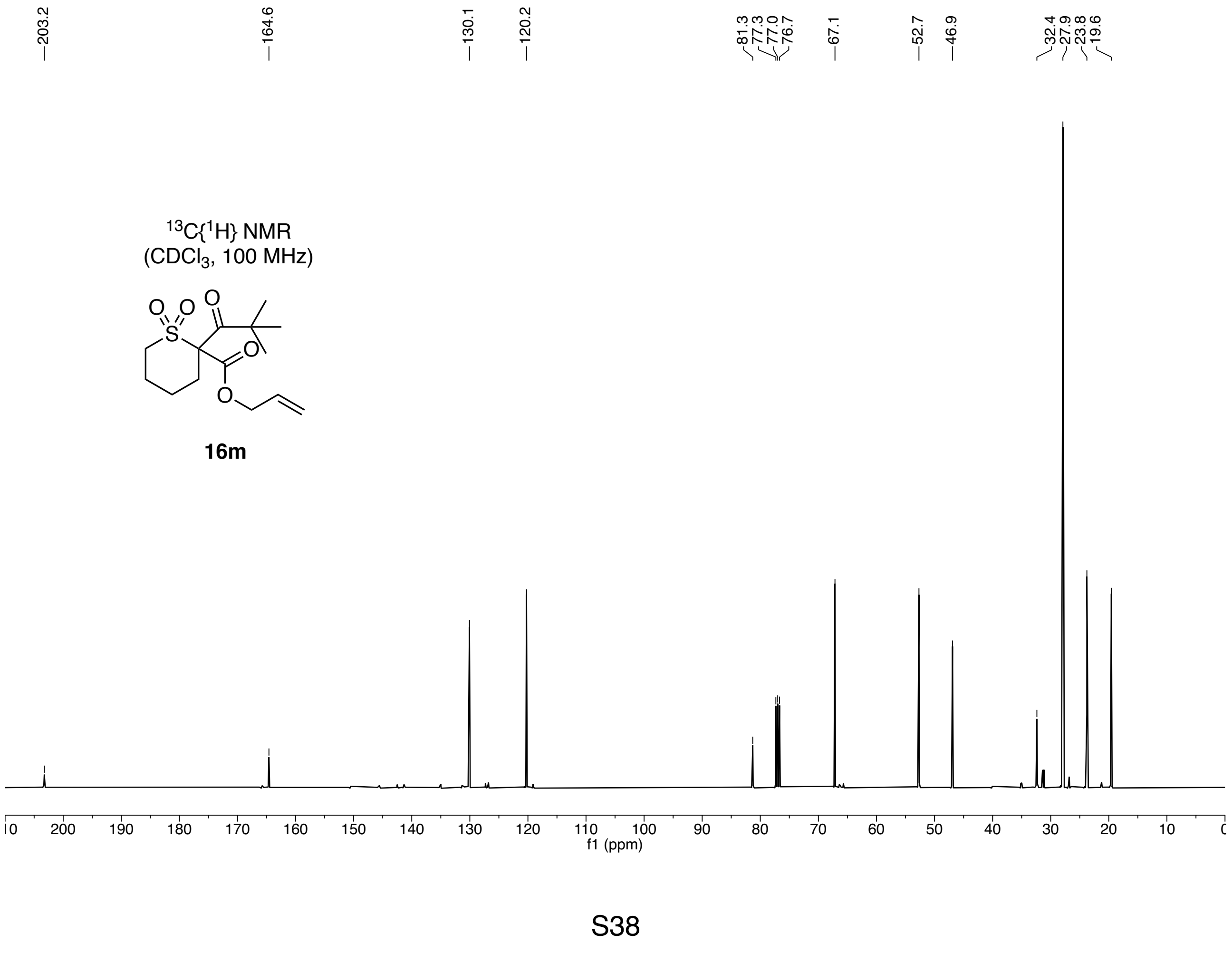
203.2 164.6 130.1 120.2 81.3 77.3 77.0 76.7 67.1 52.7 46.9 32.4 27.9 23.8 19.6
13C{1H} NMR
(CDCl3, 100 MHz)
16m
f1 (ppm)

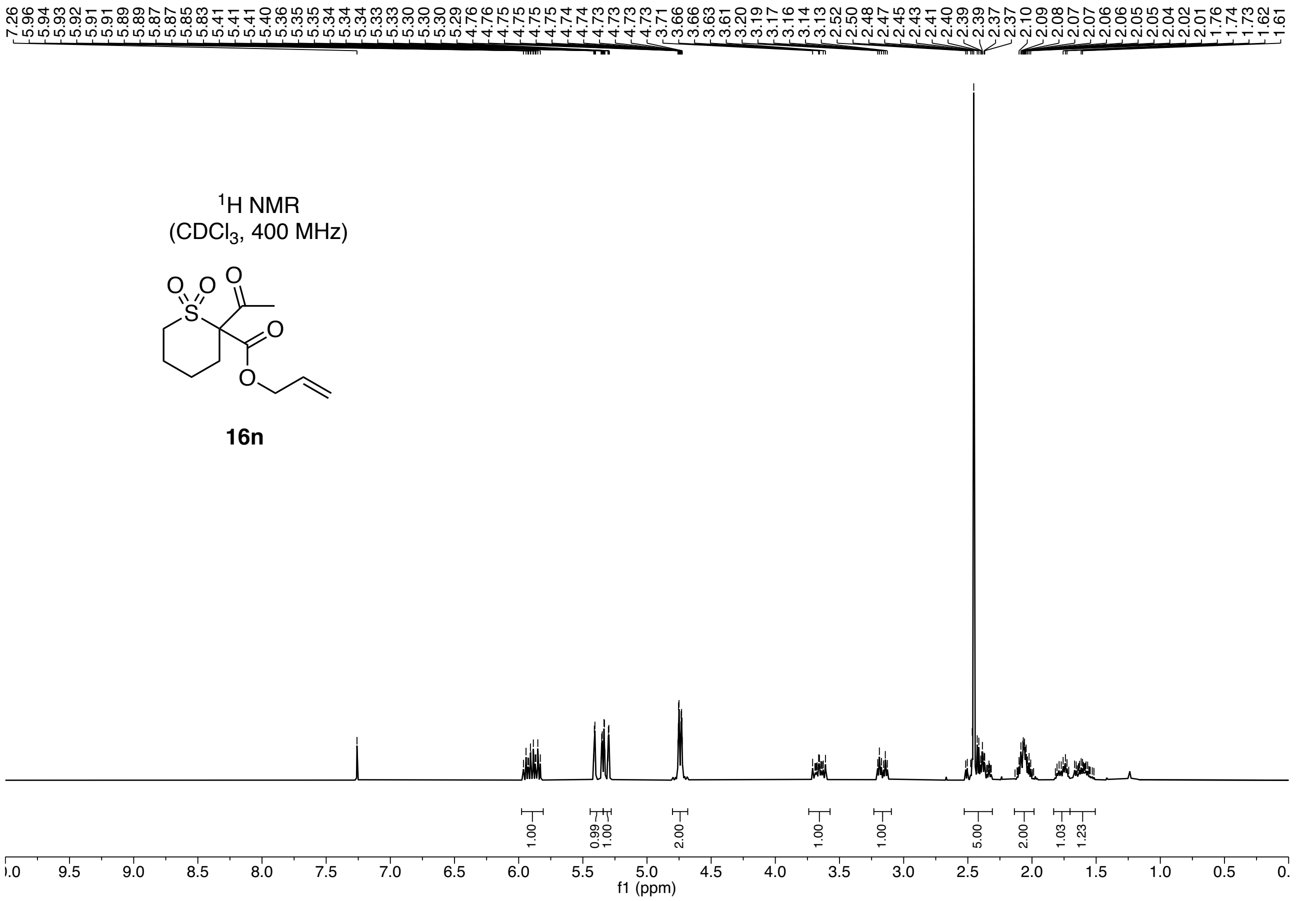

7.26 5.96 5.94 5.93 5.92 5.91 5.89 5.89 5.87 5.85 5.83 5.41 5.41 5.41 5.40 5.36 5.35 5.34 5.34 5.34 5.33 5.33 5.30 5.30 5.29 4.76 4.75 4.75 4.75 4.74 4.74 4.73 4.73 4.73 4.71 3.71 3.66 3.66 3.63 3.61 3.20 3.19 3.17 3.16 3.14 3.13 2.52 2.50 2.48 2.47 2.45 2.43 2.41 2.40 2.39 2.39 2.37 2.37 2.10 2.09 2.08 2.07 2.06 2.06 2.05 2.05 2.04 2.02 2.01 1.76 1.74 1.73 1.62 1.61
^{1}H NMR
(CDCl$_3$, 400 MHz)
O O O S O O
16n
1.00 0.99 1.00 2.00 1.00 1.00 5.00 2.00 1.03 1.23
f1 (ppm)

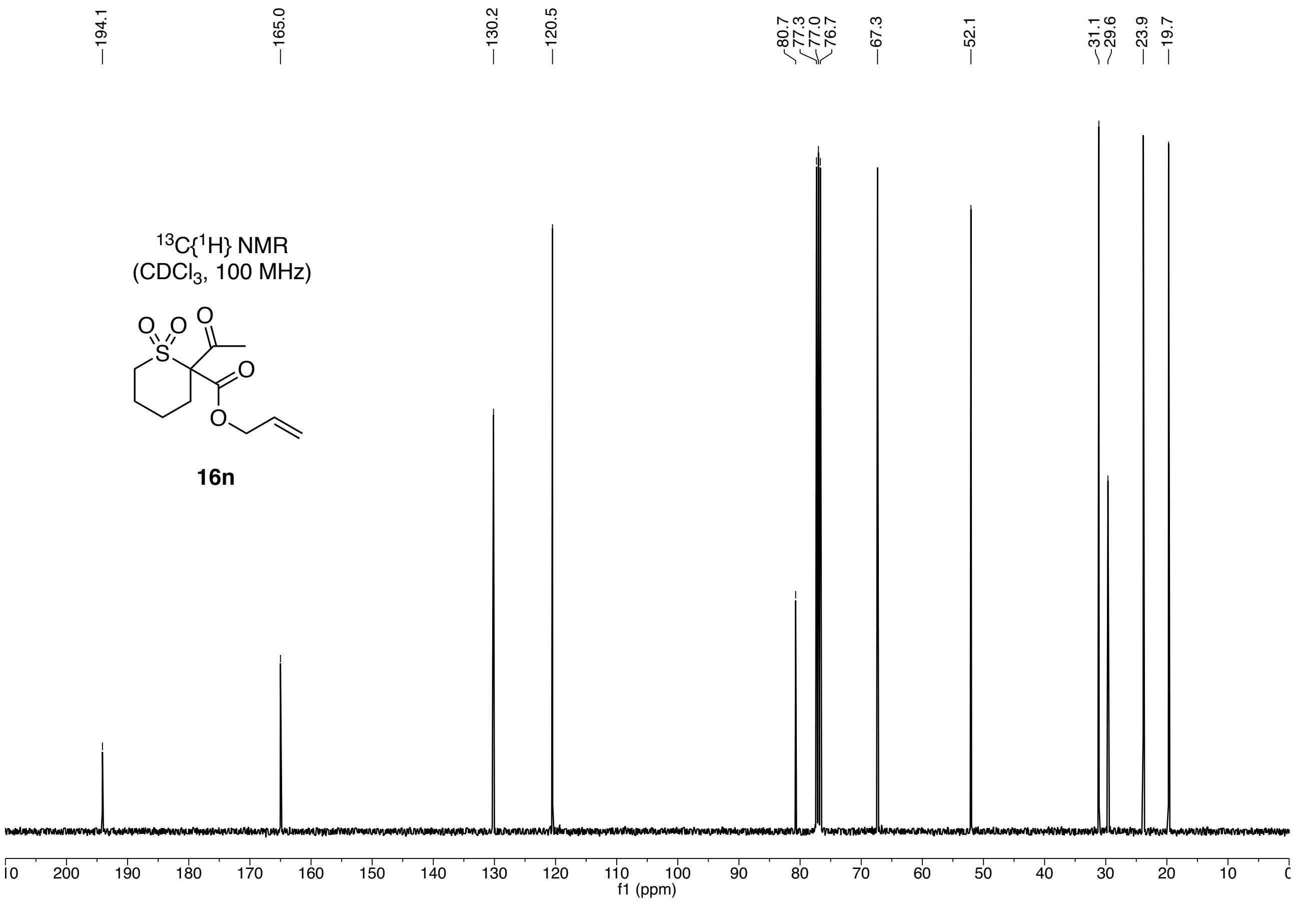

194.1 165.0 130.2 120.5 80.7 77.3 77.0 76.7 67.3 52.1 31.1 29.6 23.9 19.7
^{13}C{^{1}H} NMR
(CDCl$_3$, 100 MHz)
O O O S O O
16n
f1 (ppm)

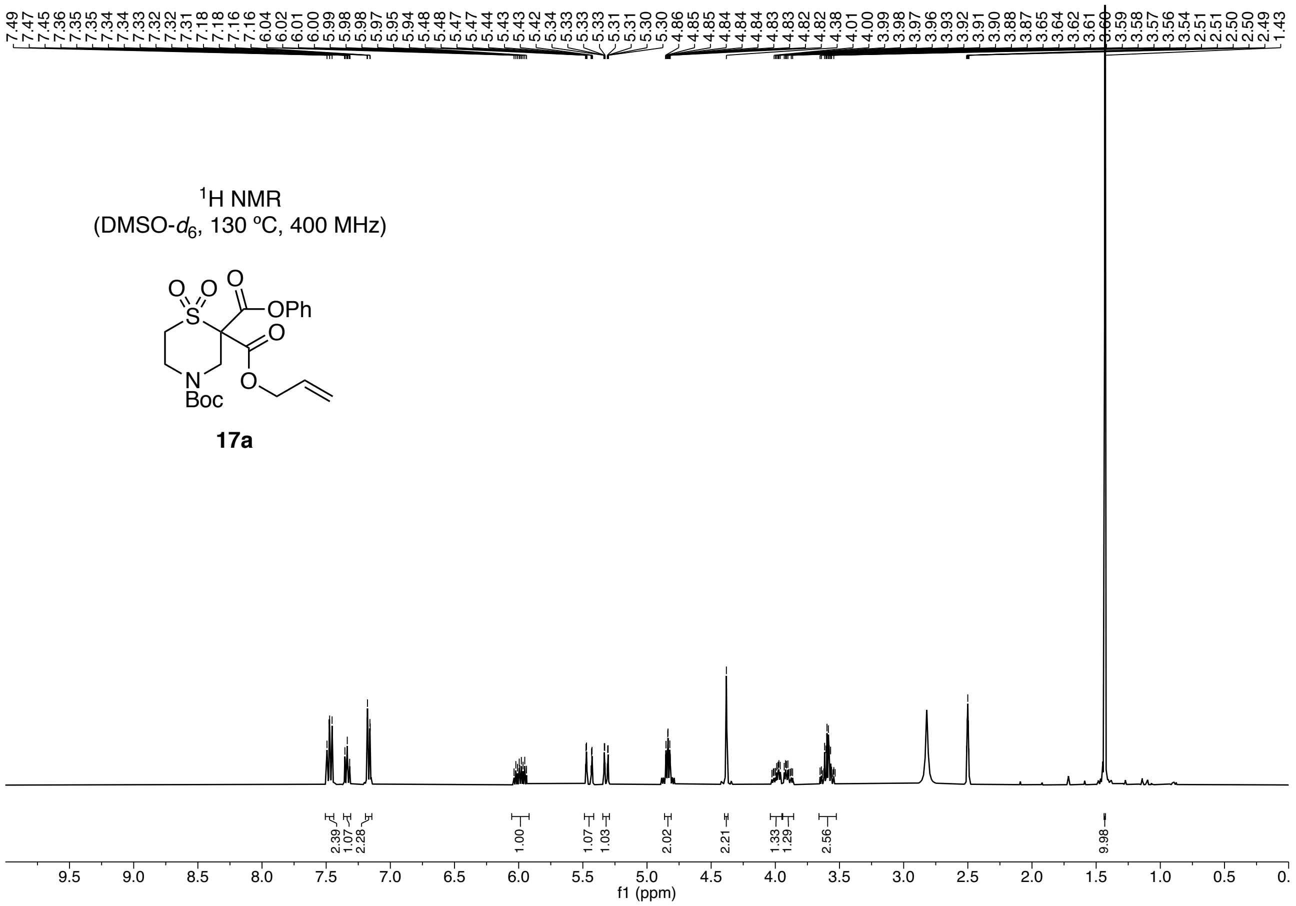
7.49 7.47 7.45 7.36 7.35 7.34 7.34 7.33 7.32 7.32 7.31 7.18 7.18 7.16 7.16 6.04 6.02 6.01 6.00 5.99 5.98 5.98 5.97 5.95 5.94 5.48 5.48 5.47 5.44 5.43 5.43 5.42 5.34 5.33 5.33 5.33 5.31 5.31 5.30 5.30 4.86 4.85 4.85 4.84 4.84 4.84 4.83 4.83 4.82 4.82 4.38 4.01 4.00 3.99 3.98 3.97 3.96 3.93 3.92 3.91 3.90 3.88 3.87 3.65 3.64 3.62 3.61 3.59 3.58 3.57 3.56 3.54 2.51 2.50 2.50 2.49 1.43
1H NMR
(DMSO-d6, 130 °C, 400 MHz)
O O O
S
OPh
O
N
O
Boc
17a
2.39 1.07 2.28 1.00 1.07 1.03 2.02 2.21 1.33 1.29 2.56 9.98
9.5 9.0 8.5 8.0 7.5 7.0 6.5 6.0 5.5 5.0 4.5 4.0 3.5 3.0 2.5 2.0 1.5 1.0 0.5
f1 (ppm)

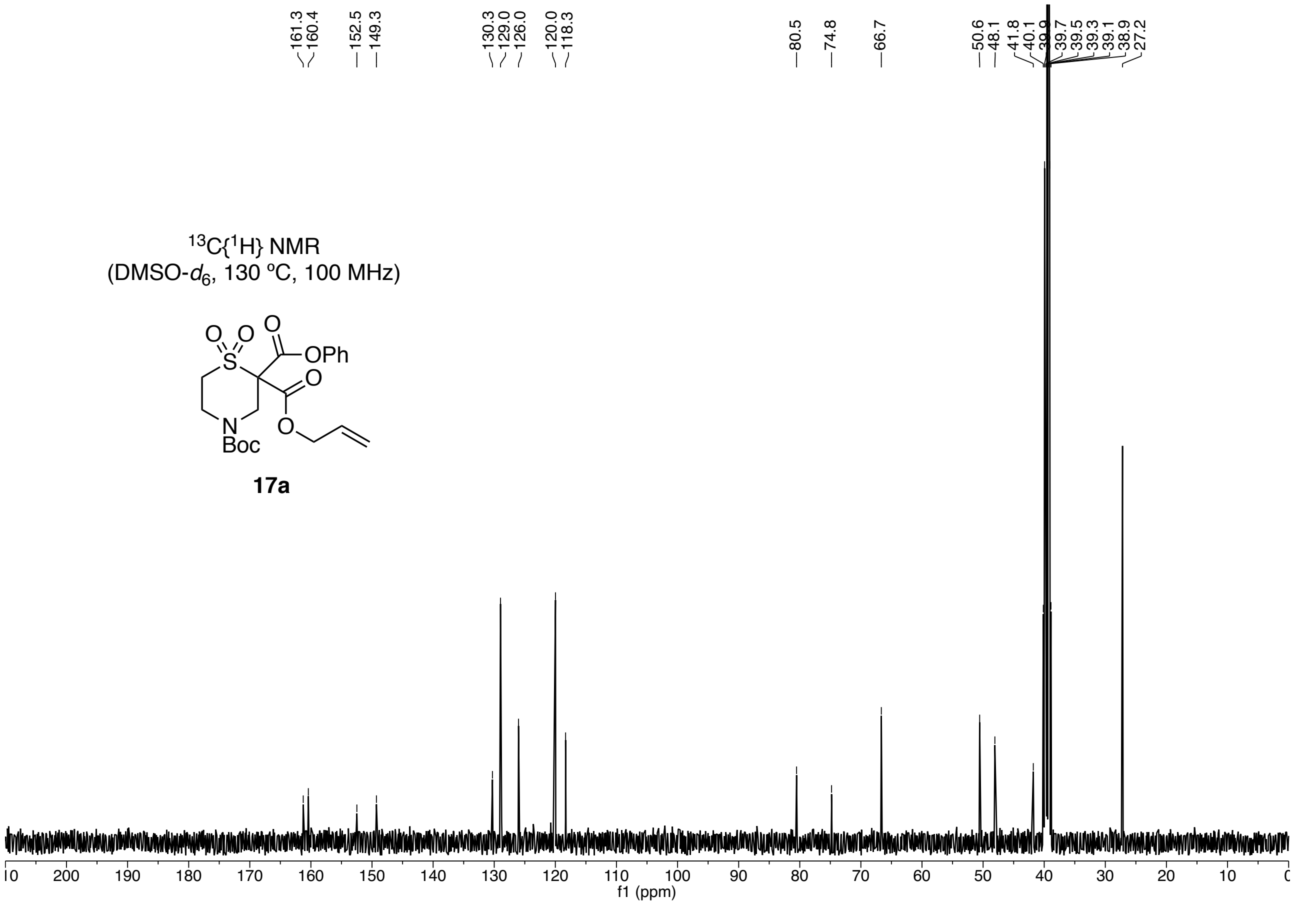
161.3 160.4 152.5 149.3 130.3 129.0 126.0 120.0 118.3 80.5 74.8 66.7 50.6 48.1 41.8 40.1 39.9 39.7 39.5 39.3 39.1 38.9 27.2
13C{1H} NMR
(DMSO-d6, 130 °C, 100 MHz)
O O O
S
OPh
O
N
O
Boc
17a
200 190 180 170 160 150 140 130 120 110 100 90 80 70 60 50 40 30 20 10
f1 (ppm)

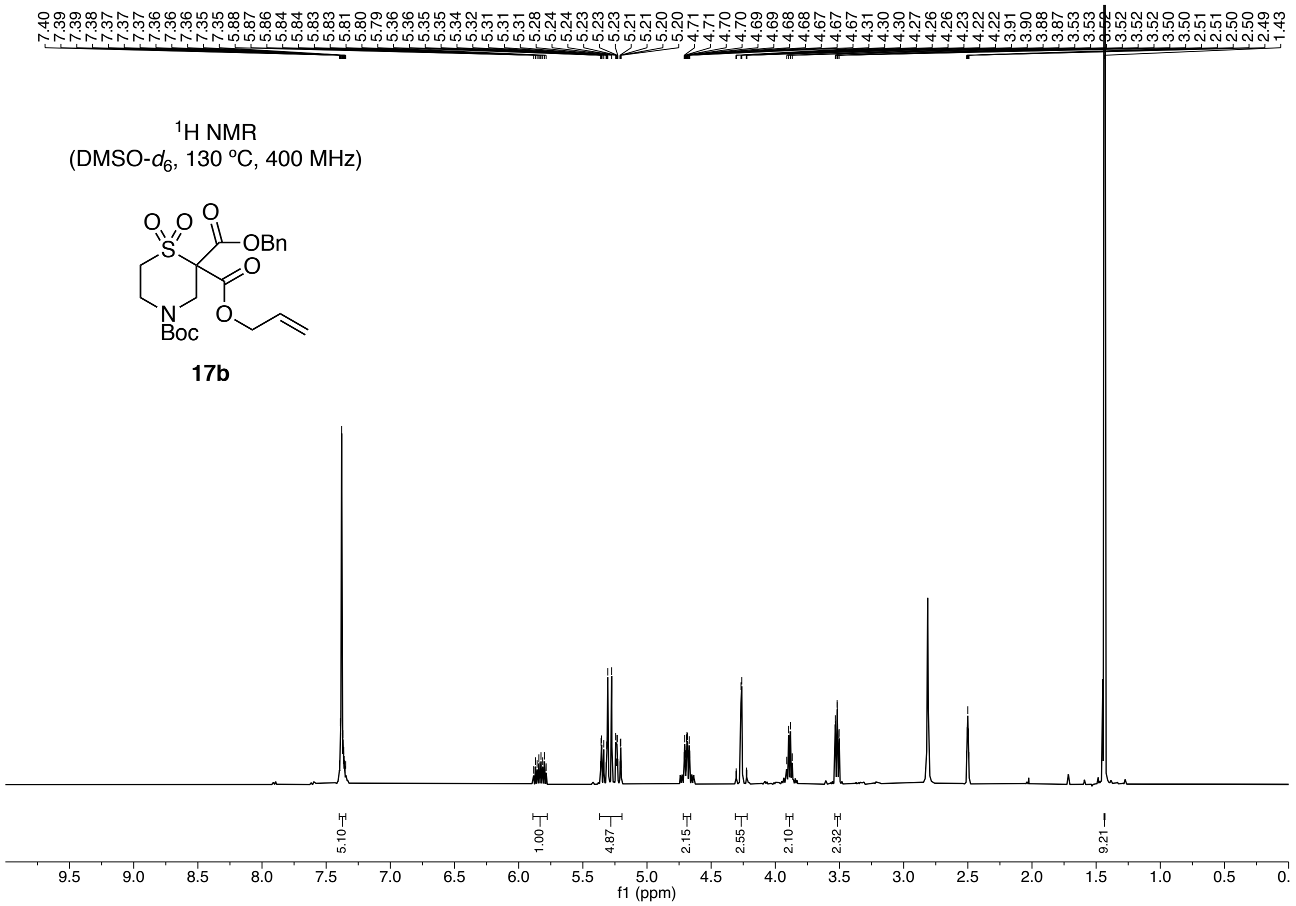

^1H NMR
(DMSO-d_6, 130 °C, 400 MHz)

17b

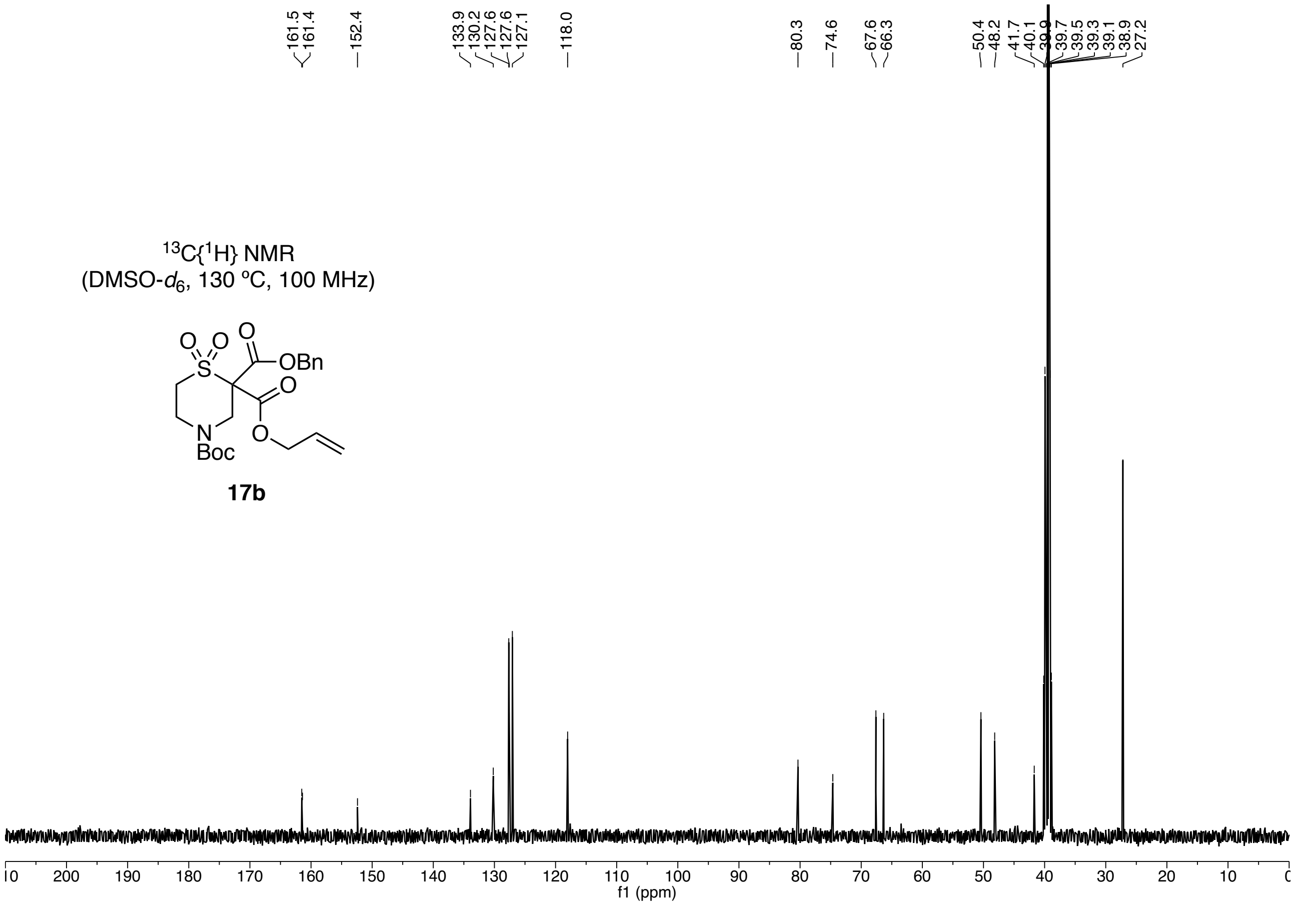

$^{13}C\{^1H\}$ NMR
(DMSO-d_6, 130 °C, 100 MHz)

17b

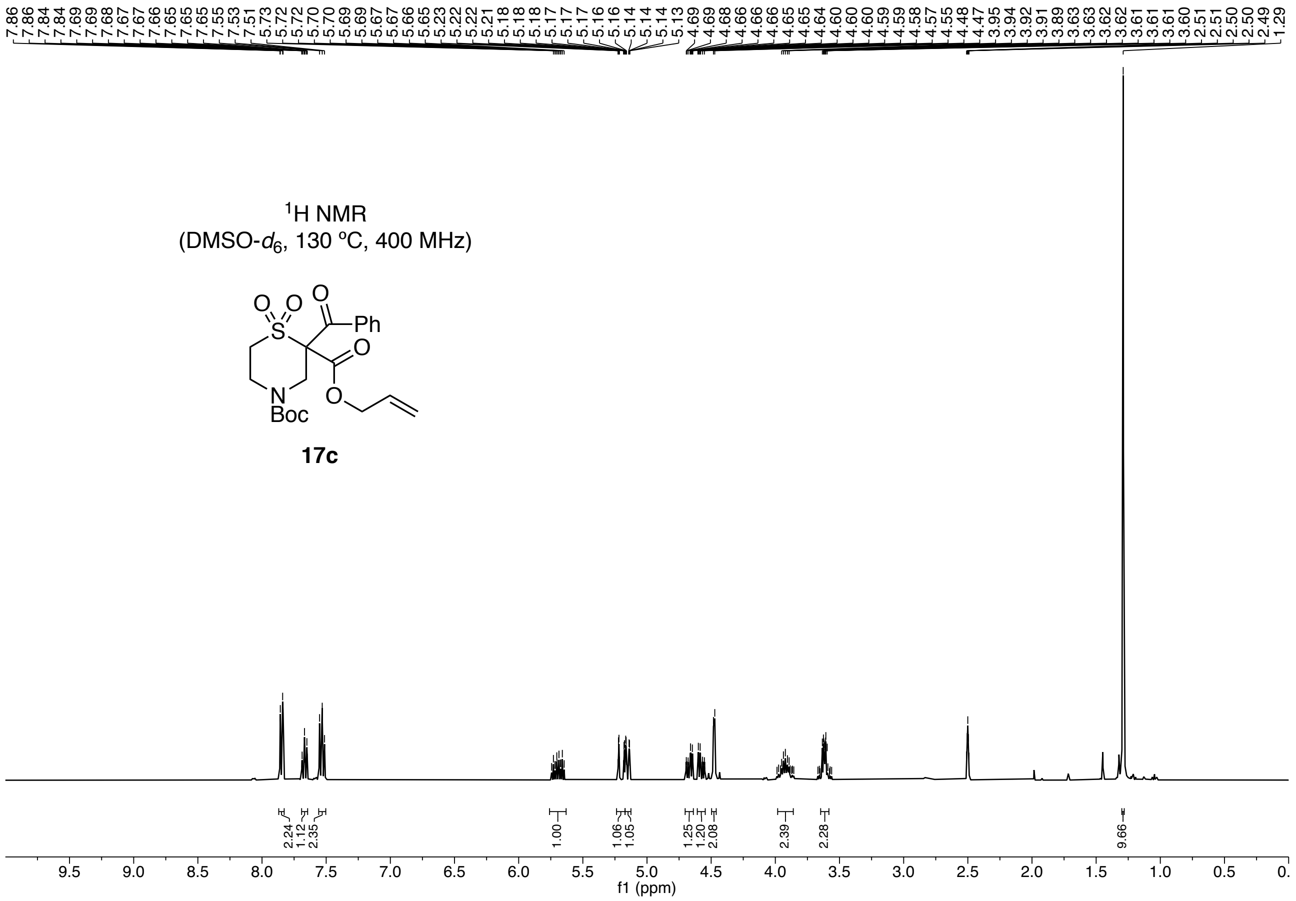

^{1}H NMR
(DMSO-d_6, 130 ºC, 400 MHz)
17c
f1 (ppm)

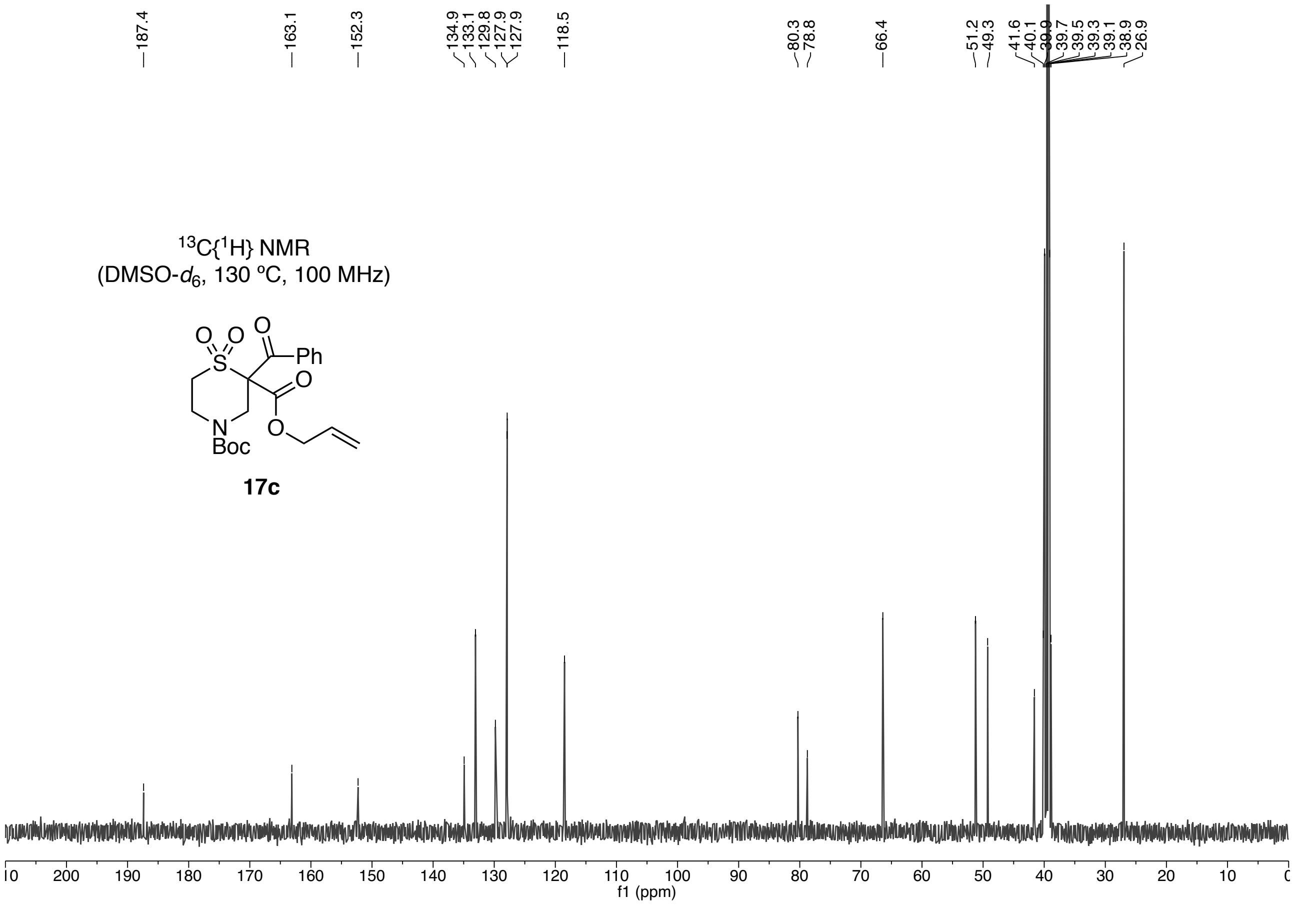

^{13}C{^{1}H} NMR
(DMSO-d_6, 130 ºC, 100 MHz)
17c
f1 (ppm)

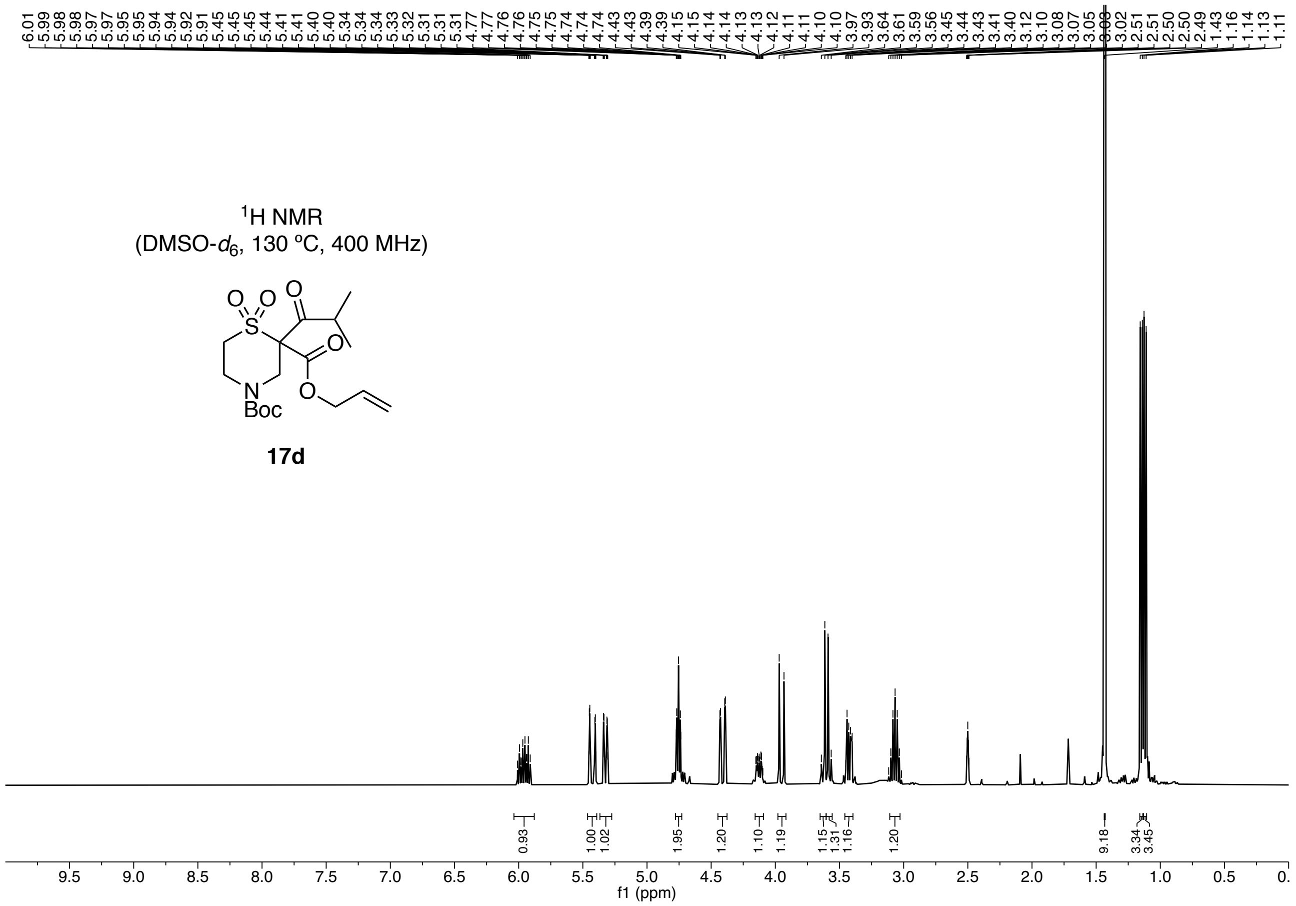

^{1}H NMR
(DMSO-d_6, 130 °C, 400 MHz)
17d
f1 (ppm)

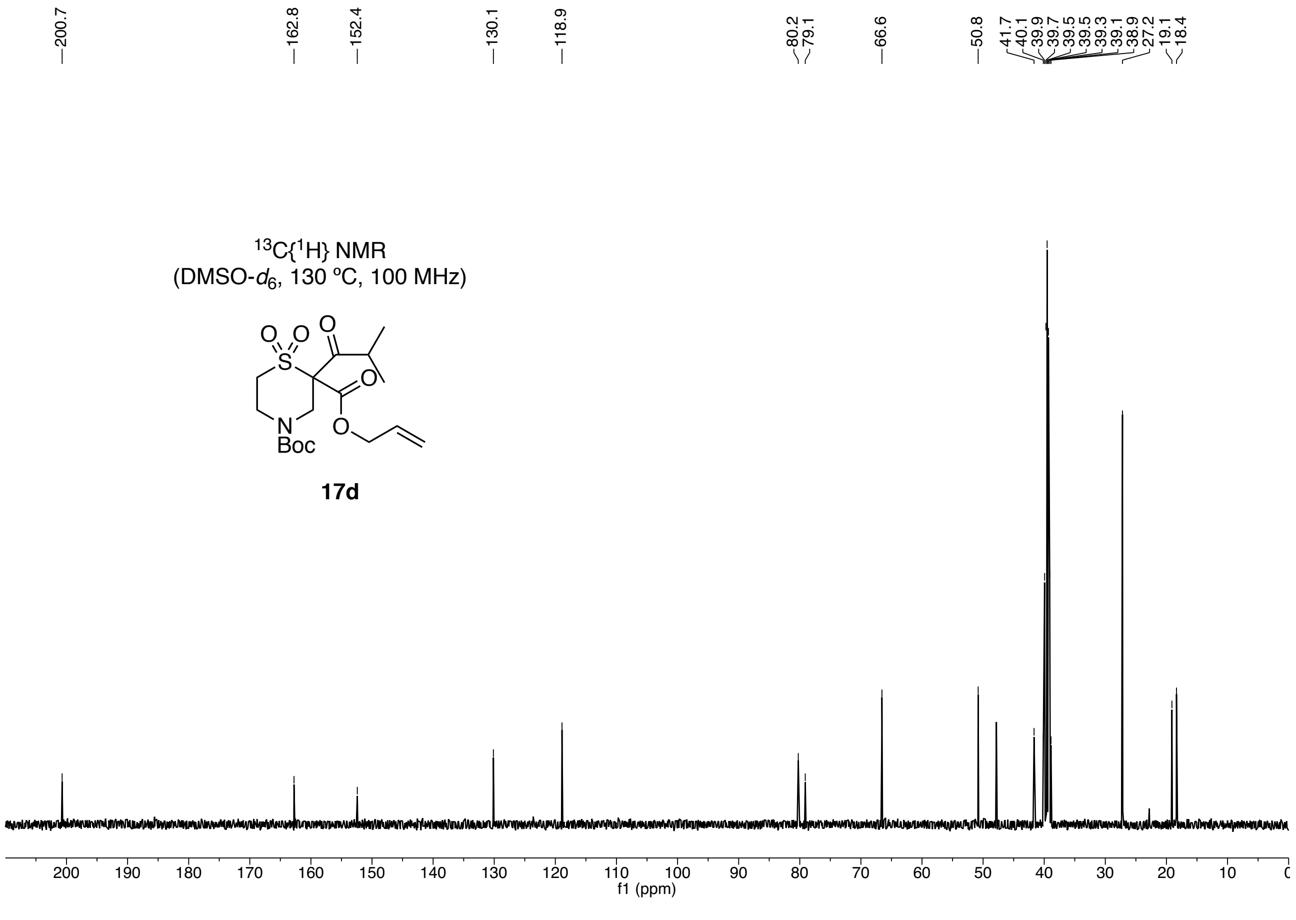

^{13}C{^{1}H} NMR
(DMSO-d_6, 130 °C, 100 MHz)
17d
f1 (ppm)

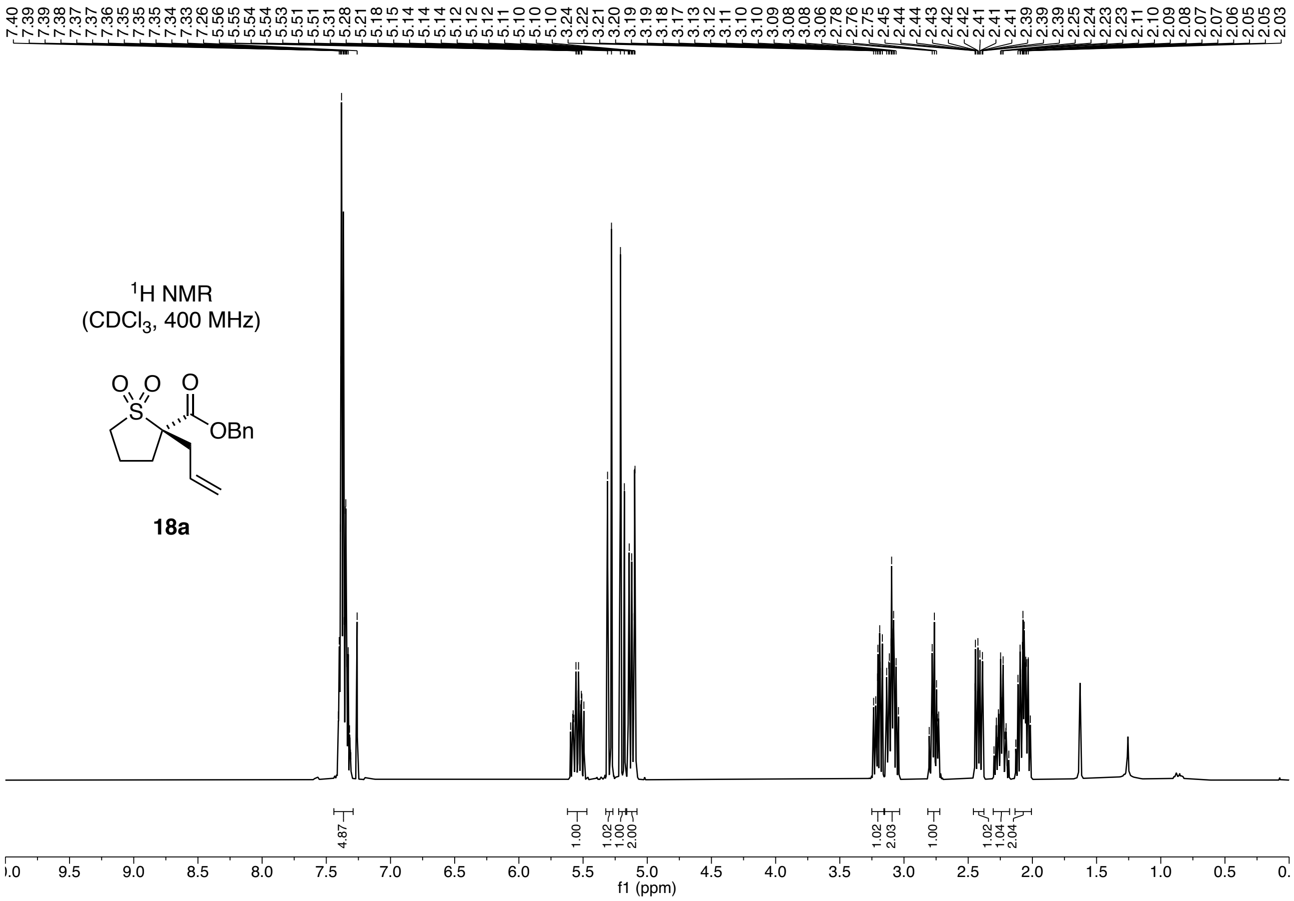

7.40 7.39 7.38 7.37 7.37 7.36 7.35 7.35 7.34 7.33 7.26 5.56 5.55 5.54 5.54 5.53 5.51 5.31 5.28 5.21 5.18 5.15 5.14 5.14 5.12 5.12 5.12 5.11 5.10 5.10 5.10 3.24 3.22 3.21 3.20 3.19 3.19 3.18 3.17 3.13 3.12 3.11 3.10 3.10 3.09 3.08 3.06 2.78 2.76 2.75 2.45 2.44 2.44 2.43 2.42 2.42 2.41 2.41 2.41 2.39 2.39 2.25 2.24 2.23 2.23 2.11 2.10 2.09 2.08 2.07 2.06 2.05 2.03
^{1}H NMR
($CDCl_3$, 400 MHz)
O O O
S
OBn
18a
4.87
1.00
1.02
1.00
2.00
1.02
2.03
1.00
1.02
1.04
2.04
f1 (ppm)

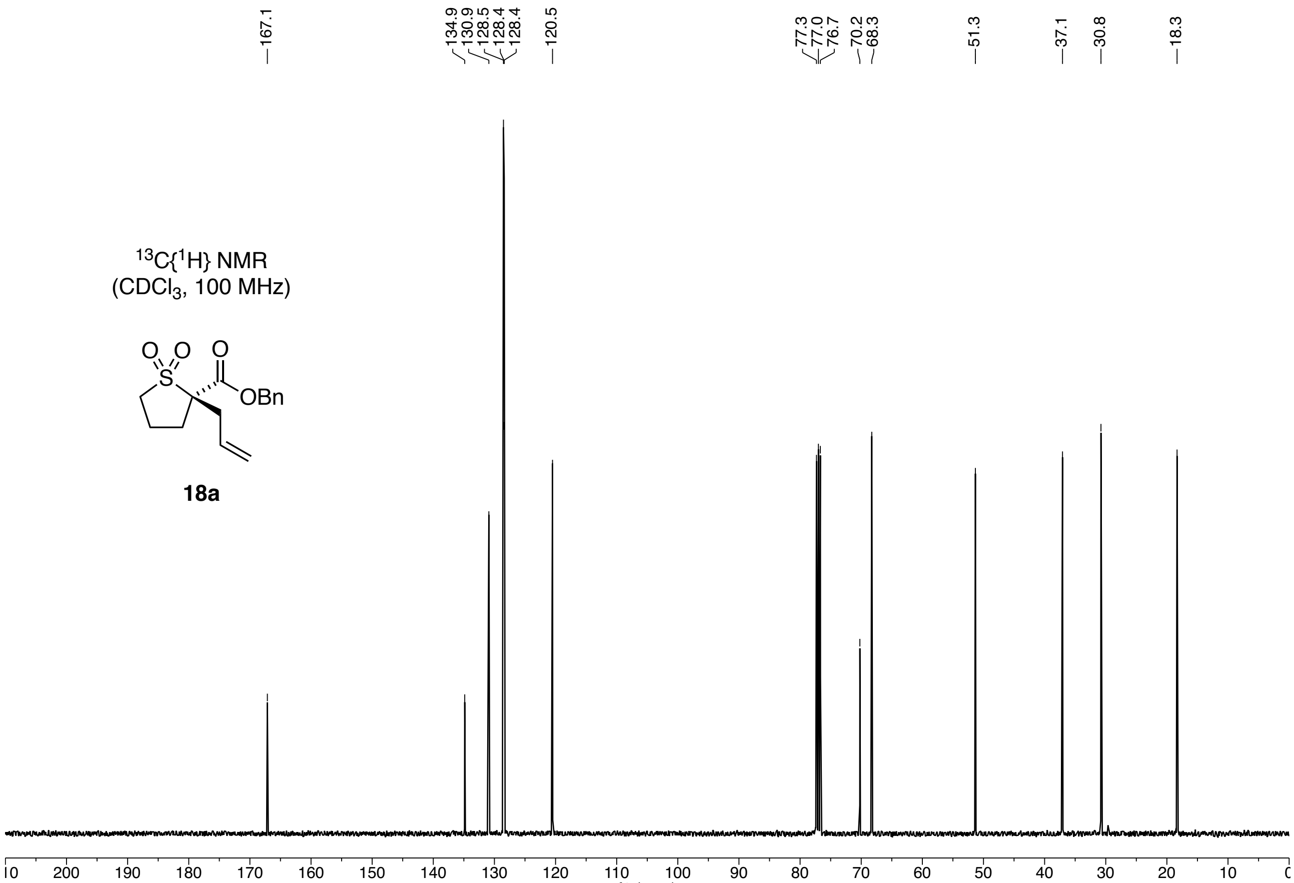

167.1
134.9 130.9 128.5 128.4 128.4
120.5
77.3 77.0 76.7
70.2
68.3
51.3
37.1
30.8
18.3
^{13}C{^{1}H} NMR
($CDCl_3$, 100 MHz)
O O O
S
OBn
18a
f1 (ppm)

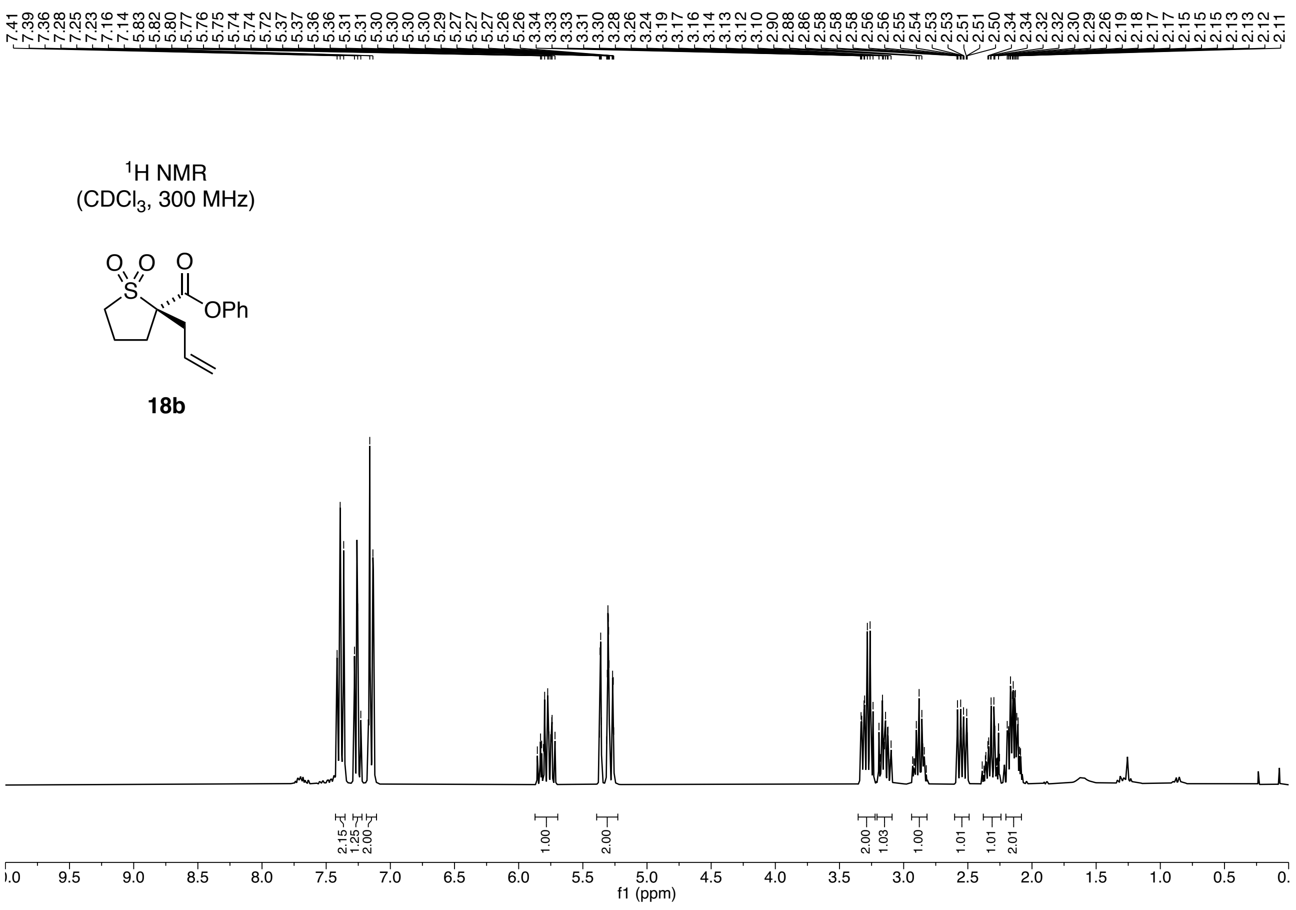
7.41 7.39 7.36 7.28 7.25 7.23 7.16 7.14 5.83 5.82 5.80 5.77 5.76 5.75 5.74 5.72 5.37 5.36 5.31 5.31 5.30 5.30 5.30 5.30 5.29 5.27 5.27 5.26 5.26 3.34 3.33 3.33 3.31 3.30 3.28 3.26 3.24 3.19 3.17 3.16 3.14 3.13 3.12 3.10 2.90 2.88 2.86 2.58 2.58 2.58 2.56 2.56 2.55 2.54 2.53 2.53 2.51 2.51 2.50 2.34 2.34 2.32 2.32 2.30 2.29 2.26 2.19 2.18 2.17 2.17 2.15 2.15 2.13 2.13 2.12 2.11
1H NMR
(CDCl3, 300 MHz)
OPh
18b
2.15 1.25 2.00 1.00 2.00 2.00 1.03 1.00 1.01 1.01 2.01
f1 (ppm)

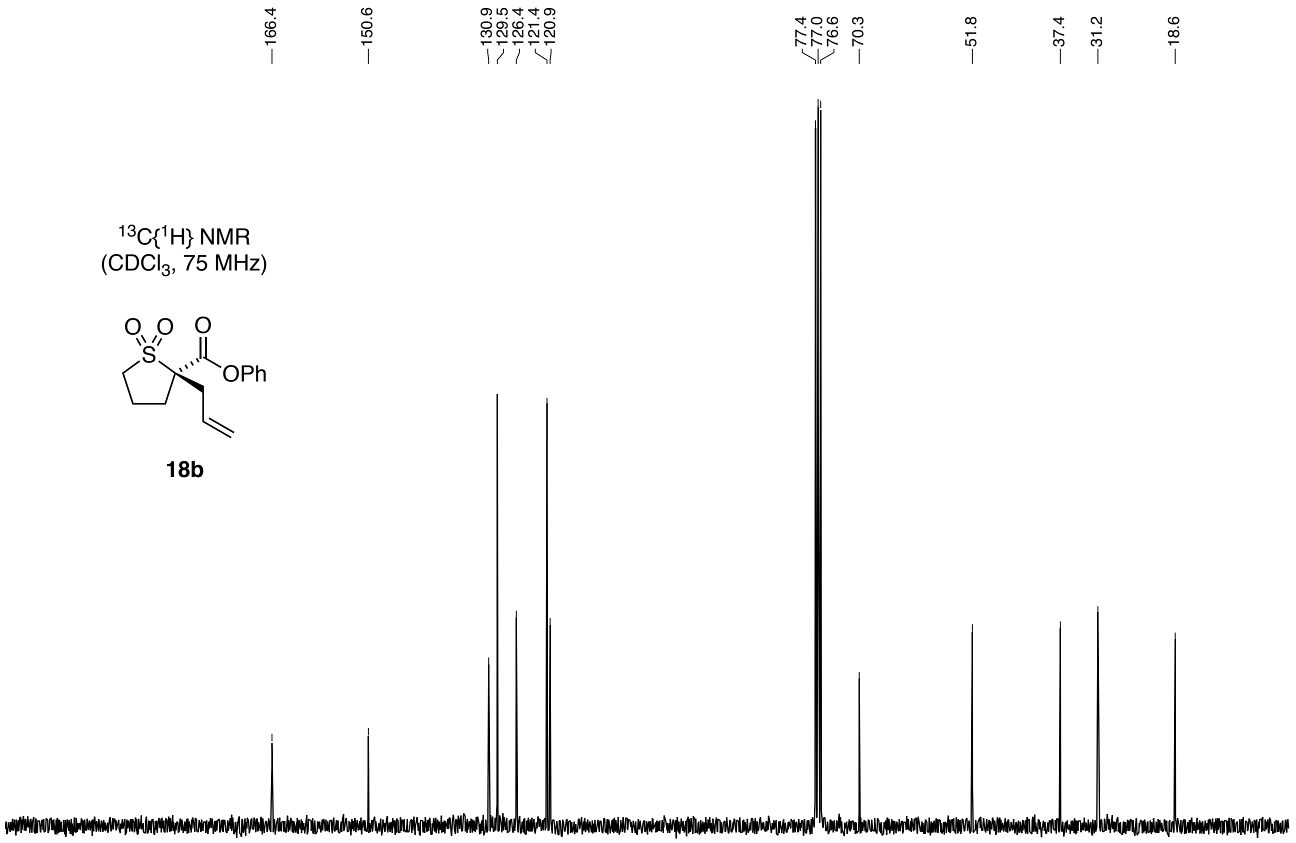
166.4 150.6 130.9 129.5 126.4 121.4 120.9 77.4 77.0 76.6 70.3 51.8 37.4 31.2 18.6
13C{1H} NMR
(CDCl3, 75 MHz)
OPh
18b
f1 (ppm)

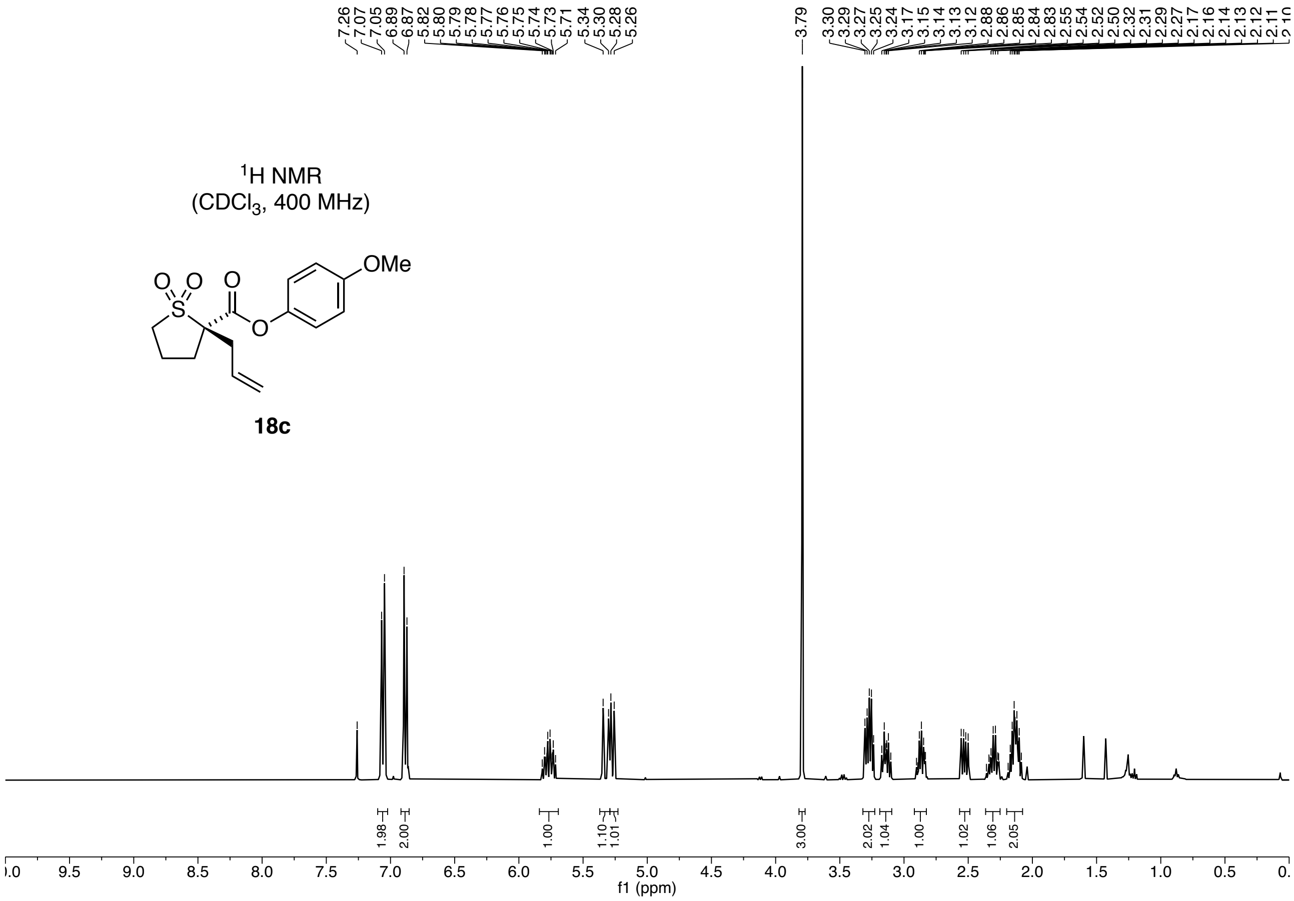
1H NMR
(CDCl3, 400 MHz)
OMe
18c
f1 (ppm)

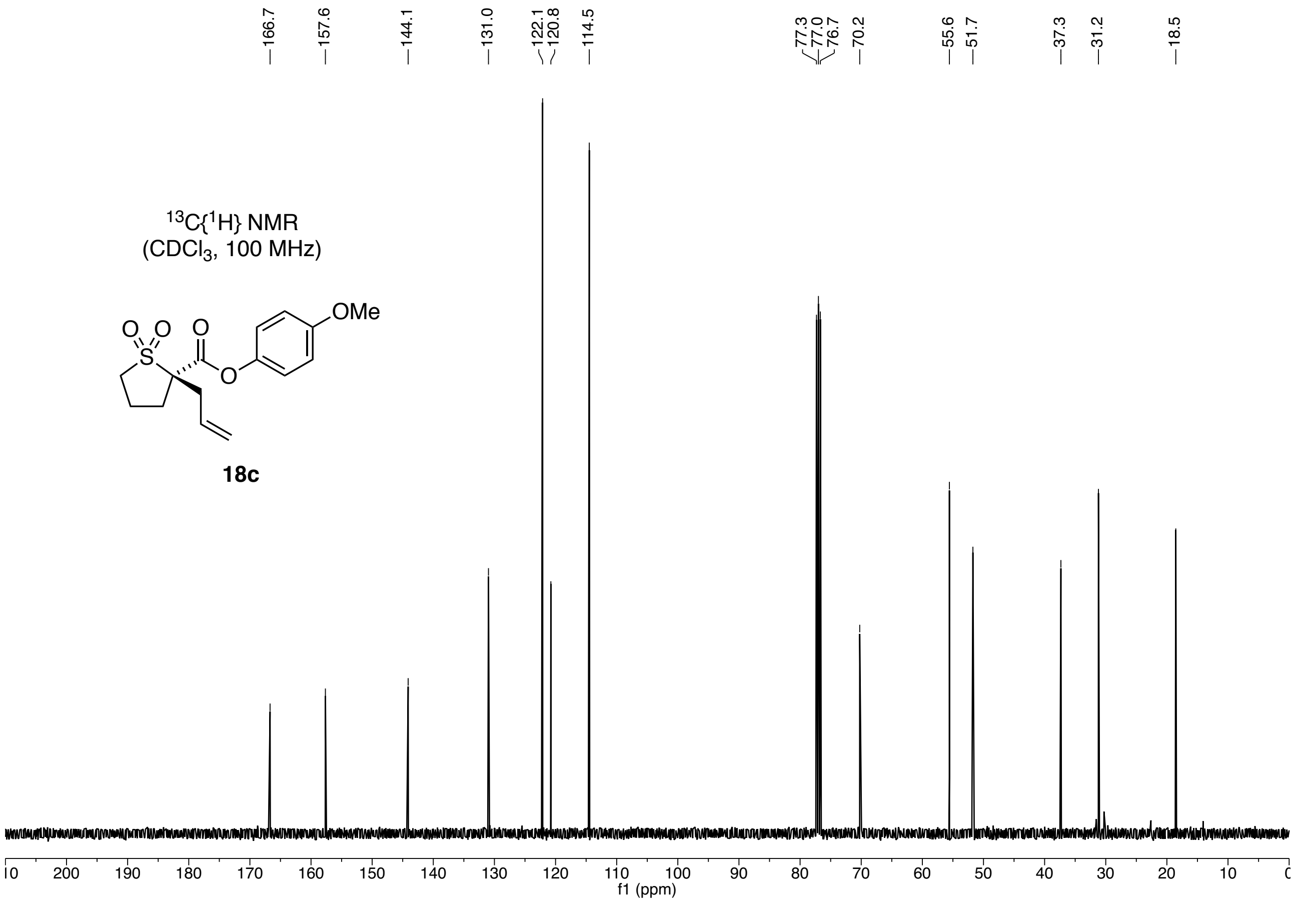
13C{1H} NMR
(CDCl3, 100 MHz)
OMe
18c
f1 (ppm)

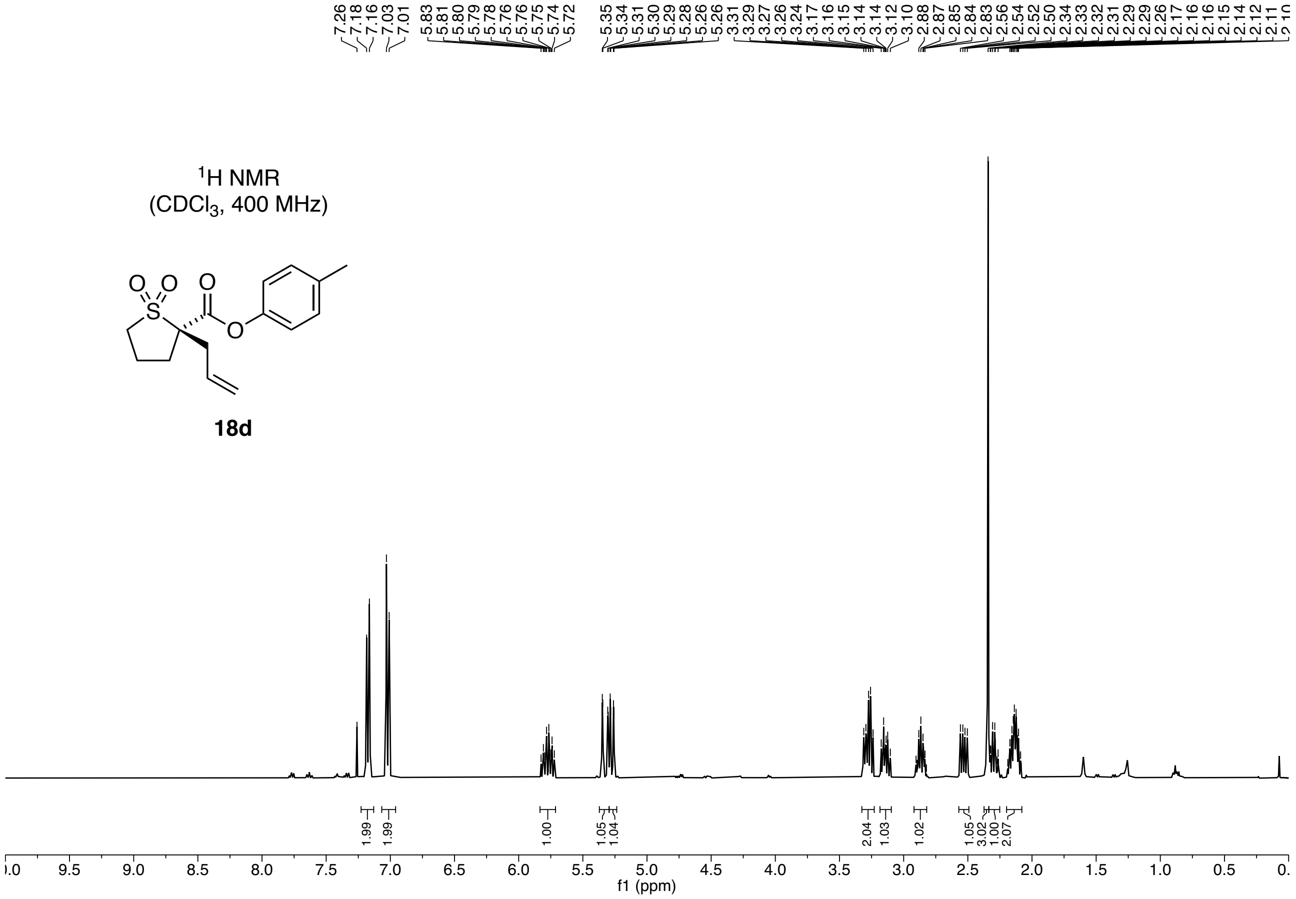

7.26 7.18 7.16 7.03 7.01 5.83 5.81 5.80 5.79 5.78 5.76 5.76 5.75 5.74 5.72 5.35 5.34 5.31 5.30 5.29 5.28 5.26 5.26 3.31 3.29 3.27 3.26 3.24 3.17 3.16 3.15 3.14 3.14 3.12 3.10 2.88 2.87 2.85 2.84 2.83 2.56 2.54 2.52 2.50 2.34 2.33 2.32 2.31 2.29 2.26 2.17 2.16 2.15 2.14 2.12 2.11
^{1}H NMR
($CDCl_3$, 400 MHz)
18d
1.99 1.99 1.00 1.05 1.04 2.04 1.03 1.02 1.05 3.02 1.00 2.07
f1 (ppm)

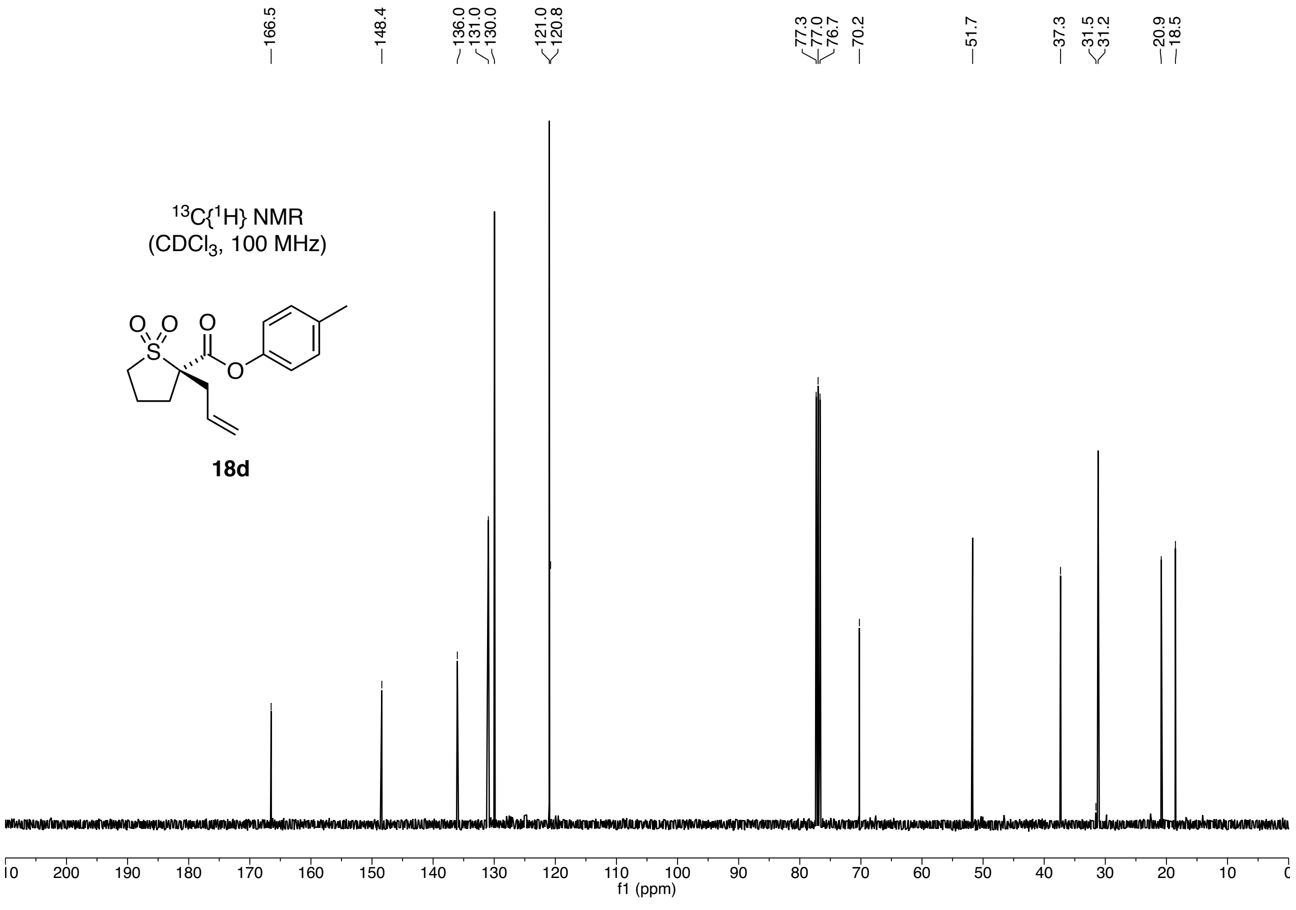

166.5 148.4 136.0 131.0 130.0 121.0 120.8 77.3 77.0 76.7 70.2 51.7 37.3 31.5 31.2 20.9 18.5
^{13}C{^{1}H} NMR
($CDCl_3$, 100 MHz)
18d
f1 (ppm)

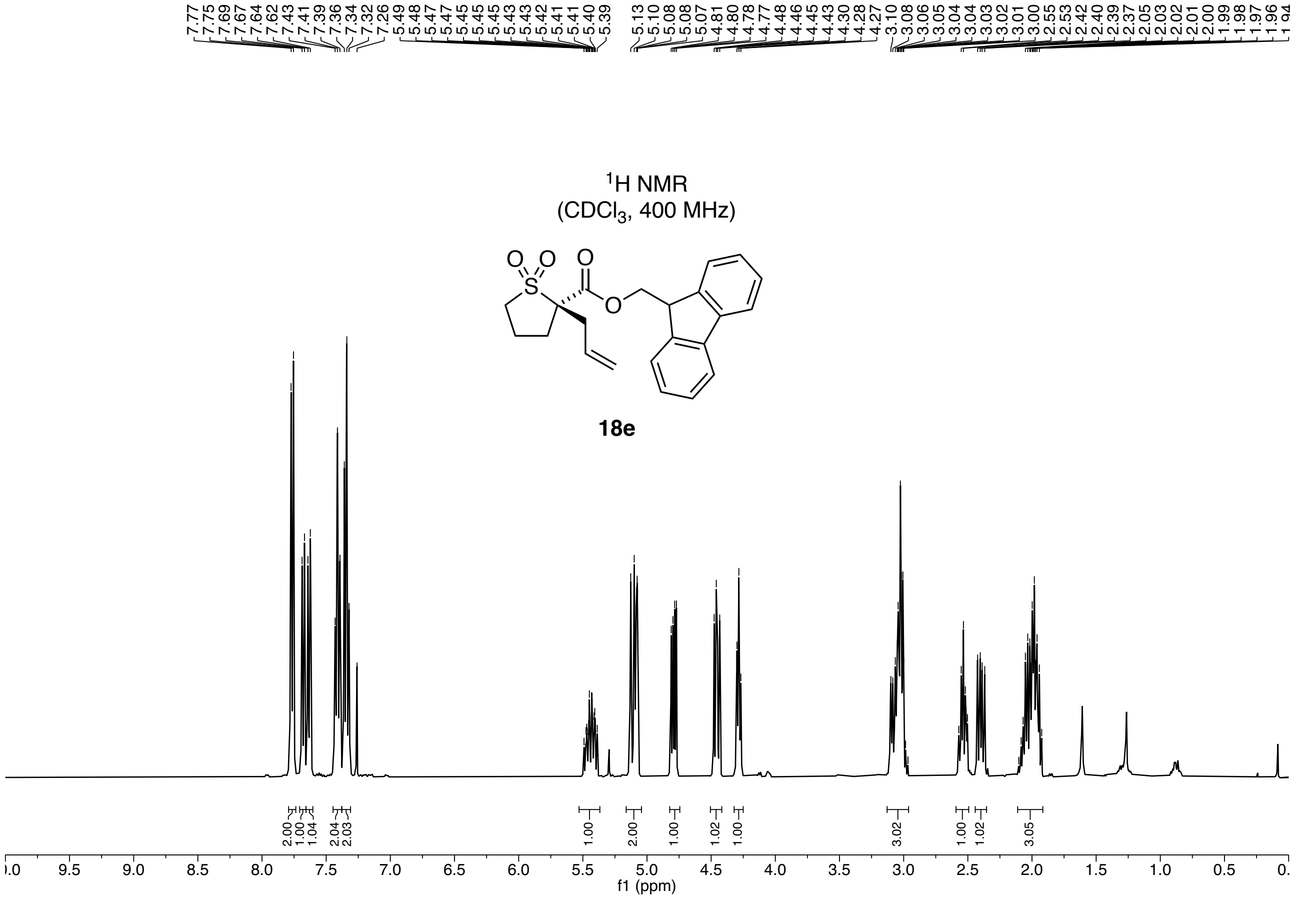

^{1}H NMR
($CDCl_3$, 400 MHz)
18e
f1 (ppm)

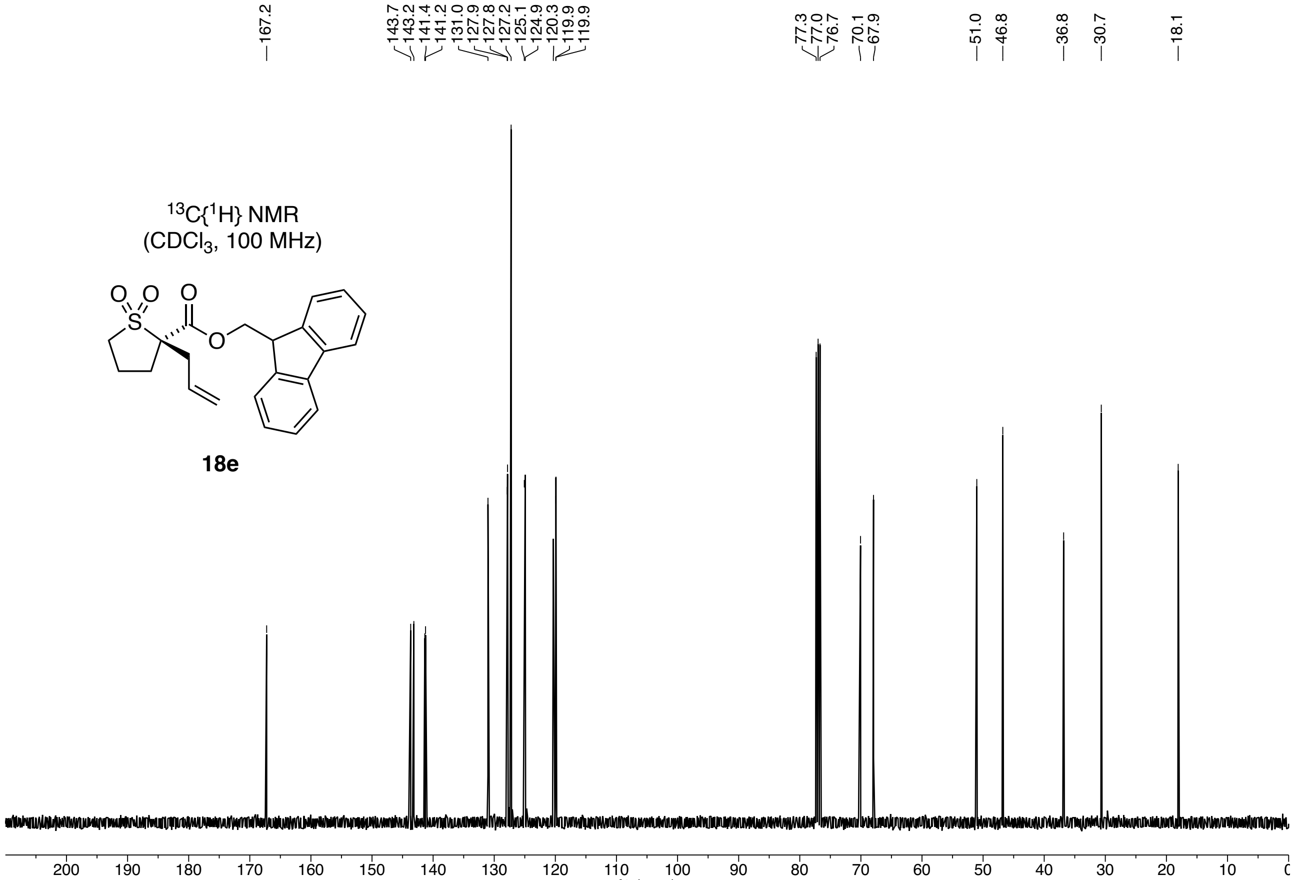

^{13}C{^{1}H} NMR
($CDCl_3$, 100 MHz)
18e
f1 (ppm)

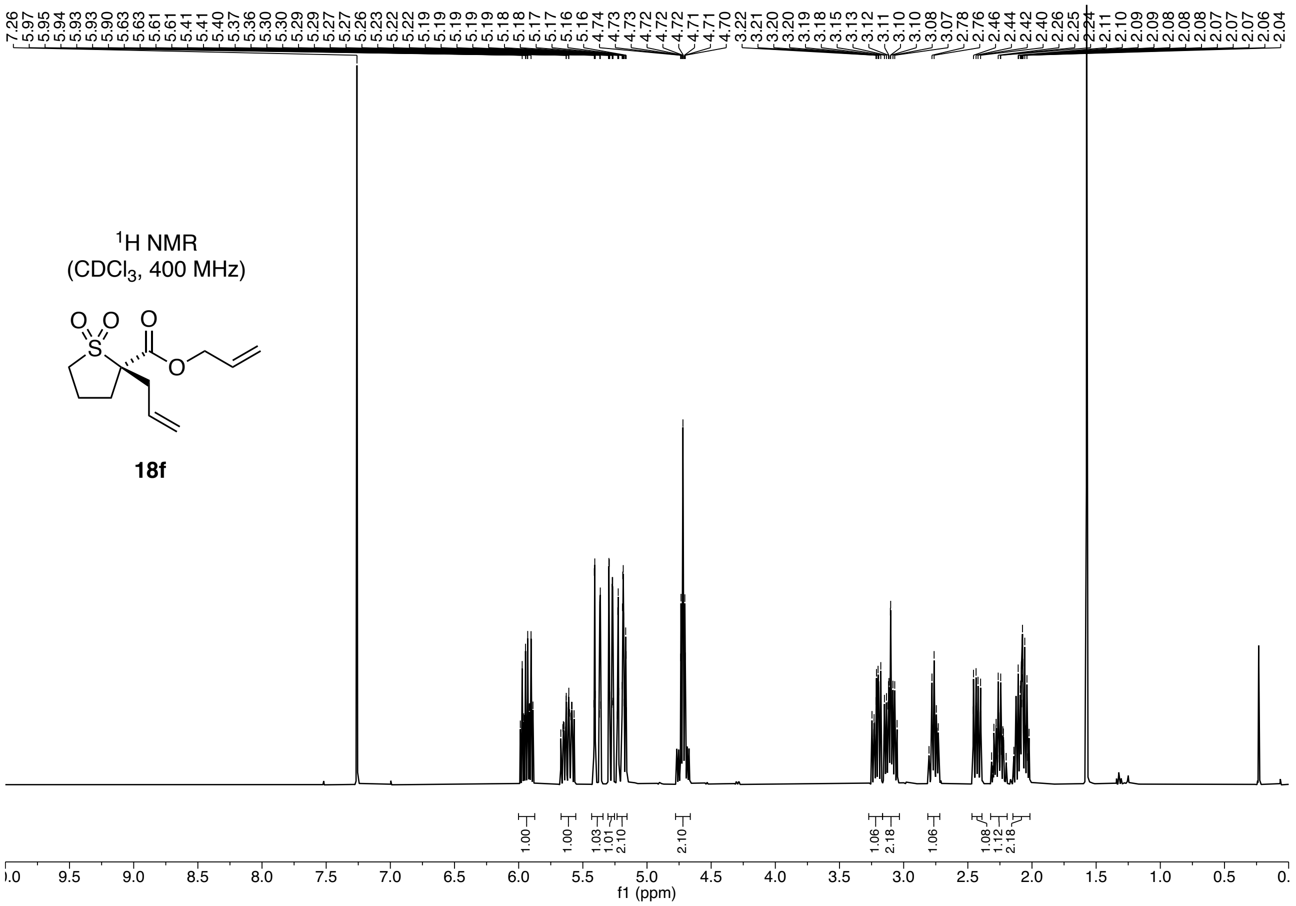

7.26
5.97
5.95
5.94
5.93
5.93
5.90
5.63
5.63
5.61
5.61
5.41
5.41
5.40
5.37
5.36
5.30
5.30
5.29
5.29
5.27
5.27
5.26
5.23
5.22
5.22
5.19
5.19
5.19
5.19
5.19
5.18
5.18
5.17
5.17
5.16
5.16
4.74
4.73
4.73
4.72
4.72
4.72
4.71
4.71
4.70
3.22
3.21
3.20
3.19
3.18
3.15
3.13
3.12
3.11
3.10
3.10
3.08
3.07
2.78
2.76
2.46
2.44
2.42
2.40
2.26
2.25
2.24
2.11
2.10
2.09
2.09
2.08
2.08
2.08
2.07
2.07
2.06
2.04
^{1}H NMR
($CDCl_3$, 400 MHz)
18f
1.00
1.00
1.03
1.01
2.10
2.10
1.06
2.18
1.06
1.08
1.12
2.18
f1 (ppm)

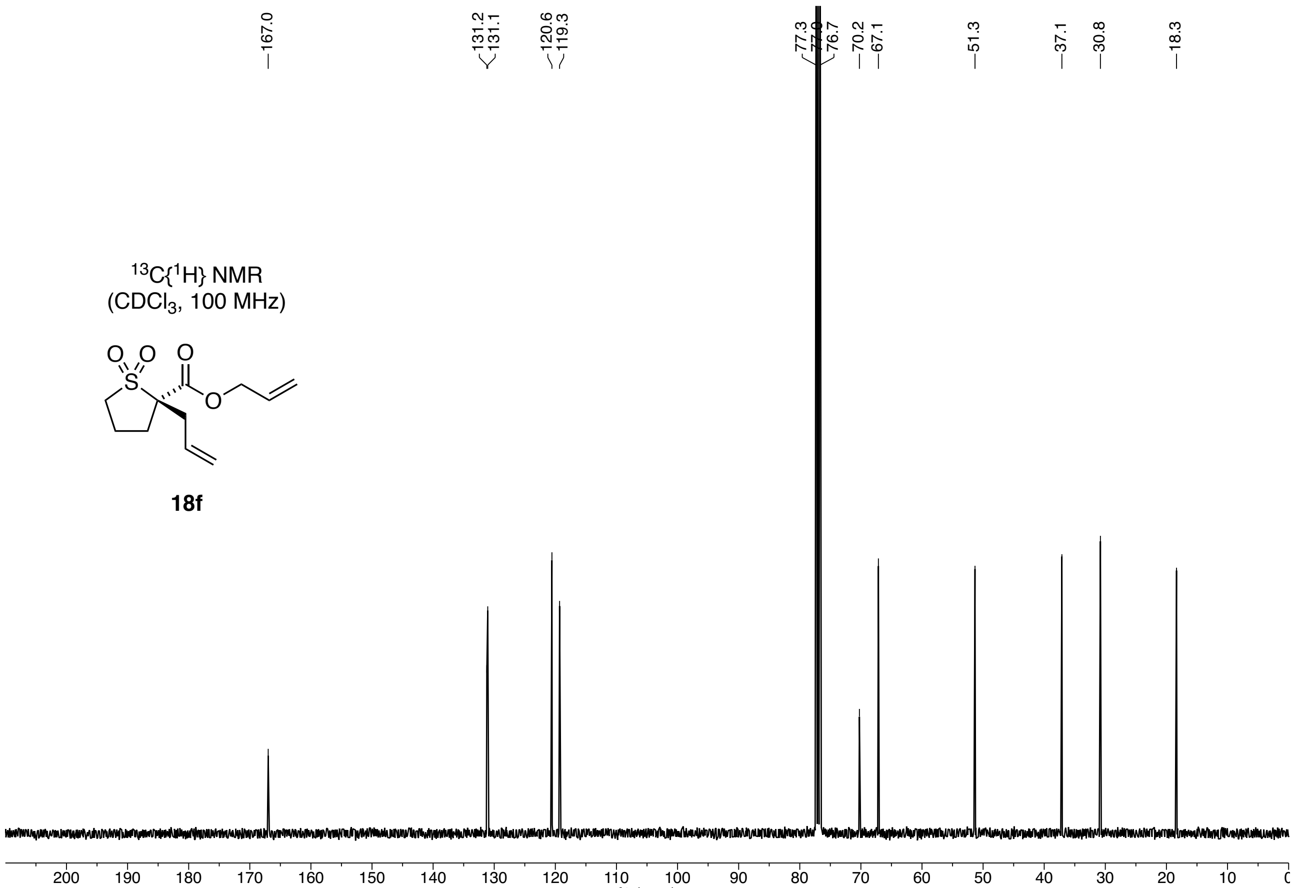

167.0
131.2
131.1
120.6
119.3
77.3
77.0
76.7
70.2
67.1
51.3
37.1
30.8
18.3
^{13}C{^{1}H} NMR
($CDCl_3$, 100 MHz)
18f
f1 (ppm)

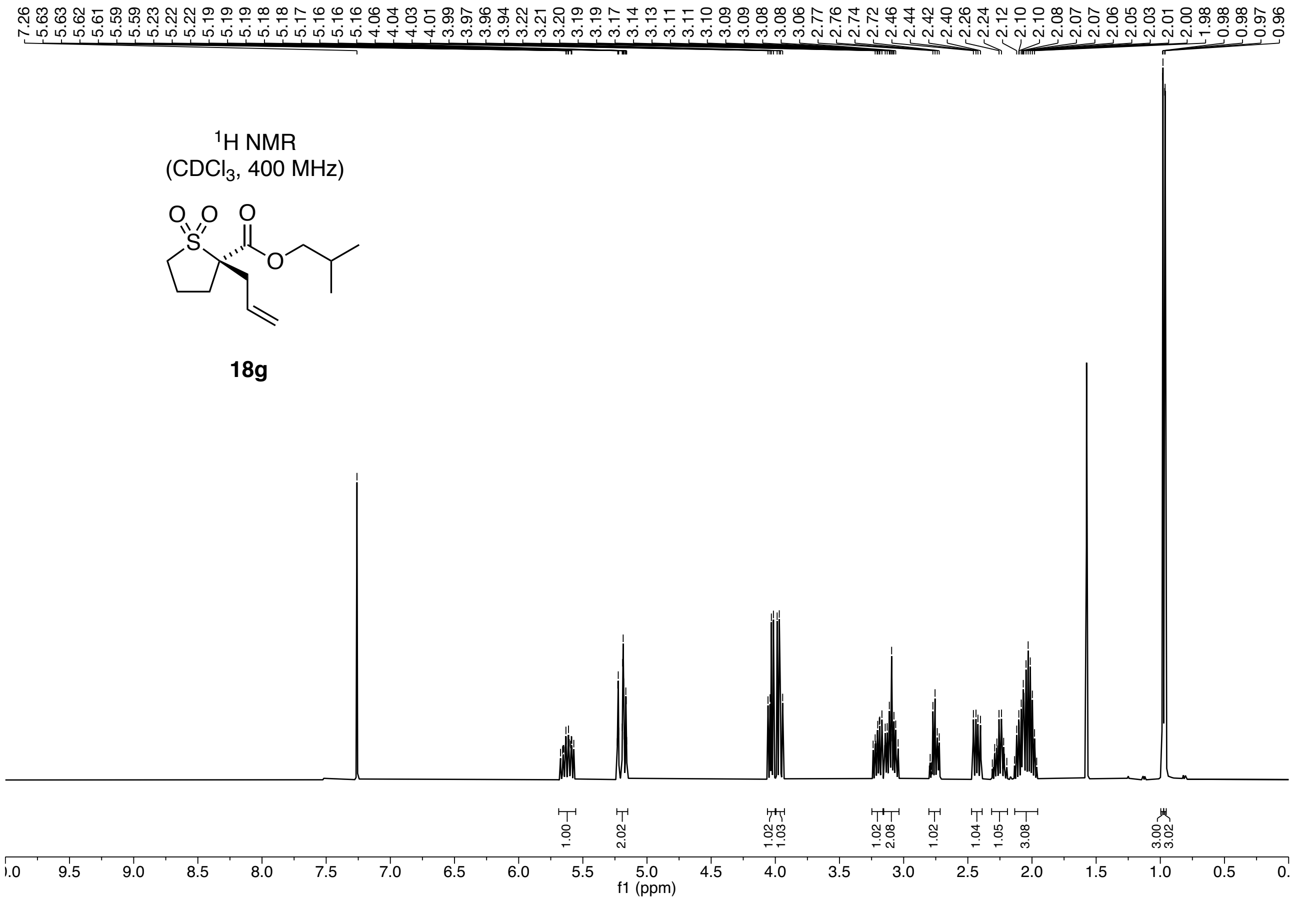
1H NMR
(CDCl3, 400 MHz)
18g
f1 (ppm)

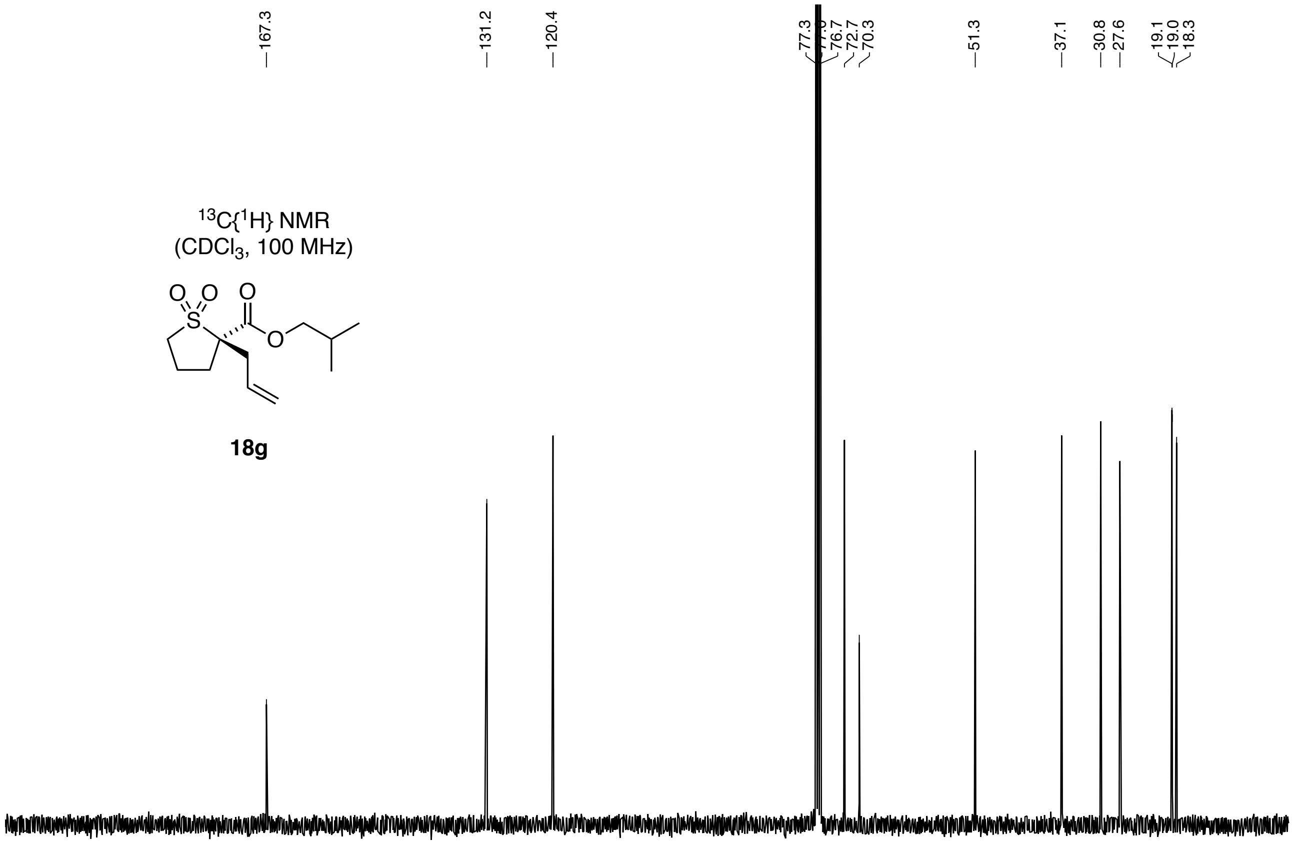
13C{1H} NMR
(CDCl3, 100 MHz)
18g
f1 (ppm)

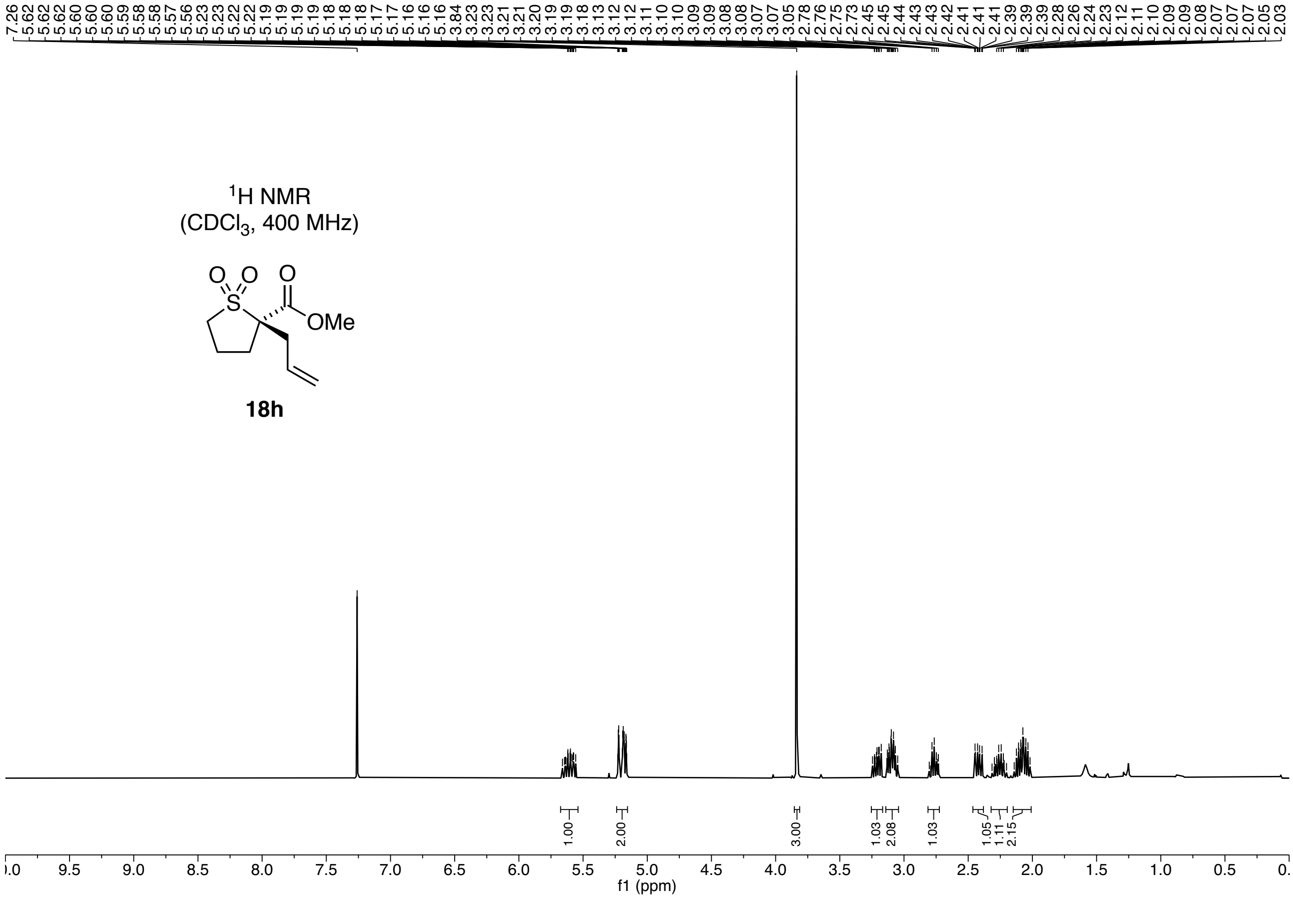
7.26 5.62 5.62 5.62 5.60 5.60 5.59 5.58 5.58 5.57 5.56 5.23 5.23 5.22 5.22 5.19 5.19 5.19 5.19 5.18 5.18 5.18 5.17 5.17 5.16 5.16 3.84 3.23 3.23 3.21 3.20 3.19 3.19 3.18 3.13 3.12 3.12 3.11 3.10 3.10 3.09 3.09 3.08 3.08 3.07 3.07 3.05 2.78 2.76 2.75 2.73 2.45 2.45 2.44 2.43 2.43 2.42 2.41 2.41 2.41 2.39 2.39 2.28 2.26 2.24 2.23 2.12 2.11 2.10 2.09 2.08 2.07 2.07 2.05 2.03
^{1}H NMR
(CDCl$_3$, 400 MHz)
OMe
18h
1.00 2.00 3.00 1.03 2.08 1.03 1.05 1.11 2.15
f1 (ppm)

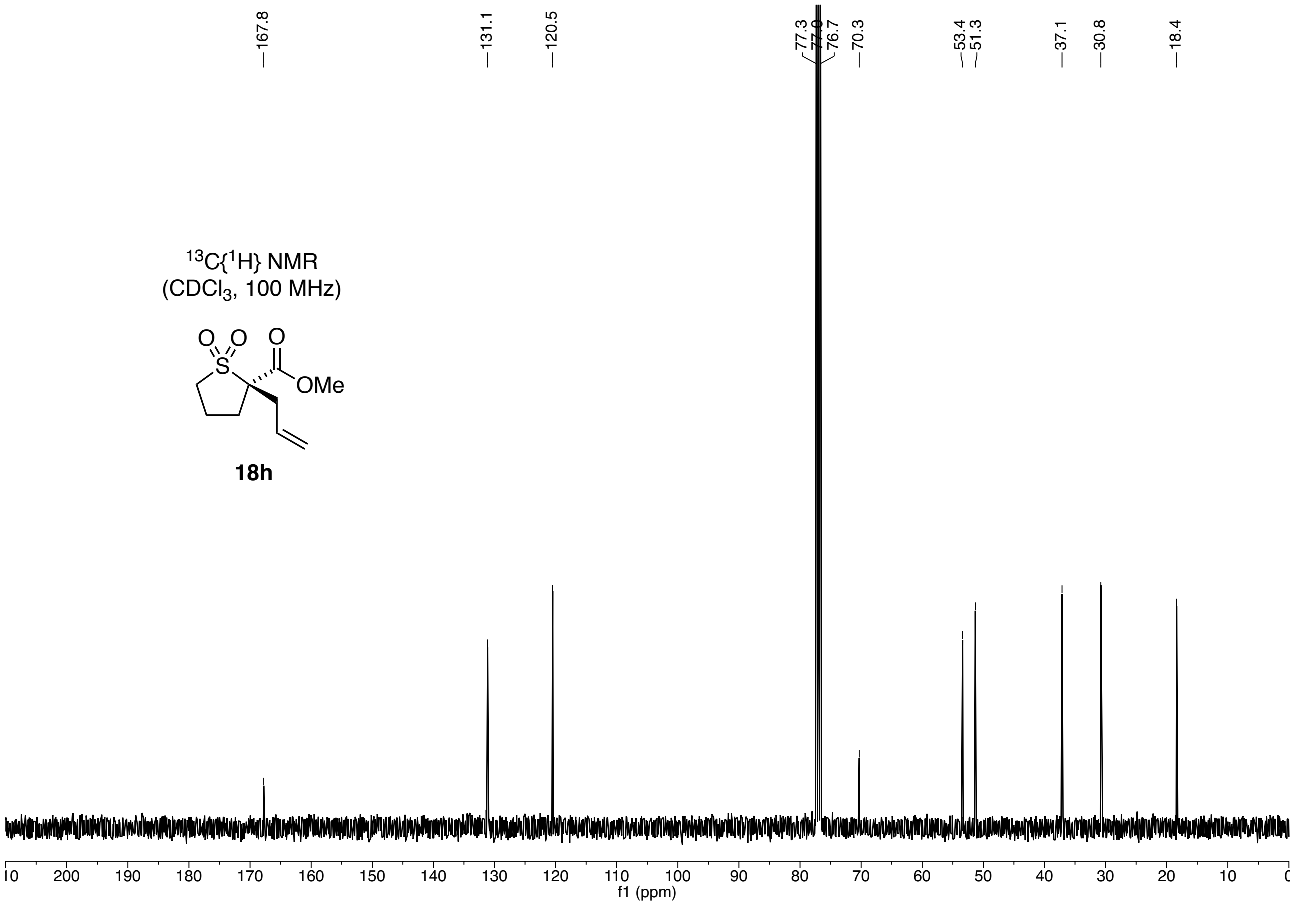
167.8 131.1 120.5 77.3 77.0 76.7 70.3 53.4 51.3 37.1 30.8 18.4
^{13}C{^{1}H} NMR
(CDCl$_3$, 100 MHz)
OMe
18h
f1 (ppm)

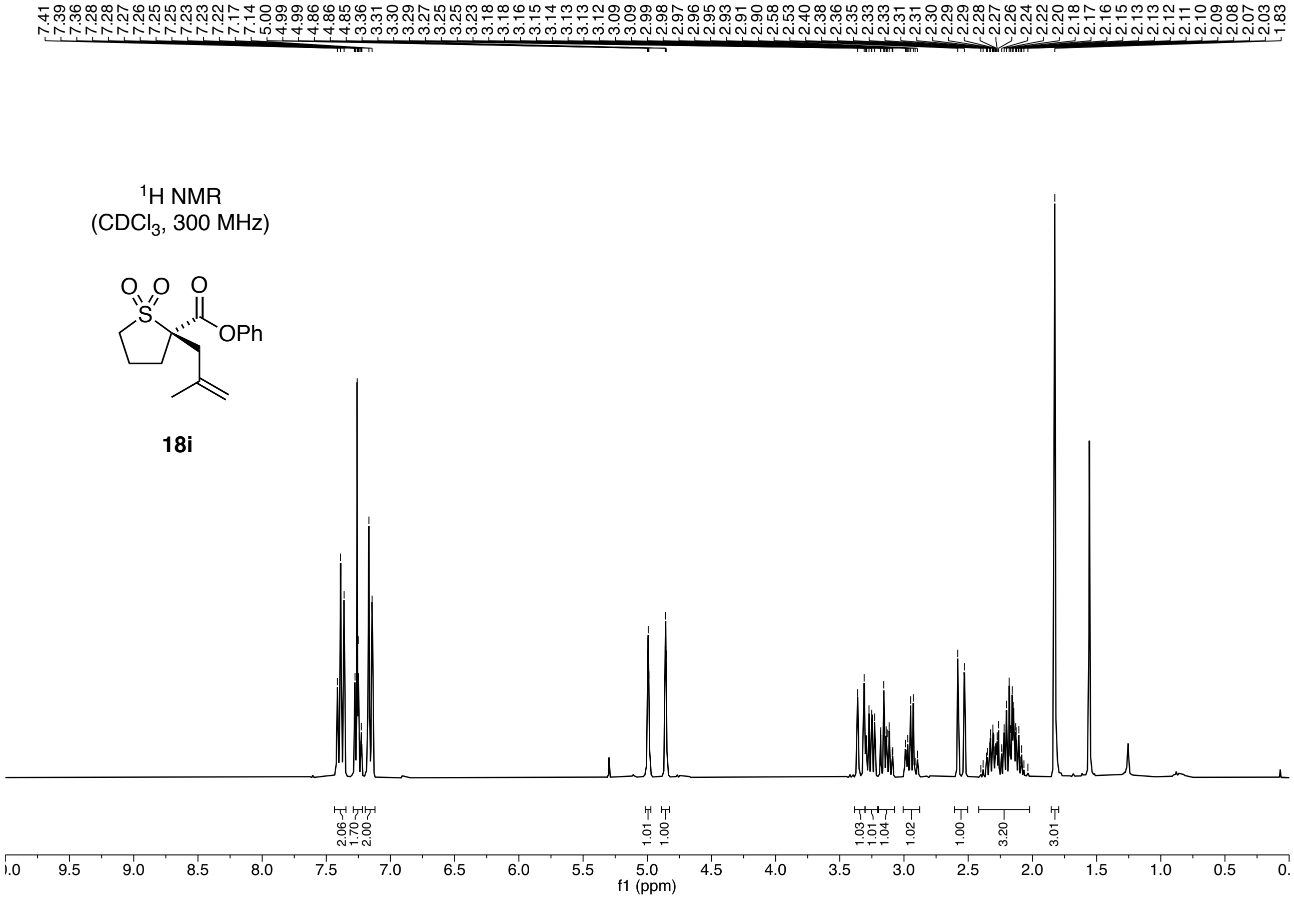
7.41 7.39 7.36 7.28 7.27 7.26 7.25 7.25 7.23 7.23 7.22 7.17 7.14 5.00 4.99 4.86 4.85 3.36 3.31 3.30 3.29 3.27 3.25 3.25 3.23 3.18 3.16 3.15 3.14 3.13 3.13 3.12 3.09 2.99 2.98 2.97 2.96 2.95 2.93 2.91 2.90 2.58 2.53 2.40 2.38 2.36 2.35 2.33 2.33 2.31 2.30 2.29 2.28 2.27 2.26 2.24 2.22 2.20 2.18 2.17 2.16 2.15 2.13 2.13 2.12 2.11 2.10 2.09 2.08 2.07 2.03 1.83
^{1}H NMR
($CDCl_3$, 300 MHz)
OPh
18i
2.06 1.70 2.00 1.01 1.00 1.03 1.01 1.04 1.02 1.00 3.20 3.01
f1 (ppm)

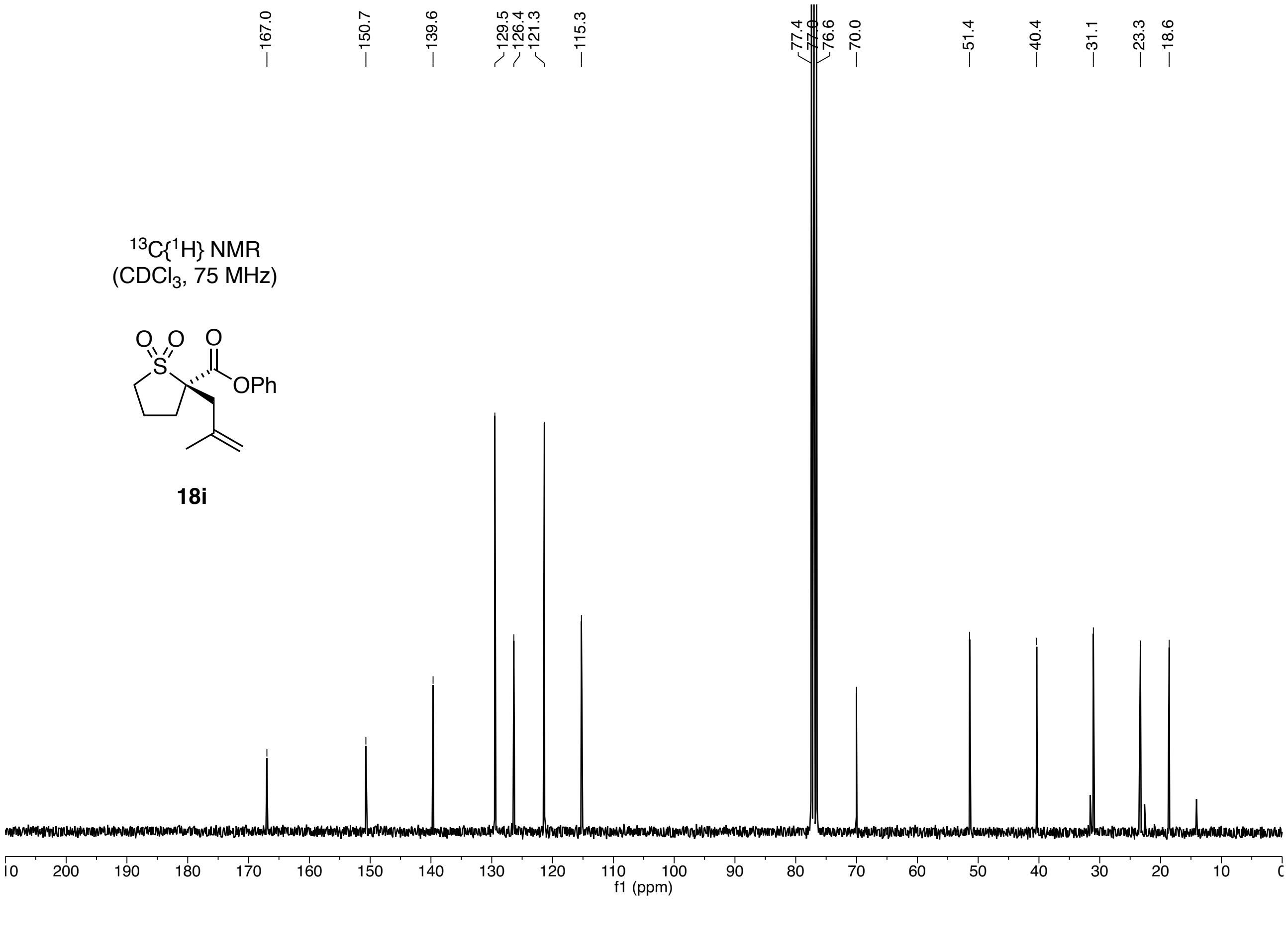
167.0 150.7 139.6 129.5 126.4 121.3 115.3 77.4 77.0 76.6 70.0 51.4 40.4 31.1 23.3 18.6
$^{13}C\{^1H\}$ NMR
($CDCl_3$, 75 MHz)
OPh
18i
f1 (ppm)

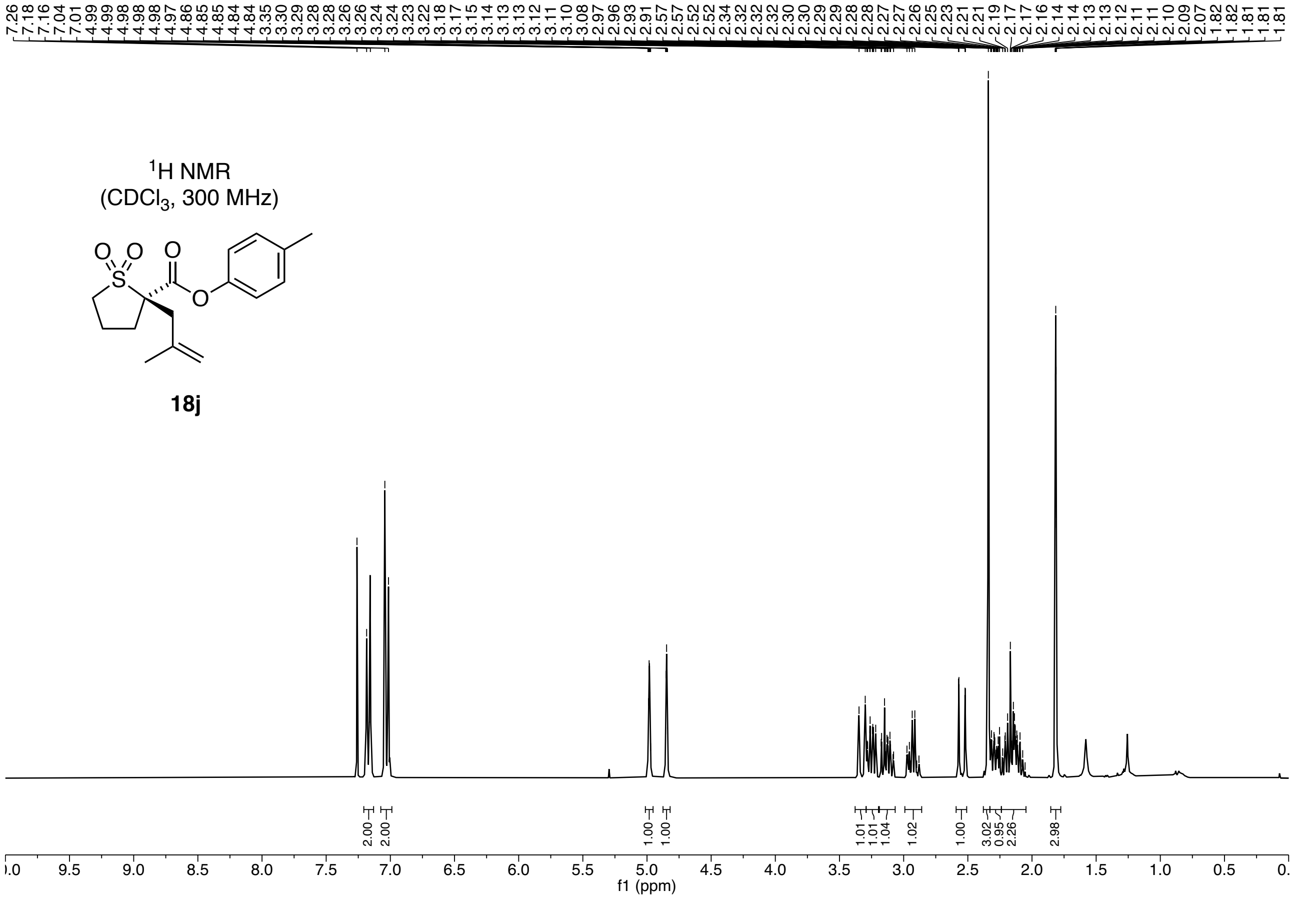
7.26 7.18 7.16 7.04 7.01 4.99 4.99 4.98 4.98 4.97 4.86 4.85 4.85 4.84 4.84 3.35 3.30 3.29 3.28 3.28 3.26 3.26 3.24 3.24 3.23 3.22 3.18 3.17 3.15 3.14 3.13 3.13 3.12 3.11 3.10 3.08 2.97 2.96 2.93 2.91 2.57 2.57 2.52 2.52 2.34 2.32 2.32 2.32 2.30 2.29 2.29 2.28 2.28 2.27 2.27 2.26 2.25 2.23 2.21 2.21 2.19 2.17 2.17 2.16 2.14 2.14 2.13 2.13 2.12 2.11 2.11 2.10 2.09 2.07 1.82 1.82 1.81 1.81 1.81
^{1}H NMR
(CDCl$_3$, 300 MHz)
18j
2.00 2.00 1.00 1.00 1.01 1.01 1.04 1.02 1.00 3.02 0.95 2.26 2.98
f1 (ppm)

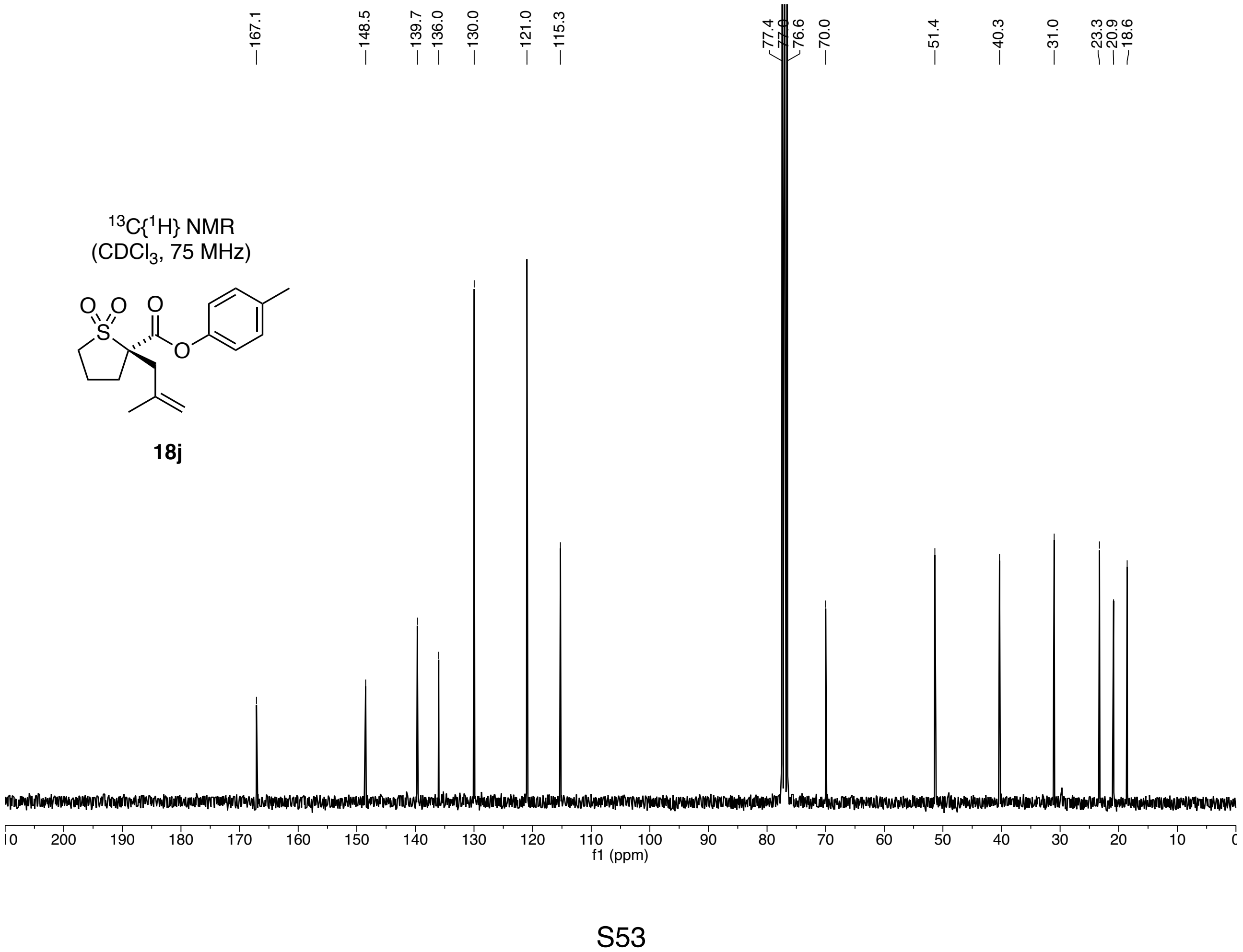
167.1 148.5 139.7 136.0 130.0 121.0 115.3 77.4 77.0 76.6 70.0 51.4 40.3 31.0 23.3 20.9 18.6
^{13}C{^{1}H} NMR
(CDCl$_3$, 75 MHz)
18j
f1 (ppm)

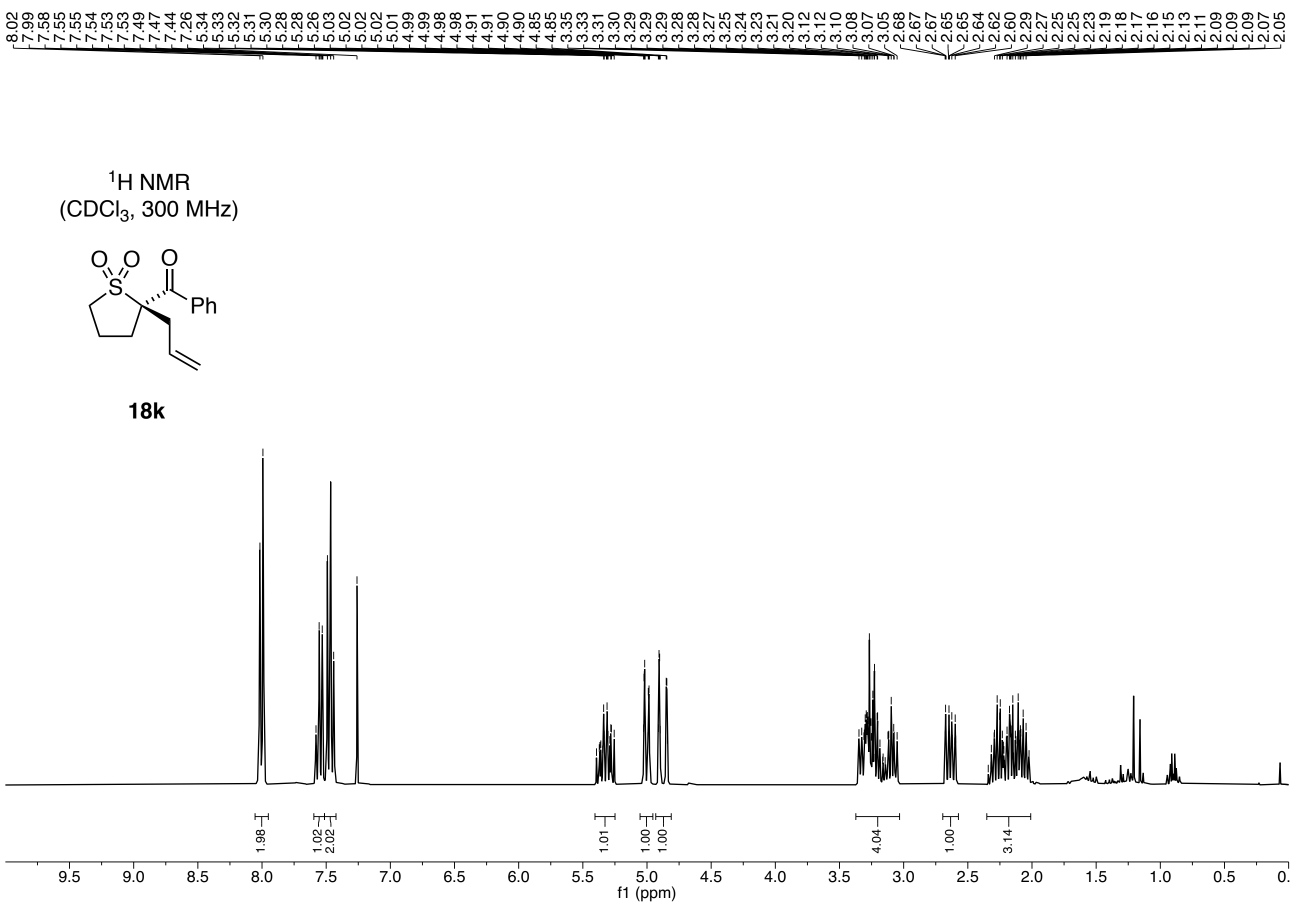
8.02 7.99 7.58 7.55 7.54 7.53 7.49 7.47 7.44 7.26 5.34 5.33 5.32 5.31 5.30 5.28 5.26 5.03 5.02 5.02 5.02 5.01 4.99 4.99 4.98 4.98 4.91 4.91 4.90 4.90 4.85 3.35 3.33 3.31 3.30 3.29 3.29 3.28 3.28 3.27 3.25 3.24 3.23 3.21 3.20 3.12 3.10 3.08 3.07 3.05 2.68 2.67 2.67 2.65 2.65 2.64 2.62 2.60 2.29 2.27 2.25 2.23 2.19 2.18 2.17 2.16 2.15 2.13 2.11 2.09 2.09 2.07 2.05
^{1}H NMR
($CDCl_3$, 300 MHz)
O O O
S
Ph
18k
1.98 1.02 2.02 1.01 1.00 1.00 4.04 1.00 3.14
9.5 9.0 8.5 8.0 7.5 7.0 6.5 6.0 5.5 5.0 4.5 4.0 3.5 3.0 2.5 2.0 1.5 1.0 0.5 0.
f1 (ppm)

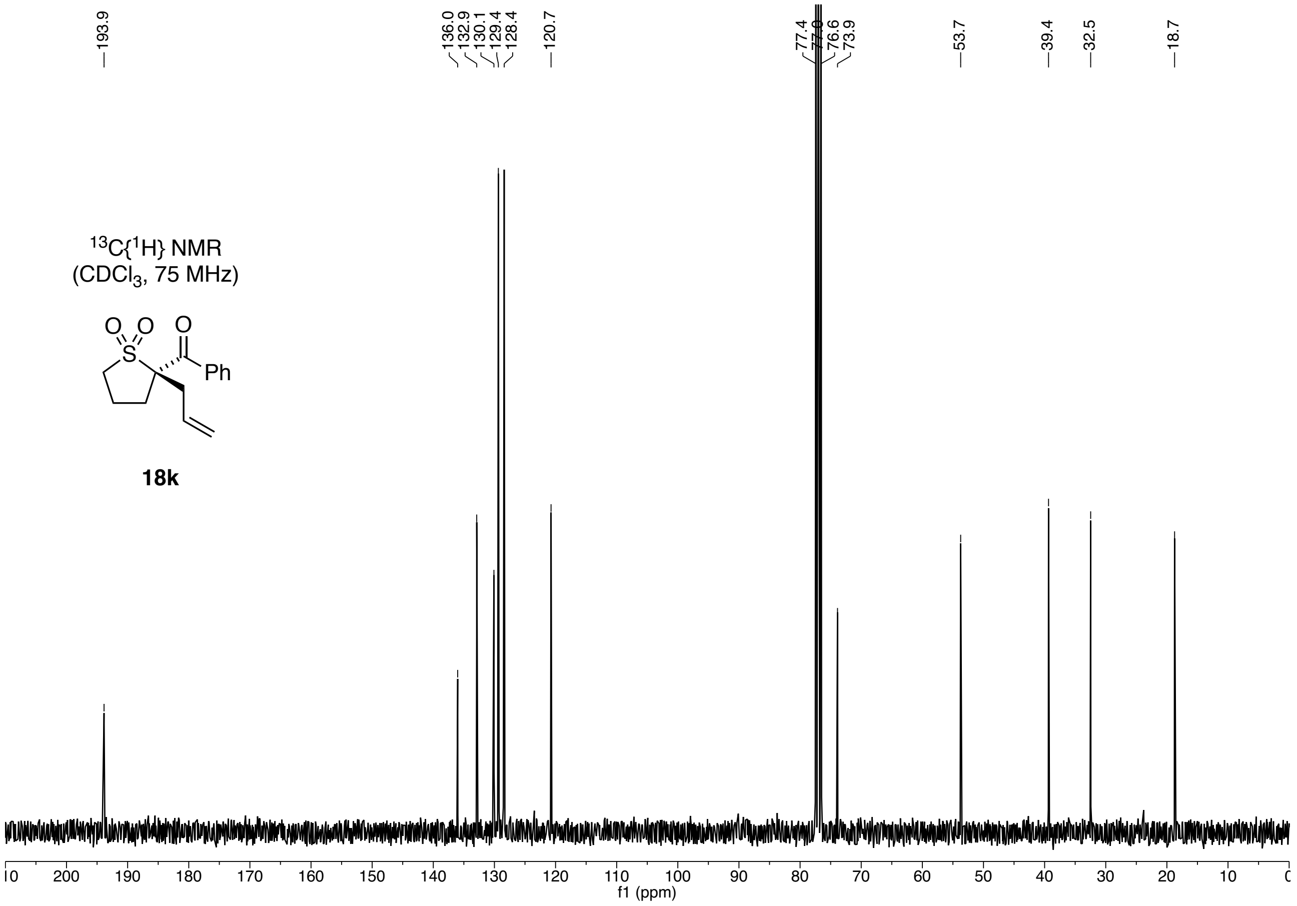
193.9
136.0 132.9 130.1 129.4 128.4 120.7
77.4 77.0 76.6 73.9
53.7
39.4 32.5
18.7
$^{13}C\{^1H\}$ NMR
($CDCl_3$, 75 MHz)
O O O
S
Ph
18k
10 200 190 180 170 160 150 140 130 120 110 100 90 80 70 60 50 40 30 20 10 0
f1 (ppm)

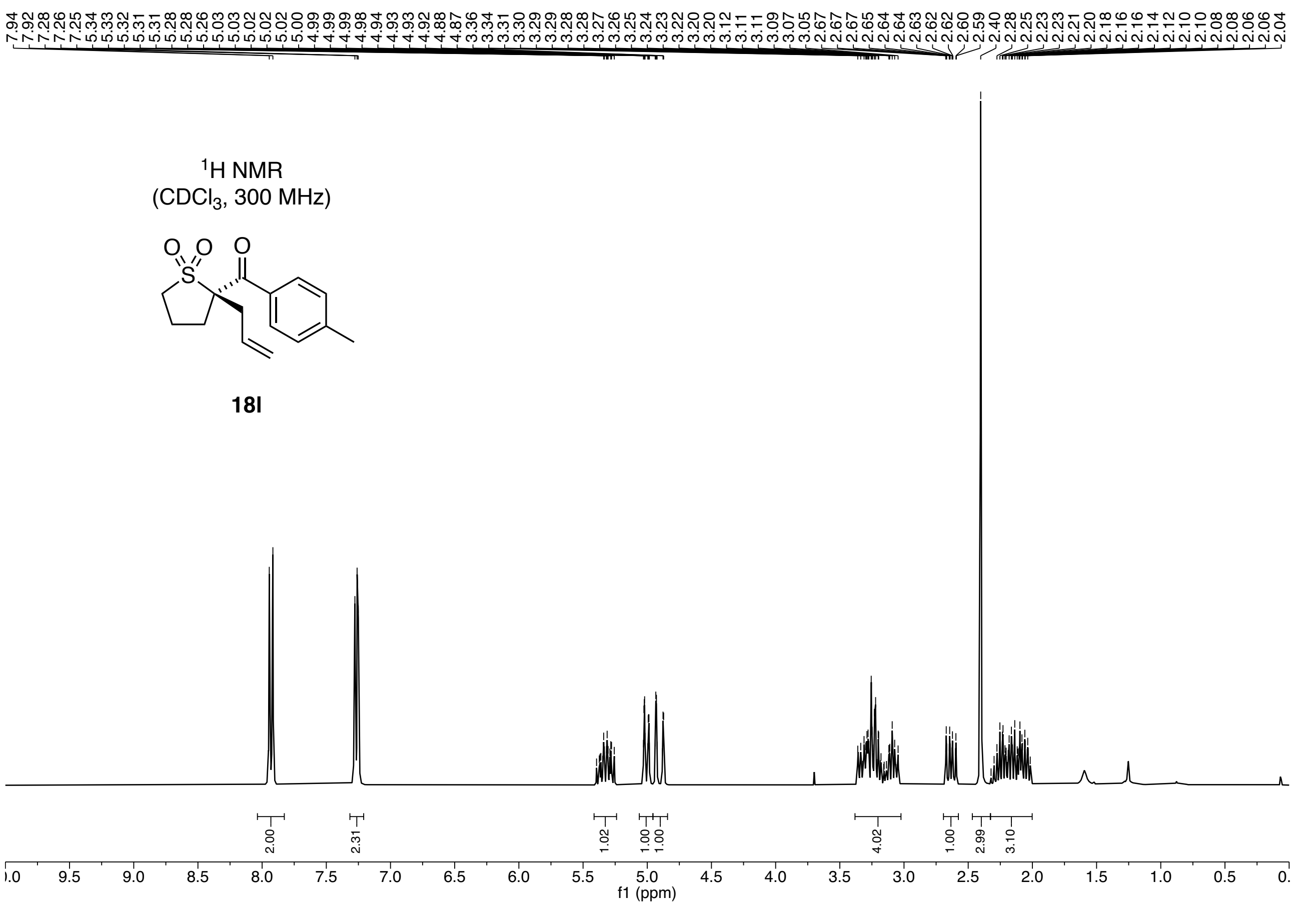
1H NMR
(CDCl3, 300 MHz)
18l
f1 (ppm)

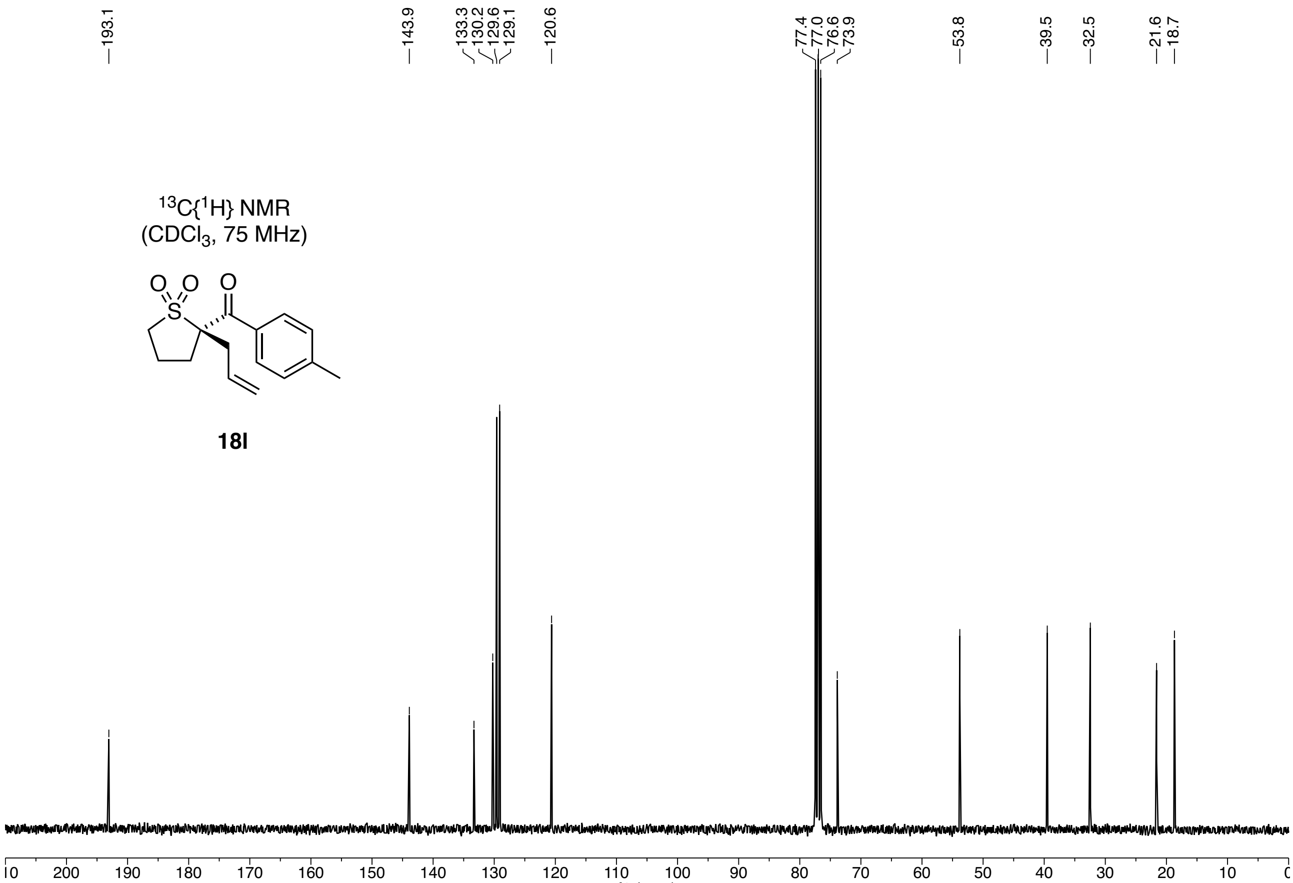
193.1
143.9
133.3
130.2
129.6
129.1
120.6
77.4
77.0
76.6
73.9
53.8
39.5
32.5
21.6
18.7
13C{1H} NMR
(CDCl3, 75 MHz)
18l
f1 (ppm)

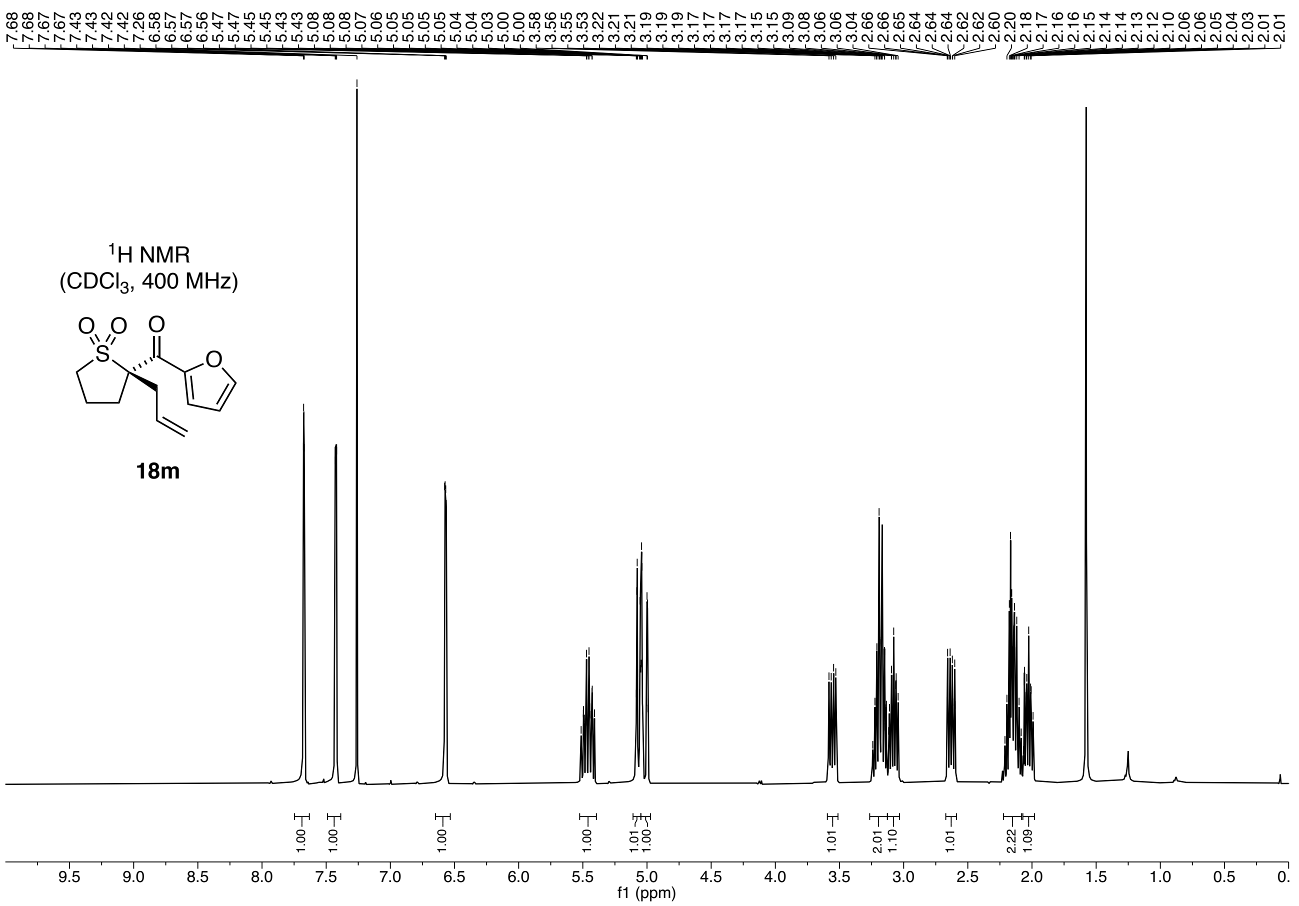

^{1}H NMR
($CDCl_3$, 400 MHz)
18m
f1 (ppm)

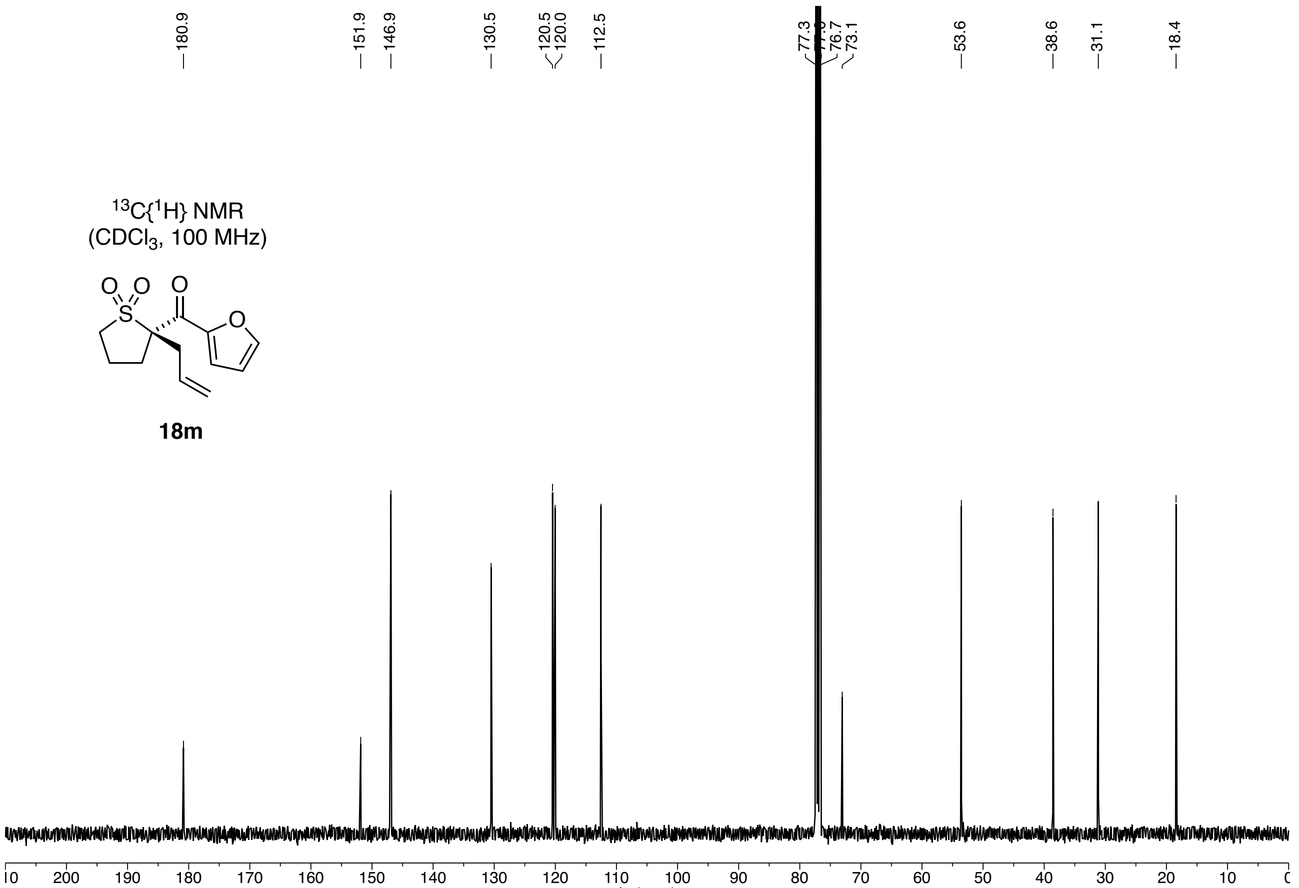

$^{13}C\{^1H\}$ NMR
($CDCl_3$, 100 MHz)
18m
180.9
151.9
146.9
130.5
120.5
120.0
112.5
77.3
76.7
73.1
53.6
38.6
31.1
18.4
f1 (ppm)

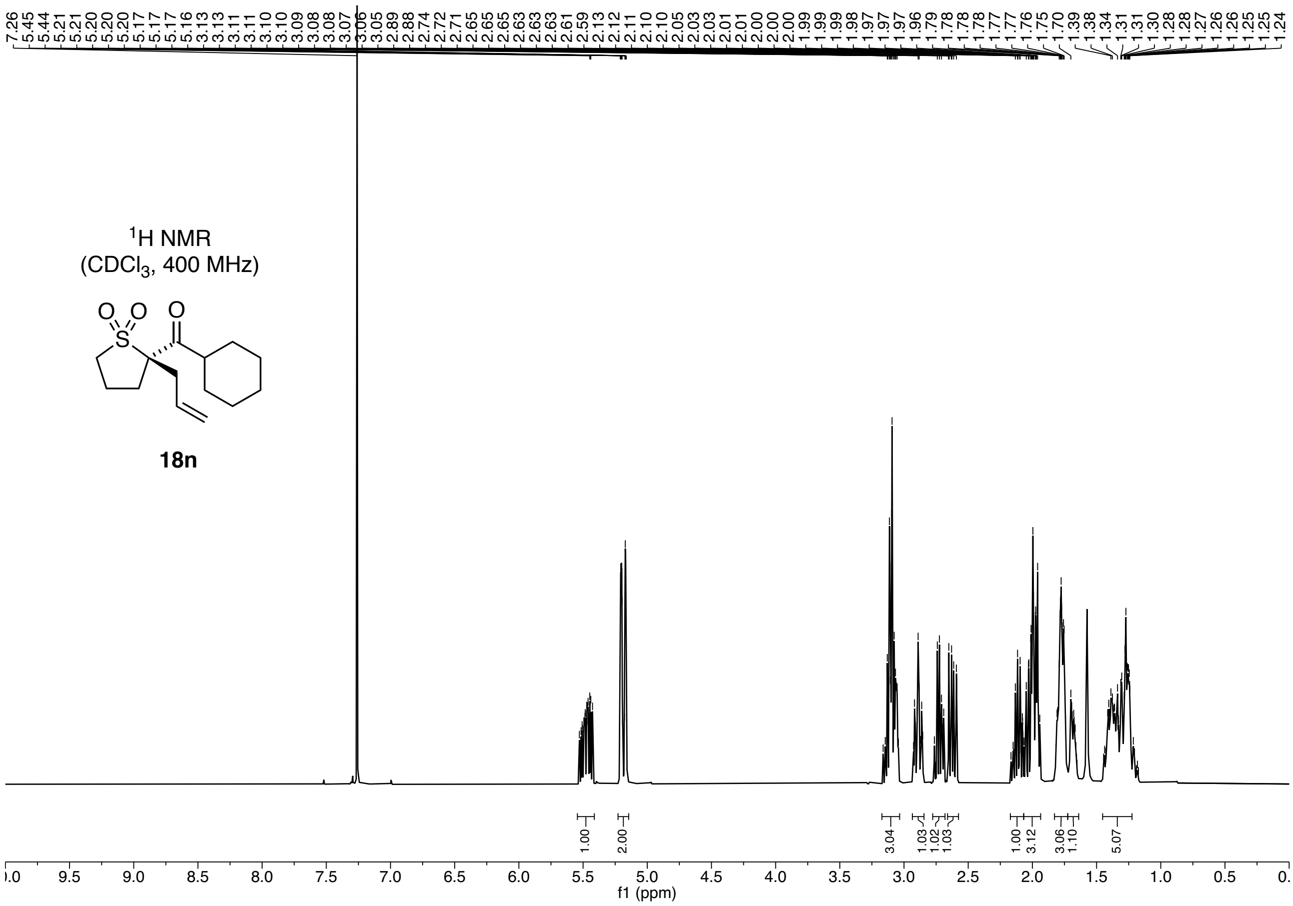

^{1}H NMR
(CDCl$_3$, 400 MHz)
18n
1.00
2.00
3.04
1.03
1.02
1.03
1.00
3.12
3.06
1.10
5.07
f1 (ppm)

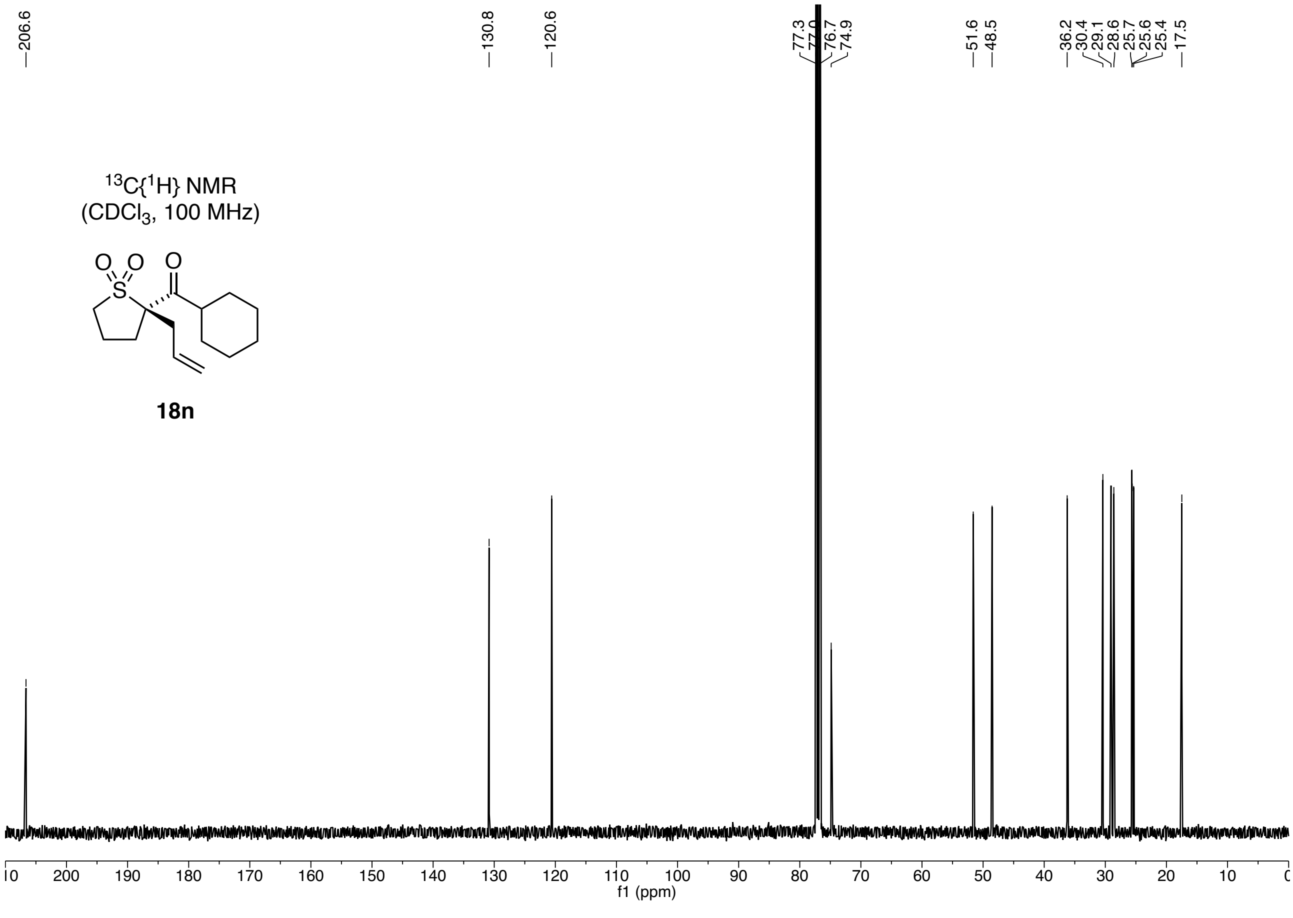

206.6
130.8
120.6
77.3
77.0
76.7
74.9
51.6
48.5
36.2
30.4
29.1
28.6
25.7
25.6
25.4
17.5
^{13}C{^{1}H} NMR
(CDCl$_3$, 100 MHz)
18n
f1 (ppm)

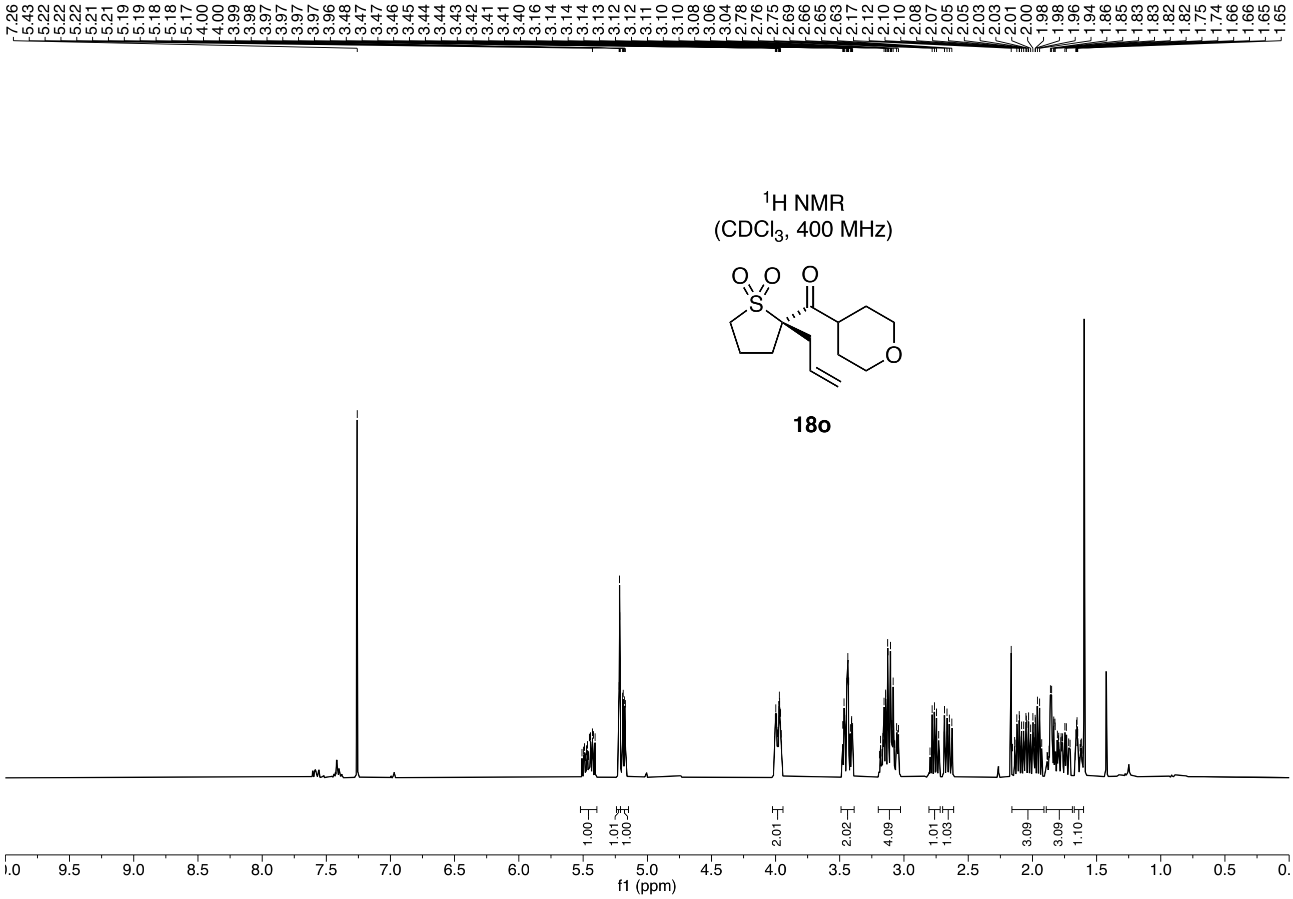
7.26
5.43
5.22
5.22
5.22
5.21
5.19
5.18
5.18
5.17
4.00
3.99
3.98
3.97
3.97
3.97
3.96
3.48
3.47
3.46
3.45
3.44
3.44
3.43
3.42
3.41
3.41
3.40
3.16
3.14
3.14
3.13
3.12
3.12
3.11
3.10
3.10
3.08
3.06
3.04
2.78
2.76
2.75
2.69
2.66
2.65
2.63
2.17
2.12
2.10
2.10
2.08
2.07
2.05
2.05
2.03
2.03
2.01
2.00
1.98
1.98
1.96
1.94
1.86
1.85
1.83
1.82
1.82
1.75
1.74
1.66
1.66
1.65
1.65
^{1}H NMR
(CDCl$_3$, 400 MHz)
18o
1.00
1.01
1.00
2.01
2.02
4.09
1.01
1.03
3.09
3.09
1.10
f1 (ppm)

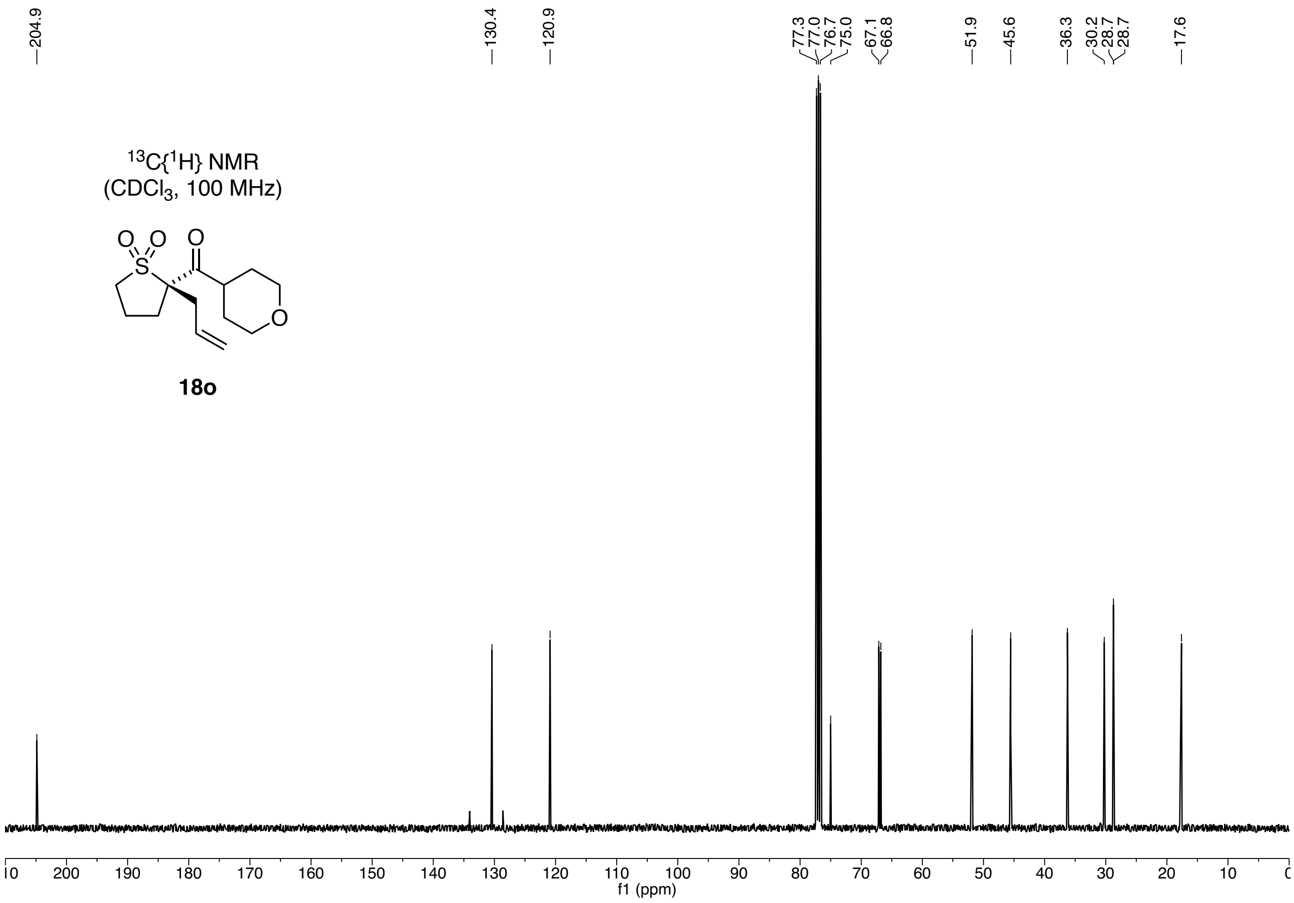
204.9
130.4
120.9
77.3
77.0
76.7
75.0
67.1
66.8
51.9
45.6
36.3
30.2
28.7
28.7
17.6
^{13}C{^{1}H} NMR
(CDCl$_3$, 100 MHz)
18o
f1 (ppm)

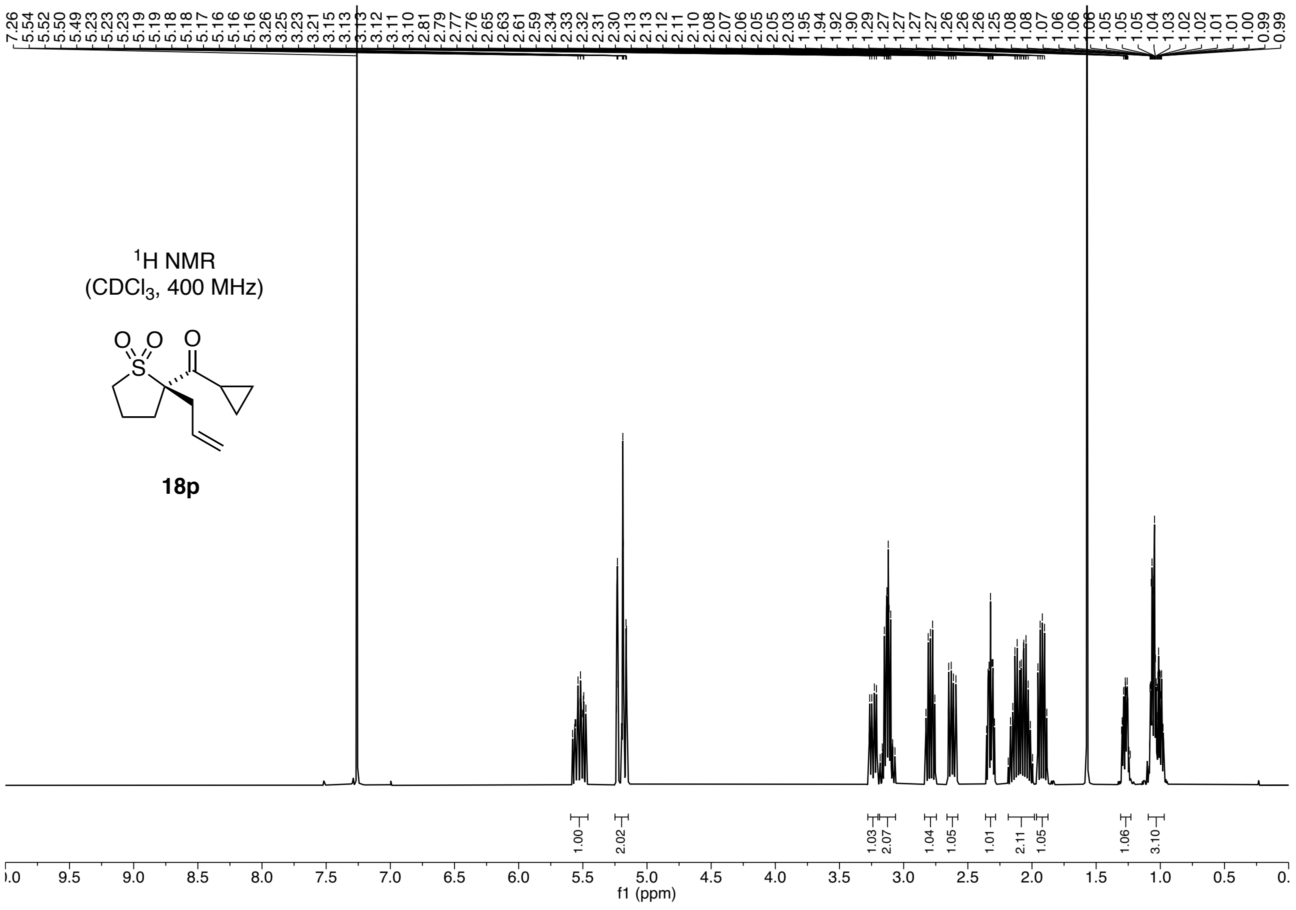

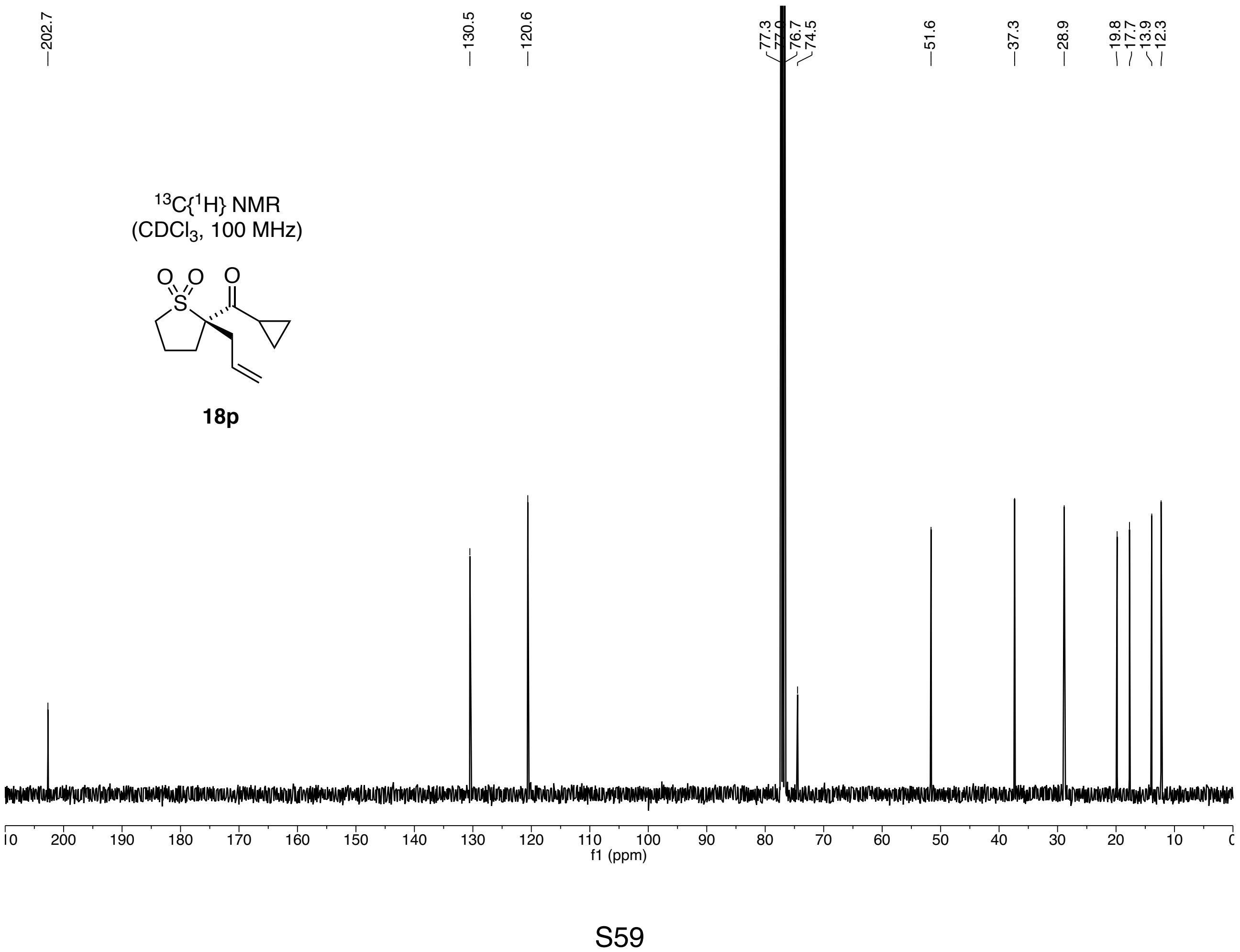

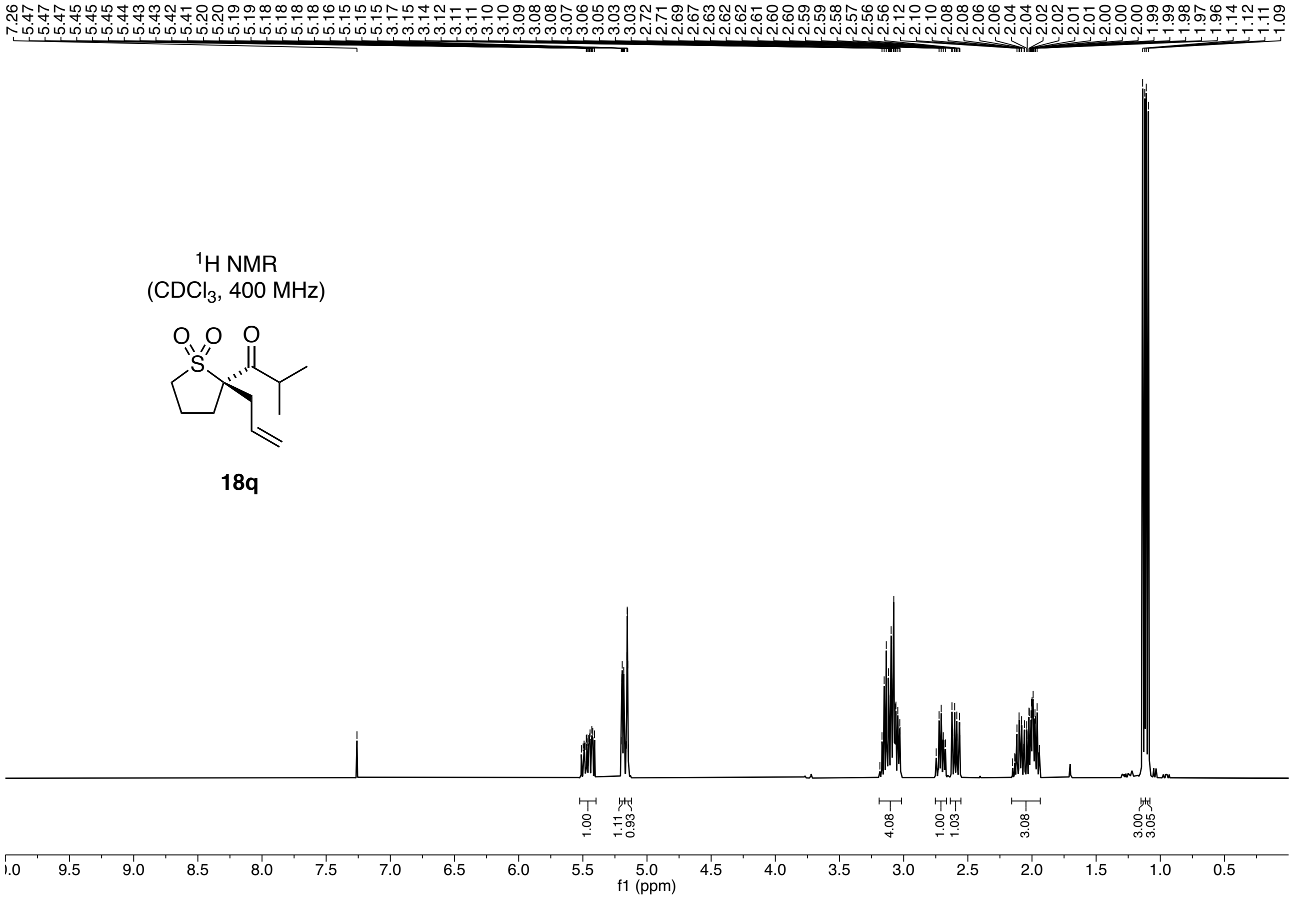
7.26 5.47 5.47 5.45 5.45 5.44 5.43 5.43 5.42 5.41 5.20 5.20 5.19 5.19 5.18 5.18 5.18 5.16 5.15 5.15 5.17 3.17 3.15 3.14 3.12 3.11 3.11 3.10 3.10 3.09 3.08 3.08 3.07 3.06 3.05 3.03 3.03 2.72 2.71 2.69 2.67 2.63 2.62 2.62 2.61 2.60 2.60 2.59 2.59 2.58 2.57 2.56 2.56 2.12 2.10 2.10 2.08 2.08 2.06 2.06 2.04 2.04 2.02 2.02 2.01 2.00 2.00 1.99 1.99 1.98 1.97 1.96 1.14 1.12 1.11 1.09
^{1}H NMR
(CDCl$_3$, 400 MHz)
18q
1.00 1.11 0.93 4.08 1.00 1.03 3.08 3.00 3.05
f1 (ppm)

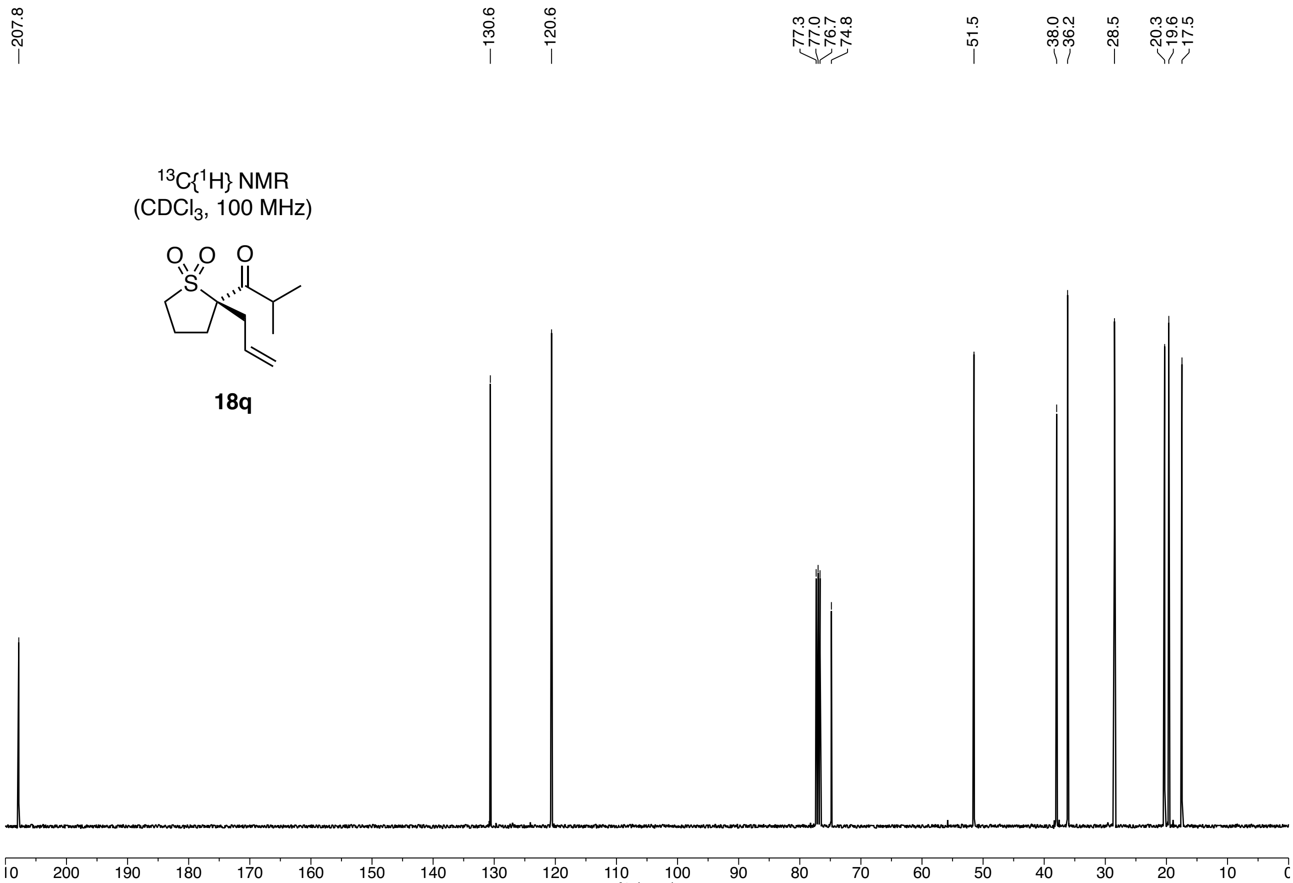
207.8 130.6 120.6 77.3 77.0 76.7 74.8 51.5 38.0 36.2 28.5 20.3 19.6 17.5
^{13}C{^{1}H} NMR
(CDCl$_3$, 100 MHz)
18q
f1 (ppm)

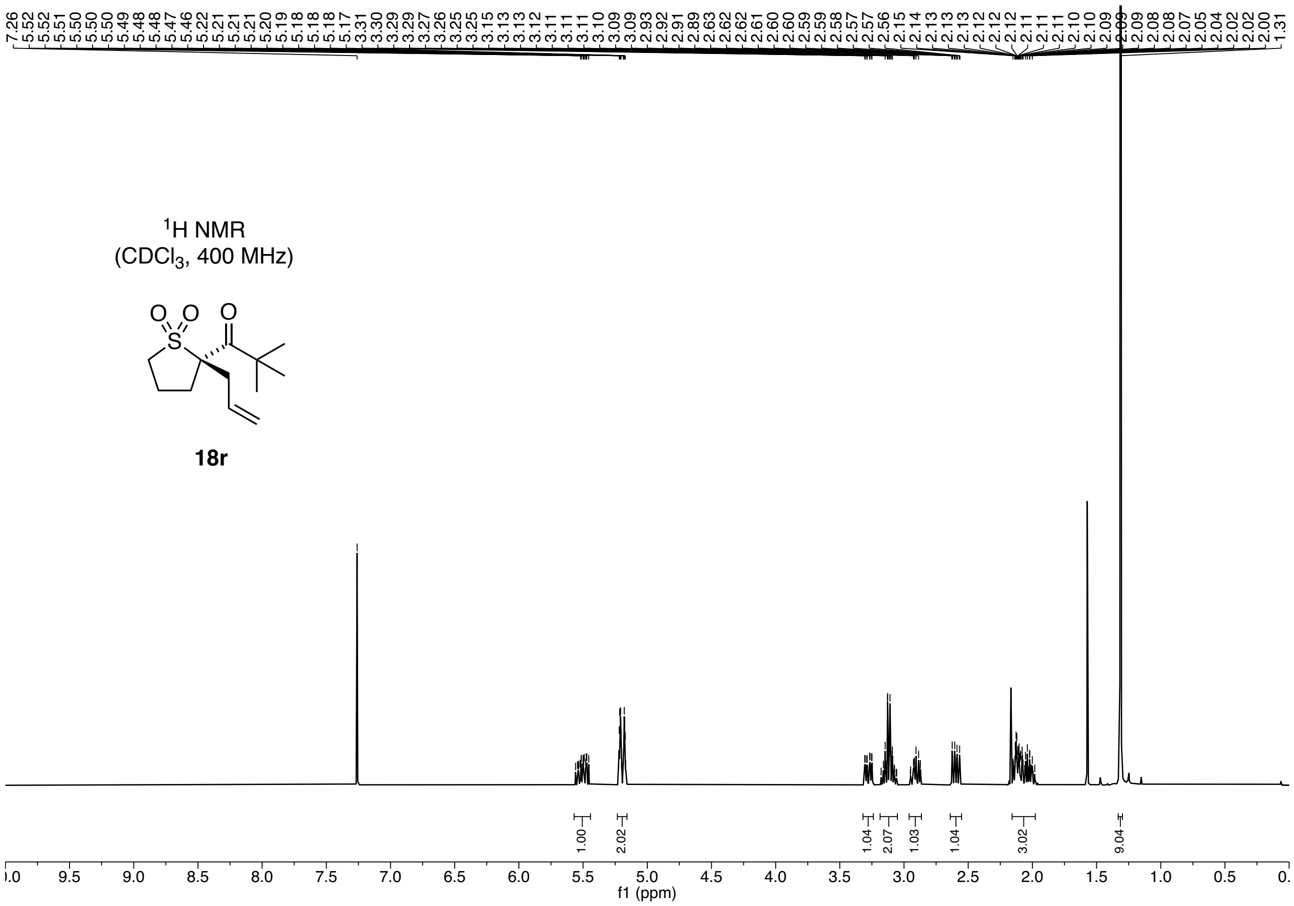
7.26 5.52 5.52 5.51 5.50 5.50 5.49 5.48 5.48 5.47 5.46 5.22 5.21 5.21 5.20 5.19 5.18 5.18 5.17 3.31 3.30 3.29 3.27 3.26 3.25 3.15 3.13 3.13 3.12 3.11 3.11 3.10 3.09 3.09 2.93 2.92 2.91 2.89 2.63 2.62 2.61 2.60 2.59 2.59 2.58 2.57 2.56 2.15 2.14 2.13 2.13 2.12 2.12 2.11 2.11 2.10 2.09 2.09 2.09 2.08 2.08 2.07 2.05 2.04 2.02 2.00 1.31
1H NMR
(CDCl3, 400 MHz)
18r
1.00 2.02 1.04 2.07 1.03 1.04 3.02 9.04
f1 (ppm)

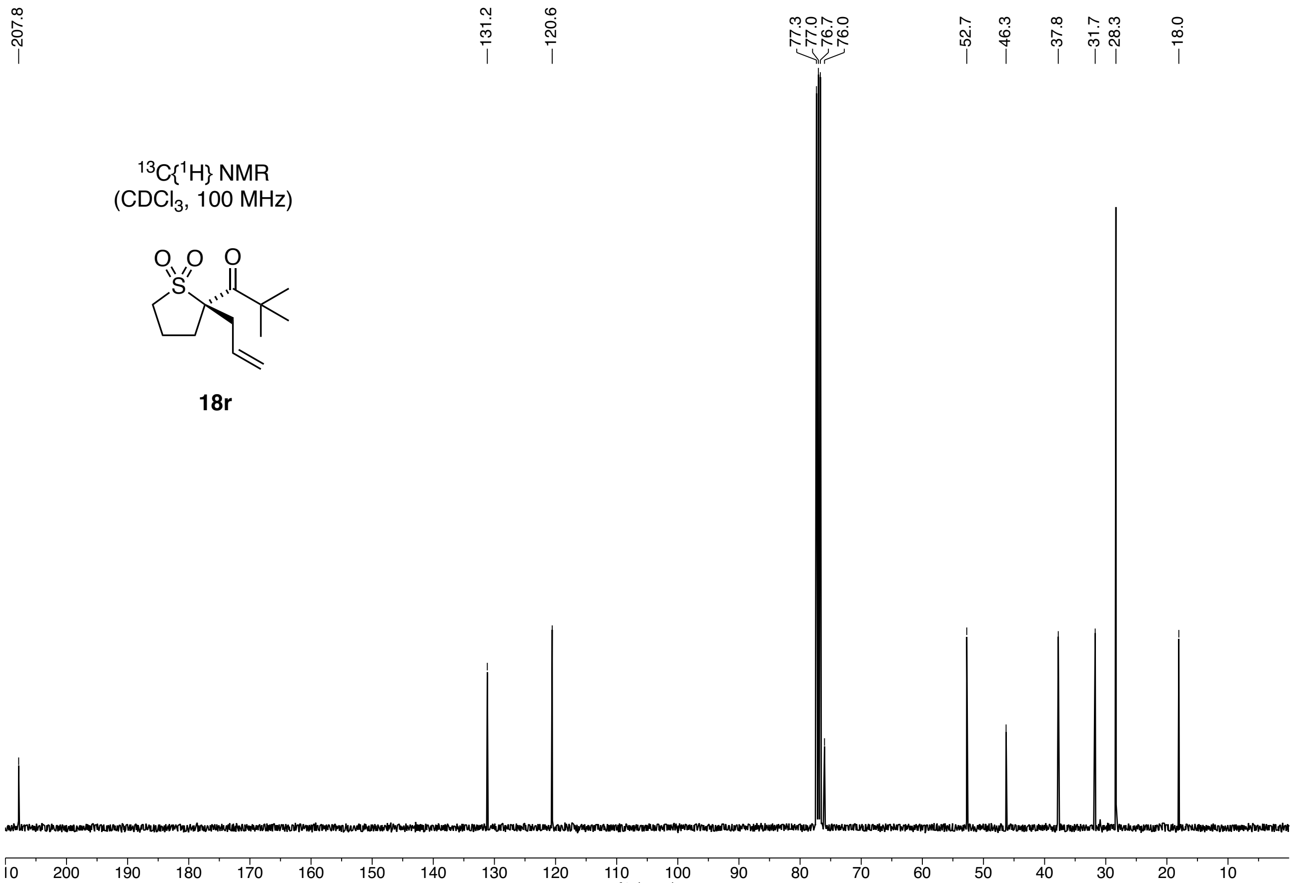
207.8 131.2 120.6 77.3 77.0 76.7 76.0 52.7 46.3 37.8 31.7 28.3 18.0
13C{1H} NMR
(CDCl3, 100 MHz)
18r
f1 (ppm)

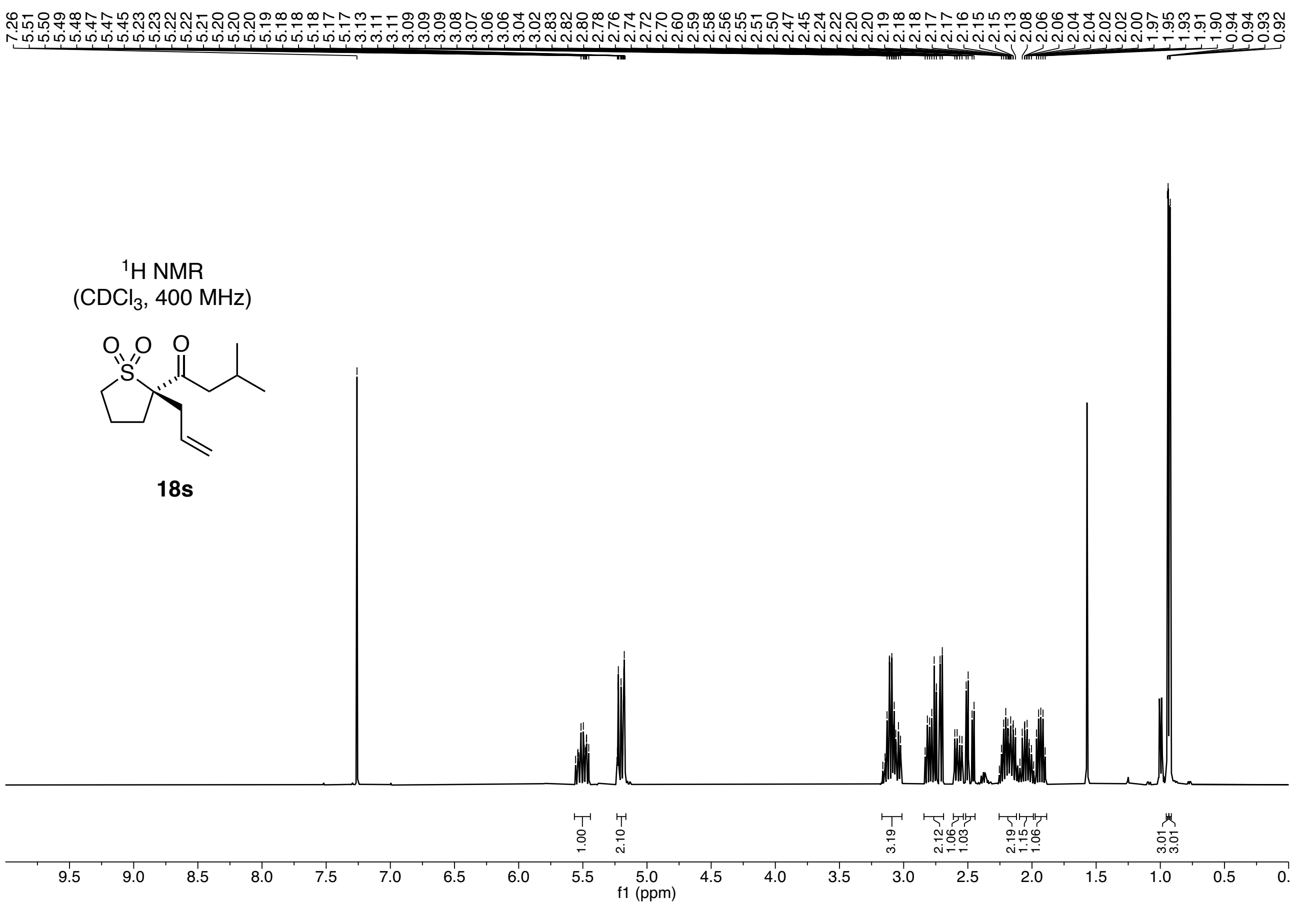

1H NMR
(CDCl3, 400 MHz)
18s
1.00
2.10
3.19
2.12
1.06
1.03
2.19
1.15
1.06
3.01
3.01
f1 (ppm)

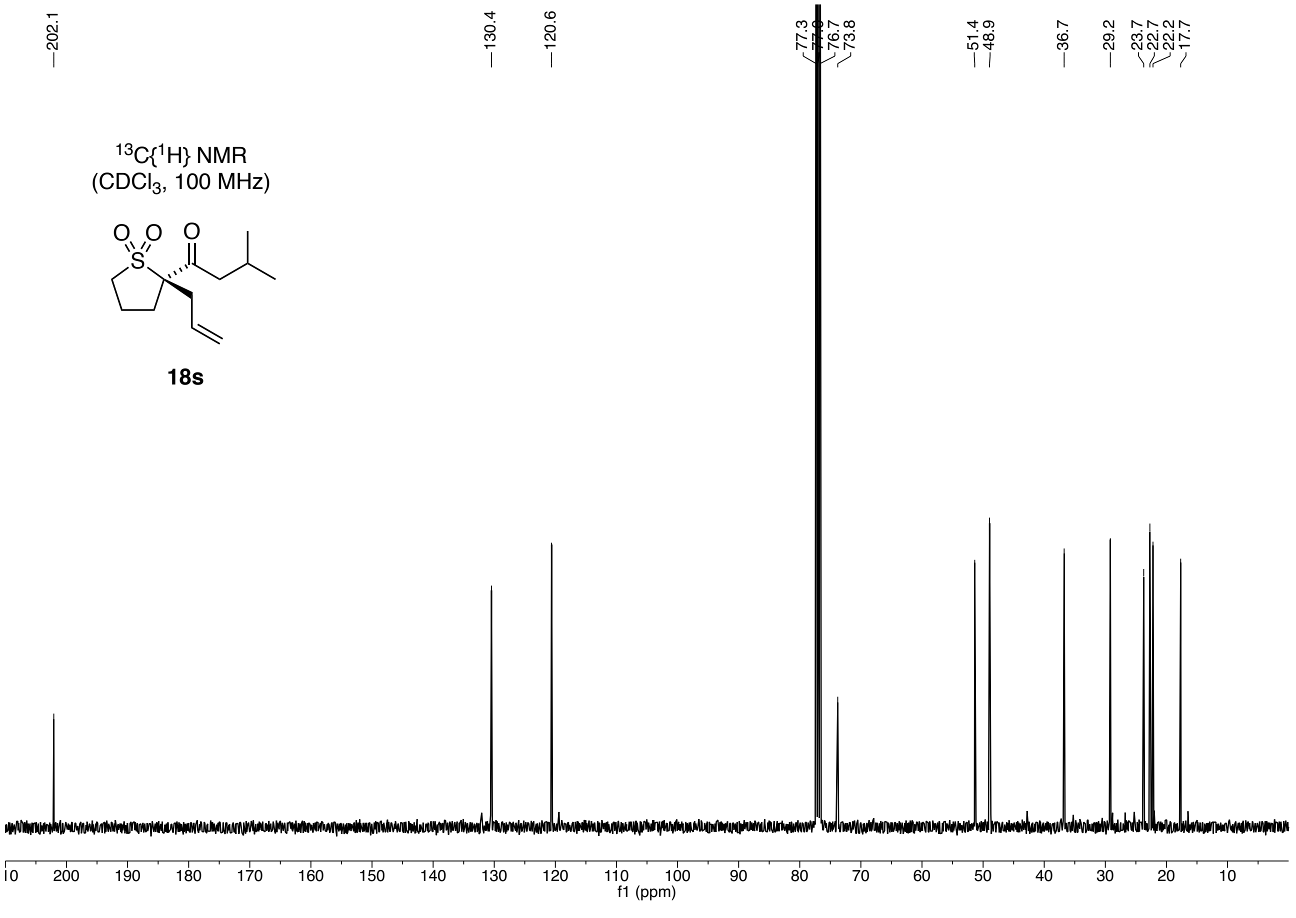

202.1
130.4
120.6
77.3
76.7
73.8
51.4
48.9
36.7
29.2
23.7
22.7
22.2
17.7
13C{1H} NMR
(CDCl3, 100 MHz)
18s
f1 (ppm)

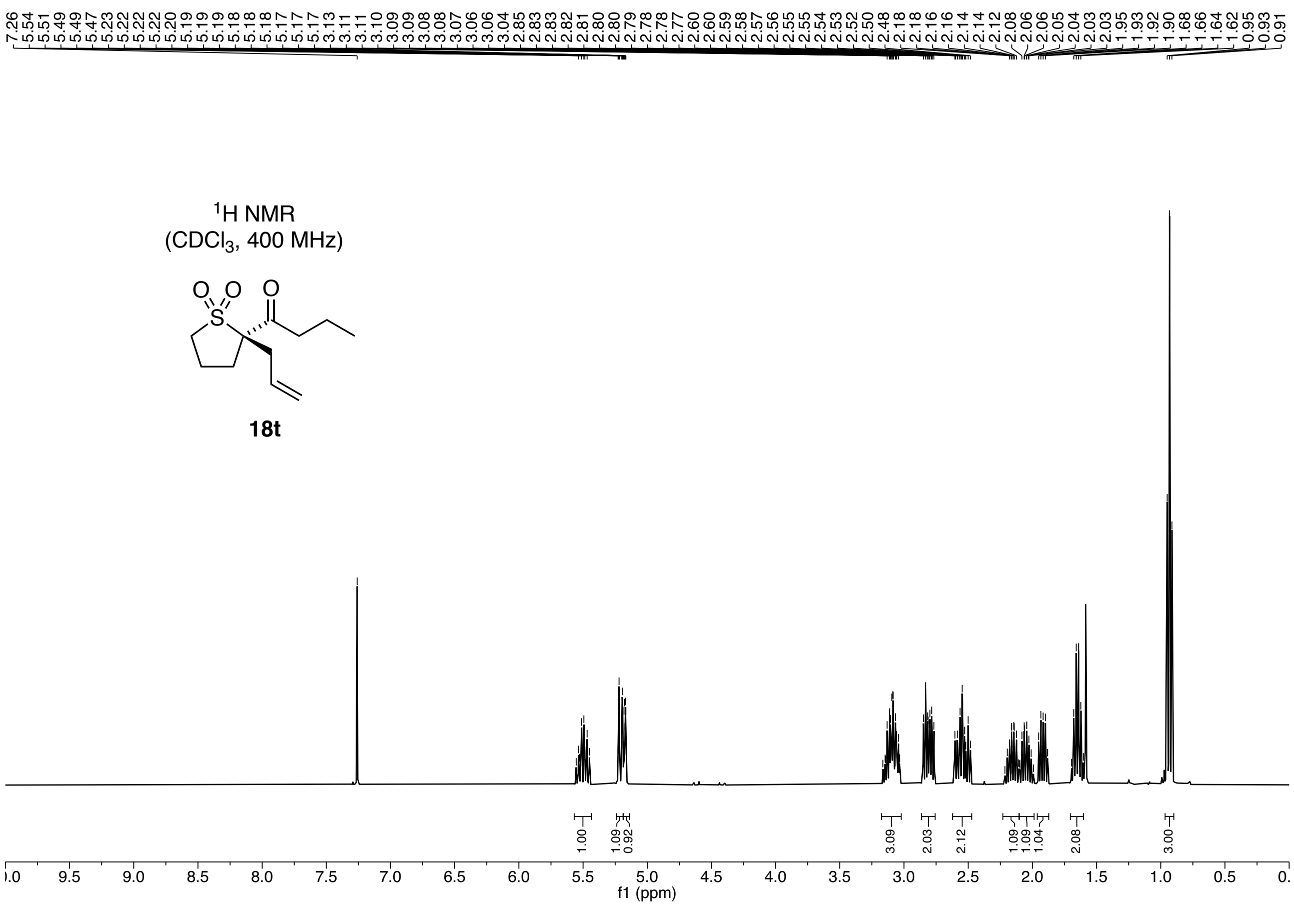
7.26 5.54 5.51 5.49 5.47 5.23 5.22 5.22 5.22 5.20 5.19 5.19 5.18 5.18 5.18 5.17 5.17 3.13 3.11 3.11 3.10 3.09 3.09 3.08 3.08 3.07 3.06 3.06 3.04 2.85 2.83 2.83 2.82 2.81 2.80 2.80 2.79 2.78 2.78 2.77 2.60 2.60 2.59 2.58 2.57 2.56 2.55 2.55 2.54 2.53 2.52 2.50 2.48 2.18 2.18 2.16 2.16 2.14 2.14 2.12 2.08 2.06 2.06 2.05 2.04 2.03 2.03 1.95 1.93 1.92 1.90 1.68 1.66 1.64 1.62 0.95 0.93 0.91
1H NMR
(CDCl3, 400 MHz)
18t
1.00 1.09 0.92 3.09 2.03 2.12 1.09 1.09 1.04 2.08 3.00
f1 (ppm)

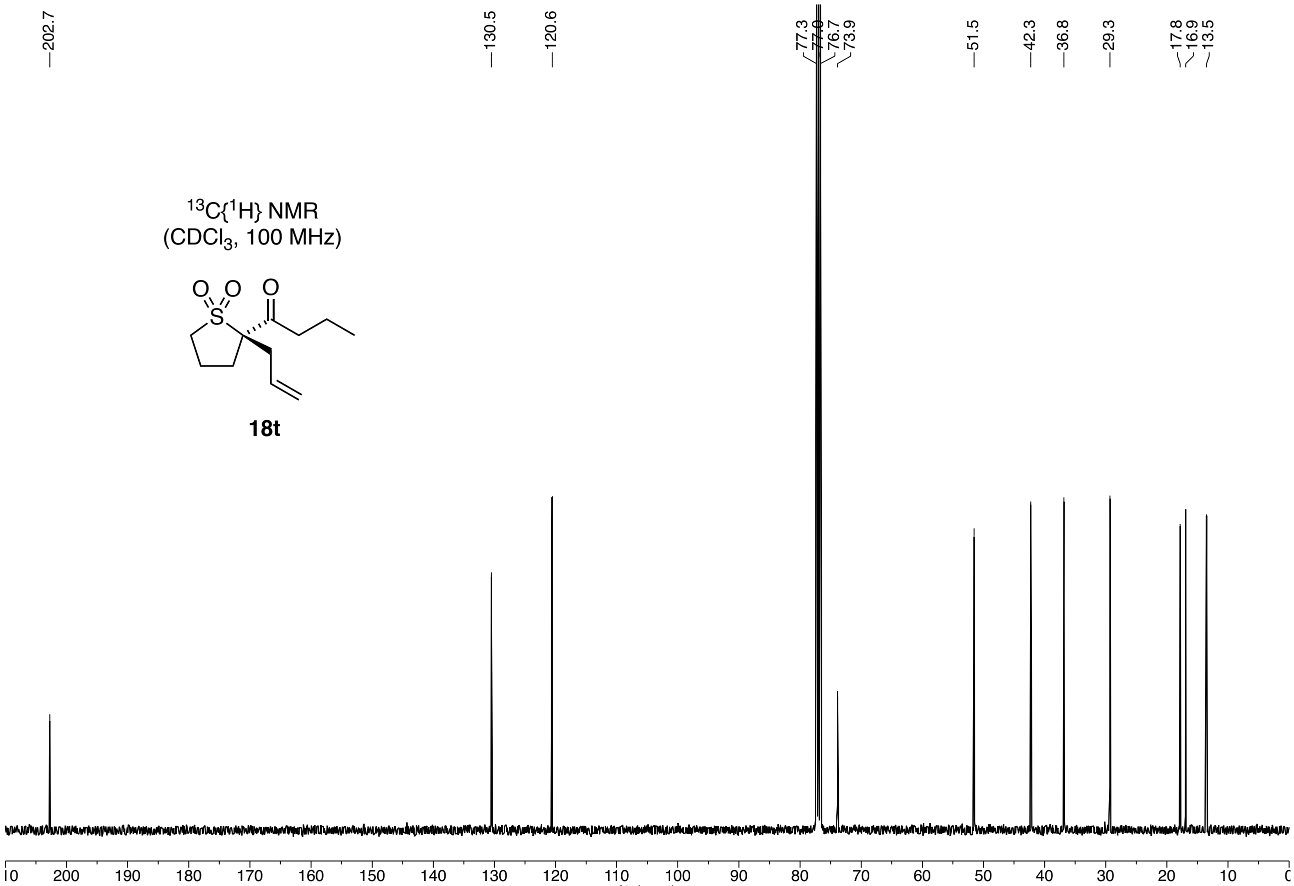
202.7 130.5 120.6 77.3 77.0 76.7 73.9 51.5 42.3 36.8 29.3 17.8 16.9 13.5
13C{1H} NMR
(CDCl3, 100 MHz)
18t
f1 (ppm)

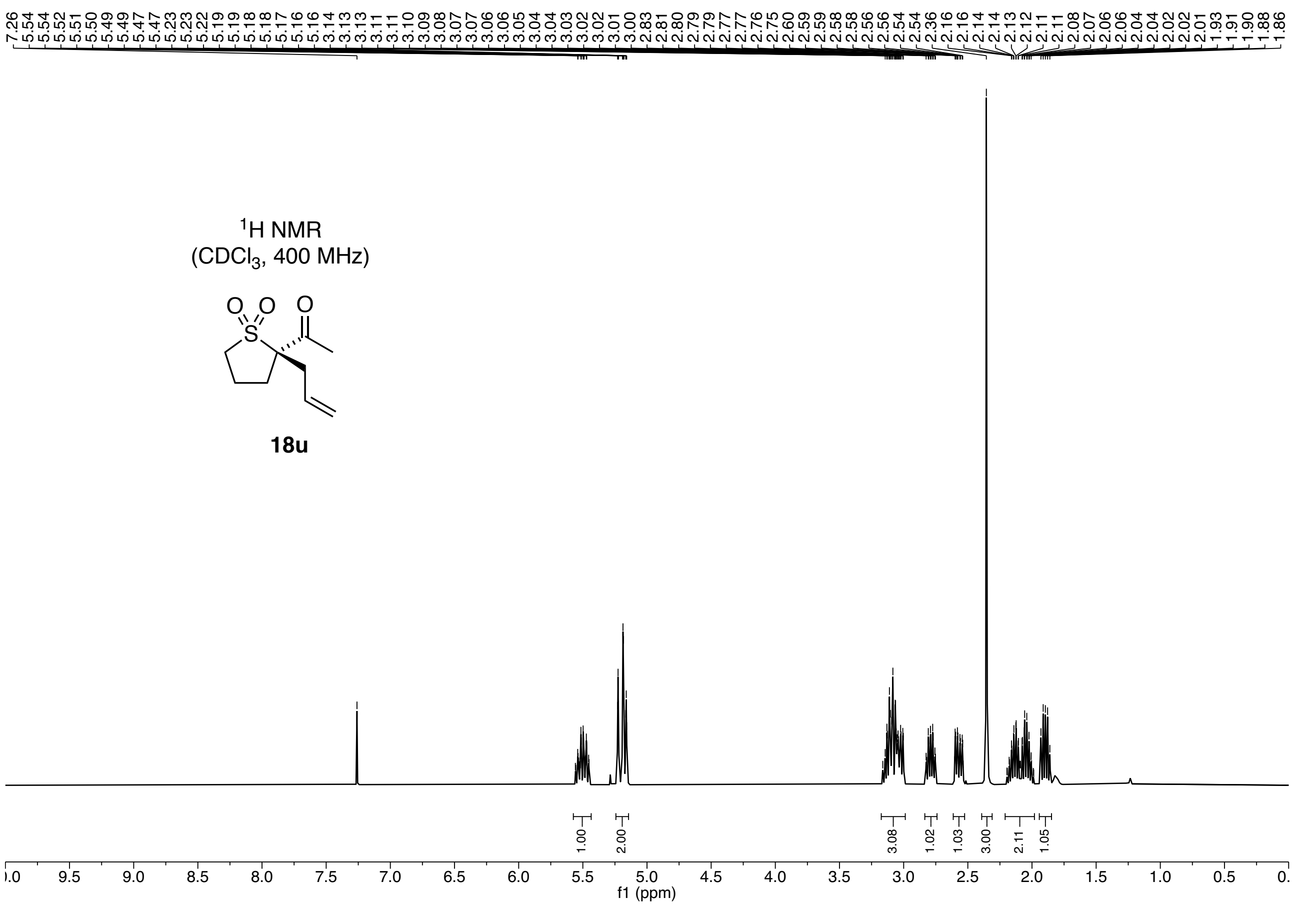

1H NMR
(CDCl3, 400 MHz)
18u
1.00
2.00
3.08
1.02
1.03
3.00
2.11
1.05
f1 (ppm)

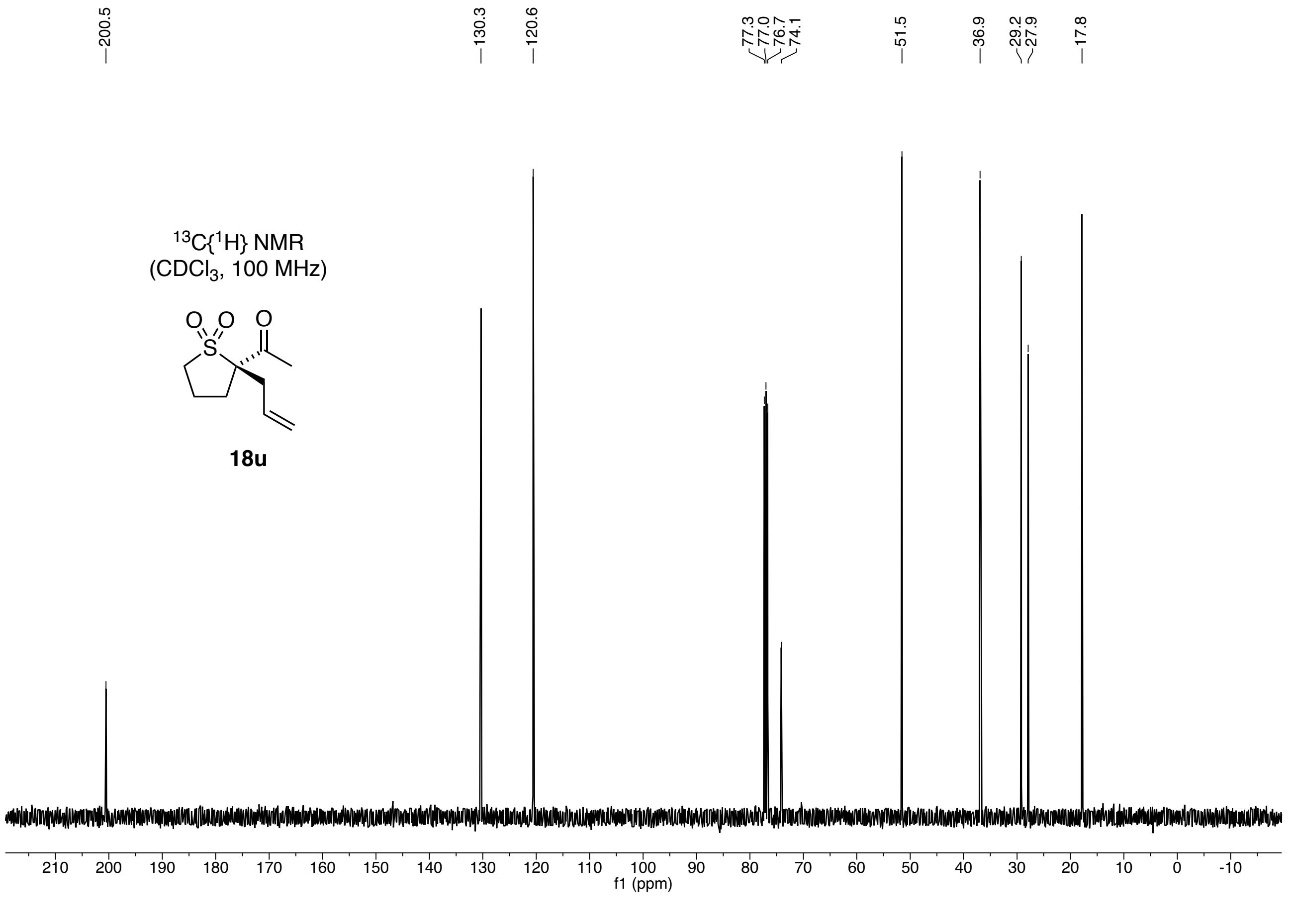

200.5
130.3
120.6
77.3
77.0
76.7
74.1
51.5
36.9
29.2
27.9
17.8
13C{1H} NMR
(CDCl3, 100 MHz)
18u
f1 (ppm)

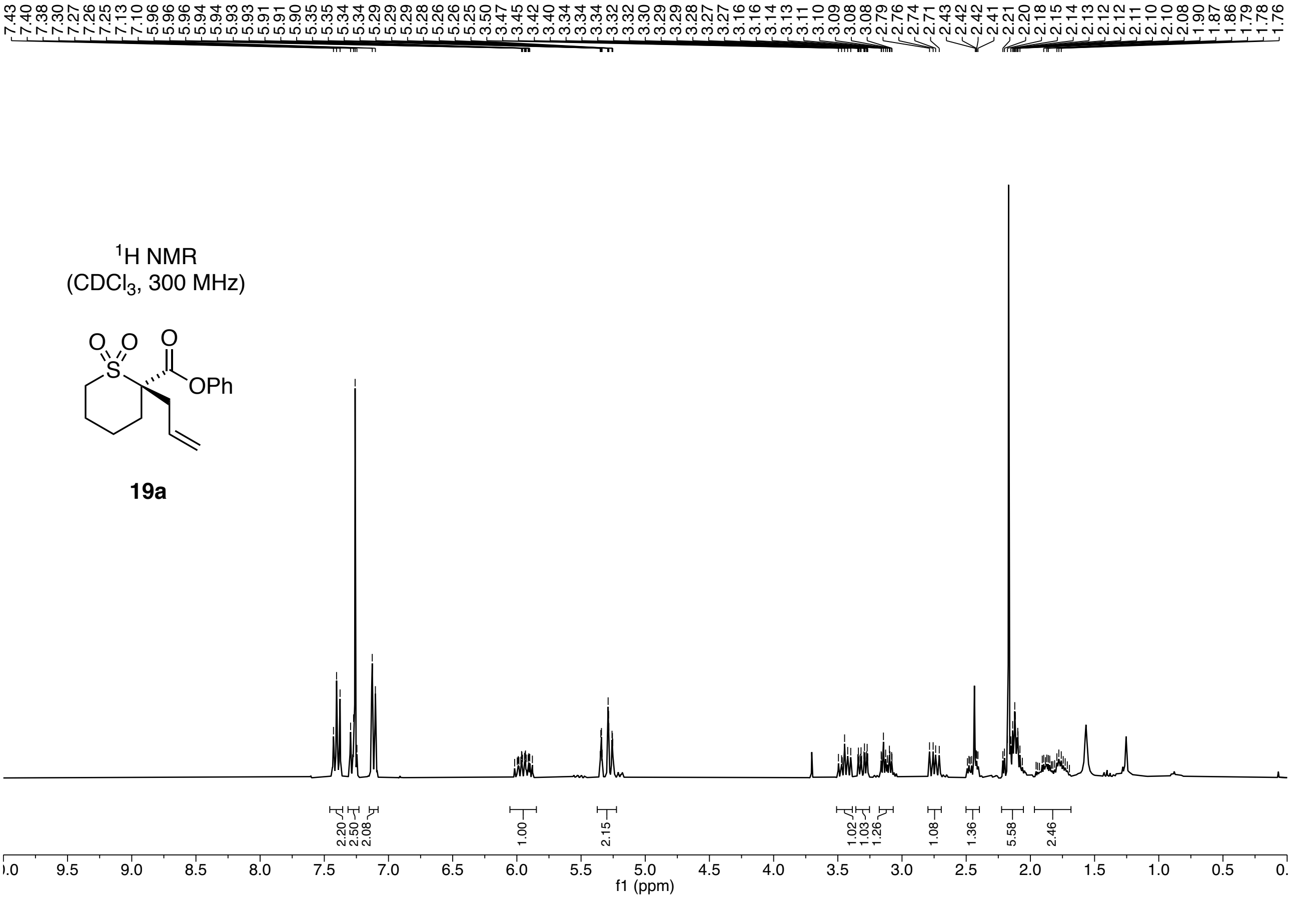
7.43 7.40 7.38 7.30 7.27 7.26 7.25 7.13 7.10 5.96 5.96 5.94 5.93 5.91 5.91 5.90 5.35 5.35 5.34 5.29 5.29 5.28 5.26 5.26 5.25 3.50 3.47 3.45 3.42 3.40 3.34 3.34 3.32 3.32 3.30 3.29 3.28 3.27 3.16 3.16 3.14 3.13 3.11 3.10 3.09 3.08 3.08 2.79 2.76 2.74 2.71 2.43 2.42 2.42 2.41 2.21 2.20 2.18 2.15 2.14 2.13 2.12 2.12 2.11 2.10 2.10 2.08 1.90 1.87 1.86 1.79 1.78 1.76
^{1}H NMR
($CDCl_3$, 300 MHz)
O O O
S
OPh
19a
2.20 2.50 2.08 1.00 2.15 1.02 1.03 1.26 1.08 1.36 5.58 2.46
9.5 9.0 8.5 8.0 7.5 7.0 6.5 6.0 5.5 5.0 4.5 4.0 3.5 3.0 2.5 2.0 1.5 1.0 0.5
f1 (ppm)

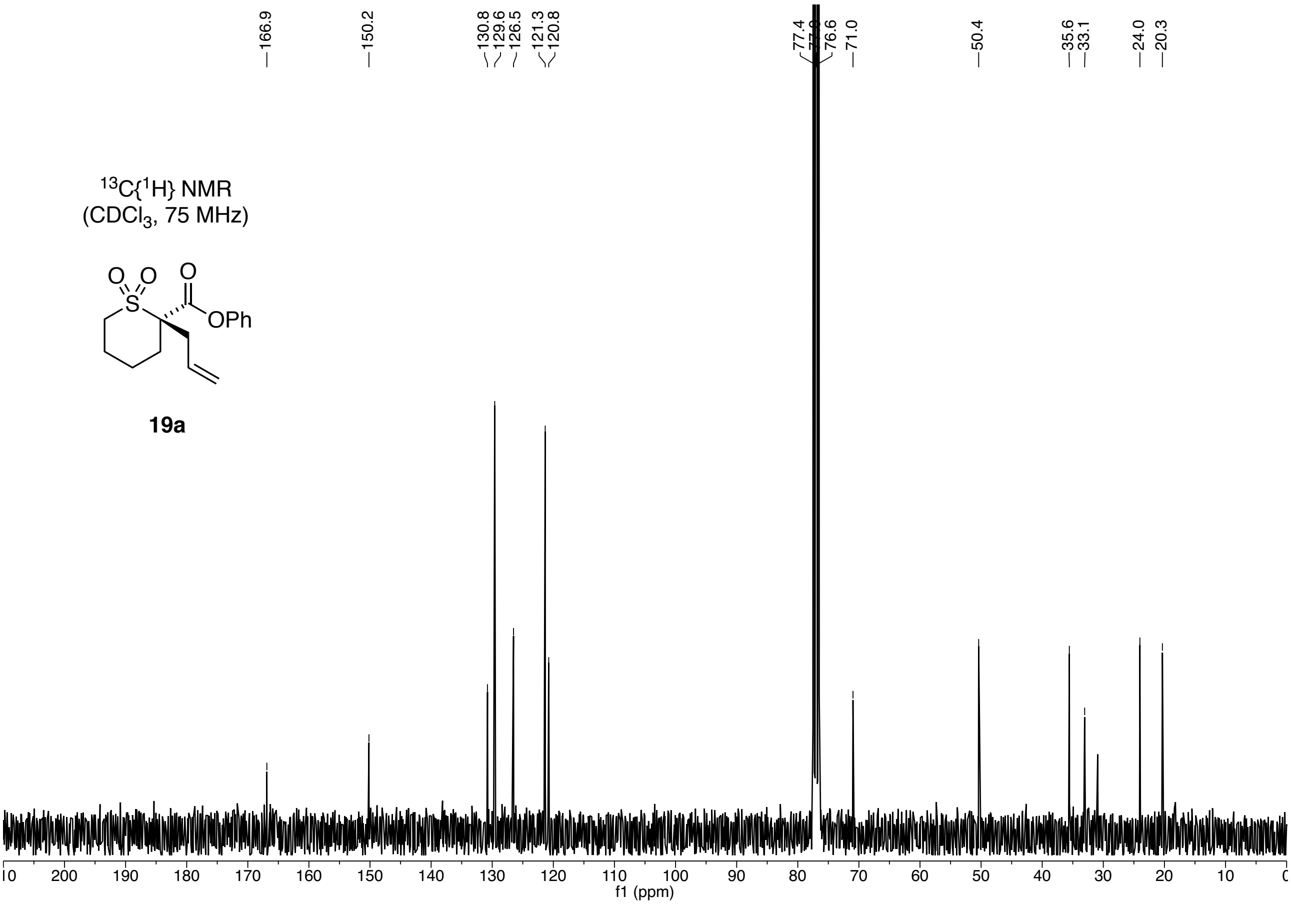
166.9 150.2 130.8 129.6 126.5 121.3 120.8 77.4 77.0 76.6 71.0 50.4 35.6 33.1 24.0 20.3
^{13}C{^{1}H} NMR
($CDCl_3$, 75 MHz)
O O O
S
OPh
19a
200 190 180 170 160 150 140 130 120 110 100 90 80 70 60 50 40 30 20 10
f1 (ppm)

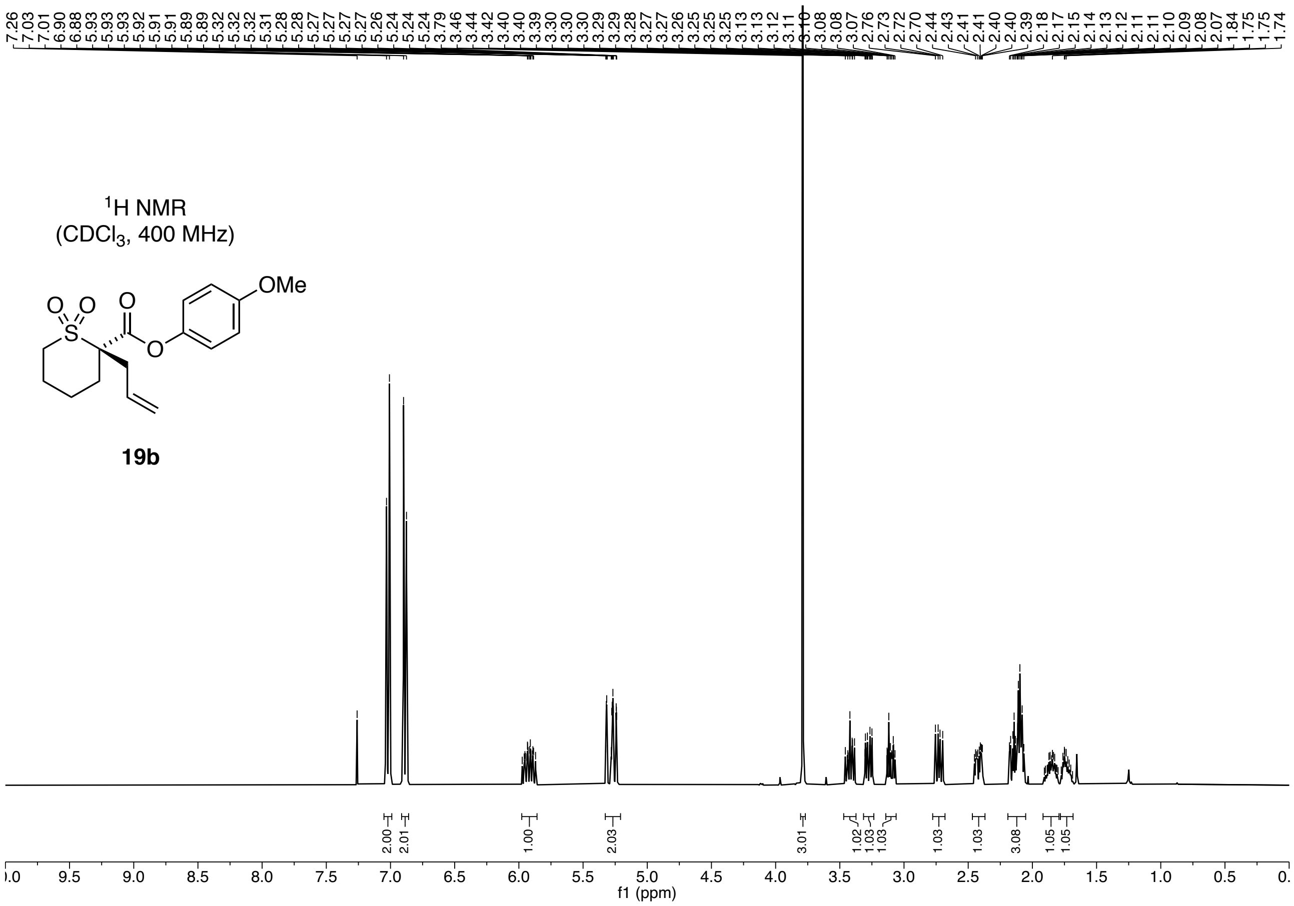
7.26 7.03 7.01 6.90 6.88 5.93 5.93 5.92 5.91 5.91 5.89 5.32 5.32 5.32 5.31 5.28 5.27 5.27 5.27 5.26 5.24 5.24 5.24 3.79 3.46 3.44 3.42 3.40 3.40 3.39 3.30 3.30 3.30 3.29 3.29 3.28 3.27 3.27 3.26 3.25 3.25 3.13 3.13 3.12 3.11 3.10 3.08 3.08 3.07 2.76 2.73 2.72 2.70 2.44 2.43 2.41 2.41 2.40 2.40 2.39 2.18 2.17 2.15 2.14 2.13 2.12 2.11 2.11 2.10 2.09 2.08 2.07 1.84 1.75 1.74
^{1}H NMR
($CDCl_3$, 400 MHz)
OMe
O
O
S
O
O
19b
2.00 2.01 1.00 2.03 3.01 1.02 1.03 1.03 1.03 1.03 3.08 1.05 1.05
9.5 9.0 8.5 8.0 7.5 7.0 6.5 6.0 5.5 5.0 4.5 4.0 3.5 3.0 2.5 2.0 1.5 1.0 0.5
f1 (ppm)

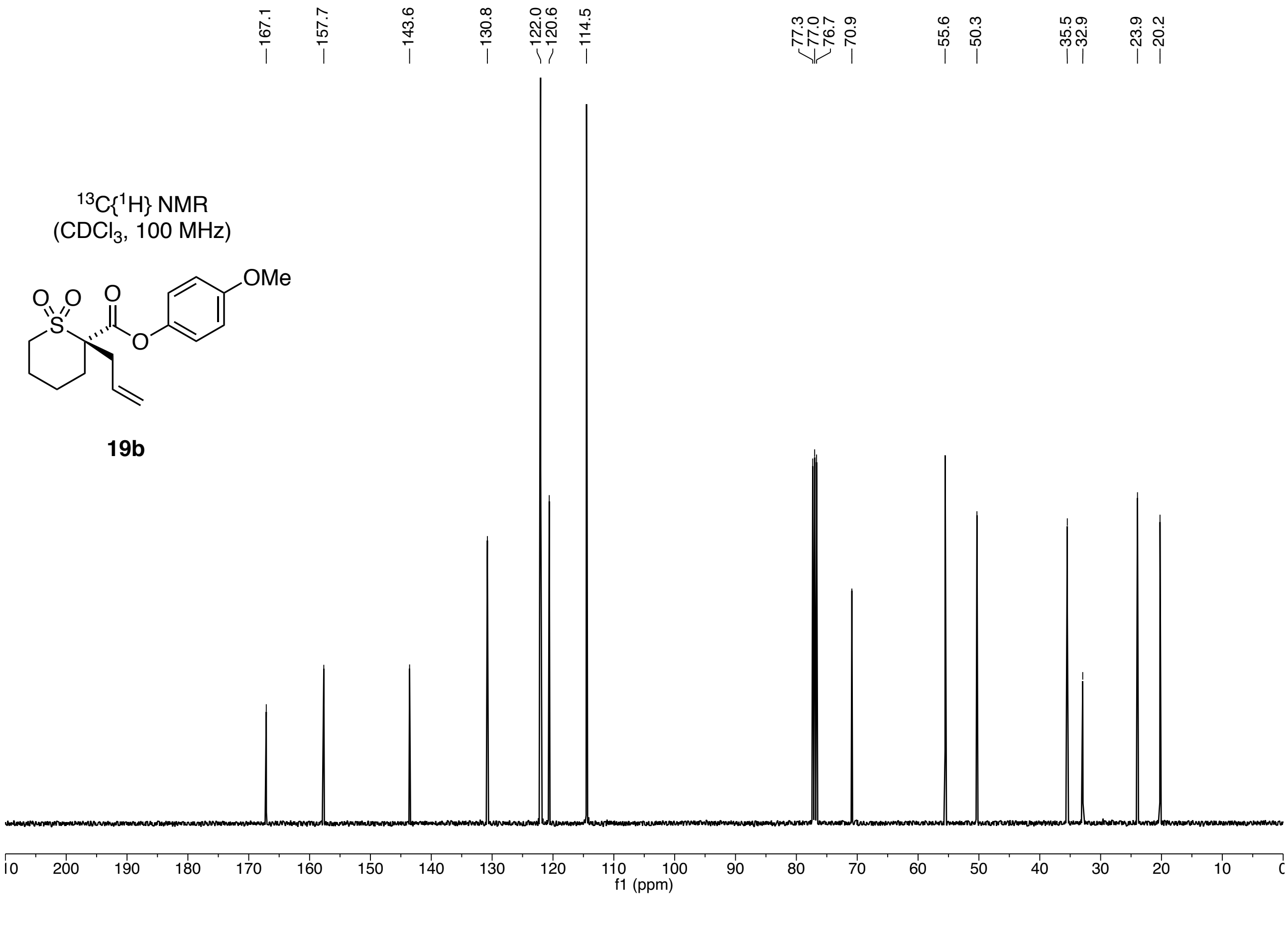
167.1 157.7 143.6 130.8 122.0 120.6 114.5 77.3 77.0 76.7 70.9 55.6 50.3 35.5 32.9 23.9 20.2
^{13}C{^{1}H} NMR
($CDCl_3$, 100 MHz)
OMe
O
O
S
O
O
19b
200 190 180 170 160 150 140 130 120 110 100 90 80 70 60 50 40 30 20 10
f1 (ppm)

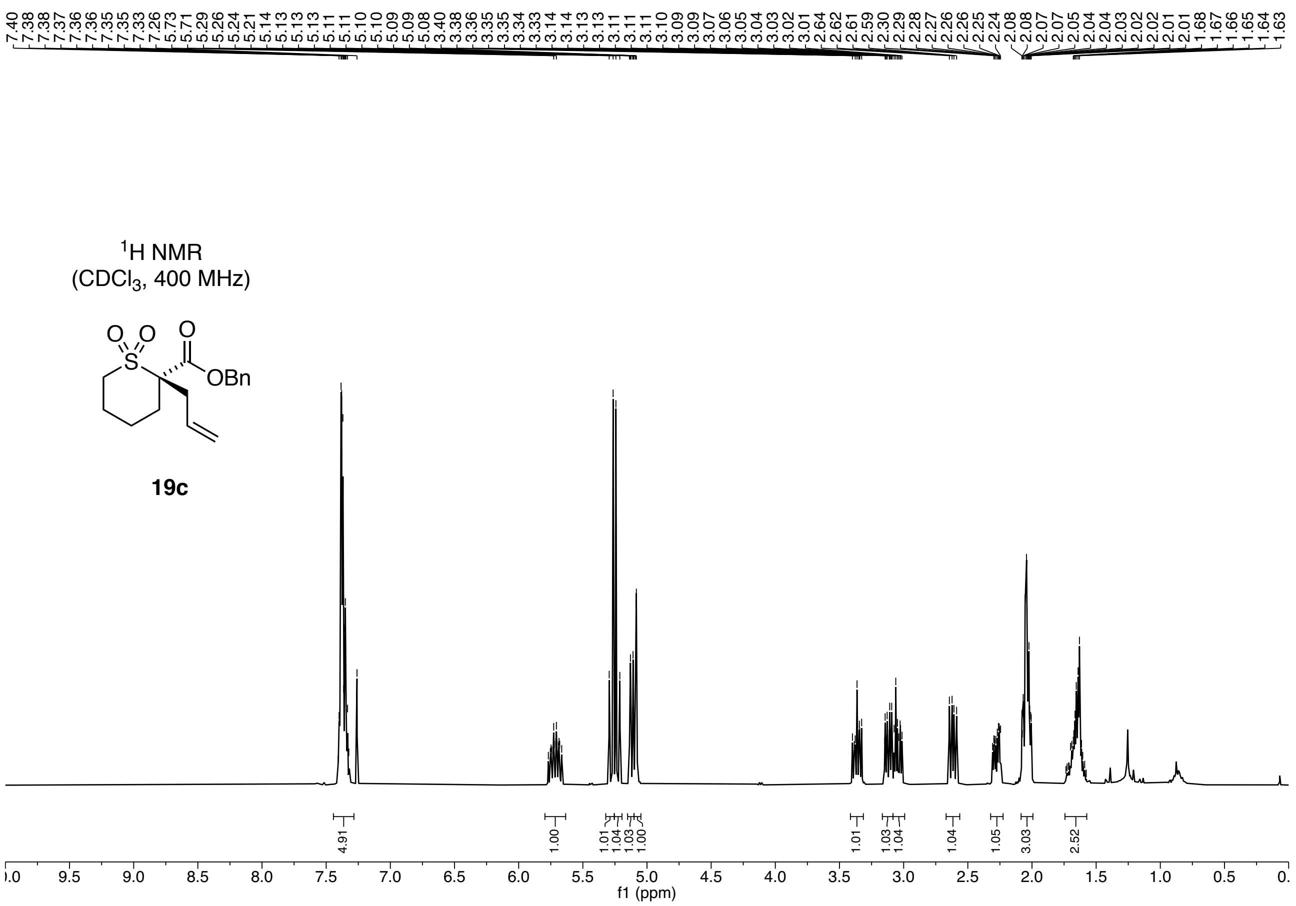
7.40 7.38 7.38 7.37 7.36 7.36 7.35 7.35 7.33 7.26 5.73 5.71 5.29 5.26 5.24 5.21 5.14 5.13 5.13 5.13 5.11 5.11 5.10 5.10 5.09 5.09 5.08 3.40 3.38 3.36 3.35 3.34 3.33 3.33 3.14 3.14 3.13 3.13 3.11 3.11 3.11 3.10 3.09 3.09 3.07 3.06 3.05 3.04 3.03 3.02 3.01 2.64 2.62 2.61 2.59 2.30 2.29 2.28 2.27 2.26 2.25 2.24 2.08 2.07 2.07 2.05 2.04 2.04 2.03 2.02 2.02 2.01 1.68 1.67 1.66 1.65 1.64 1.63
1H NMR
(CDCl3, 400 MHz)
OBn
19c
4.91 1.00 1.01 1.04 1.03 1.00 1.01 1.03 1.04 1.04 1.05 3.03 2.52
f1 (ppm)

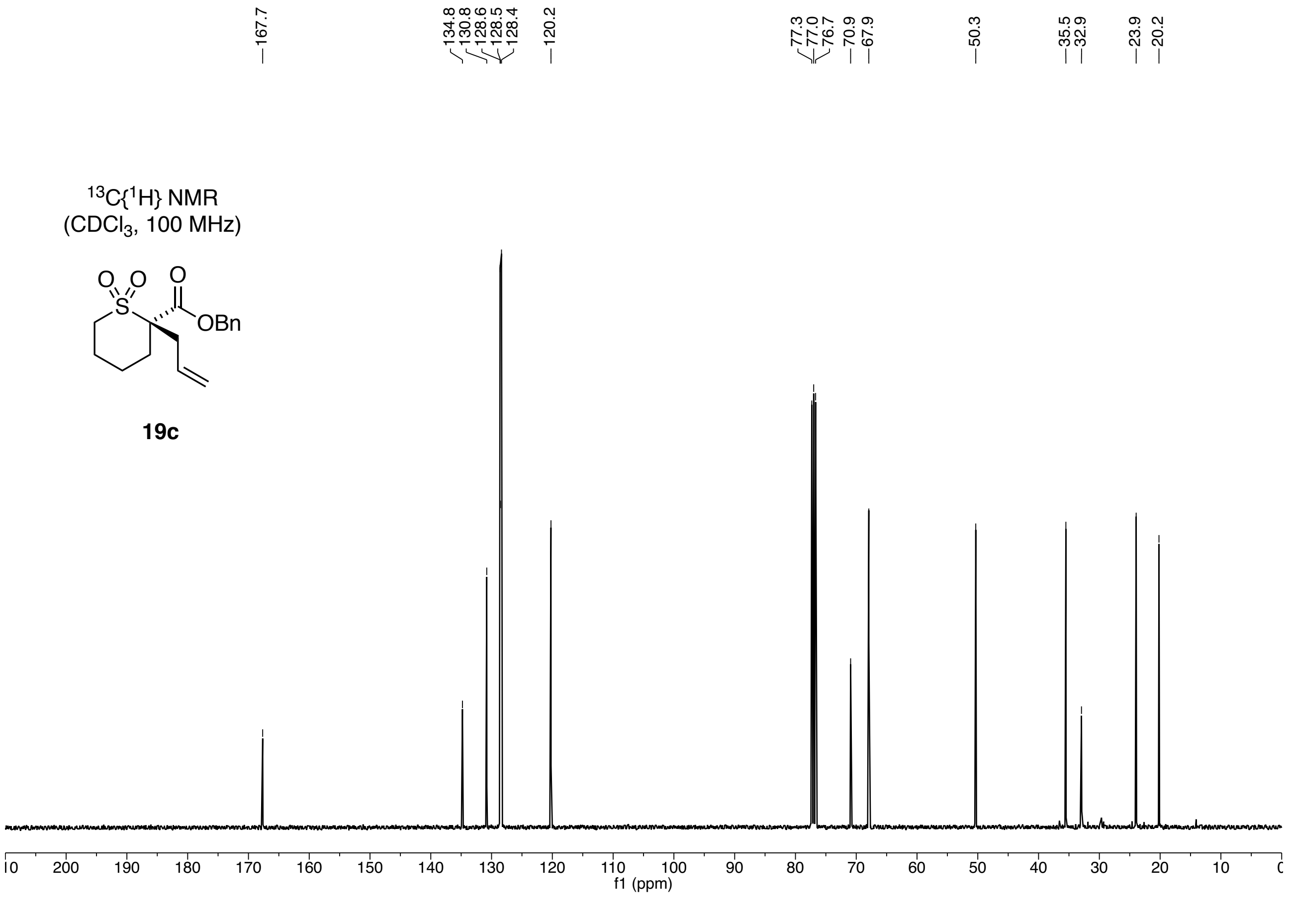
167.7 134.8 130.8 128.6 128.5 128.4 120.2 77.3 77.0 76.7 70.9 67.9 50.3 35.5 32.9 23.9 20.2
13C{1H} NMR
(CDCl3, 100 MHz)
OBn
19c
f1 (ppm)

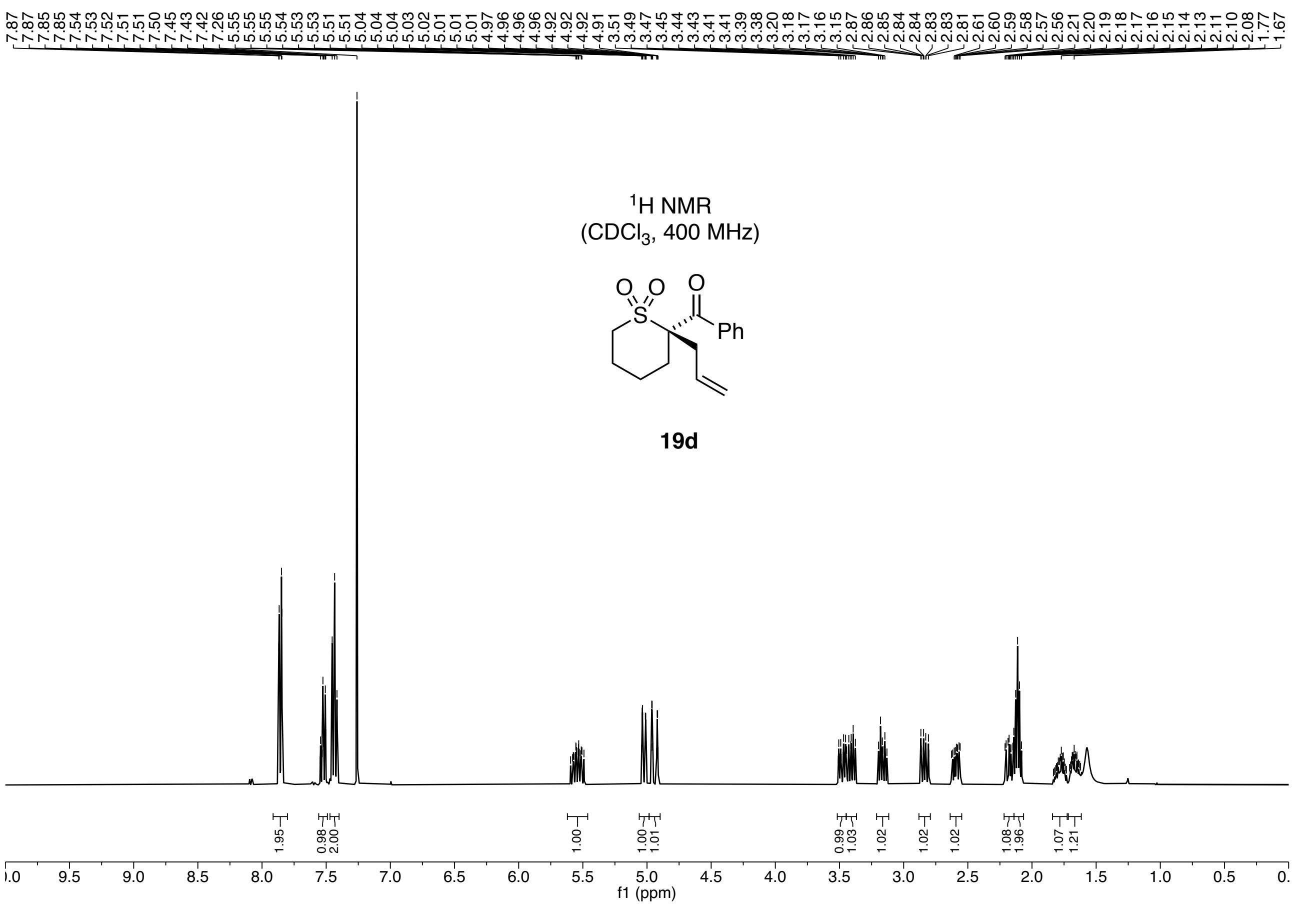
1H NMR
(CDCl3, 400 MHz)
19d
f1 (ppm)

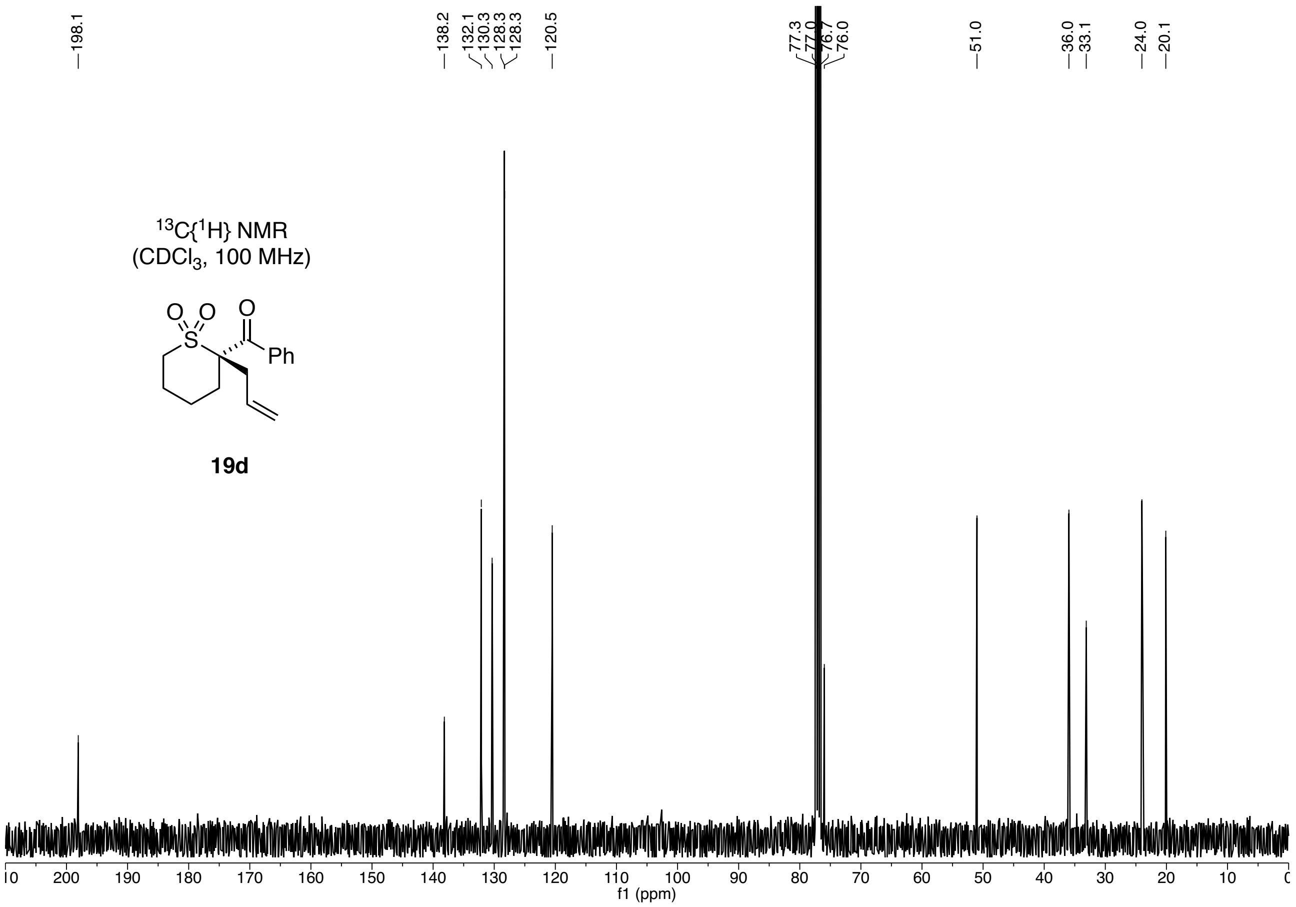
13C{1H} NMR
(CDCl3, 100 MHz)
19d
198.1
138.2
132.1
130.3
128.3
128.3
120.5
77.3
77.0
76.7
76.0
51.0
36.0
33.1
24.0
20.1
f1 (ppm)

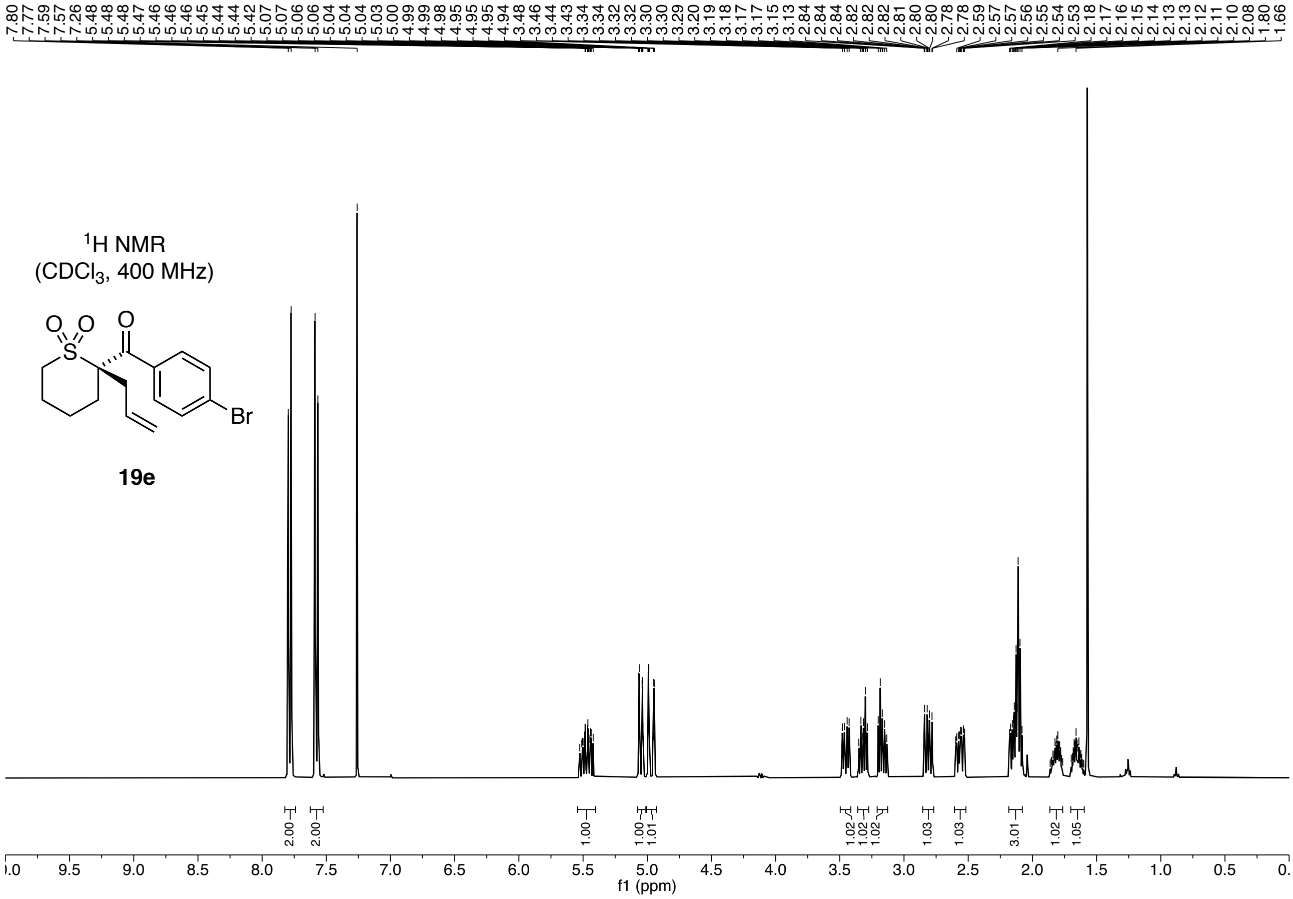
7.80 7.77 7.59 7.57 7.26 5.48 5.48 5.47 5.46 5.46 5.45 5.44 5.44 5.42 5.07 5.06 5.04 5.04 5.04 5.03 5.00 4.99 4.99 4.98 4.95 4.95 4.94 3.48 3.46 3.44 3.43 3.34 3.34 3.32 3.32 3.30 3.30 3.29 3.20 3.19 3.18 3.17 3.17 3.15 3.13 2.84 2.84 2.84 2.82 2.82 2.82 2.81 2.80 2.80 2.78 2.59 2.57 2.57 2.56 2.55 2.54 2.53 2.18 2.17 2.16 2.15 2.14 2.13 2.13 2.12 2.11 2.10 2.08 1.80 1.66
^{1}H NMR
(CDCl$_3$, 400 MHz)
O O O
S
Br
19e
2.00 2.00 1.00 1.00 1.01 1.02 1.02 1.02 1.03 1.03 3.01 1.02 1.05
f1 (ppm)

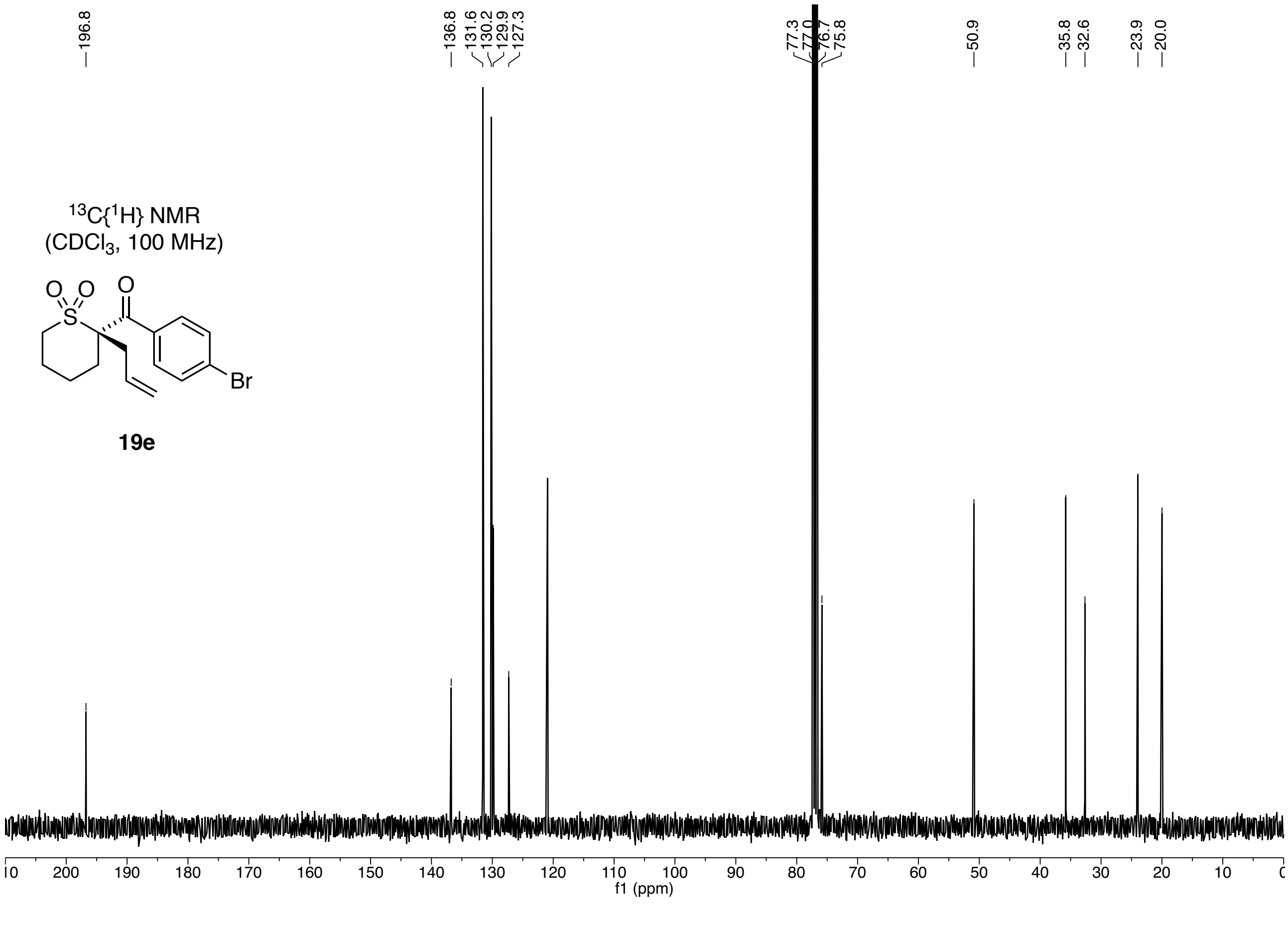
196.8 136.8 131.6 130.2 129.9 127.3 77.3 77.0 76.7 75.8 50.9 35.8 32.6 23.9 20.0
^{13}C{^{1}H} NMR
(CDCl$_3$, 100 MHz)
O O O
S
Br
19e
f1 (ppm)

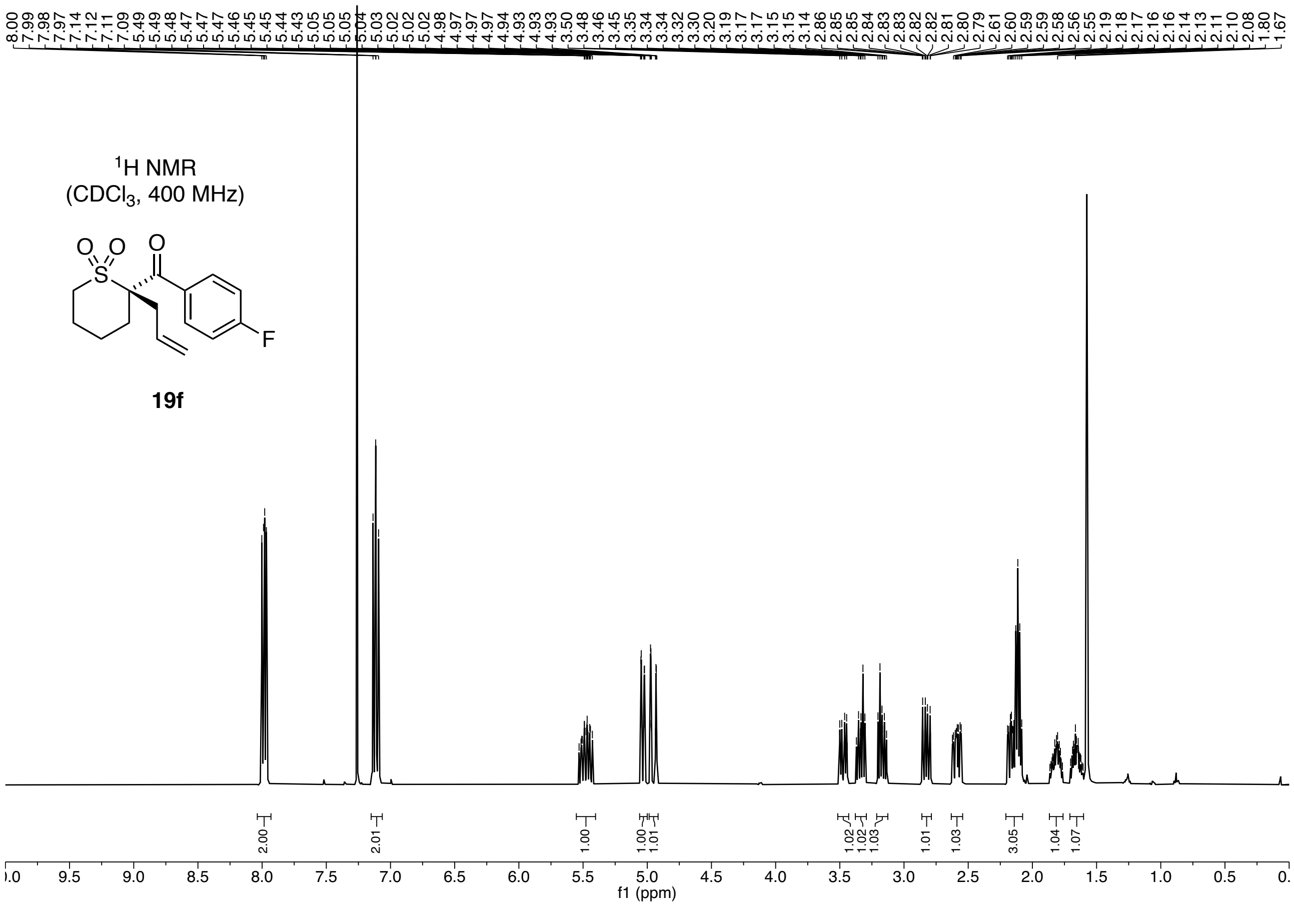

8.00 7.99 7.98 7.97 7.14 7.12 7.11 7.09 5.49 5.49 5.48 5.47 5.47 5.46 5.45 5.45 5.44 5.43 5.05 5.05 5.05 5.04 5.03 5.02 5.02 5.02 4.98 4.97 4.97 4.97 4.94 4.93 4.93 4.93 3.50 3.48 3.46 3.45 3.35 3.34 3.34 3.32 3.30 3.20 3.19 3.17 3.17 3.15 3.15 3.14 2.86 2.85 2.85 2.84 2.83 2.83 2.82 2.82 2.81 2.81 2.80 2.79 2.61 2.60 2.59 2.59 2.58 2.56 2.55 2.19 2.18 2.17 2.16 2.16 2.14 2.13 2.11 2.10 2.08 1.80 1.67
^{1}H NMR
(CDCl$_3$, 400 MHz)
19f
2.00 2.01 1.00 1.00 1.01 1.02 1.02 1.03 1.01 1.03 3.05 1.04 1.07
f1 (ppm)

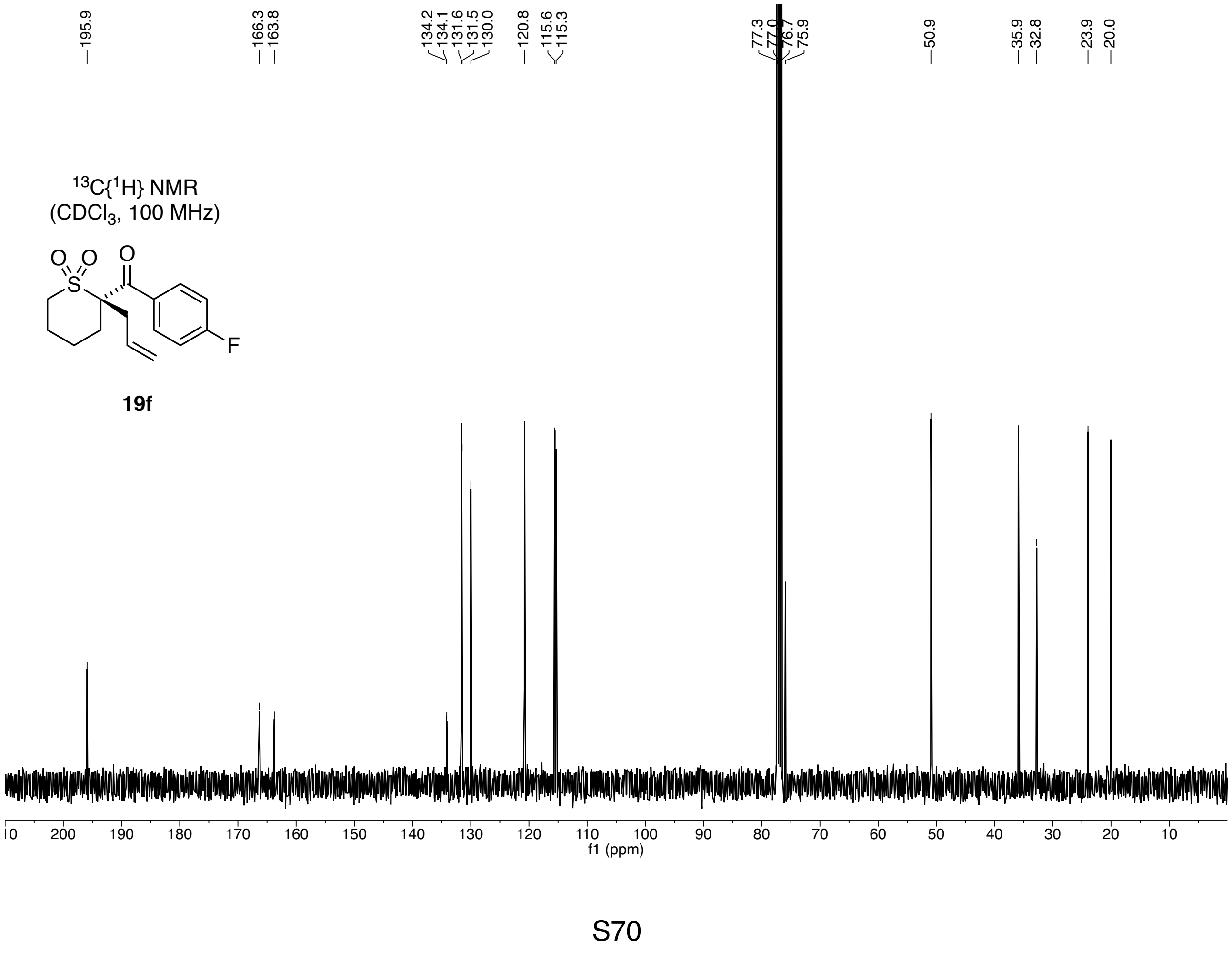

195.9 166.3 163.8 134.2 134.1 131.6 131.5 130.0 120.8 115.6 115.3 77.3 77.0 76.7 75.9 50.9 35.9 32.8 23.9 20.0
^{13}C{^{1}H} NMR
(CDCl$_3$, 100 MHz)
19f
f1 (ppm)

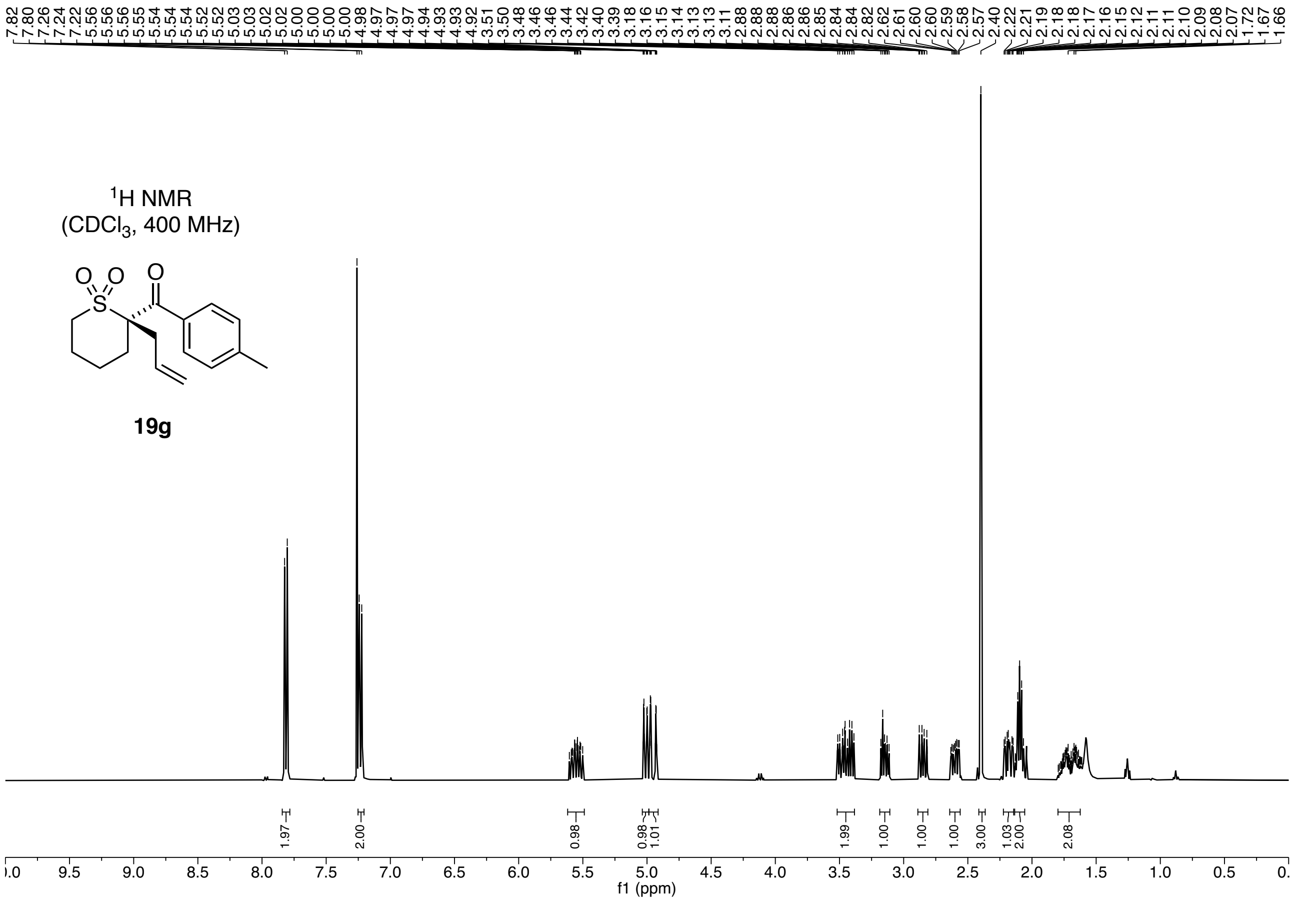
1H NMR
(CDCl3, 400 MHz)
19g
f1 (ppm)

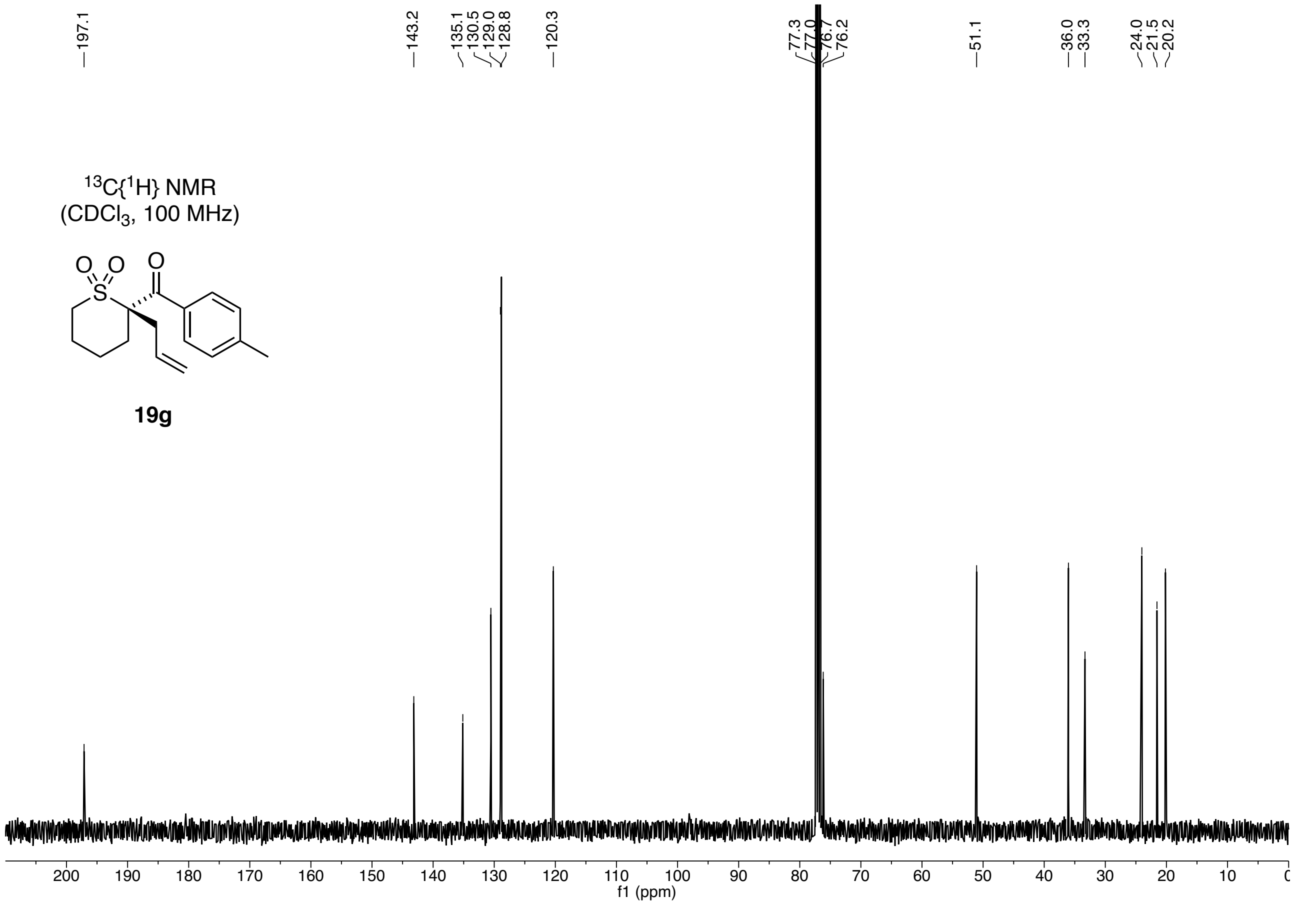
197.1
143.2
135.1
130.5
129.0
128.8
120.3
77.3
77.0
76.7
76.2
51.1
36.0
33.3
24.0
21.5
20.2
13C{1H} NMR
(CDCl3, 100 MHz)
19g
f1 (ppm)

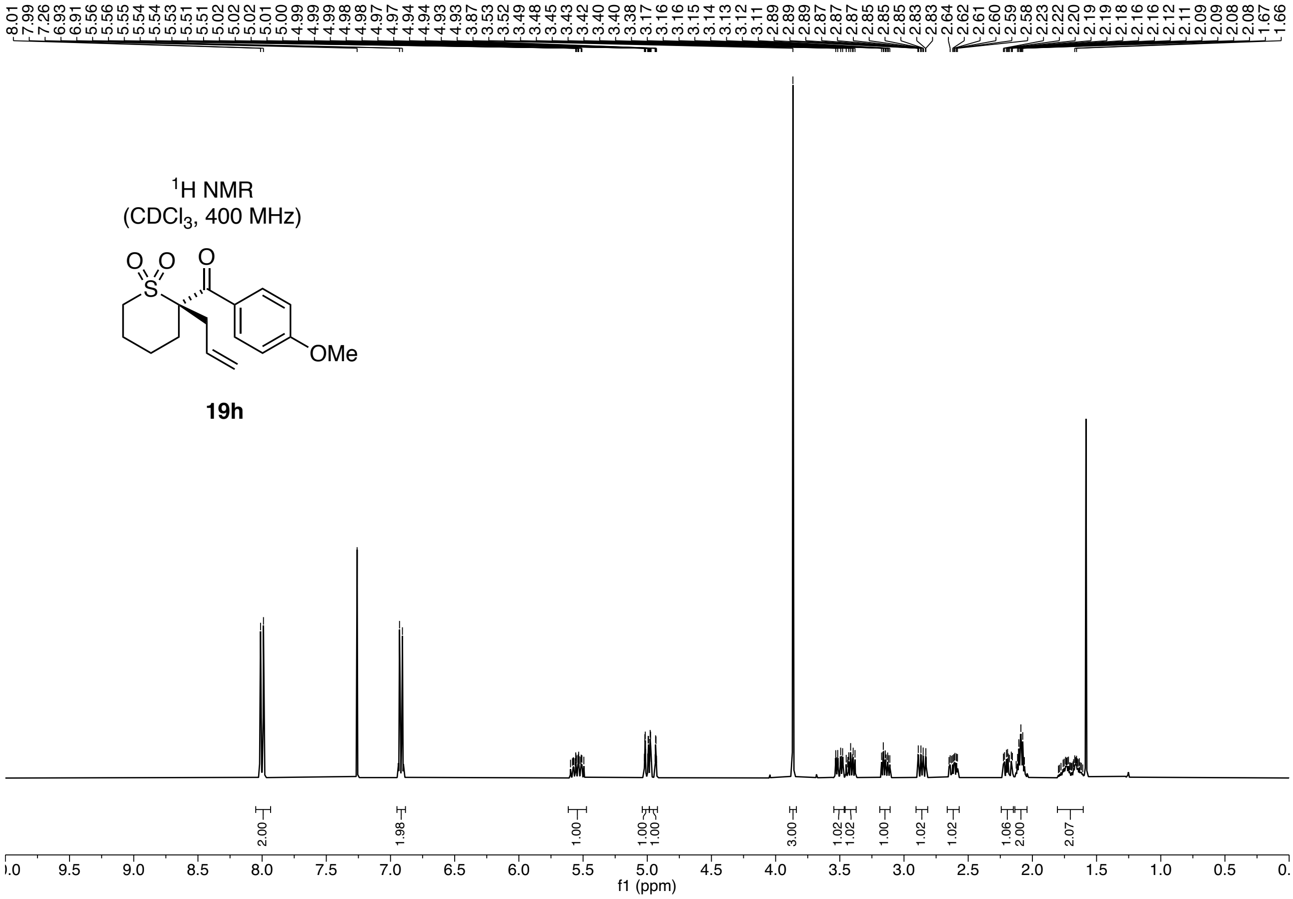
8.01 7.99 7.26 6.93 6.91 5.56 5.55 5.54 5.54 5.53 5.51 5.51 5.02 5.02 5.01 5.00 4.99 4.99 4.99 4.98 4.98 4.97 4.97 4.94 4.94 4.93 4.93 3.87 3.53 3.52 3.49 3.48 3.45 3.43 3.42 3.40 3.38 3.17 3.16 3.15 3.14 3.13 3.12 3.11 2.89 2.89 2.87 2.87 2.85 2.85 2.83 2.83 2.64 2.62 2.61 2.60 2.59 2.58 2.23 2.22 2.20 2.19 2.19 2.18 2.16 2.12 2.11 2.09 2.08 2.08 1.67 1.66
^{1}H NMR
($CDCl_3$, 400 MHz)
O O O
S
OMe
19h
2.00 1.98 1.00 1.00 1.00 3.00 1.02 1.02 1.00 1.02 1.02 1.06 2.00 2.07
f1 (ppm)

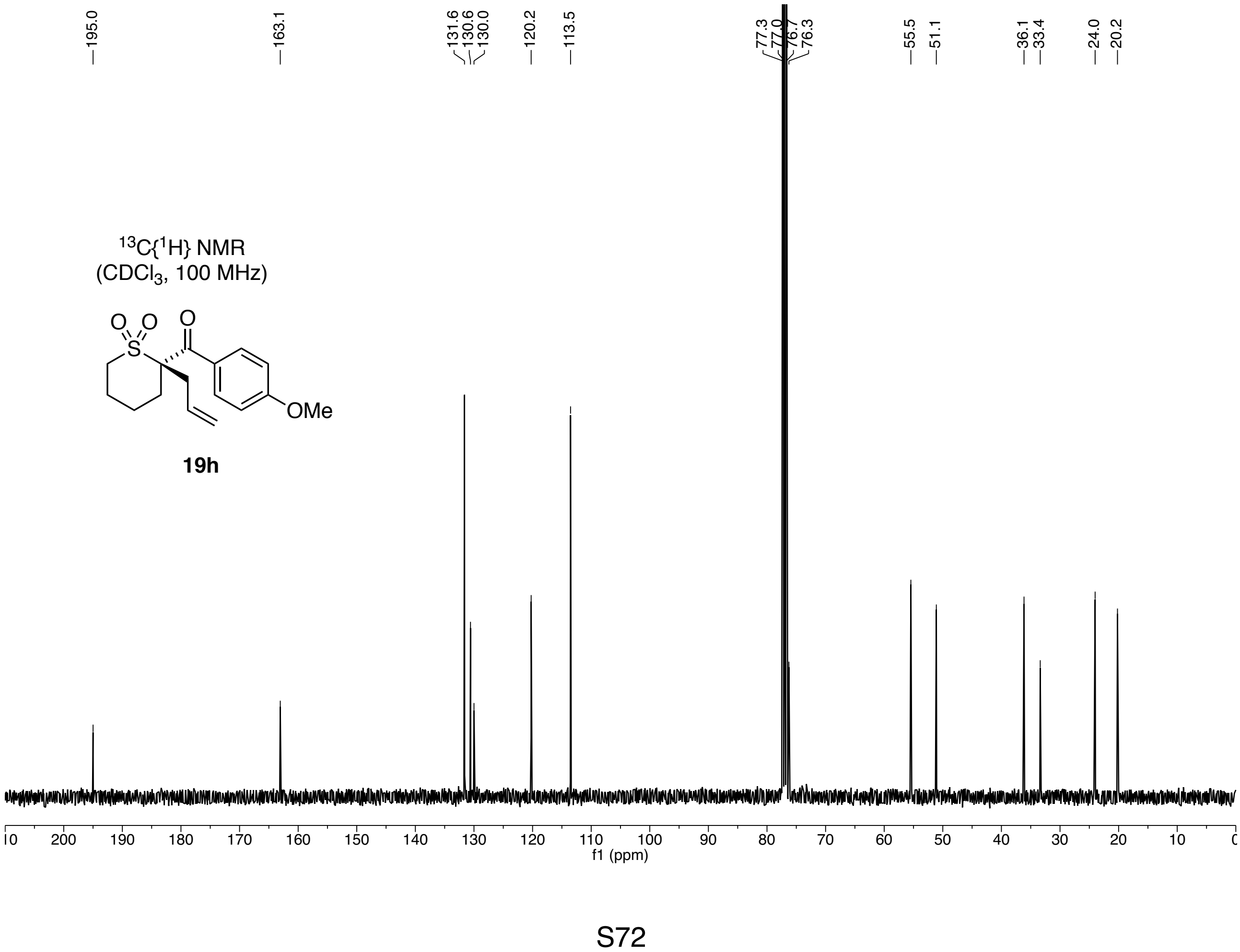
195.0 163.1 131.6 130.6 130.0 120.2 113.5 77.3 77.0 76.7 76.3 55.5 51.1 36.1 33.4 24.0 20.2
$^{13}C\{^1H\}$ NMR
($CDCl_3$, 100 MHz)
O O O
S
OMe
19h
f1 (ppm)

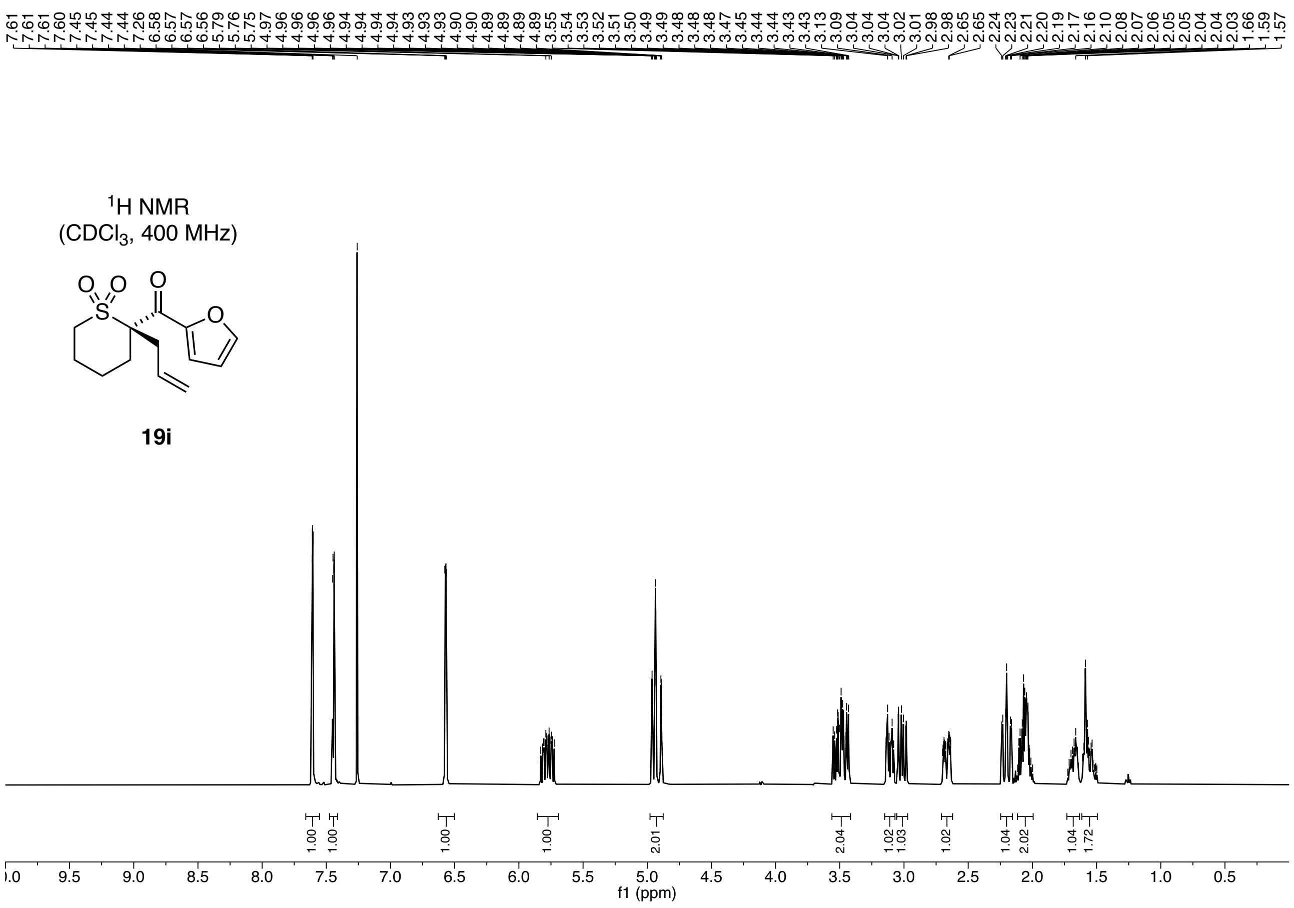
7.61 7.61 7.60 7.45 7.45 7.44 7.44 7.26 6.58 6.57 6.56 5.79 5.76 5.75 4.97 4.96 4.96 4.96 4.94 4.94 4.94 4.93 4.93 4.90 4.89 4.89 4.89 3.55 3.54 3.53 3.52 3.51 3.50 3.49 3.48 3.48 3.48 3.47 3.45 3.44 3.44 3.43 3.43 3.13 3.09 3.04 3.04 3.02 3.01 2.98 2.98 2.65 2.65 2.24 2.23 2.21 2.20 2.19 2.17 2.16 2.10 2.08 2.07 2.06 2.05 2.05 2.04 2.04 2.03 1.66 1.59 1.57
^{1}H NMR
($CDCl_3$, 400 MHz)
19i
1.00 1.00 1.00 1.00 2.01 2.04 1.02 1.03 1.02 1.04 2.02 1.04 1.72
f1 (ppm)

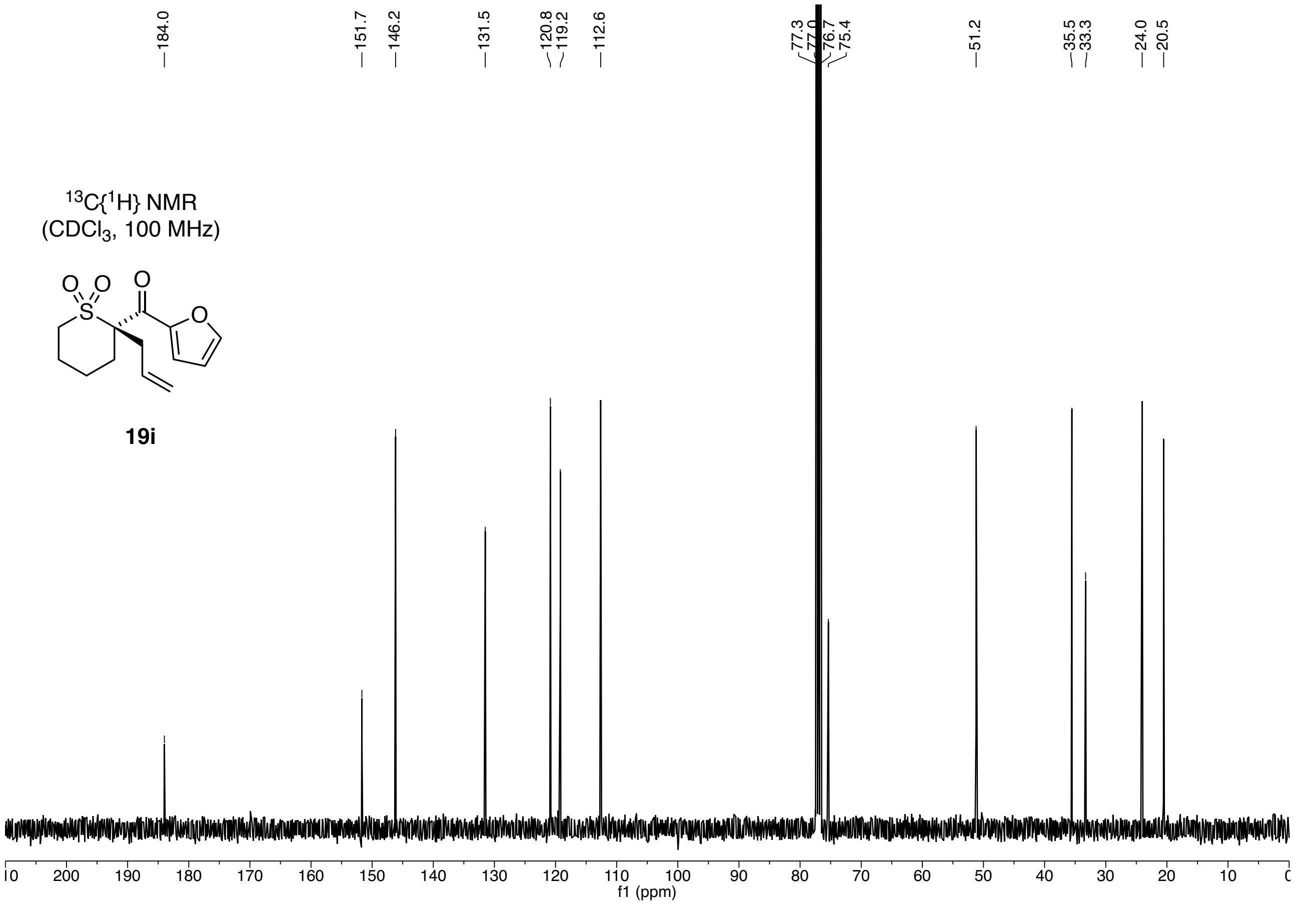
184.0 151.7 146.2 131.5 120.8 119.2 112.6 77.3 77.0 76.7 75.4 51.2 35.5 33.3 24.0 20.5
$^{13}C\{^{1}H\}$ NMR
($CDCl_3$, 100 MHz)
19i
f1 (ppm)

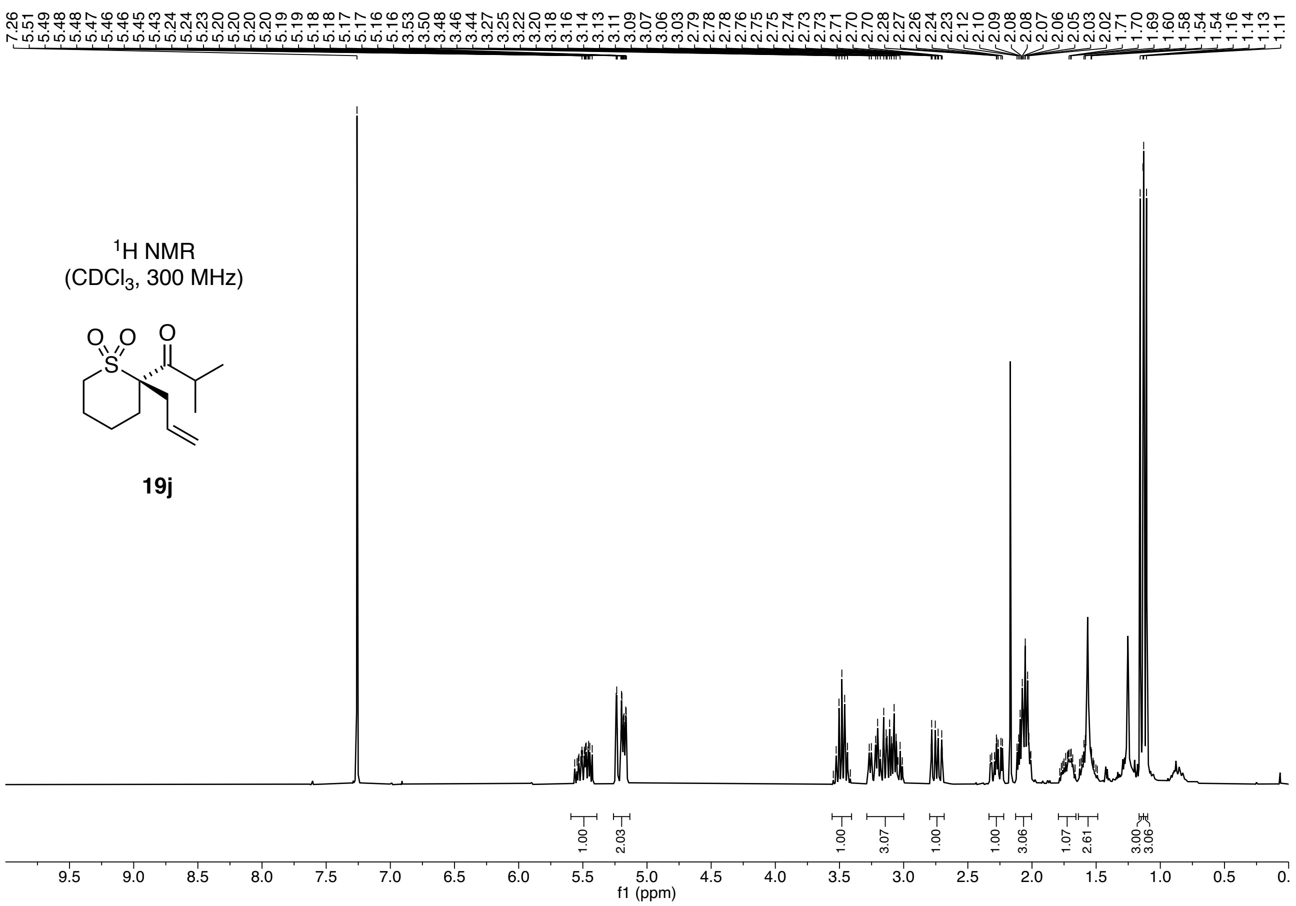

^{1}H NMR
(CDCl$_3$, 300 MHz)
19j
f1 (ppm)

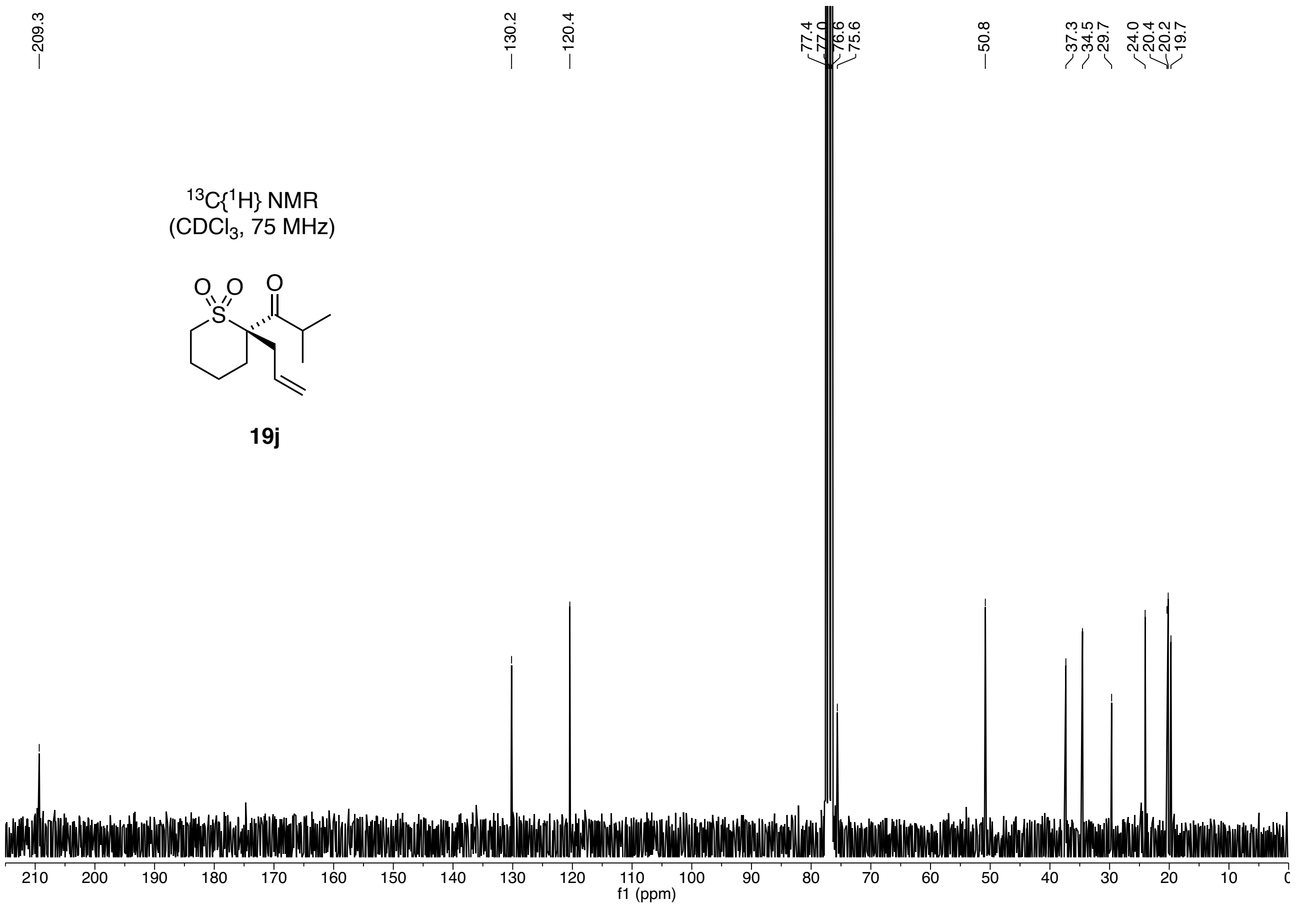

^{13}C{^{1}H} NMR
(CDCl$_3$, 75 MHz)
19j
209.3
130.2
120.4
77.4
77.0
76.6
75.6
50.8
37.3
34.5
29.7
24.0
20.4
20.2
19.7
f1 (ppm)

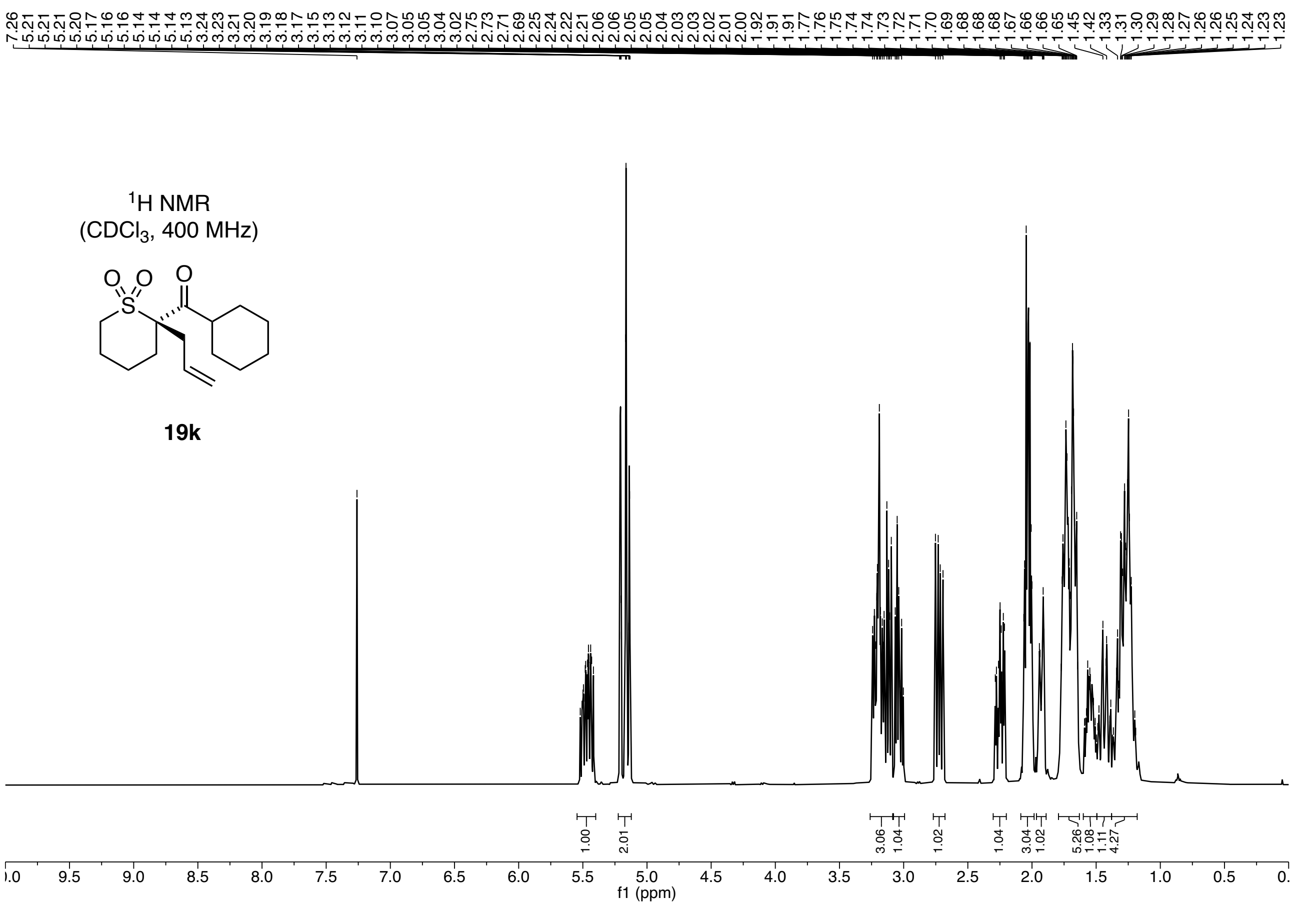
7.26 5.21 5.21 5.21 5.20 5.17 5.16 5.16 5.14 5.14 5.13 3.24 3.23 3.21 3.20 3.19 3.18 3.17 3.15 3.13 3.12 3.11 3.10 3.07 3.05 3.05 3.04 3.02 2.75 2.73 2.71 2.69 2.25 2.24 2.22 2.21 2.06 2.05 2.05 2.04 2.03 2.03 2.02 2.01 2.00 1.92 1.91 1.91 1.77 1.76 1.75 1.74 1.73 1.72 1.71 1.70 1.69 1.68 1.68 1.67 1.66 1.65 1.45 1.42 1.33 1.31 1.30 1.29 1.28 1.27 1.26 1.25 1.24 1.23 1.23
^{1}H NMR
($CDCl_3$, 400 MHz)
19k
1.00 2.01 3.06 1.04 1.02 1.04 3.04 1.02 5.26 1.08 1.11 4.27
f1 (ppm)

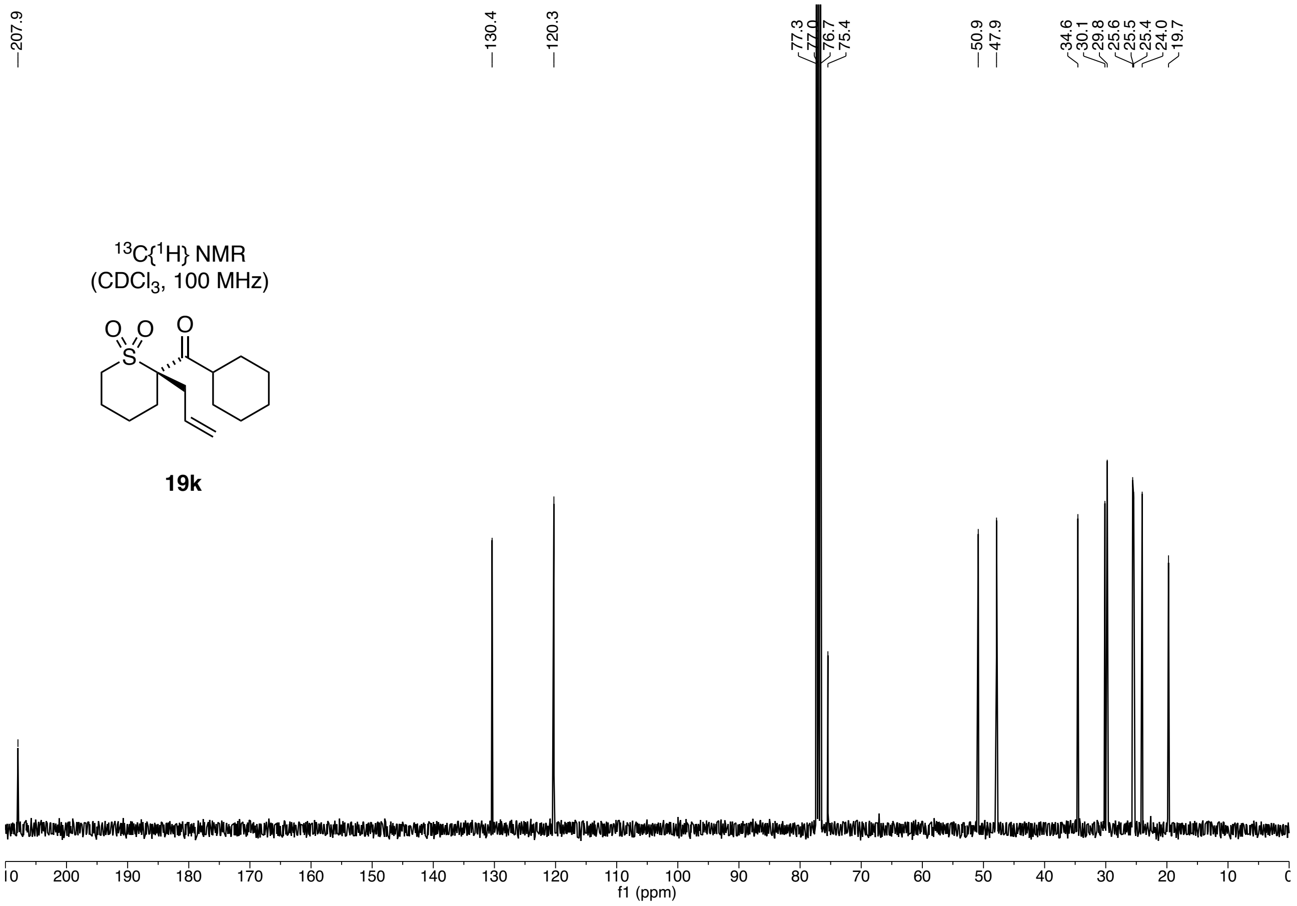
207.9 130.4 120.3 77.3 77.0 76.7 75.4 50.9 47.9 34.6 30.1 29.8 25.6 25.5 25.4 24.0 19.7
$^{13}C\{^1H\}$ NMR
($CDCl_3$, 100 MHz)
19k
f1 (ppm)

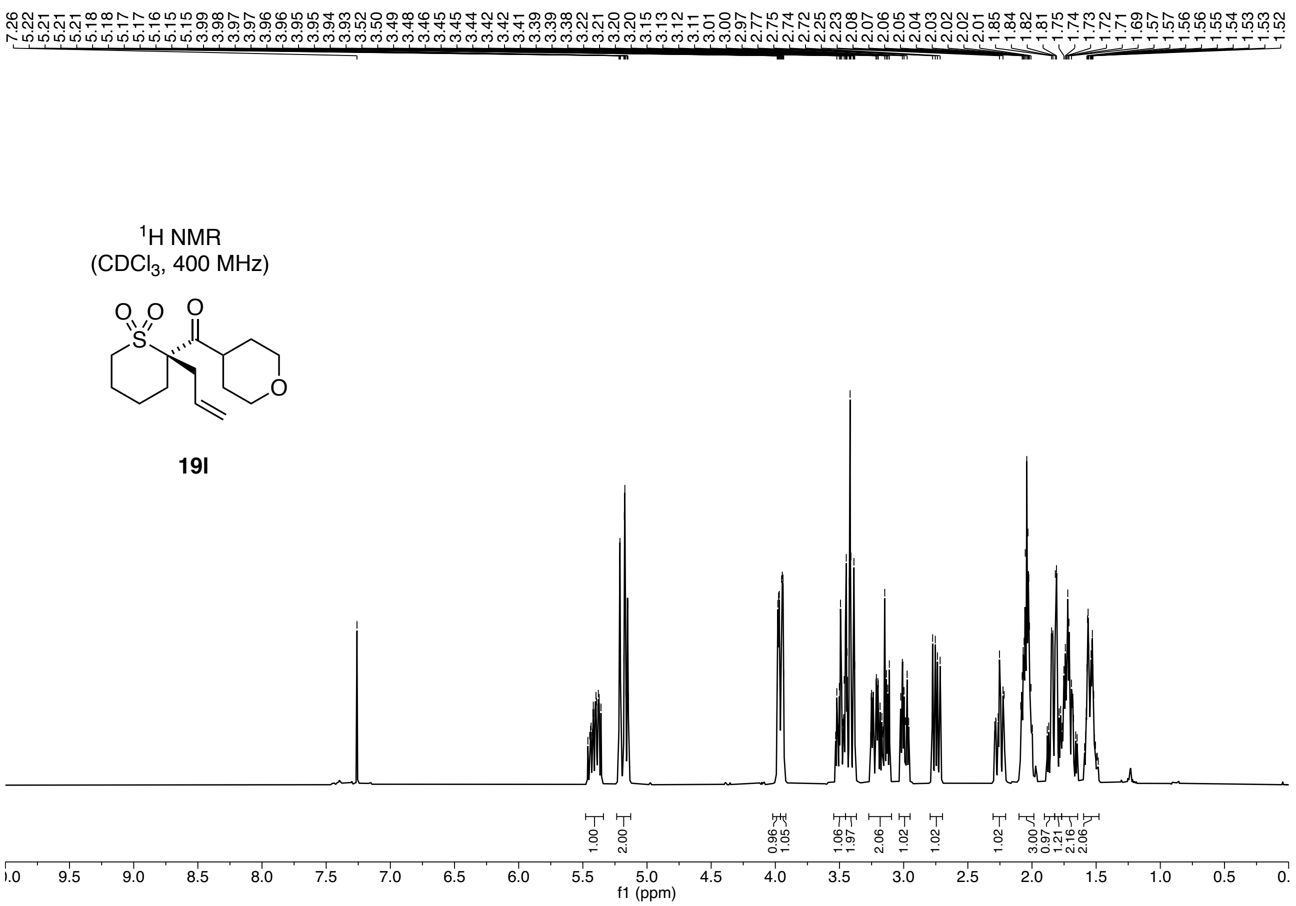
1H NMR
(CDCl3, 400 MHz)
19l
7.26 5.22 5.21 5.21 5.18 5.17 5.16 5.15 3.99 3.98 3.97 3.97 3.96 3.96 3.95 3.94 3.93 3.52 3.50 3.49 3.48 3.46 3.45 3.45 3.44 3.42 3.42 3.41 3.39 3.39 3.38 3.22 3.21 3.20 3.20 3.15 3.13 3.12 3.11 3.01 3.00 2.97 2.77 2.75 2.74 2.72 2.25 2.23 2.08 2.07 2.06 2.05 2.04 2.03 2.02 2.02 2.01 1.85 1.84 1.82 1.81 1.75 1.74 1.73 1.72 1.71 1.69 1.57 1.57 1.56 1.55 1.54 1.53 1.53 1.52
1.00 2.00 0.96 1.05 1.06 1.97 2.06 1.02 1.02 1.02 3.00 0.97 1.21 2.16 2.06
f1 (ppm)

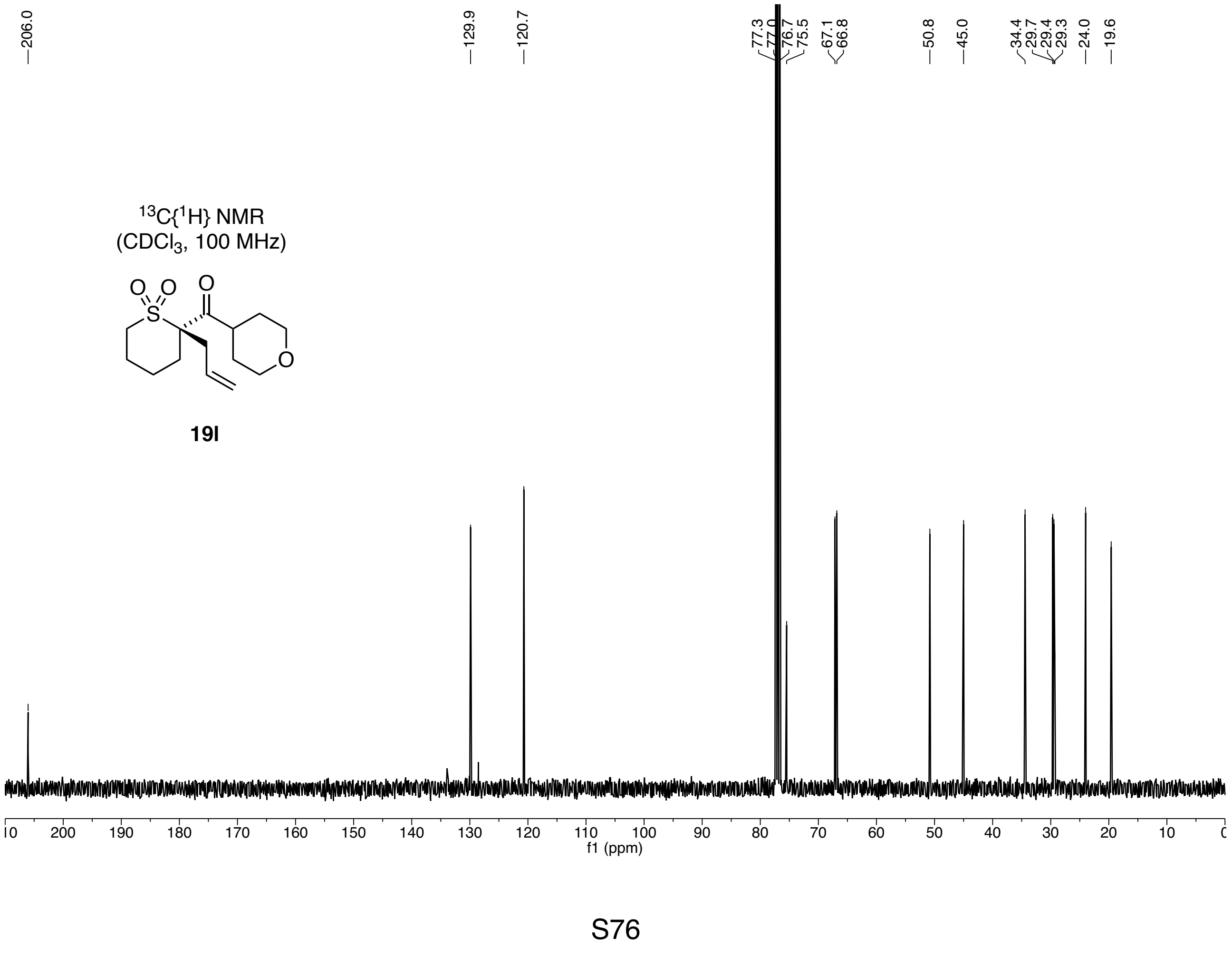
13C{1H} NMR
(CDCl3, 100 MHz)
19l
206.0 129.9 120.7 77.3 77.0 76.7 75.5 67.1 66.8 50.8 45.0 34.4 29.7 29.4 29.3 24.0 19.6
f1 (ppm)

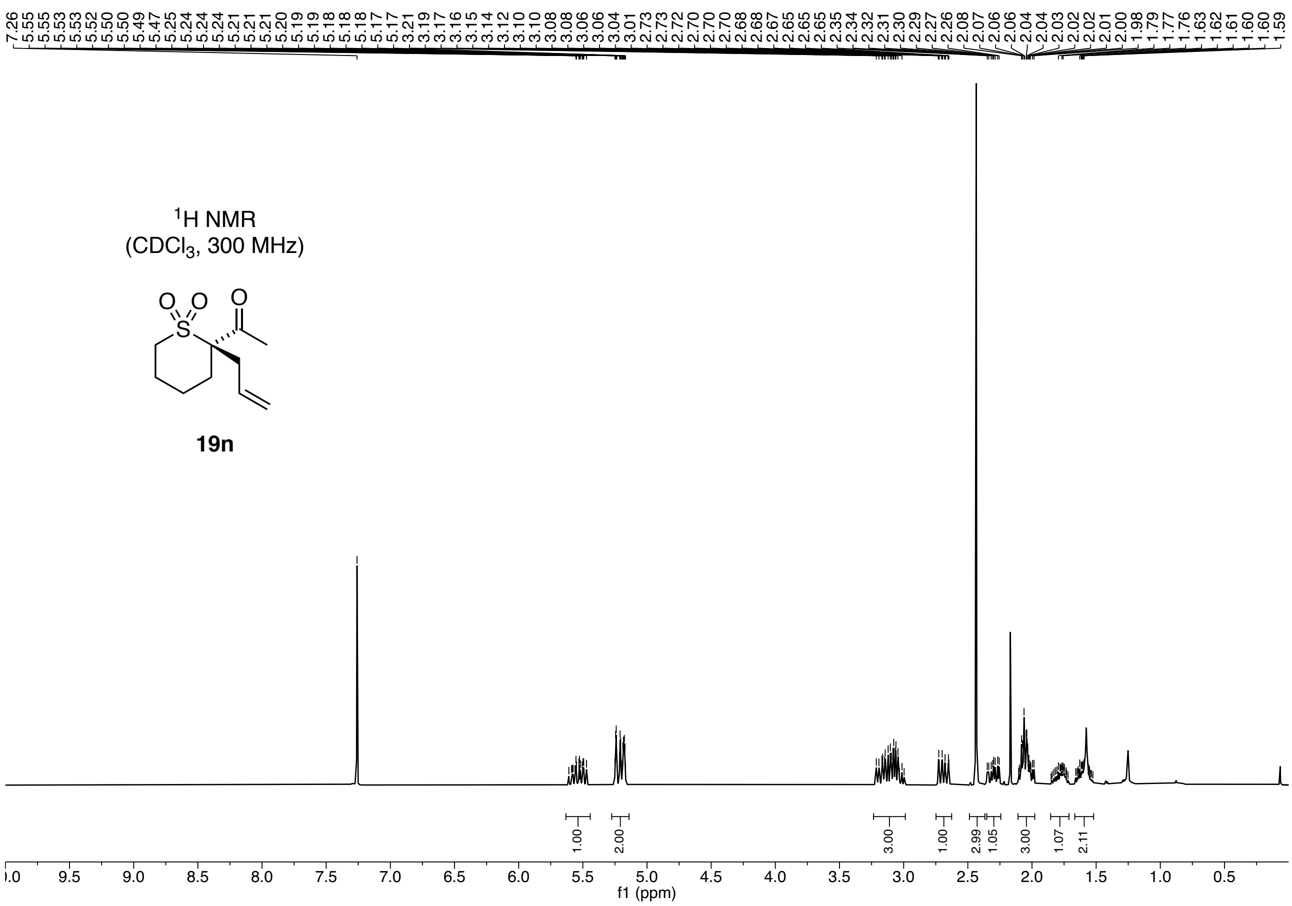

7.26 5.55 5.55 5.53 5.52 5.50 5.50 5.49 5.47 5.25 5.24 5.24 5.21 5.21 5.20 5.19 5.18 5.18 5.17 3.21 3.19 3.17 3.16 3.15 3.14 3.12 3.10 3.08 3.08 3.06 3.06 3.04 3.01 2.73 2.73 2.72 2.70 2.70 2.70 2.68 2.68 2.67 2.65 2.65 2.35 2.34 2.32 2.31 2.30 2.29 2.27 2.26 2.08 2.07 2.06 2.06 2.04 2.04 2.03 2.02 2.02 2.01 2.00 1.98 1.79 1.77 1.76 1.63 1.62 1.61 1.60 1.59
1H NMR
(CDCl3, 300 MHz)
19n
1.00
2.00
3.00
1.00
2.99
1.05
3.00
1.07
2.11
f1 (ppm)

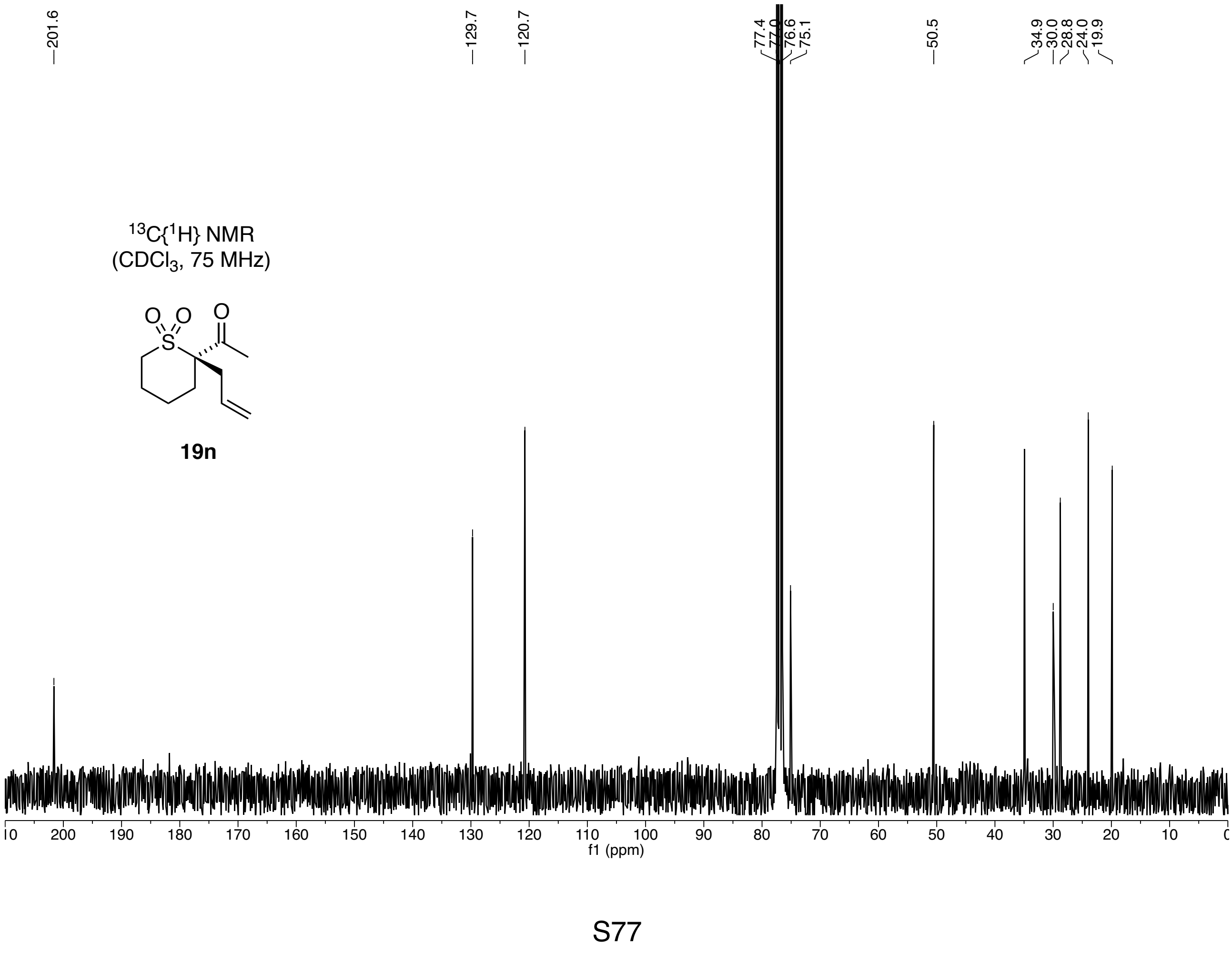

201.6
129.7
120.7
77.4 77.0 76.6 75.1
50.5
34.9 30.0 28.8 24.0 19.9
13C{1H} NMR
(CDCl3, 75 MHz)
19n
f1 (ppm)

^{1}H NMR
(DMSO-d_6, 130 °C, 400 MHz)

20a

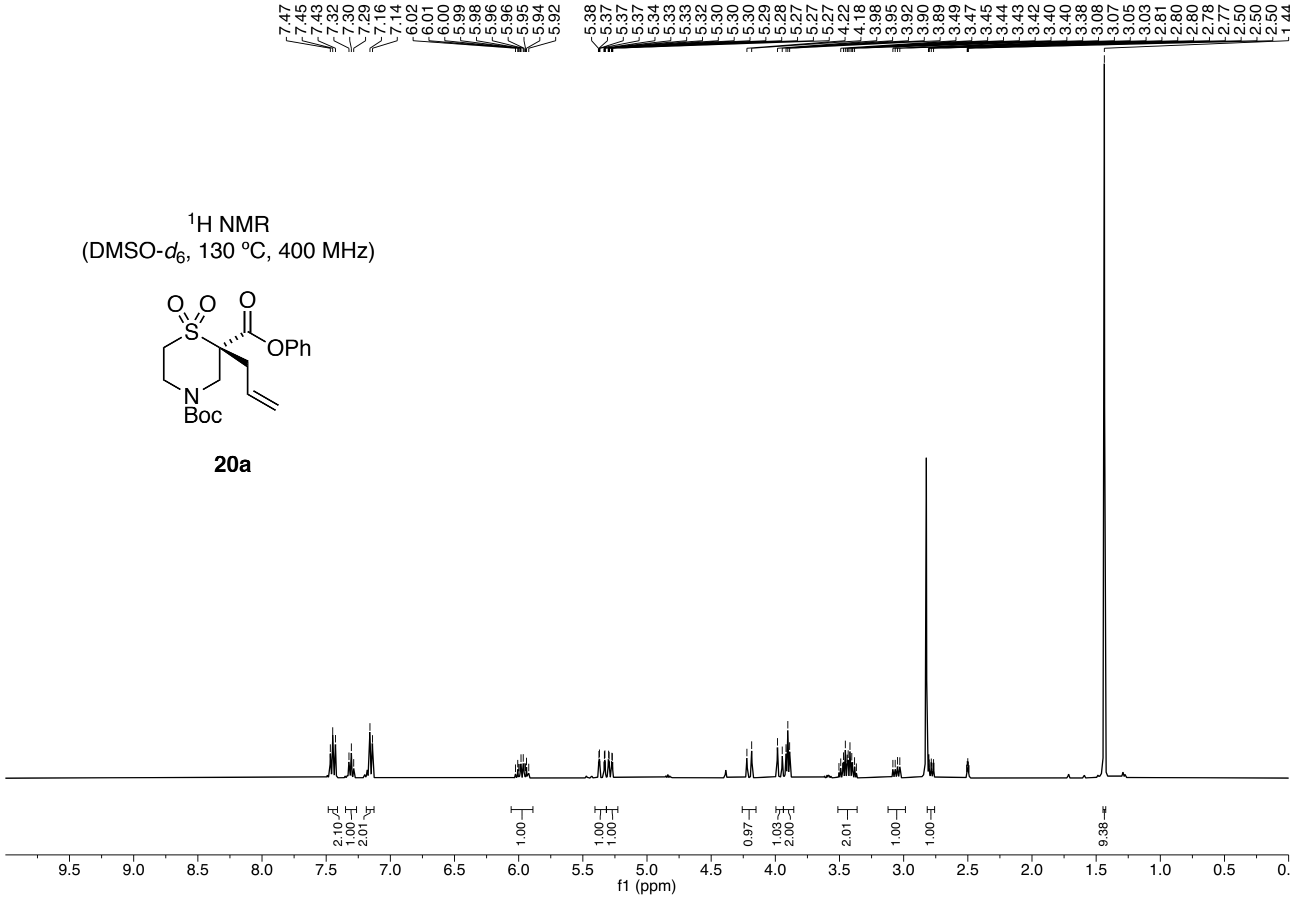

^{13}C{^{1}H} NMR
(DMSO-d_6, 130 °C, 100 MHz)

20a

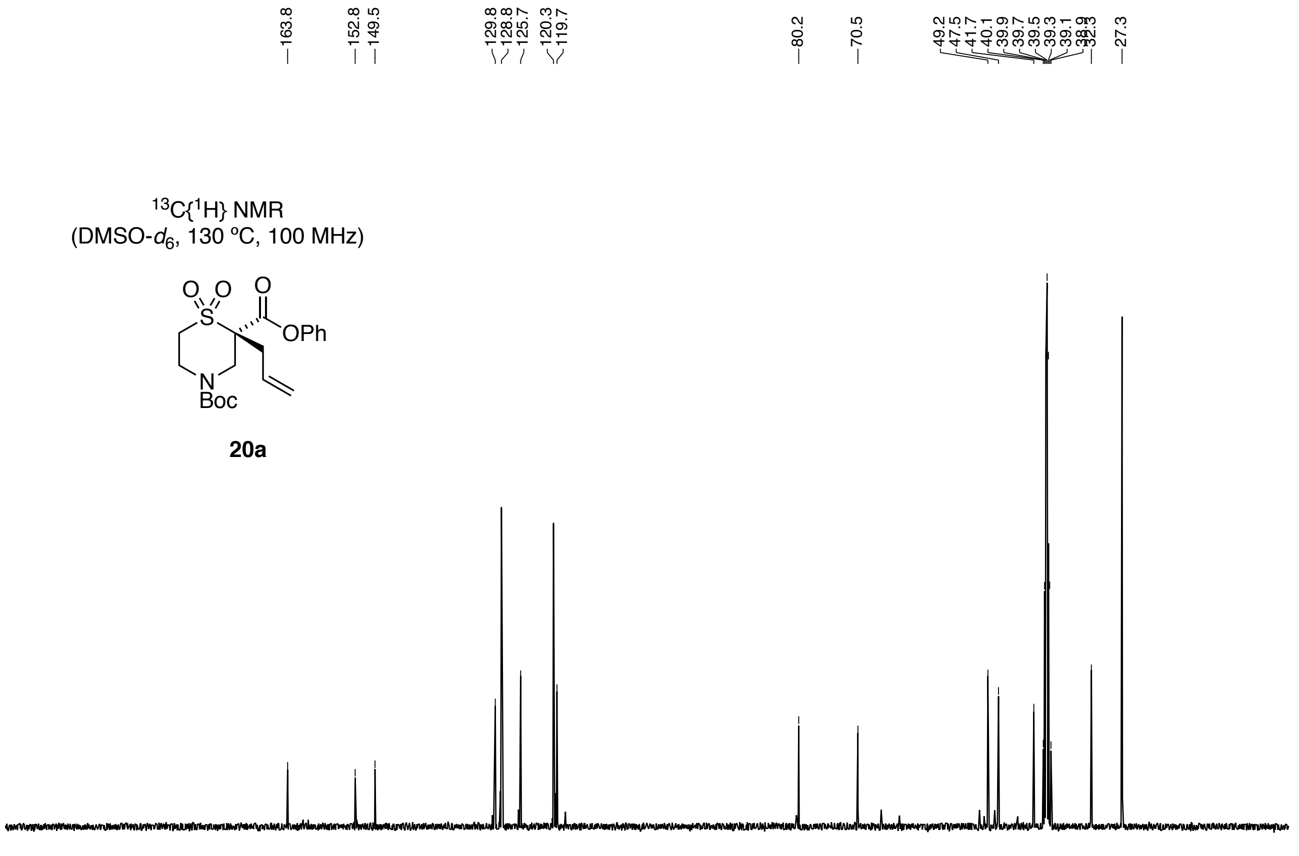

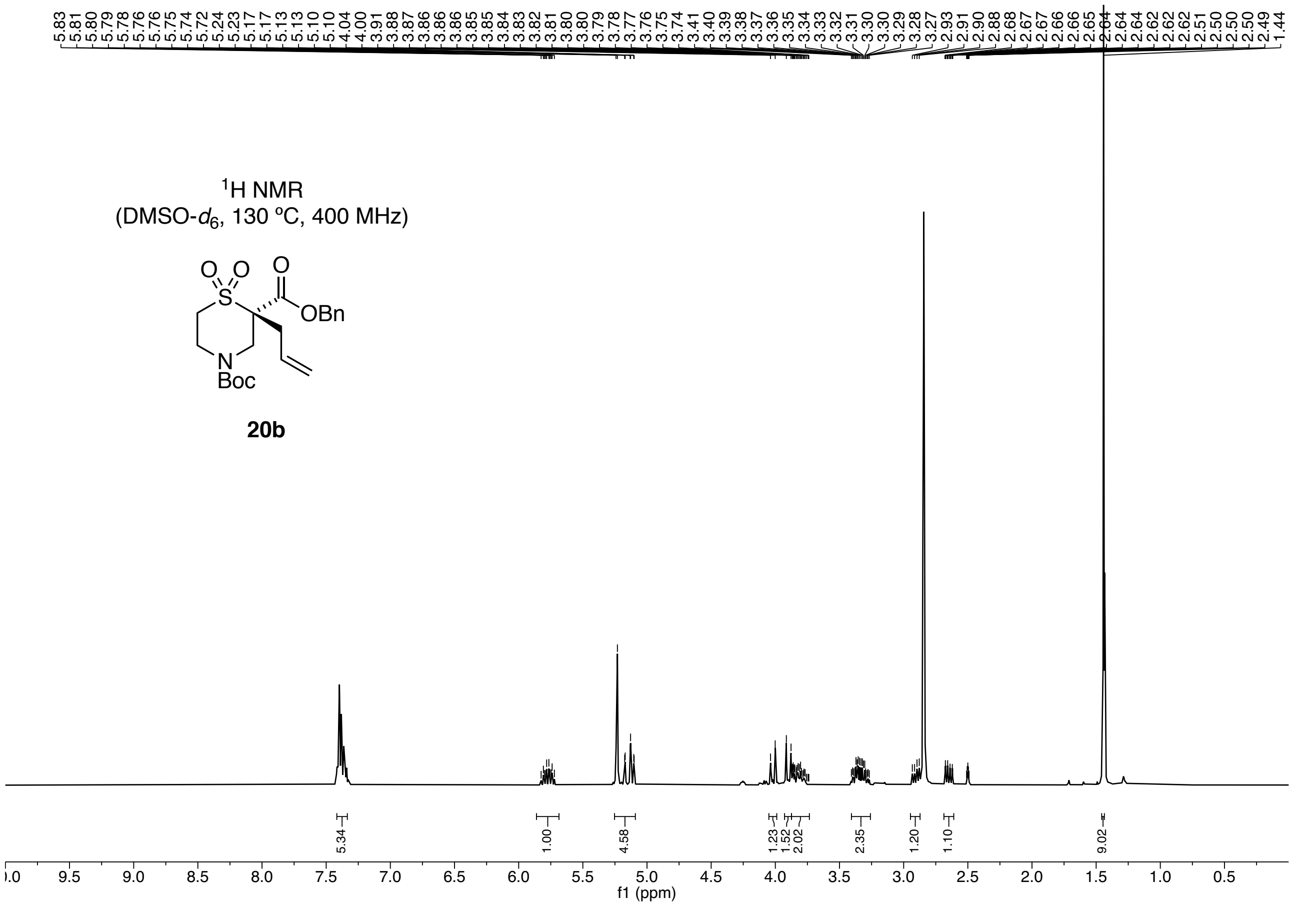
5.83 5.81 5.80 5.79 5.78 5.76 5.76 5.75 5.74 5.72 5.24 5.23 5.17 5.17 5.13 5.13 5.10 5.10 4.04 4.00 3.91 3.88 3.87 3.86 3.86 3.85 3.85 3.84 3.83 3.82 3.81 3.80 3.80 3.79 3.78 3.77 3.76 3.75 3.74 3.41 3.40 3.39 3.38 3.37 3.36 3.35 3.34 3.33 3.32 3.31 3.30 3.30 3.29 3.28 3.27 2.93 2.91 2.90 2.88 2.68 2.67 2.67 2.66 2.66 2.65 2.64 2.64 2.64 2.62 2.62 2.62 2.51 2.50 2.50 2.49 1.44
^{1}H NMR
(DMSO-d_6, 130 °C, 400 MHz)
O O O
S
OBn
N
Boc
20b
5.34
1.00
4.58
1.23
1.52
2.02
2.35
1.20
1.10
9.02
f1 (ppm)

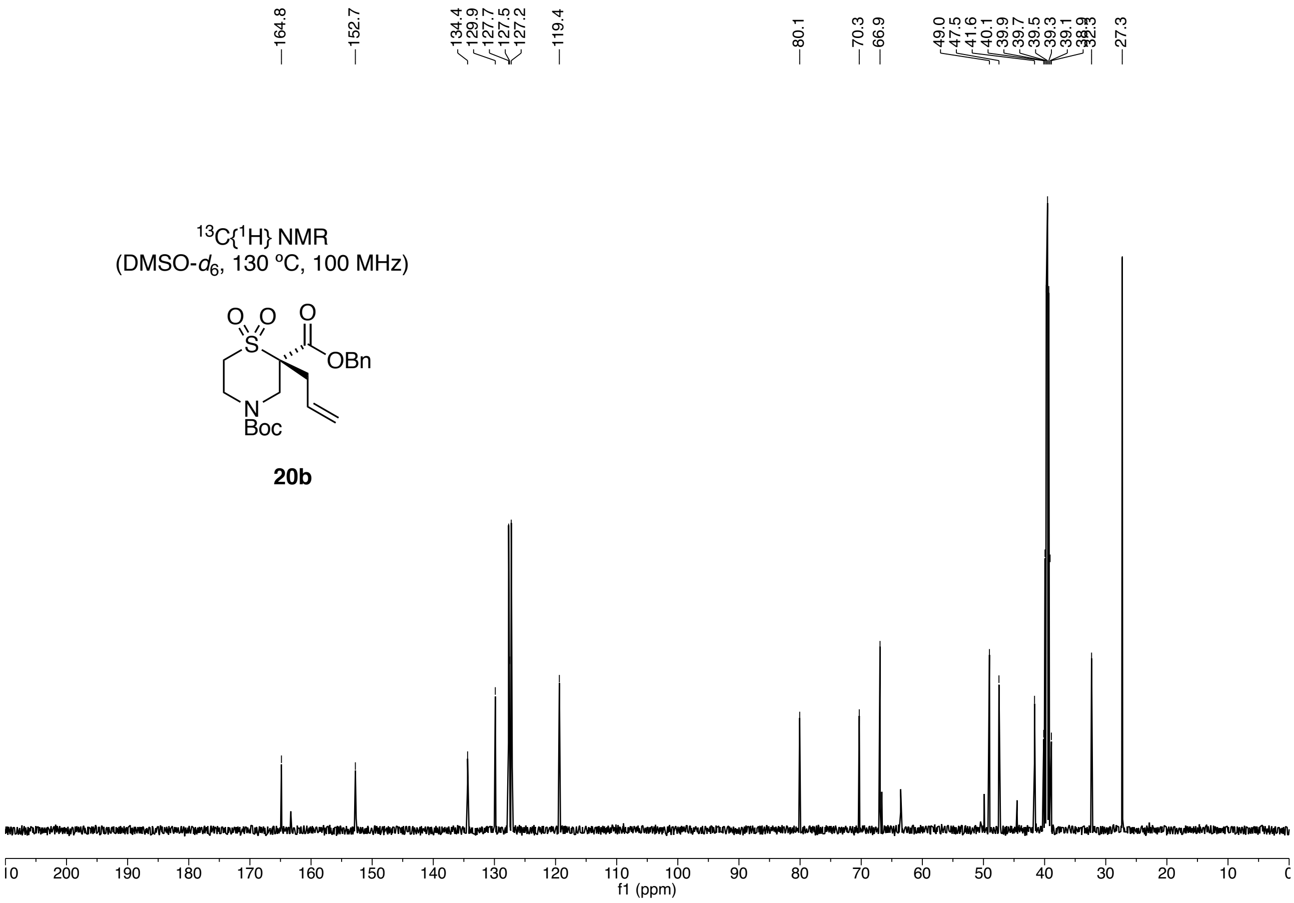
164.8
152.7
134.4 129.9 127.7 127.5 127.2
119.4
80.1
70.3
66.9
49.0 47.5 41.6 40.1 39.9 39.7 39.5 39.3 39.1 38.9 32.3
27.3
^{13}C{^{1}H} NMR
(DMSO-d_6, 130 °C, 100 MHz)
O O O
S
OBn
N
Boc
20b
f1 (ppm)

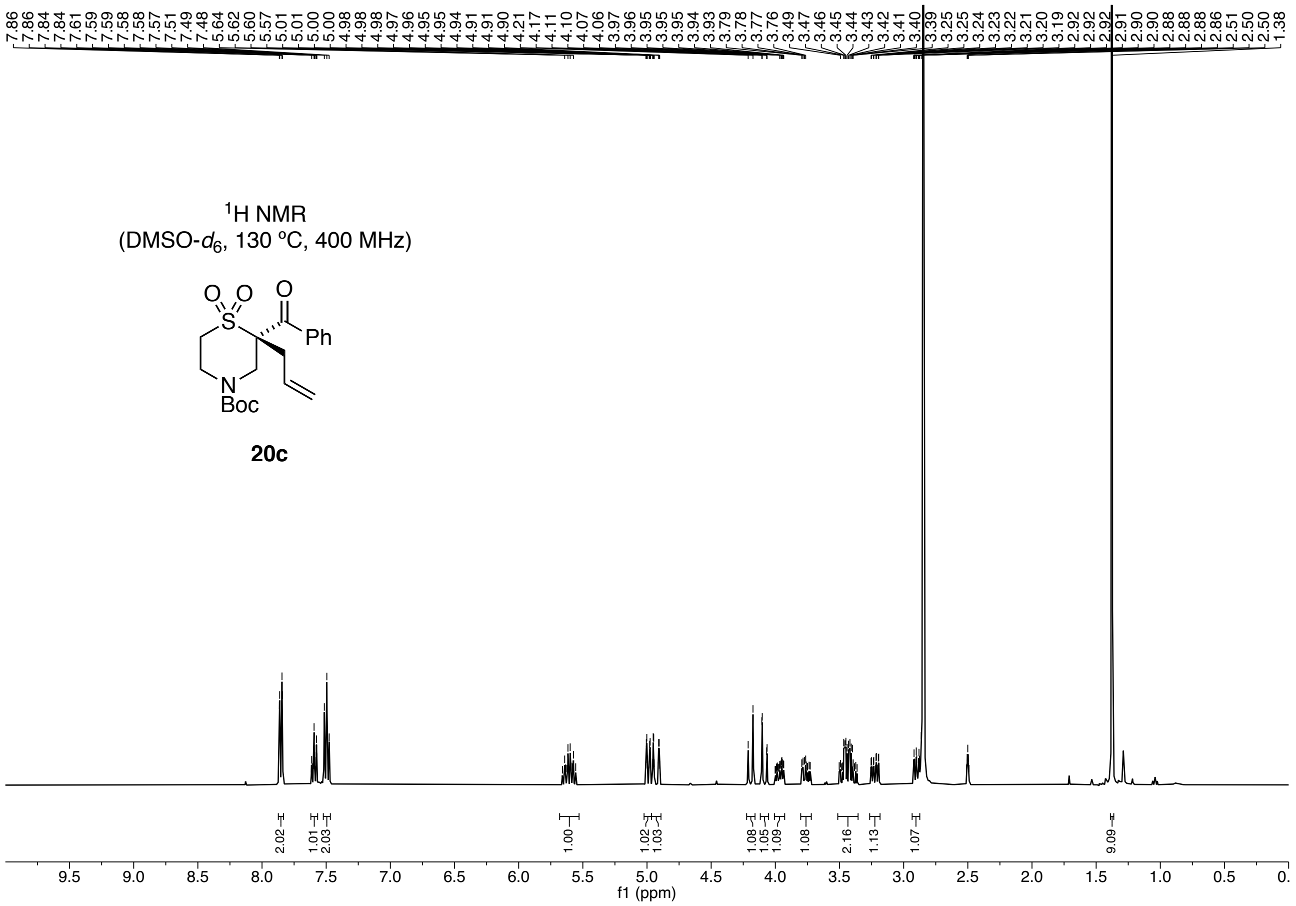
7.86 7.86 7.84 7.61 7.59 7.59 7.58 7.57 7.51 7.49 7.48 5.64 5.62 5.60 5.57 5.01 5.00 5.00 4.98 4.98 4.97 4.96 4.95 4.95 4.94 4.91 4.91 4.90 4.21 4.17 4.11 4.10 4.07 4.06 3.97 3.96 3.95 3.95 3.94 3.93 3.79 3.78 3.77 3.76 3.49 3.47 3.46 3.45 3.44 3.43 3.42 3.41 3.40 3.39 3.25 3.25 3.24 3.23 3.22 3.21 3.20 3.19 2.92 2.92 2.92 2.91 2.90 2.88 2.88 2.86 2.51 2.50 2.50 1.38
1H NMR
(DMSO-d6, 130 °C, 400 MHz)
O O O
S
Ph
N
Boc
20c
2.02 1.01 2.03 1.00 1.02 1.03 1.08 1.05 1.09 1.08 2.16 1.13 1.07 9.09
9.5 9.0 8.5 8.0 7.5 7.0 6.5 6.0 5.5 5.0 4.5 4.0 3.5 3.0 2.5 2.0 1.5 1.0 0.5 0.
f1 (ppm)

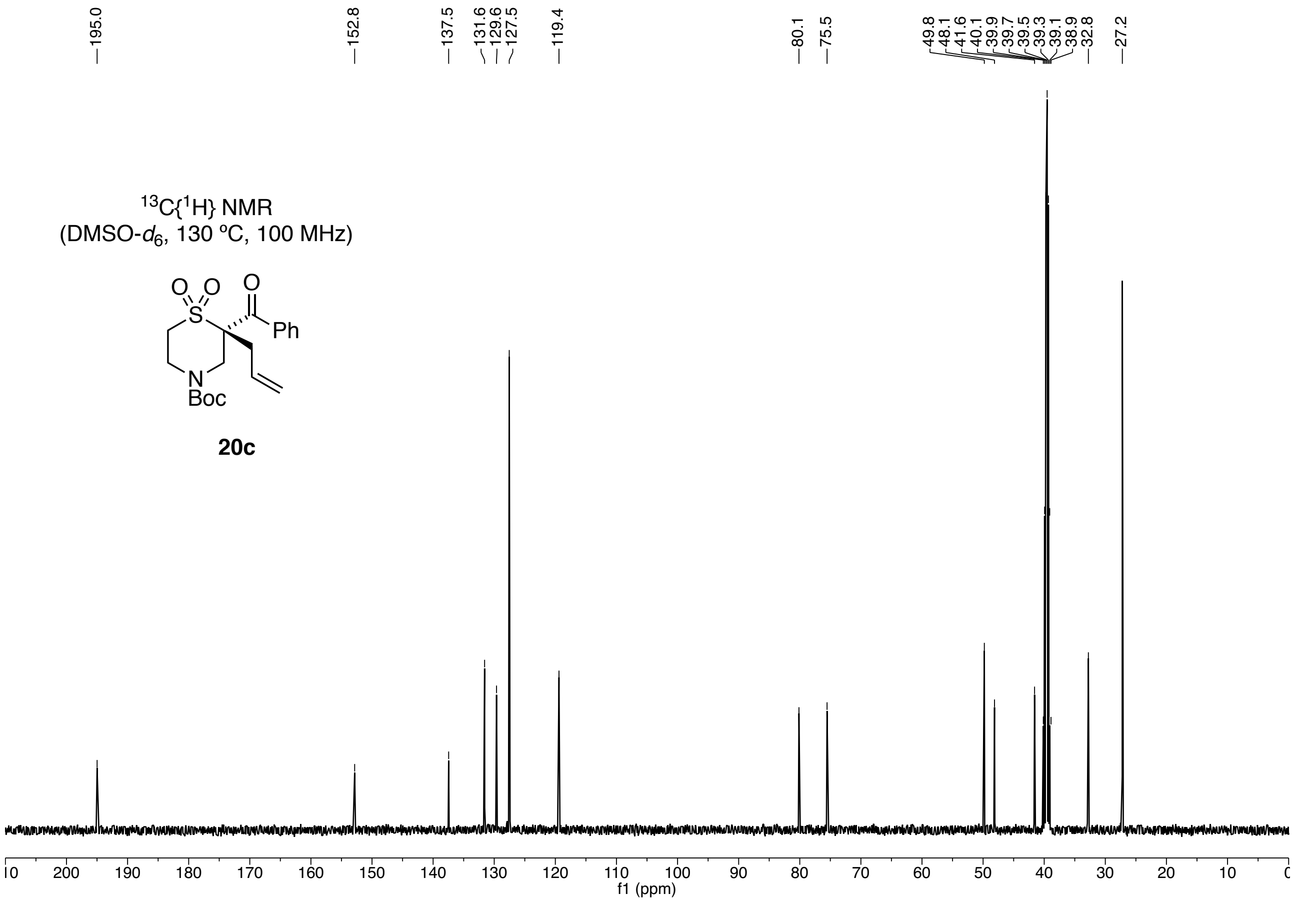
195.0 152.8 137.5 131.6 129.6 127.5 119.4 80.1 75.5 49.8 48.1 41.6 40.1 39.9 39.7 39.5 39.3 39.1 38.9 32.8 27.2
13C{1H} NMR
(DMSO-d6, 130 °C, 100 MHz)
O O O
S
Ph
N
Boc
20c
10 200 190 180 170 160 150 140 130 120 110 100 90 80 70 60 50 40 30 20 10 0
f1 (ppm)

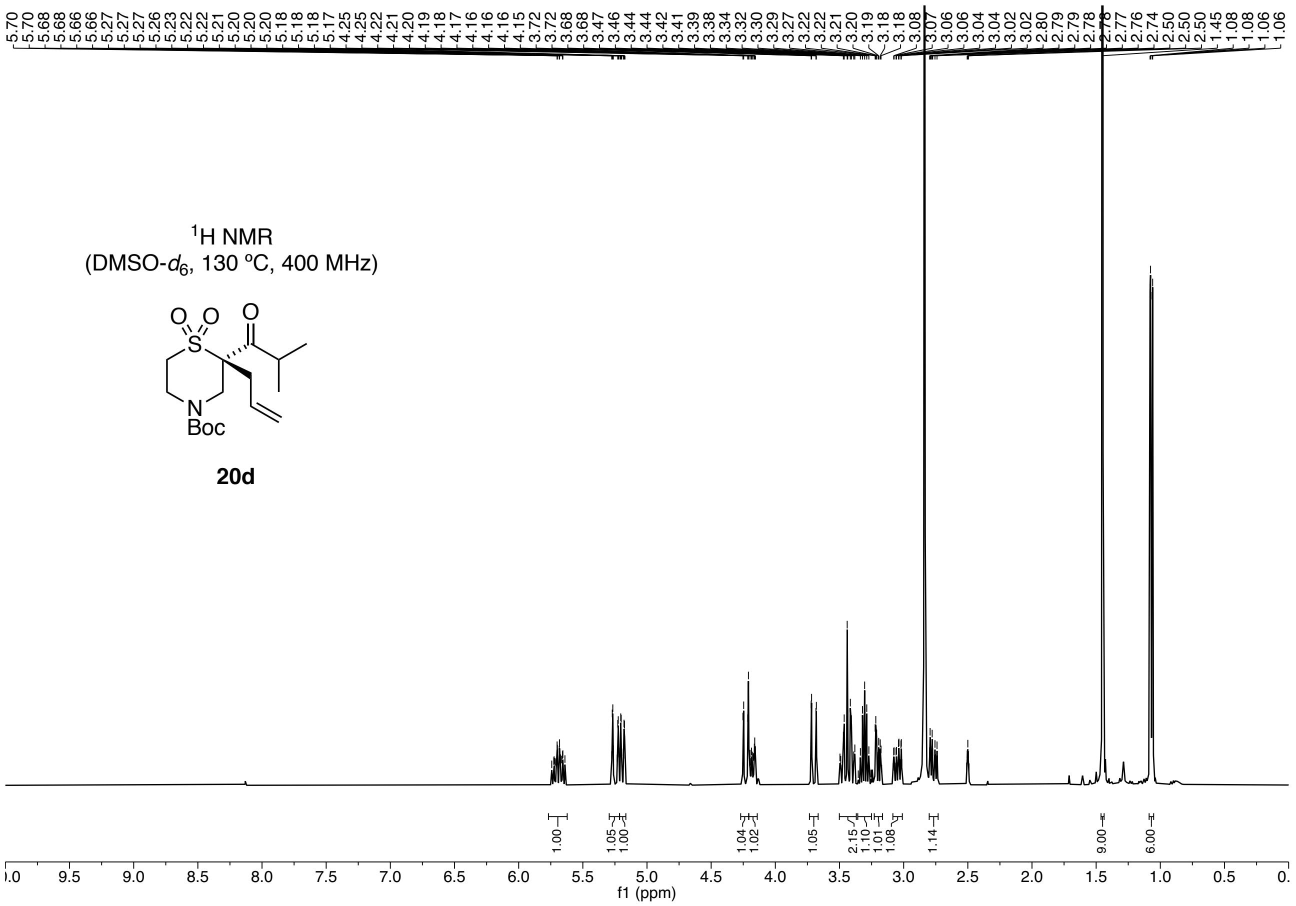
5.70 5.70 5.68 5.68 5.66 5.27 5.27 5.27 5.26 5.23 5.22 5.22 5.21 5.20 5.20 5.20 5.18 5.18 5.17 4.25 4.25 4.22 4.21 4.20 4.19 4.18 4.17 4.16 4.16 4.15 3.72 3.68 3.68 3.47 3.46 3.44 3.44 3.42 3.41 3.39 3.38 3.34 3.32 3.30 3.29 3.27 3.22 3.22 3.21 3.20 3.19 3.18 3.18 3.08 3.07 3.06 3.06 3.04 3.04 3.02 3.02 2.80 2.79 2.79 2.78 2.78 2.77 2.76 2.74 2.50 2.50 2.50 1.45 1.08 1.08 1.06 1.06
1H NMR
(DMSO-d6, 130 °C, 400 MHz)
O O O
S
N
Boc
20d
1.00 1.05 1.00 1.04 1.02 1.05 2.15 1.10 1.01 1.08 1.14 9.00 6.00
9.5 9.0 8.5 8.0 7.5 7.0 6.5 6.0 5.5 5.0 4.5 4.0 3.5 3.0 2.5 2.0 1.5 1.0 0.5
f1 (ppm)

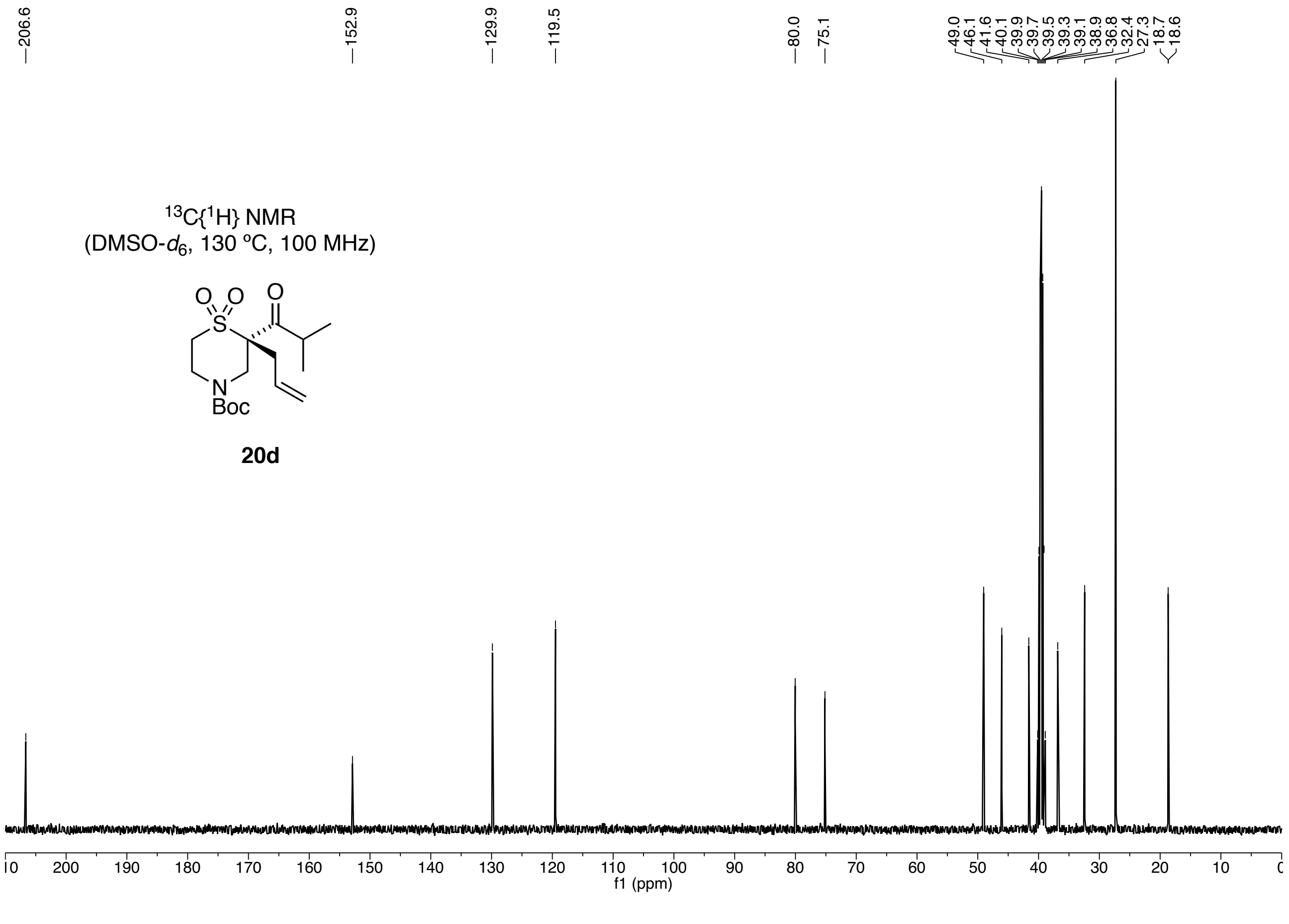
206.6 152.9 129.9 119.5 80.0 75.1 49.0 46.1 41.6 40.1 39.9 39.7 39.5 39.3 39.1 38.9 36.8 32.4 27.3 18.7 18.6
13C{1H} NMR
(DMSO-d6, 130 °C, 100 MHz)
O O O
S
N
Boc
20d
200 190 180 170 160 150 140 130 120 110 100 90 80 70 60 50 40 30 20 10
f1 (ppm)

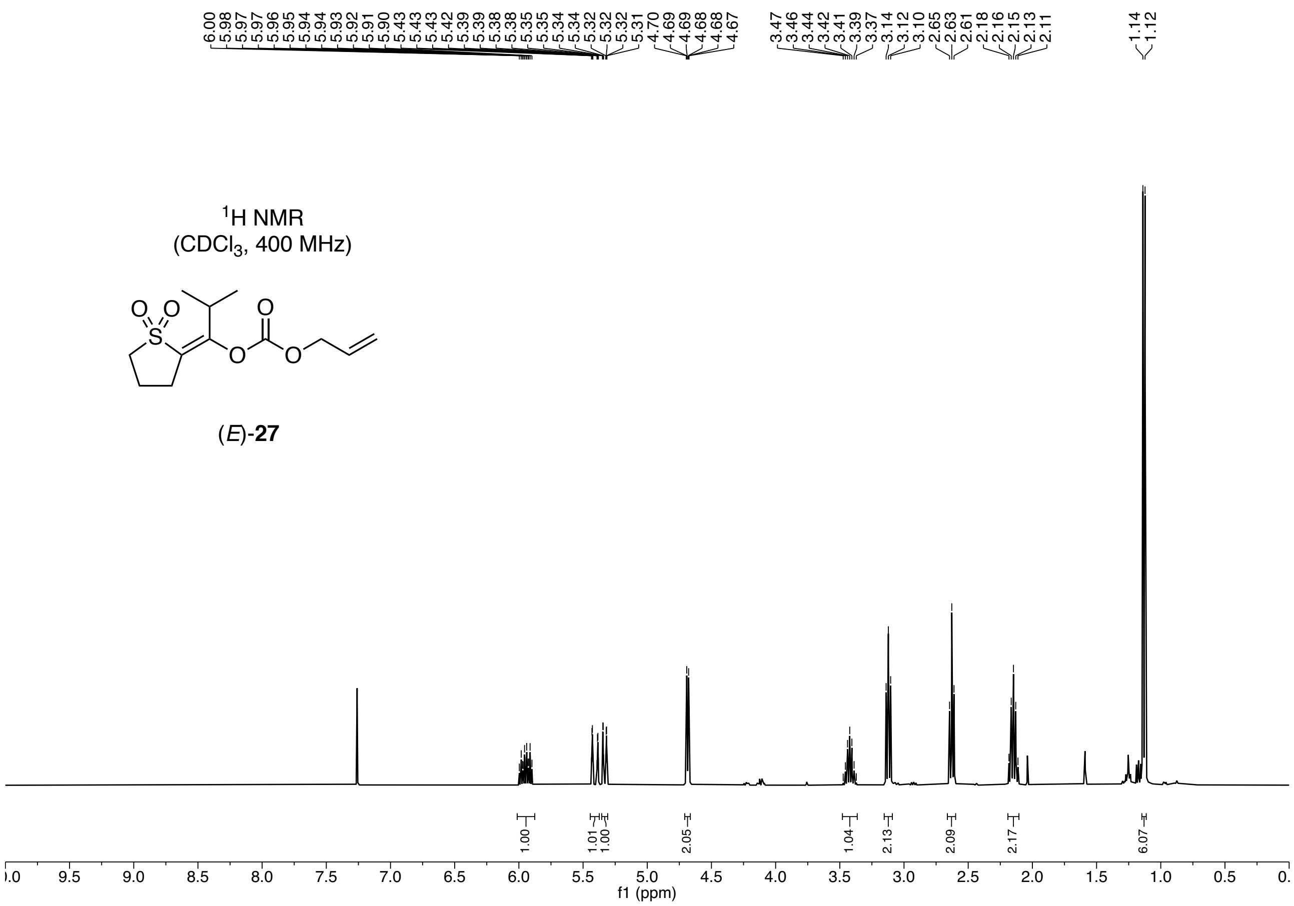
6.00 5.98 5.97 5.96 5.95 5.94 5.94 5.93 5.92 5.91 5.90 5.43 5.43 5.42 5.39 5.39 5.38 5.38 5.35 5.34 5.32 5.32 5.32 5.31 4.70 4.69 4.69 4.68 4.68 4.67
3.47 3.46 3.44 3.42 3.41 3.39 3.37 3.14 3.12 3.10 2.65 2.63 2.61 2.18 2.16 2.15 2.13 2.11
1.14 1.12
1H NMR
(CDCl3, 400 MHz)
(E)-27
1.00 1.01 1.00 2.05 1.04 2.13 2.09 2.17 6.07
f1 (ppm)

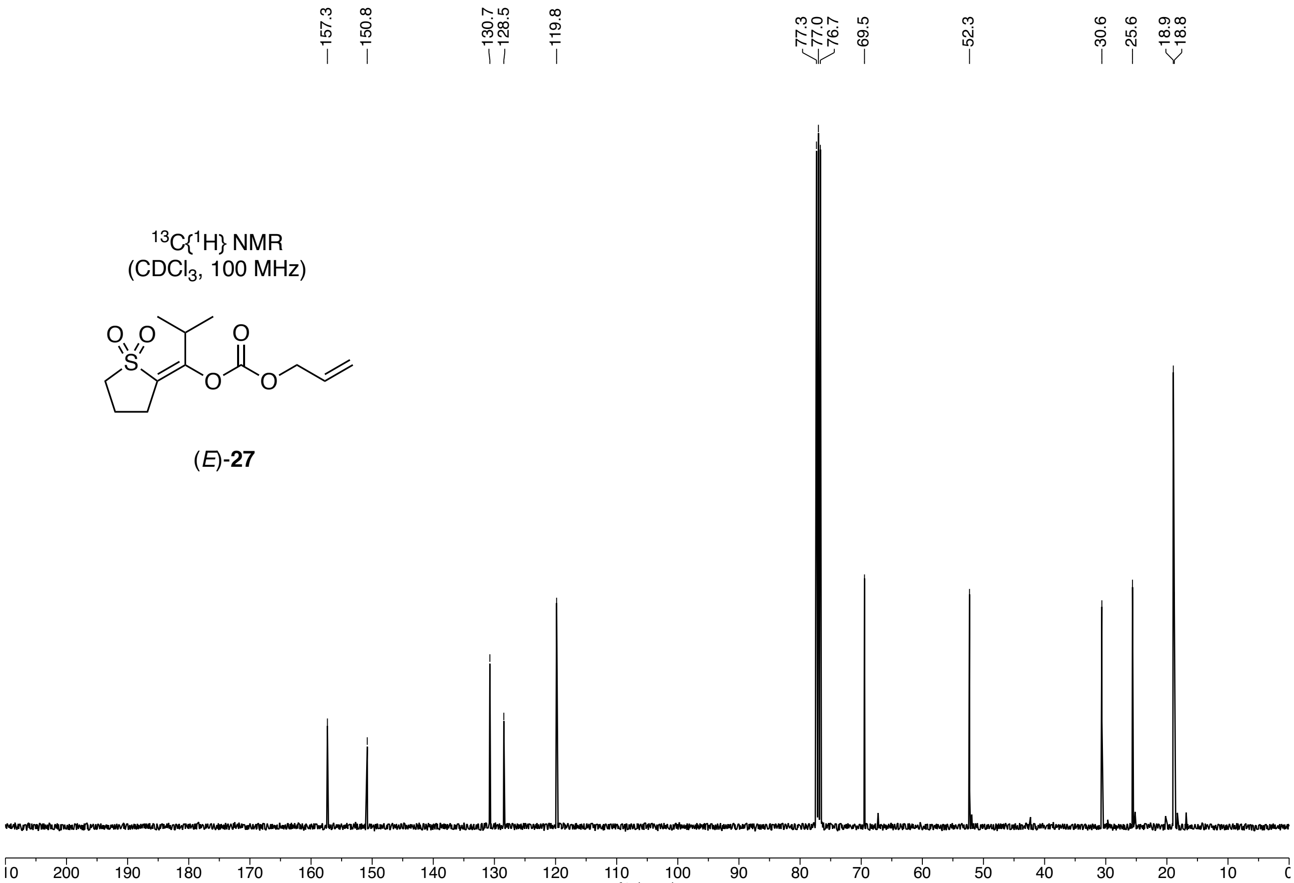
157.3 150.8 130.7 128.5 119.8 77.3 77.0 76.7 69.5 52.3 30.6 25.6 18.9 18.8
13C{1H} NMR
(CDCl3, 100 MHz)
(E)-27
f1 (ppm)

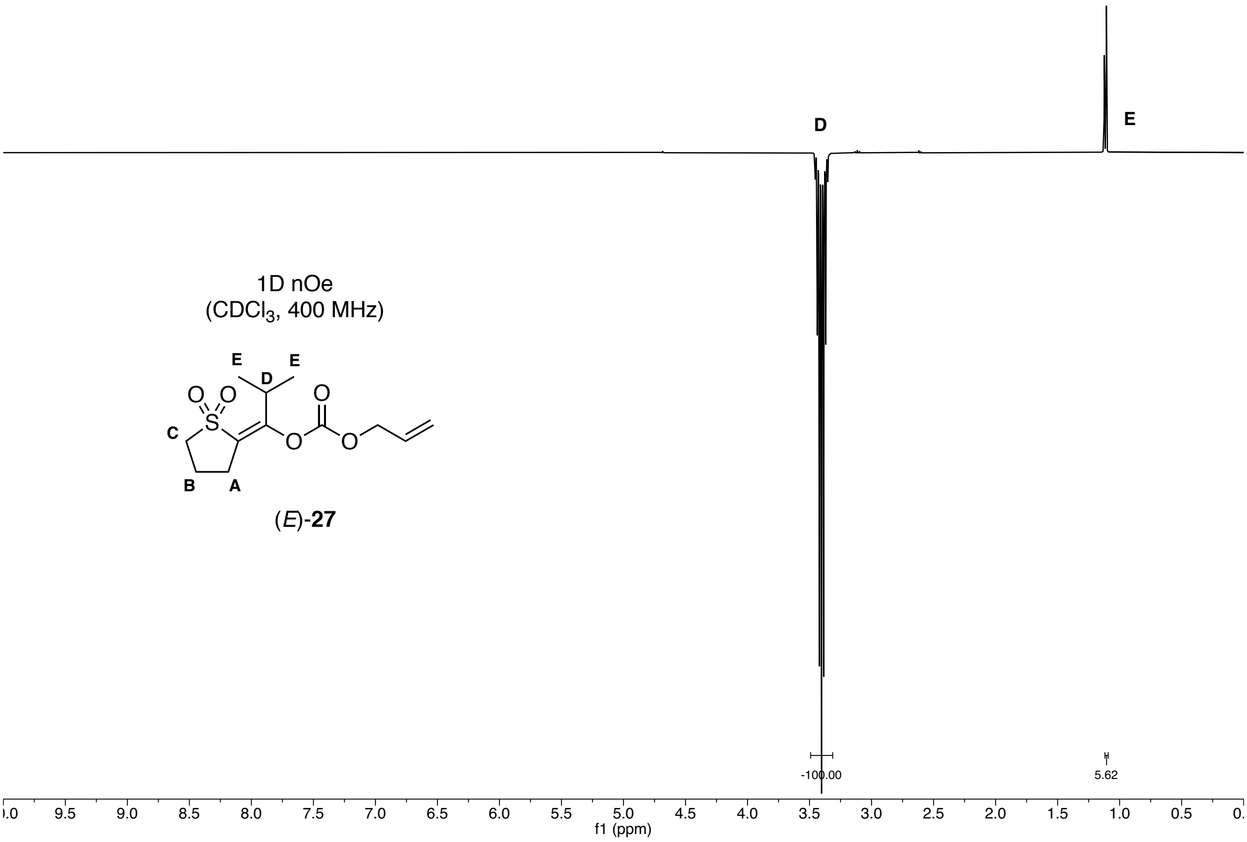
D
E
1D nOe
(CDCl3, 400 MHz)
(E)-27
-100.00
5.62
f1 (ppm)

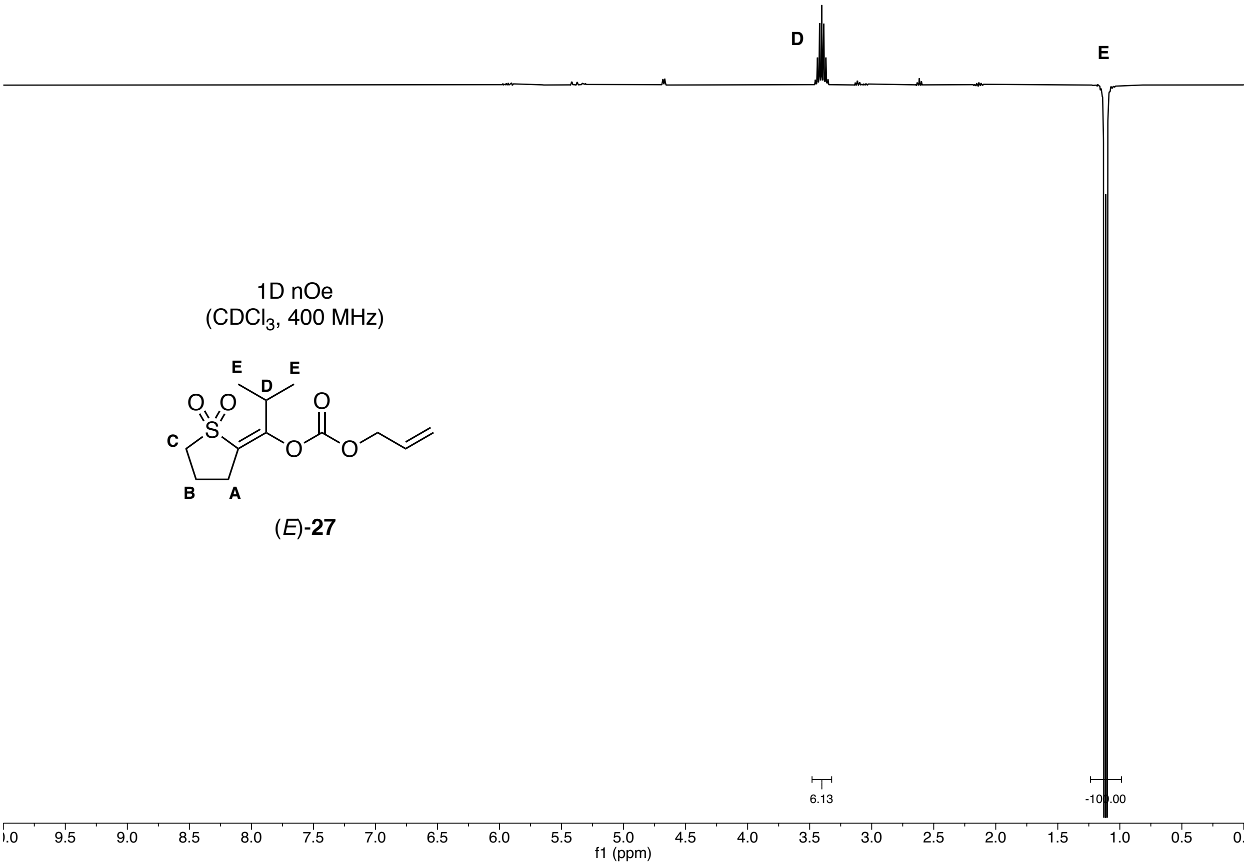
D
E
1D nOe
(CDCl3, 400 MHz)
(E)-27
6.13
f1 (ppm)

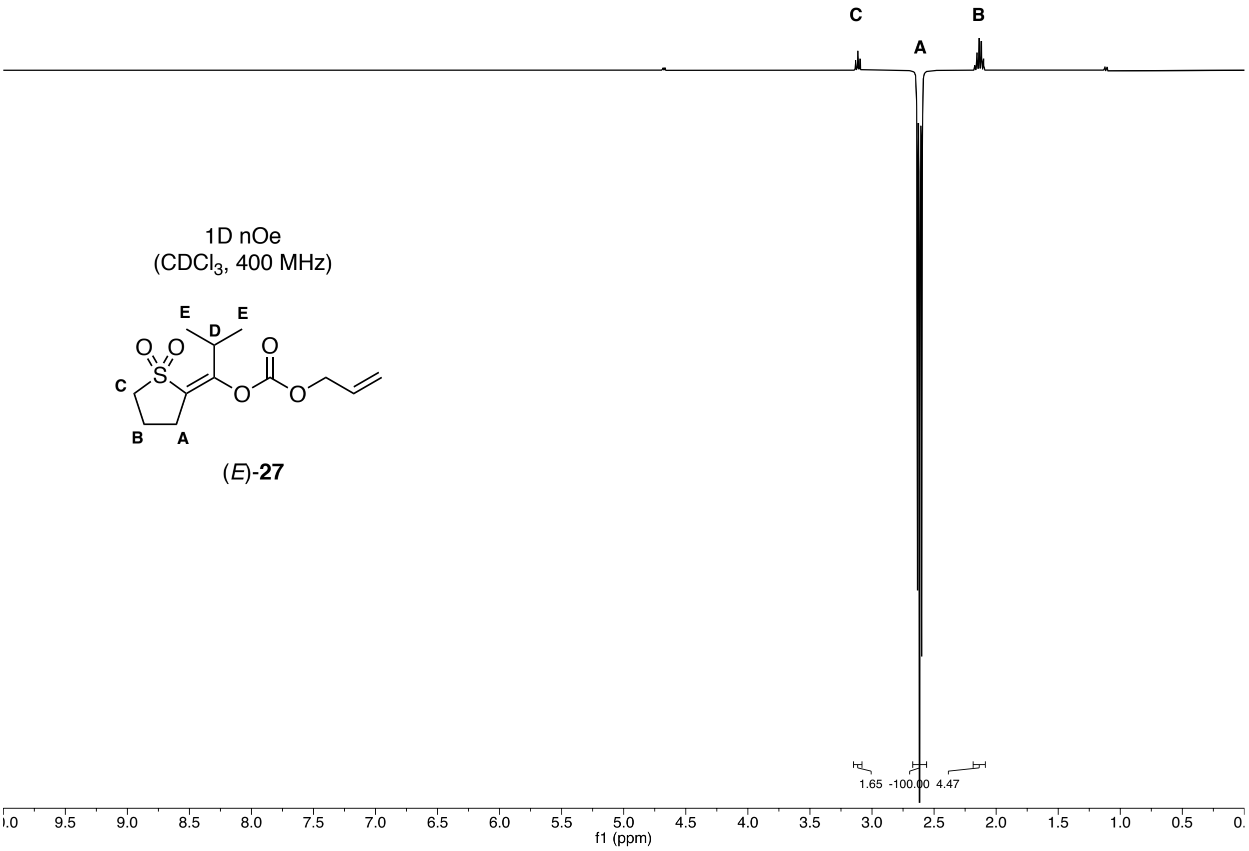

C
B
A
1D nOe
(CDCl3, 400 MHz)
E
D
E
C
B
A
(E)-27
1.65
-100.00
4.47
f1 (ppm)

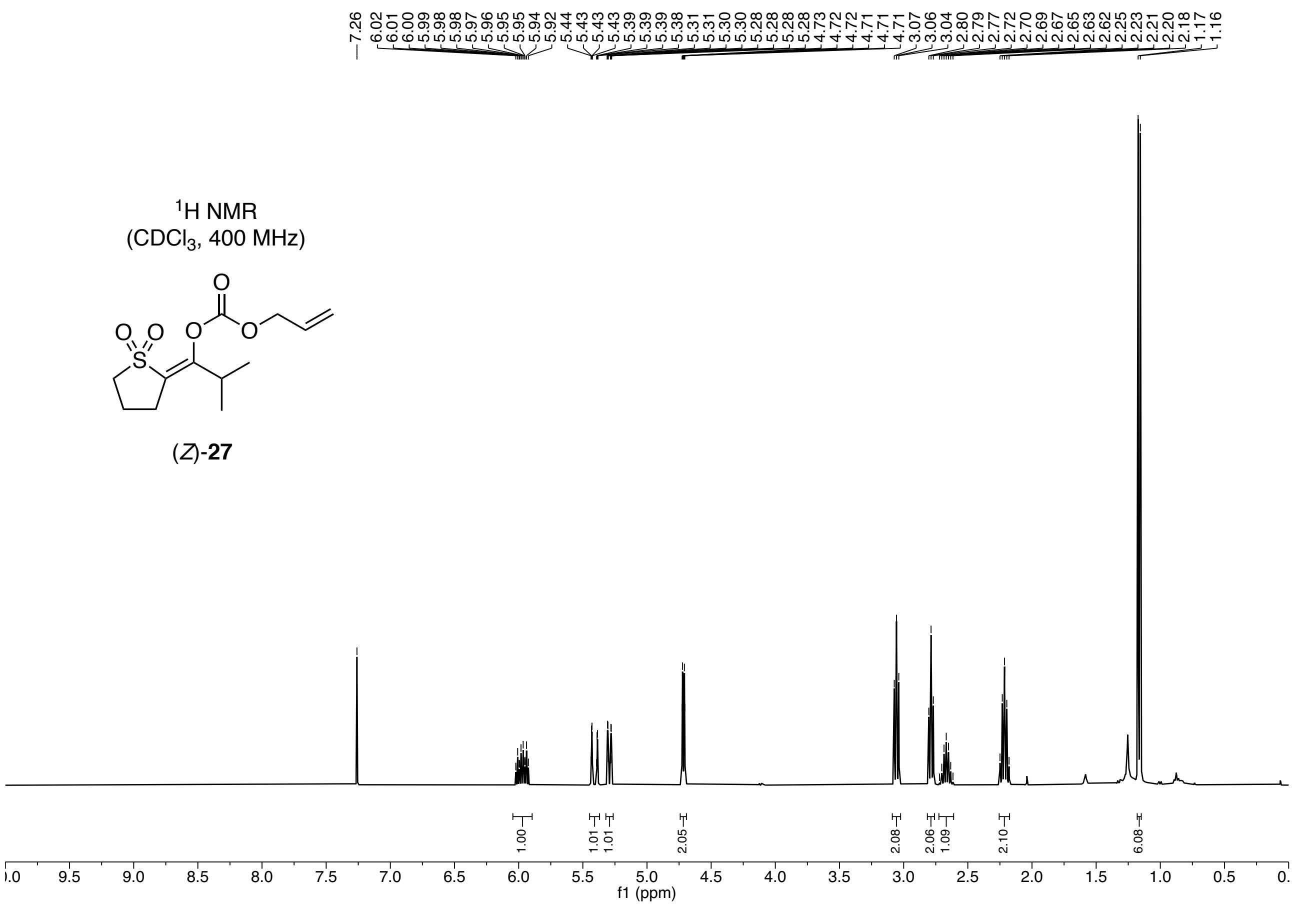

^{1}H NMR
($CDCl_3$, 400 MHz)

(*Z*)-**27**

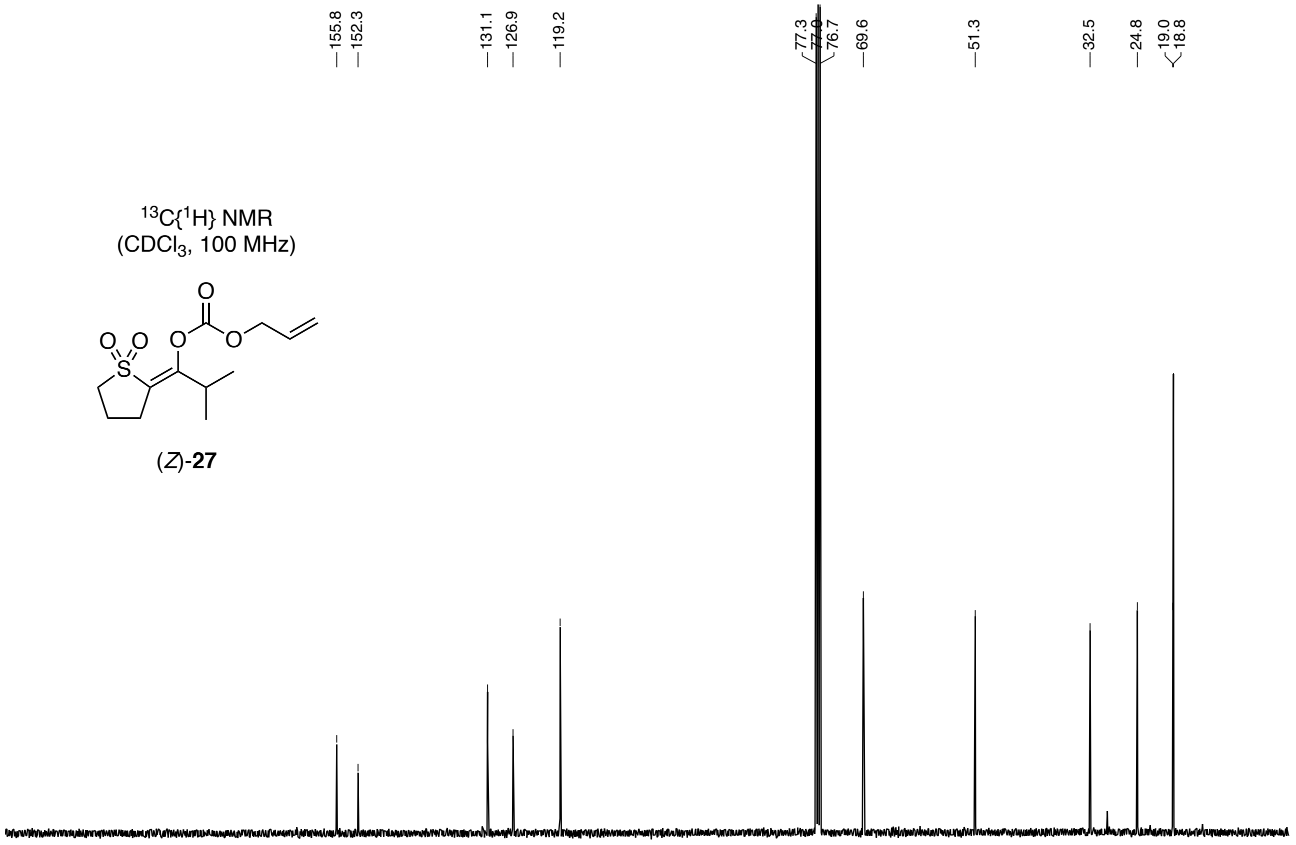

^{13}C{^{1}H} NMR
($CDCl_3$, 100 MHz)

(*Z*)-**27**

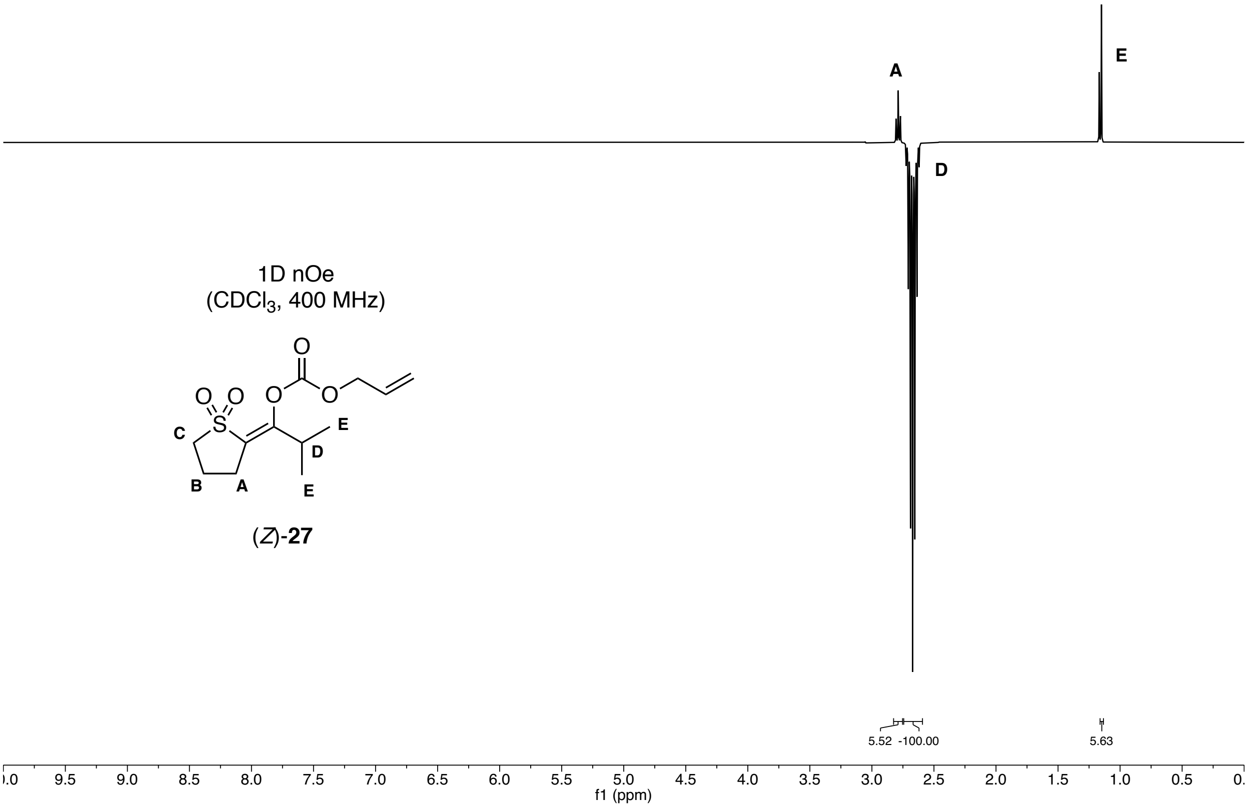
A
E
D
1D nOe
(CDCl3, 400 MHz)
C
B
A
D
E
E
(Z)-27
5.52 -100.00
5.63
f1 (ppm)

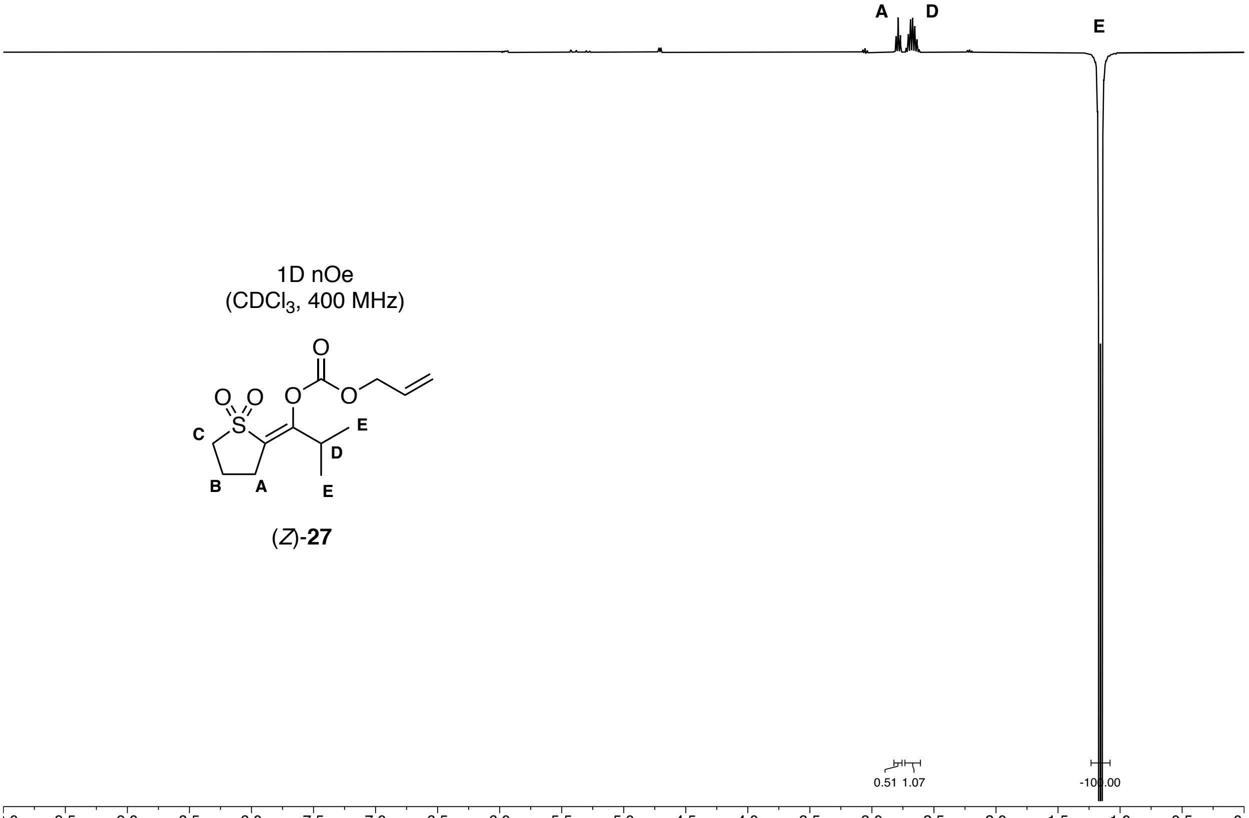
A
D
E
1D nOe
(CDCl3, 400 MHz)
C
B
A
D
E
E
(Z)-27
0.51 1.07
f1 (ppm)

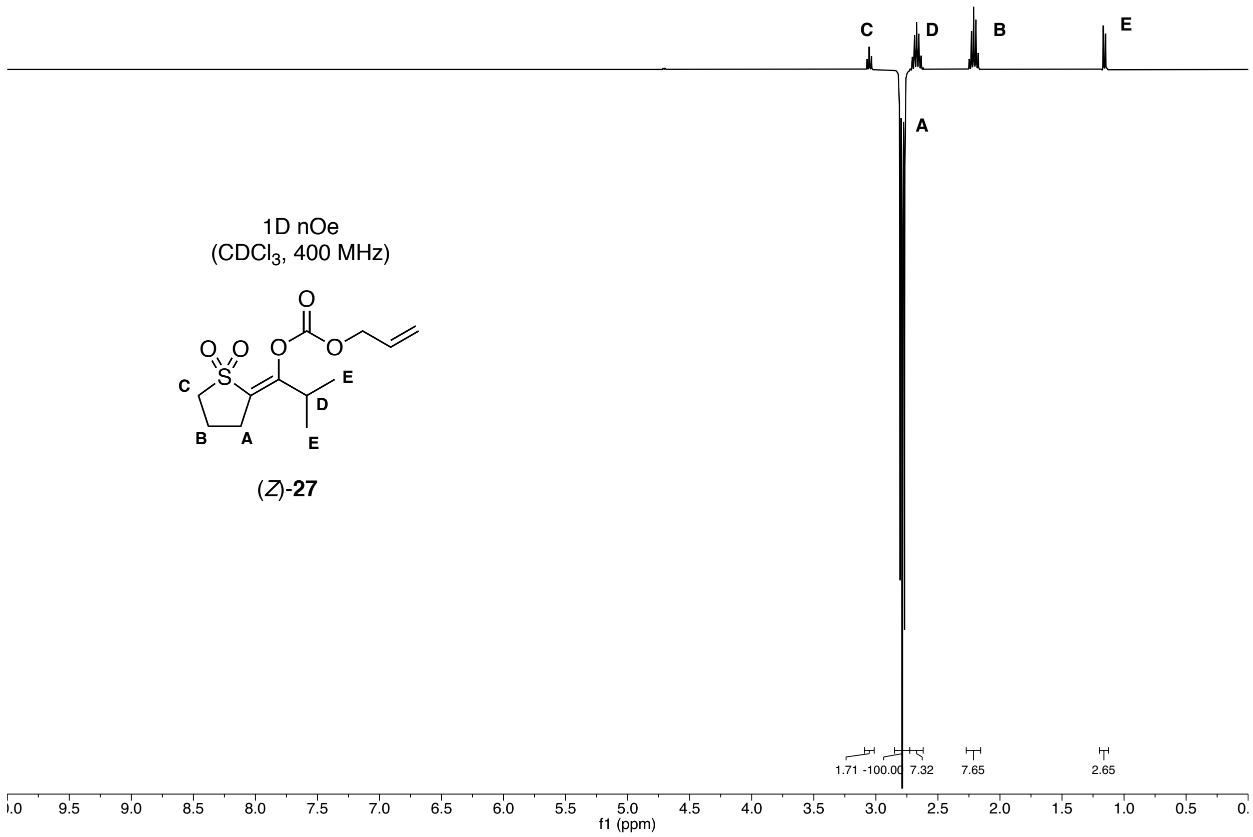
C
D
B
E
A
1D nOe
($CDCl_3$, 400 MHz)
O
O
O
S
O
O
O
C
E
D
B
A
E
(Z)-27
1.71
-100.00
7.32
7.65
2.65
f1 (ppm)

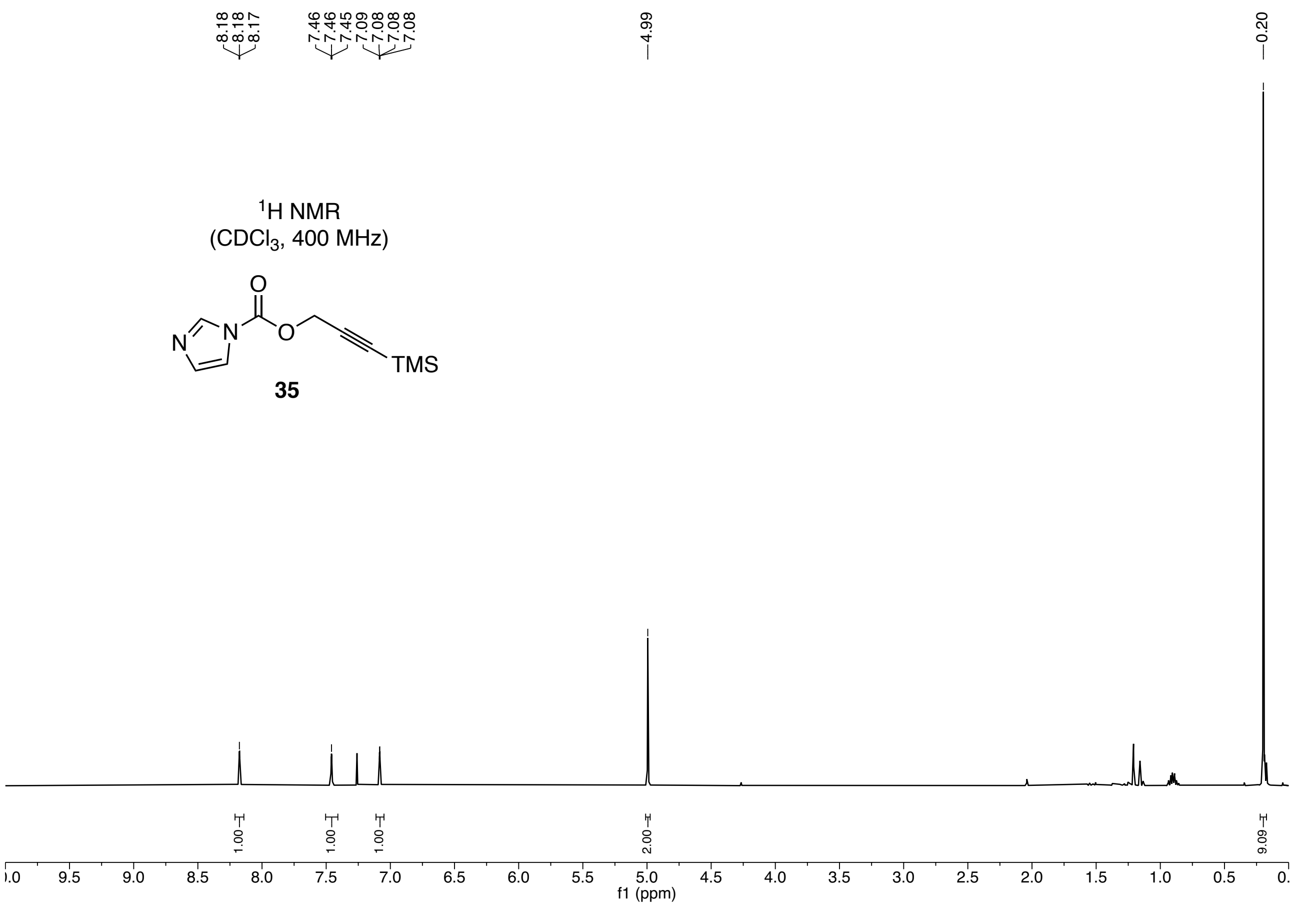
8.18
8.18
8.17
7.46
7.46
7.45
7.09
7.08
7.08
7.08
4.99
0.20
^{1}H NMR
(CDCl$_3$, 400 MHz)
O
N
N
O
TMS
35
1.00
1.00
1.00
2.00
9.09
f1 (ppm)

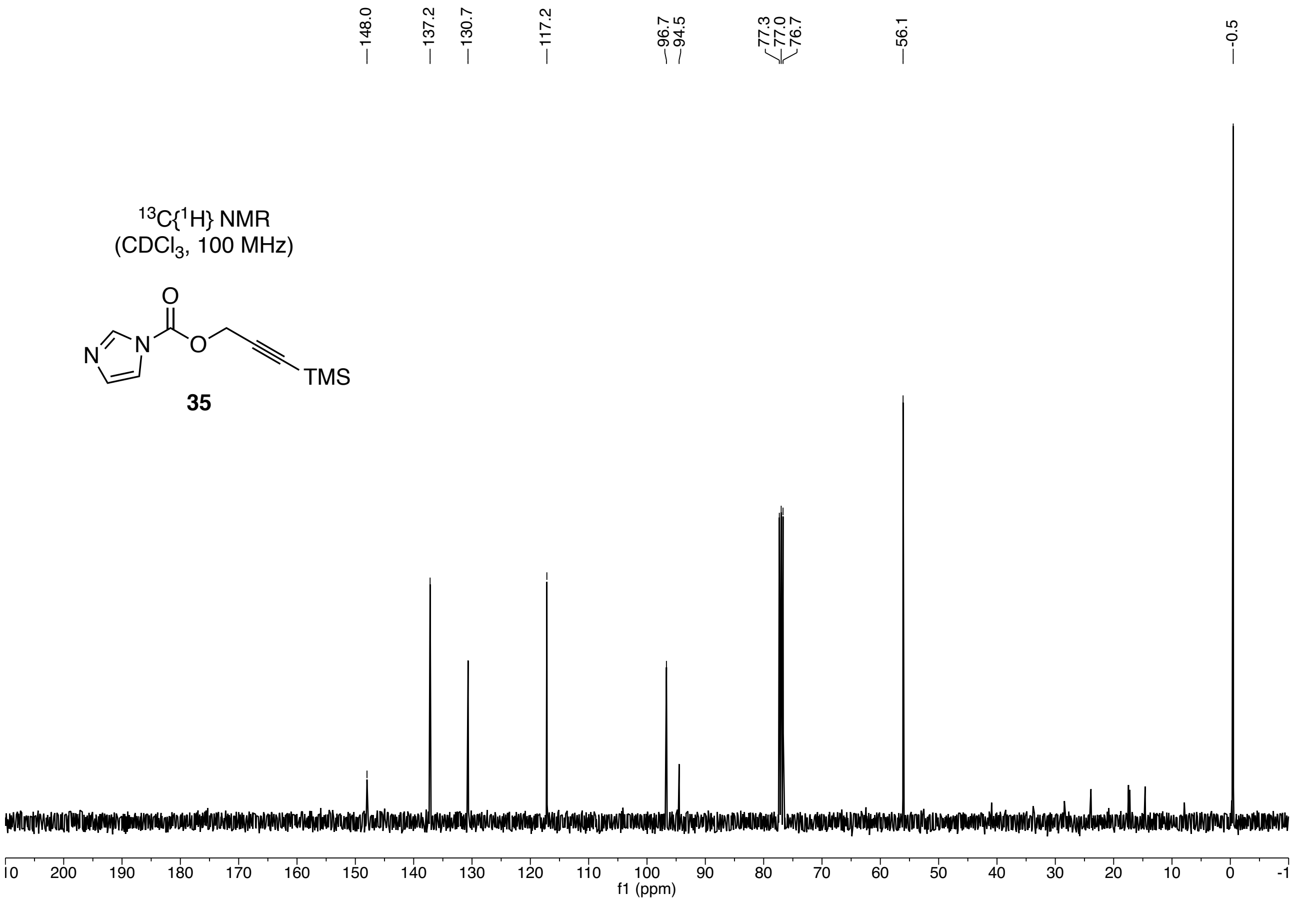
148.0
137.2
130.7
117.2
96.7
94.5
77.3
77.0
76.7
56.1
-0.5
^{13}C{^{1}H} NMR
(CDCl$_3$, 100 MHz)
O
N
N
O
TMS
35
f1 (ppm)

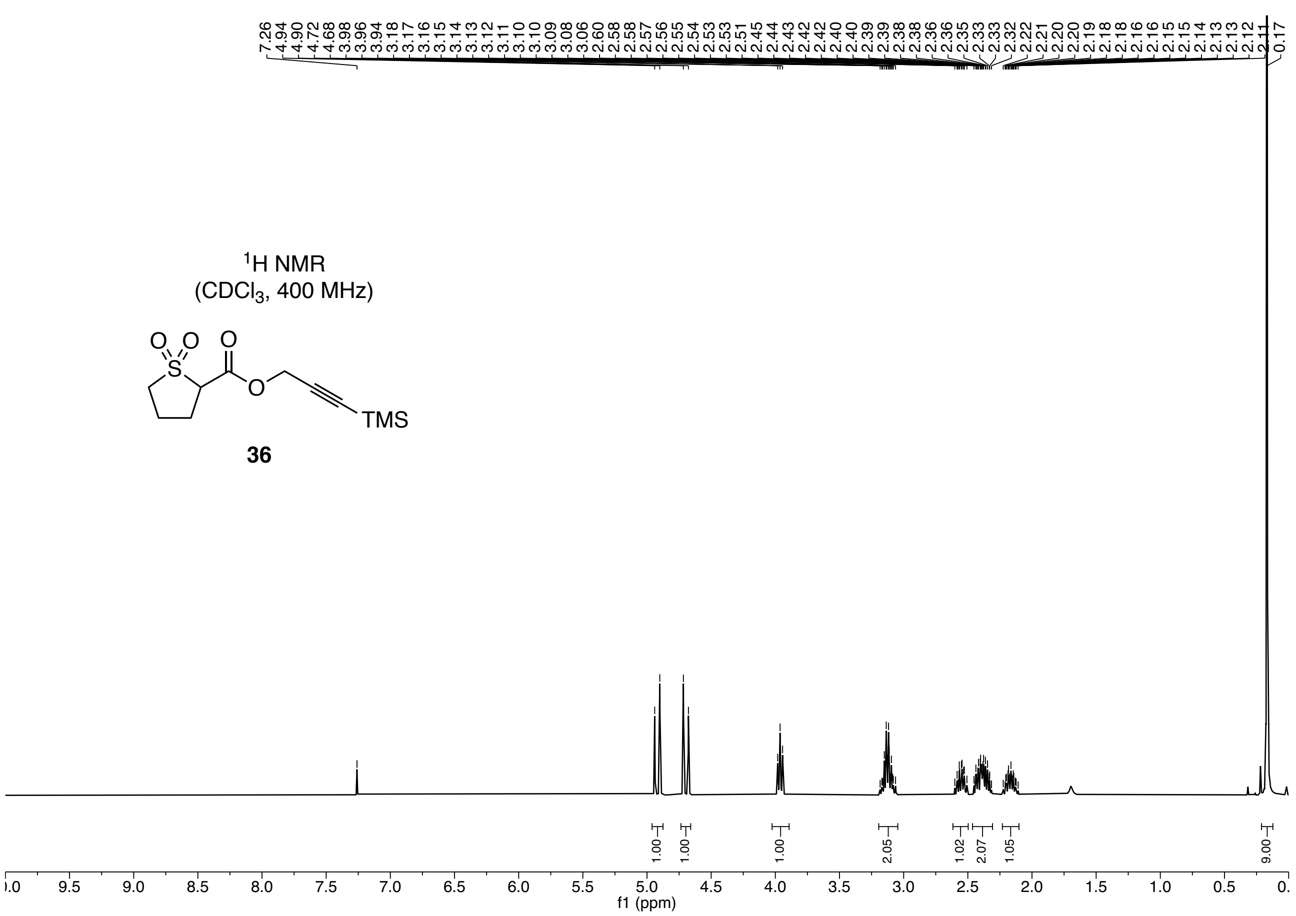

^{1}H NMR
(CDCl$_3$, 400 MHz)
TMS
36
f1 (ppm)

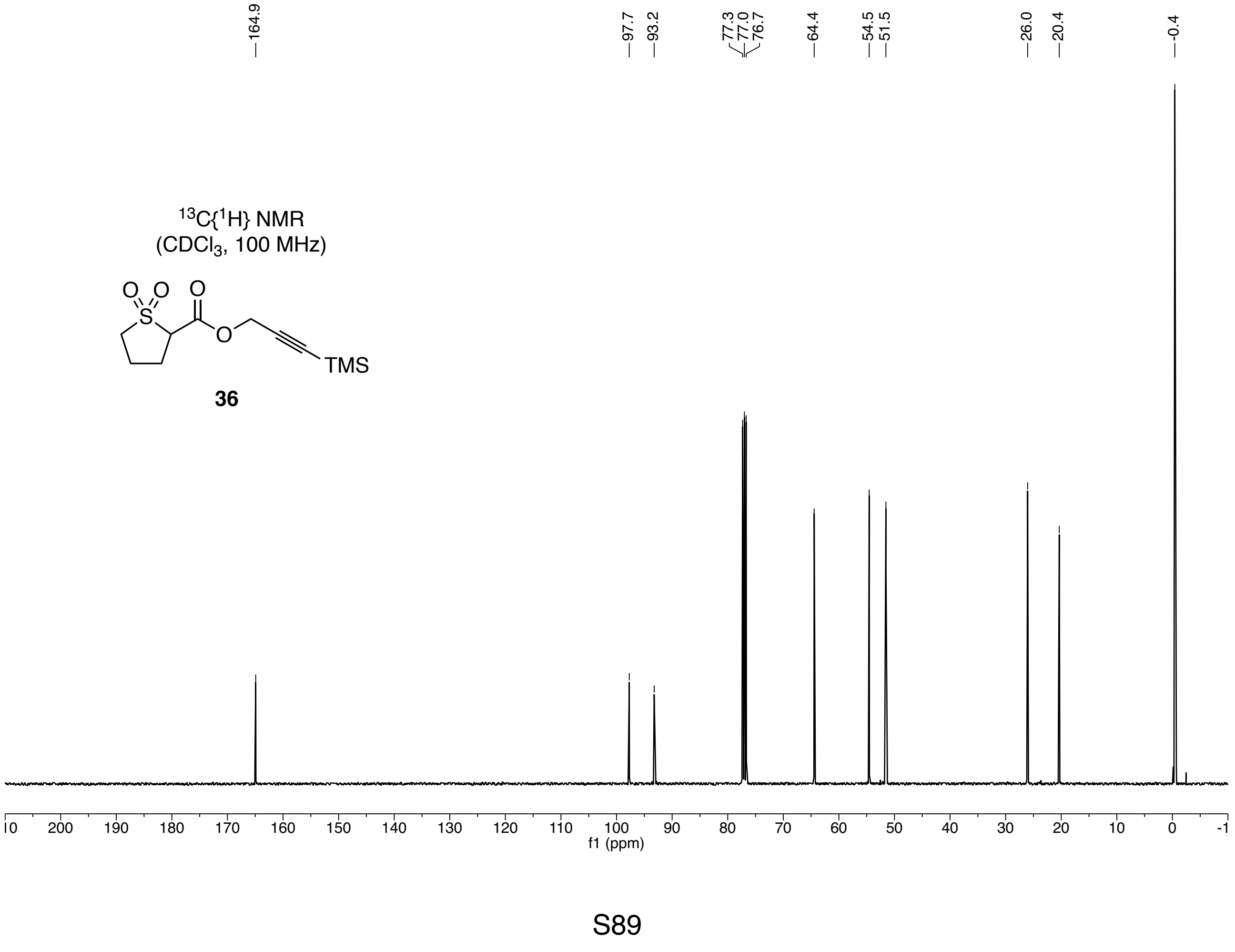

^{13}C{^{1}H} NMR
(CDCl$_3$, 100 MHz)
TMS
36
164.9
97.7
93.2
77.3
77.0
76.7
64.4
54.5
51.5
26.0
20.4
-0.4
f1 (ppm)

^{1}H NMR
($CDCl_3$, 400 MHz)

37

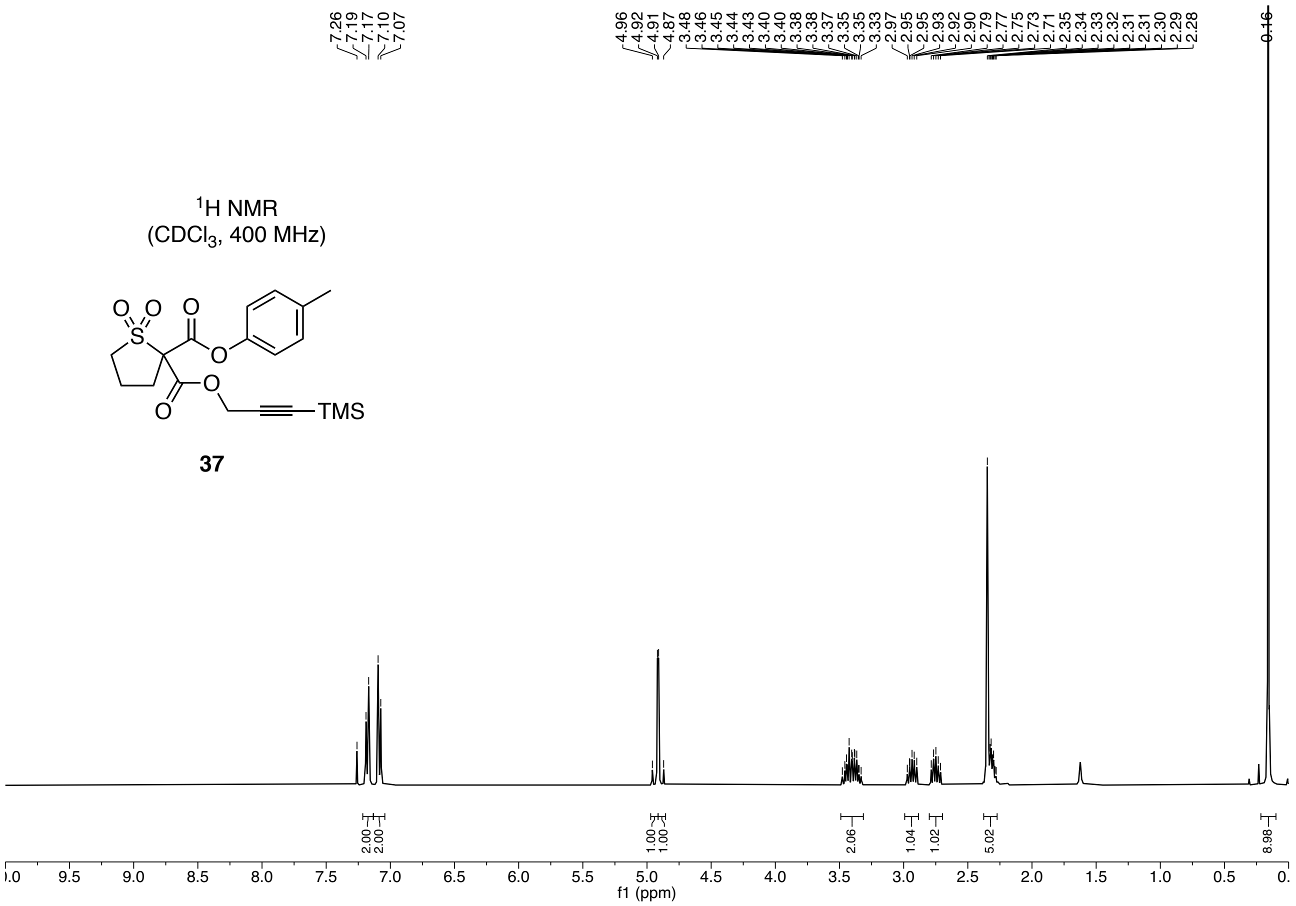

^{13}C{^{1}H} NMR
($CDCl_3$, 100 MHz)

37

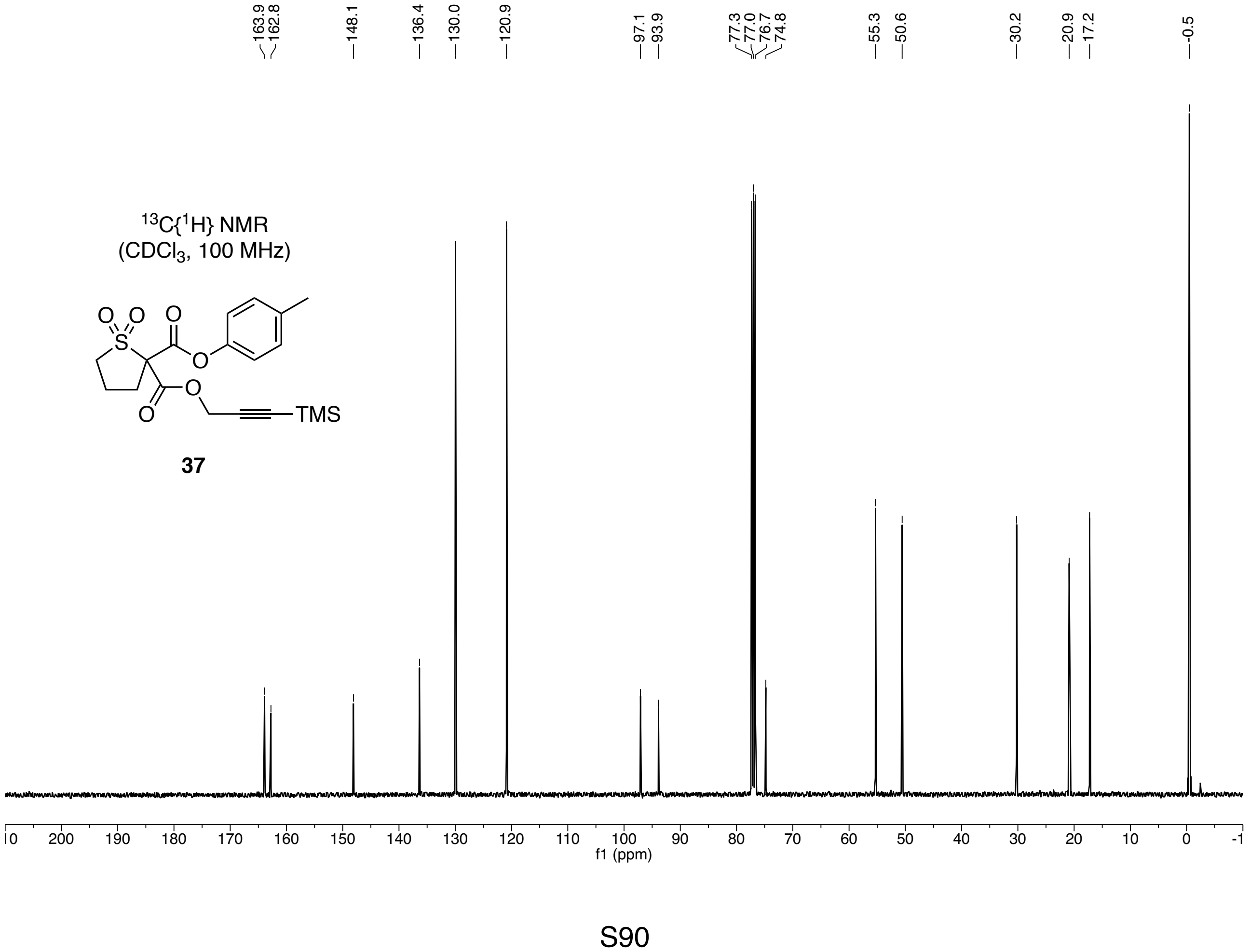

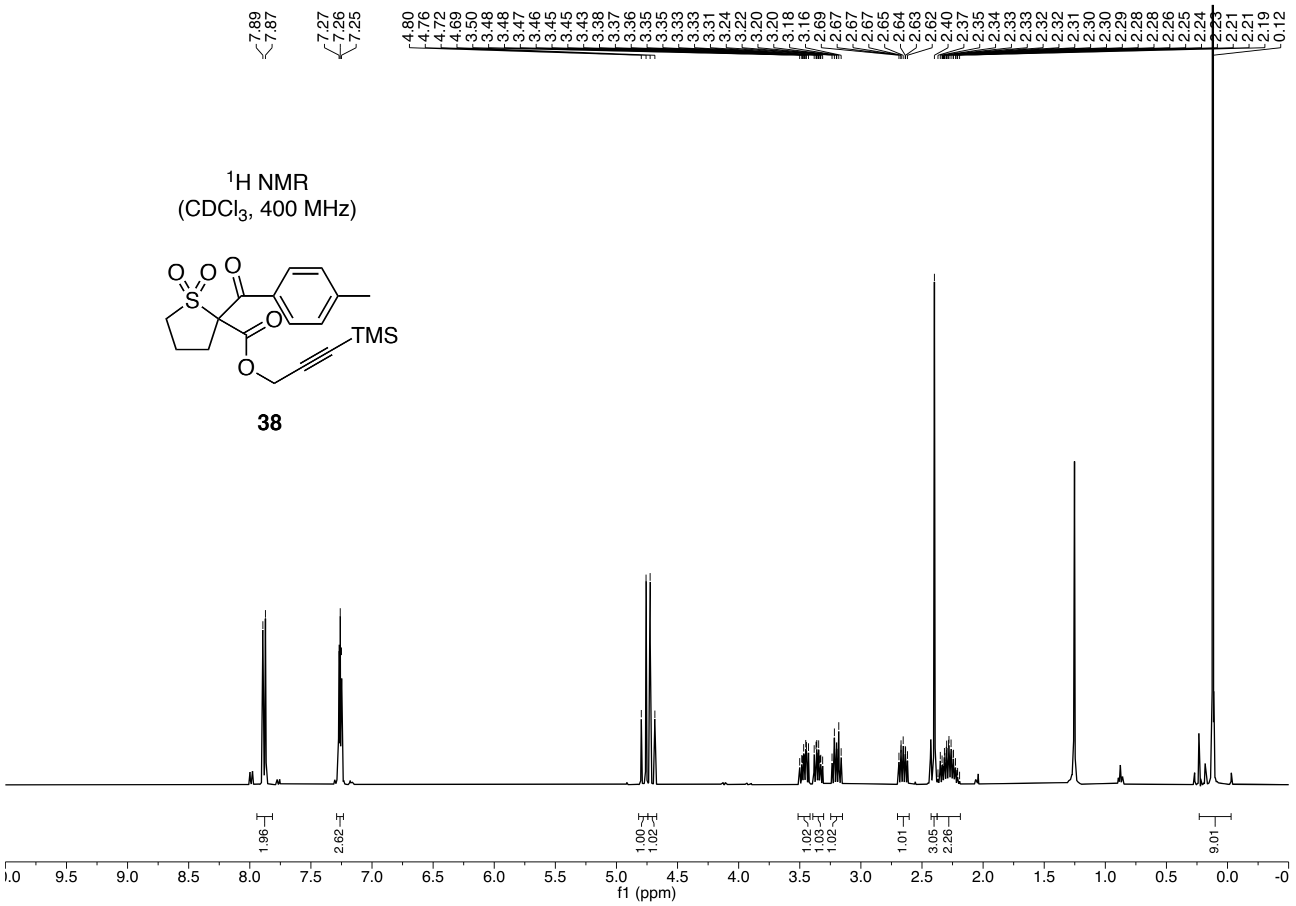

¹H NMR
(CDCl₃, 400 MHz)
TMS
38
f1 (ppm)

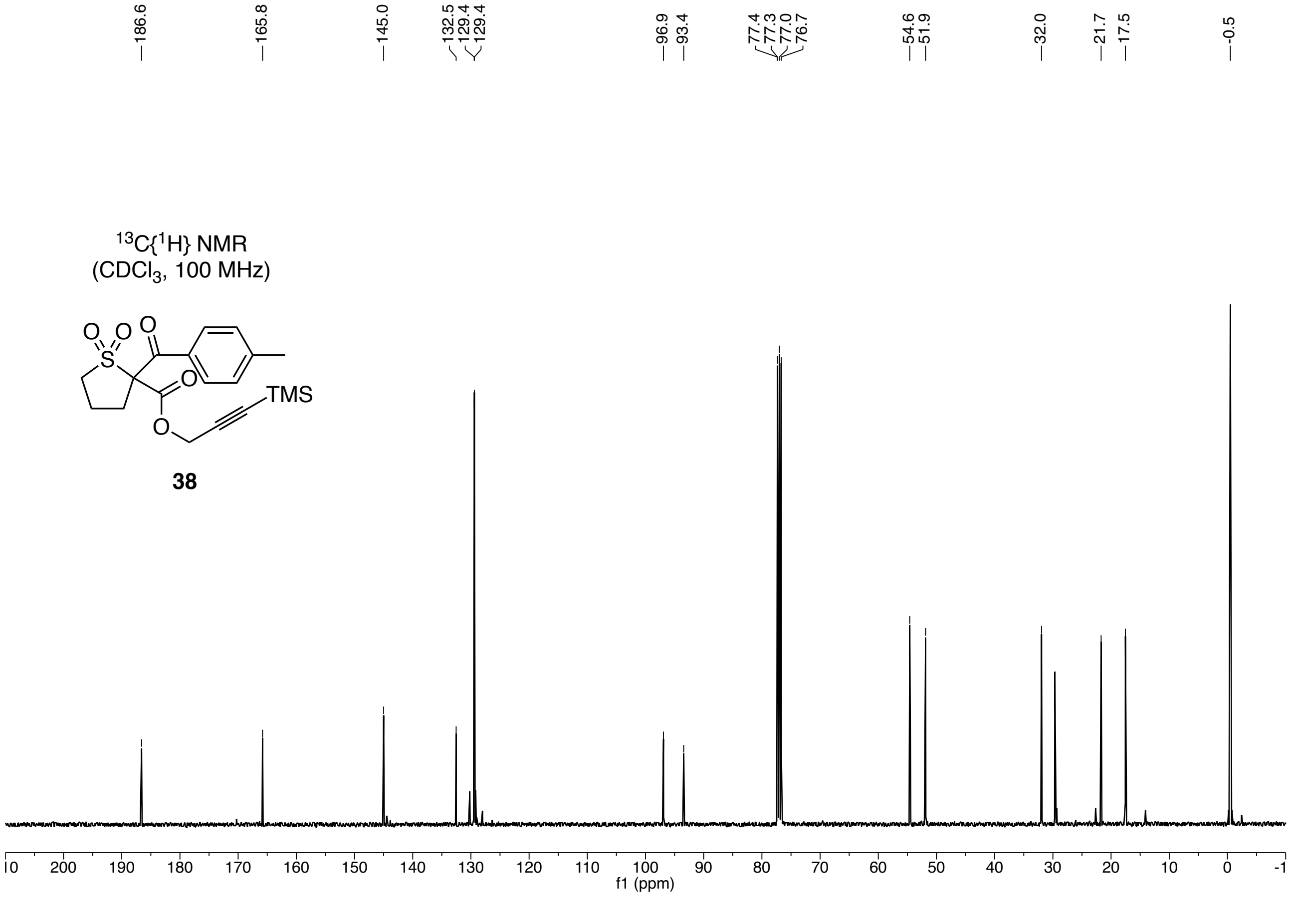

¹³C{¹H} NMR
(CDCl₃, 100 MHz)
TMS
38
f1 (ppm)

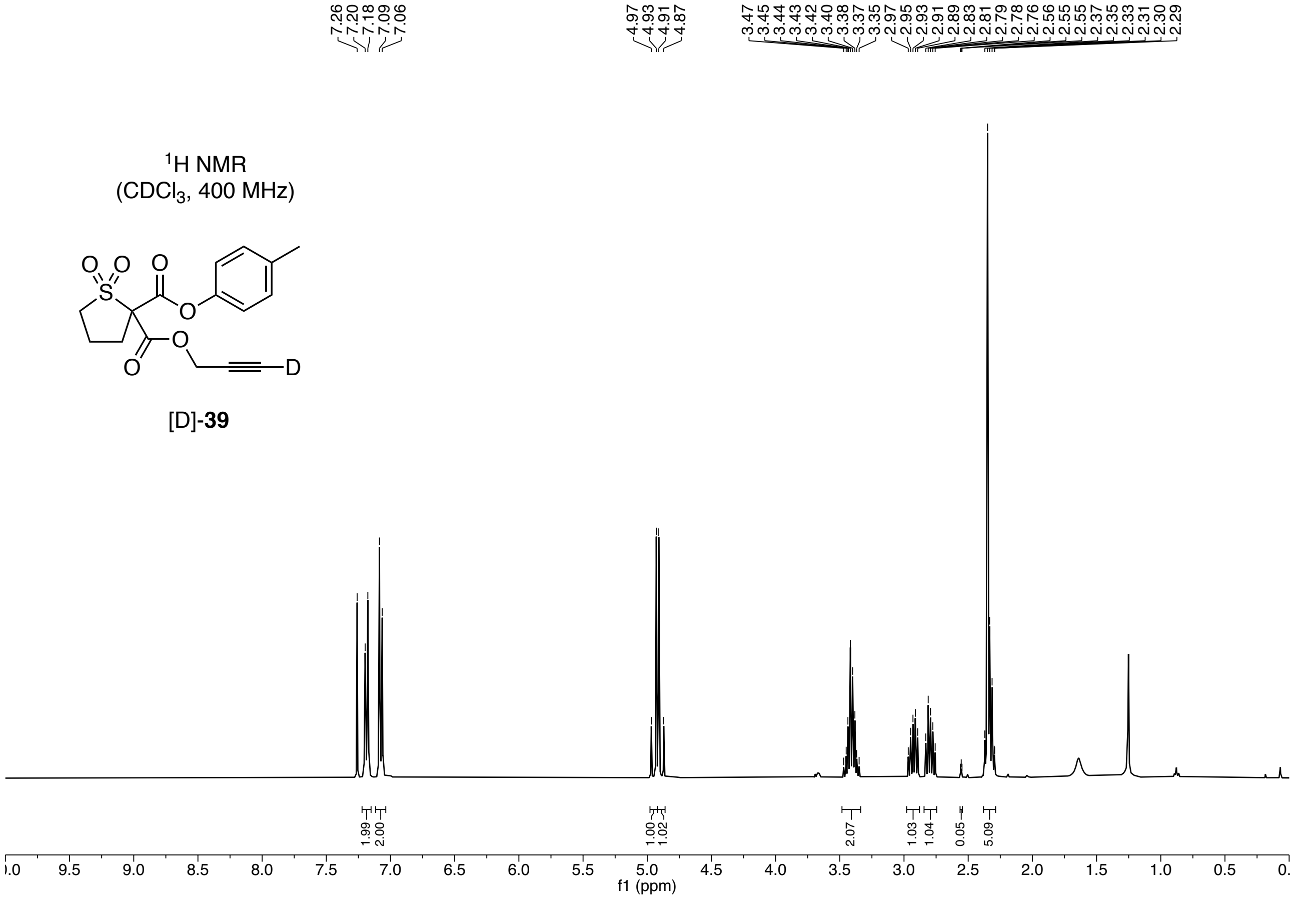
7.26
7.20
7.18
7.09
7.06
4.97
4.93
4.91
4.87
3.47
3.45
3.44
3.43
3.42
3.40
3.38
3.37
3.35
2.97
2.95
2.93
2.91
2.89
2.83
2.81
2.79
2.78
2.76
2.56
2.55
2.37
2.35
2.33
2.31
2.30
2.29
^{1}H NMR
($CDCl_3$, 400 MHz)
O O O
S
O
O
O
D
[D]-**39**
1.99
2.00
1.00
1.02
2.07
1.03
1.04
0.05
5.09
9.5 9.0 8.5 8.0 7.5 7.0 6.5 6.0 5.5 5.0 4.5 4.0 3.5 3.0 2.5 2.0 1.5 1.0 0.5
f1 (ppm)

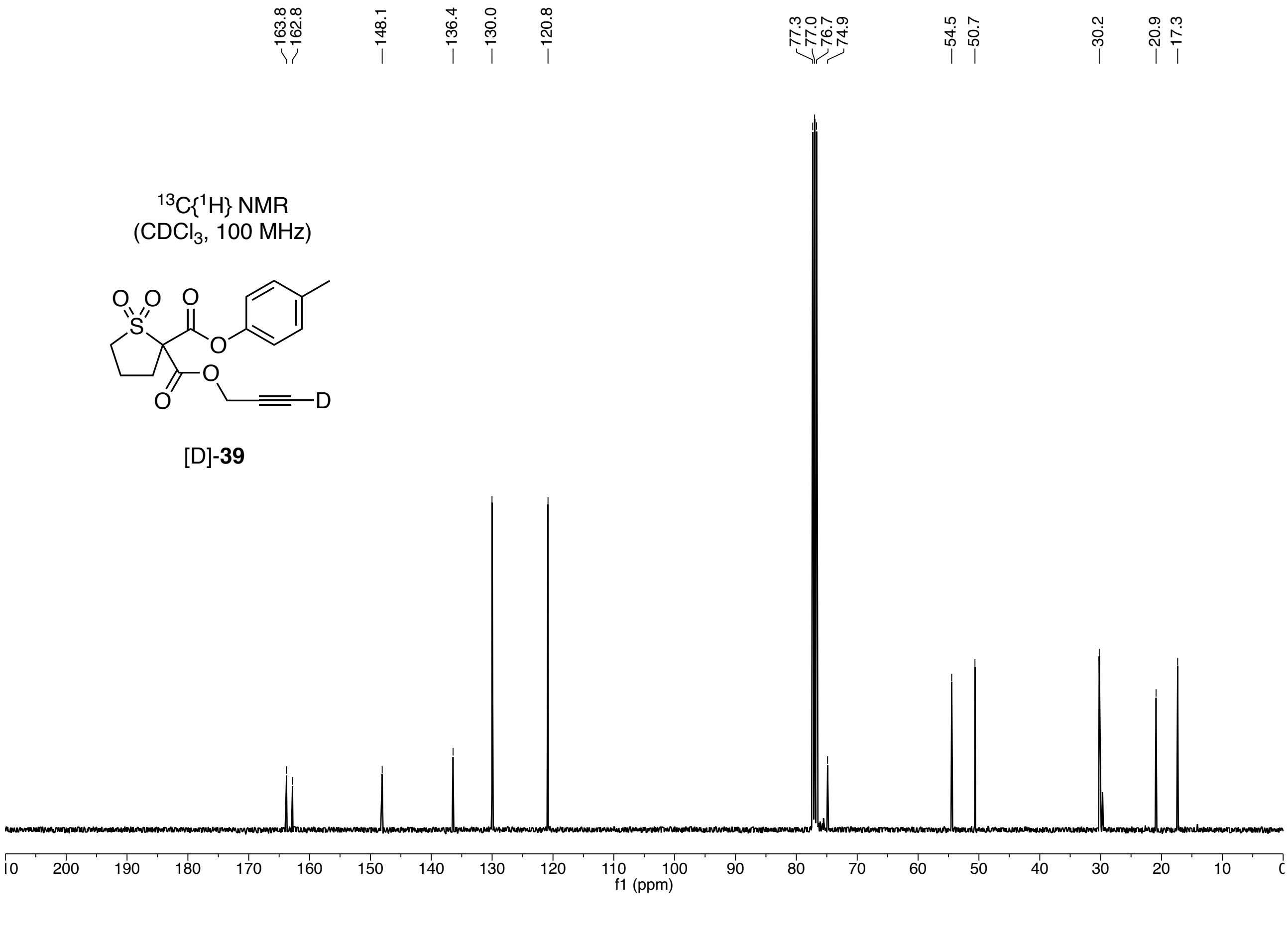
163.8
162.8
148.1
136.4
130.0
120.8
77.3
77.0
76.7
74.9
54.5
50.7
30.2
20.9
17.3
^{13}C{^{1}H} NMR
($CDCl_3$, 100 MHz)
O O O
S
O
O
O
D
[D]-**39**
200 190 180 170 160 150 140 130 120 110 100 90 80 70 60 50 40 30 20 10
f1 (ppm)

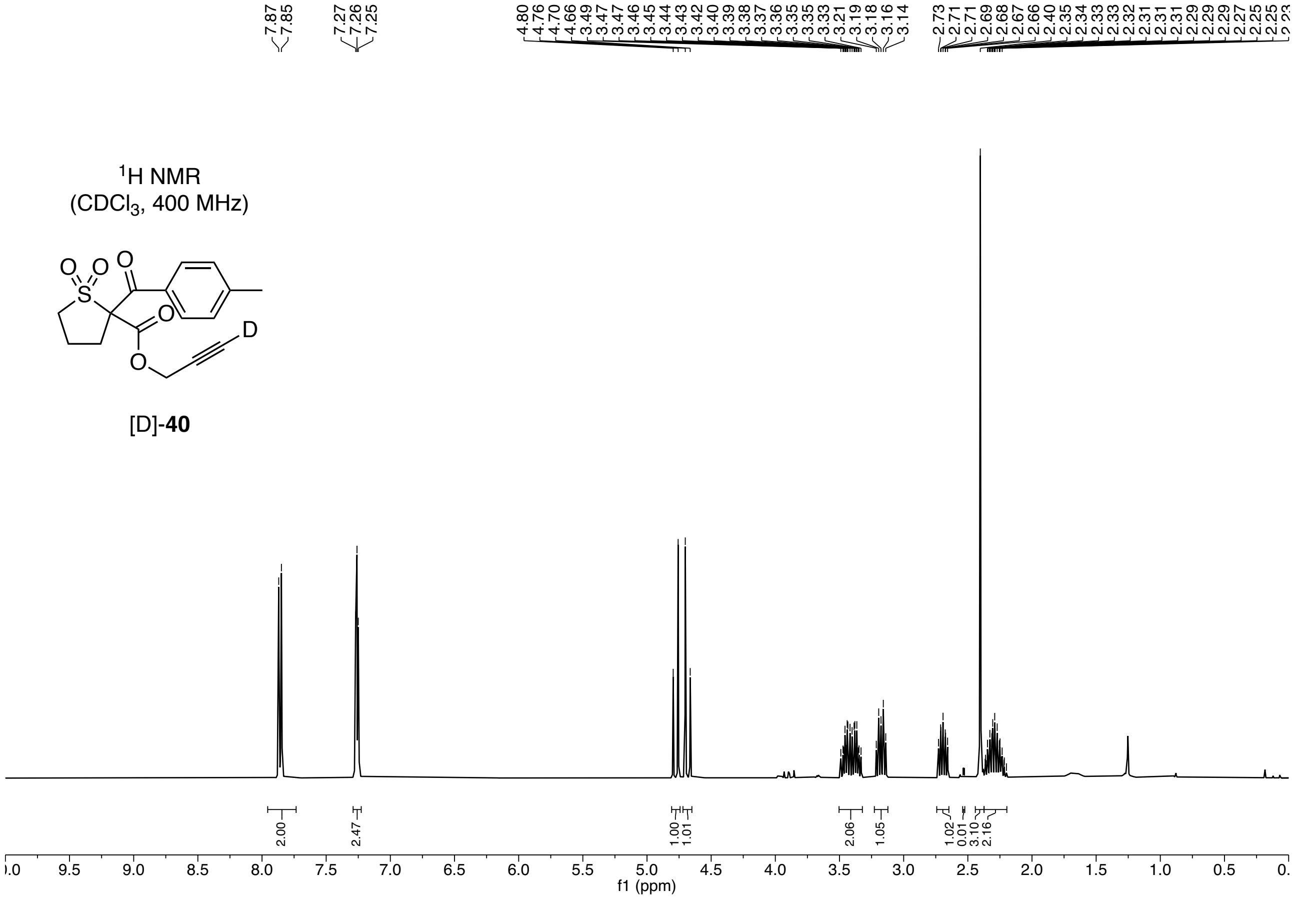
7.87
7.85
7.27
7.26
7.25
4.80
4.76
4.70
4.66
3.49
3.47
3.47
3.46
3.45
3.44
3.43
3.42
3.40
3.39
3.38
3.37
3.36
3.35
3.35
3.33
3.21
3.19
3.18
3.16
3.14
2.73
2.71
2.71
2.69
2.68
2.67
2.66
2.40
2.35
2.34
2.33
2.33
2.32
2.31
2.31
2.29
2.29
2.29
2.27
2.25
^{1}H NMR
($CDCl_3$, 400 MHz)
O
S
O
O
O
O
D
[D]-**40**
2.00
2.47
1.00
1.01
2.06
1.05
1.02
0.01
3.10
2.16
9.5
9.0
8.5
8.0
7.5
7.0
6.5
6.0
5.5
5.0
4.5
4.0
3.5
3.0
2.5
2.0
1.5
1.0
0.5
f1 (ppm)

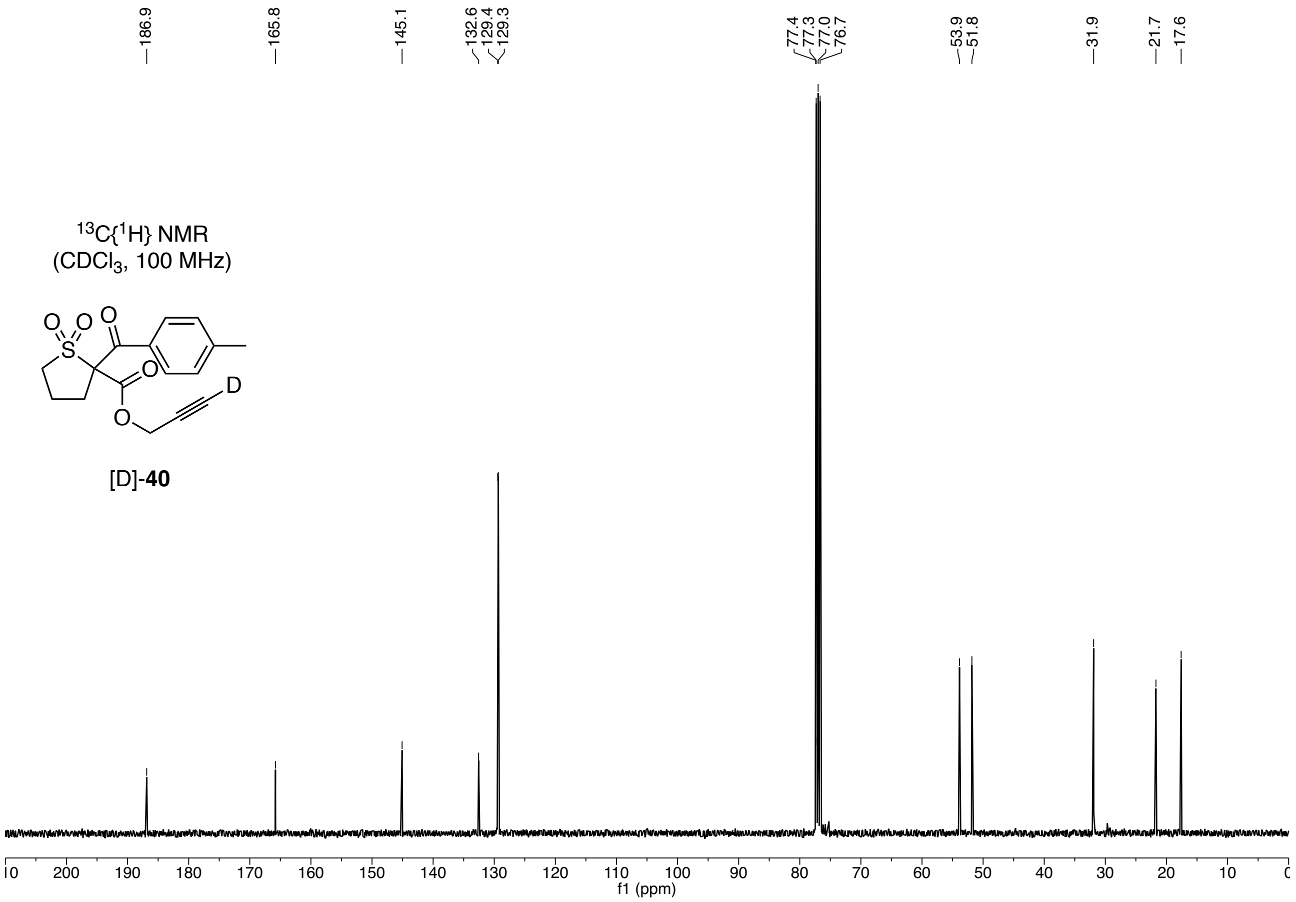
186.9
165.8
145.1
132.6
129.4
129.3
77.4
77.3
77.0
76.7
53.9
51.8
31.9
21.7
17.6
$^{13}C\{^1H\}$ NMR
($CDCl_3$, 100 MHz)
O
S
O
O
O
O
D
[D]-**40**
200
190
180
170
160
150
140
130
120
110
100
90
80
70
60
50
40
30
20
10
f1 (ppm)

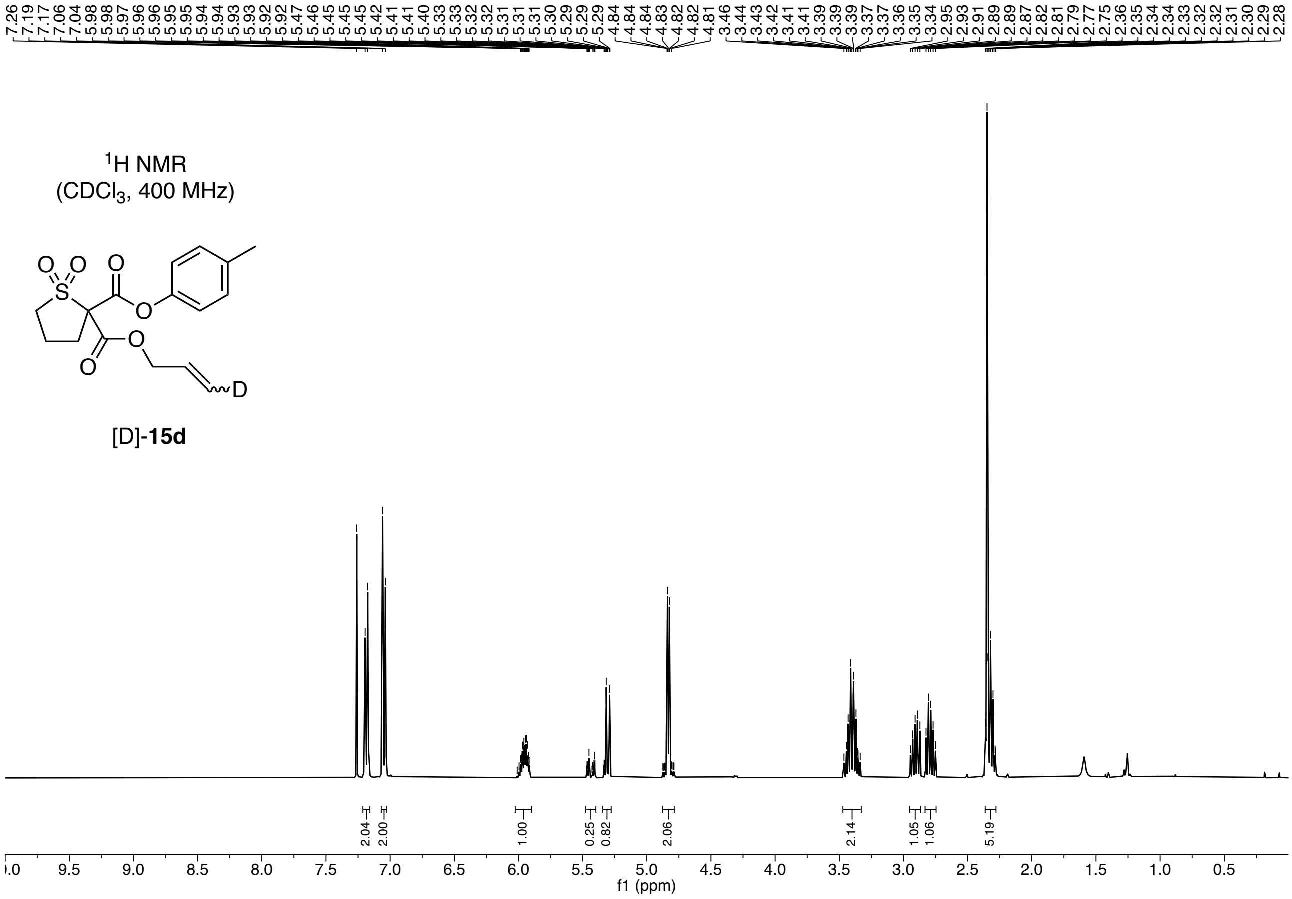

7.26 7.19 7.17 7.06 7.04 5.98 5.97 5.96 5.96 5.95 5.94 5.93 5.93 5.92 5.92 5.47 5.46 5.45 5.45 5.42 5.41 5.41 5.40 5.33 5.33 5.32 5.32 5.31 5.31 5.30 5.29 5.29 4.84 4.84 4.83 4.82 4.82 4.81 3.46 3.44 3.43 3.42 3.41 3.39 3.39 3.37 3.36 3.35 3.34 2.95 2.93 2.91 2.89 2.87 2.82 2.81 2.79 2.77 2.75 2.36 2.35 2.34 2.34 2.33 2.32 2.32 2.31 2.30 2.29 2.28
1H NMR
(CDCl3, 400 MHz)
[D]-15d
2.04 2.00 1.00 0.25 0.82 2.06 2.14 1.05 1.06 5.19
f1 (ppm)

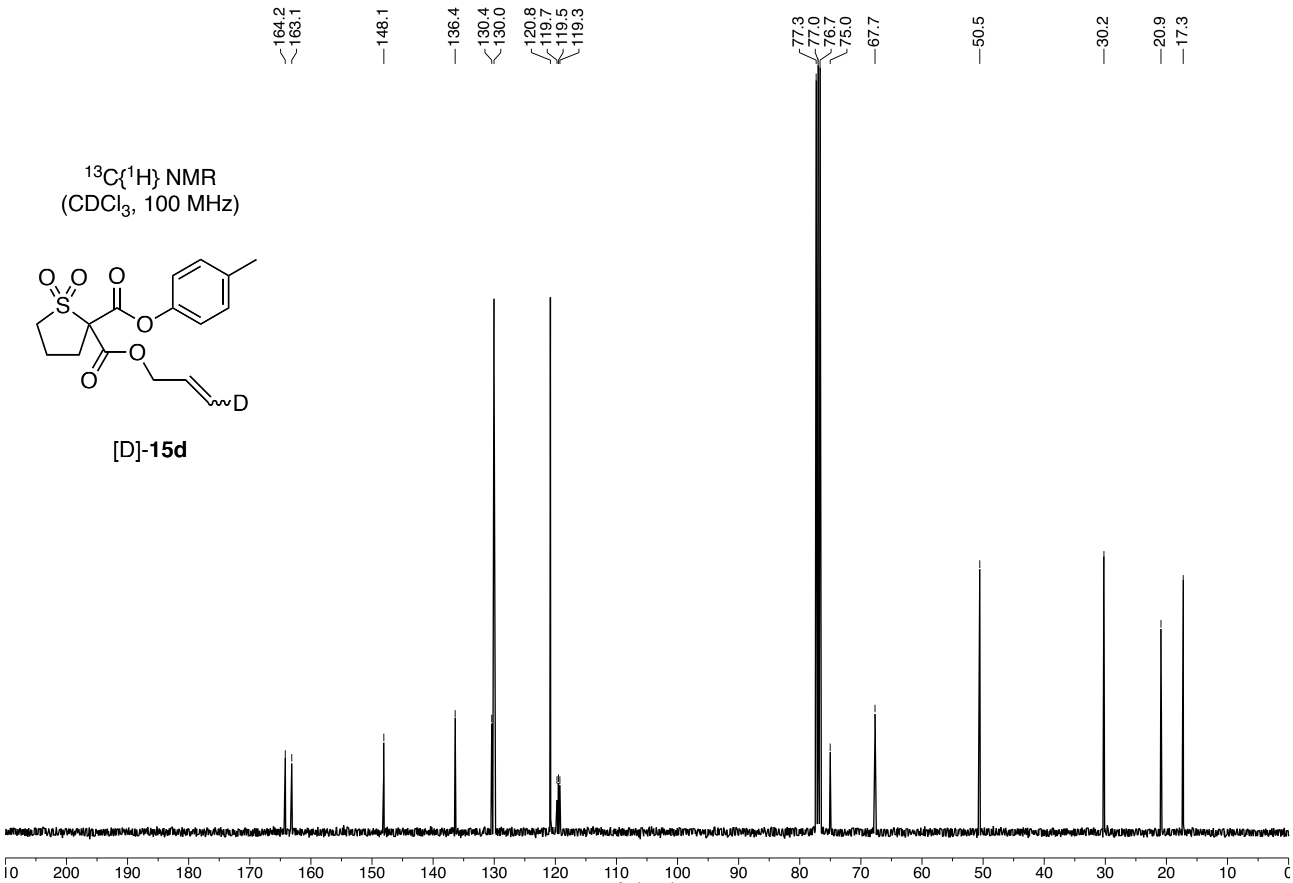

164.2 163.1 148.1 136.4 130.4 130.0 120.8 119.7 119.5 119.3 77.3 77.0 76.7 75.0 67.7 50.5 30.2 20.9 17.3
13C{1H} NMR
(CDCl3, 100 MHz)
[D]-15d
f1 (ppm)

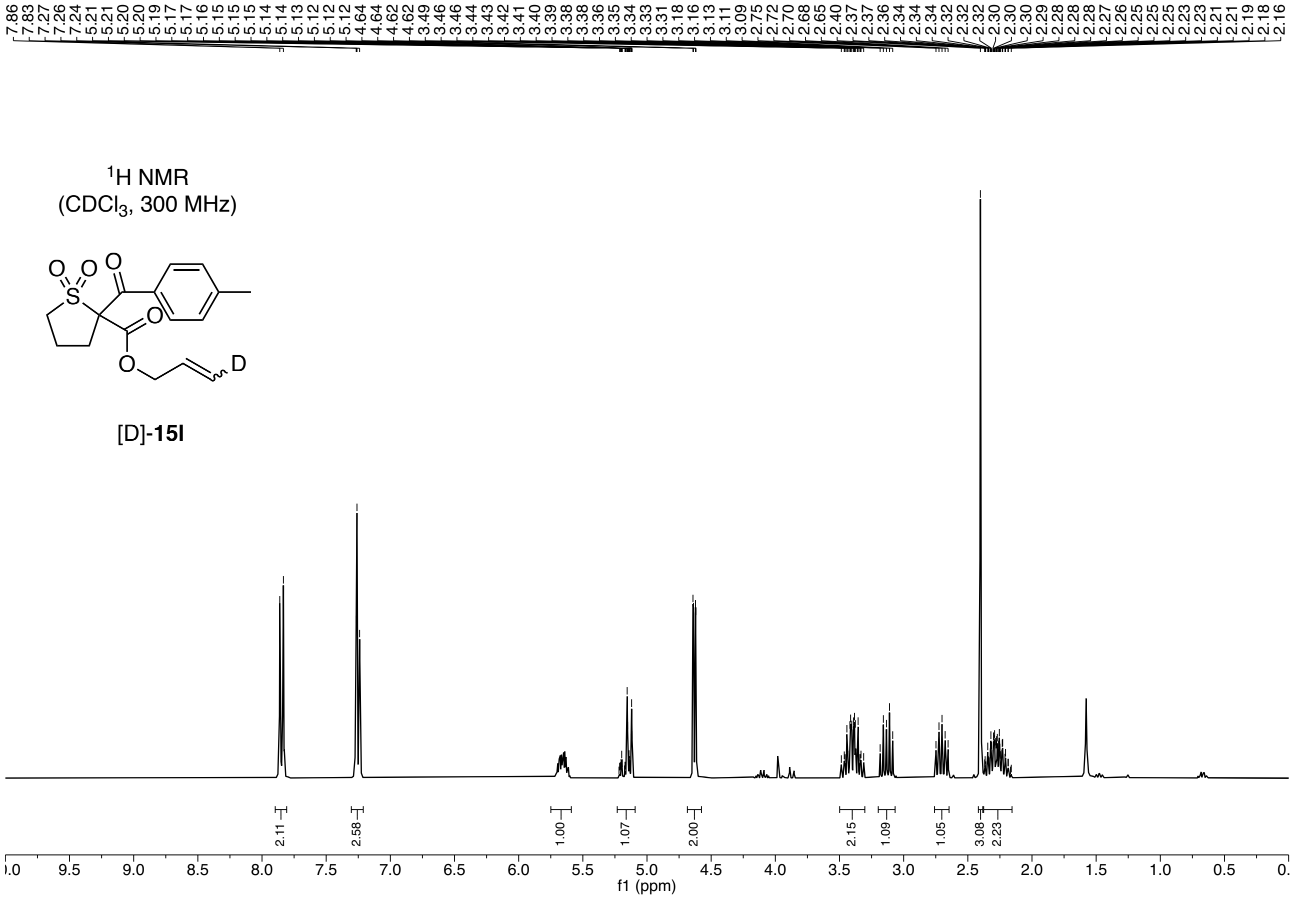
7.86 7.83 7.27 7.26 7.24 7.21 5.21 5.20 5.19 5.17 5.16 5.15 5.15 5.15 5.14 5.14 5.13 5.12 5.12 5.12 4.64 4.64 4.62 4.62 3.49 3.46 3.46 3.44 3.43 3.42 3.41 3.40 3.39 3.38 3.38 3.36 3.35 3.34 3.33 3.31 3.18 3.16 3.13 3.11 3.09 2.75 2.72 2.72 2.70 2.68 2.65 2.40 2.37 2.37 2.36 2.34 2.34 2.34 2.32 2.32 2.32 2.30 2.30 2.30 2.29 2.28 2.28 2.28 2.27 2.26 2.25 2.25 2.25 2.23 2.23 2.21 2.21 2.19 2.18 2.16
1H NMR
(CDCl3, 300 MHz)
O O O
S
O
O
D
[D]-15l
2.11 2.58 1.00 1.07 2.00 2.15 1.09 1.05 3.08 2.23
9.5 9.0 8.5 8.0 7.5 7.0 6.5 6.0 5.5 5.0 4.5 4.0 3.5 3.0 2.5 2.0 1.5 1.0 0.5
f1 (ppm)

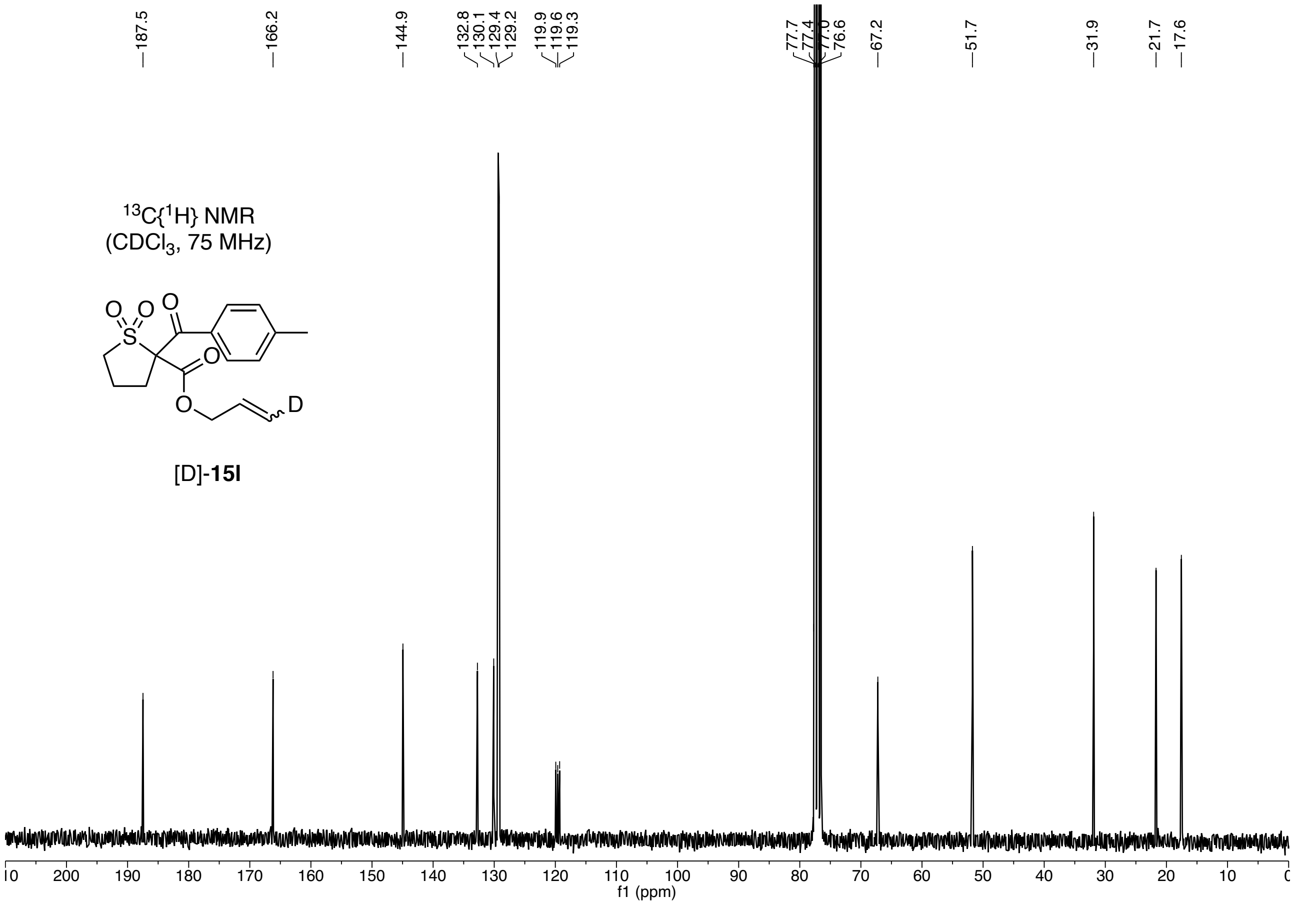
187.5 166.2 144.9 132.8 130.1 129.4 129.2 119.9 119.6 119.3 77.7 77.4 77.0 76.6 67.2 51.7 31.9 21.7 17.6
13C{1H} NMR
(CDCl3, 75 MHz)
O O O
S
O
O
D
[D]-15l
200 190 180 170 160 150 140 130 120 110 100 90 80 70 60 50 40 30 20 10
f1 (ppm)

2. HPLC Data

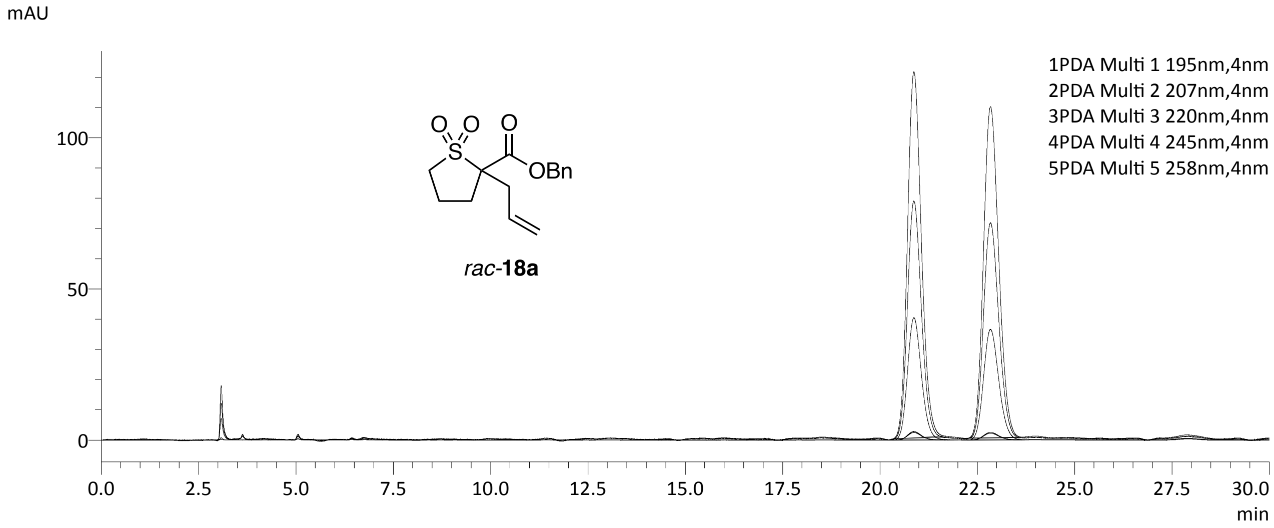

Peak Table

PDA Ch1 195nm

Name	Peak#	Ret. Time	Area	Area%	Mark
Compound 1	1	20.871	1963907	50.145	
Compound 2	2	22.843	1952518	49.855	
	Total		3916425	100.000	

PDA Ch2 207nm

Name	Peak#	Ret. Time	Area	Area%	Mark
Compound 1	1	20.873	3009961	50.287	
Compound 2	2	22.841	2975663	49.713	
	Total		5985625	100.000	

PDA Ch3 220nm

Name	Peak#	Ret. Time	Area	Area%	Mark
Compound 1	1	20.874	1012317	50.306	
Compound 2	2	22.841	1000006	49.694	
	Total		2012323	100.000	

PDA Ch4 245nm

Name	Peak#	Ret. Time	Area	Area%	Mark
Compound 1	1	20.875	61570	50.779	S
Compound 2	2	22.842	59681	49.221	
	Total		121251	100.000	

PDA Ch5 258nm

Name	Peak#	Ret. Time	Area	Area%	Mark
Compound 1	1	20.875	69192	50.229	
Compound 2	2	22.841	68560	49.771	S
	Total		137752	100.000	

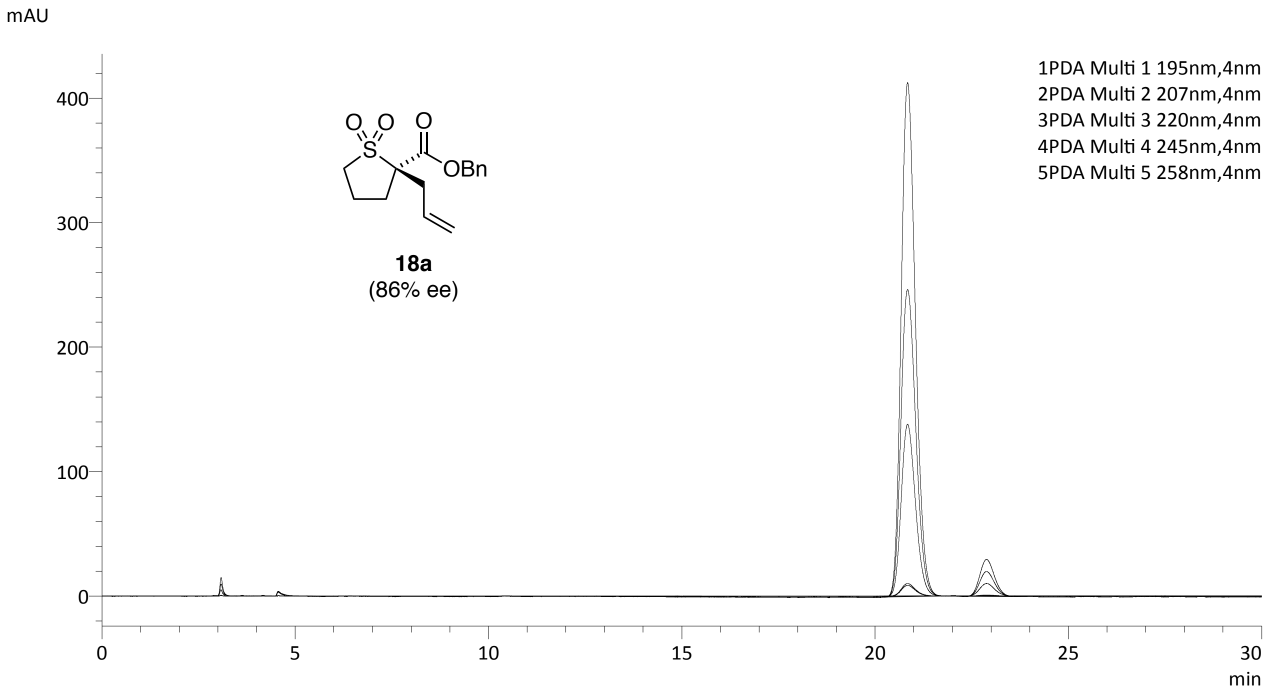

Peak Table

PDA Ch1 195nm

Name	Peak#	Ret. Time	Area	Area%	Mark
Compound 1	1	20.842	6580594	92.663	
Compound 2	2	22.883	521052	7.337	
	Total		7101646	100.000	

PDA Ch2 207nm

Name	Peak#	Ret. Time	Area	Area%	Mark
Compound 1	1	20.841	10615089	93.009	
Compound 2	2	22.884	797833	6.991	
	Total		11412922	100.000	

PDA Ch3 220nm

Name	Peak#	Ret. Time	Area	Area%	Mark
Compound 1	1	20.841	3544269	92.960	
Compound 2	2	22.884	268426	7.040	
	Total		3812696	100.000	

PDA Ch4 245nm

Name	Peak#	Ret. Time	Area	Area%	Mark
Compound 1	1	20.842	216518	93.926	
Compound 2	2	22.886	14001	6.074	
	Total		230520	100.000	

PDA Ch5 258nm

Name	Peak#	Ret. Time	Area	Area%	Mark
Compound 1	1	20.843	250282	93.744	
Compound 2	2	22.878	16702	6.256	
	Total		266984	100.000	

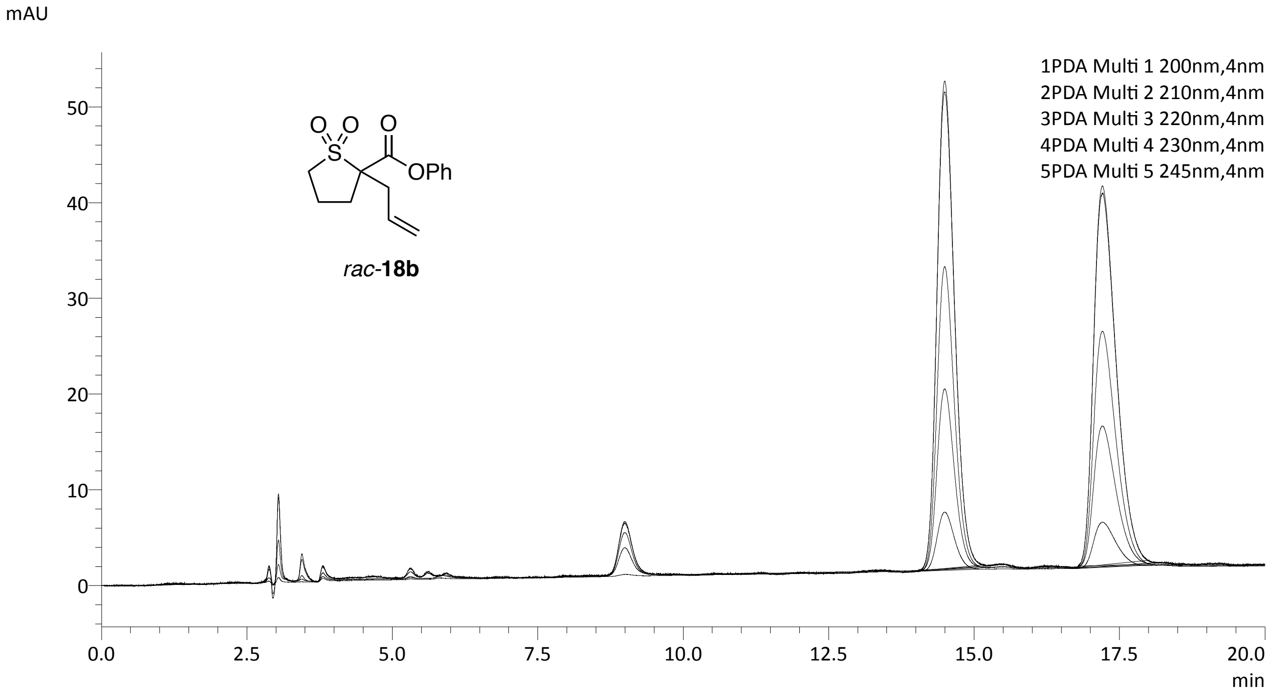

Peak Table

PDA Ch1 200nm

Name	Peak#	Ret. Time	Area	Area%	Mark
	1	14.496	1055507	50.081	M
	2	17.206	1052095	49.919	M
	Total		2107602	100.000	

PDA Ch2 210nm

Name	Peak#	Ret. Time	Area	Area%	Mark
	1	14.496	1079097	49.965	M
	2	17.206	1080602	50.035	M
	Total		2159699	100.000	

PDA Ch3 220nm

Name	Peak#	Ret. Time	Area	Area%	Mark
	1	14.495	666365	50.052	M
	2	17.206	664983	49.948	M
	Total		1331349	100.000	

PDA Ch4 230nm

Name	Peak#	Ret. Time	Area	Area%	Mark
	1	14.496	392858	50.043	M
	2	17.207	392187	49.957	M
	Total		785045	100.000	

PDA Ch5 245nm

Name	Peak#	Ret. Time	Area	Area%	Mark
	1	14.497	127130	50.196	M
	2	17.208	126135	49.804	M
	Total		253265	100.000	

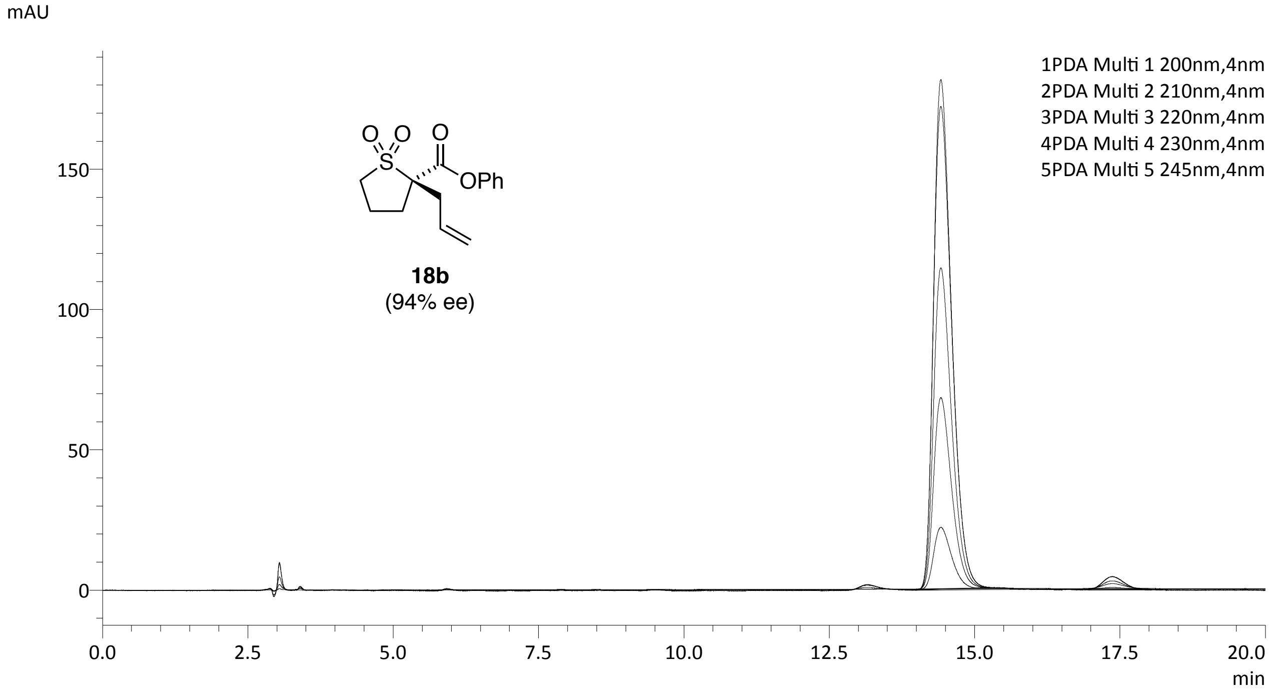

Peak Table

PDA Ch1 200nm

Name	Peak#	Ret. Time	Area	Area%	Mark
	1	14.420	3779047	96.894	M
	2	17.378	121146	3.106	SV
	Total		3900193	100.000	

PDA Ch2 210nm

Name	Peak#	Ret. Time	Area	Area%	Mark
	1	14.420	3982461	97.011	S
	2	17.366	122720	2.989	
	Total		4105180	100.000	

PDA Ch3 220nm

Name	Peak#	Ret. Time	Area	Area%	Mark
	1	14.420	2475480	96.999	
	2	17.364	76580	3.001	
	Total		2552060	100.000	

PDA Ch4 230nm

Name	Peak#	Ret. Time	Area	Area%	Mark
	1	14.420	1471840	97.091	
	2	17.370	44099	2.909	
	Total		1515939	100.000	

PDA Ch5 245nm

Name	Peak#	Ret. Time	Area	Area%	Mark
	1	14.420	476872	97.445	
	2	17.372	12504	2.555	
	Total		489376	100.000	

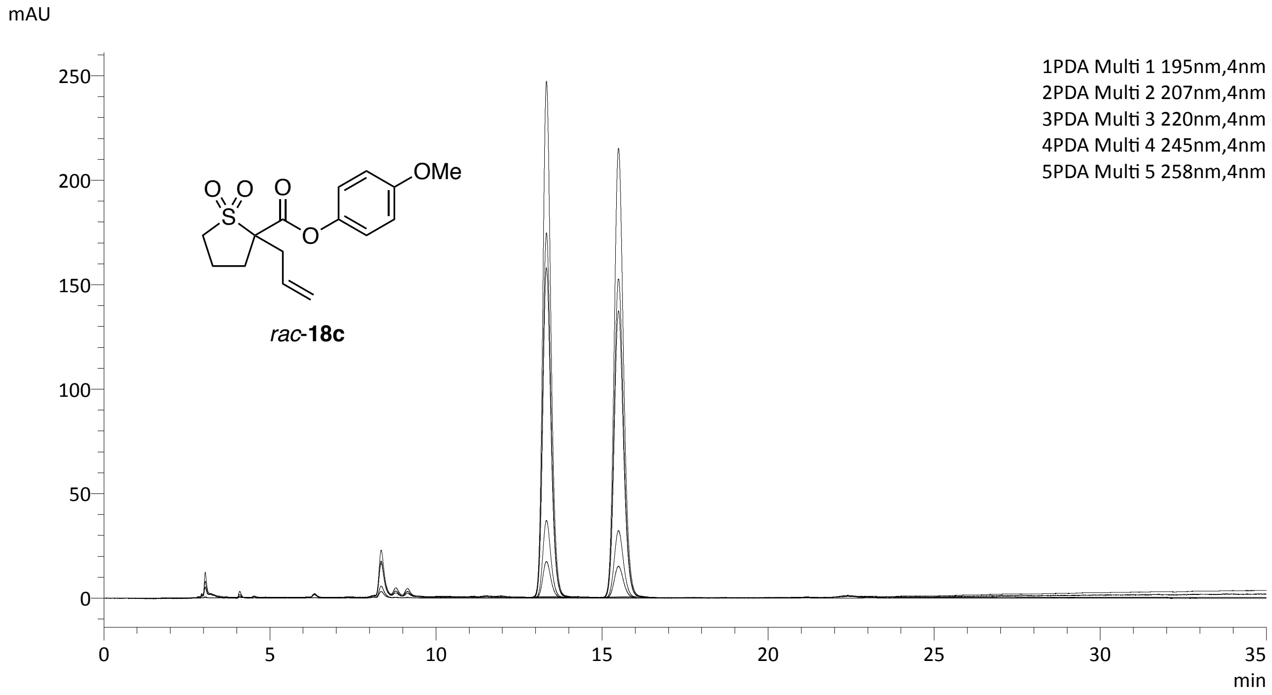

Peak Table

PDA Ch1 195nm

Name	Peak#	Ret. Time	Area	Area%	Mark
	1	13.326	3037247	49.743	M
	2	15.497	3068576	50.257	M
	Total		6105823	100.000	

PDA Ch2 207nm

Name	Peak#	Ret. Time	Area	Area%	Mark
	1	13.326	4234095	49.997	
	2	15.496	4234526	50.003	
	Total		8468620	100.000	

PDA Ch3 220nm

Name	Peak#	Ret. Time	Area	Area%	Mark
	1	13.326	2705486	49.926	
	2	15.496	2713531	50.074	
	Total		5419017	100.000	

PDA Ch4 245nm

Name	Peak#	Ret. Time	Area	Area%	Mark
	1	13.326	636160	50.054	
	2	15.496	634778	49.946	
	Total		1270938	100.000	

PDA Ch5 258nm

Name	Peak#	Ret. Time	Area	Area%	Mark
	1	13.327	300536	50.034	
	2	15.496	300128	49.966	
	Total		600664	100.000	

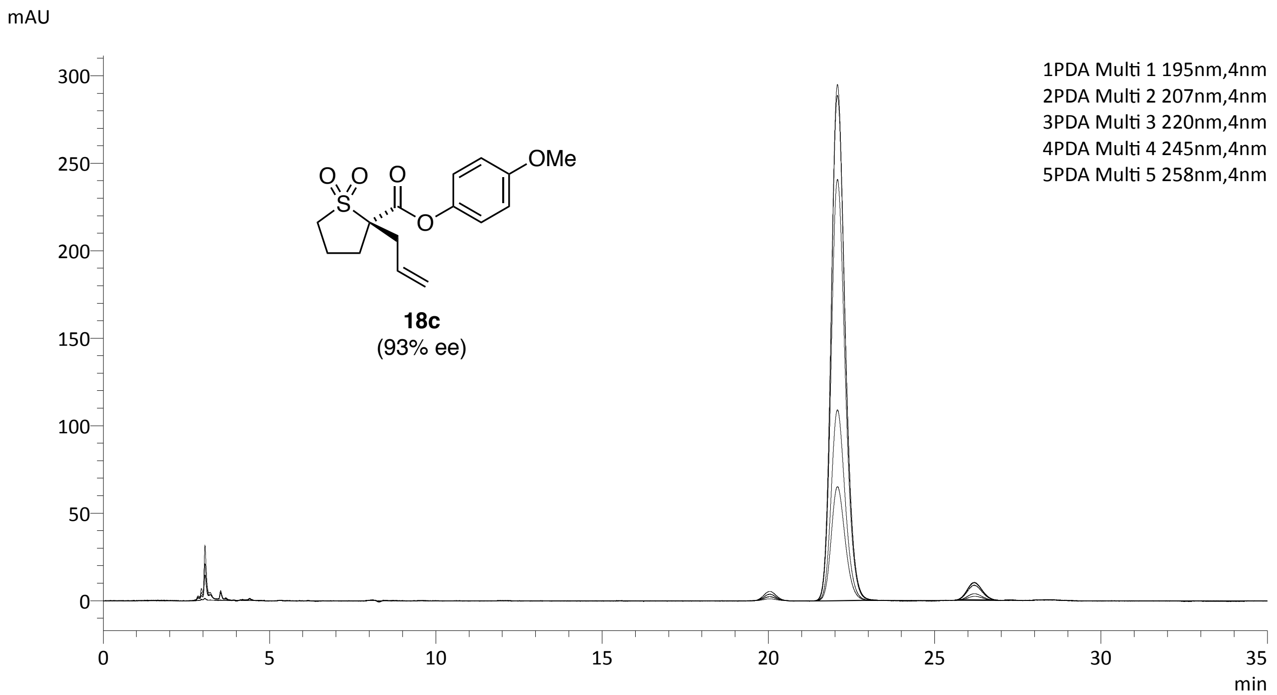

Peak Table

PDA Ch1 195nm

Name	Peak#	Ret. Time	Area	Area%	Mark
Compound 2	1	22.077	7171023	96.526	M
	2	26.187	258122	3.474	
	Total		7429145	100.000	

PDA Ch2 207nm

Name	Peak#	Ret. Time	Area	Area%	Mark
Compound 2	1	22.076	8483804	96.664	
	2	26.190	292808	3.336	
	Total		8776612	100.000	

PDA Ch3 220nm

Name	Peak#	Ret. Time	Area	Area%	Mark
Compound 2	1	22.076	8692698	96.344	M
	2	26.188	329823	3.656	
	Total		9022521	100.000	

PDA Ch4 245nm

Name	Peak#	Ret. Time	Area	Area%	Mark
Compound 2	1	22.076	3204454	96.282	
	2	26.196	123726	3.718	
	Total		3328180	100.000	

PDA Ch5 258nm

Name	Peak#	Ret. Time	Area	Area%	Mark
Compound 2	1	22.077	1917101	96.279	
	2	26.199	74100	3.721	
	Total		1991202	100.000	

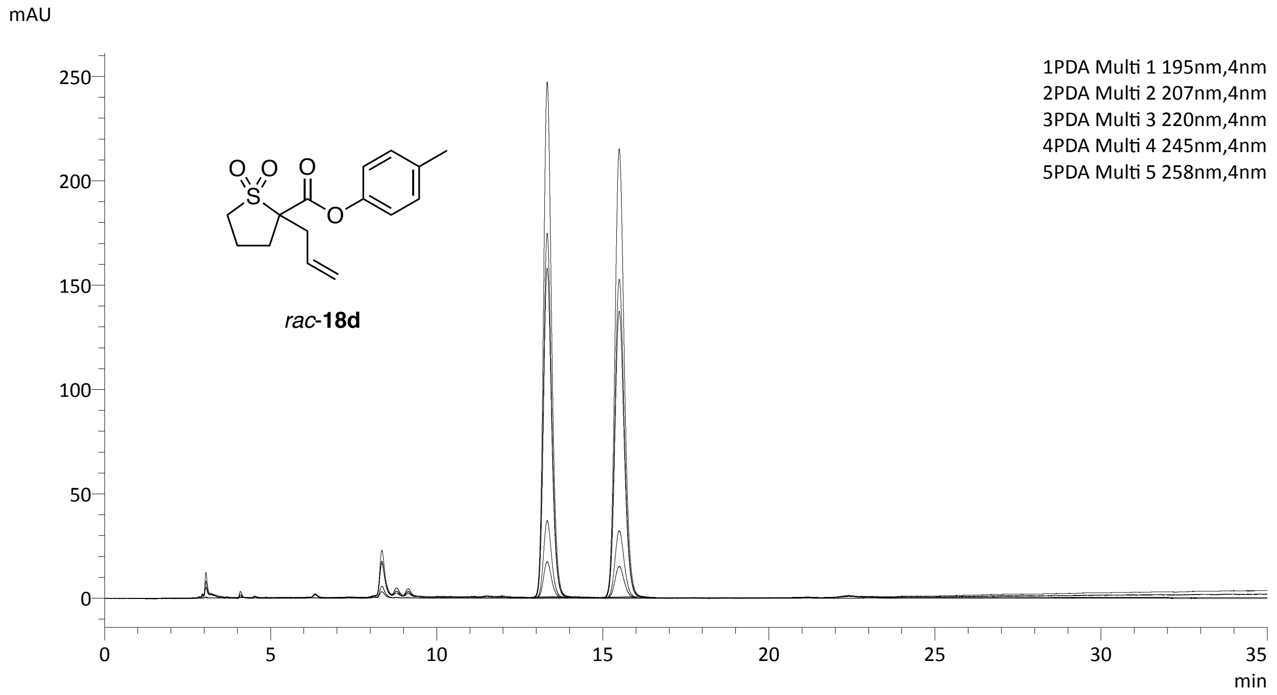

Peak Table

PDA Ch1 195nm

Name	Peak#	Ret. Time	Area	Area%	Mark
	1	13.326	3037247	49.743	M
	2	15.497	3068576	50.257	M
	Total		6105823	100.000	

PDA Ch2 207nm

Name	Peak#	Ret. Time	Area	Area%	Mark
	1	13.326	4234095	49.997	
	2	15.496	4234526	50.003	
	Total		8468620	100.000	

PDA Ch3 220nm

Name	Peak#	Ret. Time	Area	Area%	Mark
	1	13.326	2705486	49.926	
	2	15.496	2713531	50.074	
	Total		5419017	100.000	

PDA Ch4 245nm

Name	Peak#	Ret. Time	Area	Area%	Mark
	1	13.326	636160	50.054	
	2	15.496	634778	49.946	
	Total		1270938	100.000	

PDA Ch5 258nm

Name	Peak#	Ret. Time	Area	Area%	Mark
	1	13.327	300536	50.034	
	2	15.496	300128	49.966	
	Total		600664	100.000	

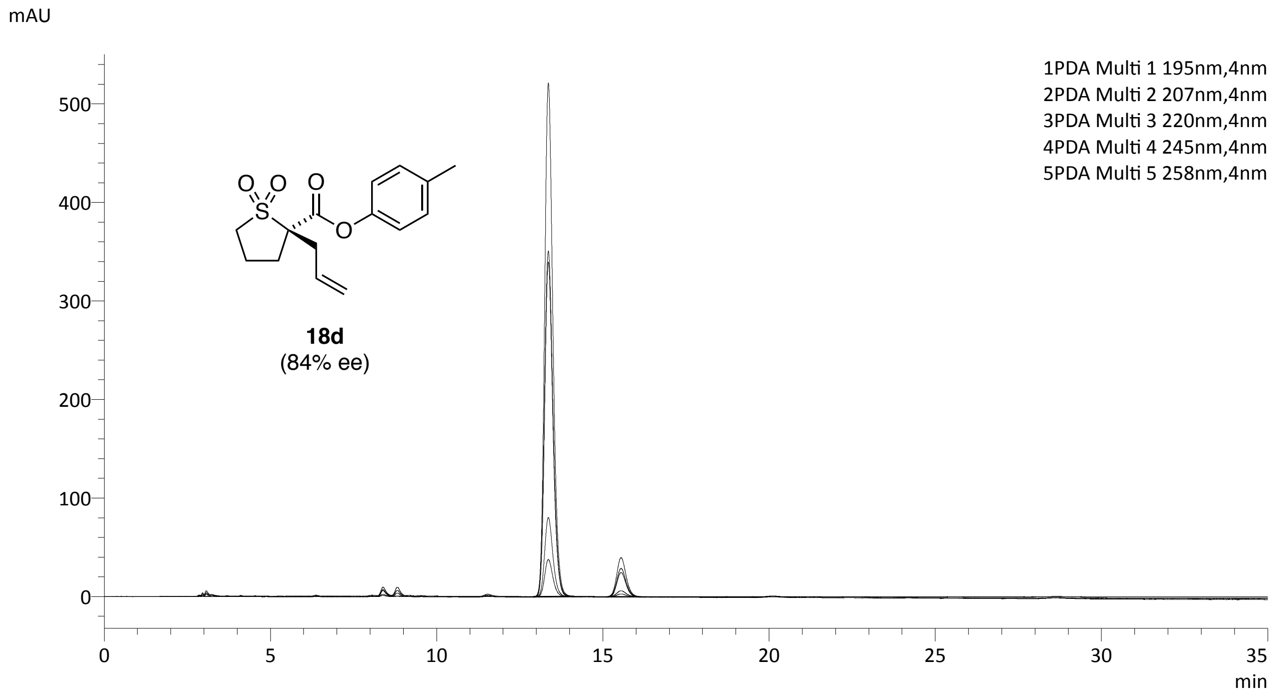

Peak Table

PDA Ch1 195nm

Name	Peak#	Ret. Time	Area	Area%	Mark
	1	13.360	6292246	91.769	
	2	15.547	564397	8.231	
	Total		6856643	100.000	

PDA Ch2 207nm

Name	Peak#	Ret. Time	Area	Area%	Mark
	1	13.360	9092207	92.144	
	2	15.548	775176	7.856	
	Total		9867383	100.000	

PDA Ch3 220nm

Name	Peak#	Ret. Time	Area	Area%	Mark
	1	13.360	5882312	92.232	
	2	15.548	495410	7.768	
	Total		6377721	100.000	

PDA Ch4 245nm

Name	Peak#	Ret. Time	Area	Area%	Mark
	1	13.360	1388113	92.268	
	2	15.548	116322	7.732	
	Total		1504435	100.000	

PDA Ch5 258nm

Name	Peak#	Ret. Time	Area	Area%	Mark
	1	13.361	655103	92.251	
	2	15.548	55032	7.749	
	Total		710135	100.000	

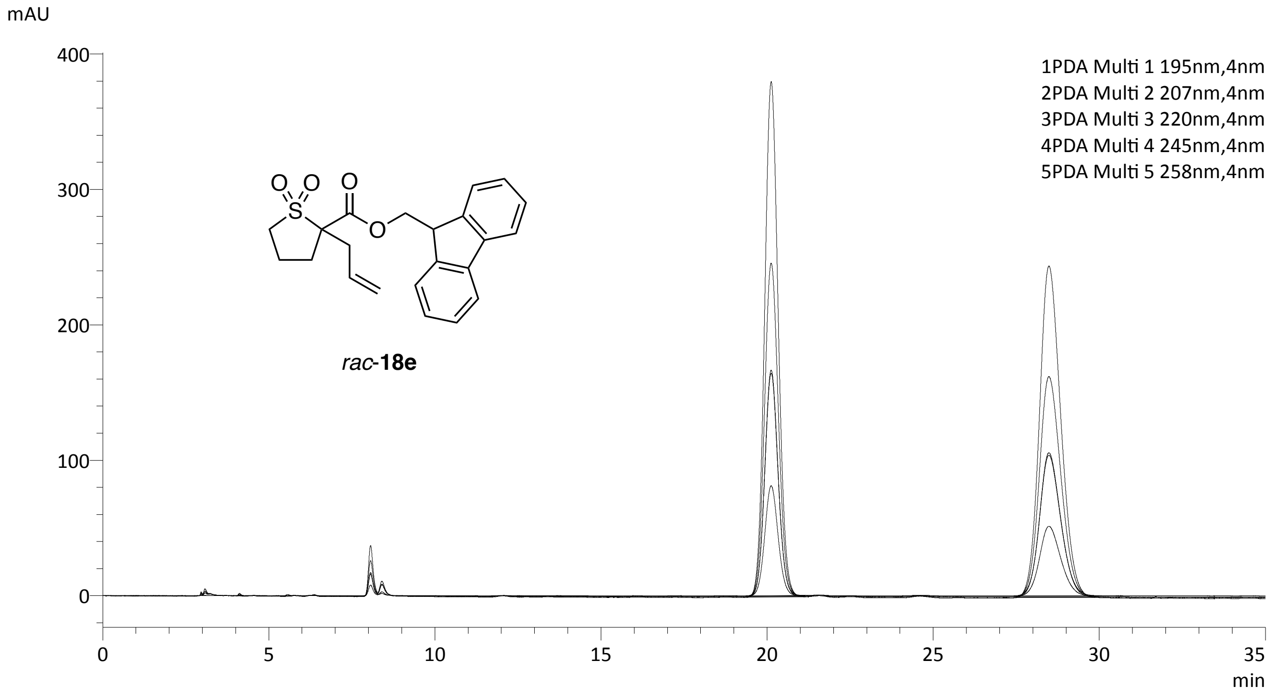

Peak Table

PDA Ch1 195nm

Name	Peak#	Ret. Time	Area	Area%	Mark
Compound 1	1	20.122	7235623	49.281	M
	2	28.484	7446767	50.719	M
	Total		14682391	100.000	

PDA Ch2 207nm

Name	Peak#	Ret. Time	Area	Area%	Mark
Compound 1	1	20.124	10867905	49.715	
	2	28.486	10992404	50.285	
	Total		21860309	100.000	

PDA Ch3 220nm

Name	Peak#	Ret. Time	Area	Area%	Mark
Compound 1	1	20.124	4736592	49.815	
	2	28.485	4771847	50.185	
	Total		9508439	100.000	

PDA Ch4 245nm

Name	Peak#	Ret. Time	Area	Area%	Mark
Compound 1	1	20.124	2291170	49.924	
	2	28.485	2298158	50.076	
	Total		4589328	100.000	

PDA Ch5 258nm

Name	Peak#	Ret. Time	Area	Area%	Mark
Compound 1	1	20.124	4625727	49.962	
	2	28.486	4632839	50.038	
	Total		9258567	100.000	

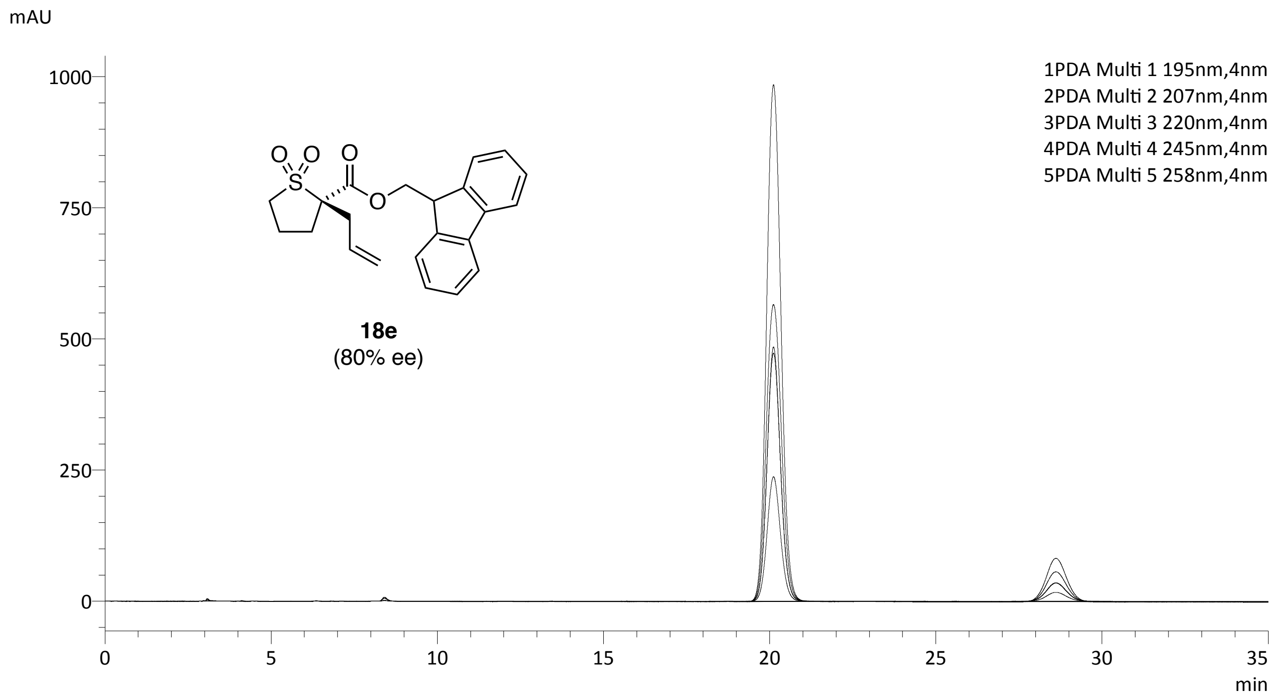

Peak Table

PDA Ch1 195nm

Name	Peak#	Ret. Time	Area	Area%	Mark
Compound 1	1	20.115	18000618	88.030	M
	2	28.607	2447550	11.970	
	Total		20448168	100.000	

PDA Ch2 207nm

Name	Peak#	Ret. Time	Area	Area%	Mark
Compound 1	1	20.116	29563929	89.262	
	2	28.613	3556501	10.738	
	Total		33120430	100.000	

PDA Ch3 220nm

Name	Peak#	Ret. Time	Area	Area%	Mark
Compound 1	1	20.116	13607564	89.768	
	2	28.614	1550987	10.232	
	Total		15158552	100.000	

PDA Ch4 245nm

Name	Peak#	Ret. Time	Area	Area%	Mark
Compound 1	1	20.116	6716380	90.072	
	2	28.613	740293	9.928	
	Total		7456673	100.000	

PDA Ch5 258nm

Name	Peak#	Ret. Time	Area	Area%	Mark
Compound 1	1	20.116	13663781	90.130	
	2	28.614	1496310	9.870	
	Total		15160092	100.000	

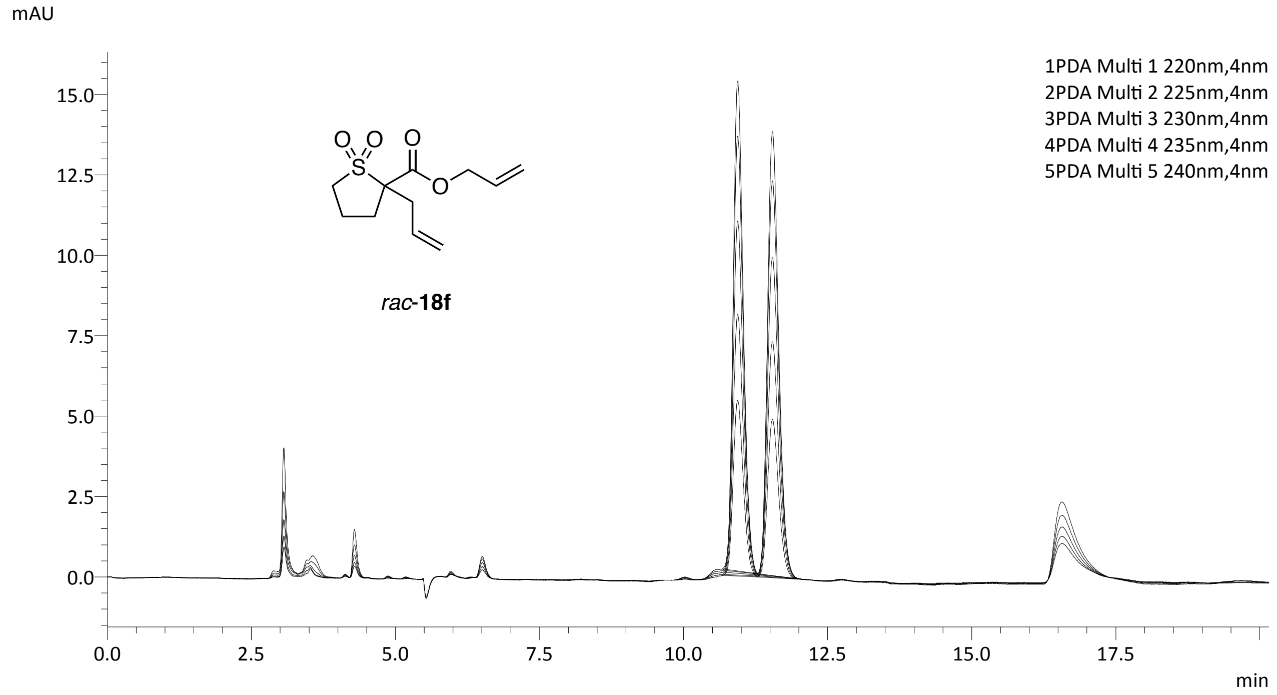

Peak Table

PDA Ch1 220nm

Name	Peak#	Ret. Time	Area	Area%	Mark
	1	10.939	187757	49.952	
	2	11.542	188120	50.048	V
	Total		375877	100.000	

PDA Ch2 225nm

Name	Peak#	Ret. Time	Area	Area%	Mark
	1	10.939	166762	49.923	
	2	11.542	167278	50.077	V
	Total		334040	100.000	

PDA Ch3 230nm

Name	Peak#	Ret. Time	Area	Area%	Mark
	1	10.939	134939	49.955	
	2	11.542	135182	50.045	V
	Total		270120	100.000	

PDA Ch4 235nm

Name	Peak#	Ret. Time	Area	Area%	Mark
	1	10.939	99993	50.084	
	2	11.542	99655	49.916	
	Total		199648	100.000	

PDA Ch5 240nm

Name	Peak#	Ret. Time	Area	Area%	Mark
	1	10.939	67403	50.127	
	2	11.543	67062	49.873	
	Total		134465	100.000	

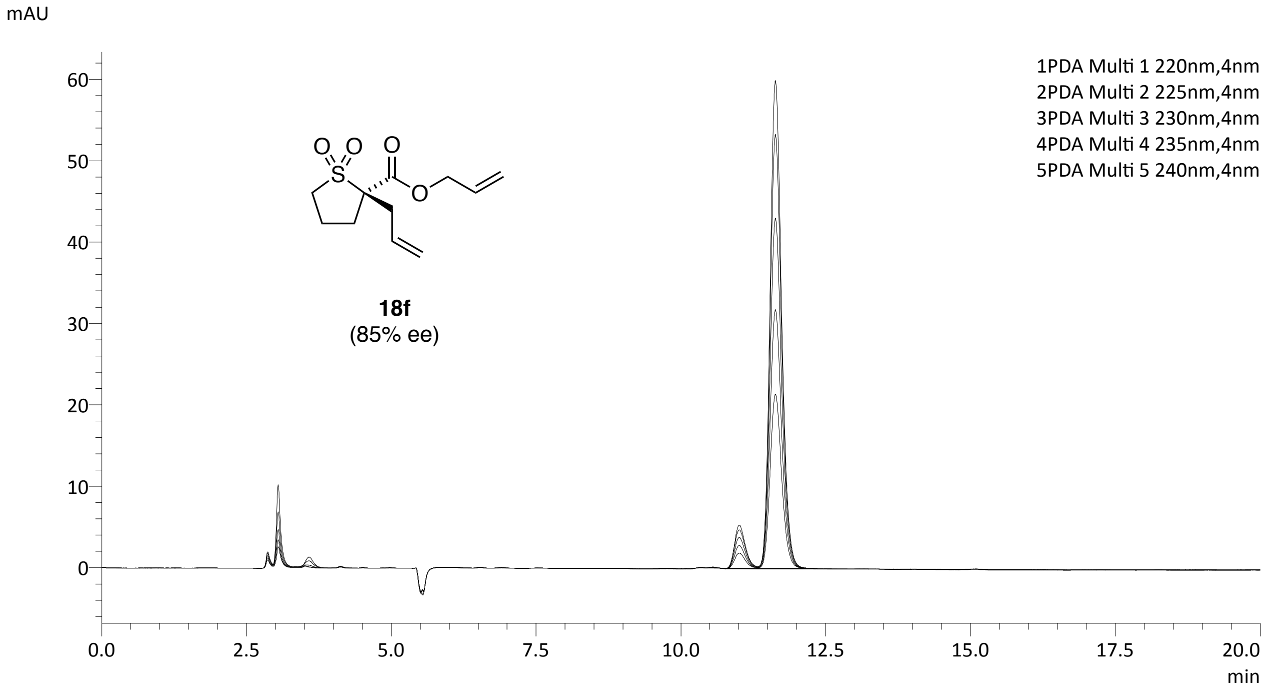

Peak Table

PDA Ch1 220nm

Name	Peak#	Ret. Time	Area	Area%	Mark
	1	11.009	70194	7.718	
	2	11.633	839232	92.282	V
	Total		909426	100.000	

PDA Ch2 225nm

Name	Peak#	Ret. Time	Area	Area%	Mark
	1	11.009	62314	7.714	
	2	11.633	745455	92.286	V
	Total		807769	100.000	

PDA Ch3 230nm

Name	Peak#	Ret. Time	Area	Area%	Mark
	1	11.009	50565	7.736	
	2	11.633	603081	92.264	V
	Total		653647	100.000	

PDA Ch4 235nm

Name	Peak#	Ret. Time	Area	Area%	Mark
	1	11.009	37266	7.713	
	2	11.633	445885	92.287	V
	Total		483151	100.000	

PDA Ch5 240nm

Name	Peak#	Ret. Time	Area	Area%	Mark
	1	11.009	24982	7.684	
	2	11.633	300120	92.316	V
	Total		325102	100.000	

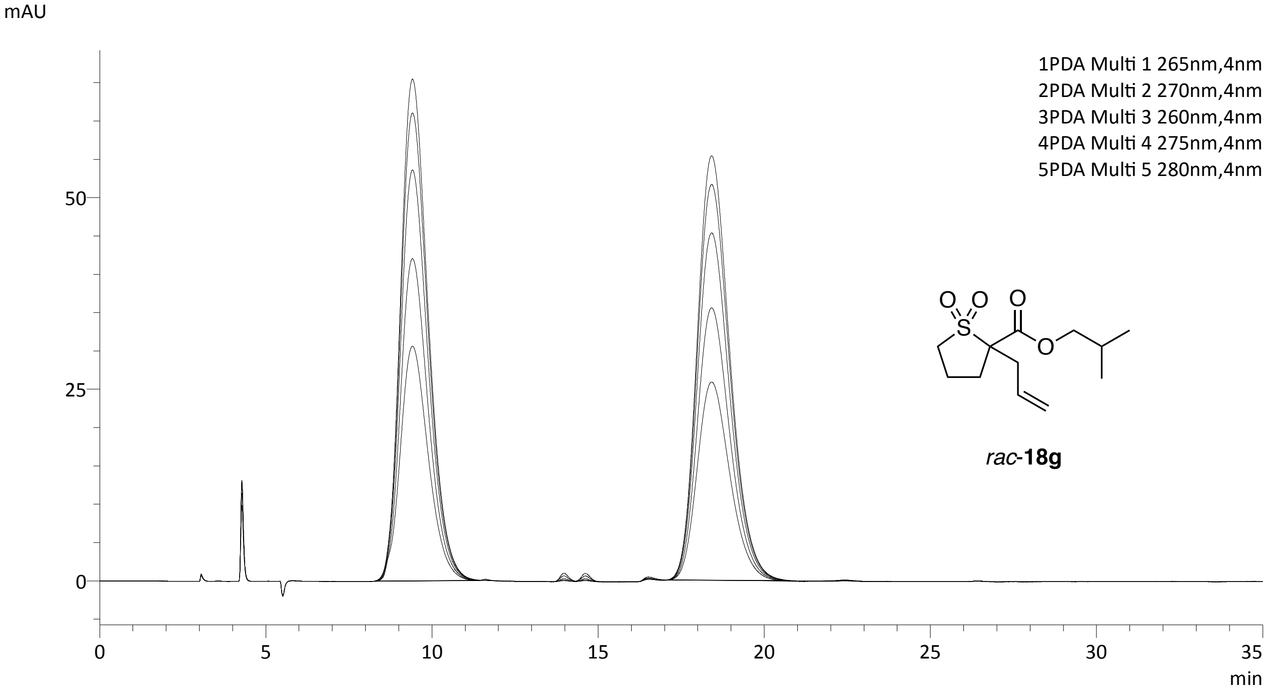

Peak Table

PDA Ch1 265nm

Name	Peak#	Ret. Time	Area	Area%	Mark
	1	9.413	2453492	50.175	
	2	18.416	2436402	49.825	
	Total		4889895	100.000	

PDA Ch2 270nm

Name	Peak#	Ret. Time	Area	Area%	Mark
	1	9.413	3125780	50.134	
	2	18.416	3109124	49.866	
	Total		6234904	100.000	

PDA Ch3 260nm

Name	Peak#	Ret. Time	Area	Area%	Mark
	1	9.414	1794042	50.361	
	2	18.418	1768332	49.639	
	Total		3562374	100.000	

PDA Ch4 275nm

Name	Peak#	Ret. Time	Area	Area%	Mark
	1	9.414	3557795	50.099	
	2	18.417	3543716	49.901	
	Total		7101511	100.000	

PDA Ch5 280nm

Name	Peak#	Ret. Time	Area	Area%	Mark
	1	9.414	3813819	50.095	
	2	18.418	3799295	49.905	
	Total		7613114	100.000	

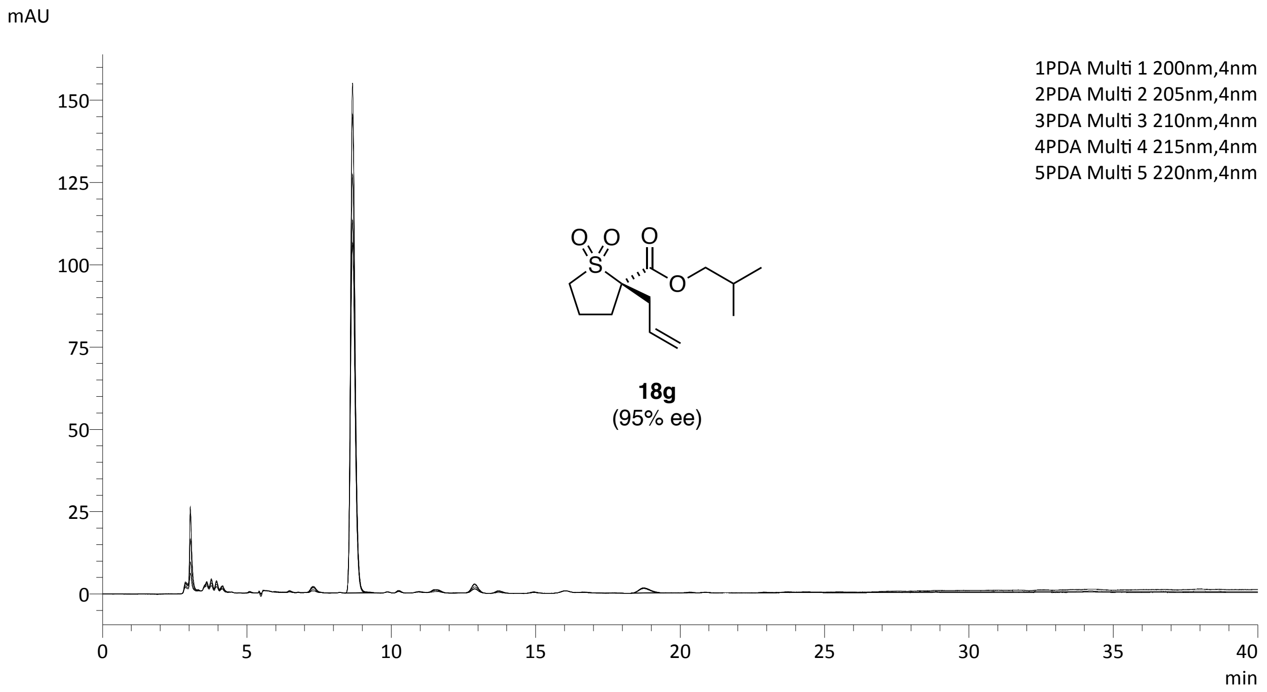

Peak Table

PDA Ch1 200nm

Name	Peak#	Ret. Time	Area	Area%	Mark
	1	8.659	1618823	97.292	S
	2	18.727	45055	2.708	M
	Total		1663878	100.000	

PDA Ch2 205nm

Name	Peak#	Ret. Time	Area	Area%	Mark
	1	8.659	1712615	97.701	
	2	18.761	40296	2.299	M
	Total		1752911	100.000	

PDA Ch3 210nm

Name	Peak#	Ret. Time	Area	Area%	Mark
	1	8.659	1387417	97.318	M
	2	18.741	38240	2.682	
	Total		1425657	100.000	

PDA Ch4 215nm

Name	Peak#	Ret. Time	Area	Area%	Mark
	1	8.659	1236579	96.980	M
	2	18.735	38511	3.020	
	Total		1275090	100.000	

PDA Ch5 220nm

Name	Peak#	Ret. Time	Area	Area%	Mark
	1	8.659	1160265	96.351	
	2	18.732	43941	3.649	
	Total		1204205	100.000	

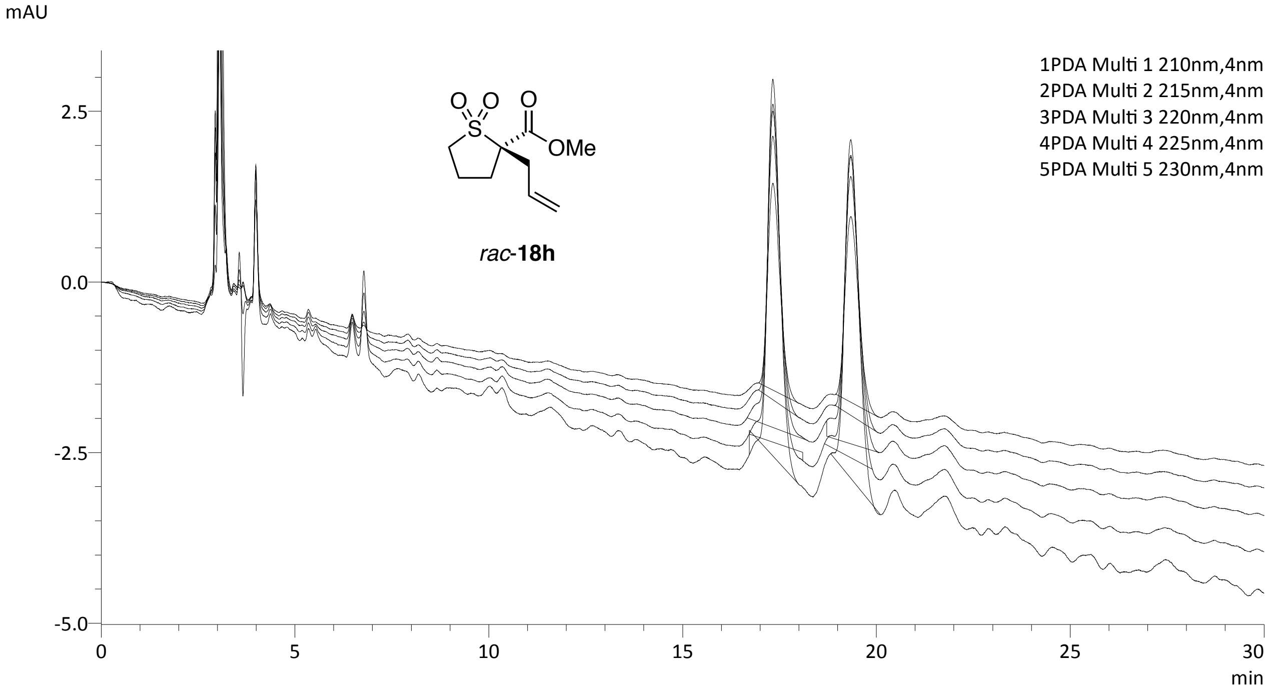

Peak Table

PDA Ch1 210nm

Name	Peak#	Ret. Time	Area	Area%	Mark
	1	17.330	118663	49.087	M
	2	19.340	123079	50.913	M
	Total		241743	100.000	

PDA Ch2 215nm

Name	Peak#	Ret. Time	Area	Area%	Mark
	1	17.331	111417	49.850	M
	2	19.340	112087	50.150	M
	Total		223505	100.000	

PDA Ch3 220nm

Name	Peak#	Ret. Time	Area	Area%	Mark
	1	17.331	109921	49.606	M
	2	19.340	111669	50.394	M
	Total		221590	100.000	

PDA Ch4 225nm

Name	Peak#	Ret. Time	Area	Area%	Mark
	1	17.330	84331	49.602	
	2	19.340	85683	50.398	
	Total		170014	100.000	

PDA Ch5 230nm

Name	Peak#	Ret. Time	Area	Area%	Mark
	1	17.331	66421	49.899	
	2	19.341	66690	50.101	
	Total		133111	100.000	

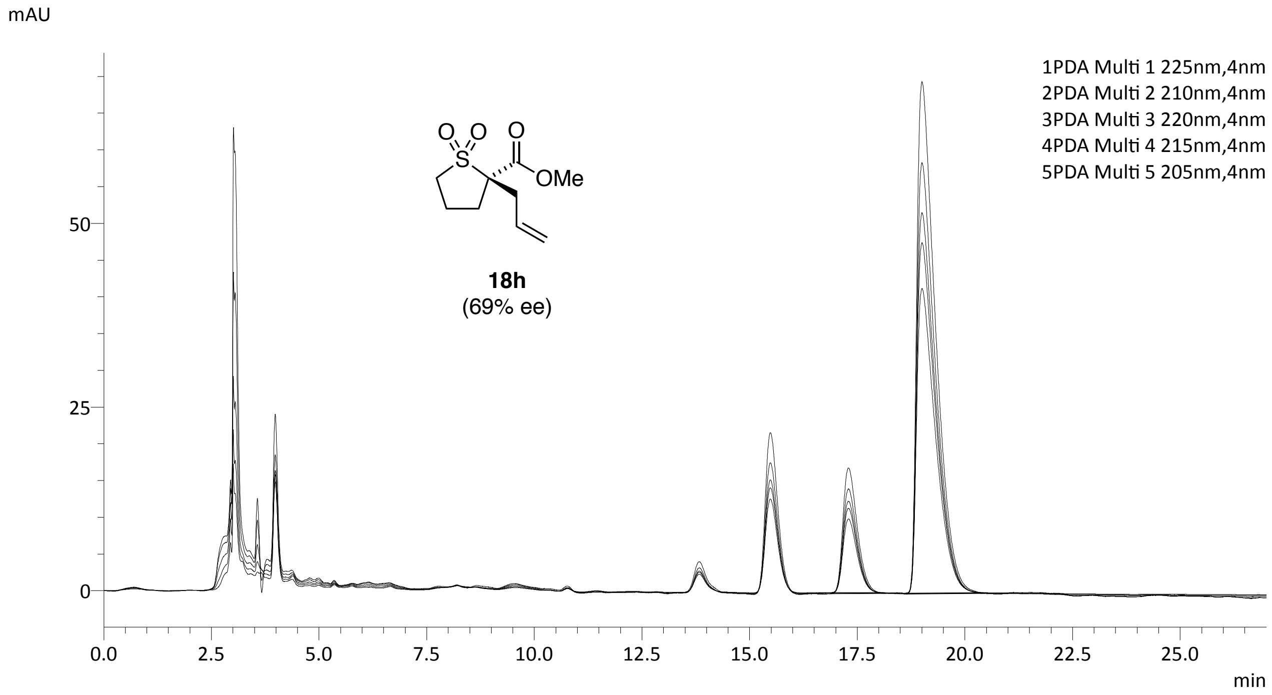

Peak Table

PDA Ch1 225nm

Name	Peak#	Ret. Time	Area	Area%	Mark
	1	17.297	230644	15.533	
	2	19.004	1254228	84.467	
	Total		1484871	100.000	

PDA Ch2 210nm

Name	Peak#	Ret. Time	Area	Area%	Mark
	1	17.297	330845	15.686	
	2	19.004	1778351	84.314	
	Total		2109197	100.000	

PDA Ch3 220nm

Name	Peak#	Ret. Time	Area	Area%	Mark
	1	17.297	266072	15.563	
	2	19.004	1443524	84.437	
	Total		1709596	100.000	

PDA Ch4 215nm

Name	Peak#	Ret. Time	Area	Area%	Mark
	1	17.297	290227	15.611	
	2	19.004	1568848	84.389	
	Total		1859076	100.000	

PDA Ch5 205nm

Name	Peak#	Ret. Time	Area	Area%	Mark
	1	17.297	399603	15.891	
	2	19.004	2115058	84.109	
	Total		2514661	100.000	

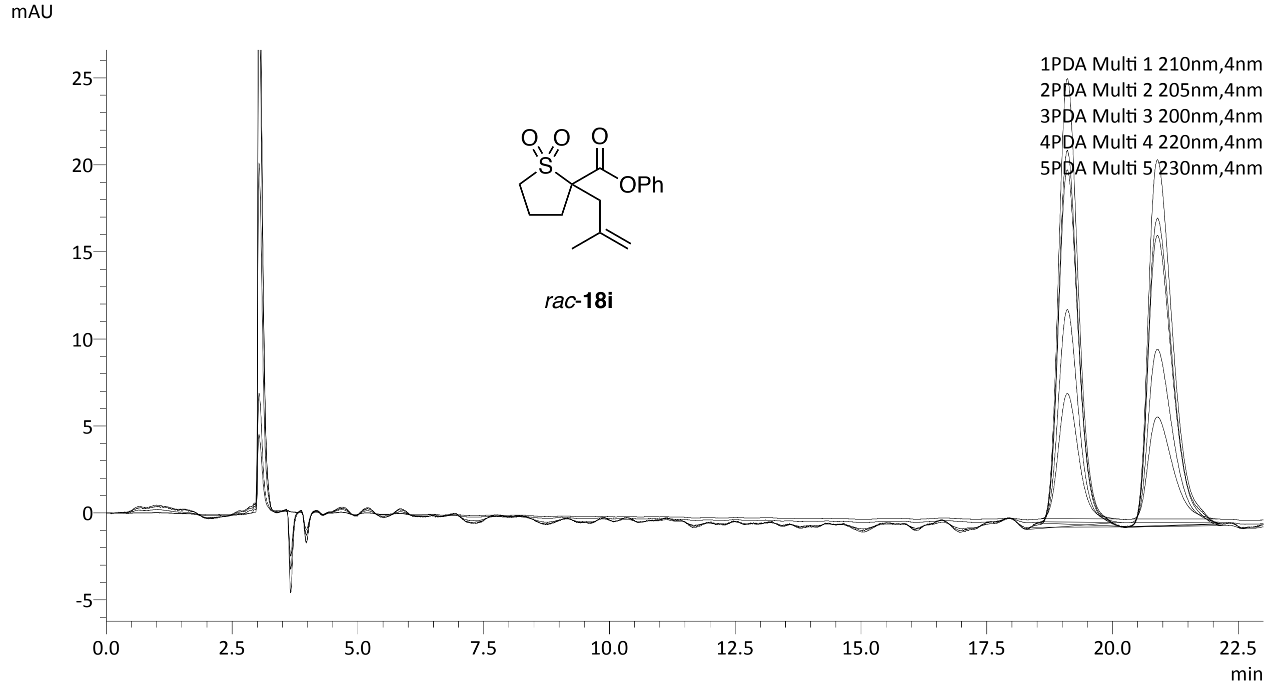

Peak Table

PDA Ch1 210nm

Name	Peak#	Ret. Time	Area	Area%	Mark
	1	19.104	578011	50.418	
	2	20.896	568425	49.582	
	Total		1146436	100.000	

PDA Ch2 205nm

Name	Peak#	Ret. Time	Area	Area%	Mark
	1	19.104	720879	50.138	M
	2	20.897	716910	49.862	M
	Total		1437790	100.000	

PDA Ch3 200nm

Name	Peak#	Ret. Time	Area	Area%	Mark
	1	19.104	599964	49.980	S
	2	20.901	600438	50.020	
	Total		1200402	100.000	

PDA Ch4 220nm

Name	Peak#	Ret. Time	Area	Area%	Mark
	1	19.104	339534	50.203	
	2	20.895	336782	49.797	
	Total		676316	100.000	

PDA Ch5 230nm

Name	Peak#	Ret. Time	Area	Area%	Mark
	1	19.105	199953	50.201	
	2	20.897	198355	49.799	
	Total		398308	100.000	

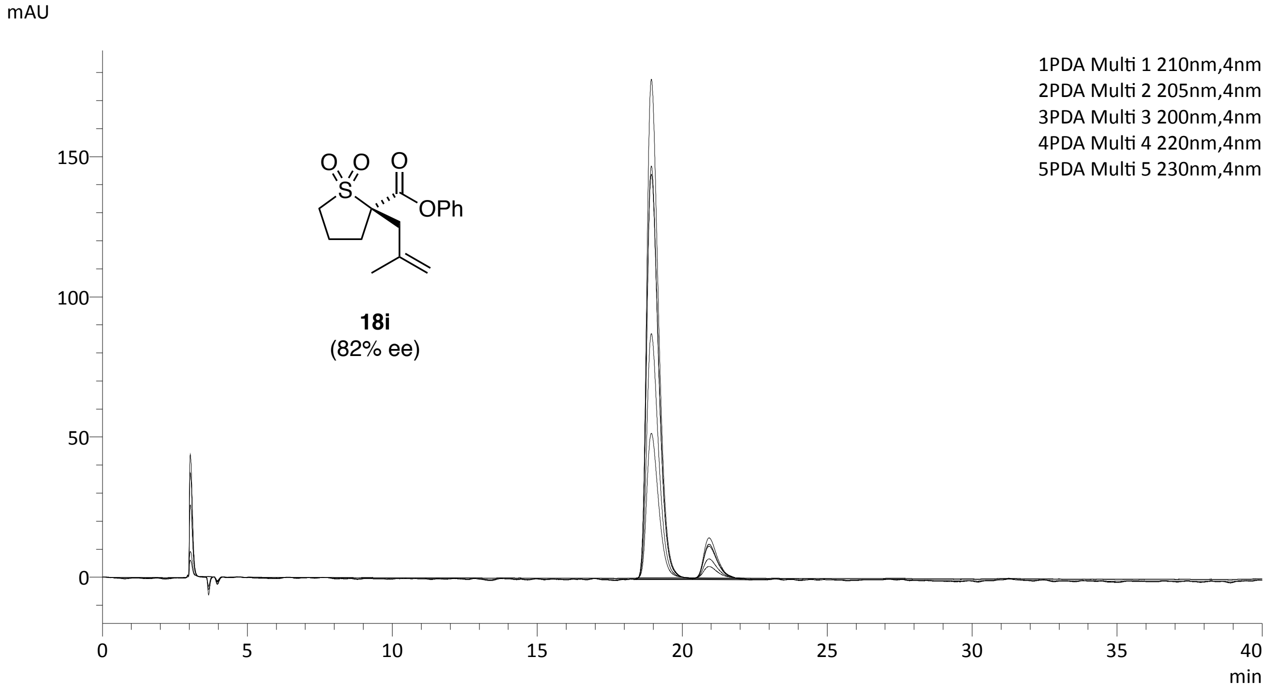

Peak Table

PDA Ch1 210nm

Name	Peak#	Ret. Time	Area	Area%	Mark
	1	18.937	4142882	91.187	
	2	20.928	400380	8.813	V
	Total		4543262	100.000	

PDA Ch2 205nm

Name	Peak#	Ret. Time	Area	Area%	Mark
	1	18.937	5160225	90.656	
	2	20.928	531858	9.344	SV
	Total		5692083	100.000	

PDA Ch3 200nm

Name	Peak#	Ret. Time	Area	Area%	Mark
	1	18.937	4279243	90.959	
	2	20.920	425358	9.041	V
	Total		4704601	100.000	

PDA Ch4 220nm

Name	Peak#	Ret. Time	Area	Area%	Mark
	1	18.937	2497453	91.047	
	2	20.926	245594	8.953	V
	Total		2743046	100.000	

PDA Ch5 230nm

Name	Peak#	Ret. Time	Area	Area%	Mark
	1	18.937	1458880	91.496	M
	2	20.927	135599	8.504	M
	Total		1594479	100.000	

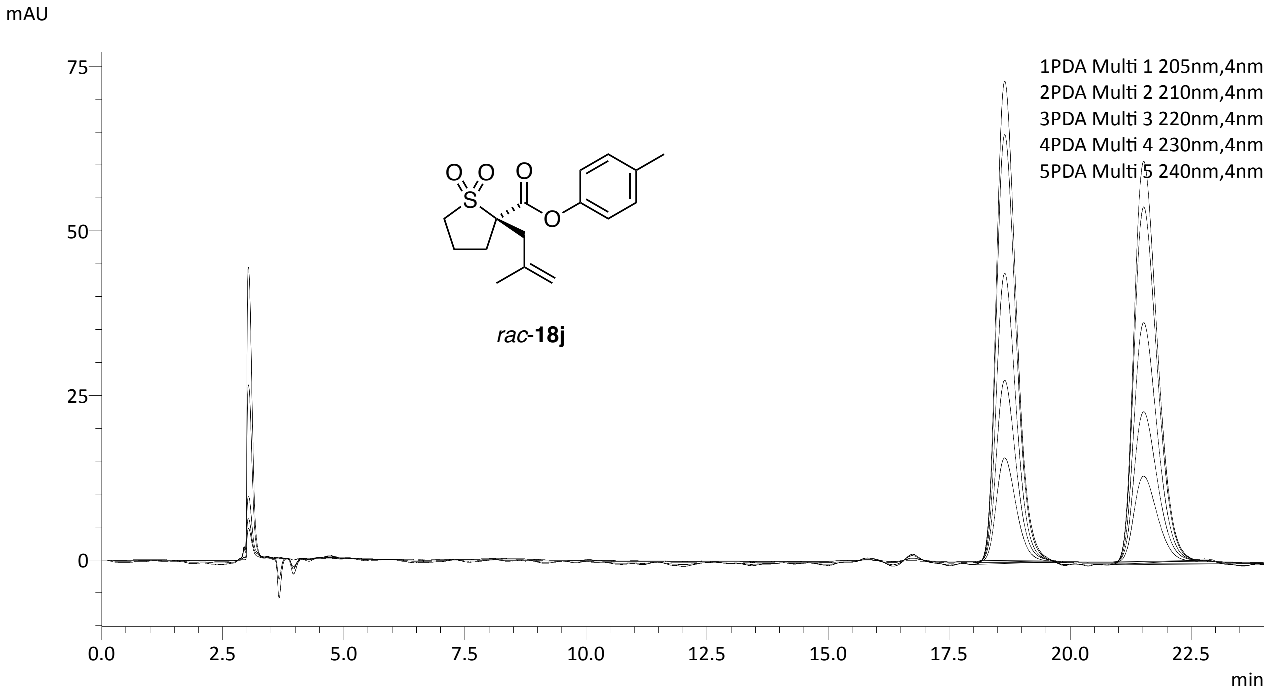

rac-**18j**

Peak Table

PDA Ch1 205nm

Name	Peak#	Ret. Time	Area	Area%	Mark
	1	18.649	2129845	50.020	M
	2	21.518	2128163	49.980	M
	Total		4258007	100.000	

PDA Ch2 210nm

Name	Peak#	Ret. Time	Area	Area%	Mark
	1	18.648	1861533	50.349	M
	2	21.517	1835750	49.651	
	Total		3697284	100.000	

PDA Ch3 220nm

Name	Peak#	Ret. Time	Area	Area%	Mark
	1	18.648	1278810	50.239	
	2	21.517	1266666	49.761	
	Total		2545476	100.000	

PDA Ch4 230nm

Name	Peak#	Ret. Time	Area	Area%	Mark
	1	18.648	785038	50.419	M
	2	21.517	771991	49.581	
	Total		1557028	100.000	

PDA Ch5 240nm

Name	Peak#	Ret. Time	Area	Area%	Mark
	1	18.648	448302	50.364	M
	2	21.517	441826	49.636	
	Total		890127	100.000	

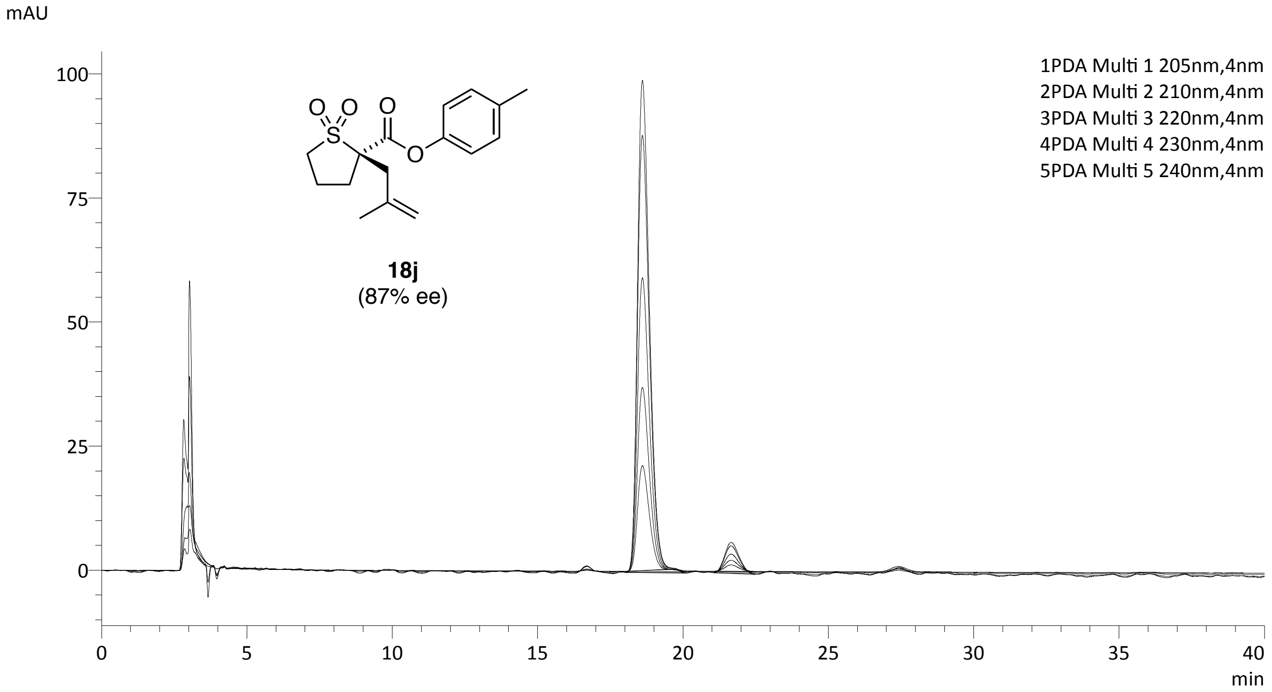

Peak Table

PDA Ch1 205nm

Name	Peak#	Ret. Time	Area	Area%	Mark
	1	18.606	2896495	92.992	
	2	21.653	218268	7.008	
	Total		3114763	100.000	

PDA Ch2 210nm

Name	Peak#	Ret. Time	Area	Area%	Mark
	1	18.606	2556662	93.212	
	2	21.657	186173	6.788	
	Total		2742836	100.000	

PDA Ch3 220nm

Name	Peak#	Ret. Time	Area	Area%	Mark
	1	18.606	1713545	93.421	
	2	21.653	120667	6.579	
	Total		1834212	100.000	

PDA Ch4 230nm

Name	Peak#	Ret. Time	Area	Area%	Mark
	1	18.606	1052191	93.398	
	2	21.655	74370	6.602	
	Total		1126561	100.000	

PDA Ch5 240nm

Name	Peak#	Ret. Time	Area	Area%	Mark
	1	18.606	598004	93.321	
	2	21.657	42802	6.679	
	Total		640806	100.000	

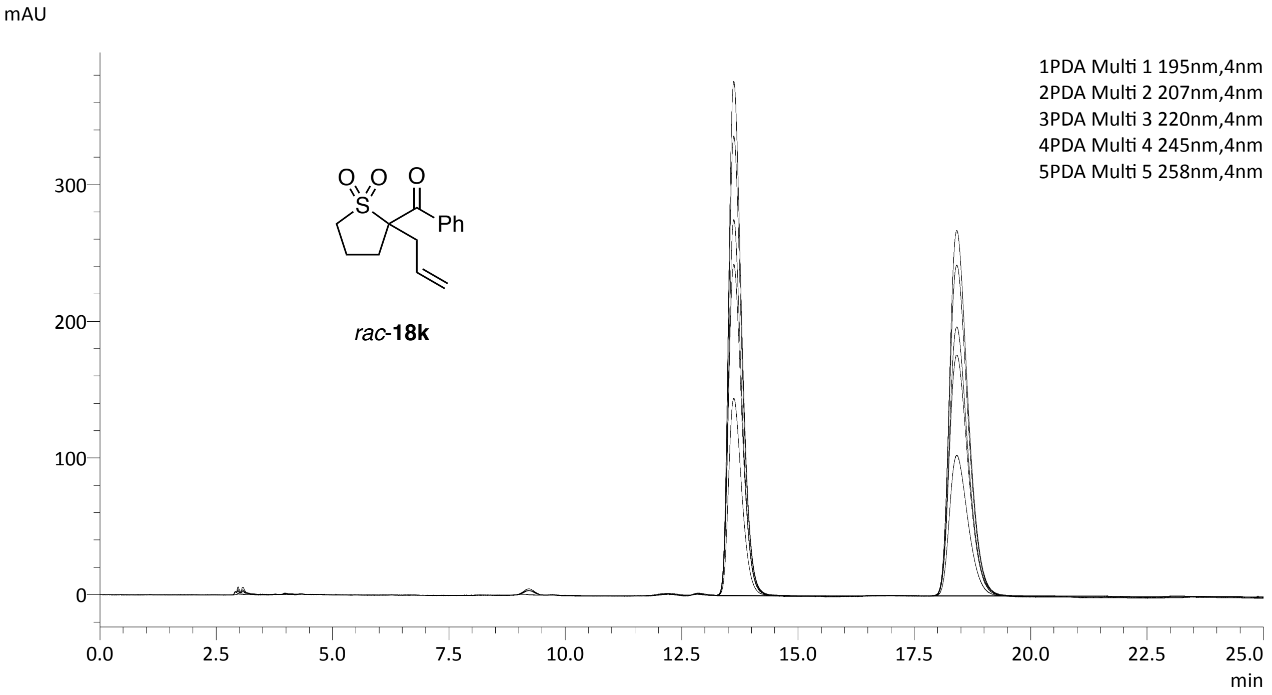

Peak Table

PDA Ch1 195nm

Name	Peak#	Ret. Time	Area	Area%	Mark
	1	13.623	4907723	49.590	
	2	18.412	4988934	50.410	
	Total		9896657	100.000	

PDA Ch2 207nm

Name	Peak#	Ret. Time	Area	Area%	Mark
	1	13.622	6737662	49.699	
	2	18.412	6819252	50.301	
	Total		13556915	100.000	

PDA Ch3 220nm

Name	Peak#	Ret. Time	Area	Area%	Mark
	1	13.622	2886378	49.982	V
	2	18.413	2888514	50.018	
	Total		5774893	100.000	

PDA Ch4 245nm

Name	Peak#	Ret. Time	Area	Area%	Mark
	1	13.622	7464724	49.919	
	2	18.412	7489085	50.081	
	Total		14953809	100.000	

PDA Ch5 258nm

Name	Peak#	Ret. Time	Area	Area%	Mark
	1	13.622	5485883	49.833	
	2	18.412	5522660	50.167	
	Total		11008543	100.000	

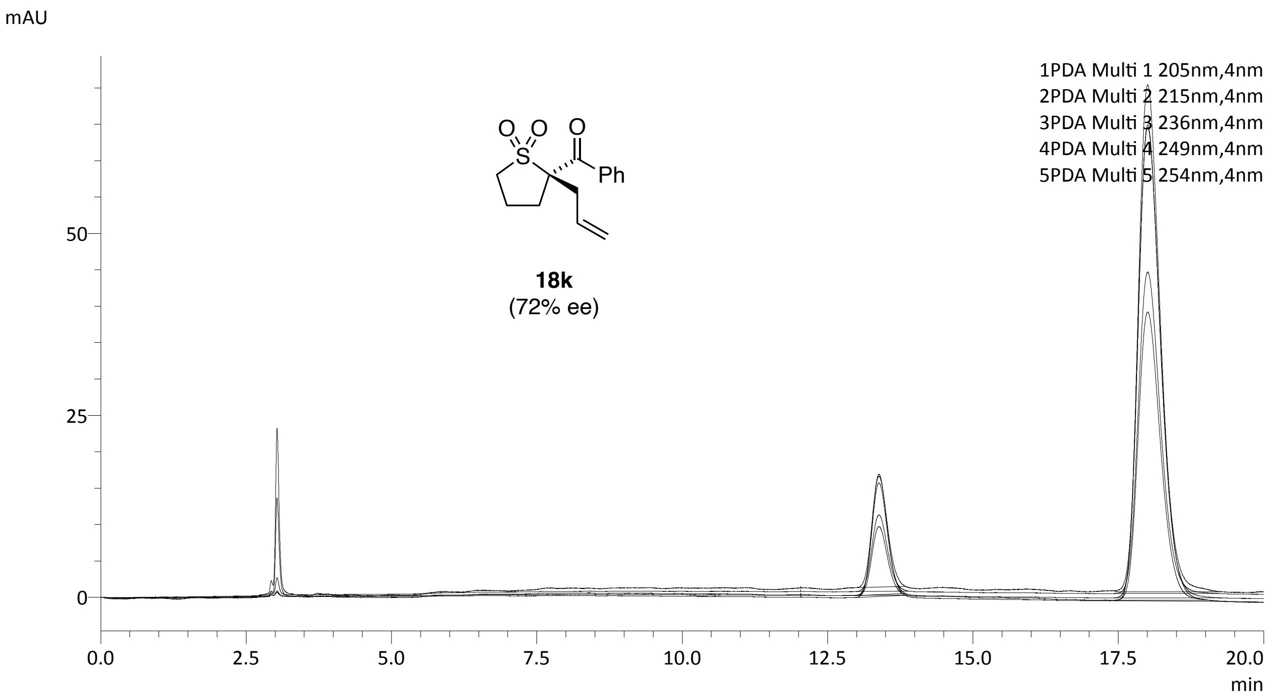

Peak Table

PDA Ch1 205nm

Name	Peak#	Ret. Time	Area	Area%	Mark
	1	13.386	288718	14.145	M
	2	18.007	1752354	85.855	V
	Total		2041073	100.000	

PDA Ch2 215nm

Name	Peak#	Ret. Time	Area	Area%	Mark
	1	13.385	179242	14.363	M
	2	18.006	1068703	85.637	M
	Total		1247945	100.000	

PDA Ch3 236nm

Name	Peak#	Ret. Time	Area	Area%	Mark
	1	13.385	201641	14.436	M
	2	18.006	1195140	85.564	
	Total		1396781	100.000	

PDA Ch4 249nm

Name	Peak#	Ret. Time	Area	Area%	Mark
	1	13.384	317369	14.190	M
	2	18.006	1919222	85.810	
	Total		2236592	100.000	

PDA Ch5 254nm

Name	Peak#	Ret. Time	Area	Area%	Mark
	1	13.385	289757	14.212	M
	2	18.006	1748996	85.788	
	Total		2038754	100.000	

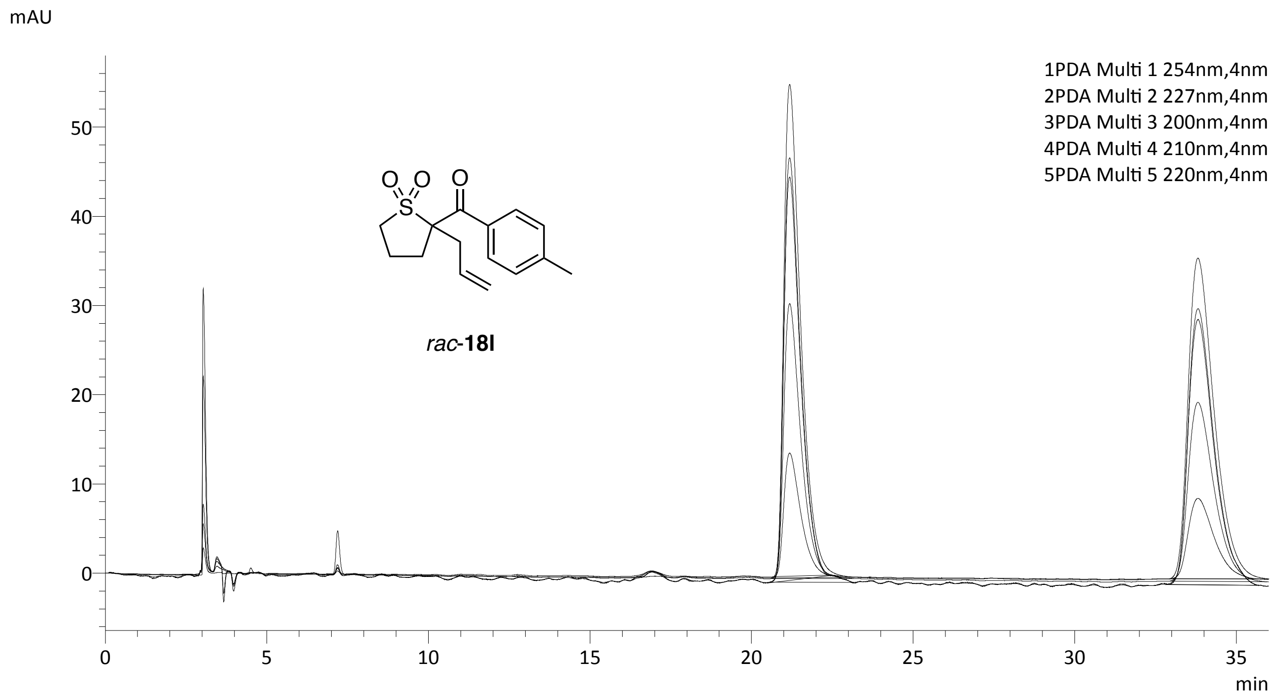

Peak Table

PDA Ch1 254nm

Name	Peak#	Ret. Time	Area	Area%	Mark
	1	21.180	2044803	50.265	
	2	33.815	2023220	49.735	
	Total		4068023	100.000	

PDA Ch2 227nm

Name	Peak#	Ret. Time	Area	Area%	Mark
	1	21.180	502134	49.950	
	2	33.816	503131	50.050	
	Total		1005265	100.000	

PDA Ch3 200nm

Name	Peak#	Ret. Time	Area	Area%	Mark
	1	21.181	1640861	49.711	
	2	33.822	1659963	50.289	
	Total		3300823	100.000	

PDA Ch4 210nm

Name	Peak#	Ret. Time	Area	Area%	Mark
	1	21.179	1774063	50.626	
	2	33.818	1730198	49.374	
	Total		3504261	100.000	

PDA Ch5 220nm

Name	Peak#	Ret. Time	Area	Area%	Mark
	1	21.181	1145113	50.319	
	2	33.817	1130611	49.681	
	Total		2275724	100.000	

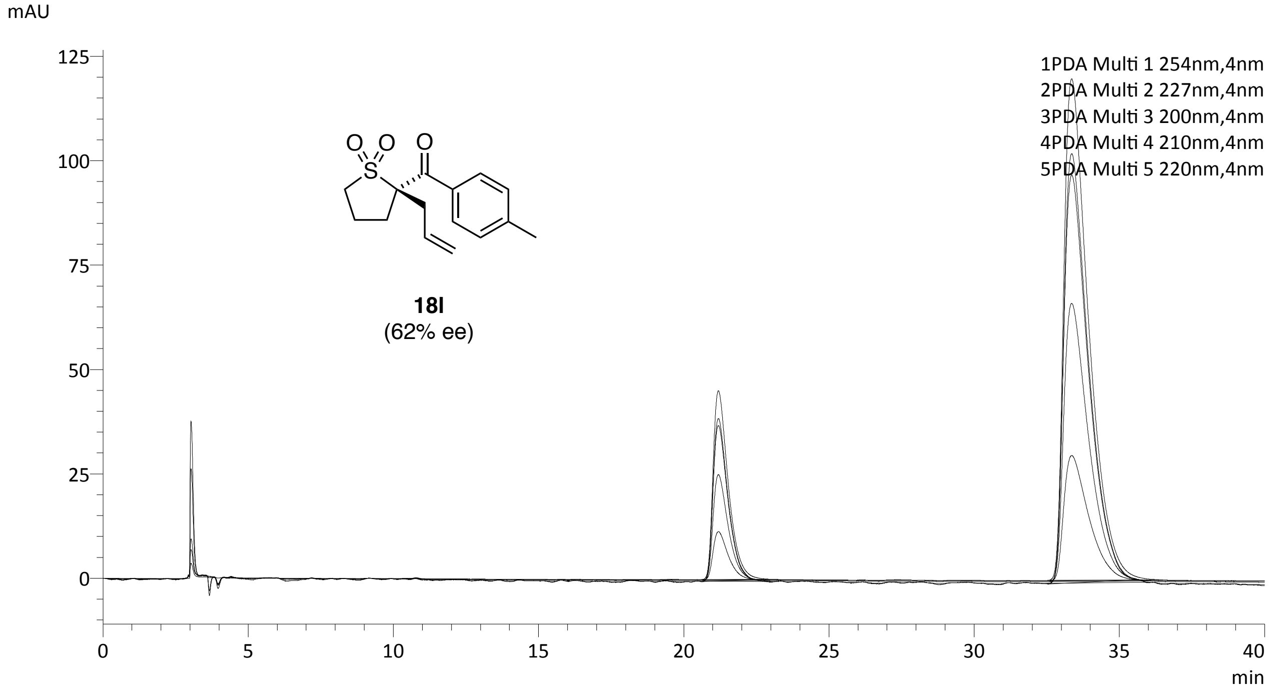

Peak Table

PDA Ch1 254nm

Name	Peak#	Ret. Time	Area	Area%	Mark
	1	21.193	1656494	18.718	
	2	33.358	7193028	81.282	
	Total		8849522	100.000	

PDA Ch2 227nm

Name	Peak#	Ret. Time	Area	Area%	Mark
	1	21.194	420115	18.900	
	2	33.360	1802727	81.100	
	Total		2222842	100.000	

PDA Ch3 200nm

Name	Peak#	Ret. Time	Area	Area%	Mark
	1	21.196	1333343	18.652	M
	2	33.358	5815244	81.348	M
	Total		7148587	100.000	

PDA Ch4 210nm

Name	Peak#	Ret. Time	Area	Area%	Mark
	1	21.193	1432633	18.839	
	2	33.360	6171832	81.161	
	Total		7604464	100.000	

PDA Ch5 220nm

Name	Peak#	Ret. Time	Area	Area%	Mark
	1	21.193	930910	18.895	
	2	33.359	3995913	81.105	
	Total		4926823	100.000	

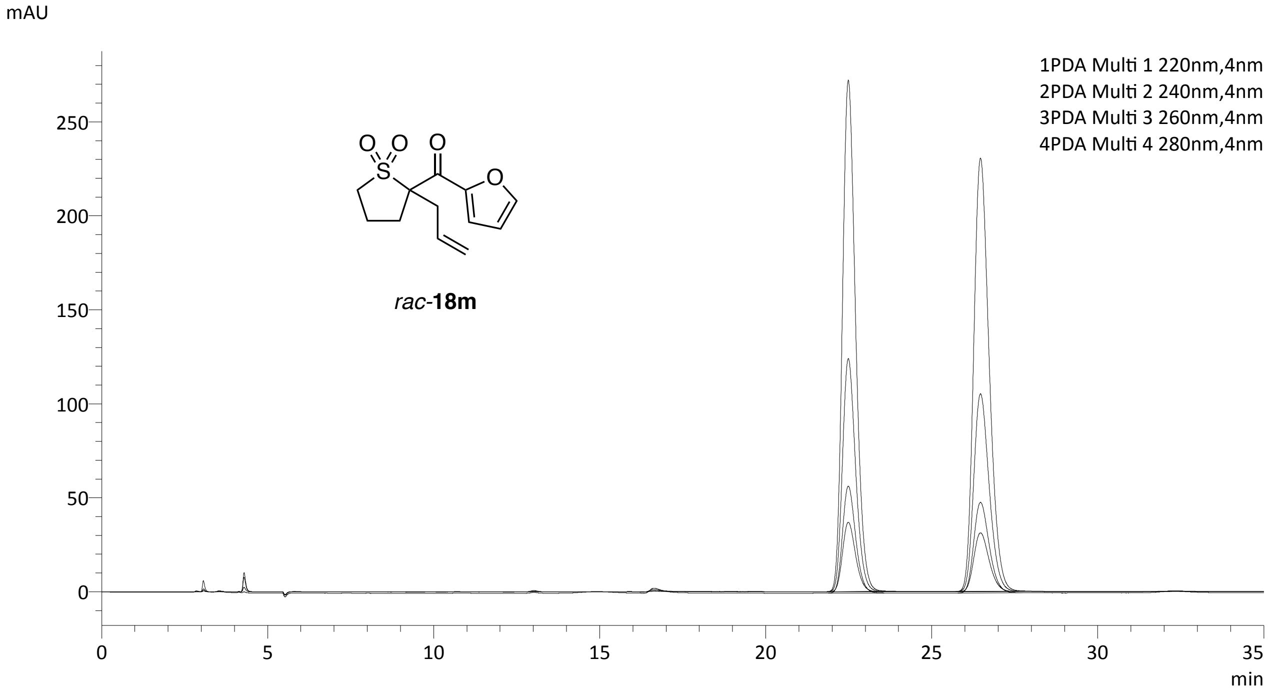

Peak Table

PDA Ch1 220nm

Name	Peak#	Ret. Time	Area	Area%	Mark
	1	22.488	1563785	50.018	
	2	26.469	1562671	49.982	
	Total		3126456	100.000	

PDA Ch2 240nm

Name	Peak#	Ret. Time	Area	Area%	Mark
	1	22.488	1016757	50.049	
	2	26.470	1014785	49.951	
	Total		2031542	100.000	

PDA Ch3 260nm

Name	Peak#	Ret. Time	Area	Area%	Mark
	1	22.488	3413608	49.998	
	2	26.469	3413893	50.002	
	Total		6827500	100.000	

PDA Ch4 280nm

Name	Peak#	Ret. Time	Area	Area%	Mark
	1	22.488	7478220	50.004	
	2	26.469	7477071	49.996	
	Total		14955291	100.000	

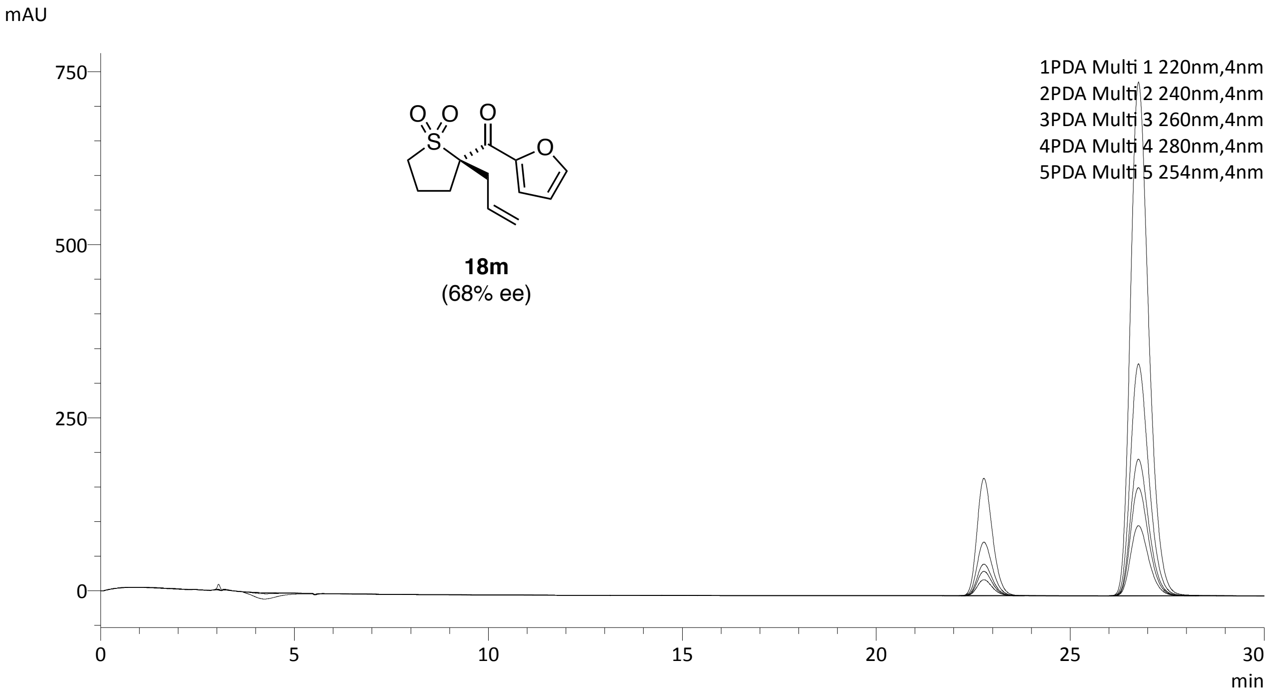

Peak Table

PDA Ch1 220nm

Name	Peak#	Ret. Time	Area	Area%	Mark
	1	22.777	979450	16.027	
	2	26.762	5131939	83.973	
	Total		6111389	100.000	

PDA Ch2 240nm

Name	Peak#	Ret. Time	Area	Area%	Mark
	1	22.777	636995	16.063	
	2	26.762	3328675	83.937	
	Total		3965670	100.000	

PDA Ch3 260nm

Name	Peak#	Ret. Time	Area	Area%	Mark
	1	22.777	2137783	16.205	
	2	26.762	11053930	83.795	
	Total		13191713	100.000	

PDA Ch4 280nm

Name	Peak#	Ret. Time	Area	Area%	Mark
	1	22.777	4689340	16.117	
	2	26.762	24406746	83.883	
	Total		29096087	100.000	

PDA Ch5 254nm

Name	Peak#	Ret. Time	Area	Area%	Mark
	1	22.777	1258058	16.193	
	2	26.762	6511250	83.807	
	Total		7769309	100.000	

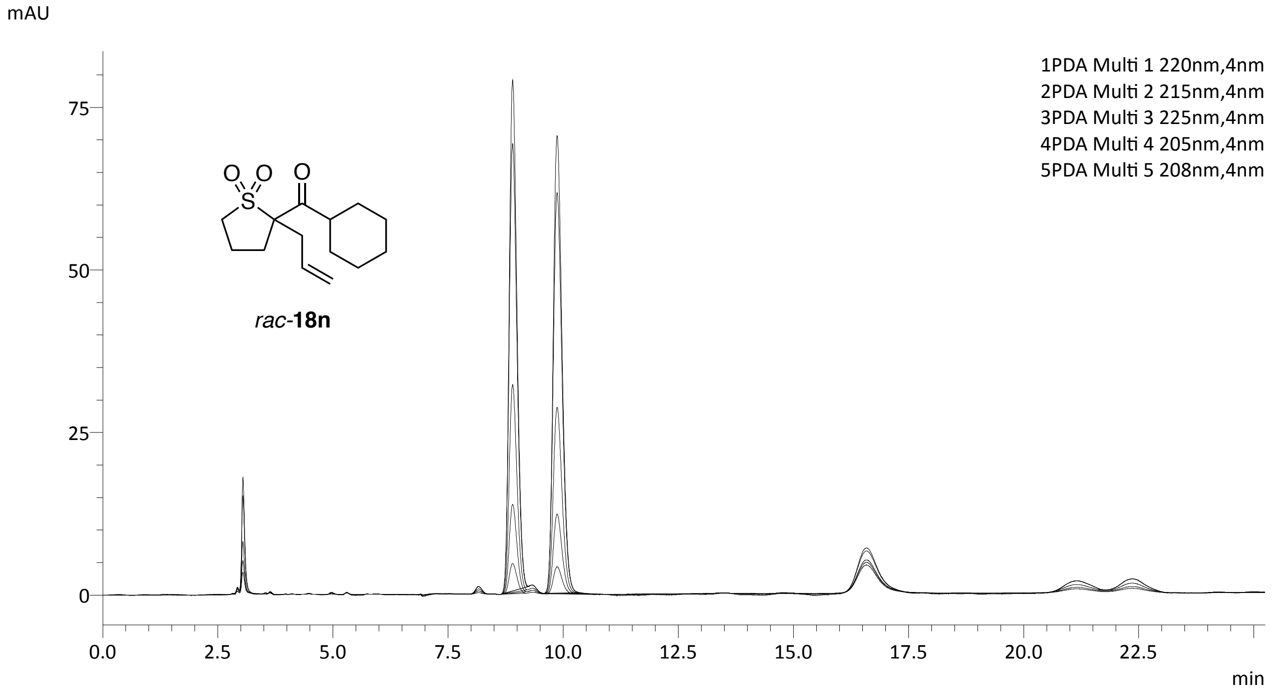

Peak Table

PDA Ch1 220nm

Name	Peak#	Ret. Time	Area	Area%	Mark
	1	8.902	159486	49.536	
	2	9.868	162473	50.464	
	Total		321959	100.000	

PDA Ch2 215nm

Name	Peak#	Ret. Time	Area	Area%	Mark
	1	8.902	374652	49.640	
	2	9.868	380094	50.360	M
	Total		754746	100.000	

PDA Ch3 225nm

Name	Peak#	Ret. Time	Area	Area%	Mark
	1	8.902	54458	49.579	
	2	9.869	55383	50.421	
	Total		109842	100.000	

PDA Ch4 205nm

Name	Peak#	Ret. Time	Area	Area%	Mark
	1	8.902	927812	49.387	
	2	9.868	950829	50.613	S
	Total		1878641	100.000	

PDA Ch5 208nm

Name	Peak#	Ret. Time	Area	Area%	Mark
	1	8.902	810524	49.357	
	2	9.868	831657	50.643	
	Total		1642181	100.000	

mAU

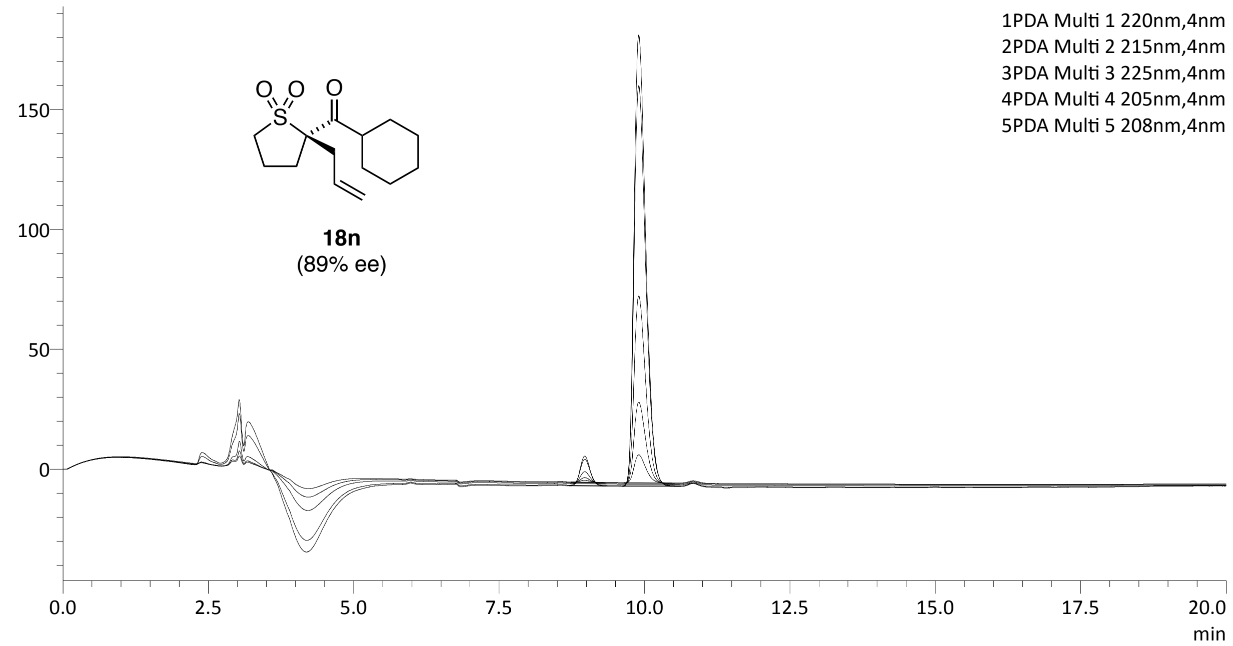

Peak Table

PDA Ch1 220nm

Name	Peak#	Ret. Time	Area	Area%	Mark
	1	8.971	26110	5.231	
	2	9.902	473071	94.769	
	Total		499182	100.000	

PDA Ch2 215nm

Name	Peak#	Ret. Time	Area	Area%	Mark
	1	8.972	60701	5.226	
	2	9.902	1100804	94.774	
	Total		1161505	100.000	

PDA Ch3 225nm

Name	Peak#	Ret. Time	Area	Area%	Mark
	1	8.970	9204	5.366	
	2	9.902	162313	94.634	
	Total		171517	100.000	

PDA Ch4 205nm

Name	Peak#	Ret. Time	Area	Area%	Mark
	1	8.973	149551	5.319	
	2	9.902	2662030	94.681	
	Total		2811581	100.000	

PDA Ch5 208nm

Name	Peak#	Ret. Time	Area	Area%	Mark
	1	8.973	130375	5.260	
	2	9.902	2348282	94.740	
	Total		2478657	100.000	

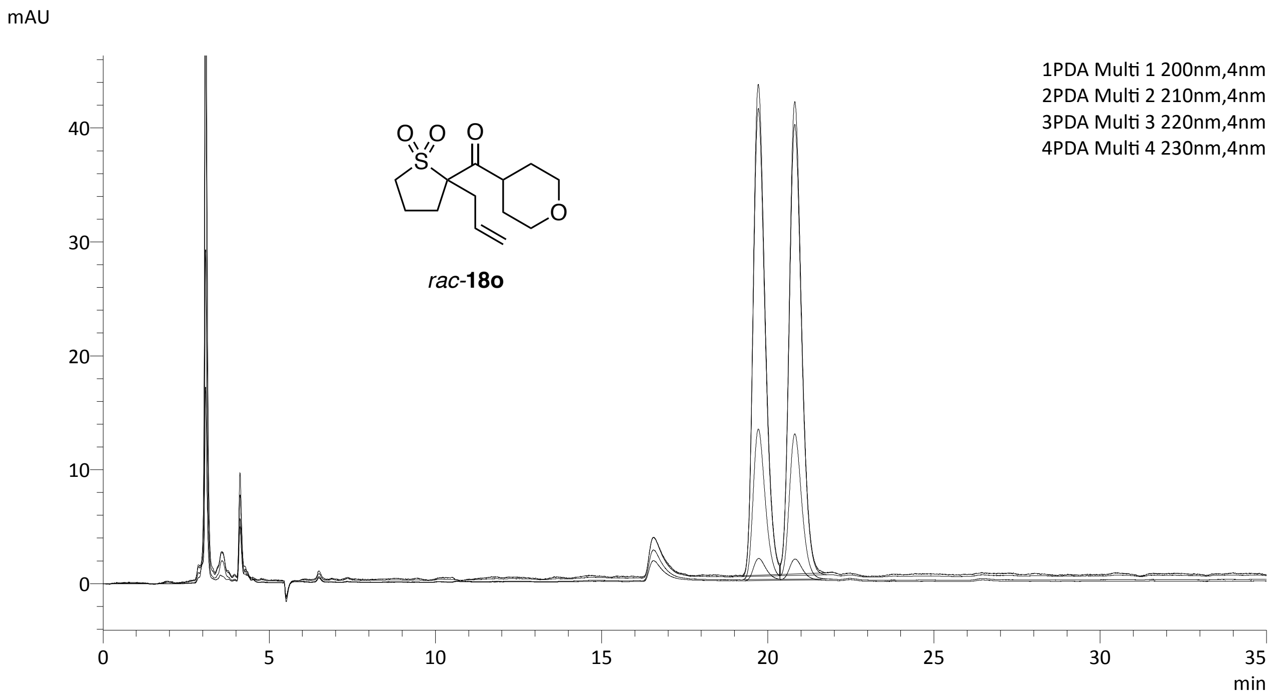

Peak Table

PDA Ch1 200nm

Name	Peak#	Ret. Time	Area	Area%	Mark
	1	19.715	1086773	50.091	
	2	20.813	1082811	49.909	V
	Total		2169584	100.000	

PDA Ch2 210nm

Name	Peak#	Ret. Time	Area	Area%	Mark
	1	19.715	1031829	49.933	
	2	20.813	1034579	50.067	V
	Total		2066408	100.000	

PDA Ch3 220nm

Name	Peak#	Ret. Time	Area	Area%	Mark
	1	19.716	335080	49.851	
	2	20.814	337081	50.149	V
	Total		672160	100.000	

PDA Ch4 230nm

Name	Peak#	Ret. Time	Area	Area%	Mark
	1	19.728	52238	50.595	
	2	20.823	51009	49.405	V
	Total		103247	100.000	

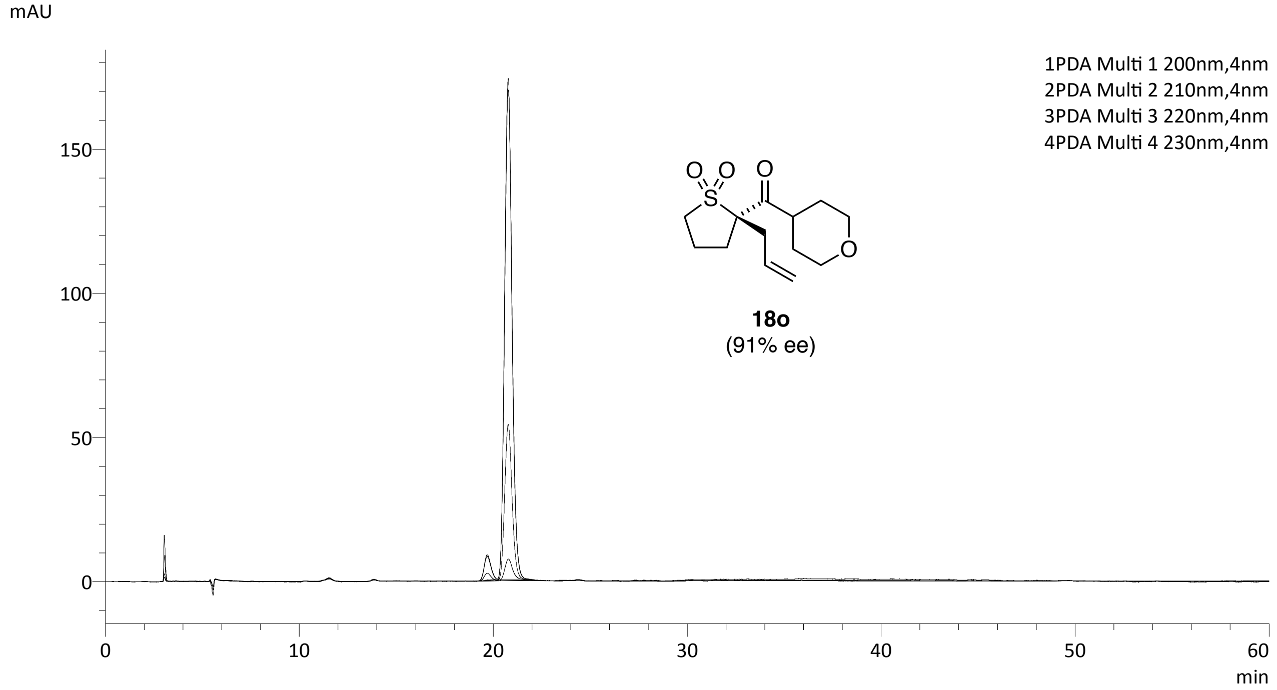

18o
(91% ee)

Peak Table

PDA Ch1 200nm

Name	Peak#	Ret. Time	Area	Area%	Mark
	1	19.689	210618	4.338	M
	2	20.774	4644691	95.662	M
	Total		4855309	100.000	

PDA Ch2 210nm

Name	Peak#	Ret. Time	Area	Area%	Mark
	1	19.693	210981	4.450	
	2	20.774	4530354	95.550	V
	Total		4741335	100.000	

PDA Ch3 220nm

Name	Peak#	Ret. Time	Area	Area%	Mark
	1	19.695	65712	4.306	
	2	20.775	1460452	95.694	V
	Total		1526164	100.000	

PDA Ch4 230nm

Name	Peak#	Ret. Time	Area	Area%	Mark
	1	19.718	8675	3.706	M
	2	20.786	225390	96.294	
	Total		234064	100.000	

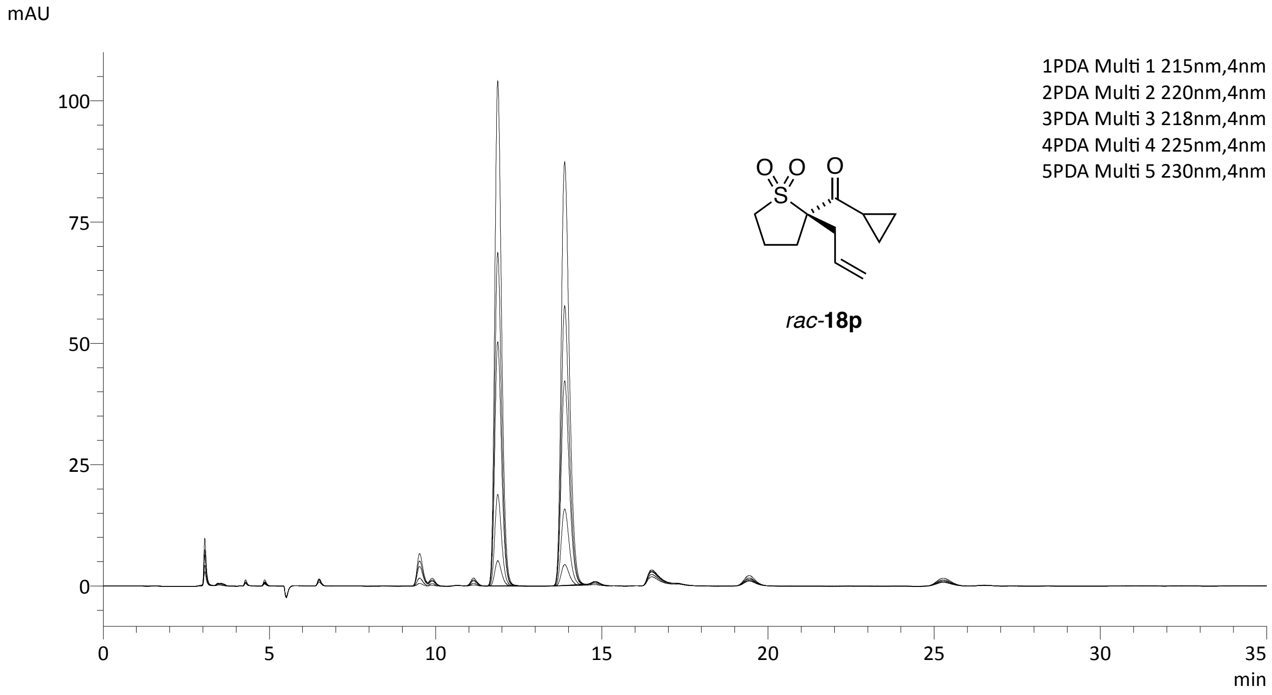

rac-**18p**

Peak Table

PDA Ch1 215nm

Name	Peak#	Ret. Time	Area	Area%	Mark
	1	11.871	1497343	50.042	
	2	13.884	1494856	49.958	
	Total		2992199	100.000	

PDA Ch2 220nm

Name	Peak#	Ret. Time	Area	Area%	Mark
	1	11.871	720910	49.988	
	2	13.884	721270	50.012	
	Total		1442181	100.000	

PDA Ch3 218nm

Name	Peak#	Ret. Time	Area	Area%	Mark
	1	11.871	987365	50.061	
	2	13.884	984961	49.939	
	Total		1972325	100.000	

PDA Ch4 225nm

Name	Peak#	Ret. Time	Area	Area%	Mark
	1	11.871	270033	49.978	
	2	13.884	270272	50.022	
	Total		540305	100.000	

PDA Ch5 230nm

Name	Peak#	Ret. Time	Area	Area%	Mark
	1	11.872	75310	50.091	
	2	13.884	75036	49.909	
	Total		150346	100.000	

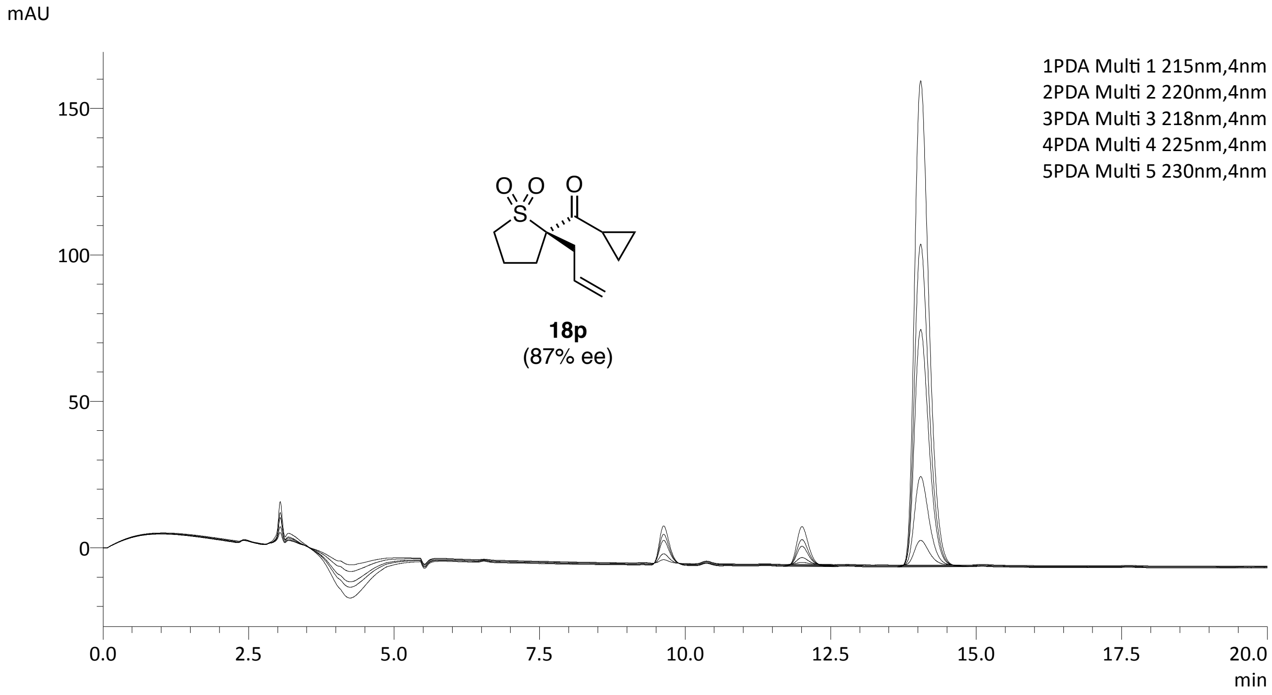

Peak Table

PDA Ch1 215nm

Name	Peak#	Ret. Time	Area	Area%	Mark
	1	12.008	196992	6.345	
	2	14.046	2907813	93.655	
	Total		3104805	100.000	

PDA Ch2 220nm

Name	Peak#	Ret. Time	Area	Area%	Mark
	1	12.008	95405	6.334	
	2	14.046	1410736	93.666	
	Total		1506141	100.000	

PDA Ch3 218nm

Name	Peak#	Ret. Time	Area	Area%	Mark
	1	12.008	129992	6.330	
	2	14.046	1923554	93.670	
	Total		2053546	100.000	

PDA Ch4 225nm

Name	Peak#	Ret. Time	Area	Area%	Mark
	1	12.009	36149	6.384	
	2	14.047	530103	93.616	
	Total		566252	100.000	

PDA Ch5 230nm

Name	Peak#	Ret. Time	Area	Area%	Mark
	1	12.009	10405	6.549	
	2	14.047	148473	93.451	
	Total		158879	100.000	

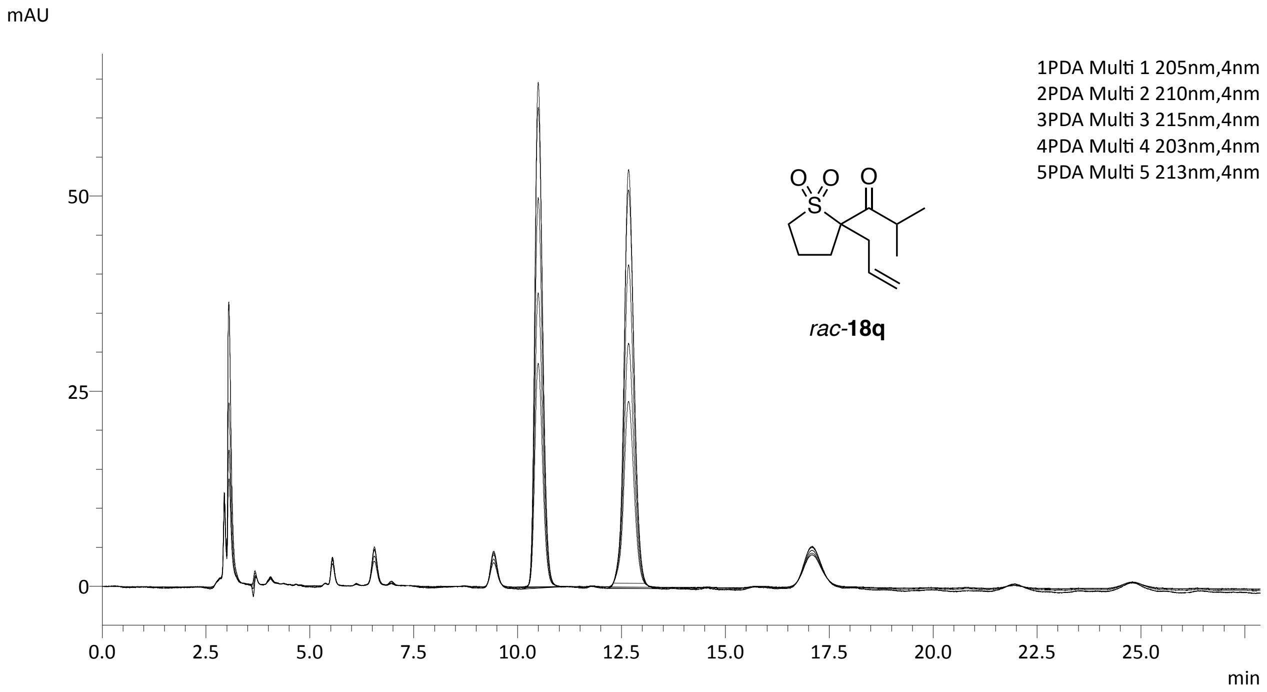

Peak Table

PDA Ch1 205nm

Name	Peak#	Ret. Time	Area	Area%	Mark
	1	10.494	863172	48.302	
	2	12.669	923864	51.698	
	Total		1787035	100.000	

PDA Ch2 210nm

Name	Peak#	Ret. Time	Area	Area%	Mark
	1	10.494	665175	48.274	M
	2	12.669	712730	51.726	M
	Total		1377905	100.000	

PDA Ch3 215nm

Name	Peak#	Ret. Time	Area	Area%	Mark
	1	10.494	381893	49.287	M
	2	12.668	392934	50.713	M
	Total		774827	100.000	

PDA Ch4 203nm

Name	Peak#	Ret. Time	Area	Area%	Mark
	1	10.494	821947	48.335	S
	2	12.670	878559	51.665	
	Total		1700506	100.000	

PDA Ch5 213nm

Name	Peak#	Ret. Time	Area	Area%	Mark
	1	10.494	501154	47.948	
	2	12.668	544054	52.052	
	Total		1045208	100.000	

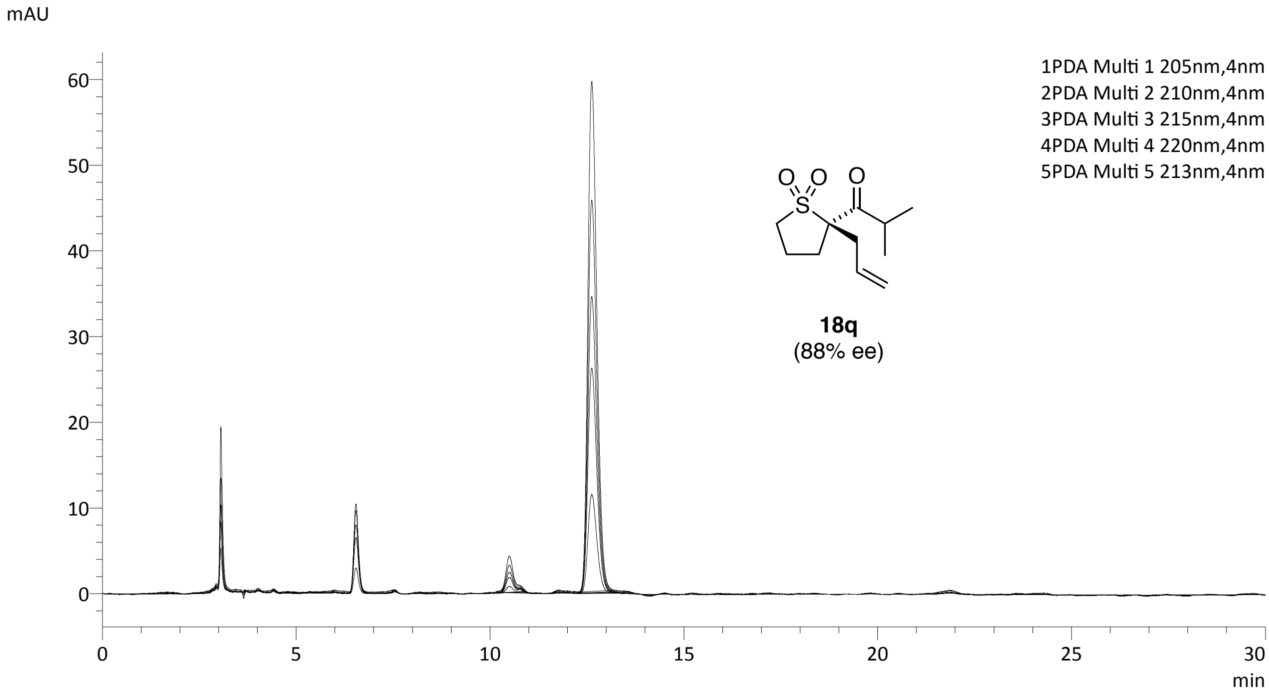

Peak Table

PDA Ch1 205nm

Name	Peak#	Ret. Time	Area	Area%	Mark
	1	10.499	67405	6.523	
	2	12.626	965887	93.477	
	Total		1033292	100.000	

PDA Ch2 210nm

Name	Peak#	Ret. Time	Area	Area%	Mark
	1	10.501	48652	6.142	
	2	12.626	743481	93.858	
	Total		792133	100.000	

PDA Ch3 215nm

Name	Peak#	Ret. Time	Area	Area%	Mark
	1	10.501	24351	5.395	M
	2	12.626	426997	94.605	
	Total		451347	100.000	

PDA Ch4 220nm

Name	Peak#	Ret. Time	Area	Area%	Mark
	1	10.499	10354	5.234	M
	2	12.626	187464	94.766	
	Total		197818	100.000	

PDA Ch5 213nm

Name	Peak#	Ret. Time	Area	Area%	Mark
	1	10.501	33741	5.668	M
	2	12.626	561549	94.332	
	Total		595289	100.000	

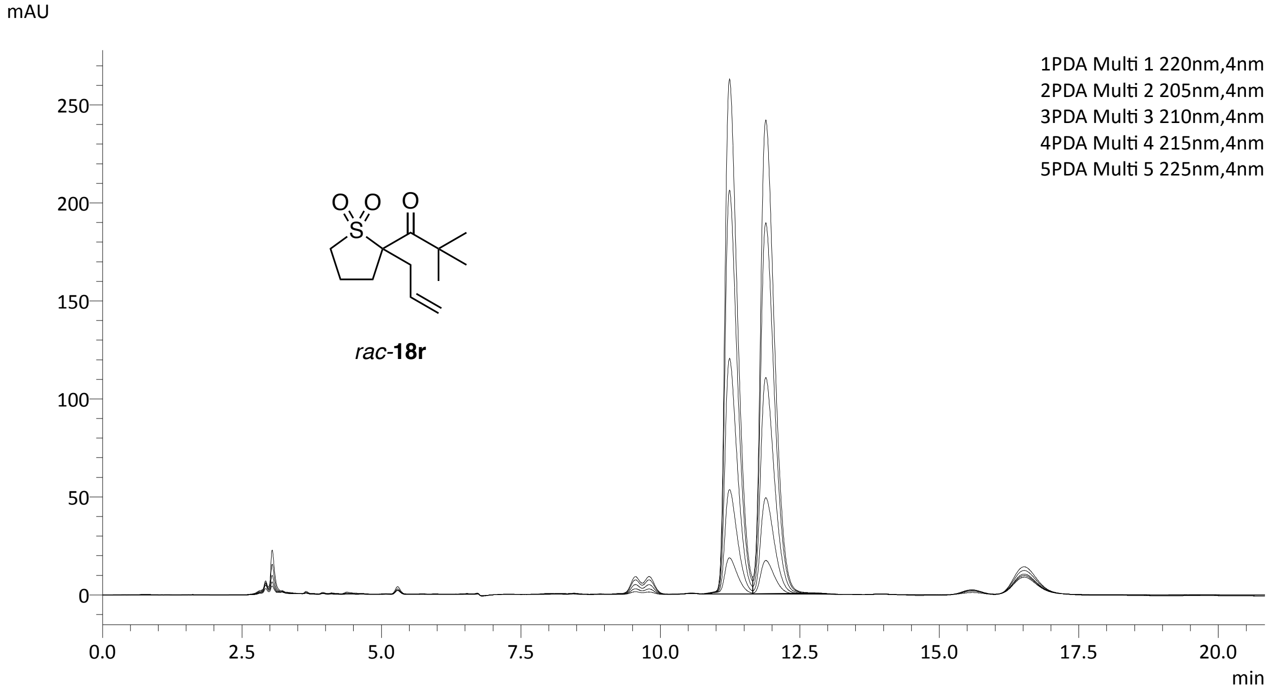

Peak Table

PDA Ch1 220nm

Name	Peak#	Ret. Time	Area	Area%	Mark
	1	11.239	843864	49.955	
	2	11.890	845379	50.045	V
	Total		1689244	100.000	

PDA Ch2 205nm

Name	Peak#	Ret. Time	Area	Area%	Mark
	1	11.240	4186829	49.459	
	2	11.890	4278491	50.541	V
	Total		8465319	100.000	

PDA Ch3 210nm

Name	Peak#	Ret. Time	Area	Area%	Mark
	1	11.240	3260323	49.488	
	2	11.890	3327766	50.512	SV
	Total		6588089	100.000	

PDA Ch4 215nm

Name	Peak#	Ret. Time	Area	Area%	Mark
	1	11.240	1896046	49.776	
	2	11.890	1913118	50.224	V
	Total		3809163	100.000	

PDA Ch5 225nm

Name	Peak#	Ret. Time	Area	Area%	Mark
	1	11.239	297529	50.406	
	2	11.891	292734	49.594	V
	Total		590263	100.000	

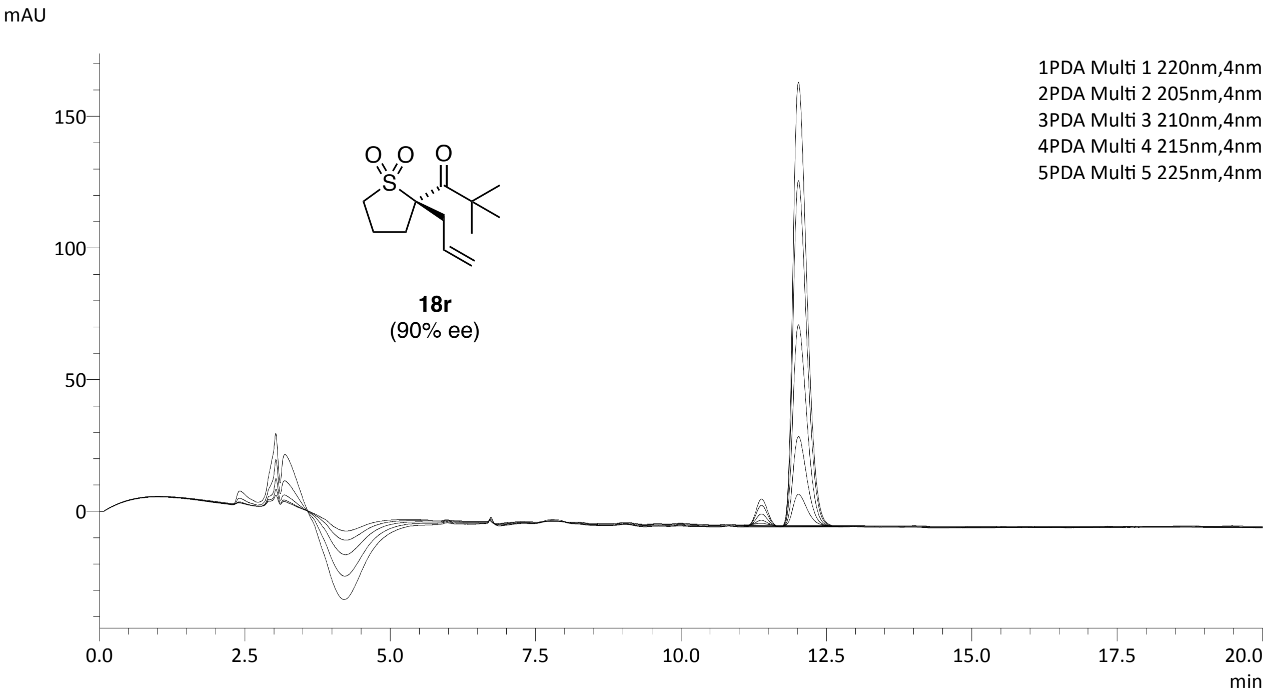

Peak Table

PDA Ch1 220nm

Name	Peak#	Ret. Time	Area	Area%	Mark
	1	11.380	31264	5.191	
	2	12.020	570979	94.809	
	Total		602242	100.000	

PDA Ch2 205nm

Name	Peak#	Ret. Time	Area	Area%	Mark
	1	11.384	151062	5.037	
	2	12.020	2847688	94.963	
	Total		2998750	100.000	

PDA Ch3 210nm

Name	Peak#	Ret. Time	Area	Area%	Mark
	1	11.384	117205	5.039	
	2	12.020	2208541	94.961	
	Total		2325746	100.000	

PDA Ch4 215nm

Name	Peak#	Ret. Time	Area	Area%	Mark
	1	11.383	69089	5.098	
	2	12.020	1286209	94.902	
	Total		1355298	100.000	

PDA Ch5 225nm

Name	Peak#	Ret. Time	Area	Area%	Mark
	1	11.371	11130	5.244	M
	2	12.021	201110	94.756	
	Total		212240	100.000	

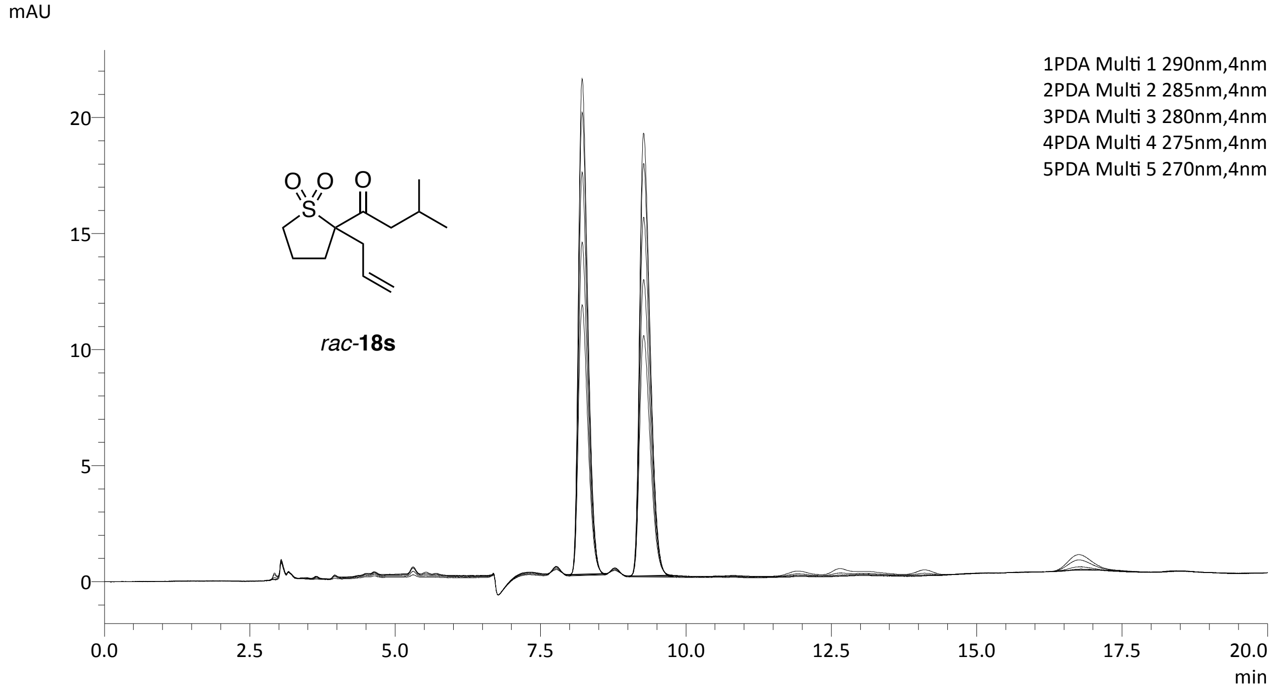

Peak Table

PDA Ch1 290nm

Name	Peak#	Ret. Time	Area	Area%	Mark
	1	8.215	242720	49.671	
	2	9.273	245940	50.329	
	Total		488660	100.000	

PDA Ch2 285nm

Name	Peak#	Ret. Time	Area	Area%	Mark
	1	8.215	226292	49.696	
	2	9.273	229063	50.304	
	Total		455355	100.000	

PDA Ch3 280nm

Name	Peak#	Ret. Time	Area	Area%	Mark
	1	8.216	197077	49.720	
	2	9.273	199300	50.280	
	Total		396376	100.000	

PDA Ch4 275nm

Name	Peak#	Ret. Time	Area	Area%	Mark
	1	8.216	163157	47.941	
	2	9.273	164894	48.451	
	3	16.746	12281	3.608	
	Total		340332	100.000	

PDA Ch5 270nm

Name	Peak#	Ret. Time	Area	Area%	Mark
	1	8.216	132512	49.723	
	2	9.274	133991	50.277	
	Total		266503	100.000	

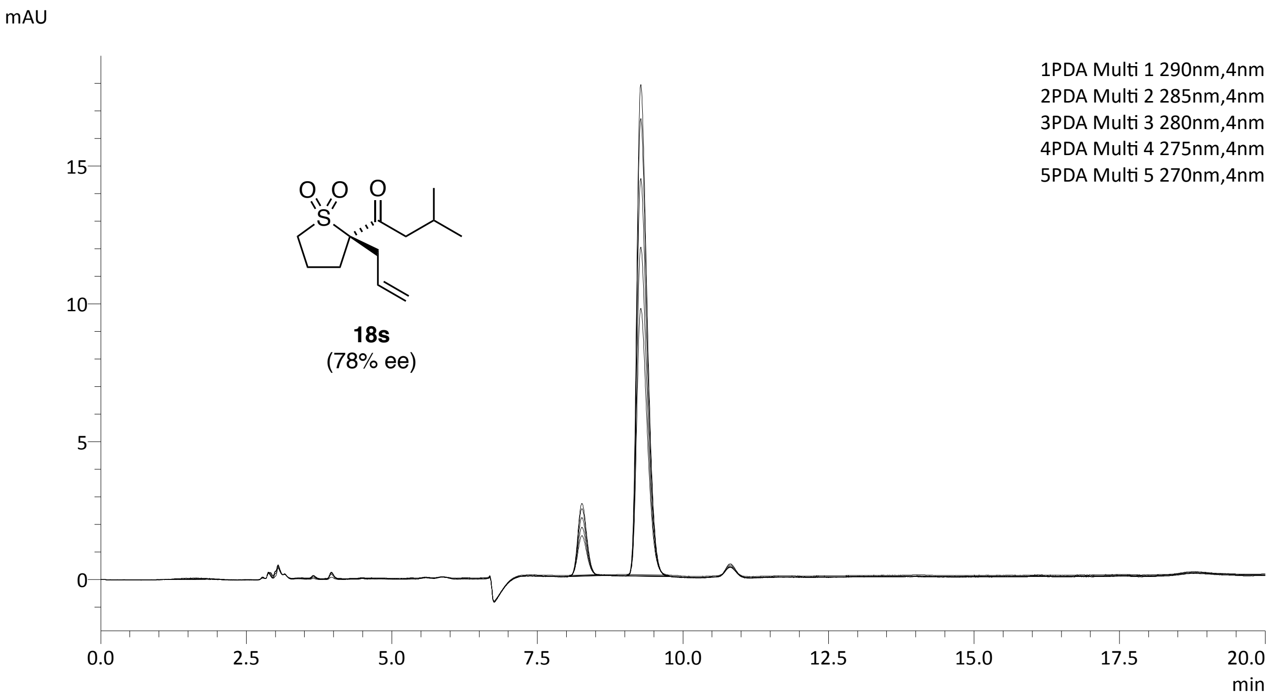

Peak Table

PDA Ch1 290nm

Name	Peak#	Ret. Time	Area	Area%	Mark
	1	8.266	28444	11.202	
	2	9.277	225465	88.798	
	Total		253909	100.000	

PDA Ch2 285nm

Name	Peak#	Ret. Time	Area	Area%	Mark
	1	8.265	26442	11.177	
	2	9.277	210129	88.823	
	Total		236571	100.000	

PDA Ch3 280nm

Name	Peak#	Ret. Time	Area	Area%	Mark
	1	8.265	23052	11.193	
	2	9.277	182893	88.807	
	Total		205945	100.000	

PDA Ch4 275nm

Name	Peak#	Ret. Time	Area	Area%	Mark
	1	8.265	19041	11.179	
	2	9.277	151292	88.821	
	Total		170333	100.000	

PDA Ch5 270nm

Name	Peak#	Ret. Time	Area	Area%	Mark
	1	8.265	15426	11.162	
	2	9.278	122779	88.838	
	Total		138205	100.000	

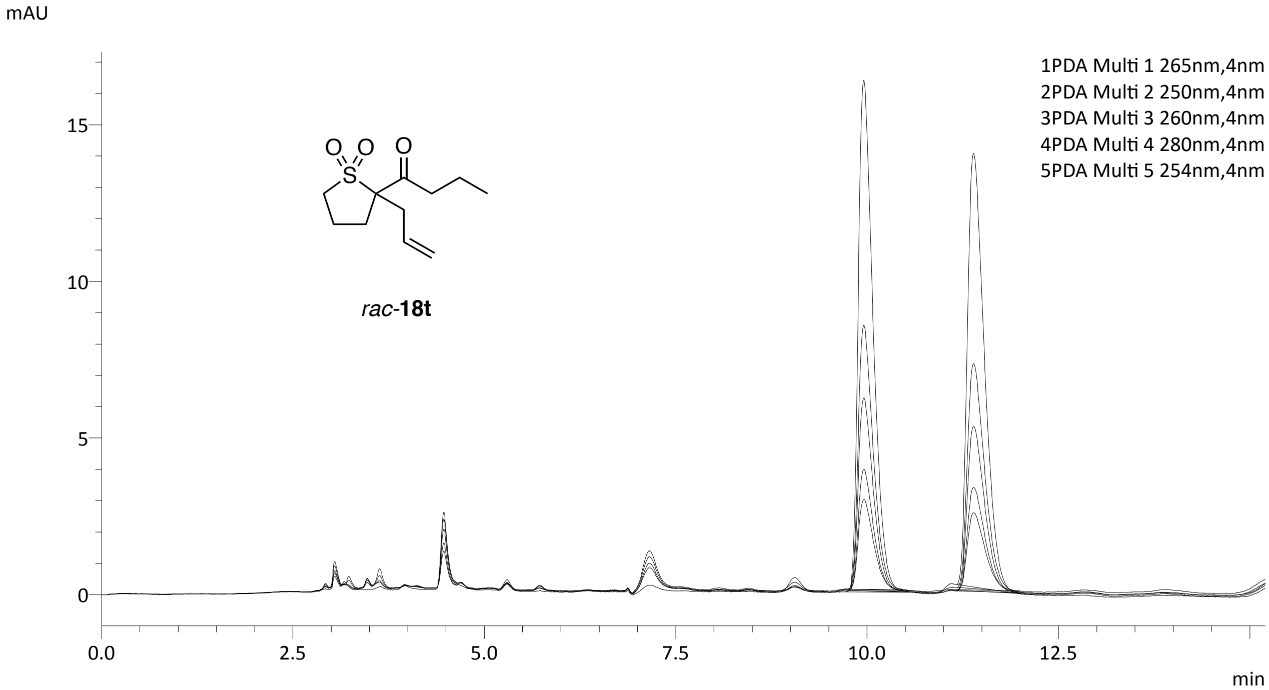

Peak Table

PDA Ch1 265nm

Name	Peak#	Ret. Time	Area	Area%	Mark
	1	9.957	115324	50.571	
	2	11.393	112719	49.429	
	Total		228042	100.000	

PDA Ch2 250nm

Name	Peak#	Ret. Time	Area	Area%	Mark
	1	9.959	39487	49.804	
	2	11.393	39799	50.196	
	Total		79286	100.000	

PDA Ch3 260nm

Name	Peak#	Ret. Time	Area	Area%	Mark
	1	9.958	84446	50.341	
	2	11.393	83301	49.659	
	Total		167747	100.000	

PDA Ch4 280nm

Name	Peak#	Ret. Time	Area	Area%	Mark
	1	9.956	221554	50.759	
	2	11.392	214930	49.241	
	Total		436484	100.000	

PDA Ch5 254nm

Name	Peak#	Ret. Time	Area	Area%	Mark
	1	9.959	53652	50.278	
	2	11.393	53058	49.722	
	Total		106710	100.000	

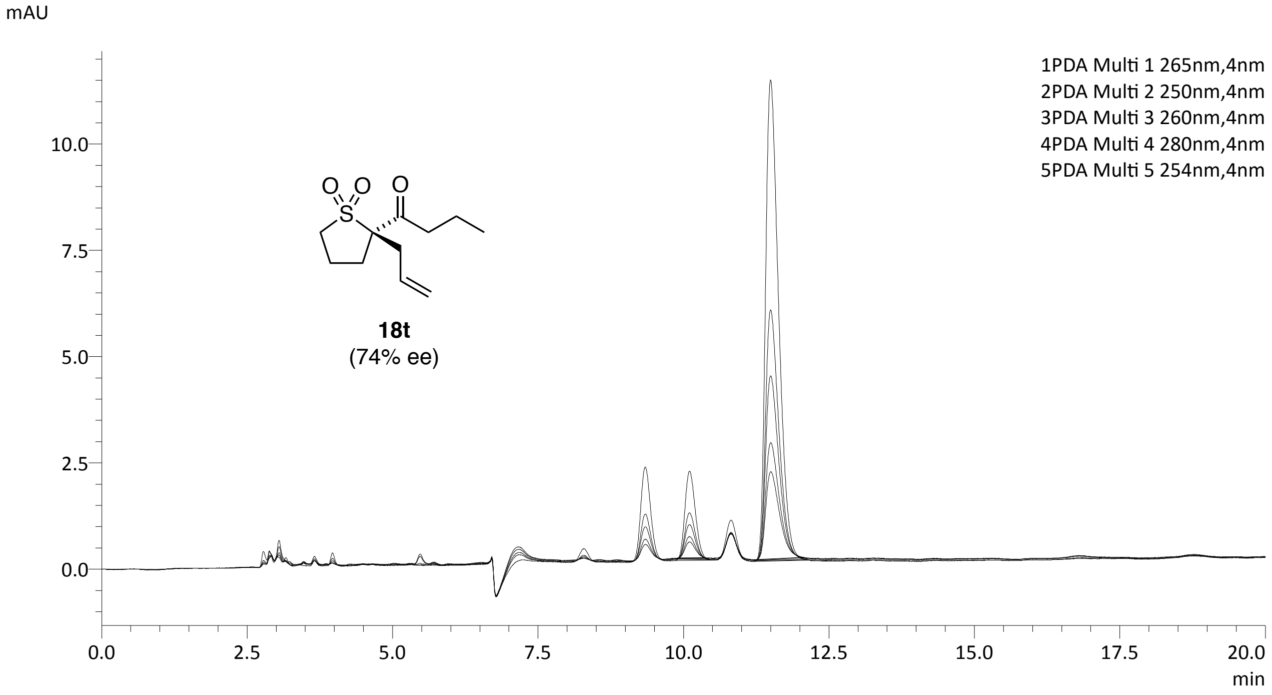

Peak Table

PDA Ch1 265nm

Name	Peak#	Ret. Time	Area	Area%	Mark
	1	10.103	13923	12.925	
	2	11.498	93797	87.075	
	Total		107719	100.000	

PDA Ch2 250nm

Name	Peak#	Ret. Time	Area	Area%	Mark
	1	10.103	4566	11.911	
	2	11.501	33772	88.089	
	Total		38339	100.000	

PDA Ch3 260nm

Name	Peak#	Ret. Time	Area	Area%	Mark
	1	10.103	10077	12.655	
	2	11.499	69553	87.345	
	Total		79630	100.000	

PDA Ch4 280nm

Name	Peak#	Ret. Time	Area	Area%	Mark
	1	10.104	26636	13.047	
	2	11.497	177523	86.953	
	Total		204158	100.000	

PDA Ch5 254nm

Name	Peak#	Ret. Time	Area	Area%	Mark
	1	10.102	6309	12.403	
	2	11.501	44554	87.597	
	Total		50863	100.000	

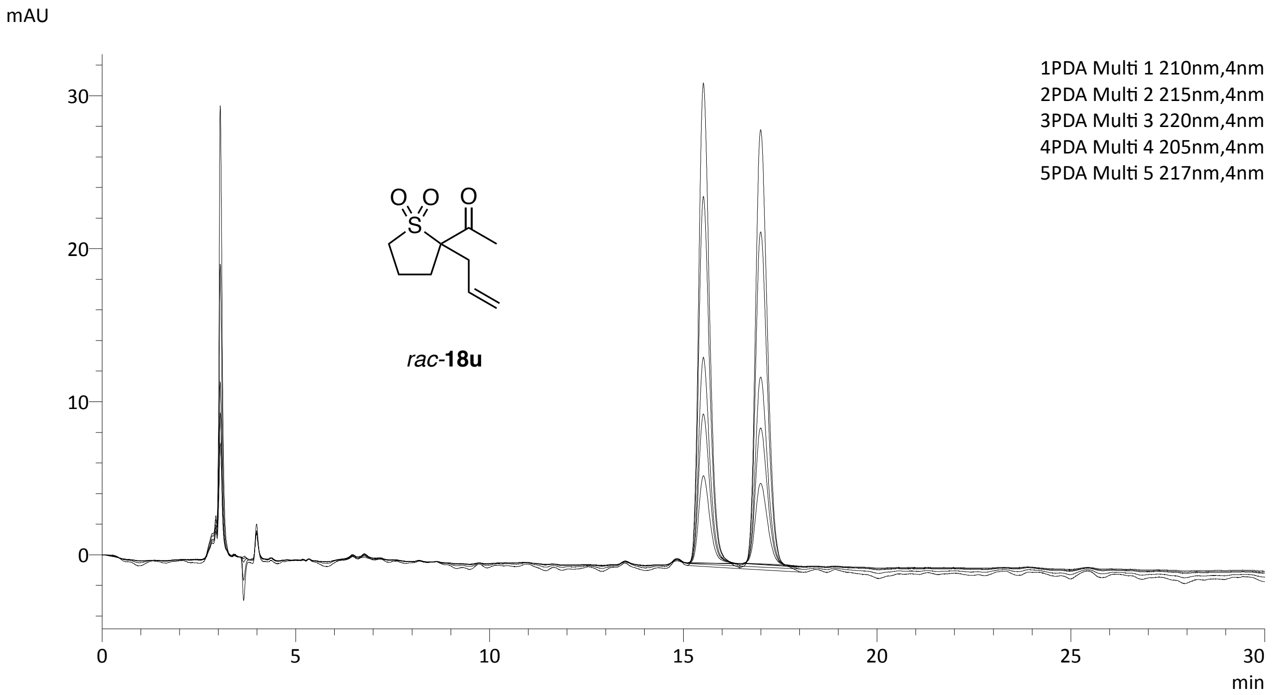

Peak Table

PDA Ch1 210nm

Name	Peak#	Ret. Time	Area	Area%	Mark
	1	15.519	478971	49.914	
	2	16.997	480618	50.086	V
	Total		959588	100.000	

PDA Ch2 215nm

Name	Peak#	Ret. Time	Area	Area%	Mark
	1	15.519	267218	49.993	
	2	16.997	267292	50.007	
	Total		534510	100.000	

PDA Ch3 220nm

Name	Peak#	Ret. Time	Area	Area%	Mark
	1	15.520	114637	50.086	
	2	16.996	114243	49.914	
	Total		228880	100.000	

PDA Ch4 205nm

Name	Peak#	Ret. Time	Area	Area%	Mark
	1	15.519	629938	49.854	
	2	16.996	633636	50.146	V
	Total		1263574	100.000	

PDA Ch5 217nm

Name	Peak#	Ret. Time	Area	Area%	Mark
	1	15.519	193969	49.935	
	2	16.997	194473	50.065	
	Total		388442	100.000	

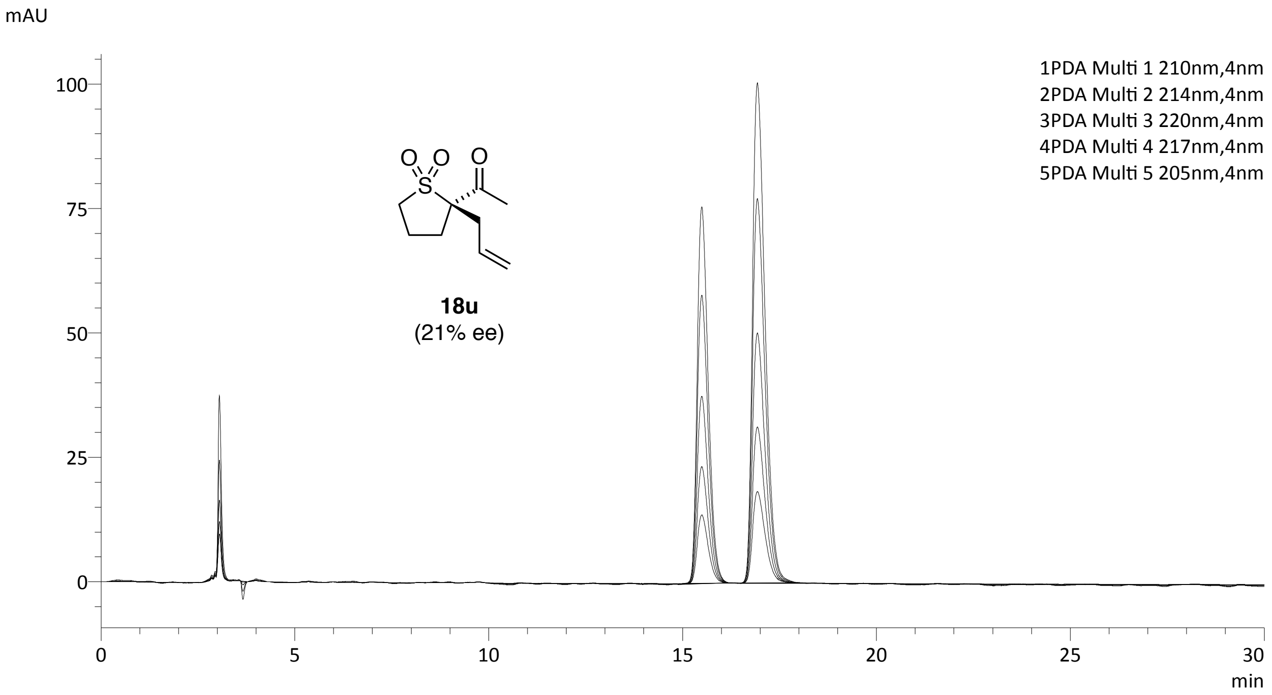

Peak Table

PDA Ch1 210nm

Name	Peak#	Ret. Time	Area	Area%	Mark
	1	15.495	1146211	39.595	
	2	16.929	1748620	60.405	
	Total		2894831	100.000	

PDA Ch2 214nm

Name	Peak#	Ret. Time	Area	Area%	Mark
	1	15.495	744319	39.637	
	2	16.930	1133519	60.363	
	Total		1877838	100.000	

PDA Ch3 220nm

Name	Peak#	Ret. Time	Area	Area%	Mark
	1	15.495	272001	39.598	
	2	16.931	414909	60.402	
	Total		686909	100.000	

PDA Ch4 217nm

Name	Peak#	Ret. Time	Area	Area%	Mark
	1	15.495	464432	39.617	
	2	16.930	707863	60.383	
	Total		1172294	100.000	

PDA Ch5 205nm

Name	Peak#	Ret. Time	Area	Area%	Mark
	1	15.495	1505581	39.736	
	2	16.929	2283353	60.264	
	Total		3788934	100.000	

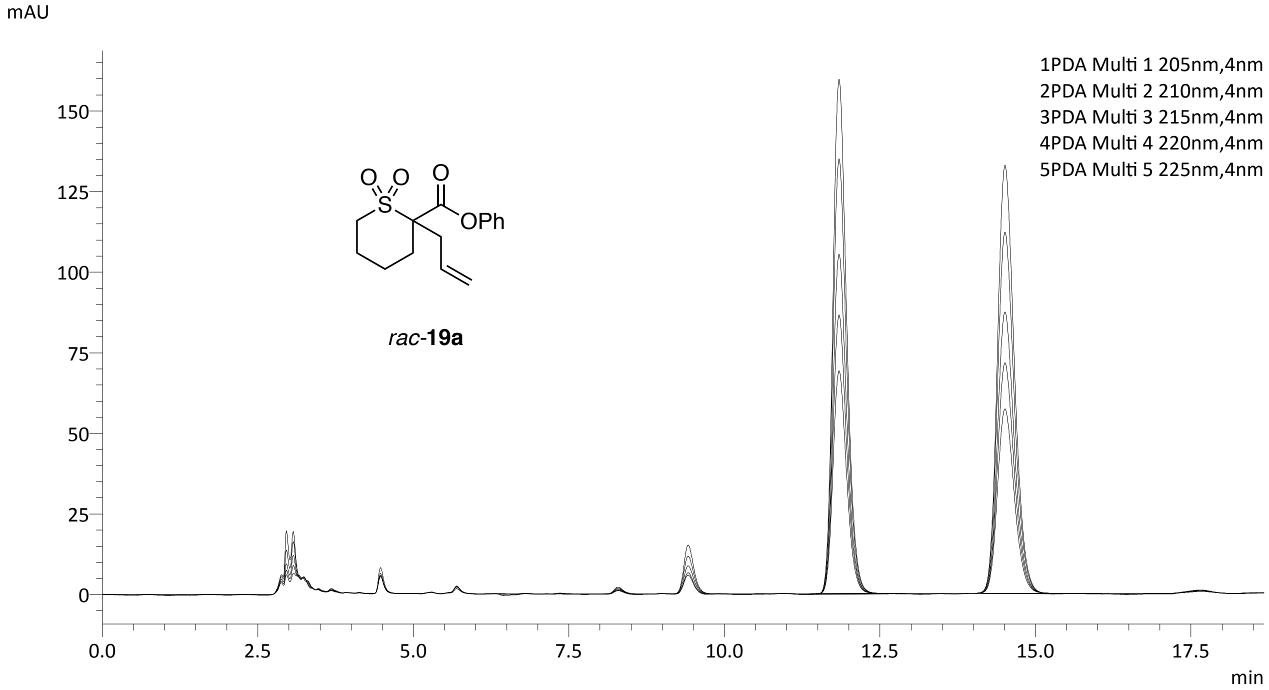

Peak Table

PDA Ch1 205nm

Name	Peak#	Ret. Time	Area	Area%	Mark
	1	11.842	2475257	49.410	
	2	14.510	2534347	50.590	
	Total		5009603	100.000	

PDA Ch2 210nm

Name	Peak#	Ret. Time	Area	Area%	Mark
	1	11.842	2078273	49.382	
	2	14.510	2130290	50.618	
	Total		4208563	100.000	

PDA Ch3 215nm

Name	Peak#	Ret. Time	Area	Area%	Mark
	1	11.842	1617316	49.413	
	2	14.510	1655722	50.587	
	Total		3273038	100.000	

PDA Ch4 220nm

Name	Peak#	Ret. Time	Area	Area%	Mark
	1	11.842	1325361	49.484	
	2	14.510	1352997	50.516	
	Total		2678357	100.000	

PDA Ch5 225nm

Name	Peak#	Ret. Time	Area	Area%	Mark
	1	11.842	1059799	49.455	
	2	14.510	1083153	50.545	
	Total		2142952	100.000	

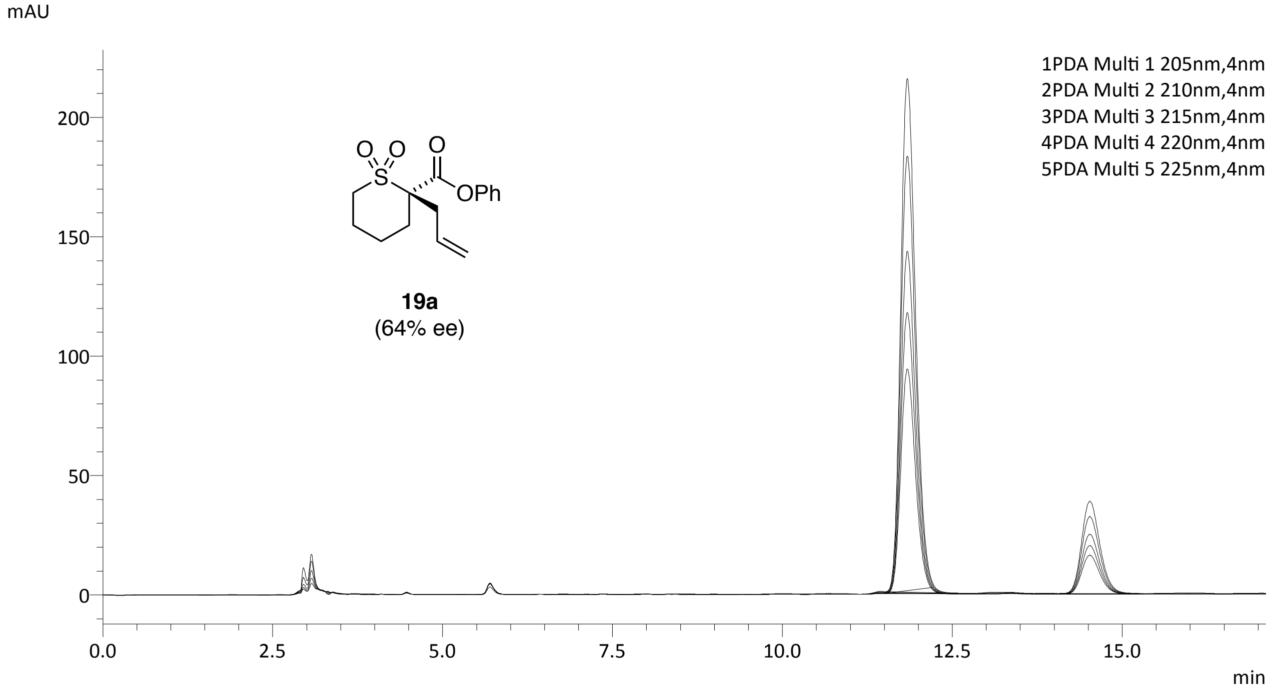

Peak Table

PDA Ch1 205nm

Name	Peak#	Ret. Time	Area	Area%	Mark
	1	11.838	3327288	82.030	
	2	14.524	728896	17.970	
	Total		4056183	100.000	

PDA Ch2 210nm

Name	Peak#	Ret. Time	Area	Area%	Mark
	1	11.838	2834427	82.332	M
	2	14.524	608273	17.668	
	Total		3442700	100.000	

PDA Ch3 215nm

Name	Peak#	Ret. Time	Area	Area%	Mark
	1	11.838	2192733	82.375	
	2	14.523	469155	17.625	
	Total		2661888	100.000	

PDA Ch4 220nm

Name	Peak#	Ret. Time	Area	Area%	Mark
	1	11.838	1737259	82.033	M
	2	14.523	380507	17.967	M
	Total		2117766	100.000	

PDA Ch5 225nm

Name	Peak#	Ret. Time	Area	Area%	Mark
	1	11.838	1438541	82.470	
	2	14.524	305769	17.530	
	Total		1744311	100.000	

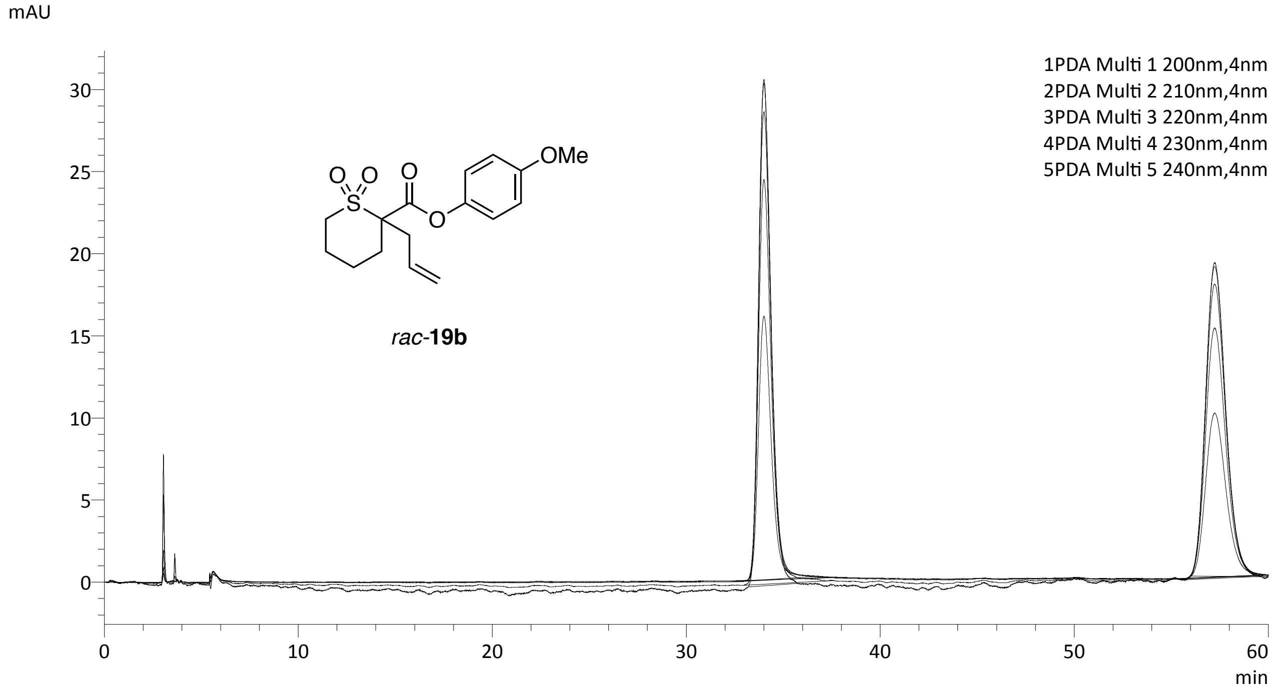

Peak Table

PDA Ch1 200nm

Name	Peak#	Ret. Time	Area	Area%	Mark
	1	34.009	1390813	50.090	S
	2	57.230	1385836	49.910	S
	Total		2776649	100.000	

PDA Ch2 210nm

Name	Peak#	Ret. Time	Area	Area%	Mark
	1	34.007	1346071	50.413	M
	2	57.241	1324030	49.587	M
	Total		2670101	100.000	

PDA Ch3 220nm

Name	Peak#	Ret. Time	Area	Area%	Mark
	1	34.006	1400563	50.063	M
	2	57.238	1397030	49.937	M
	Total		2797593	100.000	

PDA Ch4 230nm

Name	Peak#	Ret. Time	Area	Area%	Mark
	1	34.006	1134916	50.361	M
	2	57.239	1118644	49.639	M
	Total		2253560	100.000	

PDA Ch5 240nm

Name	Peak#	Ret. Time	Area	Area%	Mark
	1	34.009	764469	50.748	M
	2	57.241	741941	49.252	M
	Total		1506410	100.000	

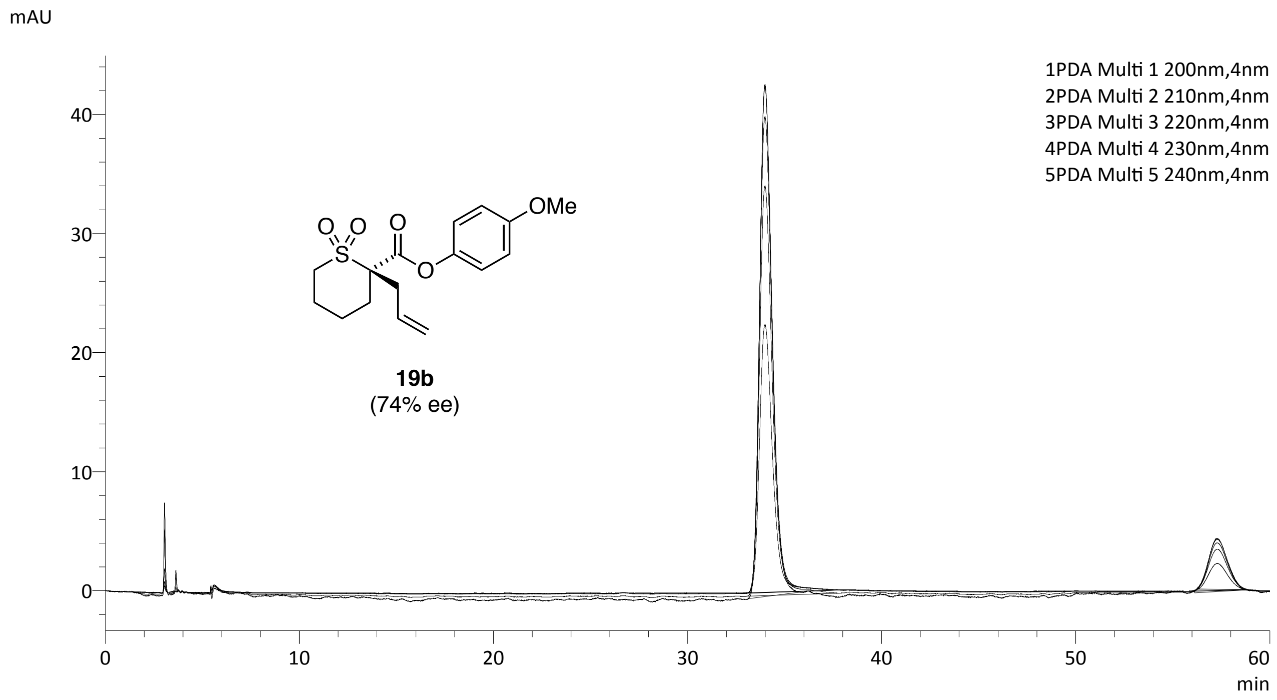

Peak Table

PDA Ch1 200nm

Name	Peak#	Ret. Time	Area	Area%	Mark
	1	33.993	1957903	86.043	S
	2	57.297	317589	13.957	SV
	Total		2275491	100.000	

PDA Ch2 210nm

Name	Peak#	Ret. Time	Area	Area%	Mark
	1	33.992	1895790	87.137	M
	2	57.313	279851	12.863	
	Total		2175641	100.000	

PDA Ch3 220nm

Name	Peak#	Ret. Time	Area	Area%	Mark
	1	33.992	1990117	86.999	M
	2	57.303	297394	13.001	
	Total		2287511	100.000	

PDA Ch4 230nm

Name	Peak#	Ret. Time	Area	Area%	Mark
	1	33.992	1600605	87.308	M
	2	57.305	232684	12.692	
	Total		1833289	100.000	

PDA Ch5 240nm

Name	Peak#	Ret. Time	Area	Area%	Mark
	1	33.992	1072398	88.044	M
	2	57.299	145626	11.956	
	Total		1218024	100.000	

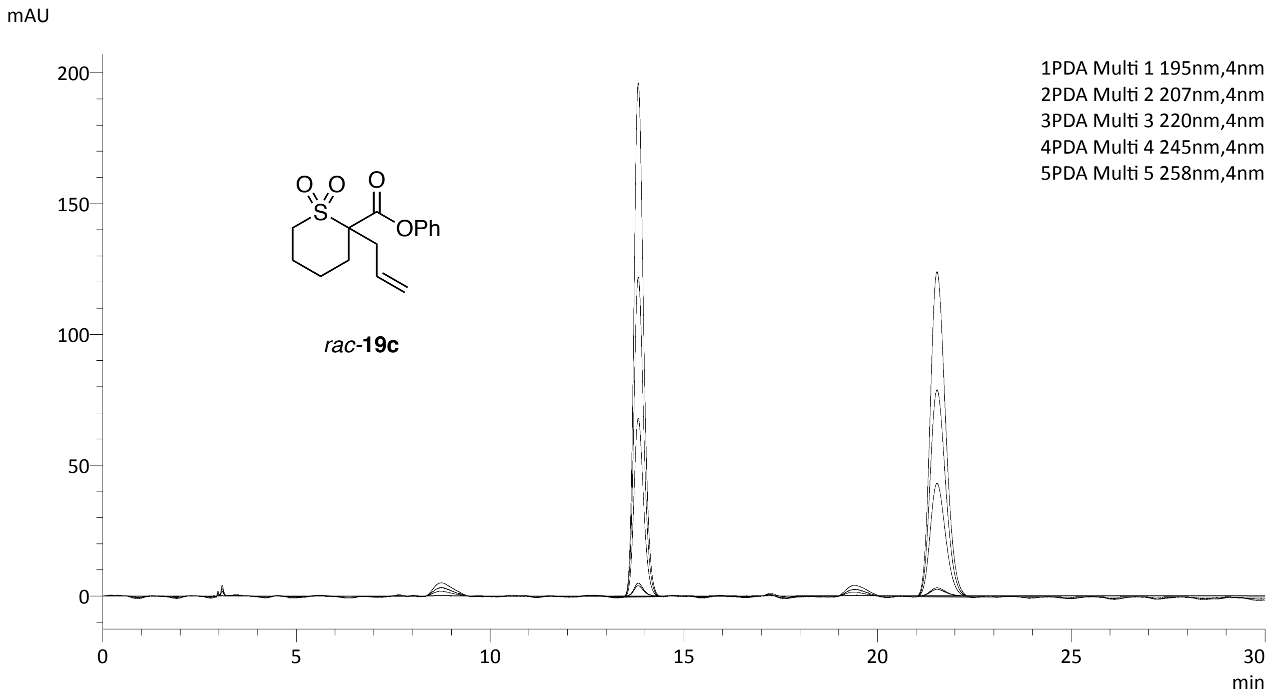

Peak Table

PDA Ch1 195nm

Name	Peak#	Ret. Time	Area	Area%	Mark
	1	13.825	2176630	49.570	M
Compound 1	2	21.537	2214392	50.430	M
	Total		4391023	100.000	

PDA Ch2 207nm

Name	Peak#	Ret. Time	Area	Area%	Mark
	1	13.825	3435900	49.861	
Compound 1	2	21.537	3455081	50.139	
	Total		6890981	100.000	

PDA Ch3 220nm

Name	Peak#	Ret. Time	Area	Area%	Mark
	1	13.825	1189684	49.762	
Compound 1	2	21.536	1201055	50.238	
	Total		2390739	100.000	

PDA Ch4 245nm

Name	Peak#	Ret. Time	Area	Area%	Mark
	1	13.825	68657	50.646	M
Compound 1	2	21.535	66905	49.354	
	Total		135562	100.000	

PDA Ch5 258nm

Name	Peak#	Ret. Time	Area	Area%	Mark
	1	13.825	84532	50.301	
Compound 1	2	21.534	83522	49.699	
	Total		168054	100.000	

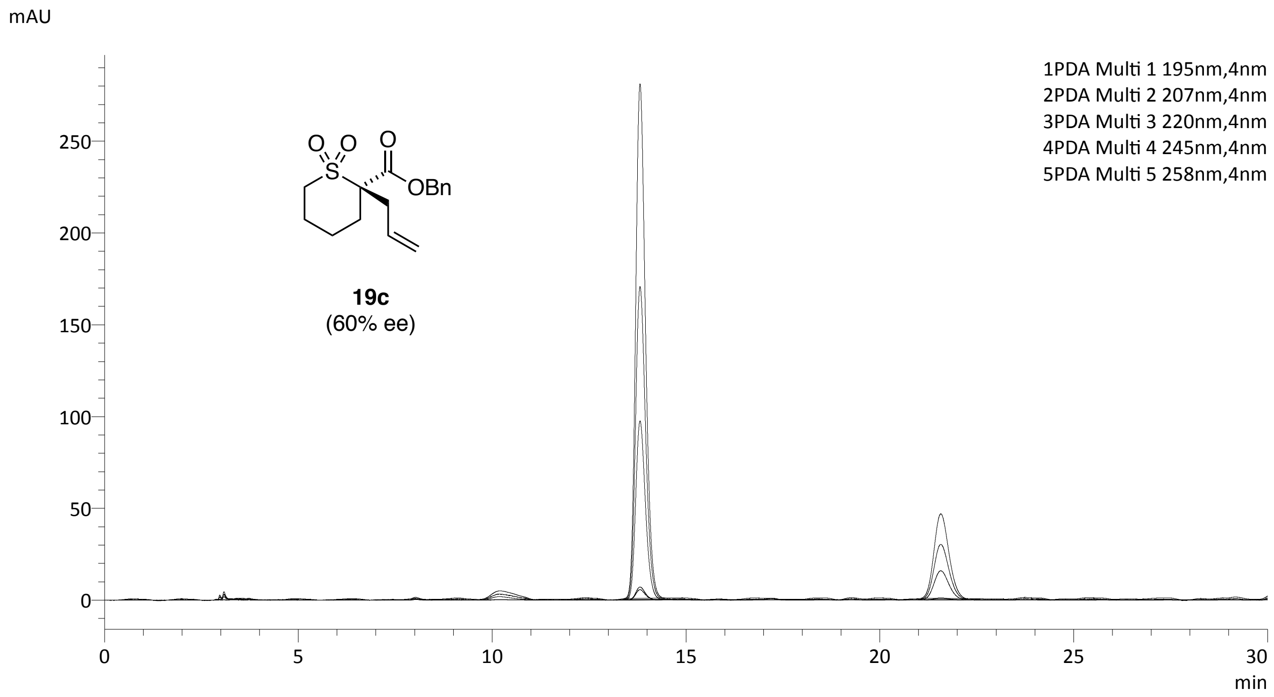

Peak Table

PDA Ch1 195nm

Name	Peak#	Ret. Time	Area	Area%	Mark
	1	13.815	3084017	79.227	M
Compound 1	2	21.565	808601	20.773	M
	Total		3892618	100.000	

PDA Ch2 207nm

Name	Peak#	Ret. Time	Area	Area%	Mark
	1	13.815	4930150	80.010	
Compound 1	2	21.573	1231802	19.990	
	Total		6161952	100.000	

PDA Ch3 220nm

Name	Peak#	Ret. Time	Area	Area%	Mark
	1	13.815	1715192	80.056	
Compound 1	2	21.573	427310	19.944	
	Total		2142502	100.000	

PDA Ch4 245nm

Name	Peak#	Ret. Time	Area	Area%	Mark
	1	13.816	97183	80.972	
Compound 1	2	21.567	22838	19.028	
	Total		120021	100.000	

PDA Ch5 258nm

Name	Peak#	Ret. Time	Area	Area%	Mark
	1	13.815	122308	80.692	
Compound 1	2	21.570	29266	19.308	
	Total		151574	100.000	

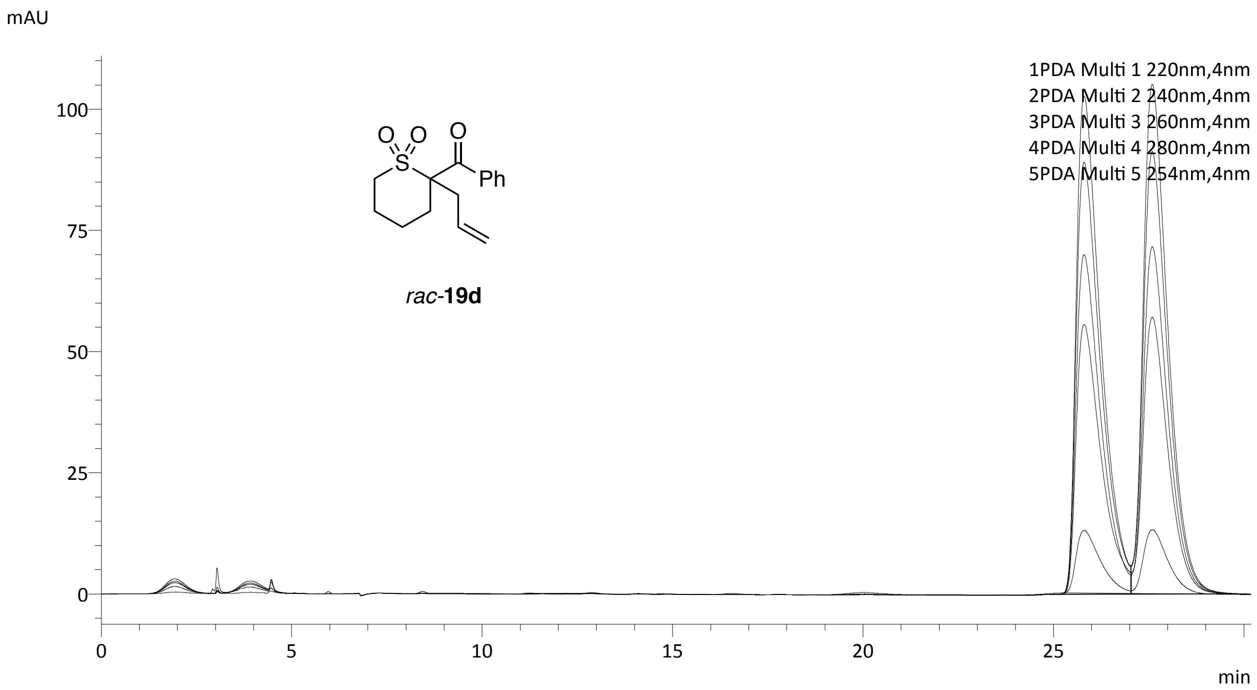

Peak Table

PDA Ch1 220nm

Name	Peak#	Ret. Time	Area	Area%	Mark
	1	25.805	2555666	49.440	
	2	27.593	2613537	50.560	V
	Total		5169203	100.000	

PDA Ch2 240nm

Name	Peak#	Ret. Time	Area	Area%	Mark
	1	25.804	4043636	49.310	
	2	27.593	4156812	50.690	V
	Total		8200449	100.000	

PDA Ch3 260nm

Name	Peak#	Ret. Time	Area	Area%	Mark
	1	25.804	3161519	49.291	
	2	27.594	3252443	50.709	V
	Total		6413962	100.000	

PDA Ch4 280nm

Name	Peak#	Ret. Time	Area	Area%	Mark
	1	25.801	572532	49.359	
	2	27.594	587402	50.641	V
	Total		1159935	100.000	

PDA Ch5 254nm

Name	Peak#	Ret. Time	Area	Area%	Mark
	1	25.805	4640437	49.229	
	2	27.594	4785872	50.771	V
	Total		9426309	100.000	

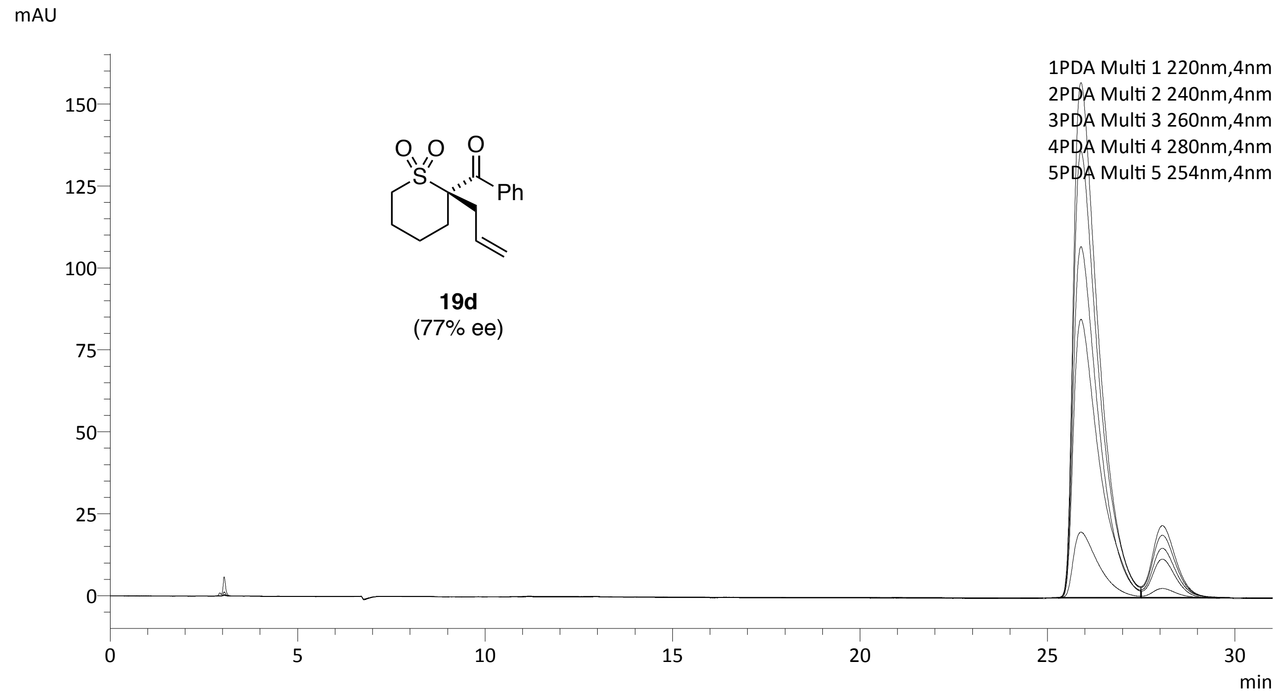

Peak Table

PDA Ch1 220nm

Name	Peak#	Ret. Time	Area	Area%	Mark
	1	25.893	4122129	88.555	
	2	28.065	532747	11.445	V
	Total		4654876	100.000	

PDA Ch2 240nm

Name	Peak#	Ret. Time	Area	Area%	Mark
	1	25.893	6440491	88.397	
	2	28.064	845383	11.603	V
	Total		7285874	100.000	

PDA Ch3 260nm

Name	Peak#	Ret. Time	Area	Area%	Mark
	1	25.893	5022548	88.288	
	2	28.065	666303	11.712	V
	Total		5688851	100.000	

PDA Ch4 280nm

Name	Peak#	Ret. Time	Area	Area%	Mark
	1	25.893	938161	88.651	
	2	28.060	120106	11.349	V
	Total		1058267	100.000	

PDA Ch5 254nm

Name	Peak#	Ret. Time	Area	Area%	Mark
	1	25.893	7365878	88.284	
	2	28.066	977467	11.716	V
	Total		8343345	100.000	

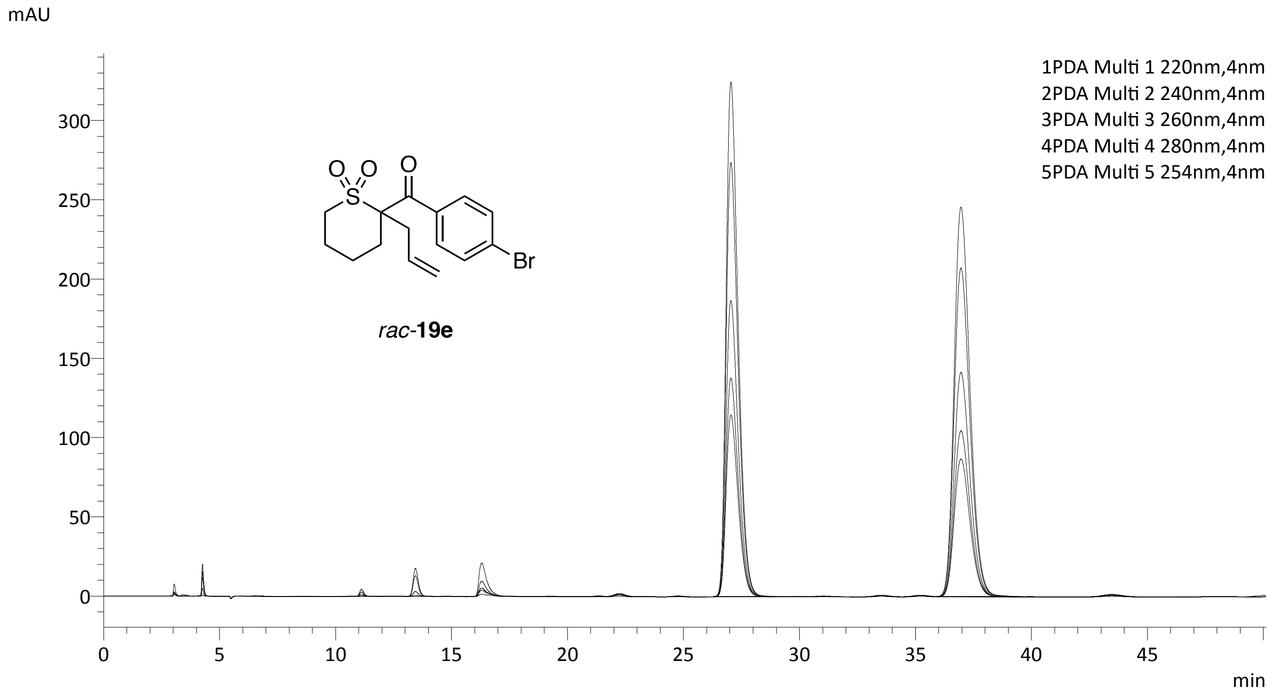

Peak Table

PDA Ch1 220nm

Name	Peak#	Ret. Time	Area	Area%	Mark
	1	27.045	6782657	49.951	
	2	36.963	6795833	50.049	
	Total		13578490	100.000	

PDA Ch2 240nm

Name	Peak#	Ret. Time	Area	Area%	Mark
	1	27.045	4166415	50.070	
	2	36.963	4154695	49.930	
	Total		8321109	100.000	

PDA Ch3 260nm

Name	Peak#	Ret. Time	Area	Area%	Mark
	1	27.045	11767012	49.993	
	2	36.963	11770160	50.007	
	Total		23537172	100.000	

PDA Ch4 280nm

Name	Peak#	Ret. Time	Area	Area%	Mark
	1	27.045	5021536	49.918	
	2	36.963	5037959	50.082	
	Total		10059495	100.000	

PDA Ch5 254nm

Name	Peak#	Ret. Time	Area	Area%	Mark
	1	27.045	9929781	49.987	
	2	36.963	9934929	50.013	
	Total		19864709	100.000	

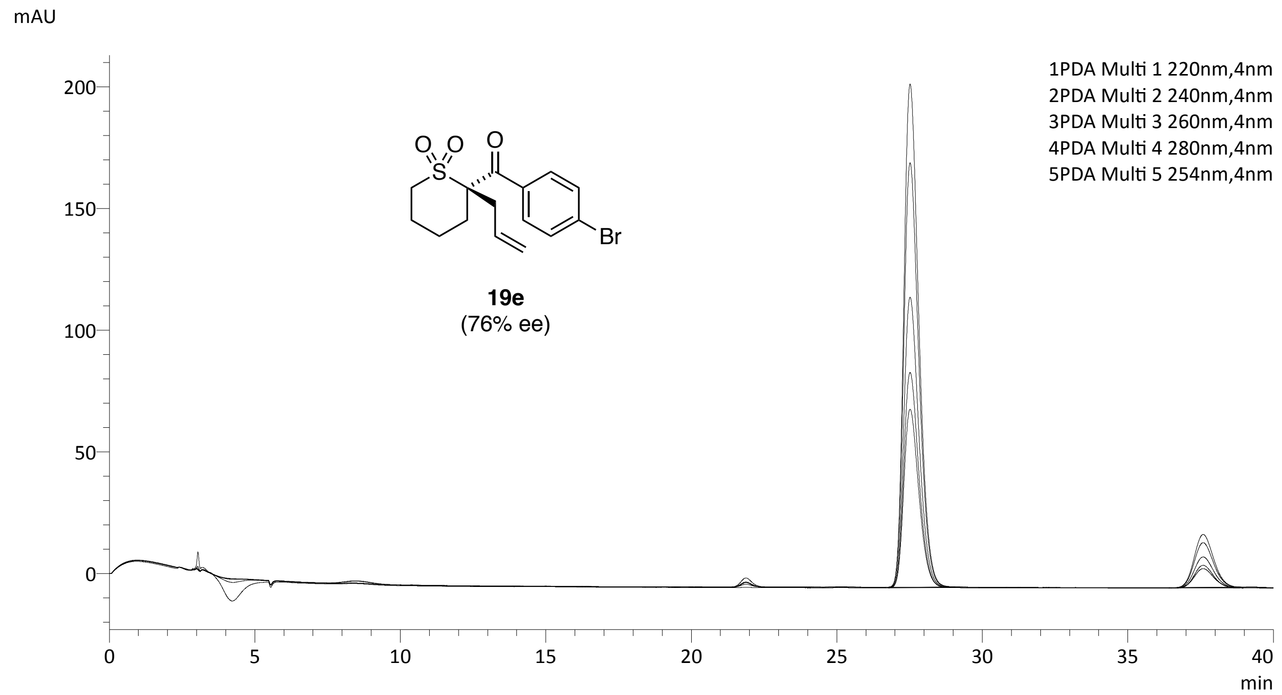

Peak Table

PDA Ch1 220nm

Name	Peak#	Ret. Time	Area	Area%	Mark
	1	27.518	4292192	88.000	
	2	37.590	585277	12.000	
	Total		4877469	100.000	

PDA Ch2 240nm

Name	Peak#	Ret. Time	Area	Area%	Mark
	1	27.518	2633102	88.109	
	2	37.592	355363	11.891	
	Total		2988465	100.000	

PDA Ch3 260nm

Name	Peak#	Ret. Time	Area	Area%	Mark
	1	27.518	7438204	88.000	
	2	37.590	1014267	12.000	
	Total		8452471	100.000	

PDA Ch4 280nm

Name	Peak#	Ret. Time	Area	Area%	Mark
	1	27.518	3185026	88.015	
	2	37.592	433699	11.985	
	Total		3618725	100.000	

PDA Ch5 254nm

Name	Peak#	Ret. Time	Area	Area%	Mark
	1	27.518	6274352	88.007	
	2	37.589	855066	11.993	
	Total		7129418	100.000	

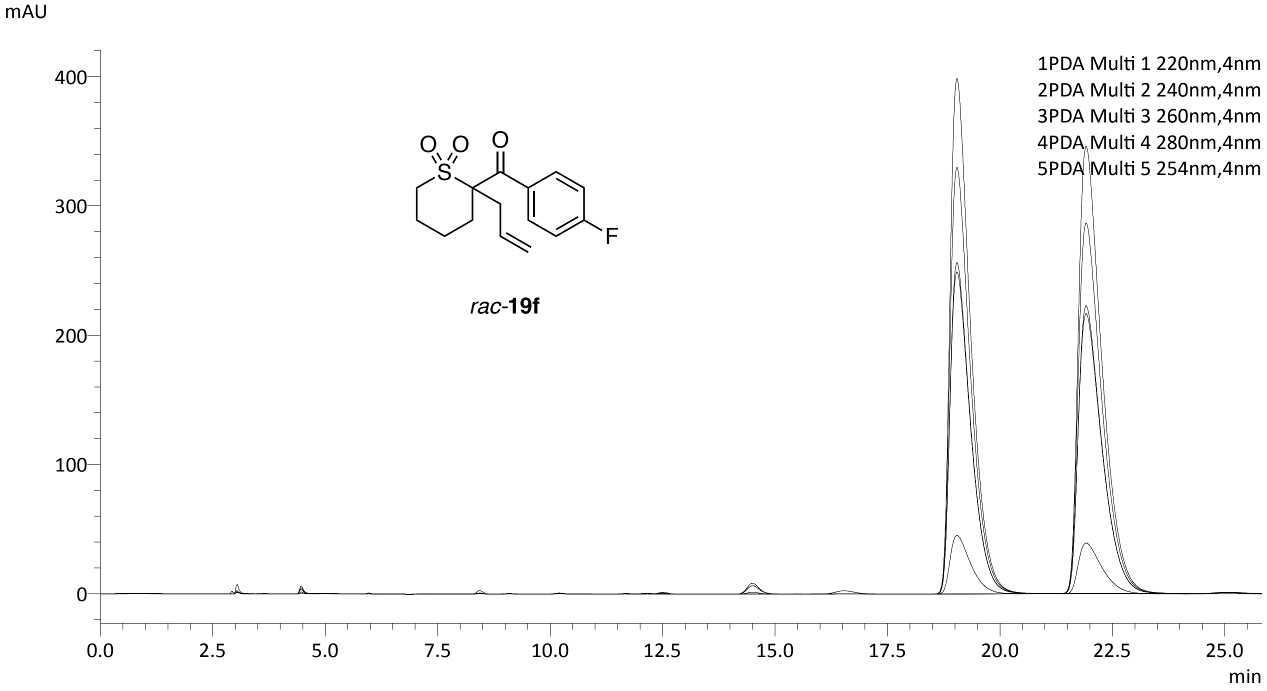

Peak Table

PDA Ch1 220nm

Name	Peak#	Ret. Time	Area	Area%	Mark
	1	19.046	7962522	49.959	
	2	21.918	7975475	50.041	
	Total		15937997	100.000	

PDA Ch2 240nm

Name	Peak#	Ret. Time	Area	Area%	Mark
	1	19.046	8159276	49.994	
	2	21.918	8161174	50.006	
	Total		16320450	100.000	

PDA Ch3 260nm

Name	Peak#	Ret. Time	Area	Area%	Mark
	1	19.046	10514122	49.972	
	2	21.918	10525799	50.028	
	Total		21039921	100.000	

PDA Ch4 280nm

Name	Peak#	Ret. Time	Area	Area%	Mark
	1	19.046	1446277	50.047	
	2	21.918	1443554	49.953	
	Total		2889831	100.000	

PDA Ch5 254nm

Name	Peak#	Ret. Time	Area	Area%	Mark
	1	19.046	12665903	49.995	
	2	21.918	12668563	50.005	
	Total		25334465	100.000	

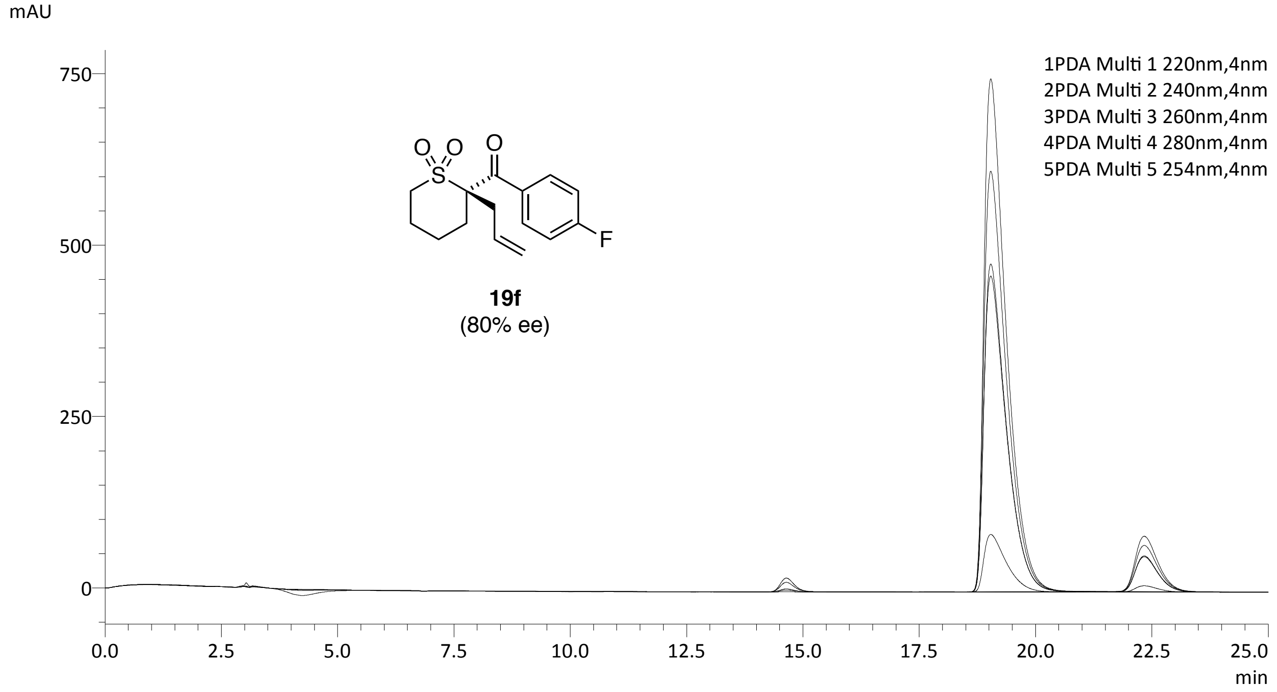

19f
(80% ee)

Peak Table

PDA Ch1 220nm

Name	Peak#	Ret. Time	Area	Area%	Mark
	1	19.039	15522410	89.844	
	2	22.337	1754691	10.156	
	Total		17277101	100.000	

PDA Ch2 240nm

Name	Peak#	Ret. Time	Area	Area%	Mark
	1	19.039	15991126	89.975	
	2	22.337	1781685	10.025	
	Total		17772811	100.000	

PDA Ch3 260nm

Name	Peak#	Ret. Time	Area	Area%	Mark
	1	19.039	20574997	89.859	
	2	22.337	2322105	10.141	
	Total		22897102	100.000	

PDA Ch4 280nm

Name	Peak#	Ret. Time	Area	Area%	Mark
	1	19.039	2828017	89.903	
	2	22.336	317630	10.097	
	Total		3145647	100.000	

PDA Ch5 254nm

Name	Peak#	Ret. Time	Area	Area%	Mark
	1	19.039	24926825	89.961	
	2	22.337	2781793	10.039	
	Total		27708618	100.000	

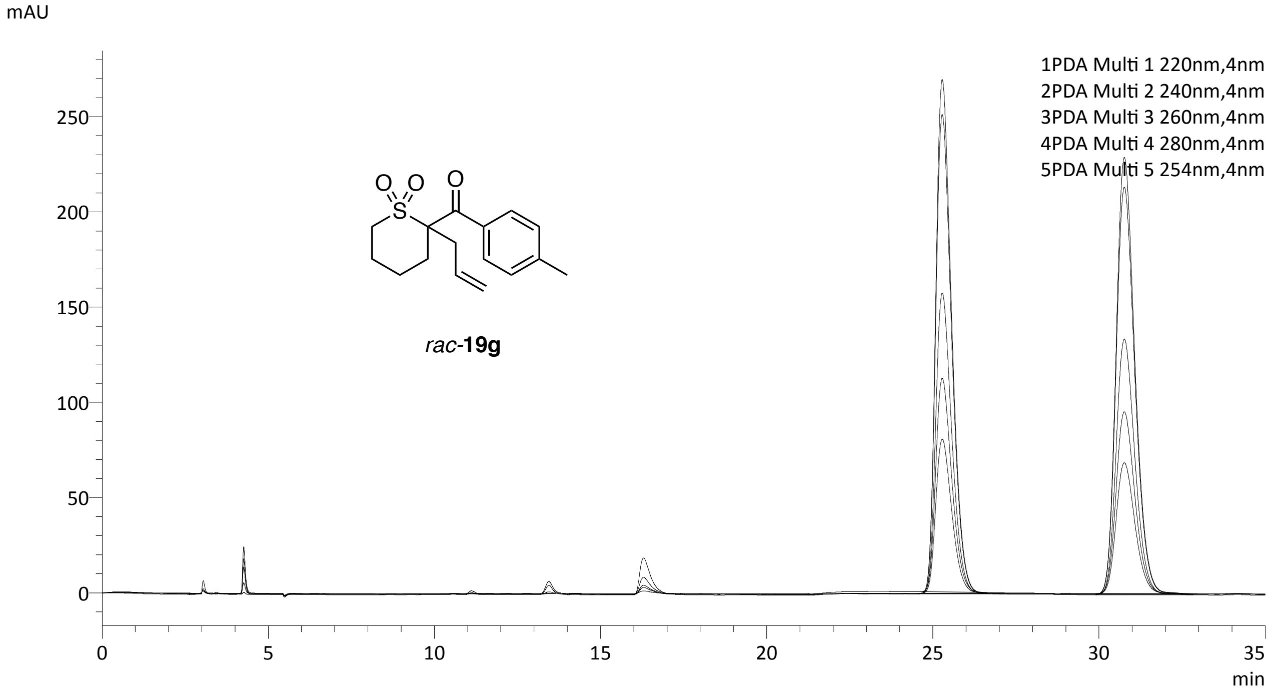

Peak Table

PDA Ch1 220nm

Name	Peak#	Ret. Time	Area	Area%	Mark
	1	25.283	5298952	49.867	
	2	30.763	5327193	50.133	
	Total		10626144	100.000	

PDA Ch2 240nm

Name	Peak#	Ret. Time	Area	Area%	Mark
	1	25.283	3766602	49.840	
	2	30.763	3790743	50.160	
	Total		7557346	100.000	

PDA Ch3 260nm

Name	Peak#	Ret. Time	Area	Area%	Mark
	1	25.283	9061658	49.929	
	2	30.763	9087402	50.071	
	Total		18149061	100.000	

PDA Ch4 280nm

Name	Peak#	Ret. Time	Area	Area%	Mark
	1	25.283	2715965	49.886	
	2	30.763	2728372	50.114	
	Total		5444337	100.000	

PDA Ch5 254nm

Name	Peak#	Ret. Time	Area	Area%	Mark
	1	25.283	8436108	49.916	
	2	30.763	8464640	50.084	
	Total		16900748	100.000	

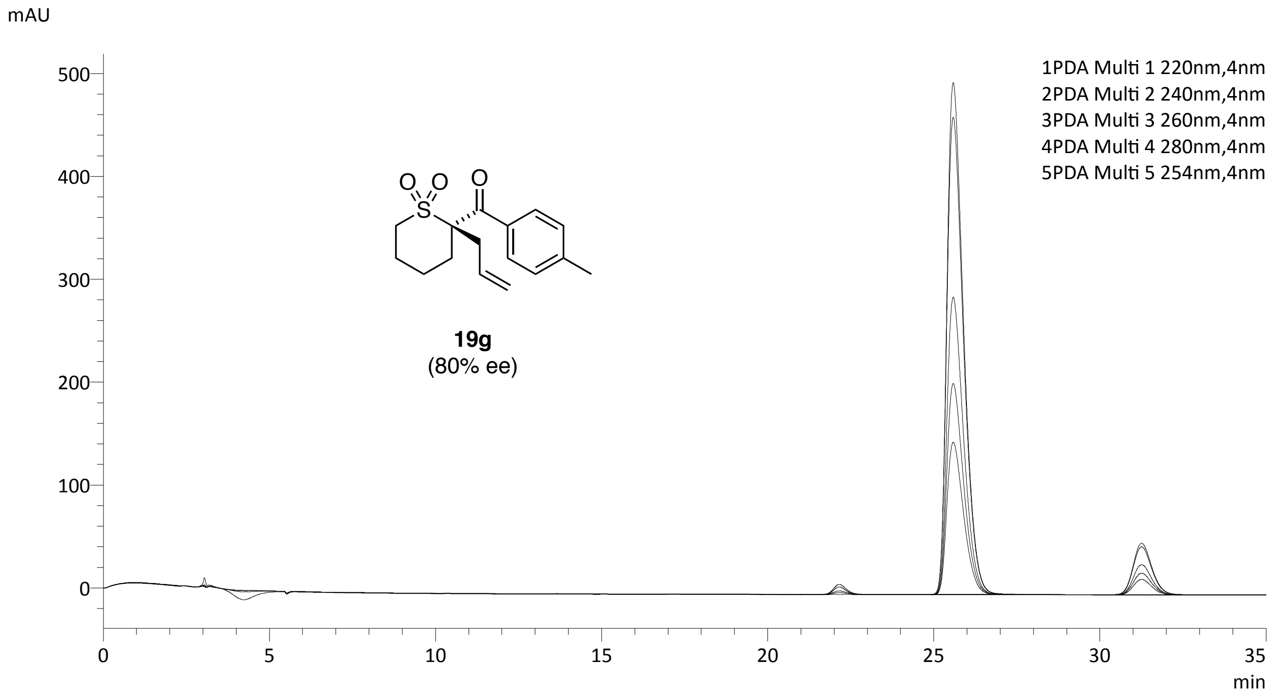

Peak Table

PDA Ch1 220nm

Name	Peak#	Ret. Time	Area	Area%	Mark
	1	25.586	10141925	89.802	
	2	31.258	1151732	10.198	
	Total		11293657	100.000	

PDA Ch2 240nm

Name	Peak#	Ret. Time	Area	Area%	Mark
	1	25.586	7194421	89.810	
	2	31.258	816256	10.190	
	Total		8010677	100.000	

PDA Ch3 260nm

Name	Peak#	Ret. Time	Area	Area%	Mark
	1	25.586	17392311	89.872	
	2	31.258	1959921	10.128	
	Total		19352232	100.000	

PDA Ch4 280nm

Name	Peak#	Ret. Time	Area	Area%	Mark
	1	25.586	5200534	89.834	
	2	31.258	588505	10.166	
	Total		5789039	100.000	

PDA Ch5 254nm

Name	Peak#	Ret. Time	Area	Area%	Mark
	1	25.586	16203467	89.884	
	2	31.258	1823696	10.116	
	Total		18027163	100.000	

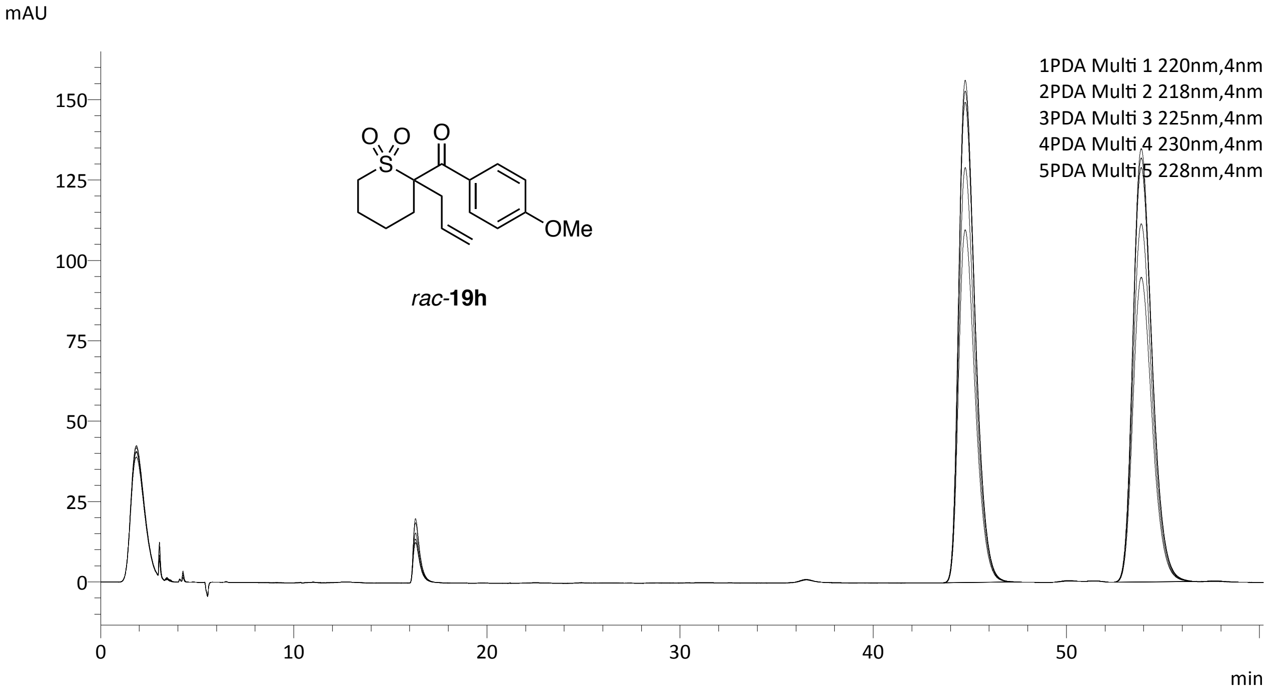

Peak Table

PDA Ch1 220nm

Name	Peak#	Ret. Time	Area	Area%	Mark
	1	44.763	9369900	49.956	
	2	53.877	9386241	50.044	
	Total		18756141	100.000	

PDA Ch2 218nm

Name	Peak#	Ret. Time	Area	Area%	Mark
	1	44.763	9171538	49.969	
	2	53.877	9182870	50.031	
	Total		18354408	100.000	

PDA Ch3 225nm

Name	Peak#	Ret. Time	Area	Area%	Mark
	1	44.763	8964776	49.951	
	2	53.877	8982188	50.049	
	Total		17946963	100.000	

PDA Ch4 230nm

Name	Peak#	Ret. Time	Area	Area%	Mark
	1	44.763	6599178	49.948	
	2	53.877	6612814	50.052	
	Total		13211992	100.000	

PDA Ch5 228nm

Name	Peak#	Ret. Time	Area	Area%	Mark
	1	44.763	7754534	49.954	
	2	53.877	7768689	50.046	
	Total		15523223	100.000	

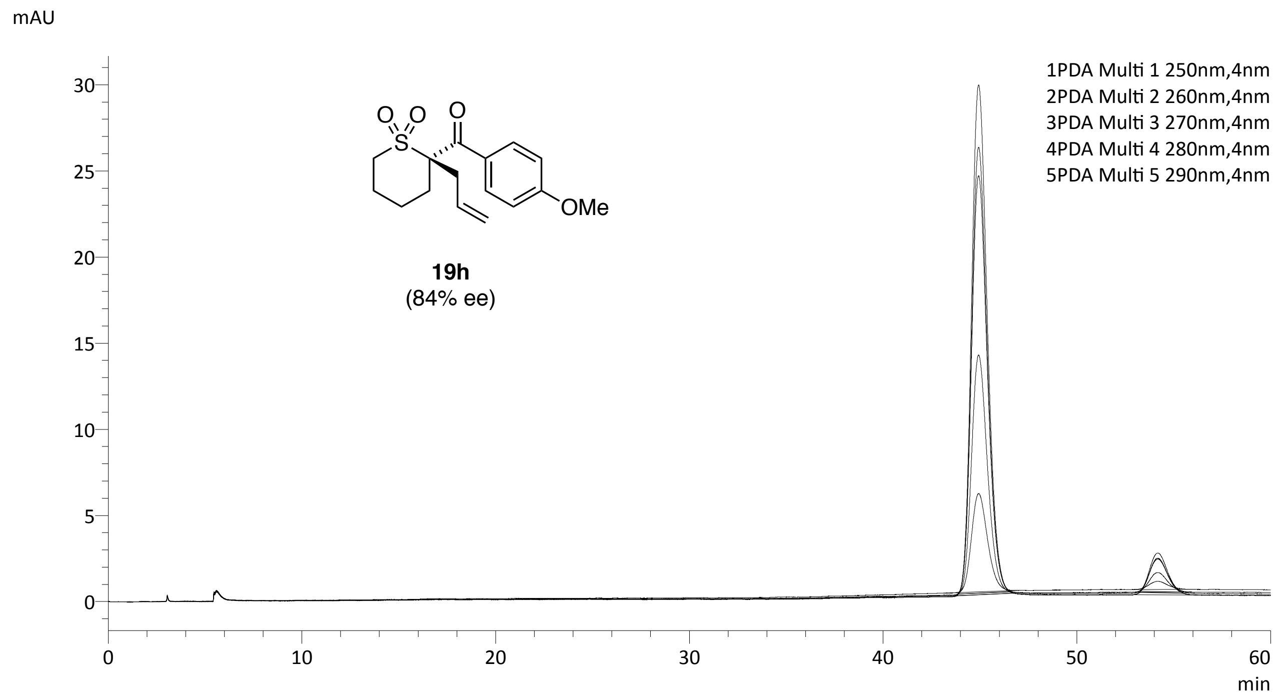

Peak Table

PDA Ch1 250nm

Name	Peak#	Ret. Time	Area	Area%	Mark
	1	44.937	329196	91.084	M
	2	54.167	32224	8.916	M
	Total		361420	100.000	

PDA Ch2 260nm

Name	Peak#	Ret. Time	Area	Area%	Mark
	1	44.934	789431	91.114	
	2	54.181	76987	8.886	M
	Total		866418	100.000	

PDA Ch3 270nm

Name	Peak#	Ret. Time	Area	Area%	Mark
	1	44.931	1383651	92.012	
	2	54.185	120117	7.988	
	Total		1503768	100.000	

PDA Ch4 280nm

Name	Peak#	Ret. Time	Area	Area%	Mark
	1	44.932	1693099	92.022	
	2	54.183	146780	7.978	
	Total		1839879	100.000	

PDA Ch5 290nm

Name	Peak#	Ret. Time	Area	Area%	Mark
	1	44.931	1489761	91.096	M
	2	54.197	145620	8.904	M
	Total		1635382	100.000	

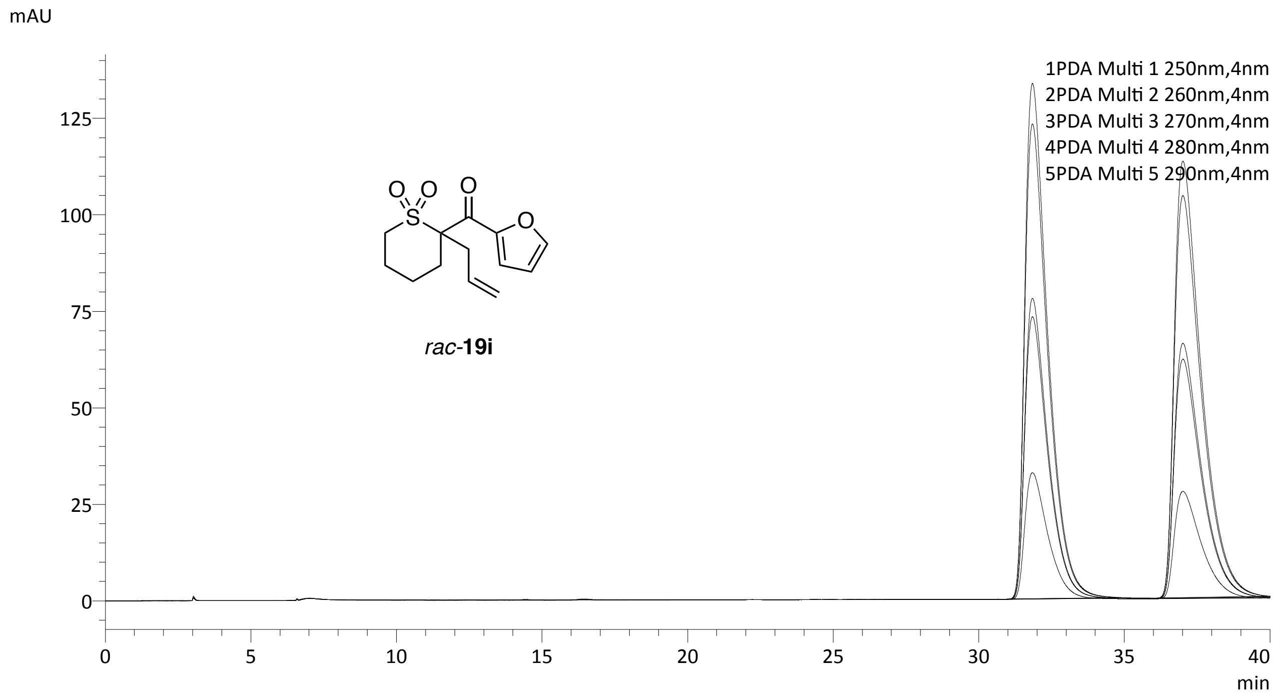

Peak Table

PDA Ch1 250nm

Name	Peak#	Ret. Time	Area	Area%	Mark
	1	31.846	1806211	50.040	
	2	37.014	1803351	49.960	
	Total		3609561	100.000	

PDA Ch2 260nm

Name	Peak#	Ret. Time	Area	Area%	Mark
	1	31.846	4035773	50.016	
	2	37.014	4033189	49.984	
	Total		8068963	100.000	

PDA Ch3 270nm

Name	Peak#	Ret. Time	Area	Area%	Mark
	1	31.846	6815258	50.137	
	2	37.013	6778142	49.863	
	Total		13593400	100.000	

PDA Ch4 280nm

Name	Peak#	Ret. Time	Area	Area%	Mark
	1	31.846	7398571	50.134	
	2	37.013	7359039	49.866	
	Total		14757610	100.000	

PDA Ch5 290nm

Name	Peak#	Ret. Time	Area	Area%	Mark
	1	31.846	4312432	50.038	
	2	37.014	4305912	49.962	
	Total		8618344	100.000	

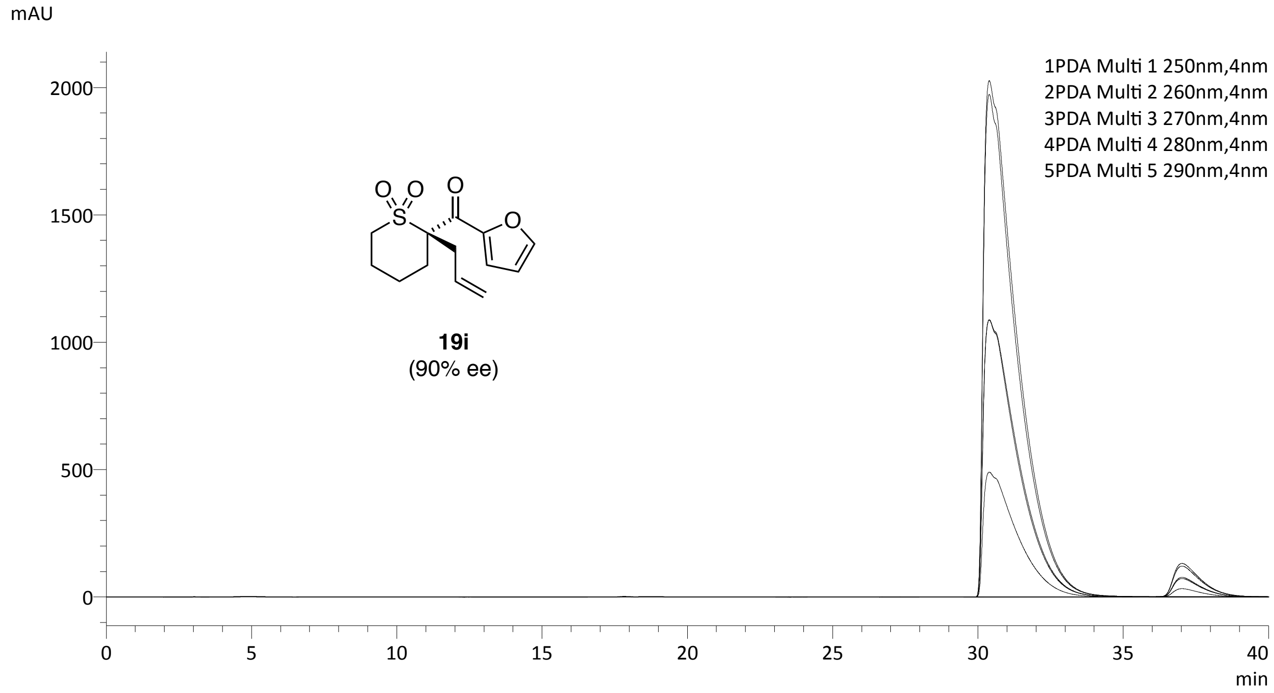

Peak Table

PDA Ch1 250nm

Name	Peak#	Ret. Time	Area	Area%	Mark
	1	30.390	39486844	94.845	
	2	37.024	2146302	5.155	
	Total		41633146	100.000	

PDA Ch2 260nm

Name	Peak#	Ret. Time	Area	Area%	Mark
	1	30.390	87826208	94.846	S
	2	37.025	4772962	5.154	T
	Total		92599170	100.000	

PDA Ch3 270nm

Name	Peak#	Ret. Time	Area	Area%	Mark
	1	30.390	153738023	95.045	S
	2	37.025	8015353	4.955	T
	Total		161753377	100.000	

PDA Ch4 280nm

Name	Peak#	Ret. Time	Area	Area%	Mark
	1	30.391	162218742	94.912	S
	2	37.025	8696351	5.088	T
	Total		170915093	100.000	

PDA Ch5 290nm

Name	Peak#	Ret. Time	Area	Area%	Mark
	1	30.390	90216722	94.666	S
	2	37.025	5083745	5.334	T
	Total		95300467	100.000	

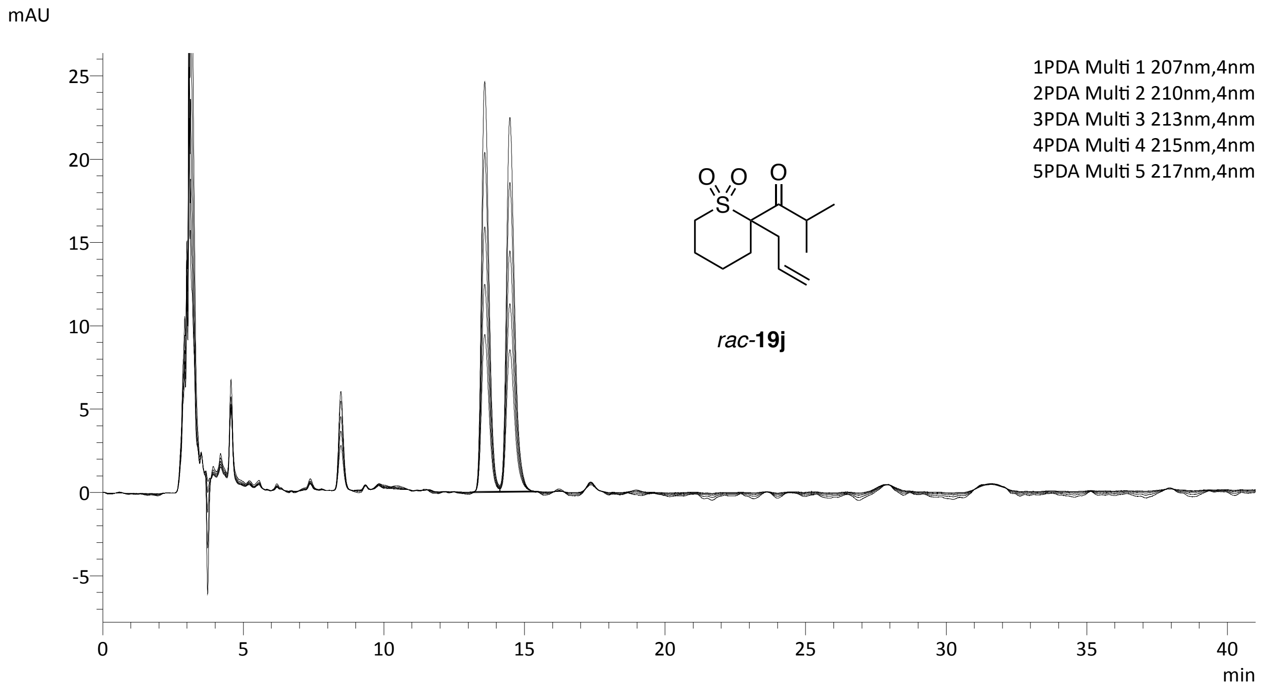

Peak Table

PDA Ch1 207nm

Name	Peak#	Ret. Time	Area	Area%	Mark
	1	13.586	457061	49.834	
	2	14.481	460109	50.166	V
	Total		917170	100.000	

PDA Ch2 210nm

Name	Peak#	Ret. Time	Area	Area%	Mark
	1	13.586	377951	49.844	
	2	14.480	380320	50.156	V
	Total		758271	100.000	

PDA Ch3 213nm

Name	Peak#	Ret. Time	Area	Area%	Mark
	1	13.586	294452	49.840	
	2	14.480	296348	50.160	V
	Total		590800	100.000	

PDA Ch4 215nm

Name	Peak#	Ret. Time	Area	Area%	Mark
	1	13.586	230688	49.826	
	2	14.480	232302	50.174	V
	Total		462991	100.000	

PDA Ch5 217nm

Name	Peak#	Ret. Time	Area	Area%	Mark
	1	13.586	174949	49.852	
	2	14.481	175991	50.148	V
	Total		350940	100.000	

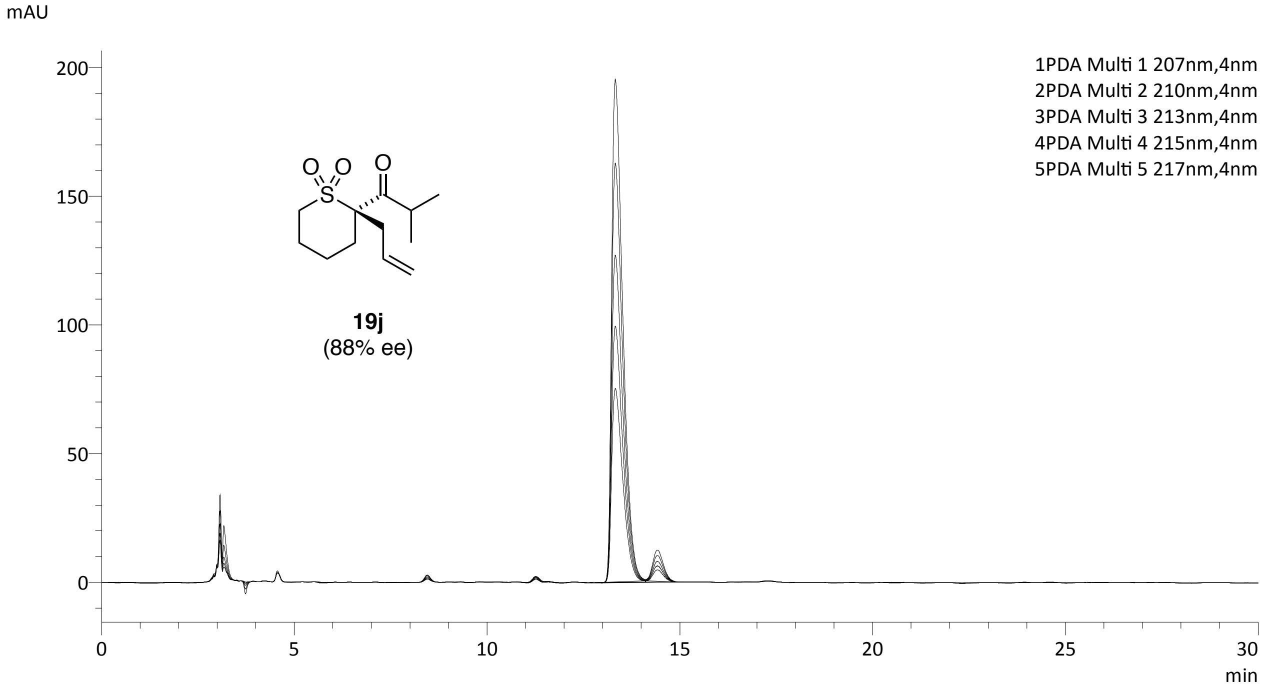

Peak Table

PDA Ch1 207nm

Name	Peak#	Ret. Time	Area	Area%	Mark
	1	13.329	3986333	94.026	
	2	14.421	253259	5.974	V
	Total		4239592	100.000	

PDA Ch2 210nm

Name	Peak#	Ret. Time	Area	Area%	Mark
	1	13.329	3311160	94.060	
	2	14.421	209094	5.940	V
	Total		3520254	100.000	

PDA Ch3 213nm

Name	Peak#	Ret. Time	Area	Area%	Mark
	1	13.329	2581247	94.067	
	2	14.421	162800	5.933	V
	Total		2744047	100.000	

PDA Ch4 215nm

Name	Peak#	Ret. Time	Area	Area%	Mark
	1	13.329	1999997	94.738	M
	2	14.421	111079	5.262	
	Total		2111076	100.000	

PDA Ch5 217nm

Name	Peak#	Ret. Time	Area	Area%	Mark
	1	13.329	1528747	94.066	
	2	14.421	96437	5.934	V
	Total		1625184	100.000	

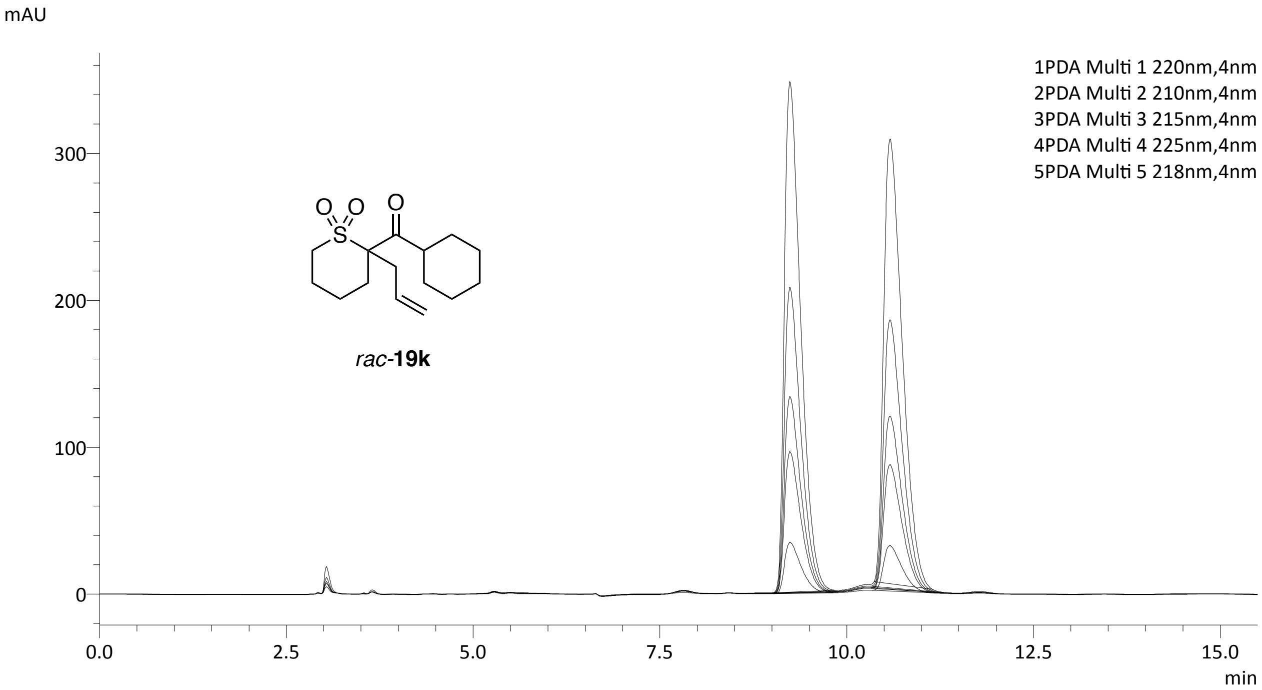

Peak Table

PDA Ch1 220nm

Name	Peak#	Ret. Time	Area	Area%	Mark
	1	9.241	1478862	50.231	
	2	10.578	1465287	49.769	
	Total		2944148	100.000	

PDA Ch2 210nm

Name	Peak#	Ret. Time	Area	Area%	Mark
	1	9.240	5320172	50.322	
	2	10.580	5252057	49.678	M
	Total		10572228	100.000	

PDA Ch3 215nm

Name	Peak#	Ret. Time	Area	Area%	Mark
	1	9.240	3178481	50.042	
	2	10.580	3173139	49.958	
	Total		6351620	100.000	

PDA Ch4 225nm

Name	Peak#	Ret. Time	Area	Area%	Mark
	1	9.242	537946	50.300	
	2	10.577	531520	49.700	
	Total		1069466	100.000	

PDA Ch5 218nm

Name	Peak#	Ret. Time	Area	Area%	Mark
	1	9.240	2048066	50.152	
	2	10.579	2035677	49.848	
	Total		4083743	100.000	

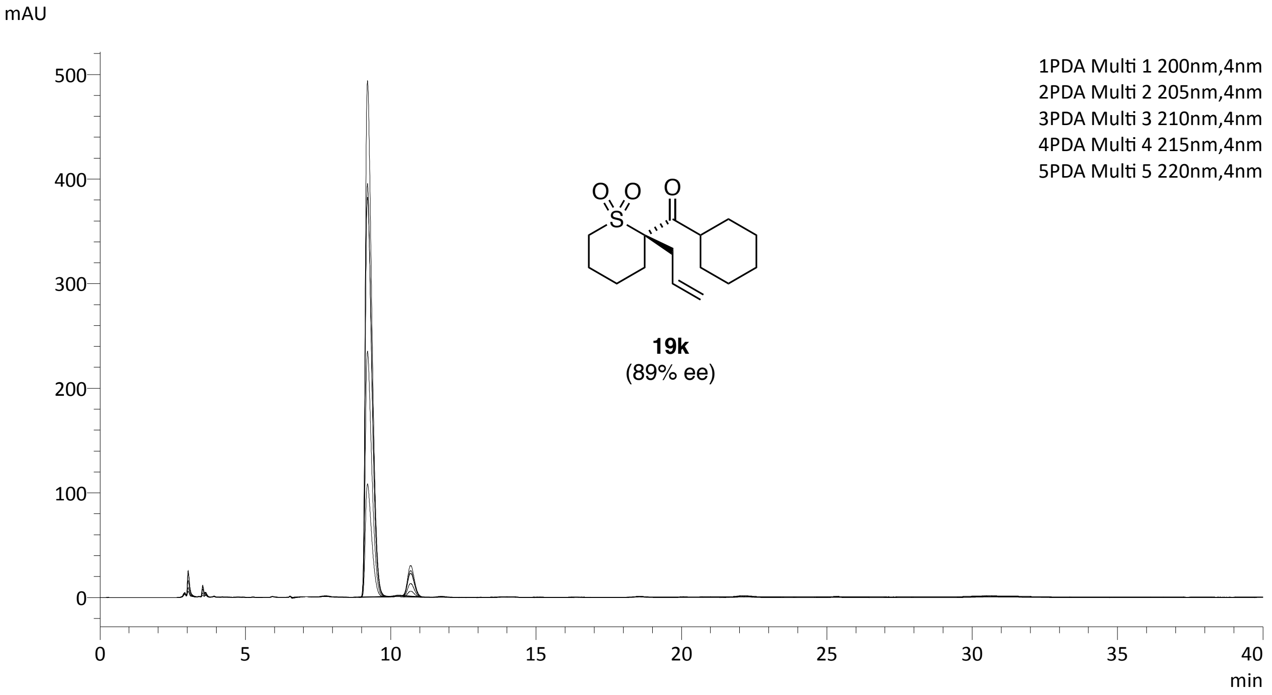

Peak Table

PDA Ch1 200nm

Name	Peak#	Ret. Time	Area	Area%	Mark
	1	9.201	6238630	94.049	V
	2	10.683	394750	5.951	M
	Total		6633380	100.000	

PDA Ch2 205nm

Name	Peak#	Ret. Time	Area	Area%	Mark
	1	9.201	7849568	94.312	
	2	10.685	473416	5.688	
	Total		8322984	100.000	

PDA Ch3 210nm

Name	Peak#	Ret. Time	Area	Area%	Mark
	1	9.201	6155929	94.546	
	2	10.685	355129	5.454	M
	Total		6511058	100.000	

PDA Ch4 215nm

Name	Peak#	Ret. Time	Area	Area%	Mark
	1	9.201	3631548	94.663	
	2	10.685	204760	5.337	M
	Total		3836308	100.000	

PDA Ch5 220nm

Name	Peak#	Ret. Time	Area	Area%	Mark
	1	9.201	1663313	94.834	
	2	10.687	90611	5.166	M
	Total		1753924	100.000	

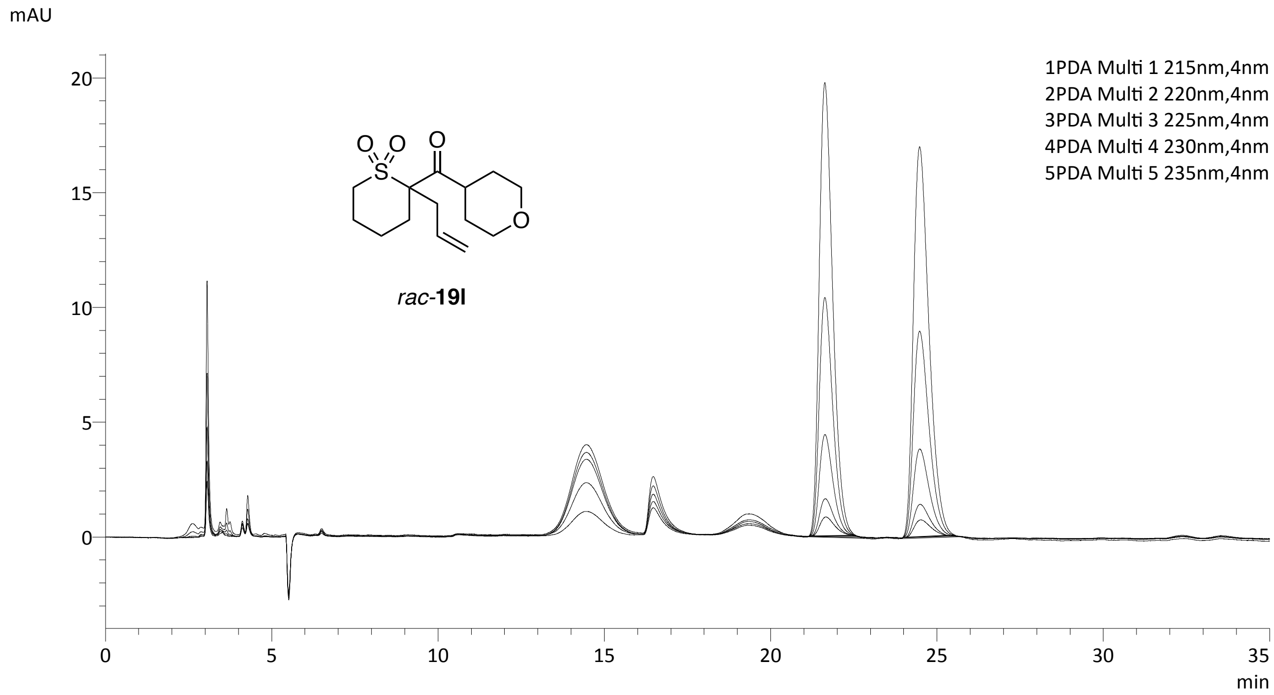

Peak Table

PDA Ch1 215nm

Name	Peak#	Ret. Time	Area	Area%	Mark
	1	21.629	568302	50.201	
	2	24.479	563752	49.799	
	Total		1132054	100.000	

PDA Ch2 220nm

Name	Peak#	Ret. Time	Area	Area%	Mark
	1	21.631	300868	50.256	
	2	24.480	297806	49.744	
	Total		598674	100.000	

PDA Ch3 225nm

Name	Peak#	Ret. Time	Area	Area%	Mark
	1	21.634	129703	50.550	
	2	24.483	126879	49.450	
	Total		256583	100.000	

PDA Ch4 230nm

Name	Peak#	Ret. Time	Area	Area%	Mark
	1	21.644	48500	50.245	
	2	24.497	48027	49.755	
	Total		96526	100.000	

PDA Ch5 235nm

Name	Peak#	Ret. Time	Area	Area%	Mark
	1	21.661	24284	49.757	
	2	24.510	24522	50.243	
	Total		48807	100.000	

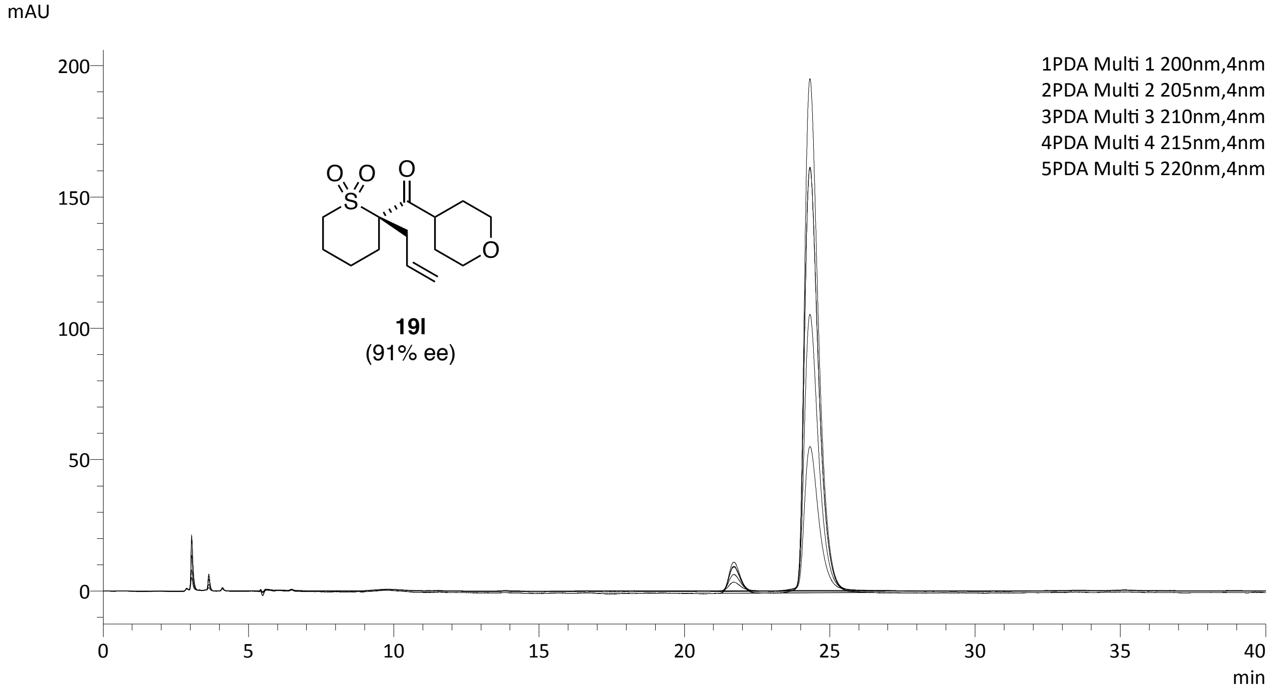

Peak Table

PDA Ch1 200nm

Name	Peak#	Ret. Time	Area	Area%	Mark
	1	21.702	281094	4.808	
	2	24.320	5564941	95.192	SV
	Total		5846036	100.000	

PDA Ch2 205nm

Name	Peak#	Ret. Time	Area	Area%	Mark
	1	21.701	329331	4.714	
	2	24.320	6656306	95.286	
	Total		6985637	100.000	

PDA Ch3 210nm

Name	Peak#	Ret. Time	Area	Area%	Mark
	1	21.702	267273	4.638	
	2	24.320	5495070	95.362	M
	Total		5762343	100.000	

PDA Ch4 215nm

Name	Peak#	Ret. Time	Area	Area%	Mark
	1	21.702	173837	4.641	
	2	24.320	3571926	95.359	M
	Total		3745764	100.000	

PDA Ch5 220nm

Name	Peak#	Ret. Time	Area	Area%	Mark
	1	21.706	89620	4.491	
	2	24.321	1906064	95.509	M
	Total		1995683	100.000	

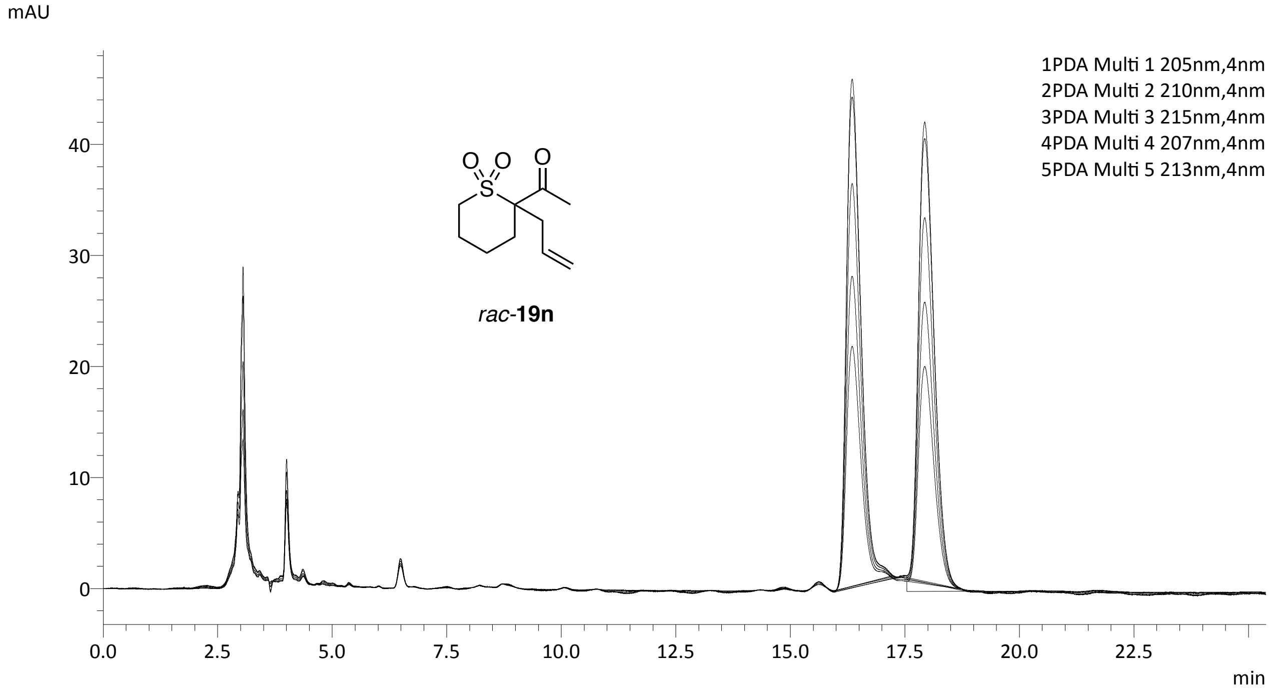

Peak Table

PDA Ch1 205nm

Name	Peak#	Ret. Time	Area	Area%	Mark
	1	16.349	1029280	50.785	M
	2	17.933	997442	49.215	M
	Total		2026722	100.000	

PDA Ch2 210nm

Name	Peak#	Ret. Time	Area	Area%	Mark
	1	16.349	806603	50.793	M
	2	17.933	781416	49.207	
	Total		1588018	100.000	

PDA Ch3 215nm

Name	Peak#	Ret. Time	Area	Area%	Mark
	1	16.350	476986	50.610	M
	2	17.933	465484	49.390	
	Total		942470	100.000	

PDA Ch4 207nm

Name	Peak#	Ret. Time	Area	Area%	Mark
	1	16.349	995414	49.739	M
	2	17.932	1005856	50.261	
	Total		2001271	100.000	

PDA Ch5 213nm

Name	Peak#	Ret. Time	Area	Area%	Mark
	1	16.349	622847	50.843	M
	2	17.933	602185	49.157	
	Total		1225032	100.000	

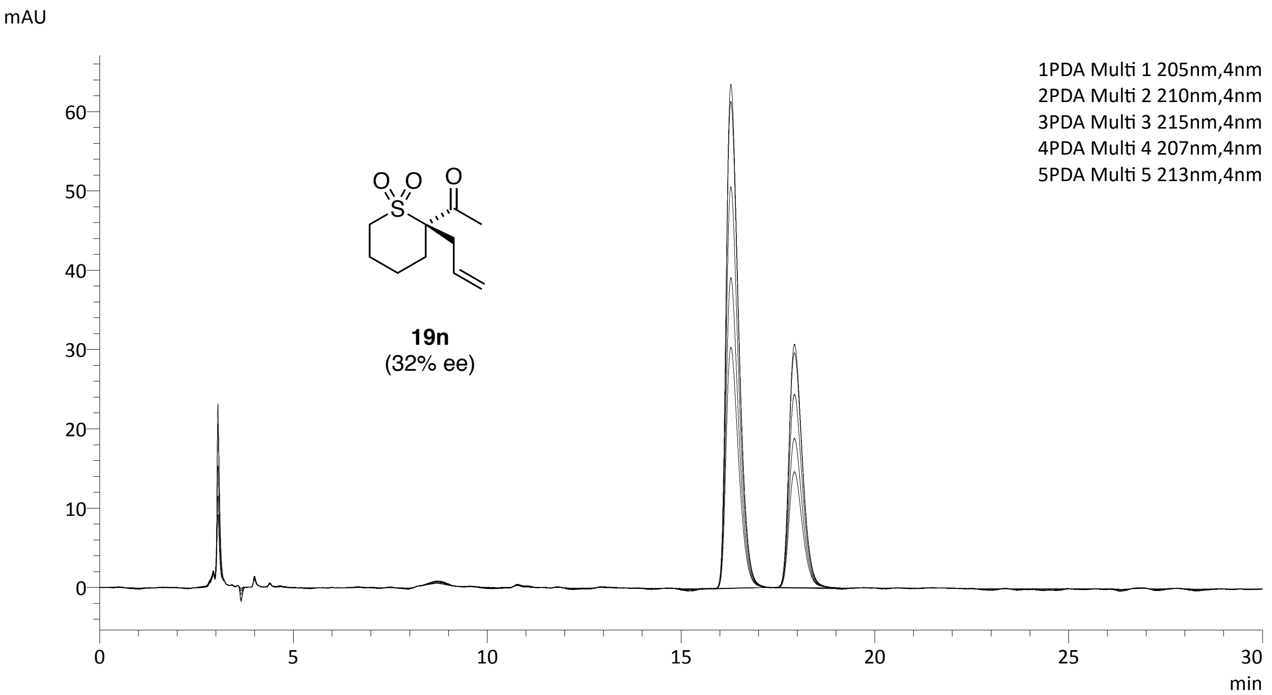

Peak Table

PDA Ch1 205nm

Name	Peak#	Ret. Time	Area	Area%	Mark
	1	16.287	1435128	65.764	
	2	17.927	747122	34.236	
	Total		2182250	100.000	

PDA Ch2 210nm

Name	Peak#	Ret. Time	Area	Area%	Mark
	1	16.287	1143803	65.899	
	2	17.927	591882	34.101	
	Total		1735684	100.000	

PDA Ch3 215nm

Name	Peak#	Ret. Time	Area	Area%	Mark
	1	16.288	686706	66.028	
	2	17.928	353314	33.972	
	Total		1040020	100.000	

PDA Ch4 207nm

Name	Peak#	Ret. Time	Area	Area%	Mark
	1	16.288	1385961	65.848	
	2	17.928	718830	34.152	
	Total		2104791	100.000	

PDA Ch5 213nm

Name	Peak#	Ret. Time	Area	Area%	Mark
	1	16.288	884041	65.966	
	2	17.928	456100	34.034	
	Total		1340141	100.000	

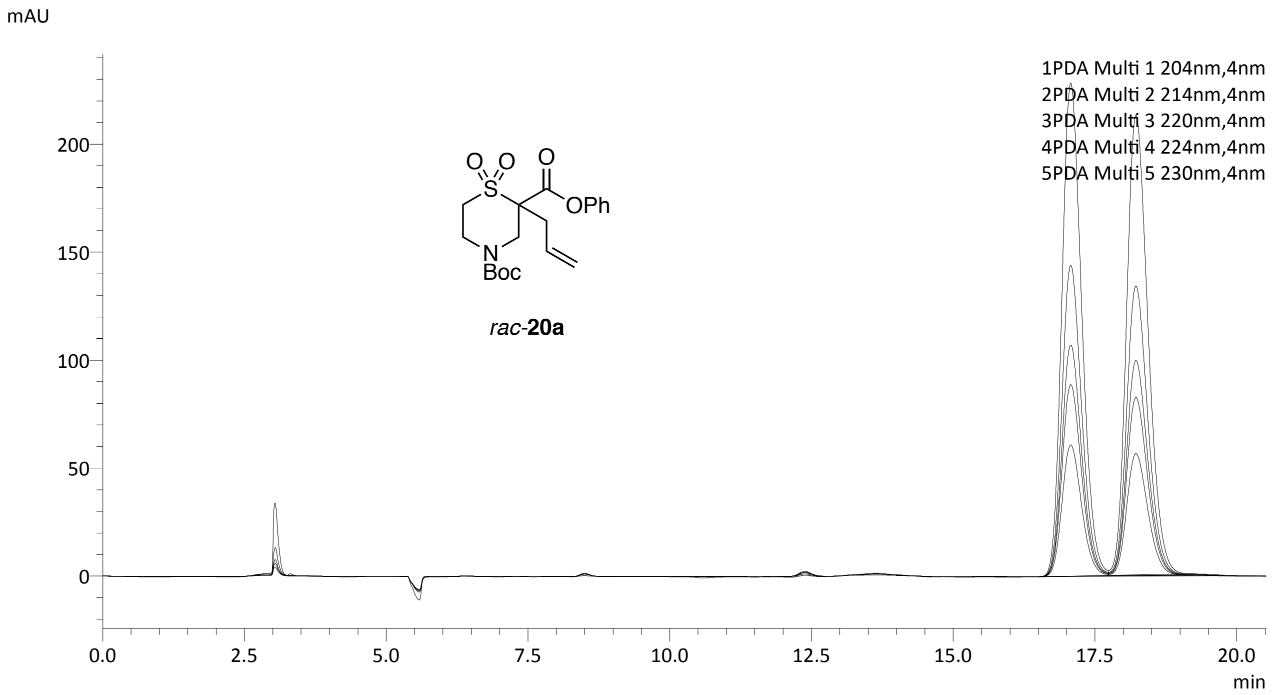

Peak Table

PDA Ch1 204nm

Name	Peak#	Ret. Time	Area	Area%	Mark
	1	17.072	5576748	49.793	
	2	18.222	5623006	50.207	V
	Total		11199754	100.000	

PDA Ch2 214nm

Name	Peak#	Ret. Time	Area	Area%	Mark
	1	17.072	3496539	49.488	
	2	18.222	3568955	50.512	V
	Total		7065494	100.000	

PDA Ch3 220nm

Name	Peak#	Ret. Time	Area	Area%	Mark
	1	17.072	2588306	49.820	
	2	18.222	2606972	50.180	V
	Total		5195279	100.000	

PDA Ch4 224nm

Name	Peak#	Ret. Time	Area	Area%	Mark
	1	17.072	2143278	49.825	
	2	18.222	2158337	50.175	V
	Total		4301614	100.000	

PDA Ch5 230nm

Name	Peak#	Ret. Time	Area	Area%	Mark
	1	17.072	1472079	49.824	
	2	18.222	1482486	50.176	V
	Total		2954565	100.000	

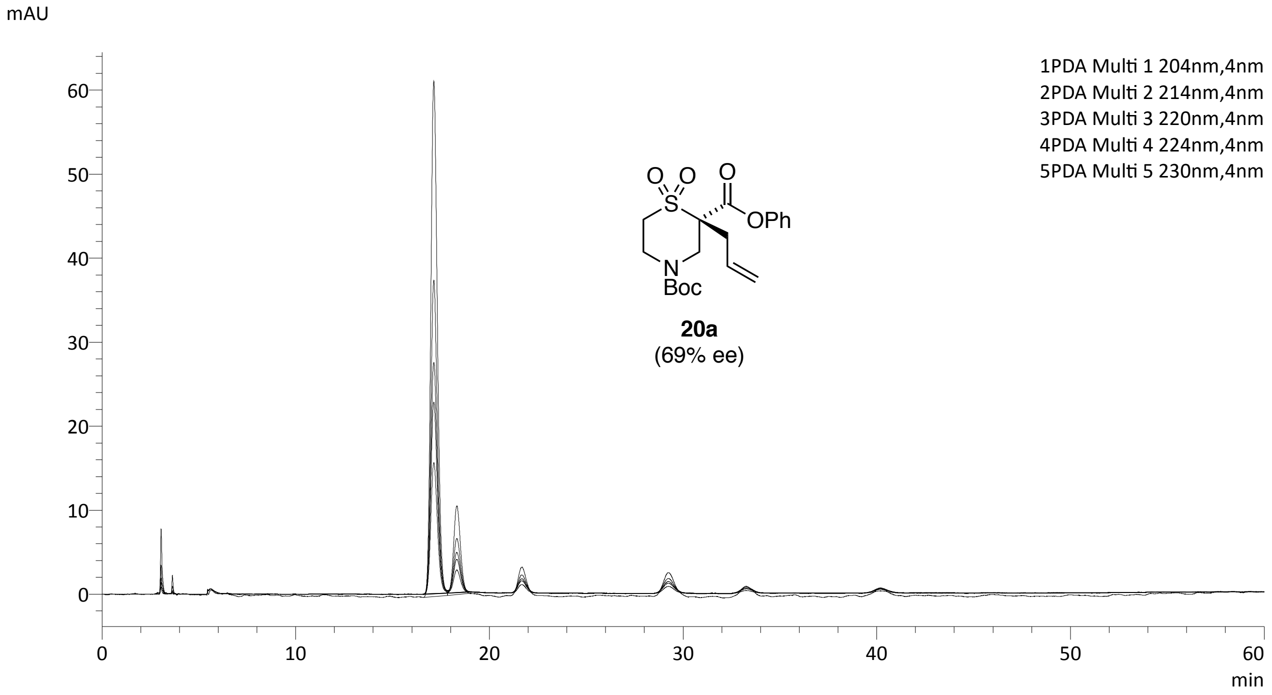

Peak Table

PDA Ch1 204nm

Name	Peak#	Ret. Time	Area	Area%	Mark
	1	17.132	1451589	84.561	
	2	18.321	265036	15.439	V
	Total		1716625	100.000	

PDA Ch2 214nm

Name	Peak#	Ret. Time	Area	Area%	Mark
	1	17.133	884854	84.344	
	2	18.321	164250	15.656	V
	Total		1049104	100.000	

PDA Ch3 220nm

Name	Peak#	Ret. Time	Area	Area%	Mark
	1	17.133	652467	84.175	
	2	18.321	122665	15.825	V
	Total		775132	100.000	

PDA Ch4 224nm

Name	Peak#	Ret. Time	Area	Area%	Mark
	1	17.133	540538	84.183	
	2	18.320	101561	15.817	V
	Total		642099	100.000	

PDA Ch5 230nm

Name	Peak#	Ret. Time	Area	Area%	Mark
	1	17.133	372056	84.119	
	2	18.321	70241	15.881	V
	Total		442297	100.000	

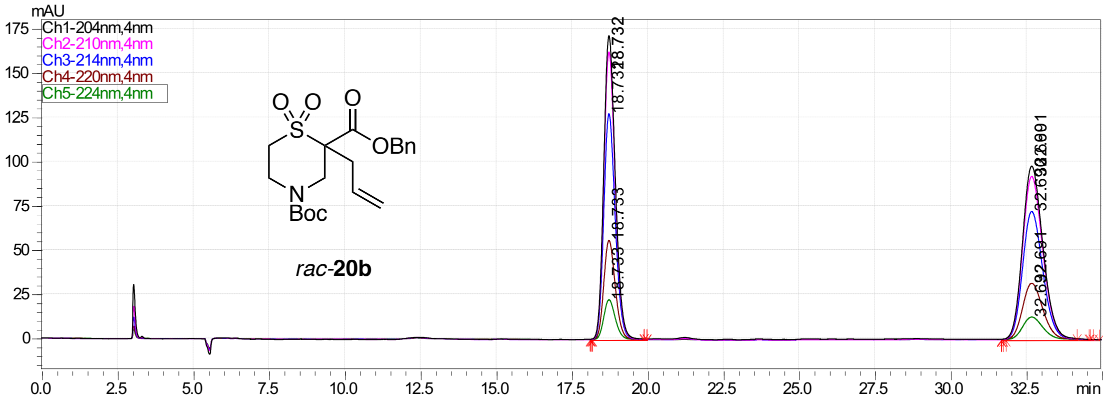

204 nm

Peak#	Ret. Time	Area	Area%
1	18.732	4548634	49.714
2	32.691	4600970	50.286
Total		9149604	100.000

210 nm

Peak#	Ret. Time	Area	Area%
1	18.732	4292817	49.718
2	32.690	4341467	50.282
Total		8634284	100.000

214 nm

Peak#	Ret. Time	Area	Area%
1	18.732	3372823	49.756
2	32.690	3405940	50.244
Total	6778763	100.000	

220 nm

Peak#	Ret. Time	Area	Area%
1	18.733	1486618	49.752
2	32.691	1501442	50.248
Total		2988061	100.000

224 nm

Peak#	Ret. Time	Area	Area%
1	18.733	596713	50.178
2	32.692	592473	49.822
Total		1189186	100.000

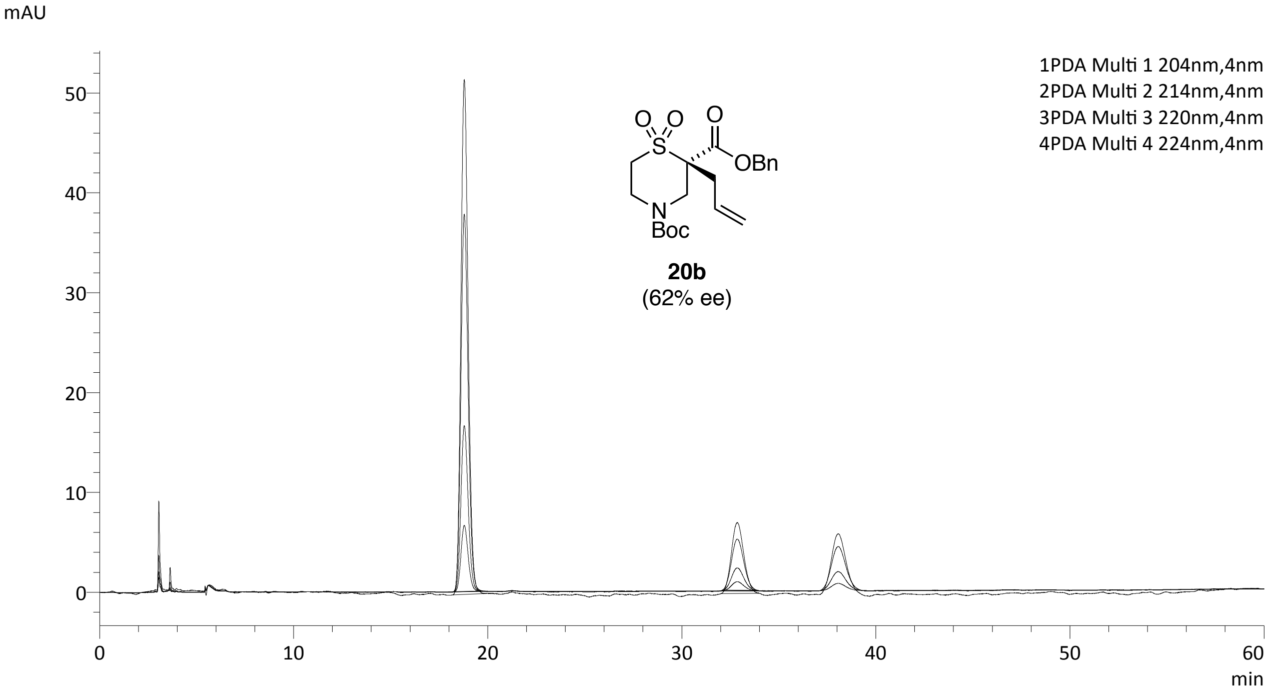

20b
(62% ee)

Peak Table

PDA Ch1 204nm

Name	Peak#	Ret. Time	Area	Area%	Mark
	1	18.793	1342997	80.818	
	2	32.867	318767	19.182	
	Total		1661764	100.000	

PDA Ch2 214nm

Name	Peak#	Ret. Time	Area	Area%	Mark
	1	18.793	981947	80.928	
	2	32.856	231405	19.072	
	Total		1213352	100.000	

PDA Ch3 220nm

Name	Peak#	Ret. Time	Area	Area%	Mark
	1	18.793	430837	81.478	
	2	32.859	97941	18.522	
	Total		528778	100.000	

PDA Ch4 224nm

Name	Peak#	Ret. Time	Area	Area%	Mark
	1	18.793	173763	80.052	M
	2	32.855	43300	19.948	M
	Total		217063	100.000	

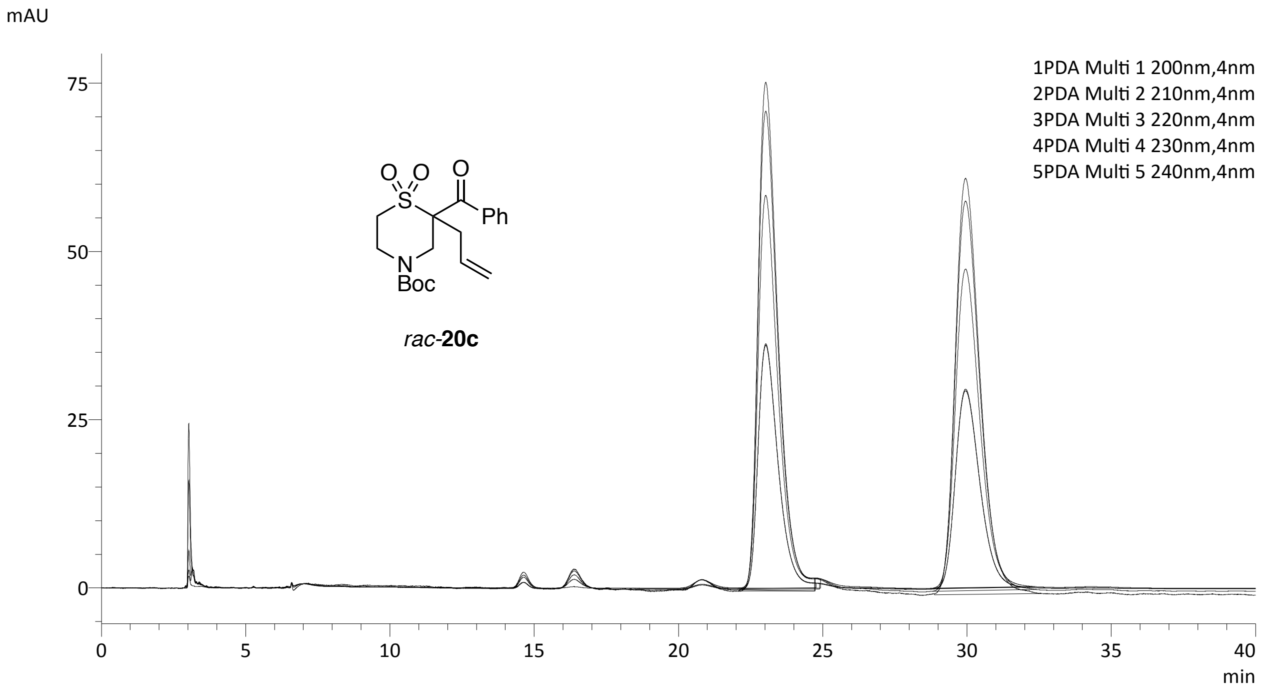

Peak Table

PDA Ch1 200nm

Name	Peak#	Ret. Time	Area	Area%	Mark
	1	23.024	3683297	49.757	M
	2	29.948	3719328	50.243	SV
	Total		7402625	100.000	

PDA Ch2 210nm

Name	Peak#	Ret. Time	Area	Area%	Mark
	1	23.026	3472102	50.137	M
	2	29.951	3453192	49.863	
	Total		6925294	100.000	

PDA Ch3 220nm

Name	Peak#	Ret. Time	Area	Area%	Mark
	1	23.027	1790017	50.418	M
	2	29.953	1760369	49.582	
	Total		3550386	100.000	

PDA Ch4 230nm

Name	Peak#	Ret. Time	Area	Area%	Mark
	1	23.026	1752847	50.153	M
	2	29.952	1742174	49.847	
	Total		3495021	100.000	

PDA Ch5 240nm

Name	Peak#	Ret. Time	Area	Area%	Mark
	1	23.025	2849278	50.330	M
	2	29.950	2811963	49.670	
	Total		5661242	100.000	

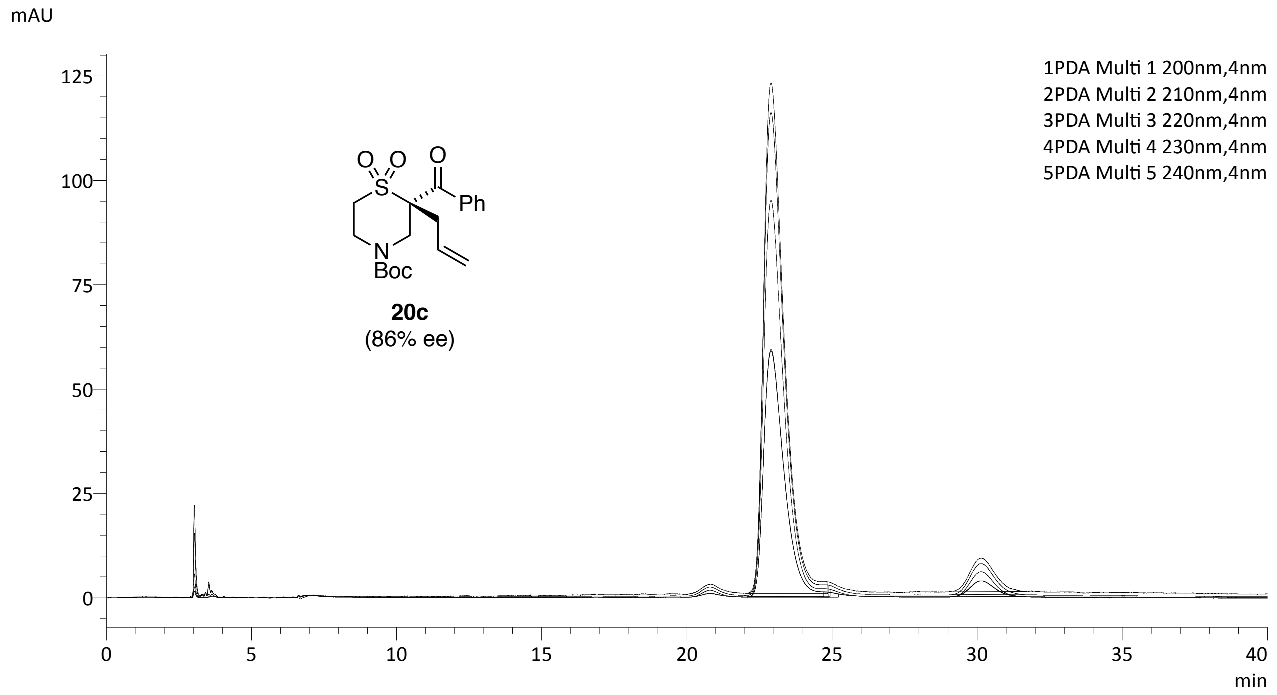

Peak Table

PDA Ch1 200nm

Name	Peak#	Ret. Time	Area	Area%	Mark
	1	22.902	6037588	92.771	M
	2	30.156	470439	7.229	S
	Total		6508027	100.000	

PDA Ch2 210nm

Name	Peak#	Ret. Time	Area	Area%	Mark
	1	22.901	5724484	92.940	M
	2	30.143	434841	7.060	
	Total		6159325	100.000	

PDA Ch3 220nm

Name	Peak#	Ret. Time	Area	Area%	Mark
	1	22.902	2947439	93.099	M
	2	30.156	218484	6.901	
	Total		3165923	100.000	

PDA Ch4 230nm

Name	Peak#	Ret. Time	Area	Area%	Mark
	1	22.902	2873201	93.019	M
	2	30.130	215647	6.981	
	Total		3088847	100.000	

PDA Ch5 240nm

Name	Peak#	Ret. Time	Area	Area%	Mark
	1	22.900	4632864	92.956	M
	2	30.143	351065	7.044	
	Total		4983929	100.000	

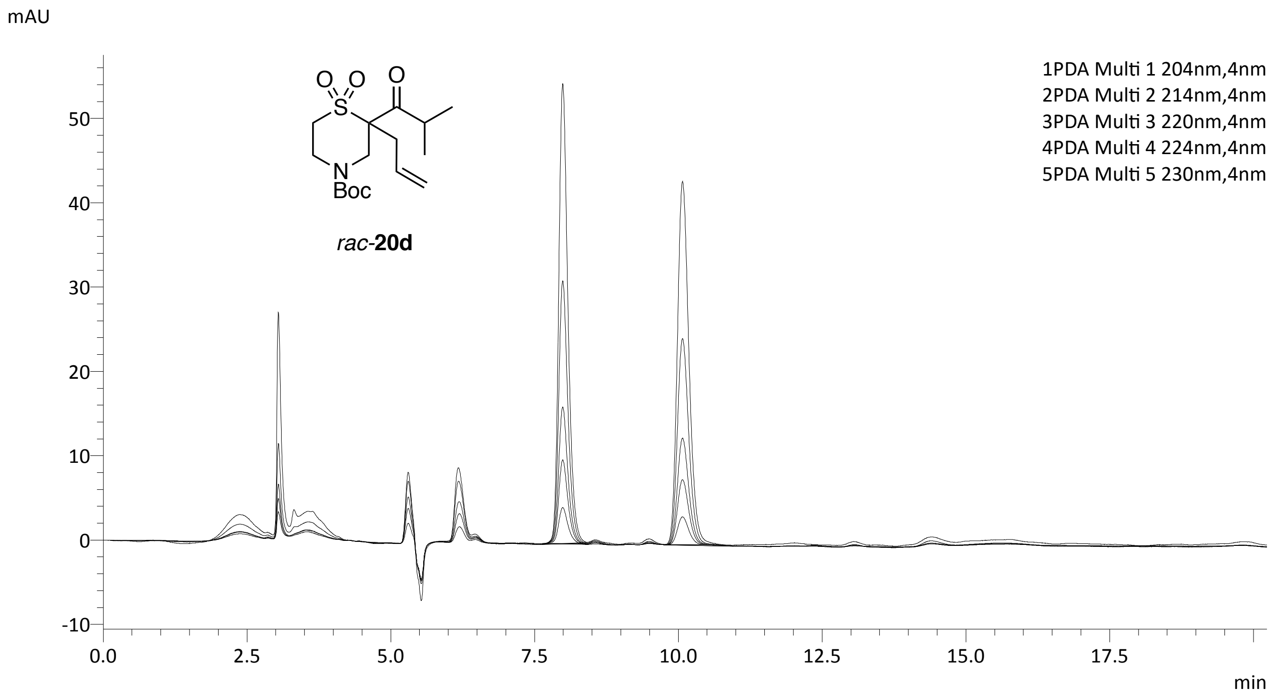

Peak Table

PDA Ch1 204nm

Name	Peak#	Ret. Time	Area	Area%	Mark
	1	7.990	609073	50.182	
	2	10.073	604666	49.818	
	Total		1213739	100.000	

PDA Ch2 214nm

Name	Peak#	Ret. Time	Area	Area%	Mark
	1	7.990	350687	50.479	
	2	10.073	344035	49.521	
	Total		694723	100.000	

PDA Ch3 220nm

Name	Peak#	Ret. Time	Area	Area%	Mark
	1	7.990	184459	50.734	
	2	10.073	179120	49.266	
	Total		363579	100.000	

PDA Ch4 224nm

Name	Peak#	Ret. Time	Area	Area%	Mark
	1	7.990	114447	51.947	
	2	10.073	105869	48.053	
	Total		220316	100.000	

PDA Ch5 230nm

Name	Peak#	Ret. Time	Area	Area%	Mark
	1	7.990	48800	52.215	M
	2	10.073	44659	47.785	
	Total		93459	100.000	

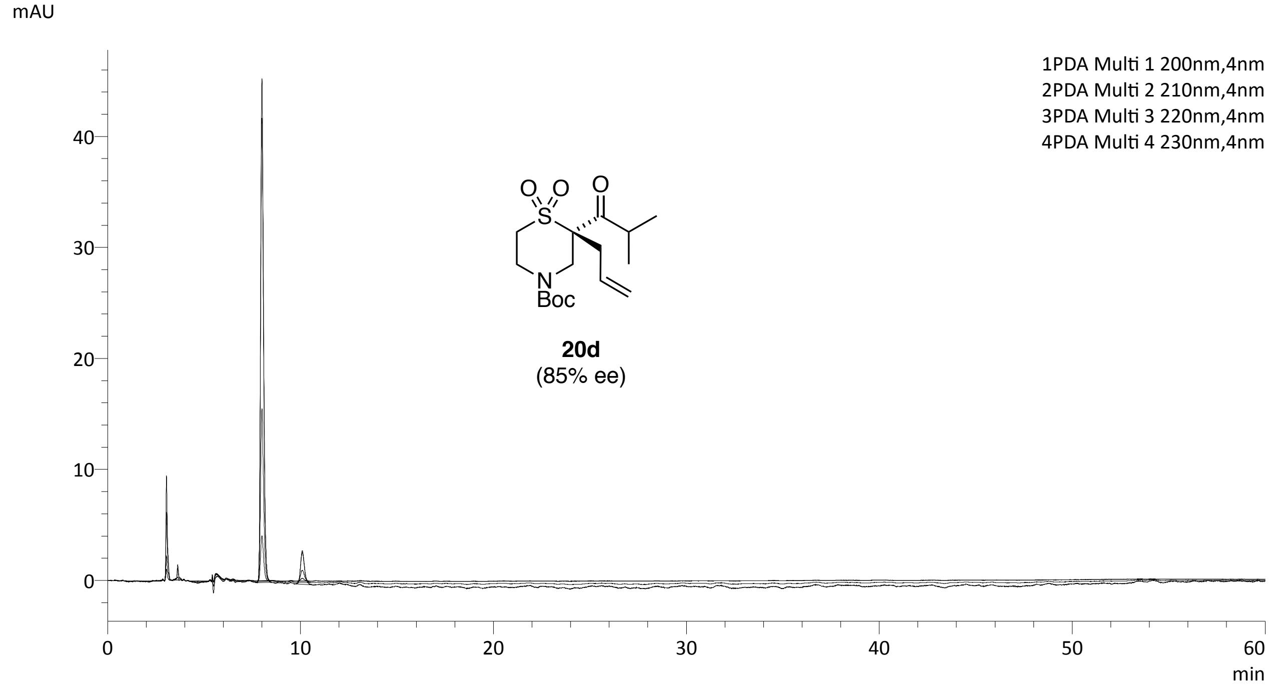

Peak Table

PDA Ch1 200nm

Name	Peak#	Ret. Time	Area	Area%	Mark
	1	8.000	489648	91.916	M
	2	10.095	43067	8.084	M
	Total		532714	100.000	

PDA Ch2 210nm

Name	Peak#	Ret. Time	Area	Area%	Mark
	1	7.999	448724	92.460	M
	2	10.093	36595	7.540	M
	Total		485318	100.000	

PDA Ch3 220nm

Name	Peak#	Ret. Time	Area	Area%	Mark
	1	7.999	166326	92.692	M
	2	10.093	13114	7.308	M
	Total		179440	100.000	

PDA Ch4 230nm

Name	Peak#	Ret. Time	Area	Area%	Mark
	1	8.000	43795	92.605	M
	2	10.107	3497	7.395	M
	Total		47293	100.000	

3. X-Ray Data for 18b

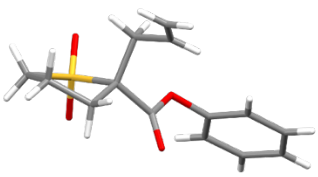

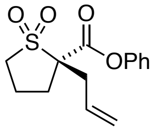

18b

Empirical formula	$C_{14}H_{16}O_4S$
Formula weight	280.33
Temperature/K	100.00(10)
Crystal system	trigonal
Space group	$P3_1$
a/Å	13.51930(12)
b/Å	13.51930(12)
c/Å	6.44771(6)
α/°	90
β/°	90
γ/°	120
Volume/Å^3	1020.57(2)
Z	3
ρ_{calc}g/cm^3	1.368
μ/mm^{-1}	2.191
F(000)	444.0
Crystal size/mm^3	0.589 × 0.26 × 0.139
Radiation	CuKα (λ = 1.54184)
2Θ range for data collection/°	7.55 to 151.842
Index ranges	-16 ≤ h ≤ 16, -16 ≤ k ≤ 16, -8 ≤ l ≤ 7
Reflections collected	13938
Independent reflections	2814 [R_{int} = 0.0348, R_{sigma} = 0.0210]
Data/restraints/parameters	2814/1/172
Goodness-of-fit on F^2	1.056
Final R indexes [I>=2σ (I)]	R_1 = 0.0284, wR_2 = 0.0761
Final R indexes [all data]	R_1 = 0.0284, wR_2 = 0.0761
Largest diff. peak/hole / e Å^{-3}	0.28/-0.32
Flack parameter	0.002(7)

Single crystals were selected and mounted, on a Mitegen loop using Paratone-N oil, on a SuperNova, Dual, Cu at zero, AtlasS2 diffractometer. The crystals were kept at 100(2) K during data collection. Data reduction was performed using CrysAlisPro1.171.38.44a (Rigaku Oxford Diffraction, 2015). Using Olex2,[1] **18b** was solved with the Superflip[2] structure solution program using Charge Flipping and refined with the ShelXL[3] refinement package using Least Squares minimisation.

1. Dolomanov, O.V., Bourhis, L.J., Gildea, R.J, Howard, J.A.K. & Puschmann, H. (2009), J. Appl. Cryst. 42, 339-341.
2. Palatinus, L. & Chapuis, G. (2007). J. Appl. Cryst., 40, 786-790; Palatinus, L. & van der Lee, A. (2008). J. Appl. Cryst. 41, 975-984; Palatinus, L., Prathapa, S. J. & van Smaalen, S. (2012). J. Appl. Cryst. 45, 575-580.
3. Sheldrick, G.M. (2008). Acta Cryst. A64, 112-122.

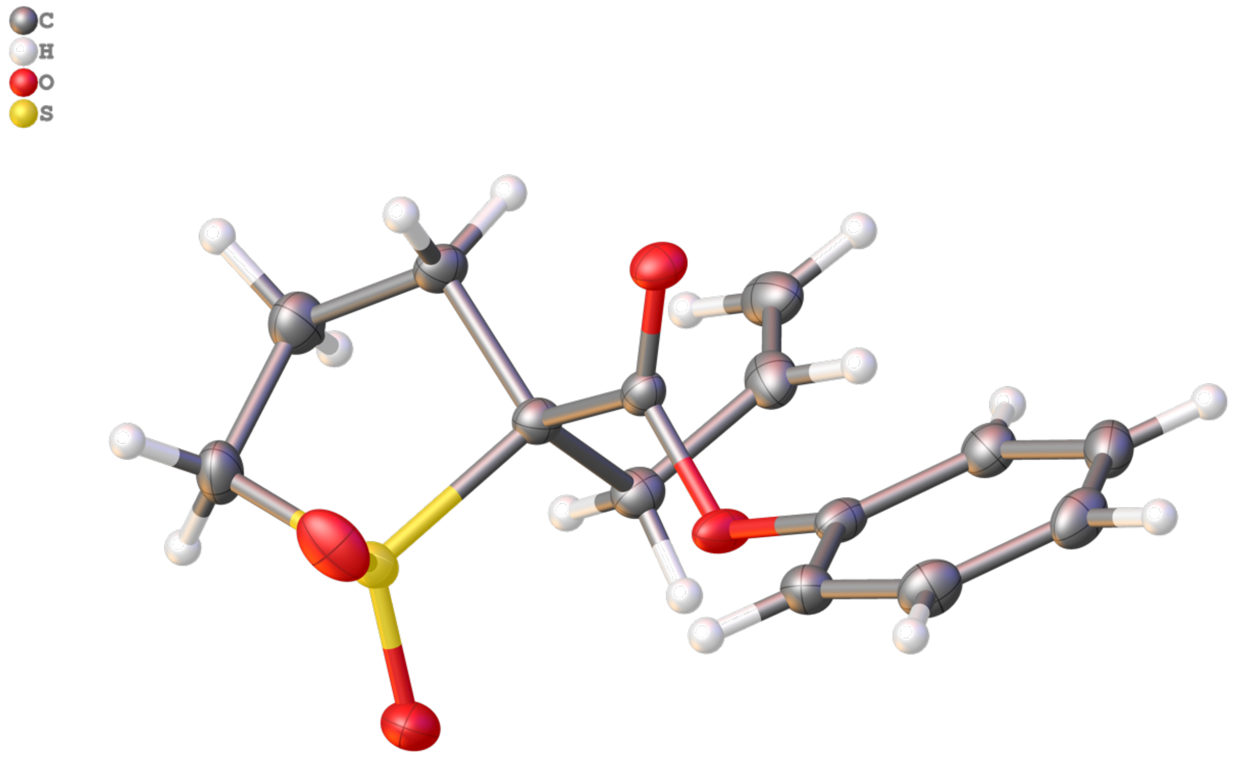

4. Mechanistic Study.

4.1. Effect of Enolate Geometry.

Conditions: [$Pd_2(dba)_3$] (5 mol%), (*S*,*S*)-**L4** (13 mol%), 1,4-dioxane, rt, 2 h

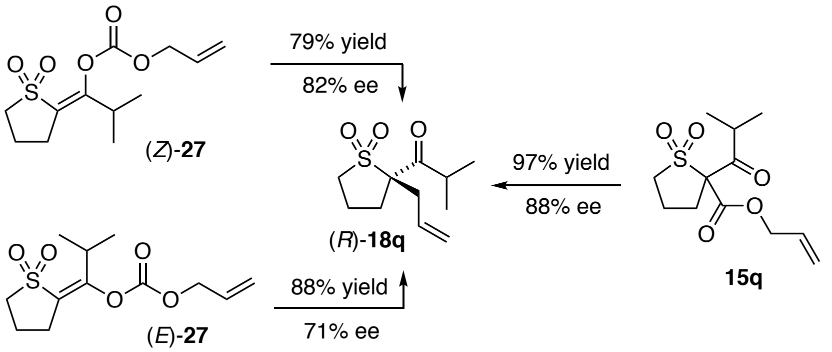

A vial was charged with (*Z*)-**27** (59 mg, 0.22 mmol), [Pd_2dba_3] (9.9 mg, 10.8 μmol), **L4** (22 mg, 28.0 μmol) and 1,4-dioxane (2.2 mL). The mixture was stirred at room temperature for 2 h, then concentrated under reduced pressure. Purification by flash column chromatography [hexane:EtOAc 6:1] gave **18q** (39 mg, 79%) as a colourless oil. HPLC: 82% ee (Chiralcel OD-H, hexane:*i*-PrOH = 95:5, flow rate = 1 mL/min, 30.0 °C, λ = 210 nm) t_R = 10.3 min (minor), t_R = 12.2 min (major).

A vial was charged with (*E*)-**27** (82 mg, 0.30 mmol), [Pd_2dba_3] (13.7 mg, 15.0 μmol), **L4** (32 mg, 39.0 μmol) and 1,4-dioxane (3 mL). The mixture was stirred at room temperature for 2 h, then concentrated under reduced pressure. Purification by flash column chromatography [hexane:EtOAc 6:1] gave **18q** (61 mg, 88%) as a colourless oil. HPLC: 71% ee (Chiralcel OD-H, hexane:*i*-PrOH = 95:5, flow rate = 1 mL/min, 30.0 °C, λ = 210 nm) t_R = 10.3 min (minor), t_R = 12.2 min (major).

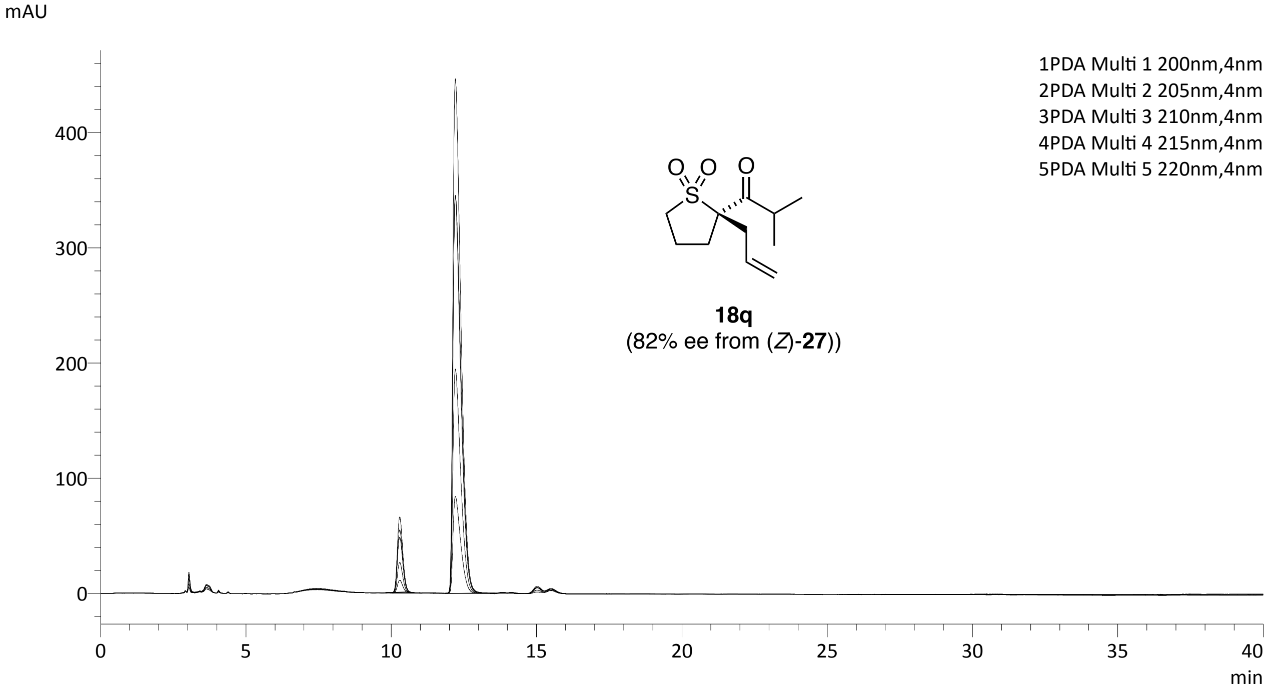

Peak Table

PDA Ch1 200nm

Name	Peak#	Ret. Time	Area	Area%	Mark
	1	10.292	719415	9.553	M
	2	12.209	6811683	90.447	
	Total		7531098	100.000	

PDA Ch2 205nm

Name	Peak#	Ret. Time	Area	Area%	Mark
	1	10.293	841756	8.922	M
	2	12.209	8593127	91.078	
	Total		9434883	100.000	

PDA Ch3 210nm

Name	Peak#	Ret. Time	Area	Area%	Mark
	1	10.293	635285	8.898	
	2	12.209	6504271	91.102	
	Total		7139556	100.000	

PDA Ch4 215nm

Name	Peak#	Ret. Time	Area	Area%	Mark
	1	10.293	349986	8.775	
	2	12.209	3638642	91.225	
	Total		3988628	100.000	

PDA Ch5 220nm

Name	Peak#	Ret. Time	Area	Area%	Mark
	1	10.293	148097	8.657	
	2	12.209	1562553	91.343	
	Total		1710650	100.000	

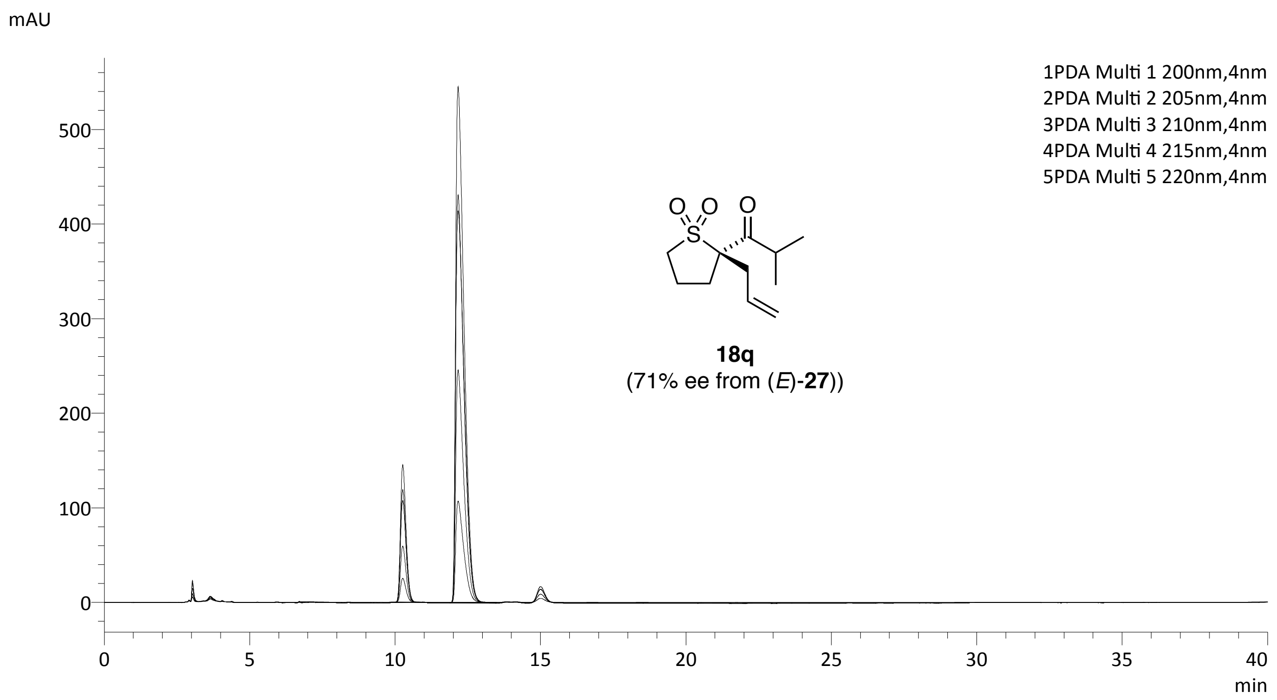

Peak Table

PDA Ch1 200nm

Name	Peak#	Ret. Time	Area	Area%	Mark
	1	10.264	1608192	15.614	M
	2	12.167	8691717	84.386	V
	Total		10299909	100.000	

PDA Ch2 205nm

Name	Peak#	Ret. Time	Area	Area%	Mark
	1	10.264	1945395	14.933	M
	2	12.167	11081991	85.067	
	Total		13027386	100.000	

PDA Ch3 210nm

Name	Peak#	Ret. Time	Area	Area%	Mark
	1	10.264	1448498	14.564	S
	2	12.167	8497266	85.436	
	Total		9945764	100.000	

PDA Ch4 215nm

Name	Peak#	Ret. Time	Area	Area%	Mark
	1	10.264	791867	14.206	
	2	12.167	4782407	85.794	
	Total		5574273	100.000	

PDA Ch5 220nm

Name	Peak#	Ret. Time	Area	Area%	Mark
	1	10.264	331109	13.791	
	2	12.167	2069830	86.209	
	Total		2400939	100.000	

4.2. Enolate Crossover.

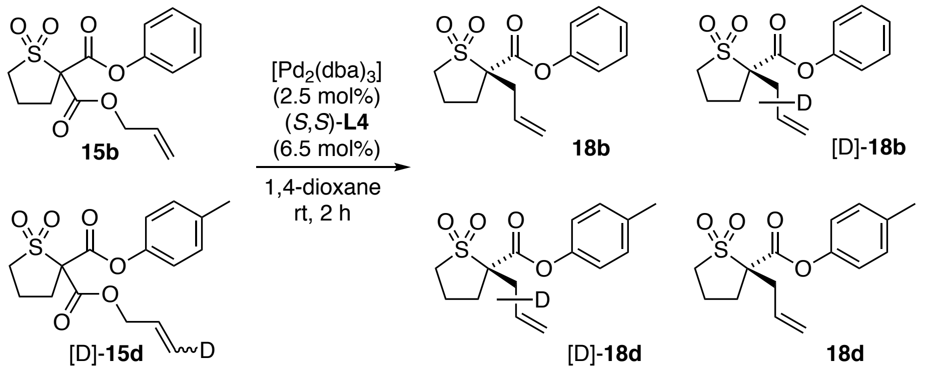

A vial was charged with **15b** (49 mg, 0.15 mmol), [D]-**15d** (52 mg, 0.15 mmol), [Pd_2dba_3] (6.9 mg, 7.5 μmol), **L4** (15.9 mg, 19.5 μmol) and 1,4-dioxane (3 mL). The mixture was stirred at room temperature for 2 h, then concentrated under reduced pressure. Purification by flash column chromatography [hexane:EtOAc 4:1] gave an inseparable mixture of **18b**, [D]-**18b**, [D]-**18d** and **18d**, each of which was detected by high resolution mass spectrometry.

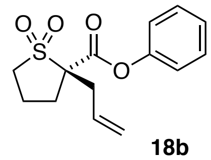

18b

Event#: 1 MS(C+) Ret. Time : 0.127 -> 0.317 - 0.095 -> 0.965 Scan# : 17 -> 41 - 13 -> 123

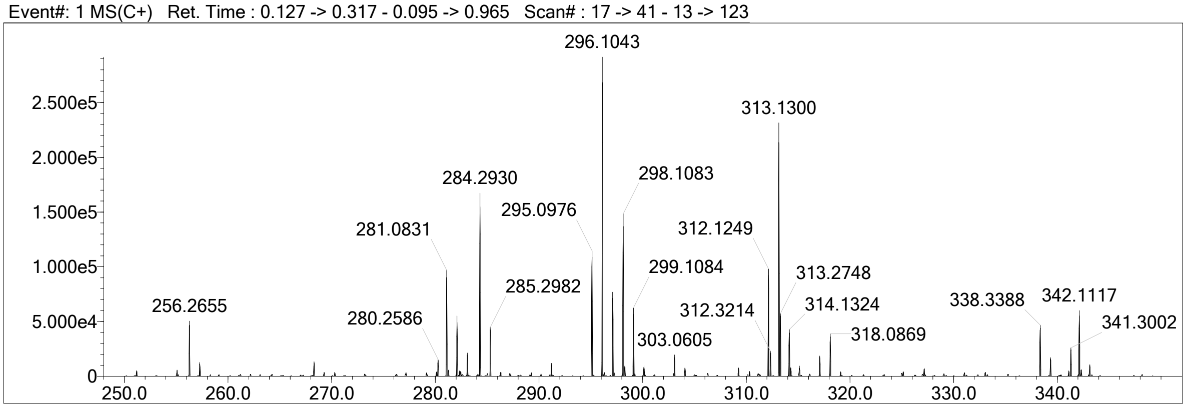

Measured region for 281.0831 m/z

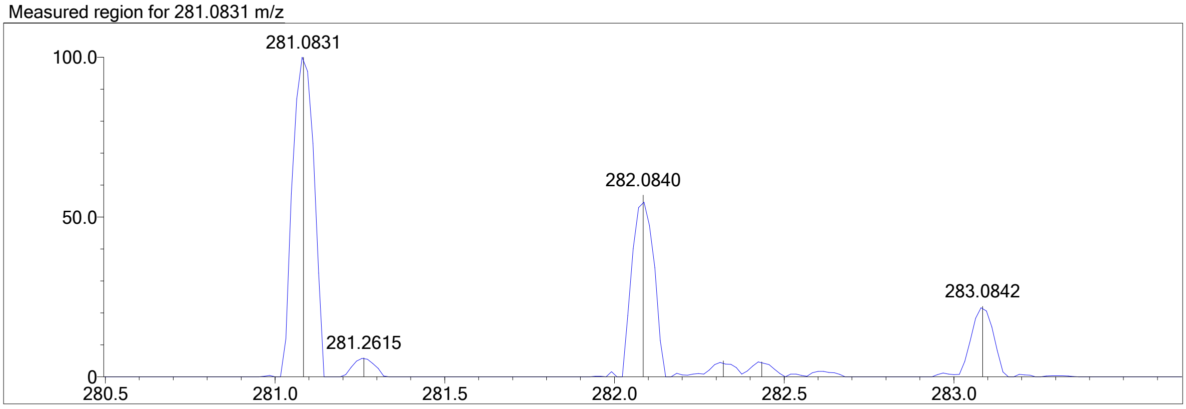

C14 H16 O4 S [M+H]+ : Predicted region for 281.0842 m/z

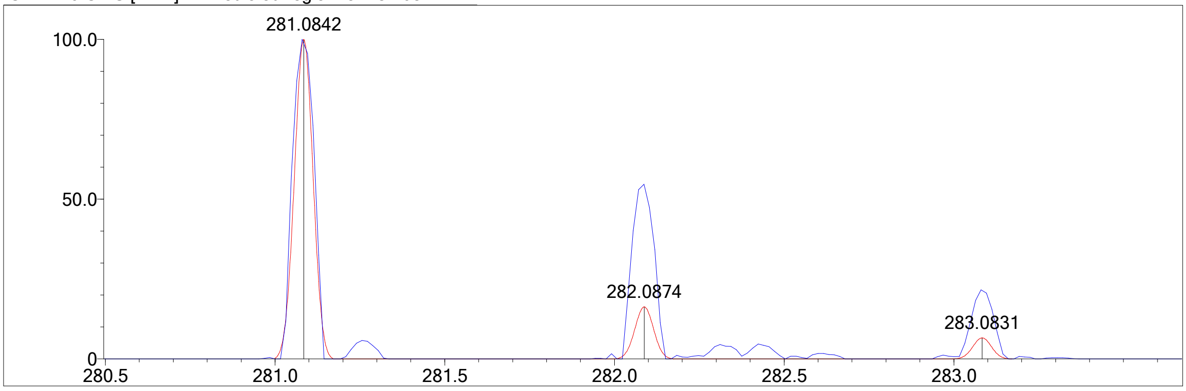

Rank	Score	Formula (M)	Ion	Meas. m/z	Pred. m/z	Df. (mDa)	Df. (ppm)	Iso	DBE
2	46.36	C14 H16 O4 S	[M+H]+	281.0831	281.0842	-1.1	-3.91	50.00	7.0

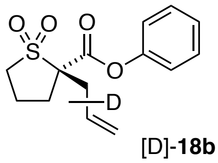

[D]-**18b**

Event#: 1 MS(C+) Ret. Time : 0.206 - 0.016 -> 0.998 Scan# : 27 - 3 -> 127

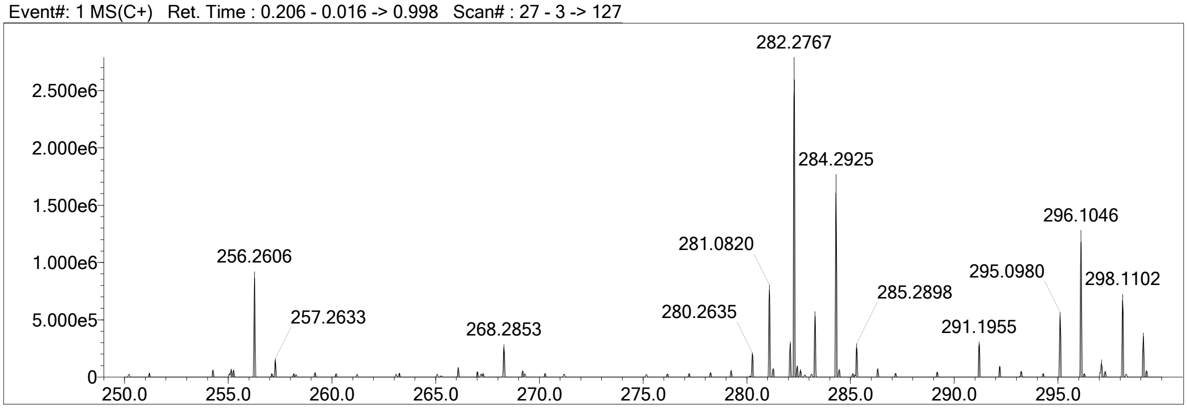

Measured region for 281.0820 m/z

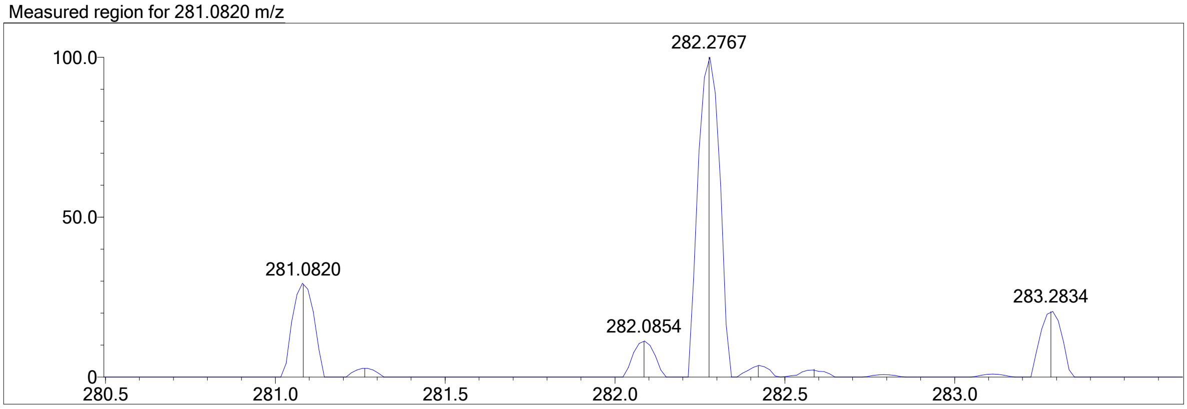

C14 H15 2H O4 S M+ : Predicted region for 281.0827 m/z

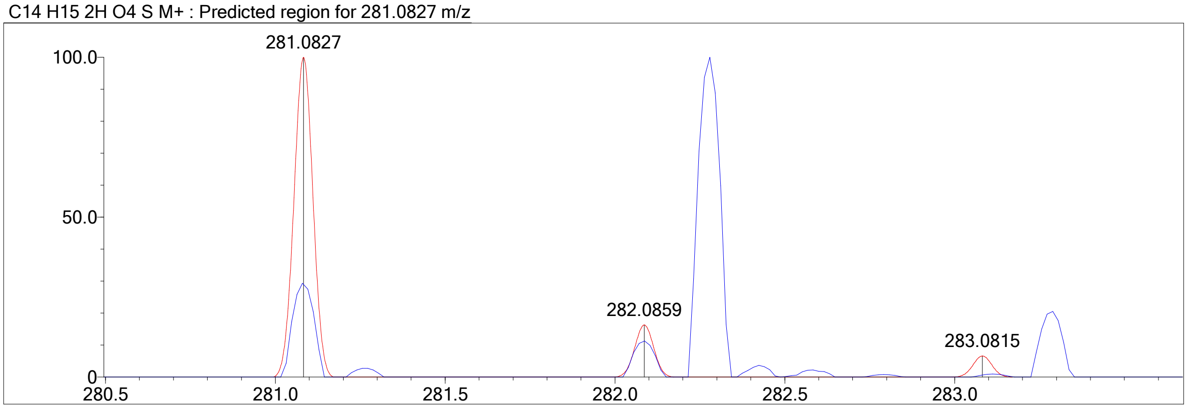

Rank	Score	Formula (M)	Ion	Meas. m/z	Pred. m/z	Df. (mDa)	Df. (ppm)	Iso	DBE
1	14.05	C14 H15 2H O4 S	M+	281.0820	281.0827	-0.7	-2.49	14.59	7.0

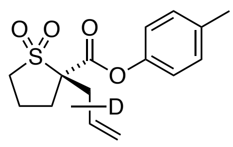

[D]-**18d**

Event#: 1 MS(C+) Ret. Time : 0.222 - 0.475 -> 0.998 Scan# : 29 - 61 -> 127

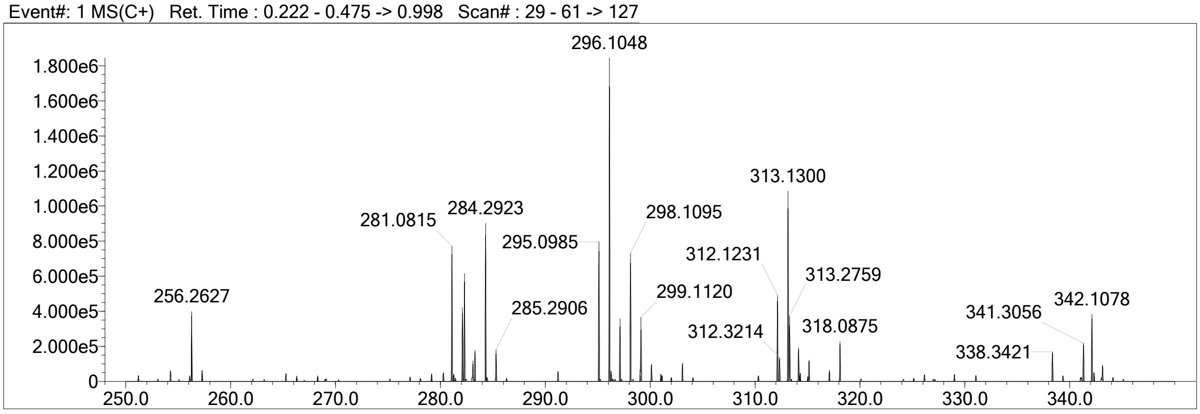

Measured region for 296.1048 m/z

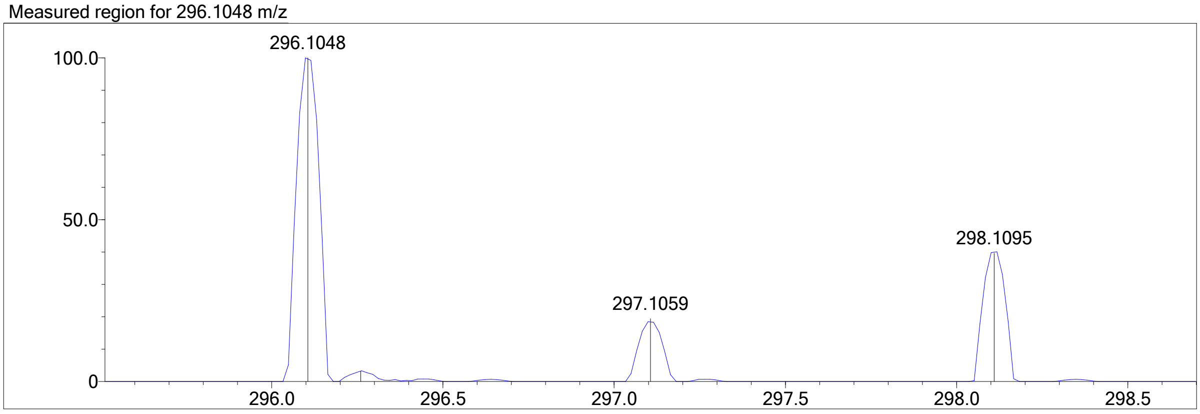

C15 H17 2H O4 S [M+H]+ : Predicted region for 296.1061 m/z

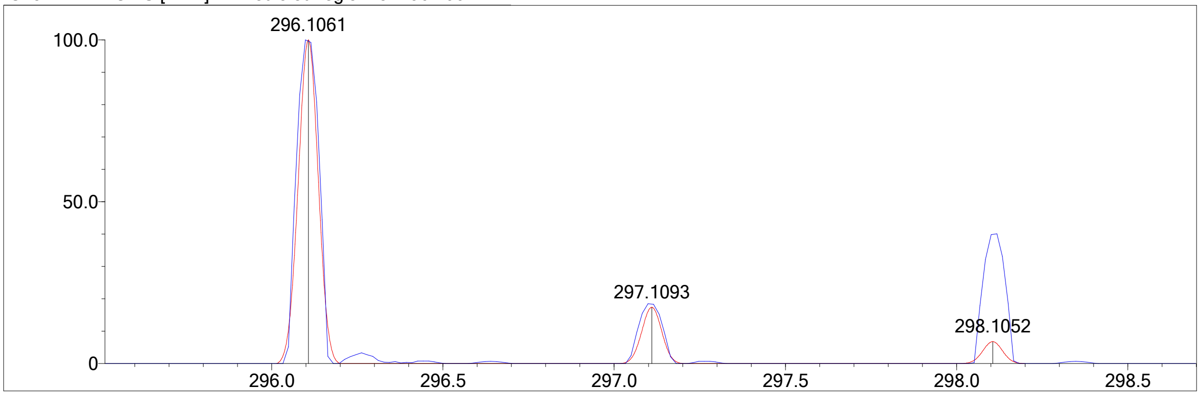

Rank	Score	Formula (M)	Ion	Meas. m/z	Pred. m/z	Df. (mDa)	Df. (ppm)	Iso	DBE
3	45.76	C15 H17 2H O4 S	[M+H]+	296.1048	296.1061	-1.3	-4.39	50.00	7.0

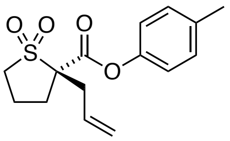

18d

Event#: 1 MS(C+) Ret. Time : 0.222 - 0.475 -> 0.998 Scan# : 29 - 61 -> 127

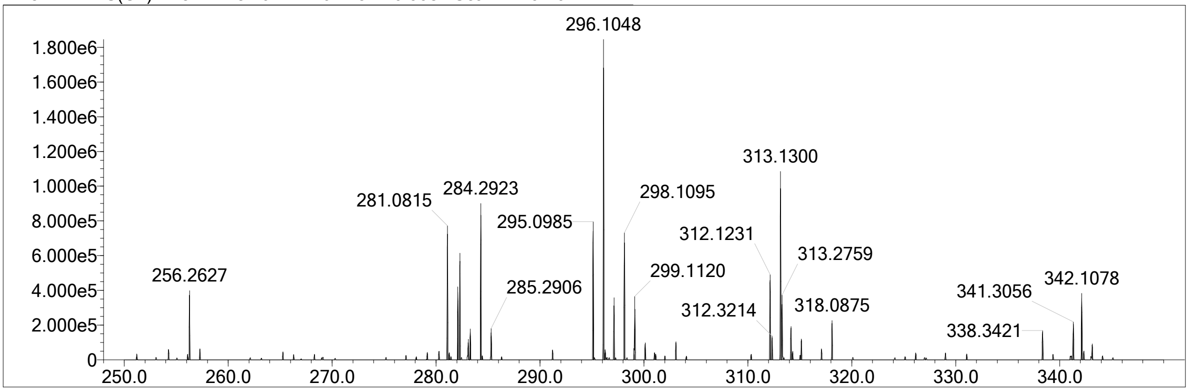

Measured region for 295.0985 m/z

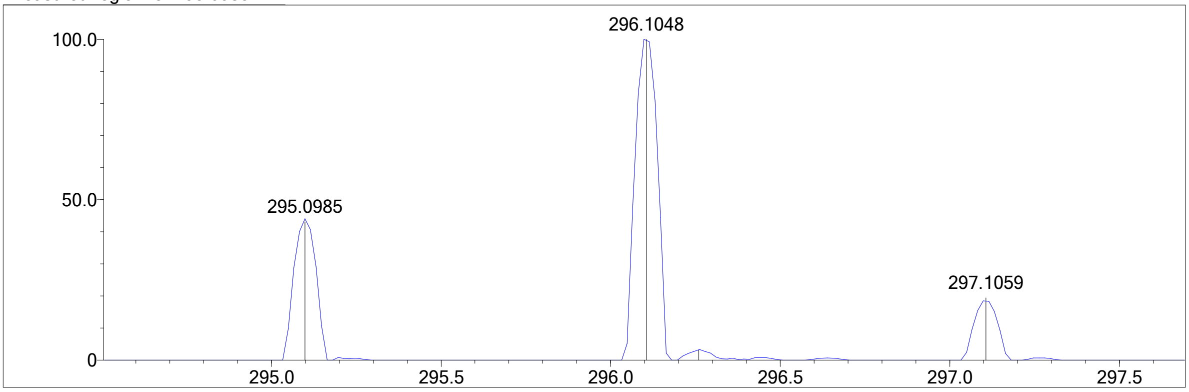

C15 H18 O4 S [M+H]+ : Predicted region for 295.0999 m/z

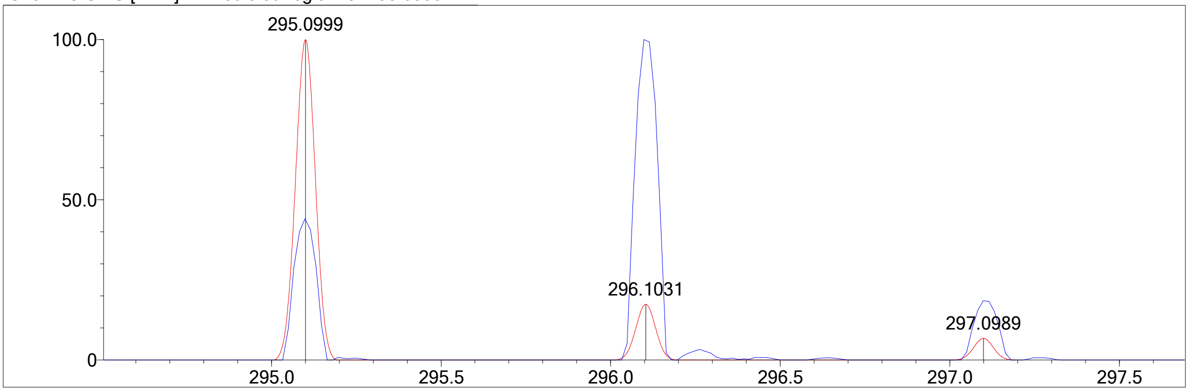

Rank	Score	Formula (M)	Ion	Meas. m/z	Pred. m/z	Df. (mDa)	Df. (ppm)	Iso	DBE
1	43.23	C15 H18 O4 S	[M+H]+	295.0985	295.0999	-1.4	-4.74	47.69	7.0

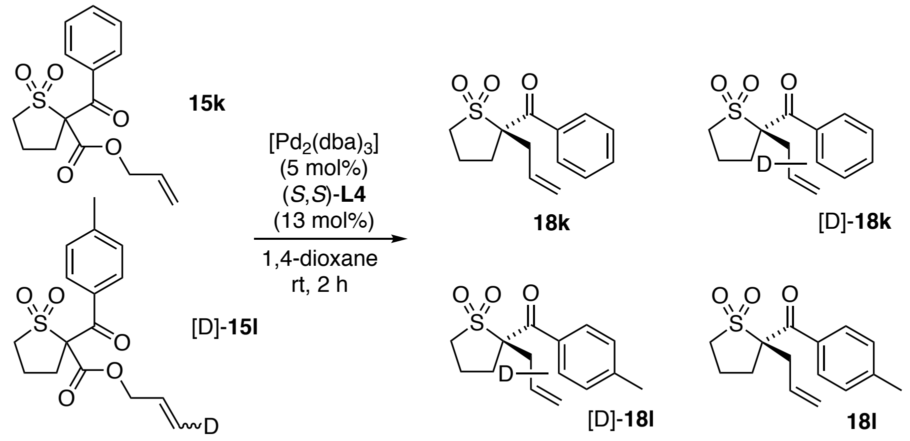

A vial was charged with **15k** (20 mg, 0.065 mmol), [D]-**15l** (21 mg, 0.065 mmol), [Pd_2dba_3] (6.0 mg, 6.5 μmol), **L4** (13.7 mg, 16.9 μmol) and 1,4-dioxane (1.3 mL). The mixture was stirred at room temperature for 2 h, then concentrated under reduced pressure. Purification by flash column chromatography [hexane:EtOAc 4:1] gave an inseparable mixture of **18k**, [D]-**18k**, [D]-**18l** and **18l**, each of which was detected by high resolution mass spectrometry.

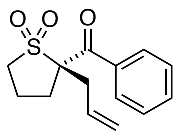

18k

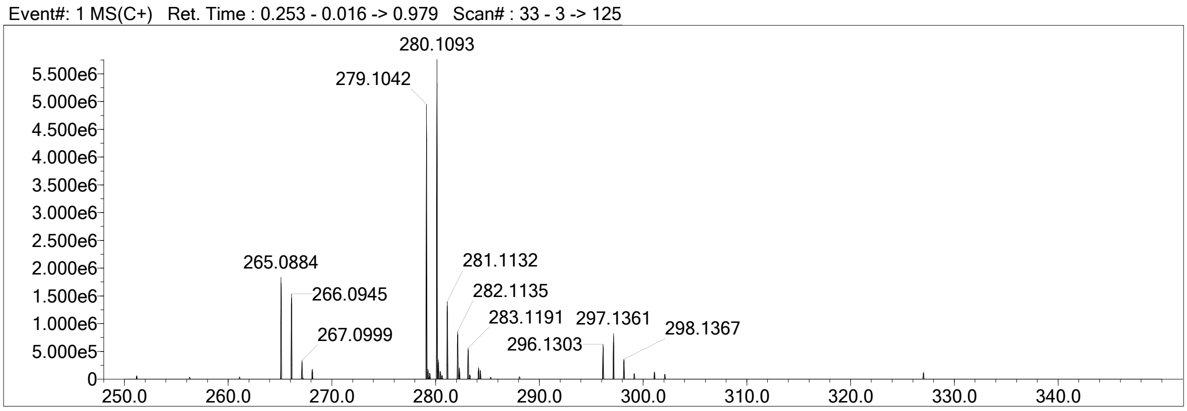

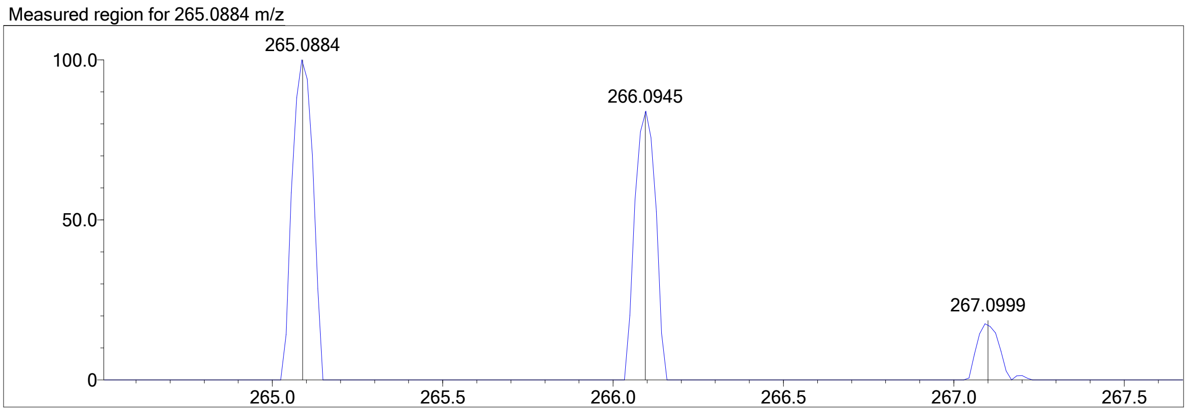

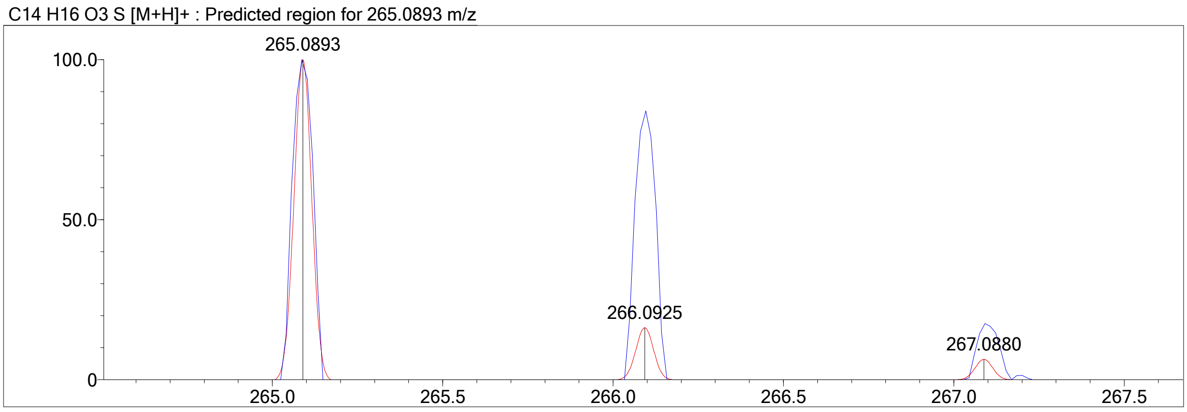

Rank	Score	Formula (M)	Ion	Meas. m/z	Pred. m/z	Df. (mDa)	Df. (ppm)	Iso	DBE
1	29.80	C14 H16 O3 S	[M+H]+	265.0884	265.0893	-0.9	-3.40	31.71	7.0

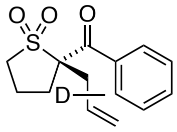

[D]-**18k**

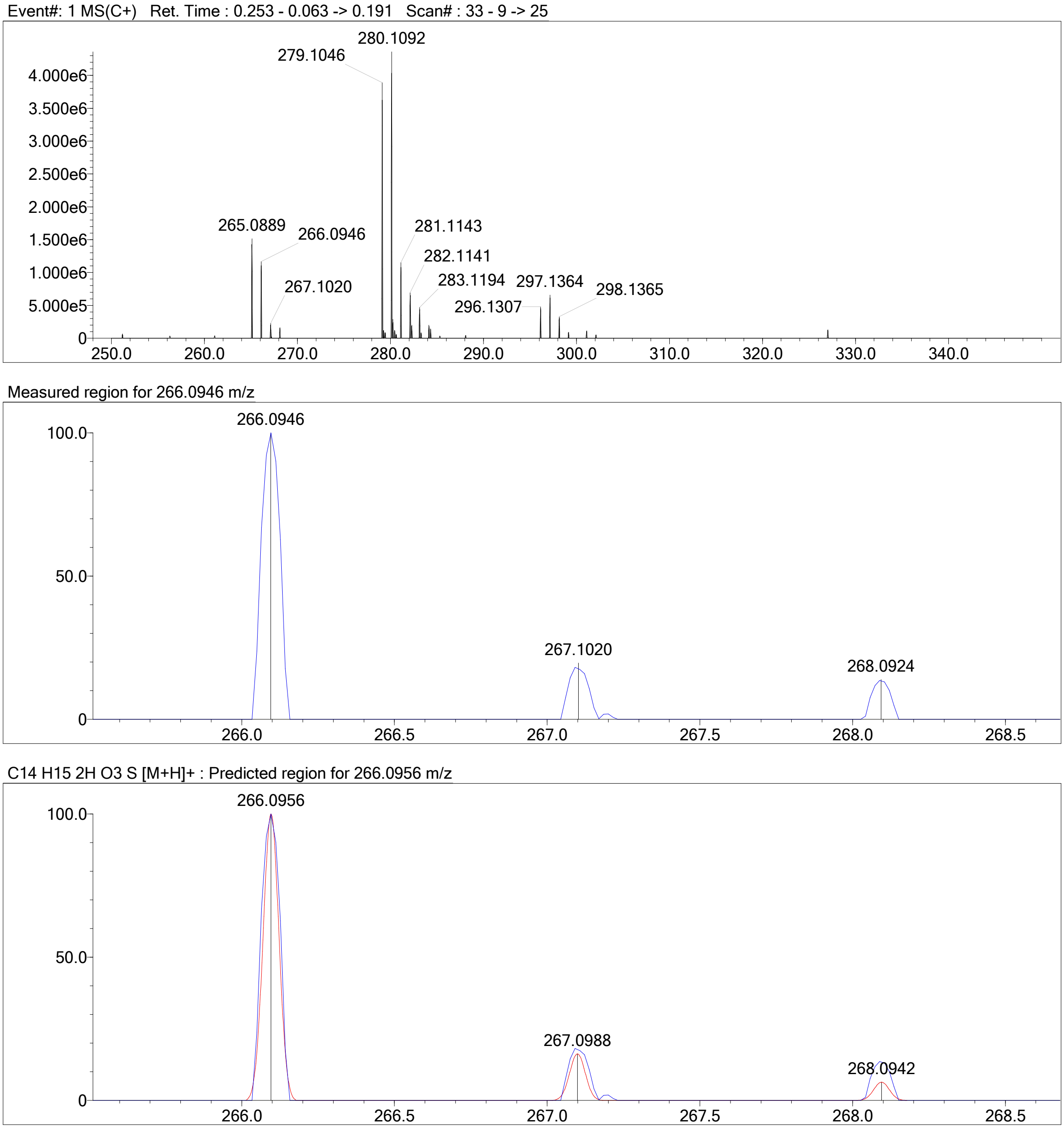

Rank	Score	Formula (M)	Ion	Meas. m/z	Pred. m/z	Df. (mDa)	Df. (ppm)	Iso	DBE
1	66.98	C14 H15 2H O3 S	[M+H]+	266.0946	266.0956	-1.0	-3.76	71.95	7.0

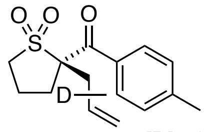

[D]-**18l**

Event#: 1 MS(C+) Ret. Time : 0.253 - 0.143 Scan# : 33 - 19

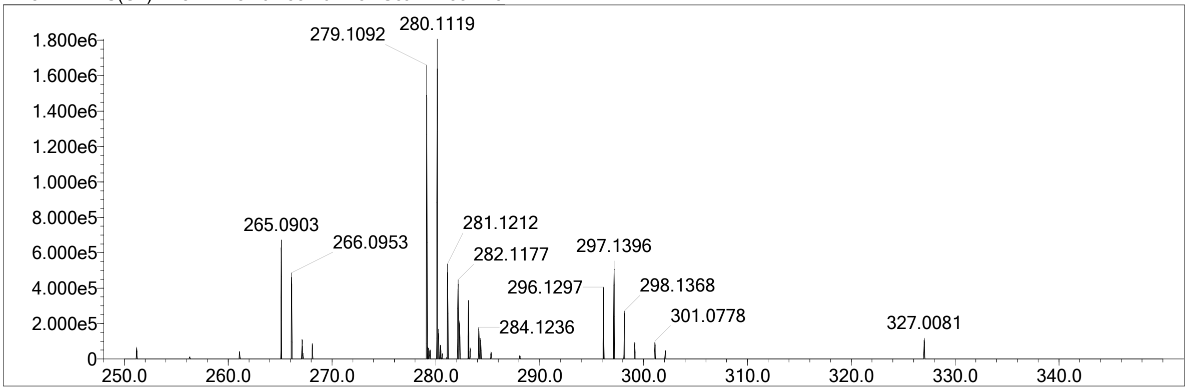

Measured region for 280.1119 m/z

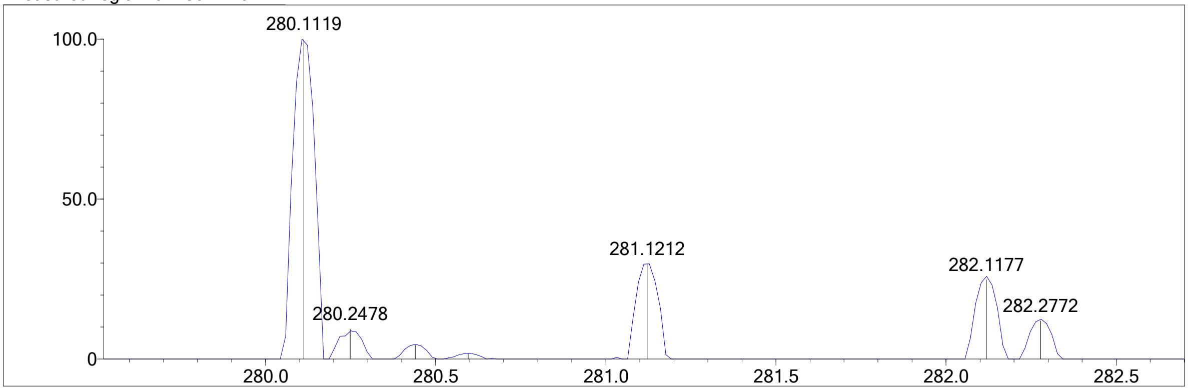

C15 H17 2H O3 S [M+H]+ : Predicted region for 280.1112 m/z

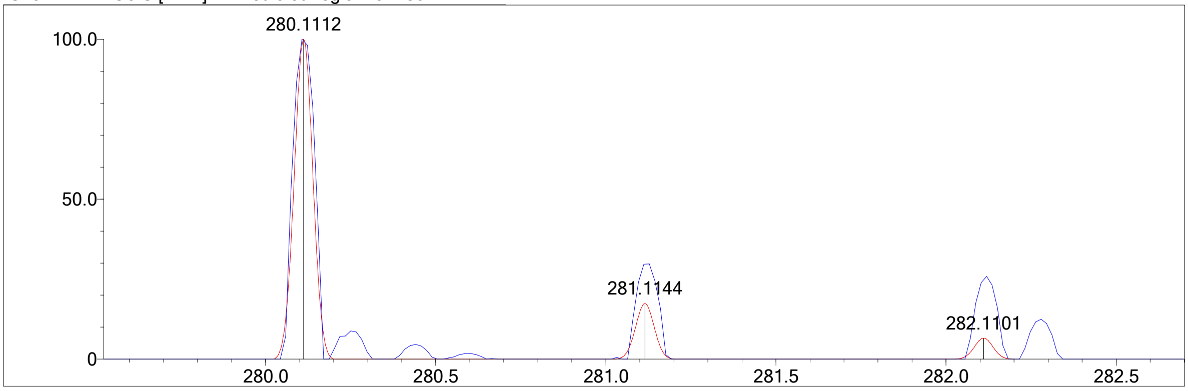

Rank	Score	Formula (M)	Ion	Meas. m/z	Pred. m/z	Df. (mDa)	Df. (ppm)	Iso	DBE
1	36.59	C15 H17 2H O3 S	[M+H]+	280.1119	280.1112	0.7	2.50	38.01	7.0

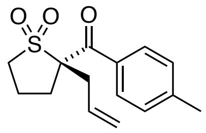

18l

Event#: 1 MS(C+) Ret. Time : 0.253 - 0.063 -> 0.191 Scan# : 33 - 9 -> 25

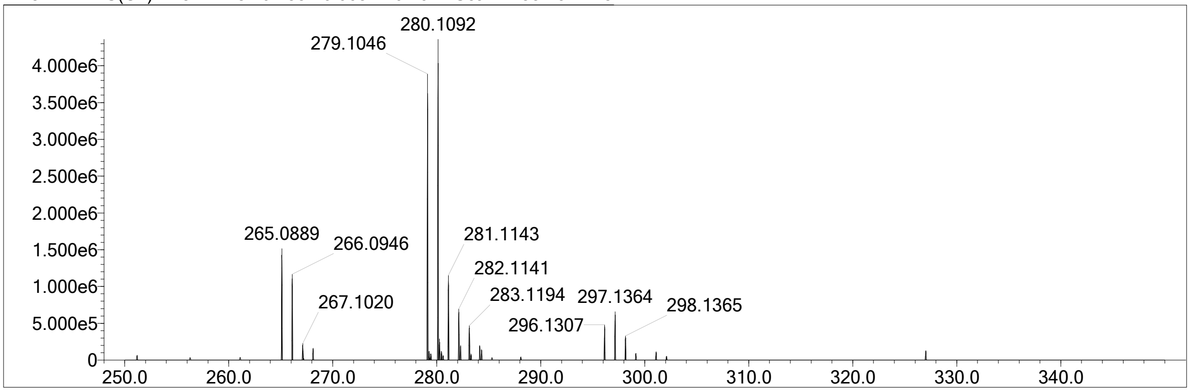

Measured region for 279.1046 m/z

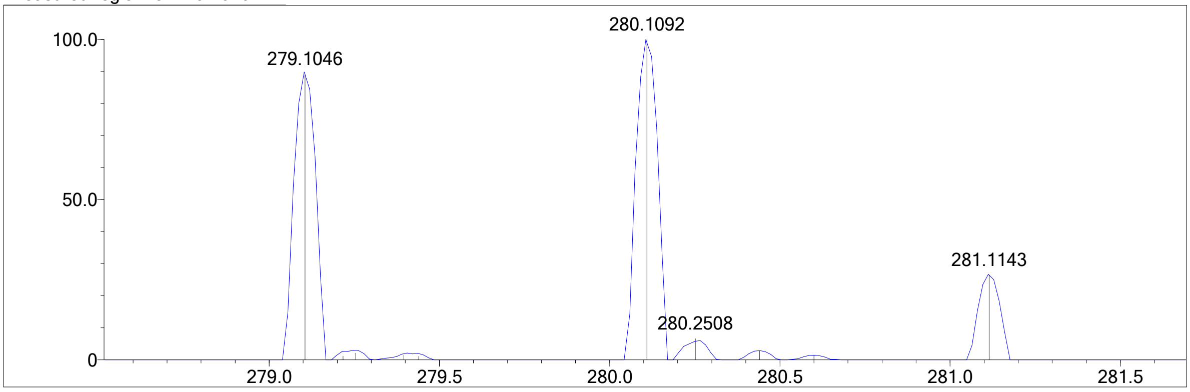

C15 H18 O3 S [M+H]+ : Predicted region for 279.1049 m/z

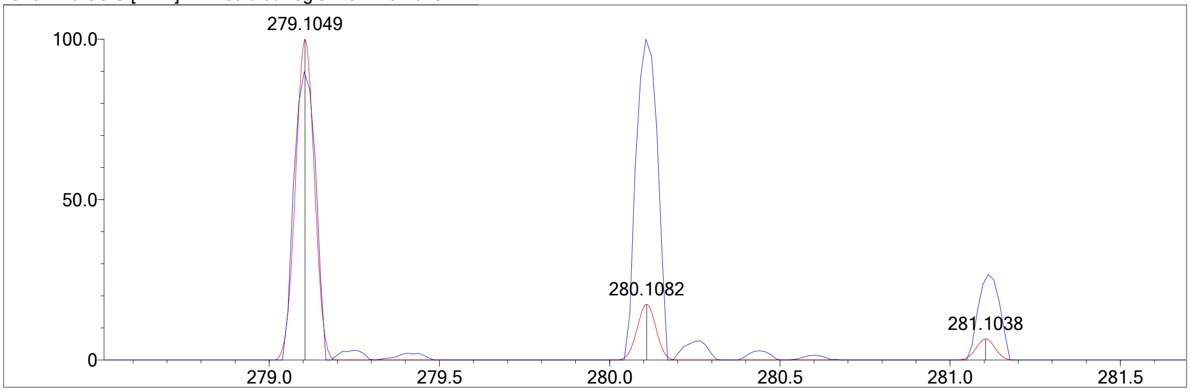

Rank	Score	Formula (M)	Ion	Meas. m/z	Pred. m/z	Df. (mDa)	Df. (ppm)	Iso	DBE
1	36.81	C15 H18 O3 S	[M+H]+	279.1046	279.1049	-0.3	-1.07	36.88	7.0

4.3. Competing Allylation.

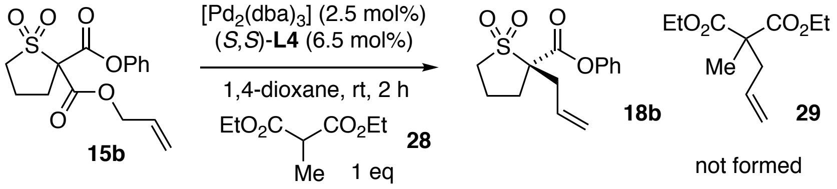

A vial was charged with **15b** (48.5 mg, 0.15 mmol), [Pd2dba3] (3.5 mg, 3.75 μmol), **L4** (8.0 mg, 9.75 μmol), diethyl methylmalonate (0.026 mL, 0.15 mmol) and 1,4-dioxane (1.5 mL). The mixture was stirred at room temperature for 2 h, then concentrated under reduced pressure. ^{1}H NMR spectroscopy of the crude product mixture indicated no formation of **29**. Flash column chromatography [petrol:EtOAc 4:1] gave **28** (18 mg, 65% recovery) and **18b** (21 mg, 50%).

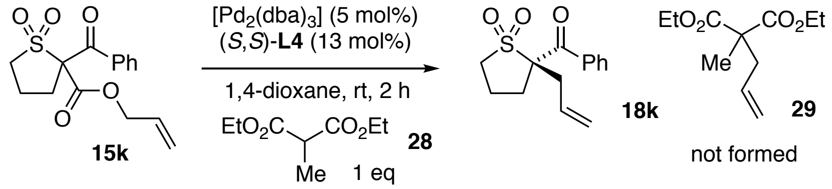

A vial was charged with **15k** (46 mg, 0.15 mmol), [Pd2dba3] (6.9 mg, 7.5 μmol), **L4** (15.9 mg, 19.5 μmol), diethyl methylmalonate (0.026 mL, 0.15 mmol) and 1,4-dioxane (1.5 mL). The mixture was stirred at room temperature for 2 h, then concentrated under reduced pressure. ^{1}H NMR spectroscopy of the crude product mixture indicated no formation of **29**. Flash column chromatography [petrol:EtOAc 4:1] gave **28** (19 mg, 67% recovery) and **18k** (23 mg, 57%).
